# Psychological intervention improves quality of life in patients with early-stage cancer: a systematic review and meta-analysis of randomized clinical trials

**DOI:** 10.1038/s41598-024-63431-y

**Published:** 2024-06-09

**Authors:** Sára Anna Bognár, Brigitta Teutsch, Stefania Bunduc, Dániel Sándor Veres, Bence Szabó, Beatrix Fogarasi, Olga Júlia Zahariev, Nóra Vörhendi, Omer Almog, Yael Hadani, Dorottya Gergő, Emese Mihály, Bálint Erőss, Stefania Bunduc, Katalin Márta, Péter Hegyi

**Affiliations:** 1 Institute of Pancreatic Diseases, Semmelweis University, 1083 Budapest, Hungary; 2 Centre for Translational Medicine, Semmelweis University, 1085 Budapest, Hungary; 3 Institute for Translational Medicine, Medical School, University of Pécs, 7623 Pecs, Hungary; 4 Department of Biophysics and Radiation Biology, Semmelweis University, 1085 Budapest, Hungary; 5 Department of Internal Medicine, Siófok City Hospital and Outpatient Clinic, 8601 Siófok, Hungary; 6 Department of Pharmacognosy, Semmelweis University, Üllői út 26, 1085 Budapest, Hungary; 7 Department of Internal Medicine and Hematology, Medical School, Semmelweis University, 1088 Budapest, Hungary; 8 Center for Digestive Diseases and Liver Transplant, Fundeni Clinical Institute, 022328 Bucharest, Romania; 9 Translational Pancreatology Research Group, Interdisciplinary Centre of Excellence for Research Development and Innovation University of Szeged, 6725 Szeged, Hungary

**Keywords:** Neoplasm, Survival, Quality of life, Psychosocial intervention, Cancer, Psychology, Health care, Oncology

## Abstract

The effectiveness of psychological interventions (PI) for malignant diseases is controversial. We aimed to investigate the effect of PI on survival and quality of life (QoL) in patients with cancer. We performed a systematic search of MEDLINE, Cochrane, and Embase databases to identify randomized controlled trials comparing PI to standard care (PROSPERO registration number CRD42021282327). Outcomes were overall survival (OS), recurrence-free survival (RFS), and different domains of QoL. Subgroup analysis was performed based on the provider-, type-, environment-, duration of intervention; cancer stage, and type. Pooled hazard ratios (HR) and standardized mean difference (SMD) with 95% confidence intervals (CI) were calculated using a random-effects model. The OS and RFS did not differ significantly between the two groups (OS:HR = 0.97; CI 0.87–1.08; RFS:HR = 0.99; CI 0.84–1.16). However, there was significant improvement in the intervention group in all the analyzed domains of QoL; in the global (SMD = 0.65; CI 0.35–0.94), emotional (SMD = 0.64; CI 0.33–0.95), social (SMD = 0.32; CI 0.13–0.51) and physical (SMD = 0.33; CI 0.05–0.60) domains. The effect of PI on QoL was generally positive immediately, 12 and 24 weeks after intervention, but the effect decreased over time and was no longer found significant at 48 weeks. The results were better in the breast cancer group and early stages of cancer. PIs do not prolong survival, but they significantly improve the QoL of cancer patients. PI should be added as standard of care 3–4 times a year, at least for patients with early-stage cancer.

## Introduction

Cancer is a leading cause of death and reduces life expectancy worldwide^1^. GLOBOCAN estimated that there were 19.3 million new cancer cases, and almost 10 million led to death in 2020, irrespective of the world region^2^. In Europe, the most common causes of cancer-related deaths are lung (380,000 deaths, one-fifth of the total), colorectal (250,000 deaths, 12.6%), breast (140,000, 7.3%; females only) and pancreatic (130,000, 6.8%) cancers. Altogether, these cancers account for 47% of all cancer-related mortality. The disease affects not only a large number of patients but also their families and the healthcare system, taking a psychological, physical, and financial toll^3^.

Survival used to be almost the only goal of cancer treatment; however, quality of life (QoL) is increasingly recognized as an essential outcome criterion for oncological treatments and has been linked to survival prediction^4^. Receiving a cancer diagnosis unquestionably has a negative impact on the QoL, which is related to the prognosis of the disease itself, the choice of treatment, and the duration of the disease. The need for frequent hospitalizations, negative emotions, and several symptoms significantly reduce the QoL of these patients^5^. For these reasons, the need for psychosocial interventions to treat and support these patients, as well as cancer survivors, has increased recently^6^.

The exact effect of how psychological interventions can improve QoL and prolong survival is inconclusive. Existing studies are based on small samples and are inconsistent regarding intervention provider, delivery methods, intervention type, duration and intensity, follow-up measurement periods, and methodological quality^7–10^.

Targeting this gap in existing literature, we aimed to investigate the impact of psychological interventions on the survival rate and QoL of patients with cancer and to present this information in a comprehensive study that breaks down this impact based on the provider, environment, type, and the duration of the psychological intervention, as well as on cancer type and stage.

## Methods

In this study, we worked from independent primary studies that consisted of data that were further analyzed here. We gathered data and analyzed it with statistical methods to derive conclusions in this article. The recommendations of PRISMA 2020^11^ and Cochrane Handbook guidelines were applied (PROSPERO registration number CRD42021282327)^12^.

### Search strategy and eligibility criteria

We formulated our clinical question and defined the search strategy using the PICO-S (population—cancer patients; intervention—psychological intervention; comparison—no psychological intervention; outcome—OS, RFS and QoL, study type—RCTs) framework. The systematic search was performed in three databases: MEDLINE (via PubMed), Cochrane Library (CENTRAL), and EMBASE from inception until 18th October 2021, with the restrictions to include adult patients. The search was updated on the 1st of February 2024 during the revision process. The search key (Supplementary Material (referred to as S.) Table S1) with the following main concepts: psychotherapy AND cancer AND randomized—was used to identify the eligible articles according to predefined criteria. We also screened the reference lists of the identified articles for further eligible reports.

Only randomized controlled trials (RCTs) were considered eligible; adult patients diagnosed with any cancer receiving cancer or palliative treatment or who reported as 'cancer survivors' were eligible for our study. Patients who received psychological interventions were included in our analysis if data on their survival (overall (OS), recurrence-free (RFS)), or QoL were provided. Regarding the psychological interventions, various methods were eligible for the study: different types of psychotherapy, mindfulness, cognitive-behavioral therapy, relaxation, meditation, stress management, self-help, psychoeducation, and counseling carried out in a group, individual or guided self-help settings; face-to-face, on the telephone, or via the internet. Interventions carried out by psychologists, nurses, or any non-specified healthcare professional were also eligible. We included only full-text publications in peer-reviewed journals.

### Data extraction

**Figure 1:**
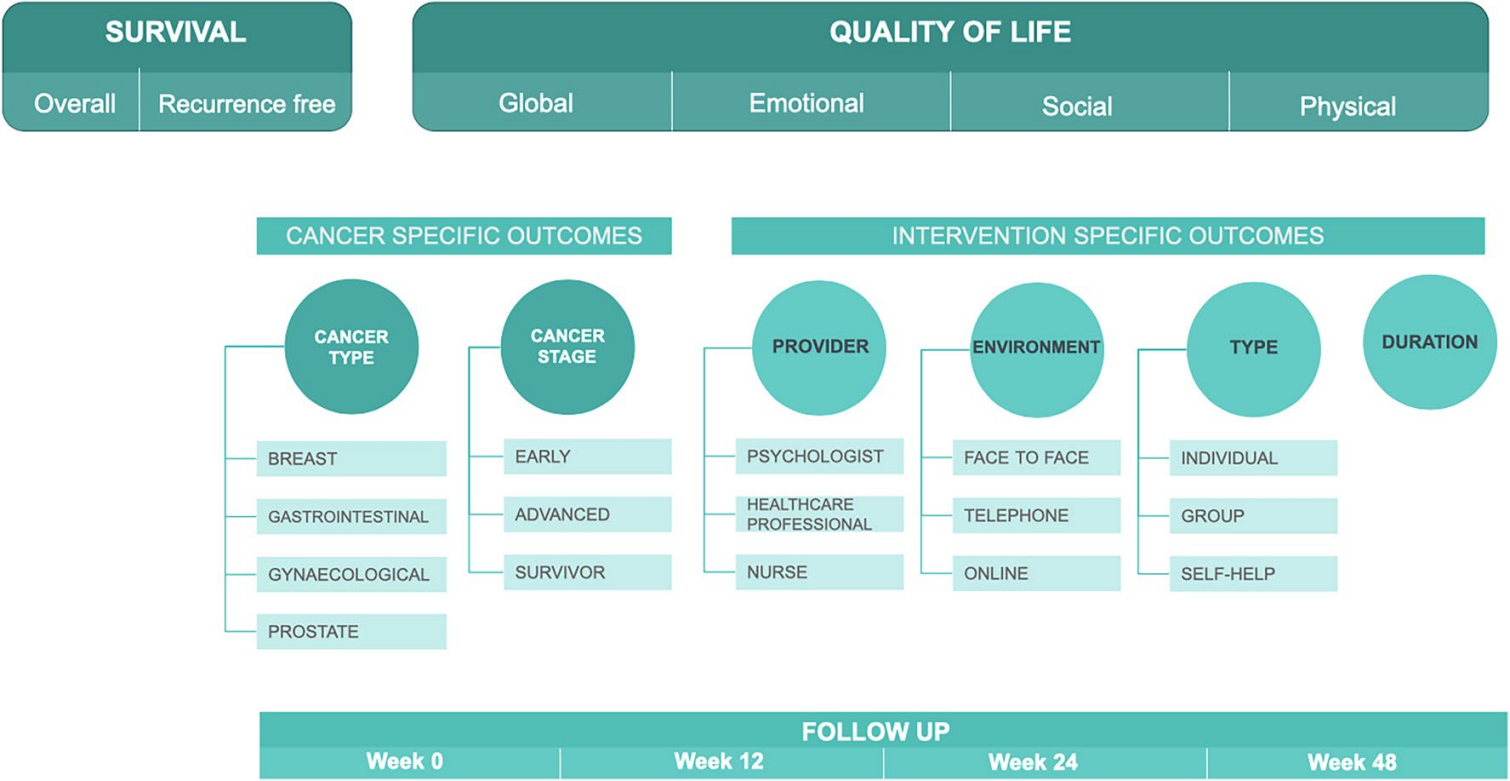
Structure of our analysis. Details of the investigated outcomes (survival and QoL), based on subgroups, and subcategories.

The duplication removal was performed using reference manager software (EndNote X9, Clarivate Analytics, Philadelphia, PA, USA). The titles, abstracts, and full texts were selected by two independent authors in pairs (DG and ASB). The possible disagreements were resolved by a third party (BF). To measure inter-rater reliability, Cohen's kappa coefficients (κ) were calculated. Studies with overlapping populations and ineligible study designs were excluded. Missing or insufficient data were included in the systematic review. From the eligible articles, data were collected manually by two authors (OA and YH) independently into a standardized data table (Microsoft Excel, Microsoft, Office 365, Redmond, WA, USA), and the disagreements were solved by a third reviewer (DG). The following data were extracted: publication characteristics: first author, year of publication, country of origin, number of centers, demographic characteristics of the study population, intervention characteristics: type, duration, provider, environment, outcomes—overall and progression-free survival, quality of life—measurement tool, time of the measurements in weeks, net minutes of intervention per patient, questionnaire scores (mean, SD)—as reported in each article. The outcomes of our analysis of the subgroups are summarized in Fig. 1. A detailed explanation of the structure is given in Section S1.

### Statistical analysis

As we anticipated considerable between-study heterogeneity, a random-effects model was used to pool effect sizes. For OS and RFS, pooled hazard ratios (HR) with 95% confidence intervals were calculated using an inverse variance weighting method; heterogeneity was estimated using a restricted maximum-likelihood estimator, with the Q profile method for the confidence interval. For QoL, the standardized mean difference (SMD) with a 95% confidence interval (CI) was used as the effect size between the intervention and the control groups due to differences in the questionnaires measuring the outcomes. We used Hedges' g to calculate SMDs^13^. SMD values of 0.2–0.5 were considered small effects, 0.5–0.8 were considered medium effects, and values > 0.8 were considered large effects. As the articles reported QoL results at different follow-up time points, to make a prediction, we created a longitudinal model for predicting QoL at 0-, 12-, 24-, and 48-weeks post-intervention, using the time and the square of time as predictors of change in time. Forest plots illustrate the results at these specific time points (T0 = week 0, T12 = week 12, T24 = week 24, T48 = week 48). A separate longitudinal prediction-model was applied for different QoL domains and subcategories. The summary plots are used to visualize the calculated overall effects for a given outcome. Inverse variance weighting was used to calculate the pooled SMD in the longitudinal meta-analysis.

Publication bias and small study effects were assessed using funnel plots. All statistical analyses were made with R (v4.2.1)^14^ using the following packages: metafor (v3.4.0)^15^ and clubSandwich (v0.5.8) for model calculations, publication bias and influential assessment, meta (v5.5.0)^16^ for forest plots, ggplot2 (v3.3.6)^17^ for additional prediction plots. A detailed description of our statistical analysis is given in the Supplementary Material in Section S2. We report the results as point estimates of the effect size (lower–upper limit of 95% CI) format.

### Study risk of bias and certainty assessment

Two authors (ASB and NV) independently performed the risk of bias assessment using the Cochrane risk-of-bias tool for randomized trials Version 2 (RoB 2)^18^. Disagreements were resolved by a third party (BF). Five key domains were assessed: the randomisation process, deviation from the intended intervention, missing out-come data, outcome measurement, and selection of the reported results. Evaluations of these domains were categorized into: "low risk," "some concerns," or "high risk” of bias, as the guideline suggests.

## Results

### Selection process

After duplicate removal and exclusion based on titles and abstracts, 11,374 articles were screened in more detail for eligibility. Subsequently, another 312 were excluded because of missing outcome parameters or ineligible study settings (e.g., wrong outcome measure, inappropriate control group, pre-existing psychological illnesses, etc.). This resulted in 129 articles analyzed in this study. In the updated search, 1431 articles were screened after duplication removal. Twenty-eight articles were searched in full length, which resulted in 14 articles analyzed in this study. The complete search and selection process is shown in the PRISMA flowchart Fig. S1. The baseline characteristics of the enrolled studies are detailed in Table S2.

### Systematic review

Thirteen studies reported on global QoL, and nine studies^19–27^ reported improved QoL in the intervention group compared to the control group. Five out of seven studies^19–21,28,29^ reported improvement in the intervention group for emotional QoL. For physical QoL, two out of three studies^26,27^ reported that scores in physical domains improved in the intervention group. For social QoL, three out of five studies^19,21,28^ reported better QoL scores in the intervention group than in the control group. These studies were not included in the meta-analysis because of missing data.

### Meta-analysis

#### Psychological interventions do not affect survival

**Figure 2:**
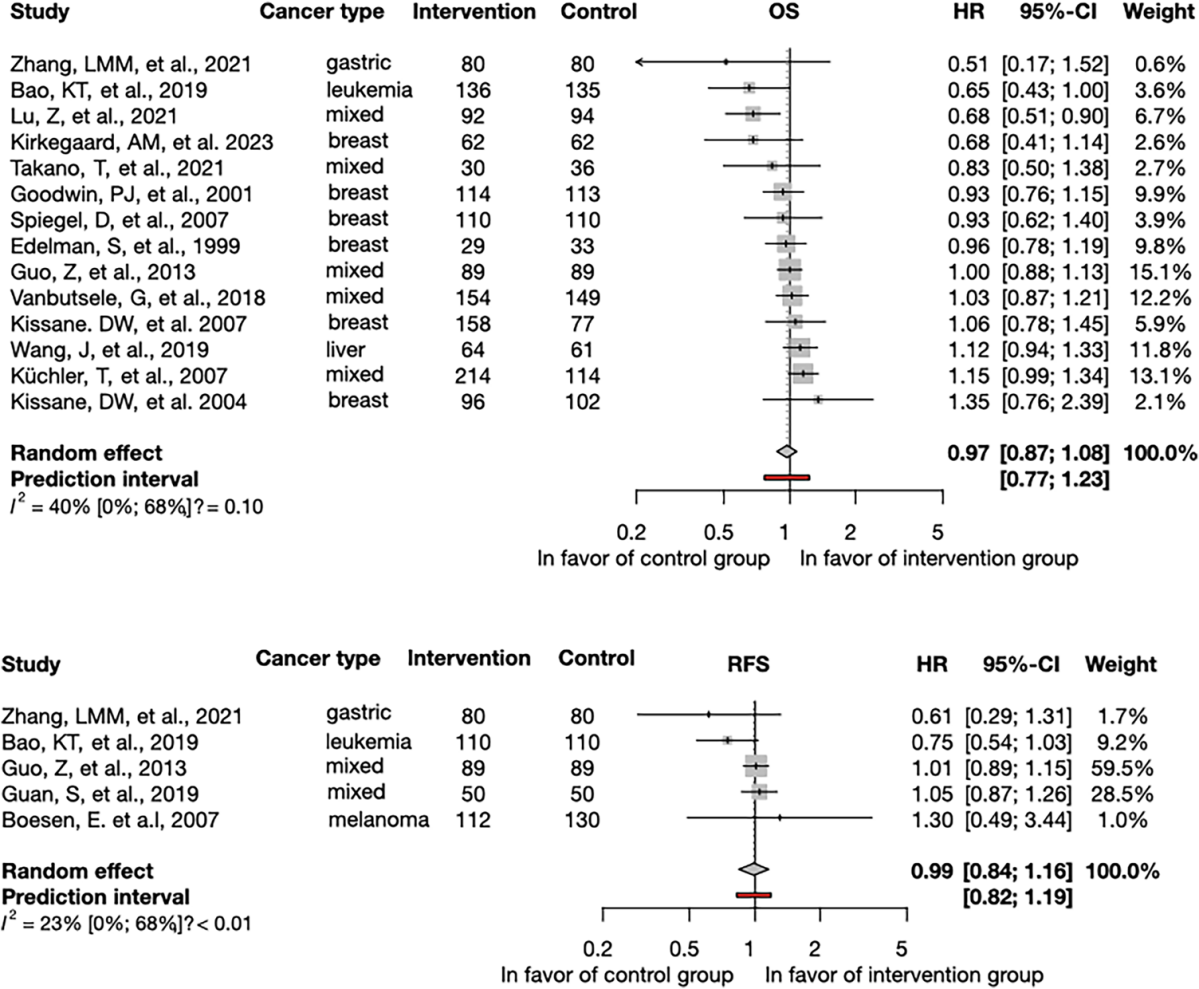
The effect of psychological interventions on overall survival and recurrence-free survival. Psychological interventions did not result in significant differences in the improvement of overall survival (OS) (**a**) and recurrence-free survival (RFS) (**b**) between the intervention and control groups. Diamonds and horizontal lines represent the hazard ratio (HR) and confidence intervals (CI) for each comparison (OS: overall survival; RFS: recurrence free survival), cancer—various cancer subtypes**.**

Fourteen studies reported on the OS, involving 2683 patients, and five reported on RFS, involving 900 patients. The OS of patients did not differ significantly between the intervention and the control groups (HR = 1.01; 95% CI = 0.95–1.07). Similarly, the two groups had no significant difference in RFS (HR = 0.99; 95% CI = 0.84–1.16) (Fig. 2).

#### Longitudinal meta-analysis: post-intervention (T0) results

##### Psychological interventions significantly improve quality of life in all measured domains

**Figure 3:**
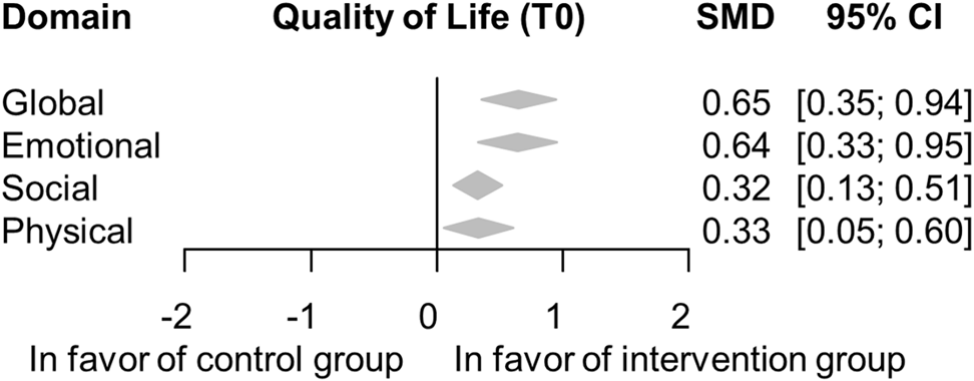
Forest plot of the pooled results in the different domains of Quality of Life. Psychological interventions significantly improve global, emotional, social and physical quality of life compared to the control group. Diamonds and horizontal lines represent standardized mean differences (SMS) and confidence intervals (CI) for each comparison.

Here, we present the results of model-based prediction values for the overall post-intervention effect sizes. In the intervention group, we found significant improvements compared to the control group in all analyzed domains of QoL: global (SMD = 0.65; 95% CI = 0.35–0.94), emotional (SMD = 0.64; 95% CI = 0.33–0.95), social (SMD = 0.32; 95% CI = 0.13–0.51) and physical (SMD = 0.33; 95% CI = 0.05–0.6) domains (Fig. 3). A forest plot of the pooled results for each domain at different time points is presented in Fig. S2.

**Figure 4:**
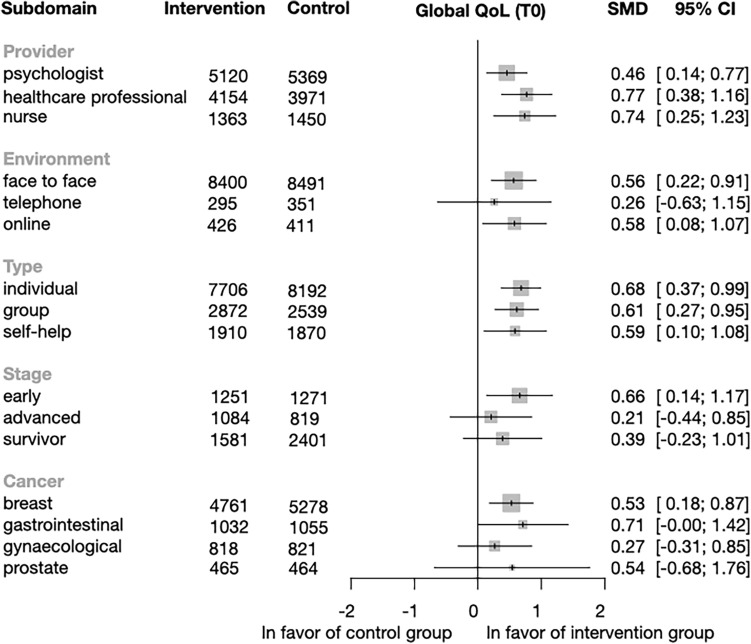
Forest plot of the subgroup analysis of Global Quality of Life (QoL). Summary plot showing results by provider, environment, type, and cancer stage for the global QoL. Diamonds and horizontal lines represent standardized mean differences (SMD) and confidence intervals (CI) for each comparison.

*Subgroup analysis of global QoL* Based on the subgroup analysis structure mentioned earlier, we found significant improvement (T0) in the intervention group in all categories of providers: psychologist (SMD = 0.46; CI = 0.14–0.77; p = 0.006), healthcare professional (SMD = 0.77; CI = 0.38–1.16; p = 0.0004), and nurse (SMD = 0.74; CI = 0.25–1.23; p = 0.005). In the environment category, significant improvements were observed for face-to-face (SMD = 0.56; CI = − 0.22 to 0.91; p = 0.003) and online (SMD = 0.58; CI = 0.08–1.07; p = 0.029) intervention, but no improvement was seen when the intervention was delivered via telephone (SMD = 0.26; CI = − 0.63 to 1.15; p = 0.418). In the category of intervention type, we found that there was a significant improvement in the experimental group for individual (SMD = 0.68; CI = 0.37–0.99; p < 0.001), group therapy (SMD = 0.61; CI = 0.27–0.95; p = 0.0009), and the guided self-help therapy (SMD = 0.59; CI = 0.1–1.08; p = 0.026). Significant improvements in the cancer stage were only seen when patients were in the early stages of cancer (SMD = 0.66; CI = 0.14–1.17; p = 0.017) (Fig. 4). Regarding cancer type, we only found a significant difference in the breast cancer group (SMD = 0.53; CI = 0.18–0.87; p = 0.004). Individual study effects and other measured time points (T12, T24, and T48) are shown in Figs. S3–S7.

**Figure 5:**
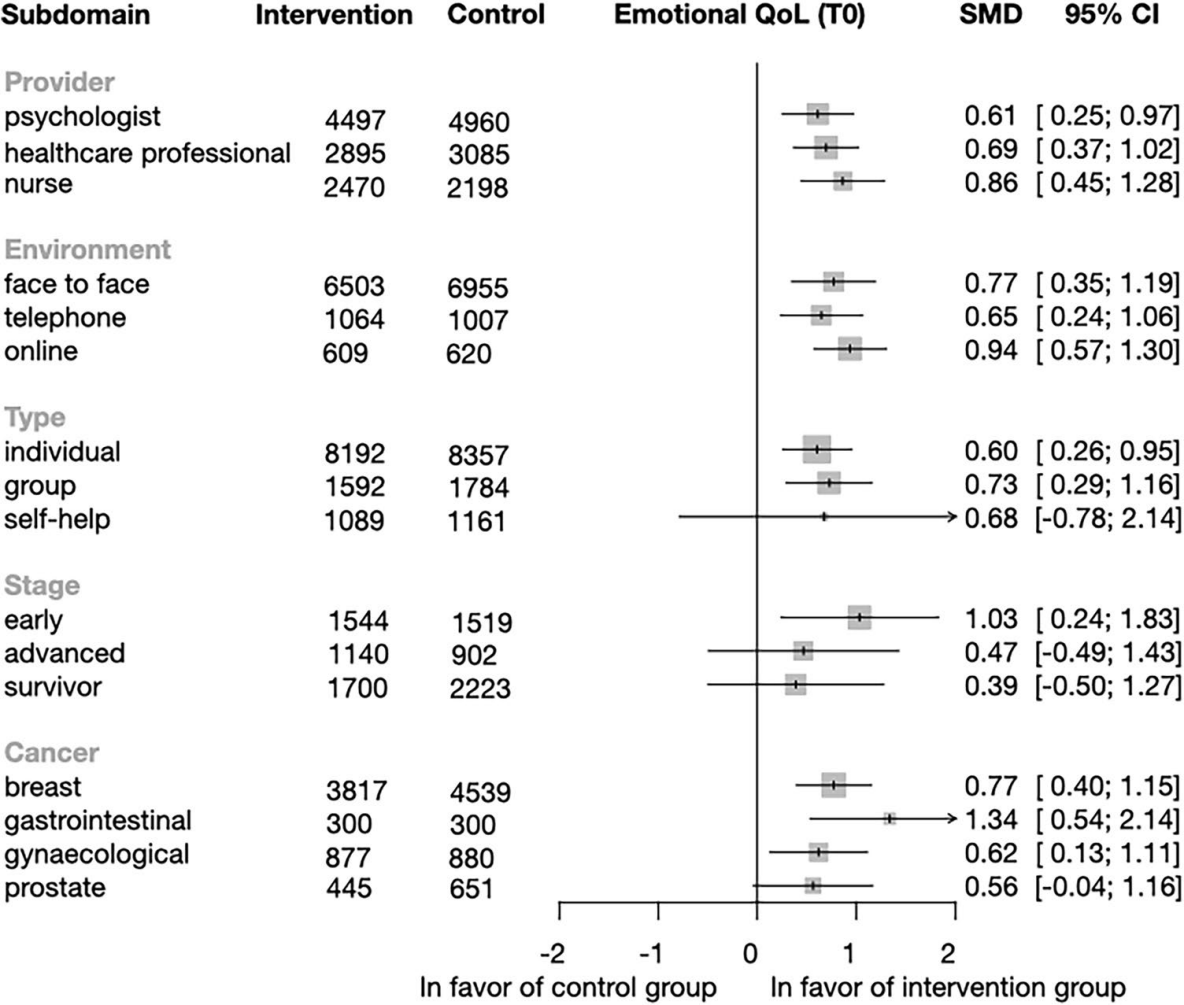
Forest plot of the subgroup analysis of Emotional Quality of Life (QoL). Forest plot showing the results of subgroup analysis of the emotional QoL domain by provider, environment, type and cancer stage. Diamonds and horizontal lines represent standardized mean differences (SMD) and confidence intervals (CI) for each comparison. mean difference, CI: confidence interval.

*Subgroup analysis of emotional QoL* Patients receiving the psychological intervention had significantly improved quality of life regardless of the provider: psychologist (SMD = 0.61; CI = 0.25–0.97; p = 0.0018), healthcare professional (SMD = 0.69; CI = 0.37–1.02, p = 0.0002), and nurse (SMD = 0.86; CI = 0.45–1.28, p = 0,0002). The results showed significant improvement in the environment subgroup in the experimental group when the intervention was delivered face-to-face (SMD = 0.77; CI = 0.35–1.19; p = 0.001), online (SMD = 0.94; CI = 0.57–1.3; p = 0.0004) or via telephone (SMD = 0.65; CI = 0.24–1.06; p = 0.005). There was a significant improvement in the type of intervention for the individual (SMD = 0.60; CI = 0.26–0.95; p = 0.001) and group-based interventions (SMD = 0.73; CI = 0.29–1.16; p = 0.0025). There was no difference when the type of intervention was guided-self-help (SMD = 0.68; CI = − 0.78 to 2.14; p = 0.212). Significant emotional QoL changes were only observed in the early-stage cancer group (SMD = 1.03; CI = 0.24–1.83; p = 0.016) (Fig. 5). Regarding cancer type, we found a significant difference in breast cancer (SMD = 0.77; CI = 0.4–1.15; p = 0.0006), gastrointestinal (SMD = 1.34; CI = 0.54–2.14; p = 0.014) and gynecological group (SMD = 0.62; CI = 0.13–1.11; p = 0.02) but no difference was seen in the prostate cancer group (SMD = 0.56; CI = − 0.04 to 1.16; p = 0.062). Individual study effects and the other time points are shown in Figs. S7–S12.

**Figure 6:**
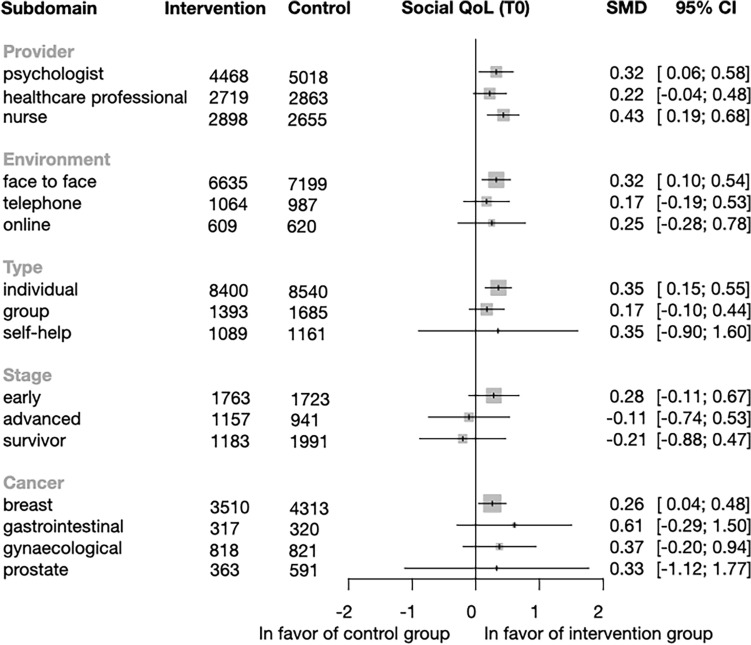
Forest plot of the subgroup analysis of Social Quality of Life (QoL). Forest plot showing the results of subgroup analysis of the social QoL domain by provider, environment, type and cancer stage. Diamonds and horizontal lines represent standardized mean differences (SMD) and confidence intervals (CI) for each comparison.

*Subgroup analysis of social QoL* Our analysis showed a significant improvement in social QoL in the subcategory of a psychologist (SMD = 0.32; CI = 0.06–0.58; p = 0.019) and nurse-delivered intervention (SMD = 0.43; CI = 0.19–0.68; p = 0.09) for the provider subgroup. The only significant environment was face-to-face intervention (SMD = 0.32; CI = 0.1–0.54; p = 0.006), and there was improvement only when the intervention was individual (SMD = 0.35; CI = 0.15–0.55; p = 0.001). Finally, there was no improvement in social QoL by the cancer stage (Fig. 6). Regarding cancer type, we only found a significant difference in the breast cancer group (SMD = 0.26; CI = 0.04–0.48; p = 0.021). Individual study effects and the other time points are shown in Figs. S13–S17.

**Figure 7:**
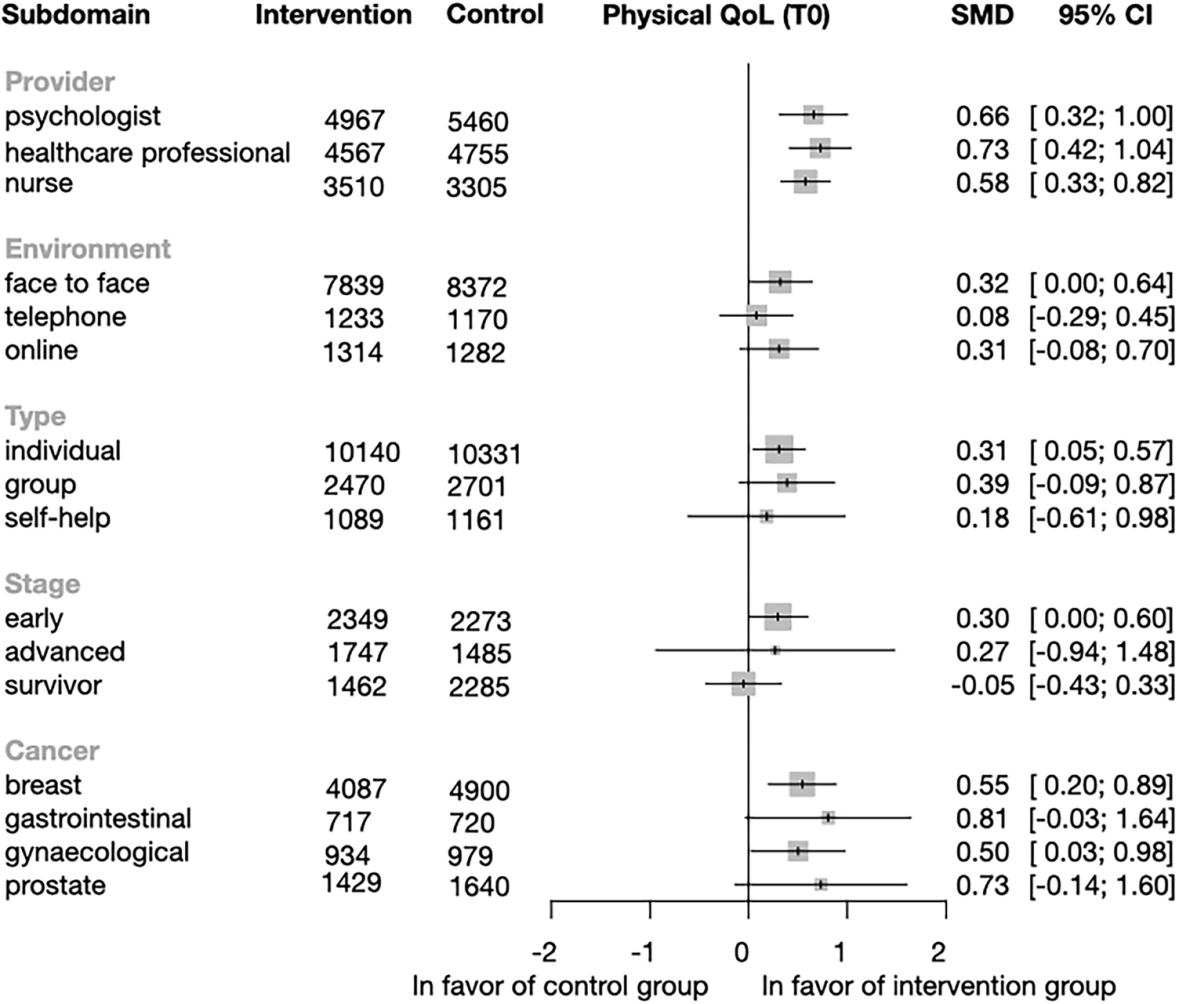
Forest plot of the subgroup analysis of Physical Quality of Life (QoL). Forest plot showing the results of subgroup analysis of the physical QoL by provider, environment, type, and cancer stage. SMD: standardized mean difference; CI: confidence interval. Diamonds and horizontal lines represent standardized mean differences (SMD) and confidence intervals (CI) for each comparison.

*Subgroup analysis of physical QoL* Physical QoL improved significantly in the intervention group compared to the control group in all categories of providers: psychologist (SMD = 0.66; CI = 0.32–0.99; p = p < 0.001), healthcare professionals (SMD = 0.73; CI = 0.42–1.04; p < 0,001), and nurse (SMD = 0.58; CI = 0.33–0.82; p < 0.001). Significant differences were only found in the environment category when the intervention was delivered face-to-face (SMD = 0.32; CI = 0.0019–0.64; p = 0.049). In the type category, only the individual (SMD = 0.31; CI = 0.05–0.57; p = 0.022) intervention had a significantly better effect. There was no notable improvements in cancer stage (Fig. 7). Regarding cancer, we can observe significant improvement in two cancer groups: breast cancer (SMD = 0.55; CI = 0.2–0.89, p = 0.003) and gynecological cancer (SMD = 0.50; CI = 0.03–0.98; p = 0.042) The effects of individual study and data for the other time points are shown in Figs. S18–S22.

##### Impact of the interventions over time in the four domains of quality of life

**Figure 8:**
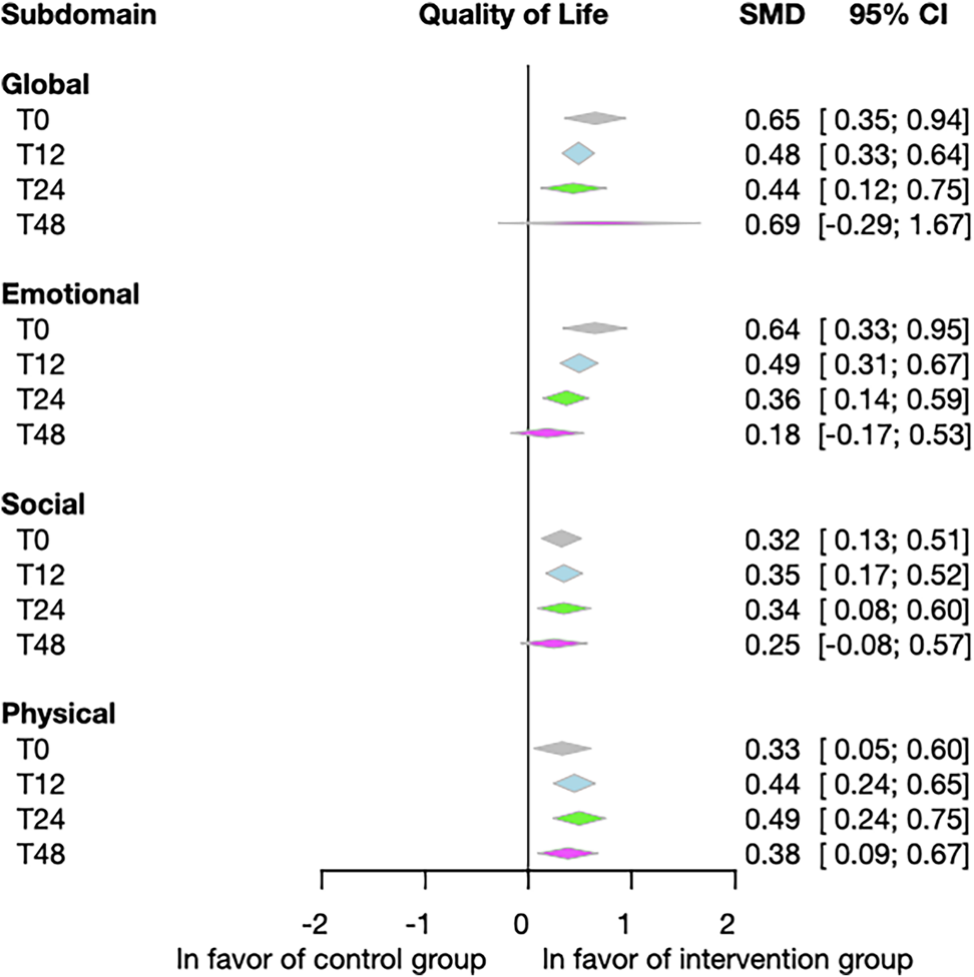
Summary forest plot showing the effect of intervention at different follow-up times. We found that the effect of follow-up time was not significant (T0—immediately after the intervention; T12—at week 12; T24—at week 24; T48—at week 48) in the four measured QoL domains. SMD: standardized mean difference; CI: confidence interval.

The post-intervention intervention had a significant effect, but the follow-up time effect was not significant. However, the point estimate decreased slightly during the follow-up time, and the 95% CI for the 48-week effect was the 0 effect (Fig. 8). The significant baseline improvement measured at the estimated time 0 t did not decrease before week 48. By week 48, the improvement had disappeared in almost all domains except physical QoL.

##### Duration of intervention

**Figure 9:**
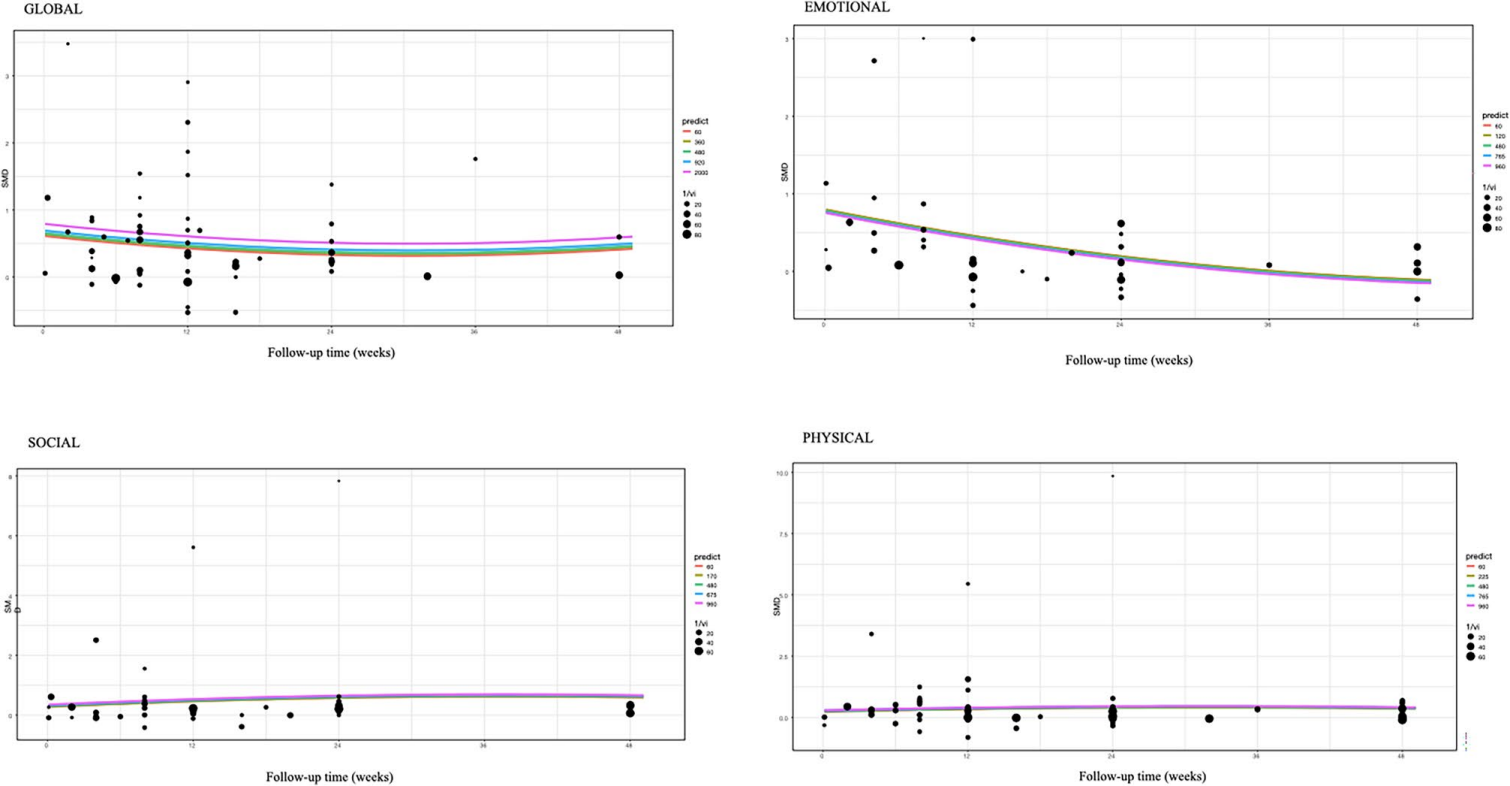
Regression plot of the duration of the intervention in the four domains of QoL. Regression plot showing the estimated SMDs of the four QoL domains by follow-up times based on the duration of the intervention with no significant difference. The size of the point represents the precision (more accurately: the size is proportional to the inverse of the variance of the given study), predictor: net minutes of interventions.

Our analysis showed that, based on the duration of the intervention (net minutes/patient), QoL SMDs did not change significantly by each week in any domain (Fig. 9). (Global QoL: SMD = 0.06; CI = 0.01–1.2/min; p = 0.05; emotional QoL: SMD = 0.80; CI = 0.28–1.33/min; p = 0,006 social QoL: SMD = 0.29; CI = − 0.10 to 0.64 /min; p = 0.146; physical QoL: SMD = 0.23; CI = − 0.09 to 0.56/min; p = 0.159). Figure 9.

### Risk of bias assessment

The detailed results of the risk of bias assessment are presented in Table S23. For survival and QoL, most of the studies had some concerns about the risk of overall bias, mainly due to the randomization process.

### Publication bias and heterogeneity

For OS, we could observe a possible publication bias of small studies, but the effect of small studies does not change the conclusion to a relevant extent. In the case of RFS, the assessment of publication bias of small studies is limited as the number of studies is very low (S24.1.–S24.2.). For QoL, the funnel plots do not exclude the presence of potential publication bias, although the figure suggests a few outlier publications rather than systemic reporting bias (see S24.3..–S24.2.6.).

Statistical heterogeneity of survival data was considered moderate: OS: I^2^ = 40% [0%, 68%]; and low: RFS: I^2^ = 23% [0%, 68%].

## Discussion

The psychosocial difficulties experienced by cancer patients in the long term are broad and include a wide range of symptoms such as anxiety, uneasiness, mourning, helplessness, fatigue, concentration difficulties, sleep problems, mental and cognitive impairments, sexual dysfunction, psychological distress, and psychiatric illnesses^30^. These symptoms are even more common in patients with poor prognosis and advanced-stage cancer^31^. Therefore, the above-mentioned psychosocial symptom-free period and QoL have become the primary endpoints^32^. Firkinns et al.^33^ found that QoL was significantly affected 2 to 26 years after cancer diagnosis. All these means that providing psychological support to cancer survivors in the long term is crucial.

Although our analysis concluded that psychological interventions do not prolong survival time, they can improve the quality of life of patients and the time that these patients and their families have left. Our analysis revealed significant improvements in all four measured QoL domains (global, emotional, social, and physical) in the intervention group compared to the control group, with the highest clinical effect in the emotional domains.

Moreover, our subgroup analysis showed significant improvements in QoL in the experimental group regardless of the intervention provider in most cases. This suggests that the rigorous research intervention and training have a strong influence on provider self-efficacy leaving less emphasis on the provider’s profession or personality itself^34^. The interventions used in the studies were mostly non-psychotherapies, where the role of a licensed psychologist would be essential.

The environment in which the interventions take place also influences the beneficial effect. Our analysis showed that face-to-face interventions were the most effective. This implies that personal interactions are important factors in delivering psychological interventions. The online form was only significant in global and emotional domains. This suggests that an online form can also be effective if a patient has difficulty going to hospital. A review article conducted on this topic found that even though online interventions may supplement traditional treatment setups for mental disorders, they could not provide consistent quality or replace face-to-face therapy^35^. Further research should evaluate how online therapies could be improved to be more effective in providing quality treatment for less mobile patients.

Regarding the type of interventions, there were significant improvements for individual therapies in all measured domains. Group-based therapies were significantly effective in the global and emotional domains. Cancer patients often express a preference for individual over group therapy for various fears despite their effectiveness^36^. Participating in group therapy where fellow patients are suffering from the same condition in a worse condition might be frightening to see. It is also possible that heterogeneous groups make it difficult to tailor the best possible treatment for each patient group. For this reason, individual therapies could be a better choice. Guided self-help was only statistically significant in the global domain, but we cannot draw conclusions due to the limited data.

It has been proposed that psychological interventions only affect the prognosis of patients with early-stage cancer, as the natural course of more advanced stages might obviate the possible effect of psychosocial factors^8^. Our results may support these findings as the point estimates were higher for early-stage patients but did not prove significant. The results for the cancer stage suggest that psychological interventions are most effective when provided in the early stages rather than in the advanced or survival phase. An interesting finding is that these interventions did not affect the survival category. These patients may have gone through post-traumatic growth, and these interventions are not strong enough for them to make a difference. A study conducted on post-traumatic self-growth among cancer survivors found that the positive effects of surviving cancer can last up to 4 years; however, after that, patients started to have lower scores^37^. We must highlight that we had limited data to analyze the effect of the cancer stage, but monitoring patients' needs, even for survival patients, should be a standard.

Regarding cancer type, we found that breast cancer patients benefited most from the psychological interventions. No improvement was seen in the prostate cancer group in any domains. This raises the question of whether gender plays a role in seeking and accepting psychological help. There is evidence in the literature to support the idea that gender is a predictor of attitudes toward seeking professional psychological help; however, other factors like cultural background and educational level are important factors, too^38,39^.

**Figure 10:**
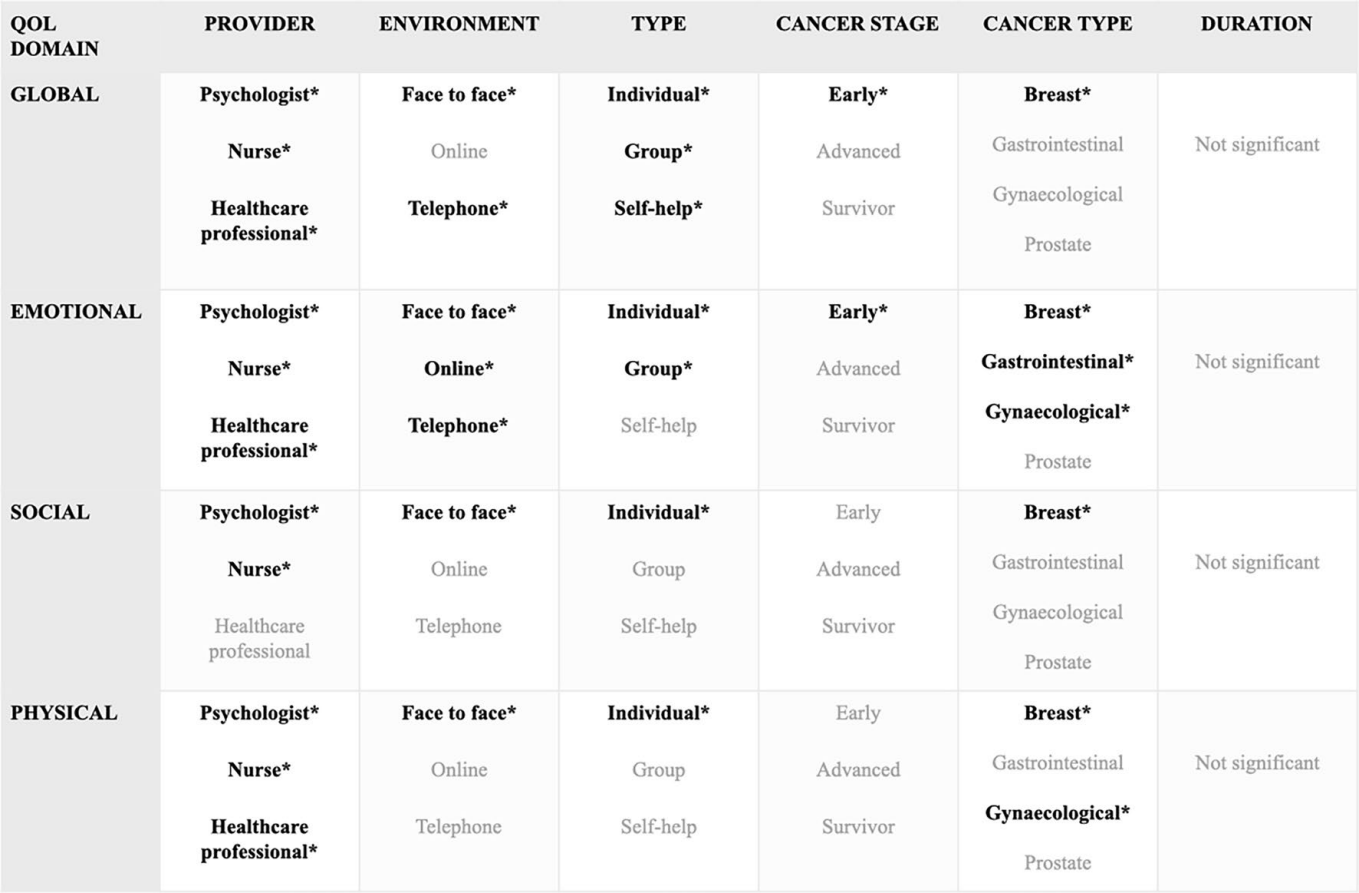
Sum of the subgroup analysis of QoL domains. The figure shows the cumulative findings of subgroup analysis of all measured QoL domains. We indicated the significant differences between the intervention and the control groups with the bold *.

Interestingly, our results showed that the duration of the intervention is not an important factor for psychological interventions in improving the four analyzed QoL domains. This aligns with the results of a study where researchers found that the number of sessions, length, and treatment intensity were unrelated to therapeutic gains^40^. Due to the heterogeneity of interventions, we were not able to analyze data by duration, frequency, and occasion; therefore, further research is needed in this subgroup. This is, however, an important finding for future recommendations and funding, as we could standardize short but intensive interventions at least three or four times a year to be cost- and time-effective when treating these patients for their QoL. Figure 10. shows the sum of the subgroup analysis of QoL domains.

Our results suggest that psychological interventions are effective and should be introduced into the routine care of oncological patients. We have gained important information based on provider, type, environment, and duration of intervention efficacy, as well as on cancer stage and type, that can be used to improve the effectiveness of psychological interventions. At the same time, the significance varying across subgroups indicates that patients have different needs; therefore, we should strive to provide personalized patient care.

### Strengths and limitation

One of the strengths of this work is its absolute objectivity, which was performed using meta-analyses and rigorous methodology. We were able to provide the highest level of evidence available by including only RCTs with a large number of enrolled patients. To our knowledge, this is the most comprehensive meta-analysis to report on the effectiveness of psychological intervention by provider, environment, type, and cancer stage subgroup. Although the results of this meta-analysis seem promising, the conclusions should be interpreted with caution. In terms of the limitations of this work, the first and most important thing to note is the heterogeneous study/clinical settings between the included studies, in particular, the different types of interventions, cancer types, and measurement tools. These differences made it necessary to use less sensitive statistical analysis. Criteria were developed to define psychological interventions; however, these terms and methods can often be used interchangeably, and the distinction may be subjective. This is further aggravated by insufficient details on interventions; therefore, decisions on inclusion or exclusion may also be superficial^7^. We could only rely on a small amount of data for the survival analysis, so conclusions should be drawn carefully. Lastly, a further limitation is the presence of moderate to high risk of bias in some areas.

### Implications for practice and research

Implementing scientific results in everyday clinical practice is crucial and can improve disease management, diagnosis, and therapy^41,42^. Our results suggest that psychotherapy should be introduced as standard care for patients with cancer. Psychologists are not part of the patient care team in many countries, and psychotherapy is unavailable for oncological patients. However, psychological interventions should be provided, especially in the early stages of cancer, and should be repeated at least three or four times to maintain the beneficial effects. Further trials could make more personalized recommendations based on cancer types, stages, and psychological methods.

Another important aspect of this review is that our results highlight the need for randomized-controlled clinical trials with standardized methods and reporting on results to accurately assess the effect of psychological interventions. Psychological research is always challenging; however, more objective analyses could be obtained by standardizing intervention methods, questionnaires, intervention duration, frequency, and how data and results are reported.

## Conclusion

Even though the survival analysis did not show significant differences between the two groups based on the limited amount of data, our results provide evidence for the beneficial effect of psychological interventions on several aspects of QoL in patients with cancer. While medical research emphasizes survival as the hardest outcome, QoL plays an important role in individuals' lives and should not be underestimated. A longer life in suffering is not necessarily better than a shorter one spent in a better well-being. For that reason, in clinical practice, the assessment of QoL should be an essential part of routine care to provide personalized psychological treatments.

### Supplementary Information

Supplementary Information.

## Data Availability

All data is shared as an additional file.
