# Supplementary material for: Psychological intervention improves quality of life in patients with early-stage cancer: a systematic review and meta-analysis of randomized clinical trials
Source: Sci Rep. 2024 Jun 9;14:13233. doi: 10.1038/s41598-024-63431-y (PMC11162999; doi:10.1038/s41598-024-63431-y)
Supplement: Supplementary file 1 — Supplementary Information. [file 41598_2024_63431_MOESM1_ESM.pdf]

Supplementary Material

Psychological Intervention Improves Quality of Life in Patients with Early-Stage Cancer: a Systematic Review and Meta-analysis of randomized clinical trials

TABLE S1. SEARCH KEY

SECTION S1. STRUCTURE OF ANALYSIS

FIGURE S1. PRISMA FLOW CHART

TABLE S2. BASELINE CHARACTERISTICS OF THE INCLUDED STUDIES

SECTION S2. STATISTICAL ANALYSIS

S2.OVERALL PLOTS

Figure.S2.1.T12

Figure S2.2.T24

Figure S2.3.T48

S3. SUBGROUP ANALYSIS OF GLOBAL QOL: PROVIDER

Figure S3.1.T0

Figure S3.2.T12

Figure S3.3.T24

Figure S3.4.T48

S4.SUBGROUP ANALYSIS OF GLOBAL QOL: ENVIRONMENT

Figure S4.1.T0

Figure S4.2.T12

Figure S4.3.T24

Figure S4.4.T48

S5.SUBGROUP ANALYSIS OF GLOBAL QOL: TYPE

Figure S5.1.T0

Figure S5.2.T12

Figure S5.3.T24

Figure S5.4.T48

S6. SUBGROUP ANALYSIS OF GLOBAL QOL: CANCER STAGE

Figure S6.1.T0

Figure S6.2.T12

Figure S6.3.T24

Figure S6.4.T48

S7. SUBGROUP ANALYSIS OF GLOBAL QOL: CANCER TYPE

Figure S7.1.T0

Figure S7.2.T12

Figure S7.3.T24

Figure S7.4.T48

S8.SUBGROUP ANALYSIS OF EMOTIONAL QOL: PROVIDER

Figure S8.1.T0

Figure S8.2.T12

Figure S8.3.T24

Figure S8.4.T48

S9.SUBGROUP ANALYSIS OF EMOTIONAL QOL: ENVIRONMENT

Figure S9.1.T0

Figure S9.2.T12

Figure S9.3.T24

Figure S9.4.T48

S10.SUBGROUP ANALYSIS OF EMOTIONAL QOL: TYPE

Figure S10.1.T0

Figure S10.2.T12

Figure S10.3.T24

Figure S10.4.T48

S11.SUBGROUP ANALYSIS OF EMOTIONAL QOL: CANCER STAGE

Figure S11.1.T0

Figure S11.2.T12

Figure S11.3.T24

Figure S11.4.T48

Figure S12.1.T0

Figure S12.2.T12

Figure S12.3.T24

Figure S12.4.T48

S13.SUBGROUP ANALYSIS OF SOCIAL QOL: PROVIDER

Figure S13.1.T0

Figure S13.2.T12

Figure S13.3.T24

Figure S13.4.T48

S14.SUBGROUP ANALYSIS OF SOCIAL QOL: ENVIRONMENT

Figure S14.1.T0

Figure S14.2.T12

Figure S14.3.T24

Figure S14.4.T48

S15.SUBGROUP ANALYSIS OF SOCIAL QOL: TYPE

Figure S15.1.T0

Figure S15.2.T12

Figure S15.3.T24

Figure S15.4.T48

S16.SUBGROUP ANALYSIS OF SOCIAL QOL: CANCER STAGE

Figure S16.1.T0

Figure S16.2.T12

Figure S16.3.T24

Figure S16.4.T48

S17.SUBGROUP ANALYSIS OF SOCIAL QOL: CANCER TYPE

Figure S17.1.T0

Figure S17.2.T12

Figure S17.3.T24

Figure S17.4.T48

S18. SUBGROUP ANALYSIS OF PHYSICAL QOL: PROVIDER

Figure S18.1.T0

Figure S18.2.T12

Figure S18.3.T24

Figure S18.4.T48

S19. SUBGROUP ANALYSIS OF PHYSICAL QOL: ENVIRONMENT

Figure S19.1.T0

Figure S19.2.T12

Figure S19.3.T24

Figure S19.4.T48

S20. SUBGROUP ANALYSIS OF PHYSICAL QOL: TYPE

Figure S20.1.T0

Figure S20.2.T12

Figure S20.3.T24

Figure S20.4.T48

S21. SUBGROUP ANALYSIS OF PHYSICAL QOL: CANCER STAGE

Figure S21.1.T0

Figure S21.2.T12

Figure S21.3.T24

Figure S21.4.T48

S22.SUBGROUP ANALYSIS OF PHYSICAL QOL: CANCER TYPE

Figure S22.1.T0

Figure S22.2.T12

Figure S22.3.T24

Figure S22.4.T48

S23. TABLE S.3. RISK OF BIAS ASSESSMENT

S24. PUBLICATION BIAS

Figure S24.1.Overall Survival

Figure S4.2. Recurrence-free Survival

S24.3.Global Qol

Figure S24.3.1.Provider
Figure S24.3.2.Environment
Figure S24.3.3.Type
Figure S24.3.4.Duration of Intervention
Figure S24.3.5.Cancer Stage
Figure S24.3.6.Cancer Type

S24.4.Emotional Qol

Figure S4.4.1.Provider
Figure S24.4.2.Environment
Figure S24.4.3.Type
Figure S24.4.4.Duration of Intervention
Figure S24.4.5.Cancer Stage
Figure S24.4.6.Cancer Type

S24.5.Social Qol

Figure S24.5.1.Provider
Figure S24.5.2.Environment
Figure S24.5.3.Type
Figure S24.5.4. Duration of Intervention
Figure S24.5.5.Cancer Stage
Figure S24.5.6.Cancer Type

S24.6. Physical Qol
Figure S24.6.1. Provider
Figure S24.6.2. Environment
Figure S24.6.3.Type
Figure S24.6.4. Duration of Intervention
Figure S24.6.5. Cancer Stage
Figure S24.6.6. Cancer Type

REFERENCES

Table S1. Search key

Search Key	Database
(psychotherapy OR psychotherap* OR "mindfulness" OR "cognitive therapy" OR "cognitive treatment" OR "cognitive intervention" OR CBT OR "behavior therapy" OR "behavioral therapy" OR "behaviour therapy" OR "behavioural therapy" OR "behavior treatment" OR "behavioral treatment" OR "behaviour treatment" OR "behavioural treatment" OR "behavior intervention" OR "behavioral intervention" OR "behaviour intervention" OR "behavioural intervention" OR "psychological intervention" OR "psychosocial intervention" OR "psychological support" or "psychosocial support" OR counselling OR counseling) AND (neoplasm OR cancer) AND (random OR randomised OR randomization OR randomly OR randomness)	Medline
	Embase
	Central

Table S.1. The Search Key. The search key used to identify all eligible studies

Section S1. Structure of analysis

At first we were interested in whether psychological interventions have an effect on overall and recurrence free survival. Next we continued our analysis to see if cancer patients' QoL can be improved by psychological interventions. We chose to analyze four quality of life domains that we considered the most relevant for psychological interventions: global, physical, emotional, and social.

Subgroup analysis with subcategories.

A. Firstly, we were interested to see if the provider of the interventions made a difference, so we made a provider subgroup and categorized the data from the articles into three subcategories: 1. psychologist, 2. healthcare professional and 3. nurse.. (The healthcare professional could be any personnel who did not belong to the earlier two subcategories. e.g. research assistant, medical doctor, doctoral student, therapists, or if they were mentioned as a team so we could not be specific.)
B. We were also interested to see if the channel or the environment of the interventions are important factors. So, we made three subcategories for that: 1. face-to-face (taking place in a clinical environment), 2. Telephone (taking place in patients' home), or via 3. Online (taking place in patients' home) as described in the included articles.
C. As next step we assessed whether the type of the intervention had any effect on the quality of life so we made again three subcategories: 1. individual, 2. group or 3. guided self-help. (With the guided self help we are referring to the fact that these materials were specifically made in the studies for these patients to improve their quality of life.)
D. Cancer stage: 1. early (I, II), 2. advanced (III, IV), 3. survivors as they were categorized in the original studies, regardless of cancer type.
E. Cancer type: four subcategories were made for this subgroup: 1. breasts, 2. gynecological, 3. gastrointestinal and 4. prostate. For further categories we did not have enough data.

Additionally we were interested to know whether the duration of the intervention was an important factor to improve QoL. Due to the heterogeneity of the durations, frequencies and occasions we could only make subgroups based on how many minutes per patient were used in the articles.

Lastly we wanted to see the time effect of these interventions based on the predicted follow–up times.

Figure S1. PRISMA flow chart

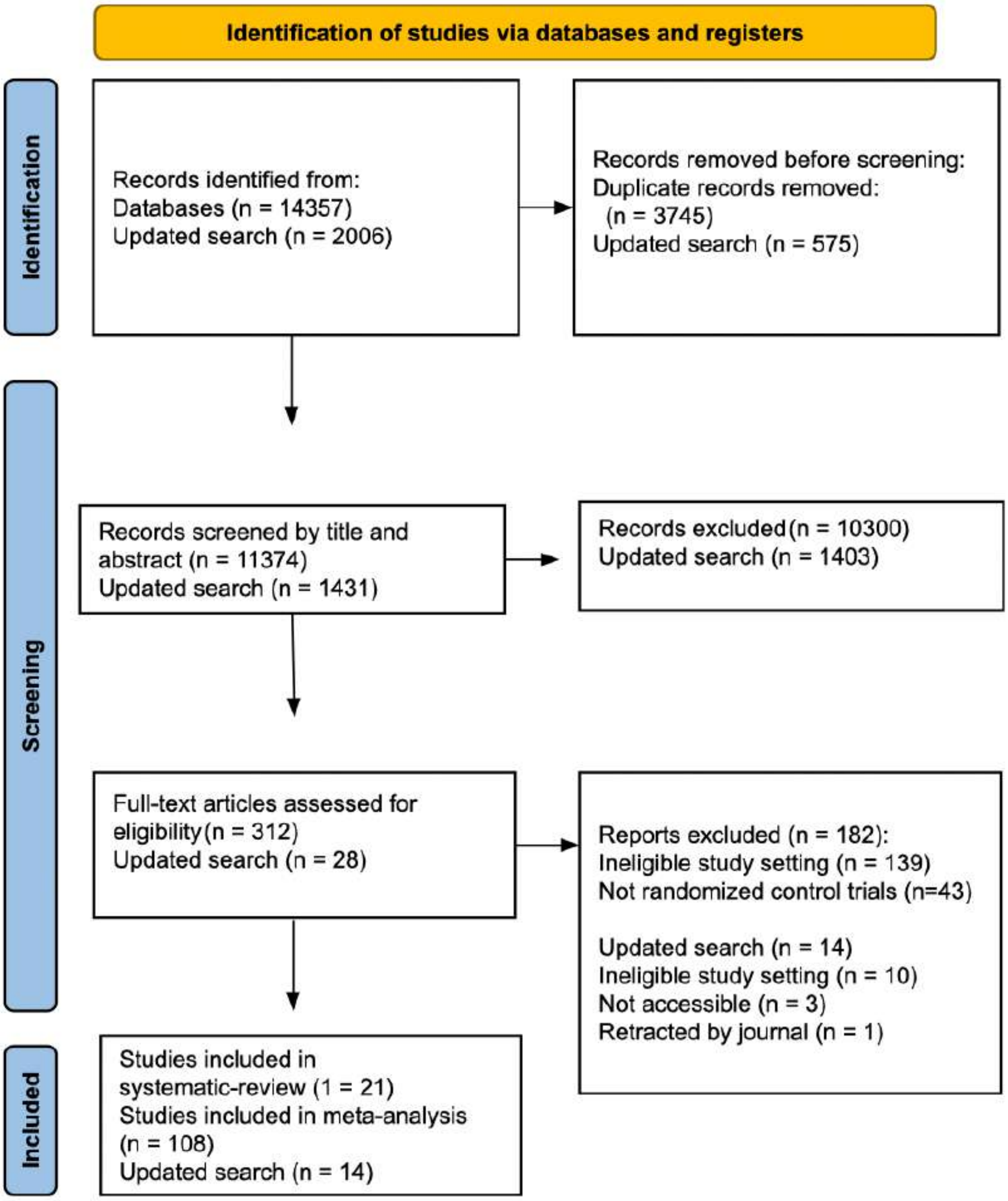

Figure 1. PRISMA flow diagram of the screening and selection process. The search was updated during the revision process.

Table S2. Baseline characteristics of the included studies

Author	Country	Cancer type	Sample size (female% of total)	Sample size (female% of intervention)	Sample size (female% of control)	Intervention name	Intervention's duration	Intervention's provider	Intervention's environment	Intervention type	Outcome	Questionnaire	Time of the measurement in weeks
**Articles included in the meta-analysis**													
**Huri M, et al., 2015[1]**	Turkey	prostate	34 (0)	19 (0)	15 (0)	cognitive behavior therapy based occupational therapy	twice a week, 12 weeks ,60 session	Psy	FF	G	QoL	EORTC-QLQ-C30 and PR25	12
**Giesler, BR, et al., 2005[2]**	USA	prostate	99 (0)	48 (0)	51 (0)	cancer intervention identified and tracked QoL, tailored support and education based on problems	once a month for 24 weeks	N	FF	I	QoL	PCQoL, SF-36	16,28,48

Author	Country	Cancer type	Sample size (female% of total)	Sample size (female% of intervention)	Sample size (female% of control)	Intervention name	Intervention's duration	Intervention's provider	Intervention's environment	Intervention type	Outcome	Questionnaire	Time of the measurement in weeks
**Wang, J, et al., 2019[3]**	China	liver	136 (22)	68 (16)	68 (n/a)	comprehensive education and care program	12 month, weekly different types of intervention	N	M (FF, T)	M (P, G)	QoL	EORTC-QLQ-C30	48
**Trask, PC, et al. 2003[4]**	USA	melanoma	48 (71)	25 (68)	23 (74)	cognitive-behavioral intervention for distress	3 subsequent 50-minute weekly sessions, over a period of 4 weeks.	HcP	FF	I	QoL	SF-36	8, 24
**Takano, T, et al., 2021[5]**	Japan	mixed	69 (n/a)	31(83)	38 (78)	patients received self-help workbooks. investigators including medical oncologists, psychiatrists, and clinical psychologists made the workbook originally for this study	24 weeks	SH	SH	SH	QoL	GQOL	12
												EORTC QLQ-C30	12
												GQOL	24
												EORTC QLQ-C30	24

Author	Country	Cancer type	Sample size (female% of total)	Sample size (female% of intervention)	Sample size (female% of control)	Intervention name	Intervention's duration	Intervention's provider	Intervention's environment	Intervention type	Outcome	Questionnaire	Time of the measurement in weeks
**McCaughan, E, et al., 2018[6]**	UK	prostate	17 (0)	13 (0)	4 (0)	program focused on symptom management, sexual dysfunction, uncertainty management, positive thinking and couple communication	5 session 2 hours group and 2 session for phone	HcP	M (FF, T)	M (G, P)	QoL	FACT-G	n/a
													4
**Walker, J, et al. 2014[7]**	UK	lung	142 (65)	68 (65)	74 (65)	depression care	every four weeks for 32 weeks	N	FF	I	QoL	EORTC-QLQ-C30	15
**Zhang, LMM, et al., 2021[8]**	China	gastric	160 (54)	80 (53)	80 (55)	reminiscence therapy	60 minutes, twice per month, for 12 consecutive months, patients were given a total of 24 reminiscence therapy sessions	N	FF	G	QoL	EORTC-QLQ-C30	12
													24
													36
													48
**Walker, LG, et al.,1998[9]**	UK	breast	88 (100)	44 (100)	44 (100)	relaxation combined with guided imagery	daily practice based on audio cassette and 40 women received 5 live training	SH	SH	SH	QoL	GQOL	15
**Arving, C, et al., 2007[10]**	Sweden	breast	85 (100)	47 (100)	38 (100)	individual psychosocial support	four 3 hours session	Psy	FF	I	QoL	EORTC QLQ-C30	4
													12

Author	Country	Cancer type	Sample size (female% of total)	Sample size (female% of intervention)	Sample size (female% of control)	Intervention name	Intervention's duration	Intervention's provider	Intervention's environment	Intervention type	Outcome	Questionnaire	Time of the measurement in weeks
													24
													4
													12
													24
**Liu, T, et al., 2019[11]**	China	thyroid	102 (n/a)	49 (34)	53 (38)	mindfulness-based stress reduction	8 session, weekly	Psy	FF	G	QoL	EORTC-QLQ-C30	9
													12
**Lutgendorf, SK, et al., 2010[12]**	USA	cervical	39 (100)	20 (100)	19 (100)	relaxation training	for 6 weeks, 4 times per week, 20-25 minutes each session	HcP	FF	G	QoL	FACT	4
													6
**Miaskowski, C, et al., 2007[13]**	USA	mixed	28 (71)	16 (69)	12 (74)	psychoeducational intervention	one academic session	N	FF	G	QoL	SF-36	6
**Lee, YH, et al. 2018[14]**	Taiwan	mixed	51(89)	25 (92)	26 (85)	stress management	one time occasion 110 minutes	HcP	FF	I	QoL	FACT-G	12
													24
**Hawkes, AL, et al. 2014[15]**	Australia	colorectal	410 (46)	205 (n/a)	205 (n/a)	health coaching	at least 6 of the 11 session, average length 31,5 min	HcP	T	I	QOL	FACT-C	24
													48
**Yoo, HJ, et al. 2004[16]**	Korea	breast	60 (100)	30 (100)	30 (100)	guided imagery and muscle relaxation	6 session	Psy	FF	G	QoL	FACT-B	12
													24

Author	Country	Cancer type	Sample size (female% of total)	Sample size (female% of intervention)	Sample size (female% of control)	Intervention name	Intervention's duration	Intervention's provider	Intervention's environment	Intervention type	Outcome	Questionnaire	Time of the measurement in weeks
**Klinkhammer-Schalke, M et al., 2012[17]**	Germany	breast	200 (100)	100 (100)	100 (100)	psychotherapy	n/a	Psy	FF	I	QoL	EORTC QLQ-C30	12<br>24<br>36<br>48
**Marchioro, G, et al. 1996[18]**	Italy	breast	36 (100)	18 (100)	18 (100)	cognitive behavioral psychotherapy and family counseling	during 9 month, weekly 50 minutes session for patients, bimonthly family counseling	Psy	FF	I	QoL	FLIC	4<br>12<br>24<br>36
**Walczak, A, et al.,2017[19]**	Australia	mixed	110 (33)	61 (34)	49 (31)	communication support program	one time 45 min session and one time 15 min phone call	N	M (FF, T)	I	QoL	FACT-G	4
**Petersen, RW, et al.,2002[20]**	n/a	mixed	50 (100)	25 (100)	25 (100)	progressive muscle relaxation, guided imagery and counseling sessions	one day intervention	HcP	FF	I	QoL	GHQ-28	6
**Vanbutsele, G, et al. 2018[21]**	n/a	mixed	186 (n/a)	92 (36)	94 (27)	early and systematic integration of palliative care	not specified, until death	HcP	FF	I	QoL	EORTC QLQ-C30, MQOL Single Item Scale, MQOL	12<br>18<br>24<br>n/a

Author	Country	Cancer type	Sample size (female% of total)	Sample size (female% of intervention)	Sample size (female% of control)	Intervention name	Intervention's duration	Intervention's provider	Intervention's environment	Intervention type	Outcome	Questionnaire	Time of the measurement in weeks
**Schofield, P, et al., 2013[22]**	Australia	lung	108 (na)	55 (44)	53 (36)	tailored intervention	two consultation session	HcP	FF	I	QoL	EORTC QLQ-C30	8<br>12
**Qin, X, et al., 2017[23]**	China	gastric	100 (na)	50 (n/a)	50 (n/a)	active psychological intervention based on routine nursing	during hospitalization	N	FF	I	QoL	EORTC QLQ-C30	n/a
**Zhou, L, et al., 2020[24]**	China	ovarian	73 (100)	37 (100)	36 (100)	at-home cognitive behavioral therapy	n/a	N	M (FF, SH)	I	QoL	EORTC QLQ-C30	12
**Northouse, LL, et al., 2007[25]**	USA	prostate	135 (0)	112 (0)	123 (0)	standard care plus a family-based intervention	4 months: consisted of 3 90-minute home visits and 2x30-minute T sessions spaced 2 weeks apart and delivered between baseline and 4 months.	N	M (FF, T)	I<br>I	QoL	FACT-G, SF-12	16<br>32<br>48
**Penedo, FJ, et al., 2020[26]**	USA	prostate	192 (0)	95 (0)	97 (0)	cognitive-behavioral stress management, cognitive-behavioral stress-and self management skills and relaxation	10 weeks 90 min/ session	Psy	O	G	QoL	FACT-G	24<br>48
**Penedo, FJ, et al., 2007[27]**	USA	prostate	71 (0)	41 (0)	30 (0)	cognitive-behavioral stress management - cognitive-behavioral stress-and self management skills and relaxation + half a day seminar	10x 2 hours/ sessions, once a week	Psy	FF	G	QoL	FACT-G	12

Author	Country	Cancer type	Sample size (female% of total)	Sample size (female% of intervention)	Sample size (female% of control)	Intervention name	Intervention's duration	Intervention's provider	Intervention's environment	Intervention type	Outcome	Questionnaire	Time of the measurement in weeks
**Rodríguez Vega, B, et al., 2010[28]**	Spain	mixed	72 (81)	39 (87)	33 (73)	native therapy	12x 45 min weekly sessions	Psy	FF	I	QoL	EORTC QLQ-C30	12<br>24
**Peoples, AR, et al., 2016[29]**	USA	mixed	48 (88)	24 (88)	24 (75)	cognitive behavior therapy	7 weeks, seven individual weekly CBT-I sessions, with sessions 1, 2, and 4 (30–60 min) conducted in person and sessions 3, 5, 6, and 7 (15–30 min) conducted over the phone	Psy	M (FF , T)	I	QoL	FACT-G	7<br>12
**Parker, PA, et al., 2009[30]**	USA	prostate	75 (0)	39 (0)	36 (0)	supportive attention	2 sessions + 2 booster sessions, 60-90 min each before surgery	Psy	FF	I	QoL	PCI, SF-36	6<br>24<br>48
**Parker, PA, et al., 2009[30]**	USA	prostate	75 (0)	39 (0)	36 (0)	cognitive behavioral stress management	2 sessions + 2 booster sessions, 60-90 min each before surgery	Psy	FF	I	QoL	PCI, SF-36	6<br>24<br>48
**van de Wal, M, et al., 2017[31]**	Australia	mixed	88 (53)	48 (53)	43 (53)	cognitive behavior therapy for high fear of recurrence	12 weeks, five individual 1-hour FF sessions (sessions 1-3, 5, and 8) combined with three 15-minute e-consultations (chat application, no video) with access to a Web site (sessions 4, 6, and 7)	Psy	M (O, FF)	I	QoL	EORTC QLQ-C30	12

Author	Country	Cancer type	Sample size (female% of total)	Sample size (female% of intervention)	Sample size (female% of control)	Intervention name	Intervention's duration	Intervention's provider	Intervention's environment	Intervention type	Outcome	Questionnaire	Time of the measurement in weeks
**Serfaty M, et al., 2018[32]**	US	mixed	42 (74)	20 (70)	22 (77)	acceptance and commitment therapy	8 sessions for 3 months, 1 hour/ session	Psy	FF	I	QoL	FACT-G, EQ-5D-VAS, 5Q-5D-5L	12<br>18<br>24
**Sandsund, C, et al., 2017[33]**	UK	gynaecological	142 (100)	72 (100)	70 (100)	consultation with professional familiar with behavioral change , collaborative care plan	3 months, number of sessions not specified	HcP	M (FF, T)	I	QoL	EORTC QLQ-C30, SF-36	12<br>24
**Wu, DY, et al., 2016[34]**	China	thyroid	60 (75)	30 (73)	30 (77)	psychological and behavioral intervention -	1 year, sessions not specified	N	M (P, O)	M (I, G)	Qol	EORTC QLQ-C30	48
**Rodin, G, et al., 2019[35]**	USA	leukemia	42 (38)	22 (36)	20 (40)	psychotherapeutic intervention	8–12 psychotherapeutic sessions, approx. 30–60 min each, delivered over 8 weeks, the first 8 sessions occur once or twice weekly during hospitalization (typically 1 month), adjusted in terms of frequency and duration depending on the patient's ability to participate. The remaining 4 sessions occur weekly or bi-weekly after discharge, in coordination with outpatient clinic visits.	HcP	FF	I	QoL	FACIT-Sp	4<br>8<br>12

Author	Country	Cancer type	Sample size (female% of total)	Sample size (female% of intervention)	Sample size (female% of control)	Intervention name	Intervention's duration	Intervention's provider	Intervention's environment	Intervention type	Outcome	Questionnaire	Time of the measurement in weeks
**Zhou, J, et al., 2020[36]**	China	leukemia	118 (58)	59 (59)	59 (56)	,disease-related health education, catheterization management, psychological counseling	not specified - during the hospitalization period	N	FF	I	QoL	SF-36	n/a
**Thomas, ML, et al., 2012[37]**	USA	mixed	152 (13)	64 (16)	88 (10)	coaching - education (video for managing cancer pain, overcoming attitudinal barriers pamphlet) + motivational interviewing - exploring beliefs about pain, use of analgesics, nonpharmacologic pain management strategies, communication about pain management	4 x 30 minutes telephone call	N	T	I	QoL	FACT-G, SF-36	12
**Rodríguez, C.F. et al., 2014[38]**	Spain	lung	90 (17)	40 (26)	50 (10)	behavior activation	4 x 60 min sessions	Psy	FF	I	QoL	EORTC QLQ-C30	n/a
													12
**Fann, JR, et al., 2009[39]**	USA	mixed	215 (60)	112 (63)	103 (58)	depression management	6 to 8 sessions for up to 12 months. the first treatment session lasts one hour, with subsequent sessions lasting 30 minutes	HcP	FF	I	QoL	1-10 scale	12
													24
													48
													72

Author	Country	Cancer type	Sample size (female% of total)	Sample size (female% of intervention)	Sample size (female% of control)	Intervention name	Intervention's duration	Intervention's provider	Intervention's environment	Intervention type	Outcome	Questionnaire	Time of the measurement in weeks
													96
Ohlson-Nevo, EO, et al, 2015[40]	Sweden	mixed	80 (37)	44(42)	36 (32)	education and psychological	once a week for seven weeks, each time 60 minutes of lecture followed by 60 minutes of discussion	N	FF	G	QoL	SF-36	4<br>24<br>48
Antoni, MH, et al., 2006[41]	USA	breast	199 (100)	92 (100)	107 (100)	cognitive behavior therapy	once a week, for 2 hours, for 10 weeks	Psy	FF	G	QoL	POSM	24<br>48
Ashing, KT, et al., 2019[42]	USA	breast	40 (100)	20 (100)	20 (100)	cognitive-behavioral	30-40 minutes sessions with a booster session 1 month after complication of phone sessions	HcP	T	I	QoL	FACT-G	16
Esplen MJ, et al., 2018[43]	Canada	breast	194 (100)	131 (100)	63 (100)	cognitive behavior therapy	once a week for 90 min	Psy	FF	G		FACT-BC, FACT-G	8<br>24<br>48
Burns DS, et al., 2001[44]	USA	mixed	8 (100)	4 (100)	4 (100)	bonny method of GIM	once a week for ten weeks, for one to two hours	Psy	FF	I	QoL	QoL-CV	10<br>16
Dos Santos, M, et al., 2020[45]	France	mixed	167 (96)	48 (n/a)	51 (n/a)	computer-assisted cognitive rehabilitation	9 standardized sessions (45-60 minutes) over 3 months	Psy	FF	I	QoL	FACT-G	12
Gaston-Johansson, F, et al., 2011[46]	USA	breast	73 (100)	38 (100)	35 (100)	comprehensive coping strategy program	1.5 hour session with additional 5 follow ups for 20 minutes each time	HcP	FF	I	QoL	QOLI-CV	48

Author	Country	Cancer type	Sample size (female% of total)	Sample size (female% of intervention)	Sample size (female% of control)	Intervention name	Intervention's duration	Intervention's provider	Intervention's environment	Intervention type	Outcome	Questionnaire	Time of the measurement in weeks
**Garssen B, et al., 2012[47]**	Netherlands	breast	70 (100)	34 (100)	36 (100)	stress management training	4 sessions of 45-60 minutes each	Psy	FF	I	QoL	EORTC QLQ-C30	2
													5
													4
													13
**Hall S ,et al., 2011[48]**	UK	mixed	45 (51)	22 (59)	23 (43)	dignity therapy: a brief palliative care Psychotherapy	not sure (maybe 30-60 minutes) of 1 session	Psy	FF	I	QoL	EQ-5D, QoL	1
													4
**Nápoles AM, et al. 2015[49]**	USA	breast	151 (100)	76 (100)	75 (100)	cognitive–behavioral stress management program integrating evidence-based and community best practices	eight weekly, 90 minute sessions	Peer	FF	I	QoL	FACT-B	12
													24
**Gao Q, et al. 2020[50]**	China	gastric	80 (22)	40 (18)	40 (28)	health education intervention program	N/A	N	FF	I	QoL	EORTC QLQ-STO22	4
**Dieng M, et al. 2020[51]**	Australia	skin	151 (45)	70 (29)	81 (59)	psychological intervention designed to reduce fear of cancer recurrence	3 sessions within a 4-week period	M (SH, Psy)	M (SH, T)	M (I, SH)	QoL	AQoL-8D	26
													48
**Karlsen RV et al., 2021[52]**	Denmark	prostate	35 (0)	16 (0)	19 (0)	n/a	up to six one hours couple counseling sessions	HcP	FF	I	QoL	SF-36 PCS, SF-36 MCS	32
													48
	Netherlands	glioma	89 (58)	45 (58)	44 (59)	self help course		HcP	O	I	QoL		6

Author	Country	Cancer type	Sample size (female% of total)	Sample size (female% of intervention)	Sample size (female% of control)	Intervention name	Intervention's duration	Intervention's provider	Intervention's environment	Intervention type	Outcome	Questionnaire	Time of the measurement in weeks
**Boele FW, et al ., 2017[53]**							5 weeks online guided self help course with an online support from a coach					SF-36 PCS, SF-36 MCS	12
**Braeken APB, et al. 2013[54]**	Netherlands	mixed	280 (n/a)	136 (n/a)	144 (n/a)	n/a	during treatment	HcP	FF	I	QoL	EORTC QLQ-C30	12
													48
**Cheung YL, et al. 2002[55]**	Hong kong	colorectal	59 (32)	29 (33)	30 (31)	progressive muscle relaxation training (PMRT)	PMRT practice at home for 2-3 times a week for 10 weeks. before starting a teaching sessions face to face, and audiocassette for a routine PMRT session	HcP	FF	I	QoL	WHO–QOL	5
													10
**Van Der Hout, AVD, et al. 2019[56]**	Netherlands	mixed	624 (51)	320 (49)	304 (52)	web-based eHealth application Oncokompas	n/a	SH	SH	SH	QoL	HRQOL	1
													12
													24
**Ham, K, et al. 2019[57]**	South korea	mixed	42 (86)	21 (86)	21(86)	app-based cognitive behavioral therapy program	1 session per day for 10 weeks at home, excluding weekends, composed of 48 sessions which takes approximately 10–15 min to complete.	SH	O	I	QoL	SF-36	10
**Ding, K, et al. 2020[58]**	China	breast	74 (100)	34 (100)	40 (100)	n/a	3 to 6 sessions of individual therapy, each lasting 30 minutes	HcP	FF	I	QoL	FACT-B, FACT-Cog	4

Author	Country	Cancer type	Sample size (female% of total)	Sample size (female% of intervention)	Sample size (female% of control)	Intervention name	Intervention's duration	Intervention's provider	Intervention's environment	Intervention type	Outcome	Questionnaire	Time of the measurement in weeks
**Fillion, L, et al. 2008[59]**	Canada	breast	87 (100)	44 (100)	43 (100)	psychoeducation and physical activity	4 weekly group meetings of 2.5 hours (1,5 hours psychoeducative and 1 hour walking training) and 1x session(5-15 minutes)	N	M (FF, T)	G	QoL	SF-12	4<br>12
**Gonzalez-Hernandez, E, et al. 2018[60]**	Spain	breast	56 (100)	28 (100)	28 (100)	cognitively-based compassion training exercises, and guided meditations.	8 weeks, weekly 2 hour session	Psy	FF	G	QoL	FACT-B	8<br>24
**Breitbart, W, et al. 2018[61]**	USA	mixed	168 (75)	94 (74)	74 (75)	individual meaning-centered psychotherapy	7	HcP	FF	I	QoL	MQOL	4<br>8<br>16
**Breitbart, W, et al. 2018[61]**	USA	mixed	160 (70)	86 (66)	74 (75)	supportive psychotherapy (SP)	7	HcP	FF	G	QoL	MQOL	4<br>8<br>16
**van der Meulen, IC, et al., 2013[62]**	Germany	mixed	179 (30)	88 (30)	91 (30)	problem-focused and patient driven counseling	maximum of six counseling sessions of 45–60 min every 2 months over a period of 1 year, starting 6 weeks after the completion of cancer treatment	N	FF	I	QoL	EORTC QLQ-C30	48
**Turner, J, et al., 2016[63]**	Australia	mixed	469 (70)	247 (74)	222 (66)	supportive sessions	four individual sessions, each up to a 30-min duration	HcP	M (FF, T)	I	QoL	EQ-5D-5L, SCNS-S34, FACT-G<br>SCNS-S34	10

Author	Country	Cancer type	Sample size (female% of total)	Sample size (female% of intervention)	Sample size (female% of control)	Intervention name	Intervention's duration	Intervention's provider	Intervention's environment	Intervention type	Outcome	Questionnaire	Time of the measurement in weeks
												FACT-G	
**Reese, JB, et al., 2021[64]**	USA	breast	144 (100)	73 (100)	71(100	multimedia intervention	n/a	Psy	FF	I	QOL	FACT-Q	8
**Powell, CB, et al., 2008[65]**	USA	gynecological	64 (100)	21 (100)	43 (100)	counselling	one-time intervention	Psy	FF	I	QOL	FACIT	12
**Nikbakhsh, N, et al., 2018[66]**	Iran	breast	40 (100)	20 (100)	20 (100)	supportive therapy	weekly for 8 weeks, each 45 min	Psy	FF	G	QoL	WHO-QOL-BREF	12
**Fang, P, et al.,2020[67]**	China	gastric	120 (40)	60 (40)	60 (40)	supportive care and humanistic care	3x40 min during hospital stay	N	FF	I	QOL	n/a	4
**Mihuta, ME, et al., 2018[68]**	Australia	mixed	65 (100)	32 (100)	33 (100)	cognitive behavioral therapy	4 session (1 per week)	HcP	O	G	QOL	FACT-Cog, EORTC-QLQ	4
**McLachlan, SA, et al., 2001[69]**	Australia	mixed	450 (41)	296 (n/a)	154 (n/a)	consultation	average 15 minute session, only one session	N	FF	I	QOL	EORTC QLQ-C30	24
**Edmonds et al.,1999[70]**	Canada	breast	66 (100)	30 (100)	36 (100)	psychoeducation	2 h per week/35 weeks	HcP	FF	G	QOL	FLIC	54
**Li, J, et al., 2019[71]**	China	colorectal	498 (n/a)	249 (n/a)	249 (n/a)	health education, psychological counseling	n/a	N	T	I	QoL	QLQ-C30	12
**Elyasi, F, et al., 2021[72]**	Iran	breast	30 (100)	15 (100)	15 (100)	CBT or hypnosis<br>hypnosis only	8x one-hour treatment sessions	HcP	FF	I	QoL	EORTC – BR 23	24
**Ferguson, RJ, et al., 2012[73]**	USA	breast	40 (100)	19 (100)	21 (100)	brief cognitive behavioral therapy	four biweekly individual office visits 30–50 minutes	HcP	FF	I	QoL	QOL-CS	8

Author	Country	Cancer type	Sample size (female% of total)	Sample size (female% of intervention)	Sample size (female% of control)	Intervention name	Intervention's duration	Intervention's provider	Intervention's environment	Intervention type	Outcome	Questionnaire	Time of the measurement in weeks
**Willems, R, et al., 2016[74]**	Netherlands	mixed	409 (81)	188 (81)	221 (81)	psychosocial support trough a website	four components divided over two sessions	SH	SH	SH	QoL	EORTC QLQ-C30	24
**Wu, Q, et al., 2021[75]**	China	breast	86 (100)	43 (100)	43 (100)	WeChat, psychological counseling	3 months	N	O	I	QoL	SF-36	12
**Yun et al., 2017[76]**	South Korea	mixed	206 (80)	134 (82)	72 (75)	health education, leadership, and coaching education, leadership, and coaching	16 sessions of tele-coaching were conducted: 30 min per week for 12 sessions, 30 min per 2 weeks for 2 sessions, and 30 min per month for 2 sessions were offered for the intervention	HcP	T	I	QoL	EORTC QLQ-C30	48
**Zhao, X, et al., 2021[77]**	China	glioma	103 (n/a)	52 (n/a)	51 (n/a)	cognitive behavioral therapy	twice a week	N	FF	I	QoL	QLQ-C30	12
**Zhao,X, et al. 2015[78]**	China	lung	124 (n/a)	62 (n/a)	62 (n/a)	supportive psychological intervention	n/a	N	FF	I	QoL	QLQ-C30	0,3
**Rosen, KD, et al., 2018[79]**	USA	breast	112 (100)	57 (100)	55 (100)	mindfulness	training fully accessible	SH	SH	SH	QoL	FACT-B	5
**Ruiz-Vozmediano, J, et al.,2020[80]**	Spain	breast	63 (100)	31 (100)	32 (100)	mindfulness	twice-weekly 90-minute session	Psy	FF	G	QoL	EORTC QLQ-C30	24
**Von Ah, D, et al.,2012[81]**	USA	breast	89 (100)	n/a (100)	n/a (100)	memory training to improve cognitive functions	10x1-hour training session, over 6-8 weeks	HcP	FF	G	QoL	QOL-CS, QOL-CV, SF-36	8

Author	Country	Cancer type	Sample size (female% of total)	Sample size (female% of intervention)	Sample size (female% of control)	Intervention name	Intervention's duration	Intervention's provider	Intervention's environment	Intervention type	Outcome	Questionnaire	Time of the measurement in weeks
						speed of processing training to improve cognitive functions							
**Lengacher, CA, et al., 2009[82]**	USA	breast	82 (100)	40 (100)	42 (100)	mindfulness-based stress reduction	weekly 2-hours session	Psy	FF	G	QoL	SF-36	6
**Beatty, L. et al. 2015[83]**	Australia	mixed	60 (95)	30 (90)	30 (100)	self-guided Web-based cognitive behavior, cancer coping	once a week, six weeks	HcP	O	I	QoL	EORTC-QLQ-C30	6
													13,03
													26,07
**Chu, X. et al, 2020[84]**	China	breast	84 (100)	42 (100)	42 (100)	mindfulness-based cognitive therapy (MBCT)	MBCT training for 8 weeks, 2 hours per week homework for each class, which takes 20 to 45 min to complete every day.	HcP	FF	I	QoL	FACT-G	8
													12
**Berglund, G. et al. 2007[85]**	Sweden	prostate	189 (0)	39 (0)	150 (0)	"Between Men" programme	seven weekly sessions (60 minutes)	HcP	FF	G	QoL	EORTC QLQ-C30	48
**Dirksen, S. et al, 2007[86]**	USA	breast	72 (100)	34 (100)	38 (100)	cognitive behavioral therapy	Total Ten weeks; 2-week pre-treatment; a 6-week treatment four times per week; 2-week post-treatment- conducted through individual weekly phone sessions. The first class was approximately 2 hours in length, the remaining three classes were an hour or less, and the phone sessions lasted	N	T	I	QoL	FACT-G, FACT-B SWB	10

Author	Country	Cancer type	Sample size (female% of total)	Sample size (female% of intervention)	Sample size (female% of control)	Intervention name	Intervention's duration	Intervention's provider	Intervention's environment	Intervention type	Outcome	Questionnaire	Time of the measurement in weeks
							approximately 15 minutes.						
**Dolbeault, S. et al. 2008[87]**	France	breast	168 (100)	81 (100)	87 (100)	psychoeducational intervention	once a week (2hrs) for eight weeks	Psy	FF	G	QoL	EORTC QLQ-C30	1<br>4
**Aranda, S. et al 2006[88]**	Australia	breast	60 (100)	30 (100)	30 (100)	psychoeducational intervention	once a week, 1.5 hours (1 hour meeting and 30 min call)	N	M (FF, T)	I	QoL	EORTC QLQ-30	4<br>13
**Baoyindelige er, L.Z. et al. 2020[89]**	China	esophageal	130 (n/a)	65 (34)	65 (30)	psychological nursing care intervention	n/a	N	FF	I	QoL	SF-36	2
**Armes, J. et al. 2007[90]**	UK	mixed	53 (60)	26 (64)	27 (56)	psychoeducational intervention	3 occasions 1 hour	HcP	FF	I	QoL	EORTC QLQ- C30	n/a<br>4<br>39
**Compen F. et al, 2018[91]**	Netherlands	mixed	155 (86)	77 (43)	78 (43)	Mindfulness-based cognitive therapy (MBCT)	eight weekly 2.5-hour group sessions + a 6-hour silent day, and daily home practice assignments	Psy	FF	G	QoL	SF-12	8
						(eMBCT) individual internet-based MBCT			O	I			
	Australia	prostate	331 (0)	165 (0)	166 (0)	consultations		N	FF	G	QoL	EPIC-26	7

Author	Country	Cancer type	Sample size (female% of total)	Sample size (female% of intervention)	Sample size (female% of control)	Intervention name	Intervention's duration	Intervention's provider	Intervention's environment	Intervention type	Outcome	Questionnaire	Time of the measurement in weeks
Schofield, P, et al., 2016[92]							beginning of treatment (week 1), mid-treatment (week 4), treatment completion (week 7) and 6-weeks post-treatment (week 13)						24
Hauffman, A, et al., 2020[93]	Sweden	mixed	245 (71)	124 (75)	121 (67)	psychoeducative lectures	continuous access for the material	M (HcP, SH)	SH	SH	QoL	EORTC QLQ-C30	4
													16
													28
													40
Jelvehzadeh, F, et al., 2022[94]	Iran	breast	48 (100)	24 (100)	24 (100)	n/a	8 sessions for 8 weeks, each session lasts for 120 min	HcP	FF	G	QoL	MQOL	8
													12
Qiu, H, et al., 2018[95]	China	breast	294 (100)	98 (100)	196 (100)	cognitve behavioral therapy	nine sessions for 12 weeks	Psy	FF	I	QoL	FACT-B	4
													12
													24
						self-care management							4
													12
													24
Girgis, A, et al., 2009[96]	Wales	mixed	356 (n/a)	n/a	n/a	supportive care interventions general oncologist/practiti oner model	baseline and at 3 and 6 months	HcP	M (FF, O)	I	QoL	EORTC QLQ-C30	12
									M (FF, O)				24

Author	Country	Cancer type	Sample size (female% of total)	Sample size (female% of intervention)	Sample size (female% of control)	Intervention name	Intervention's duration	Intervention's provider	Intervention's environment	Intervention type	Outcome	Questionnaire	Time of the measurement in weeks
						supportive care interventions telephone caseworker			T				12
									T				24
**Guan, S, et al., 2019[97]**	China	mixed	100 (100)	50 (100)	50 (100)	psychological intervention	5-year prognostic follow-up were recorded.	HcP	FF	I	QoL	EORTC-QLQ-C30	8
**Guo, Z, et al., 2013[98]**	China	mixed	178 (58)	89 (58)	89 (58)	psychoeducation, cognitive-behavioral therapy, supportive–expressive therapy	two 60-min face to face interviews each week	HcP	FF	G	QoL	EORTC QLQ-C30	2
											S	n/a	n/a
**Heiney, SP, et al., 2003[99]**	USA	breast	66 (100)	33 (100)	33 (100)	n/a	6 weekly sessions that were 90 minutes long, conference phone call	Psy	T	G	QoL	EORTC QLQ-BR23	6
													16
**Henderson, VP, et al., 2013[100]**	USA	breast	111 (100)	53 (100)	58 (100)	mindfulness based stress reduction (MBSR)	(1) an introductory meeting for one group; (2) 8 weekly 2.5- to 3.5-hour sessions in Gs of 25 to 30 women, with an additional 7.5-hour intensive retreat session given in the sixth week; and (3) 3 additional 2-hour sessions at monthly intervals following completion of the MBSR intervention	HcP	FF	G	QoL	FACT-B	16

Author	Country	Cancer type	Sample size (female% of total)	Sample size (female% of intervention)	Sample size (female% of control)	Intervention name	Intervention's duration	Intervention's provider	Intervention's environment	Intervention type	Outcome	Questionnaire	Time of the measurement in weeks
**Henderson, VP, et al., 2012[101]**	USA	breast	111 (100)	53 (100)	58 (100)	Mindfulness based stress reduction (MBSR)	(1) an introductory meeting for an introductory meeting for one group;<br>(2) the 8-week standard MBSR intervention to a heterogeneous G of patients with a variety of medical/psychiatric disorders, seven weekly 2.5 to 3.5-hour sessions and one 7.5 hour intensive silent retreat session in the 6th week;<br>(3) three monthly 2-hour sessions for BRIDGES-only participants following completion of the MBSR	HcP	FF	G	QoL	FACT-B	16
**Hoffman, CJ, et al., 2013[102]**	UK	breast	229 (100)	n/a (100)	n/a (100)	mindfulness-based stress reduction	9 weekly, 2 hours , except the 1st and last classes were 2.25 hours, plus one 6-hour day of mindfulness in week 6. Home practice was delivered by four 45-minute compact discs of formal mindfulness practices and a manual. Participants were asked to practice for 40 to 45 minutes for 6 or 7 d/wk	HcP	M (FF, SH)	M (G, SH)	QoL	FACT-B<br>FACT PWB<br>FACT SWB<br>FACT EWB<br>FACT FWB	8; 12

Author	Country	Cancer type	Sample size (female% of total)	Sample size (female% of intervention)	Sample size (female% of control)	Intervention name	Intervention's duration	Intervention's provider	Intervention's environment	Intervention type	Outcome	Questionnaire	Time of the measurement in weeks
**Johansson, B, et al., 2008[103]**	Sweden	mixed	481 (58)	n/a (n/a)	n/a (n/a)	individual support	The median number of contacts was 3 (minimum–maximum: 1–24).	Psy	M (FF, T)	I	QoL	EORTC QLQ C-30	12<br>24<br>48<br>96
**Kim, YH, et al., 2017[104]**	South Korea	breast	60 (100)	30 (100)	30 (100)	psychological intervention	6 weeks, as patients visited the clinic every 3 weeks for their chemotherapy, all the data were collected during these visits.	N	M (FF, T)	I	QoL	EORTC QLQ C-30	6<br>9
**Klafke, N, et al., 2019[105]**	Germany	breast	231 (100)	112 (100)	113 (100)	supportive care intervention using complementary and integrative medicine	weekly, maximum time of the intervention was set to 24 weeks	N	FF	I	QoL	EORTC-QLQ-C30	12<br>24<br>48
**Rahmani, S, et al. 2015[106]**	Iran	breast	24 (100)	12 (100)	12 (100)	mindfulness based stress reduction	once a week in a 2-hour session for 8 weeks	Psy	FF	G	QoL	EORTC QLQ-C30, EORTC QLQ-BR23	8<br>16
**Reich, RR, et al. 2016[107]**	USA	breast	322 (100)	167 (100)	155 (100)	mindfulness based stress reduction	six-week, two-hour per week	Psy	FF	G	QoL	SF-36	6<br>12
**Savard, J, et al., 2006[108]**	Canada	breast	37 (100)	21 (100)	16 (100)	cognitive therapy	weekly sessions of 60 to 90 min for 8 weeks plus 3 booster sessions every 3 weeks	Psy	FF	I	QoL	QLQ-C30	8

Author	Country	Cancer type	Sample size (female% of total)	Sample size (female% of intervention)	Sample size (female% of control)	Intervention name	Intervention's duration	Intervention's provider	Intervention's environment	Intervention type	Outcome	Questionnaire	Time of the measurement in weeks
**Schellekens, MPJ, et al. 2016[109]**	Canada	breast	139 (100)	69 (100)	70 (100)	mindfulness-based cancer recovery	8 weekly group sessions of 90 min each plus a 6-h silent retreat between weeks 6 and 7 for a total of 18 contact hours	HcP	FF	G	QoL	FACT-B	8
**van den Berg, SW, et al. 2015[110]**	Netherlands	breast	150 (100)	70 (100)	80 (100)	cognitive behavioral therapy	sixteen fully automated weekly modules (16 weeks)	SH	SH	SH	QoL	EORTC QLQ-C30	4
**Wang, TJ, et al., 2023[111]**	Taiwan	colorectal	142 (41)	70 (33)	72 (49)	self-management support program	two personal skills training sessions, and 12 follow-up telephone calls	SH	M (FF, T)	SH	QoL	FACT-G	8
													16
													24
**Xia, S, et. al., 2023[112]**	Ireland	colorectal	160 (n/a)	80 (41)	80 (34)	cognitive behavioral stress management	10 times, weekly 120 minutes	HcP	FF	G	QoL	EORTC QLQ-C30	4
													12
													24
**Peng, L, et al., 2022[113]**	China	breast	57 (100)	28 (100)	29 (100)	mindfulness-based stress reduction	6 week, weekly 1,5 h plus daily 30 mins	Psy	FF	G	QoL	EORTC QLQ-C30	0,1
													4
**Kissane, DW, et al., 2023[114]**	Australia	mixed	107 (n/a)	55 (73)	52 (79)	meaning and purpose theraphy	six 60 mins session every two weeks	HcP	FF	I	QoL	MQOL-revised	12
													24

Author	Country	Cancer type	Sample size (female% of total)	Sample size (female% of intervention)	Sample size (female% of control)	Intervention name	Intervention's duration	Intervention's provider	Intervention's environment	Intervention type	Outcome	Questionnaire	Time of the measurement in weeks
**Zaman, ACGNM, et al., 2021**[115]	Netherlands	mixed	88 (na)	42 (36)	46 (33)	tailored work-related intervention	3 times 30 minutes in the beggining, then at 6 month and at 9 month	HcP	M (FF, T)	I	QoL	SF-12	12
**Kim, SH, et al., 2021**[116]	South Korea	breast	94 (100)	47 (100)	47 (100)	partnership-based, needs tailored self-management support intervention	10 session, 15-20 minutes over 7 weeks	N	T	I	QoL	SF-36	8
**McCusker, J, et al., 2021**[117]	Canada	mixed	145 (n/a)	121 (75)	124 (82)	Can Direct-depression self-care intervention	15 calls for 15 mins	HcP	T	I	QoL	SF-12	12
													24
**Lee, JT, et al., 2022**[118]	Taiwan	mixed	60 (100)	30 (100)	30 (100)	mindfulness-based stress management	12 week, 2 hours weekly	Psy	FF	G	QoL	FACT-G	12
													24
**Lu, Z, et al., 2021**[119]	China	mixed	326 (n/a)	214 (30)	114 (32)	early supportive care intervention	n/a	Psy	FF	I	QoL	EORTC QLQ-C30	9
											survival	n/a	n/a
**Kirkegaard, AM, et al. 2023**[120]	Denmark	breast	198 (100)	96 (100)	102 (100)	cognitive existential therapy	2 times 6 hours session, plus eight session 2,5 hours each	Psy	FF	G	survival	n/a	n/a
**Cengiz, HO, et al., 2023**[121]	Turkey	breast	65 (100)	32 (100)	33 (100)	mindfulness-based therapy	8 weeks, weekly, 45-60 mins sessions	HcP	O	G	QoL	FACT-G, FACT-Sp	8

Author	Country	Cancer type	Sample size (female% of total)	Sample size (female% of intervention)	Sample size (female% of control)	Intervention name	Intervention's duration	Intervention's provider	Intervention's environment	Intervention type	Outcome	Questionnaire	Time of the measurement in weeks
**Seliniotaki, T, et al., 2021[122]**	Greece	breast	53 (100)	27 (100)	26 (100)	stress management program	8 session weekly, 45 minutes each	Psy	FF	I	QoL	EORTC QLQ-C30	8
**Articles included in the systematic review**													
**Anderson, K. et al, 2006[123]**	USA	mixed	57 (79)	44 (n/a)	13 (n/a)	n/a	practice at least 5 times a week (for each of 2 weeks) for approximately 20 minutes per session.	SH	SH	SH	QoL	FACT-G	2,5<br>8,5
**Björneklett, HG, et al., 2012[124]**	Sweden	breast	383 (100)	n/a (100)	n/a (100)	psychosocial support intervention	4 months after the end of adjuvant treatment and ran for 7 days, followed by a 4-day follow-up 2 months after the initial visit	HcP	FF	G	QoL	EORTC QLQ-C30, EORTC QLQ-BR23	8<br>24<br>48
**Børøsund, E. et al. 2020[125]**	Norway	mixed	172 (82)	n/a (n/a)	n/a (n/a)	n/a	10 modules on the app, practice content for at least 30 minutes per day	SH	SH	SH	QoL	HRQoL (RAND-36)	12
**Carbajal-López, E. B. et al. 2020[126]**	Mexico	stomach	27 (59)	13 (n/a)	14 (n/a)	internet-delivered cognitive behavioral stress management intervention and psychoeducation program	once a week for two hours	HcP	O	I	QoL	EORTC QLQ-30	5<br>13

Author	Country	Cancer type	Sample size (female% of total)	Sample size (female% of intervention)	Sample size (female% of control)	Intervention name	Intervention's duration	Intervention's provider	Intervention's environment	Intervention type	Outcome	Questionnaire	Time of the measurement in weeks
**Rahmani, S, et al. 2014[127]**	Iran	breast	36 (100)	n/a (100)	n/a (100)	mindfulness based stress reduction	once a week in a 2-hour session for 8 weeks	Psy	FF	G	QoL	EORTC QLQ-C30, EORTC QLQ-BR23	8<br>16
						metacognition therapy							8<br>16
**Carlson, L. et al, 2013[128]**	Canada	breast	165 (100)	111 (100)	54 (100)	mindfulness-based cancer recovery	8 weekly group sessions of 90 minutes each plus a 6-hour workshop between weeks 6 and 7 for a total of 18 contact hours	HcP	FF	G	QoL	FACT-B	8
						supportive-expressive therapy	12 weekly sessions of 90 minutes each	Psy					
**Ell, K, et al., 2008[129]**	USA	mixed	472 (100)	242 (100)	230 (100)	Alleviating Depression Among Patients With Cancer psychotherapy	weekly sessions ranging from 6 to 12 weeks	HcP	T	I	QOL	FACT-G	48
**Lapid, MI, et al., 2007[130]**	USA	geriatric	33 (100)	17 (100)	16 (100)	psychosocial intervention	in 4 weeks: 8 times 90 minutes session	Psy	FF	G	QoL	LAPAQ	4<br>8<br>27
**Lu, J, et al., 2017[131]**	China	nasopharyngeal	106 (n/a)	53 (n/a)	53 (n/a)	health education and behavior therapy and psychological counseling	n/a	N	FF	I	QoL	EORTC QLQ-C30	n/a<br>24<br>48<br>96

Author	Country	Cancer type	Sample size (female% of total)	Sample size (female% of intervention)	Sample size (female% of control)	Intervention name	Intervention's duration	Intervention's provider	Intervention's environment	Intervention type	Outcome	Questionnaire	Time of the measurement in weeks
**Murphy, MJ, et al., 2019[132]**	Australia	mixed	114 (89)	53 (87)	61 (90)	cognitive behavioral therapy	8 lessons over 16 weeks	HcP	O	I	QoL	FACT-G	16
**Pettiford, J, et al., 2017[133]**	USA	breast	103 (100)	47 (100)	56 (100)	bio-psychosocial intervention program	two occasion 4 hour class	HcP	FF	G	QoL	FACT-B	24
													48
													72
													96
**Poort, H, et al., 2020[134]**	Netherlands	mixed	134 (57)	n/a (n/a)	n/a (n/a)	cognitive behavioral therapy for fatigue	10 sessions, 1 hour each, over 12 weeks	Psy	FF	I	QoL	EORTC QLQ-C30	14
**Ramirez, AG, et al., 2019[135]**	USA	mixed	288 (54)	n/a (53)	n/a (55)	community delivered intervention	3 months, sessions not specified	HcP	M (T, O)	I	QoL	FACT-G	12
													36
													60
**Rottmann, N, et al., 2012[136]**	n/a	mixed	452 (n/a)	208 (70)	244 (59)	psychosocial rehabilitation course	6 day, weekly plus weekly 20 mins sessions	HcP	FF	G	QoL	EORTC QLQ-C30	4,24
**Sharpe, M, et al., 2014[137]**	UK	n/a	500 (n/a)	253 (90)	247 (90)	depression care for people with cancer programme	max 10 session over 4 months	N	FF or T	I	QoL	EORTC-QLQ-C30	12
													36
													48

Author	Country	Cancer type	Sample size (female% of total)	Sample size (female% of intervention)	Sample size (female% of control)	Intervention name	Intervention's duration	Intervention's provider	Intervention's environment	Intervention type	Outcome	Questionnaire	Time of the measurement in weeks
**Sohl, SJ, et al., 2017[138]**	USA	breast	139 (100)	69 (100)	70 (100)	expressive writing	four, 20-min session, two sessions per week spaced 48 h apart	SH	SH	SH	QoL	FACT-B	4
**Van Amstel et al., 2019[139]**	Netherlands	breast	194 (100)	96 (100)	98 (100)	emotional support, education about cancer	every 3 months during the follow-up visits in the first year and every 6 months during the second year of follow-up	N	FF	M (I, T)	QoL	EORTC QLQ C-30	12<br>24<br>36<br>48<br>72<br>96
**Yanez, B, et al., 2015[140]**	USA	prostate	74 (0)	37 (0)	37 (0)	manualized cognitive behavioral stress management, relaxation + stress management techniques	10 weeks , 90 min/sessions	HcP	O	G	QoL	FACT-G	24
**Wenzel, L, et al., 2015[141]**	USA	cervical	203 (100)	115 (100)	88 (100)	psychosocial counseling	included five weekly sessions and a 1-month booster.	HcP	T	I	QoL	FACT-Cx, FACT-TOI, FACT Additional Concerns, FACT-G	36<br>16
**Urech, C, et al., 2018[142]**	Switzerland	mixed	129 (85)	65 (82)	64 (88)	stress management	8 weeks	SH	SH	SH	QoL	FACT-G	8

Author	Country	Cancer type	Sample size (female% of total)	Sample size (female% of intervention)	Sample size (female% of control)	Intervention name	Intervention's duration	Intervention's provider	Intervention's environment	Intervention type	Outcome	Questionnaire	Time of the measurement in weeks
**Burns DS, et al., 2001[44]**	USA	mixed	80 (100)	40 (100)	40 (100)	bonny method of guided imagery and music	once a week for ten weeks, for one to two hours	Psy	FF	I	QoL	QoL-CA	10
													16
**Lerman, R, et al., 2012[143]**	UK	mixed	68 (100)	48 (100)	20 (100)	mindfulness-based stress reduction	once a week for 2 hours class and a single 4 hours weekend retreat held twice	Psy	FF	G	QoL	EORTC QLQ-C30	8

Table 1. Main characteristics of the included studies: Abbreviations: AQoL-8D, Assessment of Quality of Life; BSI-GSI, Brief Symptom Inventory-Global Severity Index; CBT, Cogntive Behavior Therapy CCV, Cuestionario de Calidad de Vida; EORTC QLQ-BR23, European Organization for Research and Treatment of Cancer Quality of Life Questionnaire-Breast Cancer; EORTC-QLQ-C30, European Organization for Research and Treatment of Cancer Quality of Life Questionnaire C-30; EORTC-QLQ-PR25, European Organization for Research and Treatment of Cancer Quality of Life Questionnaire-Prostate Cancer; EORTC QLQ-STO22, European Organization for Research and Treatment of Cancer Quality of Life Questionnaire-Gastric Cancer; EPIC-26 , Expanded Prostate Cancer Index Composite Short-Form; EQ-5D-VA, EuroQol-5D visual analogue scale; EuroQol 5D-5L, EuroQol-5D-5 level; FACIT-Sp, Functional Assessment of Chronic Illness Therapy-Spiritual Well-Being Scale; FACIT, Functional Assessment of Cancer Therapy; FACT-B (Functional Assessment of Cancer Therapy-Breast; FACT-C; Functional Assessment of Cancer Therapy-Colorectal; FACT-Cx, Functional Assessment of Cancer Therapy-Cervix; FACT EWB, Functional Assessment of Cancer Therapy-Emotional Well-being; FACT-F (Functional Assessment of Cancer Therapy-Fatigue; FACT FWB (Functional Assessment of Cancer Therapy-Functional Well-being; FACT-G, Functional Assessment of Cancer Therapy-General; FACT PWB, Functional Assessment of Cancer Therapy-Physical Well-Being; FACT SWB, Functional Assessment of Cancer Therapy- Social Well-Being; FF, Face-to-face; FLIC; Functional Living Index Cancer; G, Group; GHQ-28, General Health Questionnaire; GQOL, Global Quality of Life Scale; HcP(Healthcare Professional; IIEF, International Index of Erectile Function; IPSS, International Prostate Symptom Score; I, Individual; LASA, Spitzer Uniscale and Linear Analogue Self-assessment; MBCT, Mindfulness Based Cognitive Therap; MBSR, Mindfulness Based Stress Reduction; M, Mixed; MQOL, McGill Quality of Life Questionnaire; N, Nurse; O, Online; PCI, Prostate Cancer Index; PCQoL, Prostate Cancer Quality of Life Instrument; POSM, Positive States of Mind; PSQI, Pittsburgh Sleep Quality Index; Psy, Psychologist; QoL-CA, Quality of life Cancer Scale; QOLI-CV, Quality of Life Cancer Version; RAND-36-HRQoL, RAND 36-Item Health Survey- Health-Related Quality of Life; SCNS-S34, Supportive Care Needs Survey; SF-36, Short Form Health Survey; SF-36 MCS, Short Form Health Survey Mental Component Summary; SH, Self-help; T, Telephone; WHO–QOL-BREF, The World Health Organization Quality of Life brief version

Section S2. Statistical Analysis

SURVIVAL

We used hazard rate (HR) with 95% confidence interval (CI) for the effect size measure of survival. The hazard ratios with its CI were extracted from each study directly from the written text or using the published survival functions (Kaplan-Meier plots). *Additional data collection was performed, with the GetData Graph digitizer version 2.26.0.20.*

As we anticipated considerable between-study heterogeneity, a random-effects model was used to pool effect sizes.

Inverse variance weighting method was used to calculate the pooled HR using the point estimates for HR and the standard error calculated from the confidence interval (using standard normal approximation on logarithmic scale).

For the outcomes where the study number was at least 5, a Hartung-Knapp adjustment (Knapp and Hartung 2003; IntHout, Ioannidis, and Borm 2014) was used. Below 5 studies, we do not apply the adjustment.

To estimate the heterogeneity variance measure τ^2 the restricted maximum-likelihood estimator was applied with the Q profile method for confidence interval Veroniki et al. (2016).Additionally, between-study heterogeneity was described by the Higgins&Thompson's I^2 statistics (Higgins and Thompson 2002).

Forest plots were used to graphically summarize the results.

Where applicable we reported the prediction intervals (i.e. the expected range of effects of future studies) of results following the recommendations of IntHout et al. (2016).

Outlier and influence analyses were carried out following the recommendations of Harrer et al. (2021) and Viechtbauer and Cheung (2010). Publication bias was assessed with Egger's test (at significance level 10% as small study number) using the classical Egger's method to calculate the test statistic Sterne et al. (2011). Based on the Cochrane Handbook the following cut-off points are given:

"Thresholds for the interpretation of I2 can be misleading, since the importance of inconsistency depends on several factors. A rough guide to interpretation is as follows:

0% to 40%: might not be important; 30% to 60%: may represent moderate heterogeneity; 50% to 90%: may represent substantial heterogeneity; 75% to 100%: considerable heterogeneity."

All statistical analyses were made with R (R Core Team 2021, v4.1.2) using the *meta* (Schwarzer 2022, v5.2.0)and *dmetar* (Cuijpers, Furukawa, and Ebert 2022, v0.0.9000) packages.

QUALITY OF LIFE:

Due to the differences in the questionnaires, a standardized mean difference (SMD) with 95% confidence interval was used for the effect size measure between intervention and control group. We used Hedges' g as SMD(Hedges 1981).

To calculate SMD we made the following steps:

I. At first we extracted the mean.

II. Then, we extracted or estimated the SD of change as written below:
1. If SD was given we extracted those values.
2. If the standard error (SE) is given, the SD was calculated by multiplying the SE by the square root of the sample size.
3. If the 95% confidence interval of mean was given, the SE was calculated using the

R_{est}= {SD{baseline}^2 +SD_{followup}^2 - SD_{change}^2}{2 * SD_{baseline} *SD_{followup}}

2*t n−1 , where *Ul* is the upper limit and *Ll* is the lower limit of the confidence interval and *t* is the t-value of a corresponding Student t-distribution with *n-1* degrees of freedom and 5% (2,5% symmetrical) p-value. Then the SD was calculated as written before.

As we anticipated considerable between-study heterogeneity, a random-effects model was used to pool effect sizes.

As several studies reported the observed values at different times, we added an additional random-effect based on each study.

We used the following assumptions:

- A continuous-time autoregressive structure is assumed for time dependence. Baseline data also included in the T0 time point.

To estimate the correlation structure, we determined the correlation coefficient (R change) of baseline and follow up values based on the extracted or calculated SD of change if the SD for baseline and follow up results was also given, using the following formula:

$Rest = SD2baseline/$dd

-where *SD*s are the standard deviation of the corresponding situation (baseline, after follow up, change).

If there were at least 3 articles, then the mean of the calculated R values was used as a starting value for estimated correlation coefficient (Rest) in the correlation structure. (Otherwise we used the value 0.65 as in the outcomes with calculated R based on more articles resulting in this value.) In any case we run the final model with different R values to see its effect. Additionally the final model results were modified with cluster-robust test and

confidence interval estimation adjustment using the club sandwich method with small-sample and Satterthwaite approximation.

- To handle the assumed nonlinearity change in time, the squared value of time is also included in the linear model additionally on time as predictor. (Based on descriptive plots and to keep the model simpler and not more overfitted, no higher order component was used.)
- We included the following pre-identified interested predictor variables: *follow-up time value versus baseline value* (as a categorical variable) and one of the following: *provider type*, *environment type*, *intervention type*, *cancer stage*, *duration*). We did models with each predictor separately as it is easier to interpret and summarize the data, although confounding effects may occur and additionally there was limited information on *cancer stage* and *duration*.
- Possible interaction with time of interested variables was also checked using the information criterion (as AIC and BIC values). Our preliminary plan was to include the interaction in the final model if the interaction creates a better model based on the AIC and BIC values (if both AIC and BIC decreased by 2) and the parameter(s) of interaction is significant at level 5%. Based on these criterias we found no evidence to keep interactions in the model.

We report the calculated estimates with 95% CI. For categorical predictor variables an anova (Wald type) result is also given. We made predictions based on the model and we summarized it on forest plots with subgroups for easier interpretations. In this plot SMDs are also shown for individual studies and the overall effect we used the predicted value from the model without the grouping variable.

Inverse variance weighting method was used to calculate the pooled SMD.

To estimate the heterogeneity variance measure τ^2, restricted maximum-likelihood estimator was applied Veroniki et al. (2016).

Publication bias and small study effect was assessed using funnel plots.

All statistical analyses were made with R (R Core Team 2022, v4.2.1) using the following packages: *metafor*(Viechtbauer 2022, v3.4.0) for model calculations, publication bias and influential assessment, *meta* (Schwarzer 2022, v5.5.0) for forest plots, *ggplot2* (Wickham et al. 2022, v3.3.6) for prediction plots.

S2.Overall plots

Figure.S2.1.T12

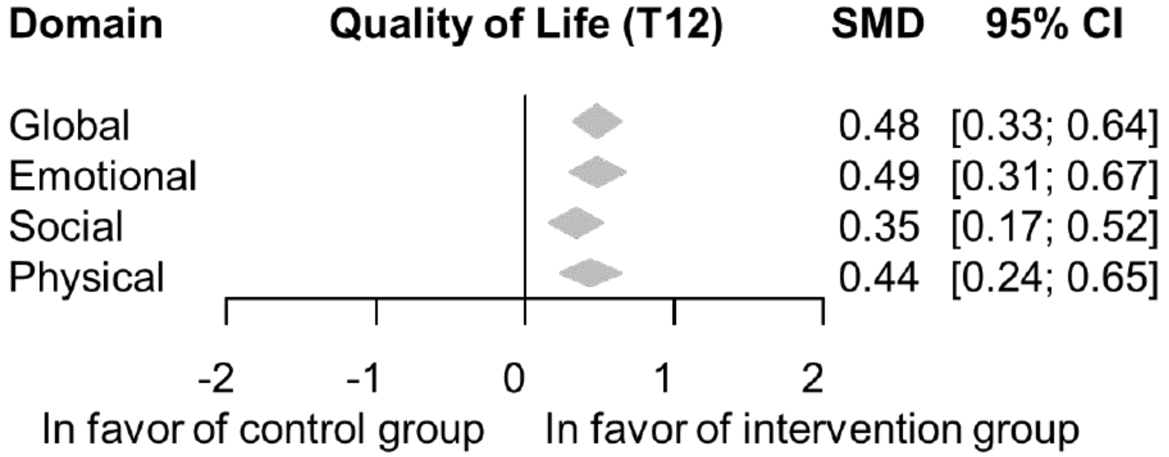

Figure S.2.1. Psychological interventions improve Quality of Life (QoL). Forest plot of pooled results representing that psychological interventions have a significant effect on improving global, emotional, social and physical quality of life compared to the control group estimated at 12 weeks (T12). SMD - Standardized mean difference, CI- confidence interval. Forest plots for individual study effects can be found in S4-S22.

Figure S2.2.T24

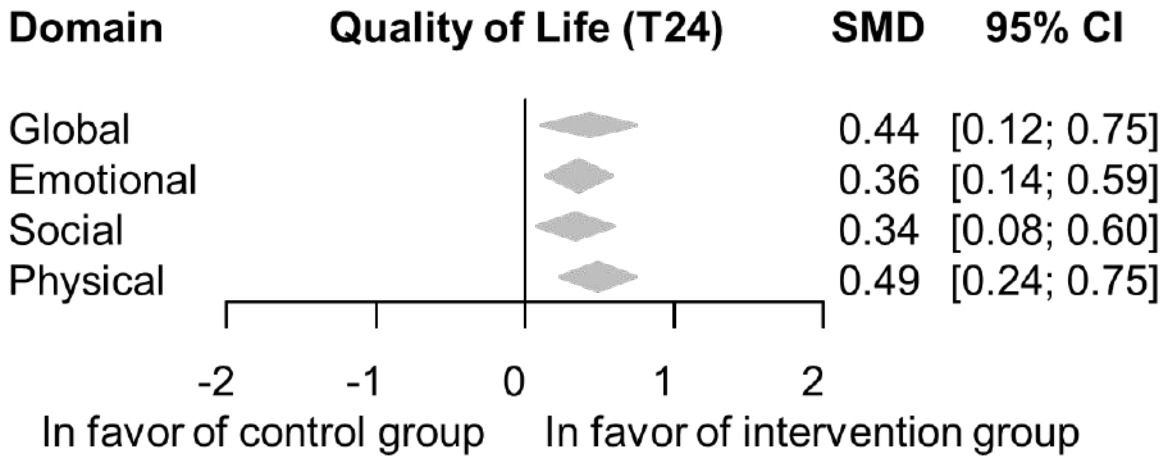

Figure S.2.2. **Psychological interventions improve Quality of Life (QoL).** Forest plot of pooled results representing that psychological interventions have a significant effect on improving global, emotional, social and physical quality of life compared to the control group estimated at 24 weeks (T24). SMD - Standardized mean difference, CI- confidence interval. Forest plots for individual study effects can be found in S4-S22.

Figure S2.3.T48

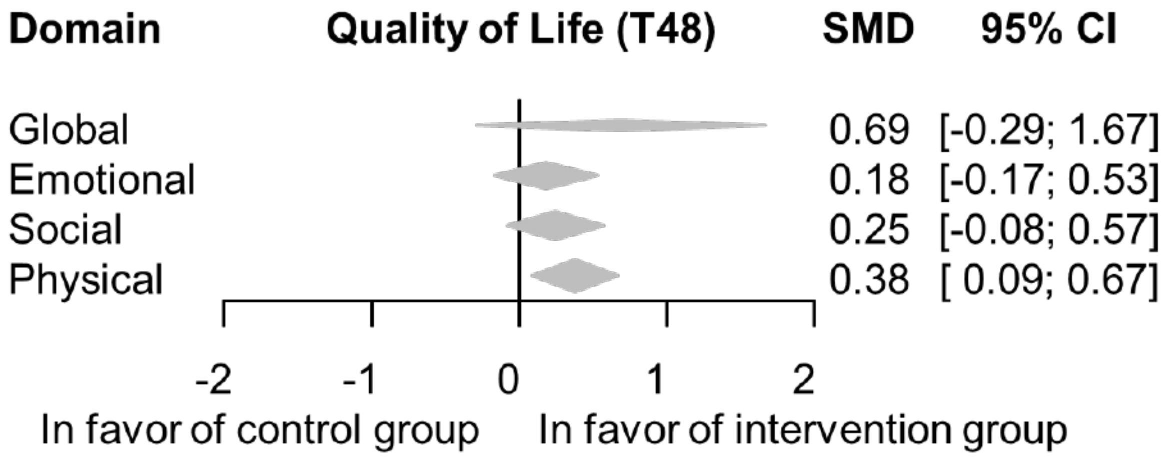

Figure S.2.3. **Psychological interventions improve Quality of Life (QoL).** Forest plot of pooled results representing that psychological interventions have a significant effect on improving physical quality of life compared to the control group estimated at 48 weeks. SMD - Standardized mean difference, CI- confidence interval. Forest plots for individual study effects can be found in S4-S22

S3. Subgroup analysis of Global QoL: Provider

Figure S3.1.T0

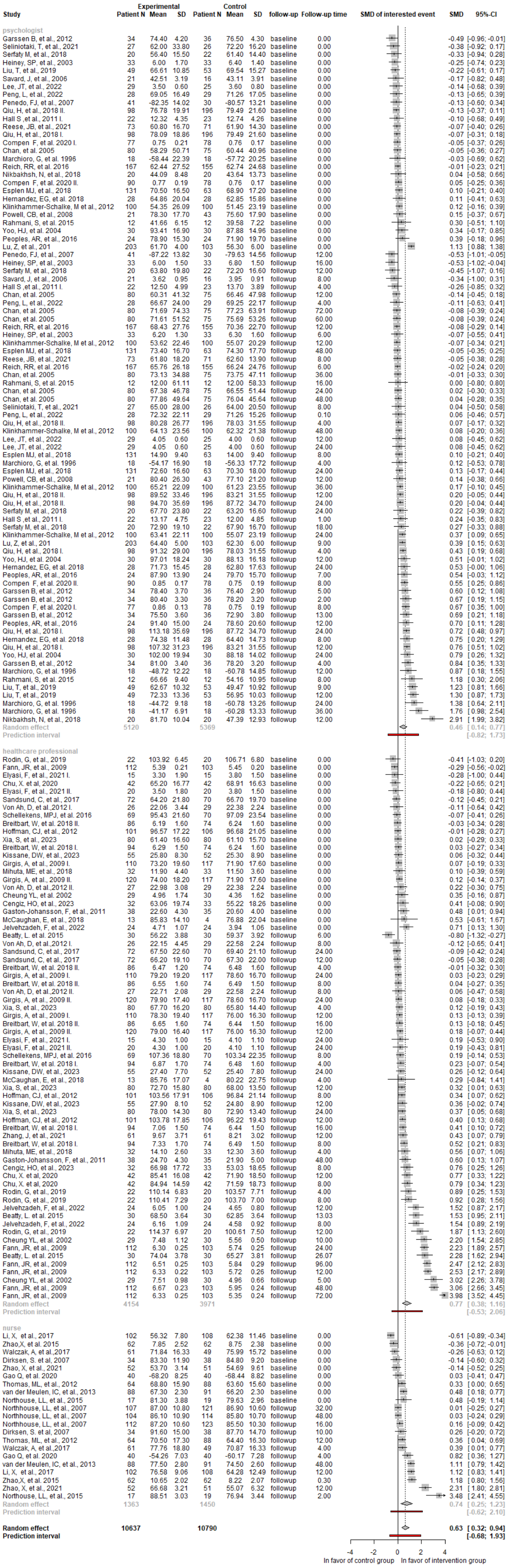

Figure S3.1. Subgroup analysis of the Global QoL. Forest plot represents the difference between the intervention vs. control group in the Global QoL domain with the provider subgroups as predicted at week 0 (post-intervention). SMD - Standardized mean difference, CI - confidence interval.

Figure S3.2.T12

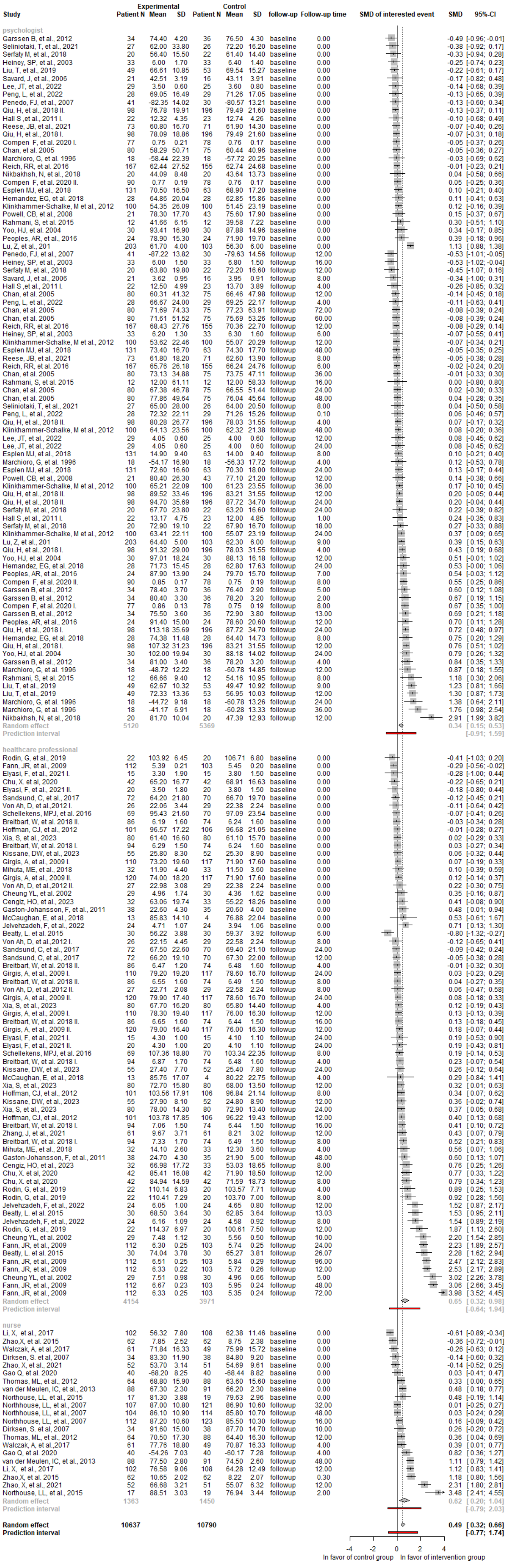

Figure S3.2. Subgroup analysis of the Global QoL Forest plot represents the difference between the intervention vs. control group in the Global QoL domain with the provider subgroups as predicted at week 12 (post-intervention). SMD - Standardized mean difference, CI - confidence interval

Figure S3.3.T24

Study	Experimental Patient N	Experimental Mean	Experimental SD	Control Patient N	Control Mean	Control SD	follow-up	Follow-up time	SMD	95%-CI
psychologist										
Garssen B, et al., 2012	34	74.40	4.20	36	76.50	4.30	baseline	0.00	-0.49	[-0.96; -0.01]
Seliniotaki, T, et al., 2021	27	62.00	33.80	26	72.20	16.20	baseline	0.00	-0.38	[-0.92; 0.17]
Serfaty M, et al., 2018	20	56.40	15.50	22	61.40	14.40	baseline	0.00	-0.33	[-0.94; 0.28]
Heiney, SP, et al., 2003	33	6.00	1.70	33	6.40	1.40	baseline	0.00	-0.25	[-0.74; 0.23]
Liu, T, et al., 2019	49	66.61	10.85	53	69.54	15.27	baseline	0.00	-0.22	[-0.61; 0.17]
Savard, J, et al., 2006	21	42.51	3.19	16	43.11	3.91	baseline	0.00	-0.17	[-0.82; 0.48]
Lee, JT, et al., 2022	29	3.50	0.60	25	3.60	0.80	baseline	0.00	-0.14	[-0.68; 0.39]
Peng, L, et al., 2022	28	69.05	16.49	29	71.26	17.05	baseline	0.00	-0.13	[-0.65; 0.39]
Penedo, FJ, et al., 2007	41	-82.35	14.02	30	-80.57	13.21	baseline	0.00	-0.13	[-0.60; 0.34]
Qiu, H, et al., 2018 II.	98	76.78	19.91	196	79.49	21.60	baseline	0.00	-0.13	[-0.37; 0.11]
Hall S ,et al., 2011 I.	22	12.32	4.35	23	12.74	4.26	baseline	0.00	-0.10	[-0.68; 0.49]
Reese, JB, et al., 2021	73	60.80	16.70	71	61.90	14.30	baseline	0.00	-0.07	[-0.40; 0.26]
Qiu, H, et al., 2018 I.	98	78.09	18.86	196	79.49	21.60	baseline	0.00	-0.07	[-0.31; 0.18]
Compen F, et al. 2020 I.	77	0.75	0.21	78	0.76	0.17	baseline	0.00	-0.05	[-0.37; 0.26]
Chan, et al. 2005	80	58.29	50.71	75	60.44	40.96	baseline	0.00	-0.05	[-0.36; 0.27]
Marchioro, G, et al. 1996	18	-58.44	22.39	18	-57.72	20.25	baseline	0.00	-0.03	[-0.69; 0.62]
Reich, RR, et al. 2016	167	62.44	27.52	155	62.74	24.68	baseline	0.00	-0.01	[-0.23; 0.21]
Nikbakhsh, N, et al., 2018	20	44.09	8.48	20	43.64	13.73	baseline	0.00	0.04	[-0.58; 0.66]
Compen F, et al. 2020 II.	90	0.77	0.19	78	0.76	0.17	baseline	0.00	0.05	[-0.25; 0.36]
Esplen MJ, et al., 2018	131	70.50	16.50	63	68.90	17.20	baseline	0.00	0.10	[-0.21; 0.40]
Hernandez, EG, et al. 2018	28	64.86	20.04	28	62.85	15.86	baseline	0.00	0.11	[-0.41; 0.63]
Klinkhammer-Schalke, M et al., 2012	100	54.35	26.09	100	51.45	23.19	baseline	0.00	0.12	[-0.16; 0.39]
Powell, CB, et al., 2008	21	78.30	17.70	43	75.60	17.90	baseline	0.00	0.15	[-0.37; 0.67]
Rahmani, S, et al. 2015	12	41.66	6.15	12	39.58	7.22	baseline	0.00	0.30	[-0.51; 1.10]
Yoo, HJ, et al. 2004	30	93.41	16.90	30	87.88	14.96	baseline	0.00	0.34	[-0.17; 0.85]
Peoples, AR, et al., 2016	24	78.90	15.30	24	71.90	19.70	baseline	0.00	0.39	[-0.18; 0.96]
Lu, Z, et al., 201	203	61.70	4.00	103	56.30	6.00	baseline	0.00	1.13	[0.88; 1.38]
Penedo, FJ, et al., 2007	41	-87.22	13.82	30	-79.63	14.56	followup	12.00	-0.53	[-1.01; -0.05]
Heiney, SP, et al., 2003	33	6.00	1.50	33	6.80	1.50	followup	16.00	-0.53	[-1.02; -0.04]
Serfaty M, et al., 2018	20	63.80	19.80	22	72.20	16.60	followup	12.00	-0.45	[-1.07; 0.16]
Savard, J, et al., 2006	21	3.62	0.95	16	3.95	0.91	followup	8.00	-0.34	[-1.00; 0.31]
Hall S ,et al., 2011 I.	22	12.50	4.99	23	13.70	3.89	followup	4.00	-0.26	[-0.85; 0.32]
Chan, et al. 2005	80	60.31	41.32	75	66.46	47.98	followup	12.00	-0.14	[-0.45; 0.18]
Peng, L, et al., 2022	28	66.67	24.00	29	69.25	22.17	followup	4.00	-0.11	[-0.63; 0.41]
Chan, et al. 2005	80	71.69	74.33	75	77.23	63.91	followup	72.00	-0.08	[-0.39; 0.24]
Chan, et al. 2005	80	71.61	51.52	75	75.69	53.26	followup	60.00	-0.08	[-0.39; 0.24]
Reich, RR, et al. 2016	167	68.43	27.76	155	70.36	22.70	followup	12.00	-0.08	[-0.29; 0.14]
Heiney, SP, et al., 2003	33	6.20	1.30	33	6.30	1.60	followup	6.00	-0.07	[-0.55; 0.41]
Klinkhammer-Schalke, M et al., 2012	100	53.62	22.46	100	55.07	20.29	followup	12.00	-0.07	[-0.34; 0.21]
Esplen MJ, et al., 2018	131	73.40	16.70	63	74.30	17.70	followup	48.00	-0.05	[-0.35; 0.25]
Reese, JB, et al., 2021	73	61.80	18.20	71	62.60	13.90	followup	8.00	-0.05	[-0.38; 0.28]
Reich, RR, et al. 2016	167	65.76	26.18	155	66.24	24.76	followup	6.00	-0.02	[-0.24; 0.20]
Chan, et al. 2005	80	73.13	34.88	75	73.75	47.11	followup	36.00	-0.01	[-0.33; 0.30]
Rahmani, S, et al. 2015	12	12.00	61.11	12	12.00	58.33	followup	16.00	0.00	[-0.80; 0.80]
Chan, et al. 2005	80	67.38	46.78	75	66.55	51.44	followup	24.00	0.02	[-0.30; 0.33]
Chan, et al. 2005	80	77.86	49.64	75	76.04	45.64	followup	48.00	0.04	[-0.28; 0.35]
Seliniotaki, T, et al., 2021	27	65.00	28.00	26	64.00	20.50	followup	8.00	0.04	[-0.50; 0.58]
Peng, L, et al., 2022	28	72.32	22.11	29	71.26	15.26	followup	0.10	0.06	[-0.46; 0.57]
Qiu, H, et al., 2018 II.	98	80.28	26.77	196	78.03	31.55	followup	4.00	0.07	[-0.17; 0.32]
Klinkhammer-Schalke, M et al., 2012	100	64.13	23.56	100	62.32	21.38	followup	48.00	0.08	[-0.20; 0.36]
Lee, JT, et al., 2022	29	4.05	0.60	25	4.00	0.60	followup	12.00	0.08	[-0.45; 0.62]
Lee, JT, et al., 2022	29	4.05	0.60	25	4.00	0.60	followup	24.00	0.08	[-0.45; 0.62]
Esplen MJ, et al., 2018	131	14.90	9.40	63	14.00	9.40	followup	8.00	0.10	[-0.21; 0.40]
Marchioro, G, et al. 1996	18	-54.17	16.90	18	-56.33	17.72	followup	4.00	0.12	[-0.53; 0.78]
Esplen MJ, et al., 2018	131	72.60	16.60	63	70.30	18.00	followup	24.00	0.13	[-0.17; 0.44]
Powell, CB, et al., 2008	21	80.40	26.30	43	77.10	21.20	followup	12.00	0.14	[-0.38; 0.66]
Klinkhammer-Schalke, M et al., 2012	100	65.21	22.09	100	61.23	23.55	followup	36.00	0.17	[-0.10; 0.45]
Qiu, H, et al., 2018 II.	98	89.52	33.46	196	83.21	31.55	followup	12.00	0.20	[-0.05; 0.44]
Qiu, H, et al., 2018 II.	98	94.70	35.69	196	87.72	34.70	followup	24.00	0.20	[-0.04; 0.44]
Serfaty M, et al., 2018	20	67.70	23.80	22	63.20	16.60	followup	24.00	0.22	[-0.39; 0.82]
Hall S ,et al., 2011 I.	22	13.17	4.75	23	12.00	4.85	followup	1.00	0.24	[-0.35; 0.83]
Serfaty M, et al., 2018	20	72.90	19.10	22	67.90	16.70	followup	18.00	0.27	[-0.33; 0.88]
Klinkhammer-Schalke, M et al., 2012	100	63.41	22.11	100	55.07	23.19	followup	24.00	0.37	[0.09; 0.65]
Lu, Z, et al., 201	203	64.40	5.00	103	62.30	6.00	followup	9.00	0.39	[0.15; 0.63]
Qiu, H, et al., 2018 I.	98	91.32	29.00	196	78.03	31.55	followup	4.00	0.43	[0.19; 0.68]
Yoo, HJ, et al. 2004	30	97.01	18.24	30	88.13	16.18	followup	12.00	0.51	[-0.01; 1.02]
Hernandez, EG, et al. 2018	28	71.73	15.45	28	62.80	17.63	followup	24.00	0.53	[-0.00; 1.06]
Peoples, AR, et al., 2016	24	87.90	13.90	24	79.70	15.70	followup	7.00	0.54	[-0.03; 1.12]
Compen F, et al. 2020 II.	90	0.85	0.17	78	0.75	0.19	followup	8.00	0.55	[0.25; 0.86]
Garssen B, et al., 2012	34	78.40	3.70	36	76.40	2.90	followup	5.00	0.60	[0.12; 1.08]
Garssen B, et al., 2012	34	80.40	3.30	36	78.20	3.20	followup	2.00	0.67	[0.19; 1.15]
Compen F, et al. 2020 I.	77	0.86	0.13	78	0.75	0.19	followup	8.00	0.67	[0.35; 1.00]
Garssen B, et al., 2012	34	75.50	3.60	36	72.90	3.80	followup	13.00	0.69	[0.21; 1.18]
Peoples, AR, et al., 2016	24	91.40	15.00	24	78.60	20.60	followup	12.00	0.70	[0.11; 1.28]
Qiu, H, et al., 2018 I.	98	113.18	35.69	196	87.72	34.70	followup	24.00	0.72	[0.48; 0.97]
Hernandez, EG, et al. 2018	28	74.38	11.48	28	64.40	14.73	followup	8.00	0.75	[0.20; 1.29]
Qiu, H, et al., 2018 I.	98	107.32	31.23	196	83.21	31.55	followup	12.00	0.76	[0.51; 1.02]
Yoo, HJ, et al. 2004	30	102.00	19.94	30	88.18	14.02	followup	24.00	0.79	[0.26; 1.32]
Garssen B, et al., 2012	34	81.00	3.40	36	78.20	3.20	followup	4.00	0.84	[0.35; 1.33]
Marchioro, G, et al. 1996	18	-48.72	12.22	18	-60.78	14.85	followup	12.00	0.87	[0.18; 1.55]
Rahmani, S, et al. 2015	12	66.66	9.40	12	54.16	10.95	followup	8.00	1.18	[0.30; 2.06]
Liu, T, et al., 2019	49	62.67	10.32	53	49.47	10.92	followup	9.00	1.23	[0.81; 1.66]
Liu, T, et al., 2019	49	72.33	13.36	53	56.95	10.03	followup	12.00	1.30	[0.87; 1.73]
Marchioro, G, et al. 1996	18	-44.72	9.18	18	-60.78	13.26	followup	24.00	1.38	[0.64; 2.11]
Marchioro, G, et al. 1996	18	-41.17	6.91	18	-60.28	13.33	followup	36.00	1.76	[0.98; 2.54]
Nikbakhsh, N, et al., 2018	20	81.70	10.04	20	47.39	12.93	followup	12.00	2.91	[1.99; 3.82]
Random effect	5120			5369					0.32	[-0.01; 0.64]
Prediction interval										[-1.00; 1.64]
healthcare professional										
Rodin, G, et al., 2019	22	103.92	6.45	20	106.71	6.80	baseline	0.00	-0.41	[-1.03; 0.20]
Fann, JR, et al., 2009	112	5.39	0.21	103	5.45	0.20	baseline	0.00	-0.29	[-0.56; -0.02]
Elyasi, F, et al., 2021 I.	15	3.30	1.90	15	3.80	1.50	baseline	0.00	-0.28	[-1.00; 0.44]
Chu, X. et al, 2020	42	65.20	16.77	42	68.91	16.63	baseline	0.00	-0.22	[-0.65; 0.21]
Elyasi, F, et al., 2021 II.	20	3.50	1.80	20	3.80	1.50	baseline	0.00	-0.18	[-0.80; 0.44]
Sandsund, C, et al., 2017	72	64.20	21.80	70	66.70	19.70	baseline	0.00	-0.12	[-0.45; 0.21]
Von Ah, D, et al.,2012 I.	26	22.06	3.44	29	22.38	2.24	baseline	0.00	-0.11	[-0.64; 0.42]
Schellekens, MPJ, et al. 2016	69	95.43	21.60	70	97.09	23.54	baseline	0.00	-0.07	[-0.41; 0.26]
Breitbart, W, et al. 2018 II.	86	6.19	1.60	74	6.24	1.60	baseline	0.00	-0.03	[-0.34; 0.28]
Hoffman, CJ, et al., 2012	101	96.57	17.22	106	96.68	21.05	baseline	0.00	-0.01	[-0.28; 0.27]
Xia, S, et al., 2023	80	61.40	16.60	80	61.10	15.70	baseline	0.00	0.02	[-0.29; 0.33]
Breitbart, W, et al. 2018 I.	94	6.29	1.50	74	6.24	1.60	baseline	0.00	0.03	[-0.27; 0.34]
Kissane, DW, et al., 2023	55	25.80	8.30	52	25.30	8.90	baseline	0.00	0.06	[-0.32; 0.44]
Girgis, A, et al., 2009 I.	110	73.20	19.60	117	71.90	17.60	baseline	0.00	0.07	[-0.19; 0.33]
Mihuta, ME, et al., 2018	32	11.90	4.40	33	11.50	3.60	baseline	0.00	0.10	[-0.39; 0.59]
Girgis, A, et al., 2009 II.	120	74.00	18.20	117	71.90	17.60	baseline	0.00	0.12	[-0.14; 0.37]
Von Ah, D, et al.,2012 II.	27	22.98	3.08	29	22.38	2.24	baseline	0.00	0.22	[-0.30; 0.75]
Cheung YL, et al. 2002	29	4.96	1.74	30	4.36	1.62	baseline	0.00	0.35	[-0.16; 0.87]
Cengiz, HO, et al., 2023	32	63.06	19.74	33	55.22	18.26	baseline	0.00	0.41	[-0.08; 0.90]
Gaston-Johansson, F, et al., 2011	38	22.60	4.30	35	20.60	4.00	baseline	0.00	0.48	[0.01; 0.94]
McCaughan, E, et al., 2018	13	85.83	14.10	4	76.88	22.04	baseline	0.00	0.53	[-0.61; 1.67]
Jelvehzadeh, F, et al., 2022	24	4.71	1.07	24	3.94	1.06	baseline	0.00	0.71	[0.13; 1.30]
Beatty, L. et al. 2015	30	56.22	3.88	30	59.37	3.92	followup	6.00	-0.80	[-1.32; -0.27]
Von Ah, D, et al.,2012 I.	26	22.15	4.45	29	22.58	2.24	followup	8.00	-0.12	[-0.65; 0.41]
Sandsund, C, et al., 2017	72	67.50	22.60	70	69.40	21.10	followup	24.00	-0.09	[-0.42; 0.24]
Sandsund, C, et al., 2017	72	66.20	19.10	70	67.30	22.00	followup	12.00	-0.05	[-0.38; 0.28]
Breitbart, W, et al. 2018 II.	86	6.47	1.20	74	6.48	1.60	followup	4.00	-0.01	[-0.32; 0.30]
Girgis, A, et al., 2009 I.	110	79.20	19.20	117	78.60	16.70	followup	24.00	0.03	[-0.23; 0.29]
Breitbart, W, et al. 2018 II.	86	6.55	1.60	74	6.49	1.50	followup	8.00	0.04	[-0.27; 0.35]
Von Ah, D, et al.,2012 II.	27	22.71	2.08	29	22.58	2.24	followup	8.00	0.06	[-0.47; 0.58]
Girgis, A, et al., 2009 II.	120	79.90	17.40	117	78.60	16.70	followup	24.00	0.08	[-0.18; 0.33]
Xia, S, et al., 2023	80	67.70	16.20	80	65.80	14.40	followup	4.00	0.12	[-0.19; 0.43]
Girgis, A, et al., 2009 I.	110	78.30	19.40	117	76.00	16.30	followup	12.00	0.13	[-0.13; 0.39]
Breitbart, W, et al. 2018 II.	86	6.65	1.60	74	6.44	1.50	followup	16.00	0.13	[-0.18; 0.45]
Girgis, A, et al., 2009 II.	120	79.00	16.40	117	76.00	16.30	followup	12.00	0.18	[-0.07; 0.44]
Elyasi, F, et al., 2021 I.	15	4.30	1.00	15	4.10	1.10	followup	24.00	0.19	[-0.53; 0.90]
Elyasi, F, et al., 2021 II.	20	4.30	1.00	20	4.10	1.10	followup	24.00	0.19	[-0.43; 0.81]
Schellekens, MPJ, et al. 2016	69	107.36	18.80	70	103.34	22.35	followup	8.00	0.19	[-0.14; 0.53]
Breitbart, W, et al. 2018 I.	94	6.87	1.70	74	6.48	1.60	followup	4.00	0.23	[-0.07; 0.54]
Kissane, DW, et al., 2023	55	27.40	7.70	52	25.40	7.80	followup	24.00	0.26	[-0.12; 0.64]
McCaughan, E, et al., 2018	13	85.76	17.07	4	80.22	22.75	followup	4.00	0.29	[-0.84; 1.41]
Xia, S, et al., 2023	80	72.70	15.80	80	68.00	13.50	followup	12.00	0.32	[0.01; 0.63]
Hoffman, CJ, et al., 2012	101	103.56	17.91	106	96.84	21.14	followup	8.00	0.34	[0.07; 0.62]
Kissane, DW, et al., 2023	55	27.90	8.10	52	24.80	8.90	followup	12.00	0.36	[-0.02; 0.74]
Xia, S, et al., 2023	80	78.00	14.30	80	72.90	13.40	followup	24.00	0.37	[0.05; 0.68]
Hoffman, CJ, et al., 2012	101	103.78	17.85	106	96.22	19.43	followup	12.00	0.40	[0.13; 0.68]
Breitbart, W, et al. 2018 I.	94	7.06	1.50	74	6.44	1.50	followup	16.00	0.41	[0.10; 0.72]
Zhang, J, et al., 2021	61	9.67	3.71	61	8.21	3.02	followup	12.00	0.43	[0.07; 0.79]
Breitbart, W, et al. 2018 I.	94	7.33	1.70	74	6.49	1.50	followup	8.00	0.52	[0.21; 0.83]
Mihuta, ME, et al., 2018	32	14.10	2.60	33	12.30	3.60	followup	4.00	0.56	[0.07; 1.06]
Gaston-Johansson, F, et al., 2011	38	24.70	4.30	35	21.90	5.00	followup	48.00	0.60	[0.13; 1.07]
Cengiz, HO, et al., 2023	32	66.98	17.72	33	53.03	18.65	followup	8.00	0.76	[0.25; 1.26]
Chu, X. et al, 2020	42	85.41	16.08	42	71.90	18.50	followup	12.00	0.77	[0.33; 1.22]
Chu, X. et al, 2020	42	84.94	14.59	42	71.59	18.73	followup	8.00	0.79	[0.34; 1.23]
Rodin, G, et al., 2019	22	110.14	6.83	20	103.57	7.71	followup	4.00	0.89	[0.25; 1.53]
Rodin, G, et al., 2019	22	110.41	7.29	20	103.70	7.00	followup	8.00	0.92	[0.28; 1.56]
Jelvehzadeh, F, et al., 2022	24	6.05	1.00	24	4.65	0.80	followup	12.00	1.52	[0.87; 2.17]
Beatty, L. et al. 2015	30	68.50	3.64	30	62.85	3.64	followup	13.03	1.53	[0.95; 2.11]
Jelvehzadeh, F, et al., 2022	24	6.16	1.09	24	4.58	0.92	followup	8.00	1.54	[0.89; 2.19]
Rodin, G, et al., 2019	22	114.37	6.97	20	100.61	7.50	followup	12.00	1.87	[1.13; 2.60]
Cheung YL, et al. 2002	29	7.48	1.12	30	5.56	0.50	followup	10.00	2.20	[1.54; 2.85]
Fann, JR, et al., 2009	112	6.30	0.25	103	5.74	0.25	followup	24.00	2.23	[1.89; 2.57]
Beatty, L. et al. 2015	30	74.04	3.78	30	65.27	3.81	followup	26.07	2.28	[1.62; 2.94]
Fann, JR, et al., 2009	112	6.51	0.25	103	5.84	0.29	followup	96.00	2.47	[2.12; 2.83]
Fann, JR, et al., 2009	112	6.33	0.22	103	5.72	0.26	followup	12.00	2.53	[2.17; 2.89]
Cheung YL, et al. 2002	29	7.51	0.98	30	4.96	0.66	followup	5.00	3.02	[2.26; 3.78]
Fann, JR, et al., 2009	112	6.67	0.23	103	5.95	0.24	followup	48.00	3.06	[2.66; 3.45]
Fann, JR, et al., 2009	112	6.33	0.25	103	5.35	0.24	followup	72.00	3.98	[3.52; 4.45]
Random effect	4154			3971					0.63	[0.16; 1.10]
Prediction interval										[-0.71; 1.97]
nurse										
Li, X, et al., 2017	102	56.32	7.80	108	62.38	11.46	baseline	0.00	-0.61	[-0.89; -0.34]
Zhao,X, et al. 2015	62	7.85	2.52	62	8.75	2.38	baseline	0.00	-0.36	[-0.72; -0.01]
Walczak, A, et al.,2017	61	71.84	16.33	49	75.99	15.72	baseline	0.00	-0.26	[-0.63; 0.12]
Dirksen, S. et al, 2007	34	83.30	11.90	38	84.80	9.20	baseline	0.00	-0.14	[-0.60; 0.32]
Zhao, X, et al., 2021	52	53.70	3.14	51	54.69	9.61	baseline	0.00	-0.14	[-0.52; 0.25]
Gao Q, et al. 2020	40	-68.20	8.25	40	-68.44	8.82	baseline	0.00	0.03	[-0.41; 0.47]
Thomas, ML, et al., 2012	64	68.80	15.90	88	63.60	15.60	baseline	0.00	0.33	[0.00; 0.65]
van der Meulen, IC, et al., 2013	88	67.30	2.30	91	66.20	2.30	baseline	0.00	0.48	[0.18; 0.77]
Northouse, LL, et al., 2015	17	81.30	3.88	19	79.63	2.96	baseline	0.00	0.48	[-0.19; 1.14]
Northhouse, LL, et al., 2007	107	87.00	10.80	121	86.90	10.60	followup	32.00	0.01	[-0.25; 0.27]
Northhouse, LL, et al., 2007	104	86.10	10.90	114	85.80	10.70	followup	48.00	0.03	[-0.24; 0.29]
Northhouse, LL, et al., 2007	112	87.20	10.60	123	85.50	10.30	followup	16.00	0.16	[-0.09; 0.42]
Dirksen, S. et al, 2007	34	91.60	15.00	38	87.70	14.70	followup	10.00	0.26	[-0.20; 0.72]
Thomas, ML, et al., 2012	64	70.50	17.30	88	64.40	16.30	followup	12.00	0.36	[0.04; 0.69]
Walczak, A, et al.,2017	61	77.76	18.80	49	70.87	16.33	followup	4.00	0.39	[0.01; 0.77]
Gao Q, et al. 2020	40	-54.26	7.03	40	-60.17	7.28	followup	4.00	0.82	[0.36; 1.27]
van der Meulen, IC, et al., 2013	88	77.50	2.80	91	74.50	2.60	followup	48.00	1.11	[0.79; 1.42]
Li, X, et al., 2017	102	76.58	9.06	108	64.28	12.49	followup	12.00	1.12	[0.83; 1.41]
Zhao,X, et al. 2015	62	10.65	2.02	62	8.22	2.07	followup	0.30	1.18	[0.80; 1.56]
Zhao, X, et al., 2021	52	66.68	3.21	51	55.07	6.32	followup	12.00	2.31	[1.80; 2.81]
Northouse, LL, et al., 2015	17	88.51	3.03	19	76.94	3.44	followup	2.00	3.48	[2.41; 4.55]
Random effect	1363			1450					0.60	[0.12; 1.08]
Prediction interval										[-0.82; 2.02]
**Random effect**	**10637**			**10790**					**0.46**	**[0.11; 0.81]**
**Prediction interval**										**[-0.89; 1.81]**

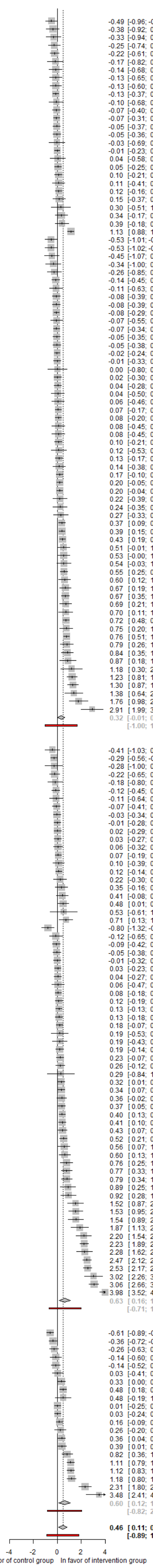

Figure S3.3. Subgroup analysis of the Global QoL. Forest plot represents the difference between the intervention vs. control group in the Global QoL domain with the provider subgroups as predicted at week 24 (post-intervention). SMD - Standardized mean difference, CI - confidence interval

Figure S3.4.T48

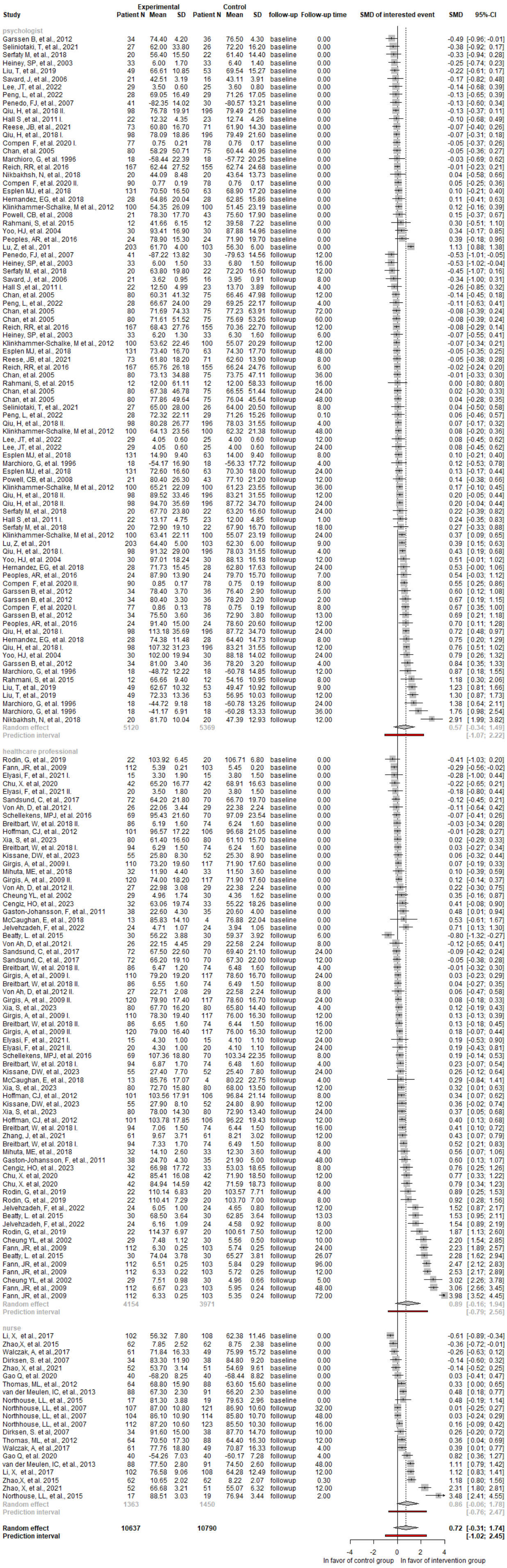

Study	Experimental Patient N	Experimental Mean	Experimental SD	Control Patient N	Control Mean	Control SD	follow-up	Follow-up time	SMD	95%-CI
**psychologist**										
Garssen B, et al., 2012	34	74.40	4.20	36	76.50	4.30	baseline	0.00	-0.49	[-0.96; -0.01]
Seliniotaki, T, et al., 2021	27	62.00	33.80	26	72.20	16.20	baseline	0.00	-0.38	[-0.92; 0.17]
Serfaty M, et al., 2018	20	56.40	15.50	22	61.40	14.40	baseline	0.00	-0.33	[-0.94; 0.28]
Heiney, SP, et al., 2003	33	6.00	1.70	33	6.40	1.40	baseline	0.00	-0.25	[-0.74; 0.23]
Liu, T, et al., 2019	49	66.61	10.85	53	69.54	15.27	baseline	0.00	-0.22	[-0.61; 0.17]
Savard, J, et al., 2006	21	42.51	3.19	16	43.11	3.91	baseline	0.00	-0.17	[-0.82; 0.48]
Lee, JT, et al., 2022	29	3.50	0.60	25	3.60	0.80	baseline	0.00	-0.14	[-0.68; 0.39]
Peng, L, et al., 2022	28	69.05	16.49	29	71.26	17.05	baseline	0.00	-0.13	[-0.65; 0.39]
Penedo, FJ, et al., 2007	41	-82.35	14.02	30	-80.57	13.21	baseline	0.00	-0.13	[-0.60; 0.34]
Qiu, H, et al., 2018 II.	98	76.78	19.91	196	79.49	21.60	baseline	0.00	-0.13	[-0.37; 0.11]
Hall S ,et al., 2011 I.	22	12.32	4.35	23	12.74	4.26	baseline	0.00	-0.10	[-0.68; 0.49]
Reese, JB, et al., 2021	73	60.80	16.70	71	61.90	14.30	baseline	0.00	-0.07	[-0.40; 0.26]
Qiu, H, et al., 2018 I.	98	78.09	18.86	196	79.49	21.60	baseline	0.00	-0.07	[-0.31; 0.18]
Compen F, et al. 2020 I.	77	0.75	0.21	78	0.76	0.17	baseline	0.00	-0.05	[-0.37; 0.26]
Chan, et al. 2005	80	58.29	50.71	75	60.44	40.96	baseline	0.00	-0.05	[-0.36; 0.27]
Marchioro, G, et al. 1996	18	-58.44	22.39	18	-57.72	20.25	baseline	0.00	-0.03	[-0.69; 0.62]
Reich, RR, et al. 2016	167	62.44	27.52	155	62.74	24.68	baseline	0.00	-0.01	[-0.23; 0.21]
Nikbakhsh, N, et al., 2018	20	44.09	8.48	20	43.64	13.73	baseline	0.00	0.04	[-0.58; 0.66]
Compen F, et al. 2020 II.	90	0.77	0.19	78	0.76	0.17	baseline	0.00	0.05	[-0.25; 0.36]
Esplen MJ, et al., 2018	131	70.50	16.50	63	68.90	17.20	baseline	0.00	0.10	[-0.21; 0.40]
Hernandez, EG, et al. 2018	28	64.86	20.04	28	62.85	15.86	baseline	0.00	0.11	[-0.41; 0.63]
Klinkhammer-Schalke, M et al., 2012	100	54.35	26.09	100	51.45	23.19	baseline	0.00	0.12	[-0.16; 0.39]
Powell, CB, et al., 2008	21	78.30	17.70	43	75.60	17.90	baseline	0.00	0.15	[-0.37; 0.67]
Rahmani, S, et al. 2015	12	41.66	6.15	12	39.58	7.22	baseline	0.00	0.30	[-0.51; 1.10]
Yoo, HJ, et al. 2004	30	93.41	16.90	30	87.88	14.96	baseline	0.00	0.34	[-0.17; 0.85]
Peoples, AR, et al., 2016	24	78.90	15.30	24	71.90	19.70	baseline	0.00	0.39	[-0.18; 0.96]
Lu, Z, et al., 201	203	61.70	4.00	103	56.30	6.00	baseline	0.00	1.13	[0.88; 1.38]
Penedo, FJ, et al., 2007	41	-87.22	13.82	30	-79.63	14.56	followup	12.00	-0.53	[-1.01; -0.05]
Heiney, SP, et al., 2003	33	6.00	1.50	33	6.80	1.50	followup	16.00	-0.53	[-1.02; -0.04]
Serfaty M, et al., 2018	20	63.80	19.80	22	72.20	16.60	followup	12.00	-0.45	[-1.07; 0.16]
Savard, J, et al., 2006	21	3.62	0.95	16	3.95	0.91	followup	8.00	-0.34	[-1.00; 0.31]
Hall S ,et al., 2011 I.	22	12.50	4.99	23	13.70	3.89	followup	4.00	-0.26	[-0.85; 0.32]
Chan, et al. 2005	80	60.31	41.32	75	66.46	47.98	followup	12.00	-0.14	[-0.45; 0.18]
Peng, L, et al., 2022	28	66.67	24.00	29	69.25	22.17	followup	4.00	-0.11	[-0.63; 0.41]
Chan, et al. 2005	80	71.69	74.33	75	77.23	63.91	followup	72.00	-0.08	[-0.39; 0.24]
Chan, et al. 2005	80	71.61	51.52	75	75.69	53.26	followup	60.00	-0.08	[-0.39; 0.24]
Reich, RR, et al. 2016	167	68.43	27.76	155	70.36	22.70	followup	12.00	-0.08	[-0.29; 0.14]
Heiney, SP, et al., 2003	33	6.20	1.30	33	6.30	1.60	followup	6.00	-0.07	[-0.55; 0.41]
Klinkhammer-Schalke, M et al., 2012	100	53.62	22.46	100	55.07	20.29	followup	12.00	-0.07	[-0.34; 0.21]
Esplen MJ, et al., 2018	131	73.40	16.70	63	74.30	17.70	followup	48.00	-0.05	[-0.35; 0.25]
Reese, JB, et al., 2021	73	61.80	18.20	71	62.60	13.90	followup	8.00	-0.05	[-0.38; 0.28]
Reich, RR, et al. 2016	167	65.76	26.18	155	66.24	24.76	followup	6.00	-0.02	[-0.24; 0.20]
Chan, et al. 2005	80	73.13	34.88	75	73.75	47.11	followup	36.00	-0.01	[-0.33; 0.30]
Rahmani, S, et al. 2015	12	12.00	61.11	12	12.00	58.33	followup	16.00	0.00	[-0.80; 0.80]
Chan, et al. 2005	80	67.38	46.78	75	66.55	51.44	followup	24.00	0.02	[-0.30; 0.33]
Chan, et al. 2005	80	77.86	49.64	75	76.04	45.64	followup	48.00	0.04	[-0.28; 0.35]
Seliniotaki, T, et al., 2021	27	65.00	28.00	26	64.00	20.50	followup	8.00	0.04	[-0.50; 0.58]
Peng, L, et al., 2022	28	72.32	22.11	29	71.26	15.26	followup	0.10	0.06	[-0.46; 0.57]
Qiu, H, et al., 2018 II.	98	80.28	26.77	196	78.03	31.55	followup	4.00	0.07	[-0.17; 0.32]
Klinkhammer-Schalke, M et al., 2012	100	64.13	23.56	100	62.32	21.38	followup	48.00	0.08	[-0.20; 0.36]
Lee, JT, et al., 2022	29	4.05	0.60	25	4.00	0.60	followup	12.00	0.08	[-0.45; 0.62]
Lee, JT, et al., 2022	29	4.05	0.60	25	4.00	0.60	followup	24.00	0.08	[-0.45; 0.62]
Esplen MJ, et al., 2018	131	14.90	9.40	63	14.00	9.40	followup	8.00	0.10	[-0.21; 0.40]
Marchioro, G, et al. 1996	18	-54.17	16.90	18	-56.33	17.72	followup	4.00	0.12	[-0.53; 0.78]
Esplen MJ, et al., 2018	131	72.60	16.60	63	70.30	18.00	followup	24.00	0.13	[-0.17; 0.44]
Powell, CB, et al., 2008	21	80.40	26.30	43	77.10	21.20	followup	12.00	0.14	[-0.38; 0.66]
Klinkhammer-Schalke, M et al., 2012	100	65.21	22.09	100	61.23	23.55	followup	36.00	0.17	[-0.10; 0.45]
Qiu, H, et al., 2018 II.	98	89.52	33.46	196	83.21	31.55	followup	12.00	0.20	[-0.05; 0.44]
Qiu, H, et al., 2018 II.	98	94.70	35.69	196	87.72	34.70	followup	24.00	0.20	[-0.04; 0.44]
Serfaty M, et al., 2018	20	67.70	23.80	22	63.20	16.60	followup	24.00	0.22	[-0.39; 0.82]
Hall S ,et al., 2011 I.	22	13.17	4.75	23	12.00	4.85	followup	1.00	0.24	[-0.35; 0.83]
Serfaty M, et al., 2018	20	72.90	19.10	22	67.90	16.70	followup	18.00	0.27	[-0.33; 0.88]
Klinkhammer-Schalke, M et al., 2012	100	63.41	22.11	100	55.07	23.19	followup	24.00	0.37	[0.09; 0.65]
Lu, Z, et al., 201	203	64.40	5.00	103	62.30	6.00	followup	9.00	0.39	[0.15; 0.63]
Qiu, H, et al., 2018 I.	98	91.32	29.00	196	78.03	31.55	followup	4.00	0.43	[0.19; 0.68]
Yoo, HJ, et al. 2004	30	97.01	18.24	30	88.13	16.18	followup	12.00	0.51	[-0.01; 1.02]
Hernandez, EG, et al. 2018	28	71.73	15.45	28	62.80	17.63	followup	24.00	0.53	[-0.00; 1.06]
Peoples, AR, et al., 2016	24	87.90	13.90	24	79.70	15.70	followup	7.00	0.54	[-0.03; 1.12]
Compen F, et al. 2020 II.	90	0.85	0.17	78	0.75	0.19	followup	8.00	0.55	[0.25; 0.86]
Garssen B, et al., 2012	34	78.40	3.70	36	76.40	2.90	followup	5.00	0.60	[0.12; 1.08]
Garssen B, et al., 2012	34	80.40	3.30	36	78.20	3.20	followup	2.00	0.67	[0.19; 1.15]
Compen F, et al. 2020 I.	77	0.86	0.13	78	0.75	0.19	followup	8.00	0.67	[0.35; 1.00]
Garssen B, et al., 2012	34	75.50	3.60	36	72.90	3.80	followup	13.00	0.69	[0.21; 1.18]
Peoples, AR, et al., 2016	24	91.40	15.00	24	78.60	20.60	followup	12.00	0.70	[0.11; 1.28]
Qiu, H, et al., 2018 I.	98	113.18	35.69	196	87.72	34.70	followup	24.00	0.72	[0.48; 0.97]
Hernandez, EG, et al. 2018	28	74.38	11.48	28	64.40	14.73	followup	8.00	0.75	[0.20; 1.29]
Qiu, H, et al., 2018 I.	98	107.32	31.23	196	83.21	31.55	followup	12.00	0.76	[0.51; 1.02]
Yoo, HJ, et al. 2004	30	102.00	19.94	30	88.18	14.02	followup	24.00	0.79	[0.26; 1.32]
Garssen B, et al., 2012	34	81.00	3.40	36	78.20	3.20	followup	4.00	0.84	[0.35; 1.33]
Marchioro, G, et al. 1996	18	-48.72	12.22	18	-60.78	14.85	followup	12.00	0.87	[0.18; 1.55]
Rahmani, S, et al. 2015	12	66.66	9.40	12	54.16	10.95	followup	8.00	1.18	[0.30; 2.06]
Liu, T, et al., 2019	49	62.67	10.32	53	49.47	10.92	followup	9.00	1.23	[0.81; 1.66]
Liu, T, et al., 2019	49	72.33	13.36	53	56.95	10.03	followup	12.00	1.30	[0.87; 1.73]
Marchioro, G, et al. 1996	18	-44.72	9.18	18	-60.78	13.26	followup	24.00	1.38	[0.64; 2.11]
Marchioro, G, et al. 1996	18	-41.17	6.91	18	-60.28	13.33	followup	36.00	1.76	[0.98; 2.54]
Nikbakhsh, N, et al., 2018	20	81.70	10.04	20	47.39	12.93	followup	12.00	2.91	[1.99; 3.82]
Random effect	5120			5369					0.57	[-0.34; 1.49]
Prediction interval										[-1.07; 2.22]
**healthcare professional**										
Rodin, G, et al., 2019	22	103.92	6.45	20	106.71	6.80	baseline	0.00	-0.41	[-1.03; 0.20]
Fann, JR, et al., 2009	112	5.39	0.21	103	5.45	0.20	baseline	0.00	-0.29	[-0.56; -0.02]
Elyasi, F, et al., 2021 I.	15	3.30	1.90	15	3.80	1.50	baseline	0.00	-0.28	[-1.00; 0.44]
Chu, X. et al, 2020	42	65.20	16.77	42	68.91	16.63	baseline	0.00	-0.22	[-0.65; 0.21]
Elyasi, F, et al., 2021 II.	20	3.50	1.80	20	3.80	1.50	baseline	0.00	-0.18	[-0.80; 0.44]
Sandsund, C, et al., 2017	72	64.20	21.80	70	66.70	19.70	baseline	0.00	-0.12	[-0.45; 0.21]
Von Ah, D, et al.,2012 I.	26	22.06	3.44	29	22.38	2.24	baseline	0.00	-0.11	[-0.64; 0.42]
Schellekens, MPJ, et al. 2016	69	95.43	21.60	70	97.09	23.54	baseline	0.00	-0.07	[-0.41; 0.26]
Breitbart, W, et al. 2018 II.	86	6.19	1.60	74	6.24	1.60	baseline	0.00	-0.03	[-0.34; 0.28]
Hoffman, CJ, et al., 2012	101	96.57	17.22	106	96.68	21.05	baseline	0.00	-0.01	[-0.28; 0.27]
Xia, S, et al., 2023	80	61.40	16.60	80	61.10	15.70	baseline	0.00	0.02	[-0.29; 0.33]
Breitbart, W, et al. 2018 I.	94	6.29	1.50	74	6.24	1.60	baseline	0.00	0.03	[-0.27; 0.34]
Kissane, DW, et al., 2023	55	25.80	8.30	52	25.30	8.90	baseline	0.00	0.06	[-0.32; 0.44]
Girgis, A, et al., 2009 I.	110	73.20	19.60	117	71.90	17.60	baseline	0.00	0.07	[-0.19; 0.33]
Mihuta, ME, et al., 2018	32	11.90	4.40	33	11.50	3.60	baseline	0.00	0.10	[-0.39; 0.59]
Girgis, A, et al., 2009 II.	120	74.00	18.20	117	71.90	17.60	baseline	0.00	0.12	[-0.14; 0.37]
Von Ah, D, et al.,2012 II.	27	22.98	3.08	29	22.38	2.24	baseline	0.00	0.22	[-0.30; 0.75]
Cheung YL, et al. 2002	29	4.96	1.74	30	4.36	1.62	baseline	0.00	0.35	[-0.16; 0.87]
Cengiz, HO, et al., 2023	32	63.06	19.74	33	55.22	18.26	baseline	0.00	0.41	[-0.08; 0.90]
Gaston-Johansson, F, et al., 2011	38	22.60	4.30	35	20.60	4.00	baseline	0.00	0.48	[0.01; 0.94]
McCaughan, E, et al., 2018	13	85.83	14.10	4	76.88	22.04	baseline	0.00	0.53	[-0.61; 1.67]
Jelvehzadeh, F, et al., 2022	24	4.71	1.07	24	3.94	1.06	baseline	0.00	0.71	[0.13; 1.30]
Beatty, L. et al. 2015	30	56.22	3.88	30	59.37	3.92	followup	6.00	-0.80	[-1.32; -0.27]
Von Ah, D, et al.,2012 I.	26	22.15	4.45	29	22.58	2.24	followup	8.00	-0.12	[-0.65; 0.41]
Sandsund, C, et al., 2017	72	67.50	22.60	70	69.40	21.10	followup	24.00	-0.09	[-0.42; 0.24]
Sandsund, C, et al., 2017	72	66.20	19.10	70	67.30	22.00	followup	12.00	-0.05	[-0.38; 0.28]
Breitbart, W, et al. 2018 II.	86	6.47	1.20	74	6.48	1.60	followup	4.00	-0.01	[-0.32; 0.30]
Girgis, A, et al., 2009 I.	110	79.20	19.20	117	78.60	16.70	followup	24.00	0.03	[-0.23; 0.29]
Breitbart, W, et al. 2018 II.	86	6.55	1.60	74	6.49	1.50	followup	8.00	0.04	[-0.27; 0.35]
Von Ah, D, et al.,2012 II.	27	22.71	2.08	29	22.58	2.24	followup	8.00	0.06	[-0.47; 0.58]
Girgis, A, et al., 2009 II.	120	79.90	17.40	117	78.60	16.70	followup	24.00	0.08	[-0.18; 0.33]
Xia, S, et al., 2023	80	67.70	16.20	80	65.80	14.40	followup	4.00	0.12	[-0.19; 0.43]
Girgis, A, et al., 2009 I.	110	78.30	19.40	117	76.00	16.30	followup	12.00	0.13	[-0.13; 0.39]
Breitbart, W, et al. 2018 II.	86	6.65	1.60	74	6.44	1.50	followup	16.00	0.13	[-0.18; 0.45]
Girgis, A, et al., 2009 II.	120	79.00	16.40	117	76.00	16.30	followup	12.00	0.18	[-0.07; 0.44]
Elyasi, F, et al., 2021 I.	15	4.30	1.00	15	4.10	1.10	followup	24.00	0.19	[-0.53; 0.90]
Elyasi, F, et al., 2021 II.	20	4.30	1.00	20	4.10	1.10	followup	24.00	0.19	[-0.43; 0.81]
Schellekens, MPJ, et al. 2016	69	107.36	18.80	70	103.34	22.35	followup	8.00	0.19	[-0.14; 0.53]
Breitbart, W, et al. 2018 I.	94	6.87	1.70	74	6.48	1.60	followup	4.00	0.23	[-0.07; 0.54]
Kissane, DW, et al., 2023	55	27.40	7.70	52	25.40	7.80	followup	24.00	0.26	[-0.12; 0.64]
McCaughan, E, et al., 2018	13	85.76	17.07	4	80.22	22.75	followup	4.00	0.29	[-0.84; 1.41]
Xia, S, et al., 2023	80	72.70	15.80	80	68.00	13.50	followup	12.00	0.32	[0.01; 0.63]
Hoffman, CJ, et al., 2012	101	103.56	17.91	106	96.84	21.14	followup	8.00	0.34	[0.07; 0.62]
Kissane, DW, et al., 2023	55	27.90	8.10	52	24.80	8.90	followup	12.00	0.36	[-0.02; 0.74]
Xia, S, et al., 2023	80	78.00	14.30	80	72.90	13.40	followup	24.00	0.37	[0.05; 0.68]
Hoffman, CJ, et al., 2012	101	103.78	17.85	106	96.22	19.43	followup	12.00	0.40	[0.13; 0.68]
Breitbart, W, et al. 2018 I.	94	7.06	1.50	74	6.44	1.50	followup	16.00	0.41	[0.10; 0.72]
Zhang, J, et al., 2021	61	9.67	3.71	61	8.21	3.02	followup	12.00	0.43	[0.07; 0.79]
Breitbart, W, et al. 2018 I.	94	7.33	1.70	74	6.49	1.50	followup	8.00	0.52	[0.21; 0.83]
Mihuta, ME, et al., 2018	32	14.10	2.60	33	12.30	3.60	followup	4.00	0.56	[0.07; 1.06]
Gaston-Johansson, F, et al., 2011	38	24.70	4.30	35	21.90	5.00	followup	48.00	0.60	[0.13; 1.07]
Cengiz, HO, et al., 2023	32	66.98	17.72	33	53.03	18.65	followup	8.00	0.76	[0.25; 1.26]
Chu, X. et al, 2020	42	85.41	16.08	42	71.90	18.50	followup	12.00	0.77	[0.33; 1.22]
Chu, X. et al, 2020	42	84.94	14.59	42	71.59	18.73	followup	8.00	0.79	[0.34; 1.23]
Rodin, G, et al., 2019	22	110.14	6.83	20	103.57	7.71	followup	4.00	0.89	[0.25; 1.53]
Rodin, G, et al., 2019	22	110.41	7.29	20	103.70	7.00	followup	8.00	0.92	[0.28; 1.56]
Jelvehzadeh, F, et al., 2022	24	6.05	1.00	24	4.65	0.80	followup	12.00	1.52	[0.87; 2.17]
Beatty, L. et al. 2015	30	68.50	3.64	30	62.85	3.64	followup	13.03	1.53	[0.95; 2.11]
Jelvehzadeh, F, et al., 2022	24	6.16	1.09	24	4.58	0.92	followup	8.00	1.54	[0.89; 2.19]
Rodin, G, et al., 2019	22	114.37	6.97	20	100.61	7.50	followup	12.00	1.87	[1.13; 2.60]
Cheung YL, et al. 2002	29	7.48	1.12	30	5.56	0.50	followup	10.00	2.20	[1.54; 2.85]
Fann, JR, et al., 2009	112	6.30	0.25	103	5.74	0.25	followup	24.00	2.23	[1.89; 2.57]
Beatty, L. et al. 2015	30	74.04	3.78	30	65.27	3.81	followup	26.07	2.28	[1.62; 2.94]
Fann, JR, et al., 2009	112	6.51	0.25	103	5.84	0.29	followup	96.00	2.47	[2.12; 2.83]
Fann, JR, et al., 2009	112	6.33	0.22	103	5.72	0.26	followup	12.00	2.53	[2.17; 2.89]
Cheung YL, et al. 2002	29	7.51	0.98	30	4.96	0.66	followup	5.00	3.02	[2.26; 3.78]
Fann, JR, et al., 2009	112	6.67	0.23	103	5.95	0.24	followup	48.00	3.06	[2.66; 3.45]
Fann, JR, et al., 2009	112	6.33	0.25	103	5.35	0.24	followup	72.00	3.98	[3.52; 4.45]
Random effect	4154			3971					0.89	[-0.16; 1.94]
Prediction interval										[-0.79; 2.56]
**nurse**										
Li, X, et al., 2017	102	56.32	7.80	108	62.38	11.46	baseline	0.00	-0.61	[-0.89; -0.34]
Zhao,X, et al. 2015	62	7.85	2.52	62	8.75	2.38	baseline	0.00	-0.36	[-0.72; -0.01]
Walczak, A, et al.,2017	61	71.84	16.33	49	75.99	15.72	baseline	0.00	-0.26	[-0.63; 0.12]
Dirksen, S. et al, 2007	34	83.30	11.90	38	84.80	9.20	baseline	0.00	-0.14	[-0.60; 0.32]
Zhao, X, et al., 2021	52	53.70	3.14	51	54.69	9.61	baseline	0.00	-0.14	[-0.52; 0.25]
Gao Q, et al. 2020	40	-68.20	8.25	40	-68.44	8.82	baseline	0.00	0.03	[-0.41; 0.47]
Thomas, ML, et al., 2012	64	68.80	15.90	88	63.60	15.60	baseline	0.00	0.33	[0.00; 0.65]
van der Meulen, IC, et al., 2013	88	67.30	2.30	91	66.20	2.30	baseline	0.00	0.48	[0.18; 0.77]
Northouse, LL, et al., 2015	17	81.30	3.88	19	79.63	2.96	baseline	0.00	0.48	[-0.19; 1.14]
Northhouse, LL, et al., 2007	107	87.00	10.80	121	86.90	10.60	followup	32.00	0.01	[-0.25; 0.27]
Northhouse, LL, et al., 2007	104	86.10	10.90	114	85.80	10.70	followup	48.00	0.03	[-0.24; 0.29]
Northhouse, LL, et al., 2007	112	87.20	10.60	123	85.50	10.30	followup	16.00	0.16	[-0.09; 0.42]
Dirksen, S. et al, 2007	34	91.60	15.00	38	87.70	14.70	followup	10.00	0.26	[-0.20; 0.72]
Thomas, ML, et al., 2012	64	70.50	17.30	88	64.40	16.30	followup	12.00	0.36	[0.04; 0.69]
Walczak, A, et al.,2017	61	77.76	18.80	49	70.87	16.33	followup	4.00	0.39	[0.01; 0.77]
Gao Q, et al. 2020	40	-54.26	7.03	40	-60.17	7.28	followup	4.00	0.82	[0.36; 1.27]
van der Meulen, IC, et al., 2013	88	77.50	2.80	91	74.50	2.60	followup	48.00	1.11	[0.79; 1.42]
Li, X, et al., 2017	102	76.58	9.06	108	64.28	12.49	followup	12.00	1.12	[0.83; 1.41]
Zhao,X, et al. 2015	62	10.65	2.02	62	8.22	2.07	followup	0.30	1.18	[0.80; 1.56]
Zhao, X, et al., 2021	52	66.68	3.21	51	55.07	6.32	followup	12.00	2.31	[1.80; 2.81]
Northouse, LL, et al., 2015	17	88.51	3.03	19	76.94	3.44	followup	2.00	3.48	[2.41; 4.55]
Random effect	1363			1450					0.86	[-0.06; 1.78]
Prediction interval										[-0.76; 2.47]
**Random effect**	**10637**			**10790**					**0.72**	**[-0.31; 1.74]**
**Prediction interval**										**[-1.02; 2.45]**

Figure S3.4. Subgroup analysis of the Global QoL. Forest plot represents the difference between the intervention vs. control group in the Global QoL domain with the provider subgroups as predicted at week 48 (post-intervention). SMD - Standardized mean difference, CI - confidence interval

S4.Subgroup analysis of Global QoL: Environment

Figure S4.1.T0

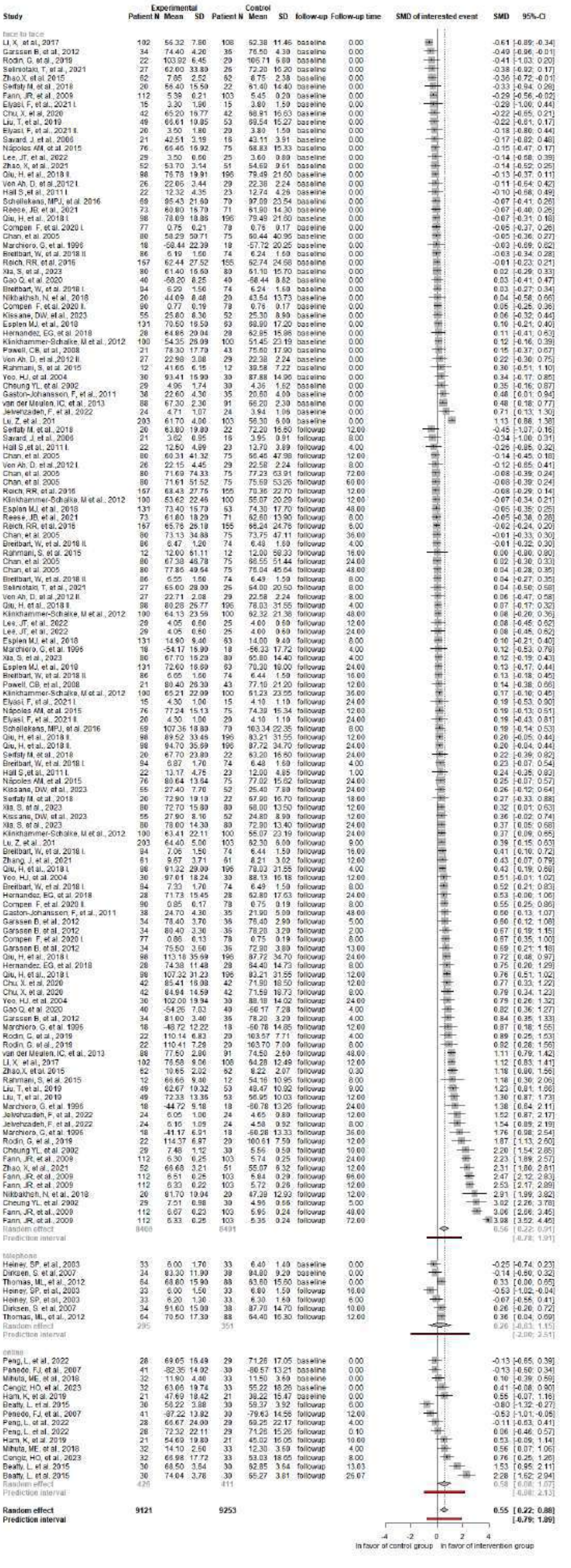

Figure S4.1.Subgroup analysis of the Global QoL Forest plot represents the difference between the intervention vs. control group in the Global QoL domain with the environment subgroups as predicted at week 0 (post-intervention). SMD - Standardized mean difference, CI - confidence interval.

Figure S4.2.T12

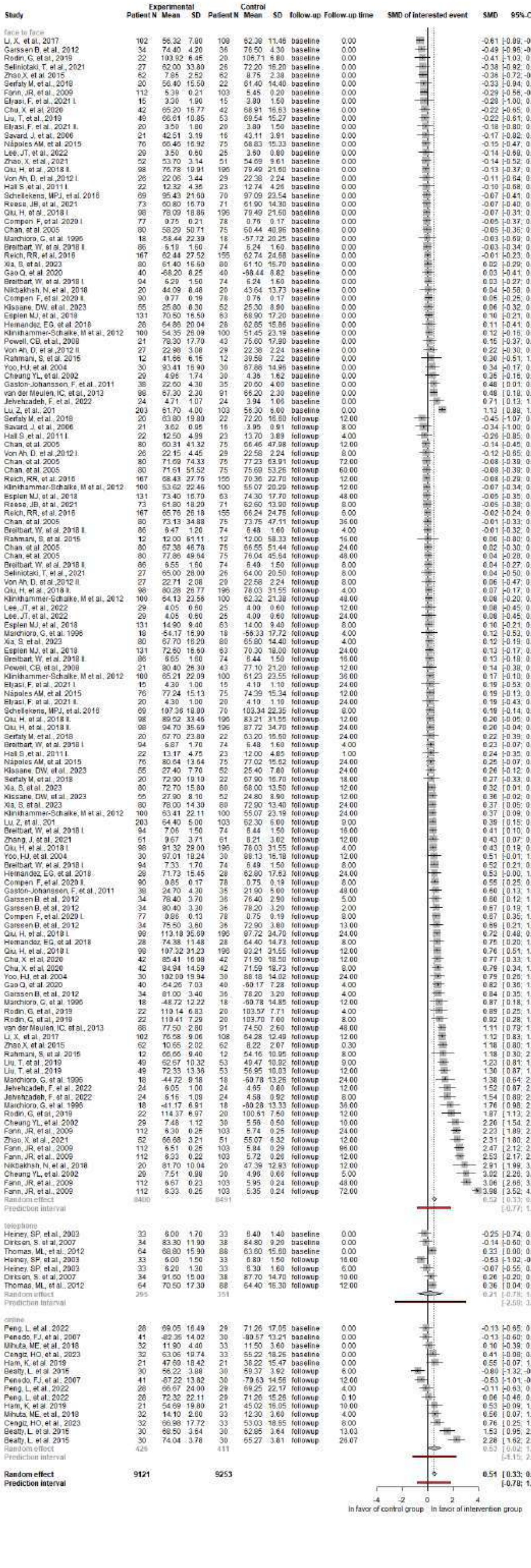

Figure S4.2. Subgroup analysis of the Global QoL. Forest plot represents the difference between the intervention vs. control group in the Global QoL domain with the environment subgroups as predicted at week 12 (post-intervention). SMD - Standardized mean difference, CI - confidence interval.

Figure S4.3.T24

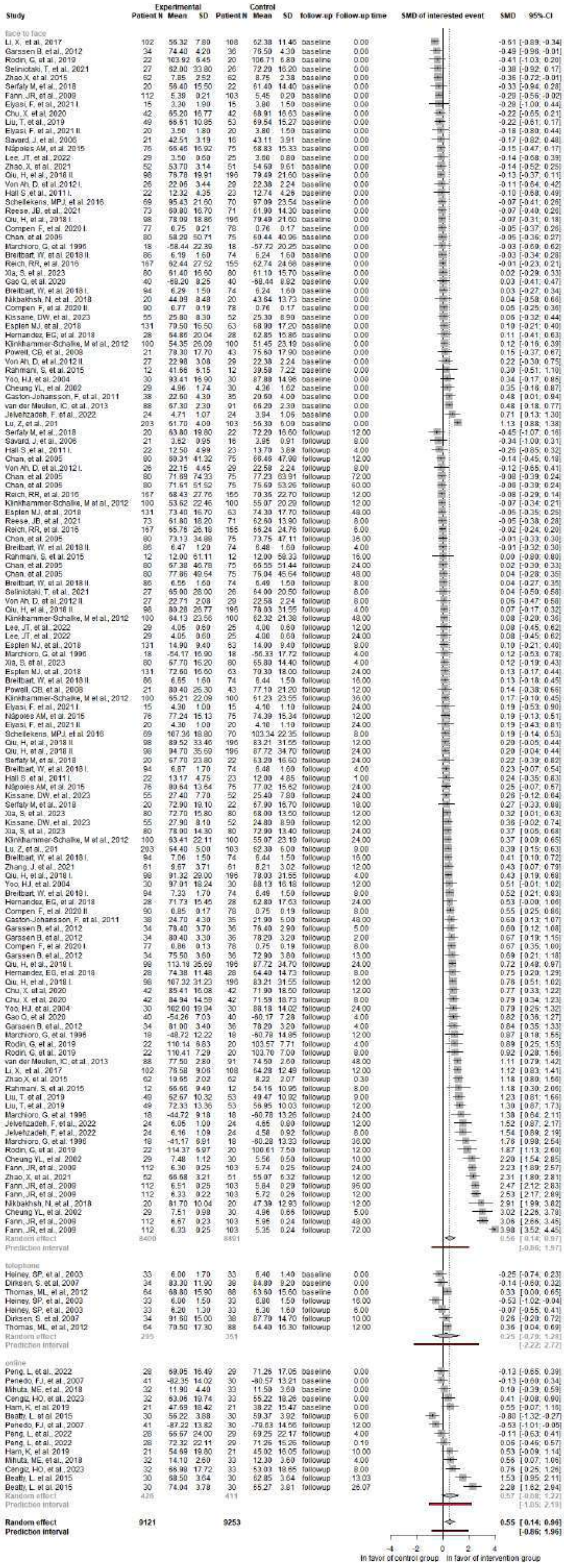

Figure S4.3. Subgroup analysis of the Global QoL. Forest plot represents the difference between the intervention vs. control group in the Global QoL domain with the environment subgroups as predicted at week 24 (post-intervention). SMD - Standardized mean difference, CI - confidence interval.

Figure S4.4.T48

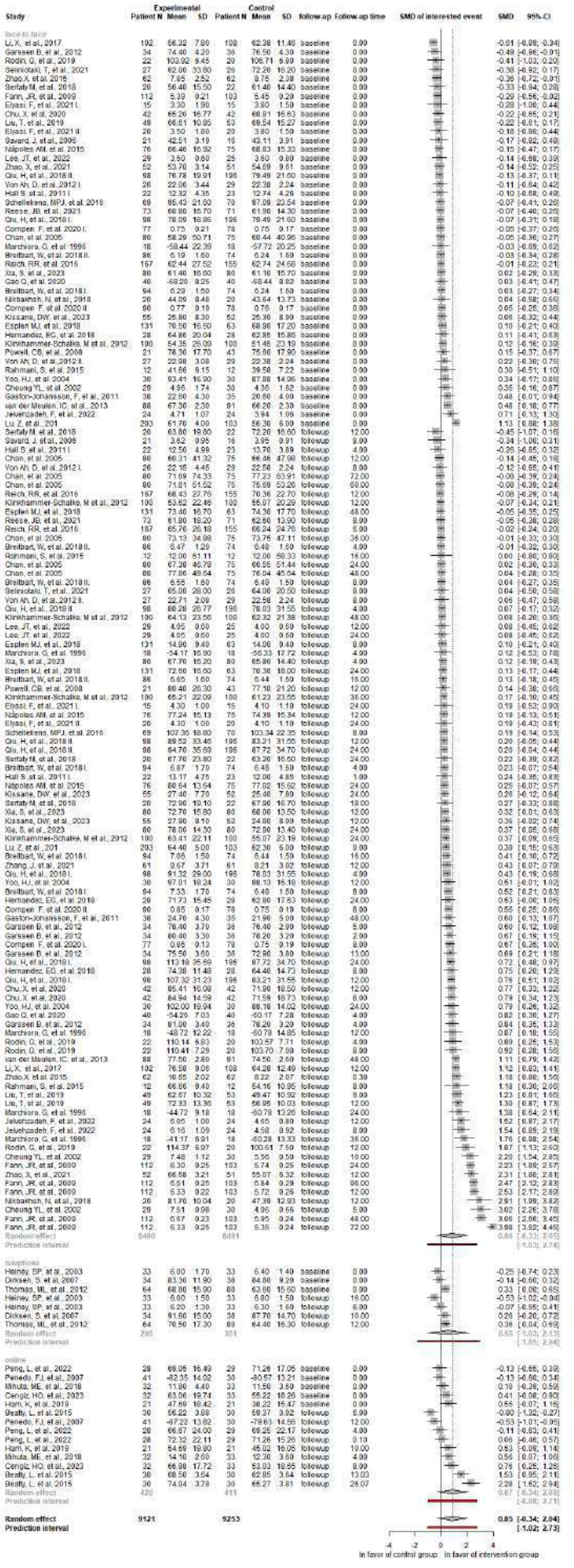

Figure S4.4.Subgroup analysis of the Global QoL Forest plot represents the difference between the intervention vs. control group in the Global QoL domain with the environment subgroups as predicted at week 48 (post-intervention). SMD - Standardized mean difference, CI - confidence interval.

S5.Subgroup analysis of Global QoL: Type

Figure S5.1.T0

Study	Experimental Patient N	Experimental Mean	Experimental SD	Control Patient N	Control Mean	Control SD	follow-up	Follow-up time	SMD	95%-CI
**individual**										
Li, X, et al., 2017	102	56.32	7.80	108	62.38	11.46	baseline	0.00	-0.61	[-0.89; -0.34]
Garssen B, et al., 2012	34	74.40	4.20	36	76.50	4.30	baseline	0.00	-0.49	[-0.96; -0.01]
Rodin, G, et al., 2019	22	103.92	6.45	20	106.71	6.80	baseline	0.00	-0.41	[-1.03; 0.20]
Seliniotaki, T, et al., 2021	27	62.00	33.80	26	72.20	16.20	baseline	0.00	-0.38	[-0.92; 0.17]
Zhao,X, et al. 2015	62	7.85	2.52	62	8.75	2.38	baseline	0.00	-0.36	[-0.72; -0.01]
Serfaty M, et al., 2018	20	56.40	15.50	22	61.40	14.40	baseline	0.00	-0.33	[-0.94; 0.28]
Fann, JR, et al., 2009	112	5.39	0.21	103	5.45	0.20	baseline	0.00	-0.29	[-0.56; -0.02]
Elyasi, F, et al., 2021 I.	15	3.30	1.90	15	3.80	1.50	baseline	0.00	-0.28	[-1.00; 0.44]
Walczak, A, et al.,2017	61	71.84	16.33	49	75.99	15.72	baseline	0.00	-0.26	[-0.63; 0.12]
Chu, X. et al, 2020	42	65.20	16.77	42	68.91	16.63	baseline	0.00	-0.22	[-0.65; 0.21]
Elyasi, F, et al., 2021 II.	20	3.50	1.80	20	3.80	1.50	baseline	0.00	-0.18	[-0.80; 0.44]
Savard, J, et al., 2006	21	42.51	3.19	16	43.11	3.91	baseline	0.00	-0.17	[-0.82; 0.48]
Nápoles AM, et al. 2015	76	66.46	16.92	75	68.83	15.33	baseline	0.00	-0.15	[-0.47; 0.17]
Dirksen, S. et al, 2007	34	83.30	11.90	38	84.80	9.20	baseline	0.00	-0.14	[-0.60; 0.32]
Zhao, X, et al., 2021	52	53.70	3.14	51	54.69	9.61	baseline	0.00	-0.14	[-0.52; 0.25]
Qiu, H, et al., 2018 II.	98	76.78	19.91	196	79.49	21.60	baseline	0.00	-0.13	[-0.37; 0.11]
Sandsund, C, et al., 2017	72	64.20	21.80	70	66.70	19.70	baseline	0.00	-0.12	[-0.45; 0.21]
Hall S ,et al., 2011 I.	22	12.32	4.35	23	12.74	4.26	baseline	0.00	-0.10	[-0.68; 0.49]
Reese, JB, et al., 2021	73	60.80	16.70	71	61.90	14.30	baseline	0.00	-0.07	[-0.40; 0.26]
Qiu, H, et al., 2018 I.	98	78.09	18.86	196	79.49	21.60	baseline	0.00	-0.07	[-0.31; 0.18]
Chan, et al. 2005	80	58.29	50.71	75	60.44	40.96	baseline	0.00	-0.05	[-0.36; 0.27]
Marchioro, G, et al. 1996	18	-58.44	22.39	18	-57.72	20.25	baseline	0.00	-0.03	[-0.69; 0.62]
Breitbart, W, et al. 2018 II.	86	6.19	1.60	74	6.24	1.60	baseline	0.00	-0.03	[-0.34; 0.28]
Gao Q, et al. 2020	40	-68.20	8.25	40	-68.44	8.82	baseline	0.00	0.03	[-0.41; 0.47]
Breitbart, W, et al. 2018 I.	94	6.29	1.50	74	6.24	1.60	baseline	0.00	0.03	[-0.27; 0.34]
Kissane, DW, et al., 2023	55	25.80	8.30	52	25.30	8.90	baseline	0.00	0.06	[-0.32; 0.44]
Girgis, A, et al., 2009 I.	110	73.20	19.60	117	71.90	17.60	baseline	0.00	0.07	[-0.19; 0.33]
Girgis, A, et al., 2009 II.	120	74.00	18.20	117	71.90	17.60	baseline	0.00	0.12	[-0.14; 0.37]
Klinkhammer-Schalke, M et al., 2012	100	54.35	26.09	100	51.45	23.19	baseline	0.00	0.12	[-0.16; 0.39]
Powell, CB, et al., 2008	21	78.30	17.70	43	75.60	17.90	baseline	0.00	0.15	[-0.37; 0.67]
Thomas, ML, et al., 2012	64	68.80	15.90	88	63.60	15.60	baseline	0.00	0.33	[0.00; 0.65]
Cheung YL, et al. 2002	29	4.96	1.74	30	4.36	1.62	baseline	0.00	0.35	[-0.16; 0.87]
Peoples, AR, et al., 2016	24	78.90	15.30	24	71.90	19.70	baseline	0.00	0.39	[-0.18; 0.96]
Gaston-Johansson, F, et al., 2011	38	22.60	4.30	35	20.60	4.00	baseline	0.00	0.48	[0.01; 0.94]
van der Meulen, IC, et al., 2013	88	67.30	2.30	91	66.20	2.30	baseline	0.00	0.48	[0.18; 0.77]
Northouse, LL, et al., 2015	17	81.30	3.88	19	79.63	2.96	baseline	0.00	0.48	[-0.19; 1.14]
Ham, K, et al. 2019	21	47.69	18.42	21	38.22	15.47	baseline	0.00	0.55	[-0.07; 1.16]
Lu, Z, et al., 201	203	61.70	4.00	103	56.30	6.00	baseline	0.00	1.13	[0.88; 1.38]
Beatty, L. et al. 2015	30	56.22	3.88	30	59.37	3.92	followup	6.00	-0.80	[-1.32; -0.27]
Serfaty M, et al., 2018	20	63.80	19.80	22	72.20	16.60	followup	12.00	-0.45	[-1.07; 0.16]
Savard, J, et al., 2006	21	3.62	0.95	16	3.95	0.91	followup	8.00	-0.34	[-1.00; 0.31]
Hall S ,et al., 2011 I.	22	12.50	4.99	23	13.70	3.89	followup	4.00	-0.26	[-0.85; 0.32]
Chan, et al. 2005	80	60.31	41.32	75	66.46	47.98	followup	12.00	-0.14	[-0.45; 0.18]
Sandsund, C, et al., 2017	72	67.50	22.60	70	69.40	21.10	followup	24.00	-0.09	[-0.42; 0.24]
Chan, et al. 2005	80	71.69	74.33	75	77.23	63.91	followup	72.00	-0.08	[-0.39; 0.24]
Chan, et al. 2005	80	71.61	51.52	75	75.69	53.26	followup	60.00	-0.08	[-0.39; 0.24]
Klinkhammer-Schalke, M et al., 2012	100	53.62	22.46	100	55.07	20.29	followup	12.00	-0.07	[-0.34; 0.21]
Sandsund, C, et al., 2017	72	66.20	19.10	70	67.30	22.00	followup	12.00	-0.05	[-0.38; 0.28]
Reese, JB, et al., 2021	73	61.80	18.20	71	62.60	13.90	followup	8.00	-0.05	[-0.38; 0.28]
Chan, et al. 2005	80	73.13	34.88	75	73.75	47.11	followup	36.00	-0.01	[-0.33; 0.30]
Breitbart, W, et al. 2018 II.	86	6.47	1.20	74	6.48	1.60	followup	4.00	-0.01	[-0.32; 0.30]
Northhouse, LL, et al., 2007	107	87.00	10.80	121	86.90	10.60	followup	32.00	0.01	[-0.25; 0.27]
Chan, et al. 2005	80	67.38	46.78	75	66.55	51.44	followup	24.00	0.02	[-0.30; 0.33]
Northhouse, LL, et al., 2007	104	86.10	10.90	114	85.80	10.70	followup	48.00	0.03	[-0.24; 0.29]
Girgis, A, et al., 2009 I.	110	79.20	19.20	117	78.60	16.70	followup	24.00	0.03	[-0.23; 0.29]
Chan, et al. 2005	80	77.86	49.64	75	76.04	45.64	followup	48.00	0.04	[-0.28; 0.35]
Breitbart, W, et al. 2018 II.	86	6.55	1.60	74	6.49	1.50	followup	8.00	0.04	[-0.27; 0.35]
Seliniotaki, T, et al., 2021	27	65.00	28.00	26	64.00	20.50	followup	8.00	0.04	[-0.50; 0.58]
Qiu, H, et al., 2018 II.	98	80.28	26.77	196	78.03	31.55	followup	4.00	0.07	[-0.17; 0.32]
Girgis, A, et al., 2009 II.	120	79.90	17.40	117	78.60	16.70	followup	24.00	0.08	[-0.18; 0.33]
Klinkhammer-Schalke, M et al., 2012	100	64.13	23.56	100	62.32	21.38	followup	48.00	0.08	[-0.20; 0.36]
Marchioro, G, et al. 1996	18	-54.17	16.90	18	-56.33	17.72	followup	4.00	0.12	[-0.53; 0.78]
Girgis, A, et al., 2009 I.	110	78.30	19.40	117	76.00	16.30	followup	12.00	0.13	[-0.13; 0.39]
Breitbart, W, et al. 2018 II.	86	6.65	1.60	74	6.44	1.50	followup	16.00	0.13	[-0.18; 0.45]
Powell, CB, et al., 2008	21	80.40	26.30	43	77.10	21.20	followup	12.00	0.14	[-0.38; 0.66]
Northhouse, LL, et al., 2007	112	87.20	10.60	123	85.50	10.30	followup	16.00	0.16	[-0.09; 0.42]
Klinkhammer-Schalke, M et al., 2012	100	65.21	22.09	100	61.23	23.55	followup	36.00	0.17	[-0.10; 0.45]
Girgis, A, et al., 2009 II.	120	79.00	16.40	117	76.00	16.30	followup	12.00	0.18	[-0.07; 0.44]
Elyasi, F, et al., 2021 I.	15	4.30	1.00	15	4.10	1.10	followup	24.00	0.19	[-0.53; 0.90]
Nápoles AM, et al. 2015	76	77.24	15.13	75	74.39	15.34	followup	12.00	0.19	[-0.13; 0.51]
Elyasi, F, et al., 2021 II.	20	4.30	1.00	20	4.10	1.10	followup	24.00	0.19	[-0.43; 0.81]
Qiu, H, et al., 2018 II.	98	89.52	33.46	196	83.21	31.55	followup	12.00	0.20	[-0.05; 0.44]
Qiu, H, et al., 2018 II.	98	94.70	35.69	196	87.72	34.70	followup	24.00	0.20	[-0.04; 0.44]
Serfaty M, et al., 2018	20	67.70	23.80	22	63.20	16.60	followup	24.00	0.22	[-0.39; 0.82]
Breitbart, W, et al. 2018 I.	94	6.87	1.70	74	6.48	1.60	followup	4.00	0.23	[-0.07; 0.54]
Hall S ,et al., 2011 I.	22	13.17	4.75	23	12.00	4.85	followup	1.00	0.24	[-0.35; 0.83]
Nápoles AM, et al. 2015	76	80.64	13.64	75	77.02	15.62	followup	24.00	0.25	[-0.07; 0.57]
Kissane, DW, et al., 2023	55	27.40	7.70	52	25.40	7.80	followup	24.00	0.26	[-0.12; 0.64]
Dirksen, S. et al, 2007	34	91.60	15.00	38	87.70	14.70	followup	10.00	0.26	[-0.20; 0.72]
Serfaty M, et al., 2018	20	72.90	19.10	22	67.90	16.70	followup	18.00	0.27	[-0.33; 0.88]
Kissane, DW, et al., 2023	55	27.90	8.10	52	24.80	8.90	followup	12.00	0.36	[-0.02; 0.74]
Thomas, ML, et al., 2012	64	70.50	17.30	88	64.40	16.30	followup	12.00	0.36	[0.04; 0.69]
Klinkhammer-Schalke, M et al., 2012	100	63.41	22.11	100	55.07	23.19	followup	24.00	0.37	[0.09; 0.65]
Walczak, A, et al.,2017	61	77.76	18.80	49	70.87	16.33	followup	4.00	0.39	[0.01; 0.77]
Lu, Z, et al., 201	203	64.40	5.00	103	62.30	6.00	followup	9.00	0.39	[0.15; 0.63]
Breitbart, W, et al. 2018 I.	94	7.06	1.50	74	6.44	1.50	followup	16.00	0.41	[0.10; 0.72]
Zhang, J, et al., 2021	61	9.67	3.71	61	8.21	3.02	followup	12.00	0.43	[0.07; 0.79]
Qiu, H, et al., 2018 I.	98	91.32	29.00	196	78.03	31.55	followup	4.00	0.43	[0.19; 0.68]
Breitbart, W, et al. 2018 I.	94	7.33	1.70	74	6.49	1.50	followup	8.00	0.52	[0.21; 0.83]
Ham, K, et al. 2019	21	54.69	19.80	21	45.02	16.05	followup	10.00	0.53	[-0.09; 1.14]
Peoples, AR, et al., 2016	24	87.90	13.90	24	79.70	15.70	followup	7.00	0.54	[-0.03; 1.12]
Gaston-Johansson, F, et al., 2011	38	24.70	4.30	35	21.90	5.00	followup	48.00	0.60	[0.13; 1.07]
Garssen B, et al., 2012	34	78.40	3.70	36	76.40	2.90	followup	5.00	0.60	[0.12; 1.08]
Garssen B, et al., 2012	34	80.40	3.30	36	78.20	3.20	followup	2.00	0.67	[0.19; 1.15]
Garssen B, et al., 2012	34	75.50	3.60	36	72.90	3.80	followup	13.00	0.69	[0.21; 1.18]
Peoples, AR, et al., 2016	24	91.40	15.00	24	78.60	20.60	followup	12.00	0.70	[0.11; 1.28]
Qiu, H, et al., 2018 I.	98	113.18	35.69	196	87.72	34.70	followup	24.00	0.72	[0.48; 0.97]
Qiu, H, et al., 2018 I.	98	107.32	31.23	196	83.21	31.55	followup	12.00	0.76	[0.51; 1.02]
Chu, X. et al, 2020	42	85.41	16.08	42	71.90	18.50	followup	12.00	0.77	[0.33; 1.22]
Chu, X. et al, 2020	42	84.94	14.59	42	71.59	18.73	followup	8.00	0.79	[0.34; 1.23]
Gao Q, et al. 2020	40	-54.26	7.03	40	-60.17	7.28	followup	4.00	0.82	[0.36; 1.27]
Garssen B, et al., 2012	34	81.00	3.40	36	78.20	3.20	followup	4.00	0.84	[0.35; 1.33]
Marchioro, G, et al. 1996	18	-48.72	12.22	18	-60.78	14.85	followup	12.00	0.87	[0.18; 1.55]
Rodin, G, et al., 2019	22	110.14	6.83	20	103.57	7.71	followup	4.00	0.89	[0.25; 1.53]
Rodin, G, et al., 2019	22	110.41	7.29	20	103.70	7.00	followup	8.00	0.92	[0.28; 1.56]
van der Meulen, IC, et al., 2013	88	77.50	2.80	91	74.50	2.60	followup	48.00	1.11	[0.79; 1.42]
Li, X, et al., 2017	102	76.58	9.06	108	64.28	12.49	followup	12.00	1.12	[0.83; 1.41]
Zhao,X, et al. 2015	62	10.65	2.02	62	8.22	2.07	followup	0.30	1.18	[0.80; 1.56]
Marchioro, G, et al. 1996	18	-44.72	9.18	18	-60.78	13.26	followup	24.00	1.38	[0.64; 2.11]
Beatty, L. et al. 2015	30	68.50	3.64	30	62.85	3.64	followup	13.03	1.53	[0.95; 2.11]
Marchioro, G, et al. 1996	18	-41.17	6.91	18	-60.28	13.33	followup	36.00	1.76	[0.98; 2.54]
Rodin, G, et al., 2019	22	114.37	6.97	20	100.61	7.50	followup	12.00	1.87	[1.13; 2.60]
Cheung YL, et al. 2002	29	7.48	1.12	30	5.56	0.50	followup	10.00	2.20	[1.54; 2.85]
Fann, JR, et al., 2009	112	6.30	0.25	103	5.74	0.25	followup	24.00	2.23	[1.89; 2.57]
Beatty, L. et al. 2015	30	74.04	3.78	30	65.27	3.81	followup	26.07	2.28	[1.62; 2.94]
Zhao, X, et al., 2021	52	66.68	3.21	51	55.07	6.32	followup	12.00	2.31	[1.80; 2.81]
Fann, JR, et al., 2009	112	6.51	0.25	103	5.84	0.29	followup	96.00	2.47	[2.12; 2.83]
Fann, JR, et al., 2009	112	6.33	0.22	103	5.72	0.26	followup	12.00	2.53	[2.17; 2.89]
Cheung YL, et al. 2002	29	7.51	0.98	30	4.96	0.66	followup	5.00	3.02	[2.26; 3.78]
Fann, JR, et al., 2009	112	6.67	0.23	103	5.95	0.24	followup	48.00	3.06	[2.66; 3.45]
Northouse, LL, et al., 2015	17	88.51	3.03	19	76.94	3.44	followup	2.00	3.48	[2.41; 4.55]
Fann, JR, et al., 2009	112	6.33	0.25	103	5.35	0.24	followup	72.00	3.98	[3.52; 4.45]
Random effect	7706			8192					0.68	[0.37; 0.99]
Prediction interval										[-0.59; 1.95]
**group**										
Heiney, SP, et al., 2003	33	6.00	1.70	33	6.40	1.40	baseline	0.00	-0.25	[-0.74; 0.23]
Liu, T, et al., 2019	49	66.61	10.85	53	69.54	15.27	baseline	0.00	-0.22	[-0.61; 0.17]
Lee, JT, et al., 2022	29	3.50	0.60	25	3.60	0.80	baseline	0.00	-0.14	[-0.68; 0.39]
Peng, L, et al., 2022	28	69.05	16.49	29	71.26	17.05	baseline	0.00	-0.13	[-0.65; 0.39]
Penedo, FJ, et al., 2007	41	-82.35	14.02	30	-80.57	13.21	baseline	0.00	-0.13	[-0.60; 0.34]
Von Ah, D, et al.,2012 I.	26	22.06	3.44	29	22.38	2.24	baseline	0.00	-0.11	[-0.64; 0.42]
Schellekens, MPJ, et al. 2016	69	95.43	21.60	70	97.09	23.54	baseline	0.00	-0.07	[-0.41; 0.26]
Compen F, et al. 2020 I.	77	0.75	0.21	78	0.76	0.17	baseline	0.00	-0.05	[-0.37; 0.26]
Reich, RR, et al. 2016	167	62.44	27.52	155	62.74	24.68	baseline	0.00	-0.01	[-0.23; 0.21]
Xia, S, et al., 2023	80	61.40	16.60	80	61.10	15.70	baseline	0.00	0.02	[-0.29; 0.33]
Nikbakhsh, N, et al., 2018	20	44.09	8.48	20	43.64	13.73	baseline	0.00	0.04	[-0.58; 0.66]
Compen F, et al. 2020 II.	90	0.77	0.19	78	0.76	0.17	baseline	0.00	0.05	[-0.25; 0.36]
Esplen MJ, et al., 2018	131	70.50	16.50	63	68.90	17.20	baseline	0.00	0.10	[-0.21; 0.40]
Mihuta, ME, et al., 2018	32	11.90	4.40	33	11.50	3.60	baseline	0.00	0.10	[-0.39; 0.59]
Hernandez, EG, et al. 2018	28	64.86	20.04	28	62.85	15.86	baseline	0.00	0.11	[-0.41; 0.63]
Von Ah, D, et al.,2012 II.	27	22.98	3.08	29	22.38	2.24	baseline	0.00	0.22	[-0.30; 0.75]
Rahmani, S, et al. 2015	12	41.66	6.15	12	39.58	7.22	baseline	0.00	0.30	[-0.51; 1.10]
Yoo, HJ, et al. 2004	30	93.41	16.90	30	87.88	14.96	baseline	0.00	0.34	[-0.17; 0.85]
Cengiz, HO, et al., 2023	32	63.06	19.74	33	55.22	18.26	baseline	0.00	0.41	[-0.08; 0.90]
Jelvehzadeh, F, et al., 2022	24	4.71	1.07	24	3.94	1.06	baseline	0.00	0.71	[0.13; 1.30]
Penedo, FJ, et al., 2007	41	-87.22	13.82	30	-79.63	14.56	followup	12.00	-0.53	[-1.01; -0.05]
Heiney, SP, et al., 2003	33	6.00	1.50	33	6.80	1.50	followup	16.00	-0.53	[-1.02; -0.04]
Von Ah, D, et al.,2012 I.	26	22.15	4.45	29	22.58	2.24	followup	8.00	-0.12	[-0.65; 0.41]
Peng, L, et al., 2022	28	66.67	24.00	29	69.25	22.17	followup	4.00	-0.11	[-0.63; 0.41]
Reich, RR, et al. 2016	167	68.43	27.76	155	70.36	22.70	followup	12.00	-0.08	[-0.29; 0.14]
Heiney, SP, et al., 2003	33	6.20	1.30	33	6.30	1.60	followup	6.00	-0.07	[-0.55; 0.41]
Esplen MJ, et al., 2018	131	73.40	16.70	63	74.30	17.70	followup	48.00	-0.05	[-0.35; 0.25]
Reich, RR, et al. 2016	167	65.76	26.18	155	66.24	24.76	followup	6.00	-0.02	[-0.24; 0.20]
Rahmani, S, et al. 2015	12	12.00	61.11	12	12.00	58.33	followup	16.00	0.00	[-0.80; 0.80]
Peng, L, et al., 2022	28	72.32	22.11	29	71.26	15.26	followup	0.10	0.06	[-0.46; 0.57]
Von Ah, D, et al.,2012 II.	27	22.71	2.08	29	22.58	2.24	followup	8.00	0.06	[-0.47; 0.58]
Lee, JT, et al., 2022	29	4.05	0.60	25	4.00	0.60	followup	12.00	0.08	[-0.45; 0.62]
Lee, JT, et al., 2022	29	4.05	0.60	25	4.00	0.60	followup	24.00	0.08	[-0.45; 0.62]
Esplen MJ, et al., 2018	131	14.90	9.40	63	14.00	9.40	followup	8.00	0.10	[-0.21; 0.40]
Xia, S, et al., 2023	80	67.70	16.20	80	65.80	14.40	followup	4.00	0.12	[-0.19; 0.43]
Esplen MJ, et al., 2018	131	72.60	16.60	63	70.30	18.00	followup	24.00	0.13	[-0.17; 0.44]
Schellekens, MPJ, et al. 2016	69	107.36	18.80	70	103.34	22.35	followup	8.00	0.19	[-0.14; 0.53]
Xia, S, et al., 2023	80	72.70	15.80	80	68.00	13.50	followup	12.00	0.32	[0.01; 0.63]
Xia, S, et al., 2023	80	78.00	14.30	80	72.90	13.40	followup	24.00	0.37	[0.05; 0.68]
Yoo, HJ, et al. 2004	30	97.01	18.24	30	88.13	16.18	followup	12.00	0.51	[-0.01; 1.02]
Hernandez, EG, et al. 2018	28	71.73	15.45	28	62.80	17.63	followup	24.00	0.53	[-0.00; 1.06]
Compen F, et al. 2020 II.	90	0.85	0.17	78	0.75	0.19	followup	8.00	0.55	[0.25; 0.86]
Mihuta, ME, et al., 2018	32	14.10	2.60	33	12.30	3.60	followup	4.00	0.56	[0.07; 1.06]
Compen F, et al. 2020 I.	77	0.86	0.13	78	0.75	0.19	followup	8.00	0.67	[0.35; 1.00]
Hernandez, EG, et al. 2018	28	74.38	11.48	28	64.40	14.73	followup	8.00	0.75	[0.20; 1.29]
Cengiz, HO, et al., 2023	32	66.98	17.72	33	53.03	18.65	followup	8.00	0.76	[0.25; 1.26]
Yoo, HJ, et al. 2004	30	102.00	19.94	30	88.18	14.02	followup	24.00	0.79	[0.26; 1.32]
Rahmani, S, et al. 2015	12	66.66	9.40	12	54.16	10.95	followup	8.00	1.18	[0.30; 2.06]
Liu, T, et al., 2019	49	62.67	10.32	53	49.47	10.92	followup	9.00	1.23	[0.81; 1.66]
Liu, T, et al., 2019	49	72.33	13.36	53	56.95	10.03	followup	12.00	1.30	[0.87; 1.73]
Jelvehzadeh, F, et al., 2022	24	6.05	1.00	24	4.65	0.80	followup	12.00	1.52	[0.87; 2.17]
Jelvehzadeh, F, et al., 2022	24	6.16	1.09	24	4.58	0.92	followup	8.00	1.54	[0.89; 2.19]
Nikbakhsh, N, et al., 2018	20	81.70	10.04	20	47.39	12.93	followup	12.00	2.91	[1.99; 3.82]
Random effect	2872			2539					0.61	[0.27; 0.95]
Prediction interval										[-0.66; 1.89]
**self-help**										
van den Berg, SW, et al. 2015 II.	70	66.79	16.57	80	69.79	17.91	baseline	0.00	-0.17	[-0.49; 0.15]
Wang, TJ, et al 2023	70	102.30	18.40	72	104.40	17.10	baseline	0.00	-0.12	[-0.45; 0.21]
van der Hout, AVD, et al. 2019	320	85.30	14.90	304	85.40	13.60	baseline	0.00	-0.01	[-0.16; 0.15]
Walker, LG, et al.,1998	48	3.25	0.70	48	3.25	0.66	baseline	0.00	0.00	[-0.40; 0.40]
Rosen, KD, et al., 2018	57	93.61	22.68	55	92.96	25.12	baseline	0.00	0.03	[-0.34; 0.40]
Wang, TJ, et al 2023	70	105.40	17.80	72	103.50	20.00	followup	8.00	0.10	[-0.23; 0.43]
van der Hout, AVD, et al. 2019	320	88.70	13.20	304	86.50	13.10	followup	12.00	0.17	[0.01; 0.32]
van der Hout, AVD, et al. 2019	320	88.40	12.10	304	86.20	12.80	followup	1.00	0.18	[0.02; 0.33]
van der Hout, AVD, et al. 2019	320	89.30	12.30	304	87.00	12.70	followup	24.00	0.18	[0.03; 0.34]
Wang, TJ, et al 2023	70	107.30	16.70	72	103.30	19.00	followup	16.00	0.22	[-0.11; 0.55]
Wang, TJ, et al 2023	70	106.90	16.60	72	102.70	20.00	followup	24.00	0.23	[-0.10; 0.56]
Walker, LG, et al.,1998	48	3.29	0.80	48	2.90	0.78	followup	15.00	0.49	[0.08; 0.90]
Rosen, KD, et al., 2018	57	107.35	17.79	55	96.04	23.96	followup	5.00	0.53	[0.16; 0.91]
van den Berg, SW, et al. 2015 II.	70	73.30	1.78	80	69.88	1.67	followup	4.00	1.97	[1.58; 2.36]
Random effect	1910			1870					0.59	[0.10; 1.08]
Prediction interval										[-0.96; 2.13]
**Random effect**	**12488**			**12601**					**0.65**	**[0.35; 0.94]**
**Prediction interval**										**[-0.61; 1.91]**

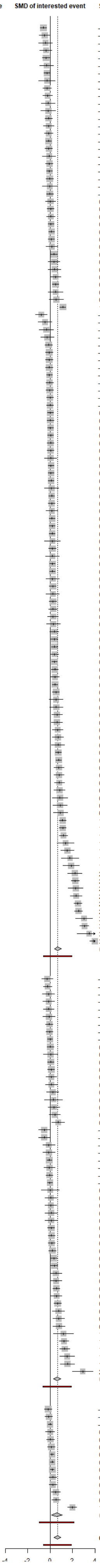

Figure S5.1. Subgroup analysis of the Global QoL Forest plot represents the difference between the intervention vs. control group in the Global QoL domain with the type subgroups as predicted at week 0 (post-intervention). SMD - Standardized mean difference, CI - confidence interval.

Figure S5.2.T12

Study	Experimental Patient N	Experimental Mean	Experimental SD	Control Patient N	Control Mean	Control SD	follow-up	Follow-up time	SMD	95%-CI
individual										
Li, X, et al., 2017	102	56.32	7.80	108	62.38	11.46	baseline	0.00	-0.61	[-0.89; -0.34]
Garssen B, et al., 2012	34	74.40	4.20	36	76.50	4.30	baseline	0.00	-0.49	[-0.96; -0.01]
Rodin, G, et al., 2019	22	103.92	6.45	20	106.71	6.80	baseline	0.00	-0.41	[-1.03; 0.20]
Seliniotaki, T, et al., 2021	27	62.00	33.80	26	72.20	16.20	baseline	0.00	-0.38	[-0.92; 0.17]
Zhao,X, et al. 2015	62	7.85	2.52	62	8.75	2.38	baseline	0.00	-0.36	[-0.72; -0.01]
Serfaty M, et al., 2018	20	56.40	15.50	22	61.40	14.40	baseline	0.00	-0.33	[-0.94; 0.28]
Fann, JR, et al., 2009	112	5.39	0.21	103	5.45	0.20	baseline	0.00	-0.29	[-0.56; -0.02]
Elyasi, F, et al., 2021 I.	15	3.30	1.90	15	3.80	1.50	baseline	0.00	-0.28	[-1.00; 0.44]
Walczak, A, et al.,2017	61	71.84	16.33	49	75.99	15.72	baseline	0.00	-0.26	[-0.63; 0.12]
Chu, X. et al, 2020	42	65.20	16.77	42	68.91	16.63	baseline	0.00	-0.22	[-0.65; 0.21]
Elyasi, F, et al., 2021 II.	20	3.50	1.80	20	3.80	1.50	baseline	0.00	-0.18	[-0.80; 0.44]
Savard, J, et al., 2006	21	42.51	3.19	16	43.11	3.91	baseline	0.00	-0.17	[-0.82; 0.48]
Nápoles AM, et al. 2015	76	66.46	16.92	75	68.83	15.33	baseline	0.00	-0.15	[-0.47; 0.17]
Dirksen, S. et al, 2007	34	83.30	11.90	38	84.80	9.20	baseline	0.00	-0.14	[-0.60; 0.32]
Zhao, X, et al., 2021	52	53.70	3.14	51	54.69	9.61	baseline	0.00	-0.14	[-0.52; 0.25]
Qiu, H, et al., 2018 II.	98	76.78	19.91	196	79.49	21.60	baseline	0.00	-0.13	[-0.37; 0.11]
Sandsund, C, et al., 2017	72	64.20	21.80	70	66.70	19.70	baseline	0.00	-0.12	[-0.45; 0.21]
Hall S ,et al., 2011 I.	22	12.32	4.35	23	12.74	4.26	baseline	0.00	-0.10	[-0.68; 0.49]
Reese, JB, et al., 2021	73	60.80	16.70	71	61.90	14.30	baseline	0.00	-0.07	[-0.40; 0.26]
Qiu, H, et al., 2018 I.	98	78.09	18.86	196	79.49	21.60	baseline	0.00	-0.07	[-0.31; 0.18]
Chan, et al. 2005	80	58.29	50.71	75	60.44	40.96	baseline	0.00	-0.05	[-0.36; 0.27]
Marchioro, G, et al. 1996	18	-58.44	22.39	18	-57.72	20.25	baseline	0.00	-0.03	[-0.69; 0.62]
Breitbart, W, et al. 2018 II.	86	6.19	1.60	74	6.24	1.60	baseline	0.00	-0.03	[-0.34; 0.28]
Gao Q, et al. 2020	40	-68.20	8.25	40	-68.44	8.82	baseline	0.00	0.03	[-0.41; 0.47]
Breitbart, W, et al. 2018 I.	94	6.29	1.50	74	6.24	1.60	baseline	0.00	0.03	[-0.27; 0.34]
Kissane, DW, et al., 2023	55	25.80	8.30	52	25.30	8.90	baseline	0.00	0.06	[-0.32; 0.44]
Girgis, A, et al., 2009 I.	110	73.20	19.60	117	71.90	17.60	baseline	0.00	0.07	[-0.19; 0.33]
Girgis, A, et al., 2009 II.	120	74.00	18.20	117	71.90	17.60	baseline	0.00	0.12	[-0.14; 0.37]
Klinkhammer-Schalke, M et al., 2012	100	54.35	26.09	100	51.45	23.19	baseline	0.00	0.12	[-0.16; 0.39]
Powell, CB, et al., 2008	21	78.30	17.70	43	75.60	17.90	baseline	0.00	0.15	[-0.37; 0.67]
Thomas, ML, et al., 2012	64	68.80	15.90	88	63.60	15.60	baseline	0.00	0.33	[0.00; 0.65]
Cheung YL, et al. 2002	29	4.96	1.74	30	4.36	1.62	baseline	0.00	0.35	[-0.16; 0.87]
Peoples, AR, et al., 2016	24	78.90	15.30	24	71.90	19.70	baseline	0.00	0.39	[-0.18; 0.96]
Gaston-Johansson, F, et al., 2011	38	22.60	4.30	35	20.60	4.00	baseline	0.00	0.48	[0.01; 0.94]
van der Meulen, IC, et al., 2013	88	67.30	2.30	91	66.20	2.30	baseline	0.00	0.48	[0.18; 0.77]
Northouse, LL, et al., 2015	17	81.30	3.88	19	79.63	2.96	baseline	0.00	0.48	[-0.19; 1.14]
Ham, K, et al. 2019	21	47.69	18.42	21	38.22	15.47	baseline	0.00	0.55	[-0.07; 1.16]
Lu, Z, et al., 201	203	61.70	4.00	103	56.30	6.00	baseline	0.00	1.13	[0.88; 1.38]
Beatty, L. et al. 2015	30	56.22	3.88	30	59.37	3.92	followup	6.00	-0.80	[-1.32; -0.27]
Serfaty M, et al., 2018	20	63.80	19.80	22	72.20	16.60	followup	12.00	-0.45	[-1.07; 0.16]
Savard, J, et al., 2006	21	3.62	0.95	16	3.95	0.91	followup	8.00	-0.34	[-1.00; 0.31]
Hall S ,et al., 2011 I.	22	12.50	4.99	23	13.70	3.89	followup	4.00	-0.26	[-0.85; 0.32]
Chan, et al. 2005	80	60.31	41.32	75	66.46	47.98	followup	12.00	-0.14	[-0.45; 0.18]
Sandsund, C, et al., 2017	72	67.50	22.60	70	69.40	21.10	followup	24.00	-0.09	[-0.42; 0.24]
Chan, et al. 2005	80	71.69	74.33	75	77.23	63.91	followup	72.00	-0.08	[-0.39; 0.24]
Chan, et al. 2005	80	71.61	51.52	75	75.69	53.26	followup	60.00	-0.08	[-0.39; 0.24]
Klinkhammer-Schalke, M et al., 2012	100	53.62	22.46	100	55.07	20.29	followup	12.00	-0.07	[-0.34; 0.21]
Sandsund, C, et al., 2017	72	66.20	19.10	70	67.30	22.00	followup	12.00	-0.05	[-0.38; 0.28]
Reese, JB, et al., 2021	73	61.80	18.20	71	62.60	13.90	followup	8.00	-0.05	[-0.38; 0.28]
Chan, et al. 2005	80	73.13	34.88	75	73.75	47.11	followup	36.00	-0.01	[-0.33; 0.30]
Breitbart, W, et al. 2018 II.	86	6.47	1.20	74	6.48	1.60	followup	4.00	-0.01	[-0.32; 0.30]
Northhouse, LL, et al., 2007	107	87.00	10.80	121	86.90	10.60	followup	32.00	0.01	[-0.25; 0.27]
Chan, et al. 2005	80	67.38	46.78	75	66.55	51.44	followup	24.00	0.02	[-0.30; 0.33]
Northhouse, LL, et al., 2007	104	86.10	10.90	114	85.80	10.70	followup	48.00	0.03	[-0.24; 0.29]
Girgis, A, et al., 2009 I.	110	79.20	19.20	117	78.60	16.70	followup	24.00	0.03	[-0.23; 0.29]
Chan, et al. 2005	80	77.86	49.64	75	76.04	45.64	followup	48.00	0.04	[-0.28; 0.35]
Breitbart, W, et al. 2018 II.	86	6.55	1.60	74	6.49	1.50	followup	8.00	0.04	[-0.27; 0.35]
Seliniotaki, T, et al., 2021	27	65.00	28.00	26	64.00	20.50	followup	8.00	0.04	[-0.50; 0.58]
Qiu, H, et al., 2018 II.	98	80.28	26.77	196	78.03	31.55	followup	4.00	0.07	[-0.17; 0.32]
Girgis, A, et al., 2009 II.	120	79.90	17.40	117	78.60	16.70	followup	24.00	0.08	[-0.18; 0.33]
Klinkhammer-Schalke, M et al., 2012	100	64.13	23.56	100	62.32	21.38	followup	48.00	0.08	[-0.20; 0.36]
Marchioro, G, et al. 1996	18	-54.17	16.90	18	-56.33	17.72	followup	4.00	0.12	[-0.53; 0.78]
Girgis, A, et al., 2009 I.	110	78.30	19.40	117	76.00	16.30	followup	12.00	0.13	[-0.13; 0.39]
Breitbart, W, et al. 2018 II.	86	6.65	1.60	74	6.44	1.50	followup	16.00	0.13	[-0.18; 0.45]
Powell, CB, et al., 2008	21	80.40	26.30	43	77.10	21.20	followup	12.00	0.14	[-0.38; 0.66]
Northhouse, LL, et al., 2007	112	87.20	10.60	123	85.50	10.30	followup	16.00	0.16	[-0.09; 0.42]
Klinkhammer-Schalke, M et al., 2012	100	65.21	22.09	100	61.23	23.55	followup	36.00	0.17	[-0.10; 0.45]
Girgis, A, et al., 2009 II.	120	79.00	16.40	117	76.00	16.30	followup	12.00	0.18	[-0.07; 0.44]
Elyasi, F, et al., 2021 I.	15	4.30	1.00	15	4.10	1.10	followup	24.00	0.19	[-0.53; 0.90]
Nápoles AM, et al. 2015	76	77.24	15.13	75	74.39	15.34	followup	12.00	0.19	[-0.13; 0.51]
Elyasi, F, et al., 2021 II.	20	4.30	1.00	20	4.10	1.10	followup	24.00	0.19	[-0.43; 0.81]
Qiu, H, et al., 2018 II.	98	89.52	33.46	196	83.21	31.55	followup	12.00	0.20	[-0.05; 0.44]
Qiu, H, et al., 2018 II.	98	94.70	35.69	196	87.72	34.70	followup	24.00	0.20	[-0.04; 0.44]
Serfaty M, et al., 2018	20	67.70	23.80	22	63.20	16.60	followup	24.00	0.22	[-0.39; 0.82]
Breitbart, W, et al. 2018 I.	94	6.87	1.70	74	6.48	1.60	followup	4.00	0.23	[-0.07; 0.54]
Hall S ,et al., 2011 I.	22	13.17	4.75	23	12.00	4.85	followup	1.00	0.24	[-0.35; 0.83]
Nápoles AM, et al. 2015	76	80.64	13.64	75	77.02	15.62	followup	24.00	0.25	[-0.07; 0.57]
Kissane, DW, et al., 2023	55	27.40	7.70	52	25.40	7.80	followup	24.00	0.26	[-0.12; 0.64]
Dirksen, S. et al, 2007	34	91.60	15.00	38	87.70	14.70	followup	10.00	0.26	[-0.20; 0.72]
Serfaty M, et al., 2018	20	72.90	19.10	22	67.90	16.70	followup	18.00	0.27	[-0.33; 0.88]
Kissane, DW, et al., 2023	55	27.90	8.10	52	24.80	8.90	followup	12.00	0.36	[-0.02; 0.74]
Thomas, ML, et al., 2012	64	70.50	17.30	88	64.40	16.30	followup	12.00	0.36	[0.04; 0.69]
Klinkhammer-Schalke, M et al., 2012	100	63.41	22.11	100	55.07	23.19	followup	24.00	0.37	[0.09; 0.65]
Walczak, A, et al.,2017	61	77.76	18.80	49	70.87	16.33	followup	4.00	0.39	[0.01; 0.77]
Lu, Z, et al., 201	203	64.40	5.00	103	62.30	6.00	followup	9.00	0.39	[0.15; 0.63]
Breitbart, W, et al. 2018 I.	94	7.06	1.50	74	6.44	1.50	followup	16.00	0.41	[0.10; 0.72]
Zhang, J, et al., 2021	61	9.67	3.71	61	8.21	3.02	followup	12.00	0.43	[0.07; 0.79]
Qiu, H, et al., 2018 I.	98	91.32	29.00	196	78.03	31.55	followup	4.00	0.43	[0.19; 0.68]
Breitbart, W, et al. 2018 I.	94	7.33	1.70	74	6.49	1.50	followup	8.00	0.52	[0.21; 0.83]
Ham, K, et al. 2019	21	54.69	19.80	21	45.02	16.05	followup	10.00	0.53	[-0.09; 1.14]
Peoples, AR, et al., 2016	24	87.90	13.90	24	79.70	15.70	followup	7.00	0.54	[-0.03; 1.12]
Gaston-Johansson, F, et al., 2011	38	24.70	4.30	35	21.90	5.00	followup	48.00	0.60	[0.13; 1.07]
Garssen B, et al., 2012	34	78.40	3.70	36	76.40	2.90	followup	5.00	0.60	[0.12; 1.08]
Garssen B, et al., 2012	34	80.40	3.30	36	78.20	3.20	followup	2.00	0.67	[0.19; 1.15]
Garssen B, et al., 2012	34	75.50	3.60	36	72.90	3.80	followup	13.00	0.69	[0.21; 1.18]
Peoples, AR, et al., 2016	24	91.40	15.00	24	78.60	20.60	followup	12.00	0.70	[0.11; 1.28]
Qiu, H, et al., 2018 I.	98	113.18	35.69	196	87.72	34.70	followup	24.00	0.72	[0.48; 0.97]
Qiu, H, et al., 2018 I.	98	107.32	31.23	196	83.21	31.55	followup	12.00	0.76	[0.51; 1.02]
Chu, X. et al, 2020	42	85.41	16.08	42	71.90	18.50	followup	12.00	0.77	[0.33; 1.22]
Chu, X. et al, 2020	42	84.94	14.59	42	71.59	18.73	followup	8.00	0.79	[0.34; 1.23]
Gao Q, et al. 2020	40	-54.26	7.03	40	-60.17	7.28	followup	4.00	0.82	[0.36; 1.27]
Garssen B, et al., 2012	34	81.00	3.40	36	78.20	3.20	followup	4.00	0.84	[0.35; 1.33]
Marchioro, G, et al. 1996	18	-48.72	12.22	18	-60.78	14.85	followup	12.00	0.87	[0.18; 1.55]
Rodin, G, et al., 2019	22	110.14	6.83	20	103.57	7.71	followup	4.00	0.89	[0.25; 1.53]
Rodin, G, et al., 2019	22	110.41	7.29	20	103.70	7.00	followup	8.00	0.92	[0.28; 1.56]
van der Meulen, IC, et al., 2013	88	77.50	2.80	91	74.50	2.60	followup	48.00	1.11	[0.79; 1.42]
Li, X, et al., 2017	102	76.58	9.06	108	64.28	12.49	followup	12.00	1.12	[0.83; 1.41]
Zhao,X, et al. 2015	62	10.65	2.02	62	8.22	2.07	followup	0.30	1.18	[0.80; 1.56]
Marchioro, G, et al. 1996	18	-44.72	9.18	18	-60.78	13.26	followup	24.00	1.38	[0.64; 2.11]
Beatty, L. et al. 2015	30	68.50	3.64	30	62.85	3.64	followup	13.03	1.53	[0.95; 2.11]
Marchioro, G, et al. 1996	18	-41.17	6.91	18	-60.28	13.33	followup	36.00	1.76	[0.98; 2.54]
Rodin, G, et al., 2019	22	114.37	6.97	20	100.61	7.50	followup	12.00	1.87	[1.13; 2.60]
Cheung YL, et al. 2002	29	7.48	1.12	30	5.56	0.50	followup	10.00	2.20	[1.54; 2.85]
Fann, JR, et al., 2009	112	6.30	0.25	103	5.74	0.25	followup	24.00	2.23	[1.89; 2.57]
Beatty, L. et al. 2015	30	74.04	3.78	30	65.27	3.81	followup	26.07	2.28	[1.62; 2.94]
Zhao, X, et al., 2021	52	66.68	3.21	51	55.07	6.32	followup	12.00	2.31	[1.80; 2.81]
Fann, JR, et al., 2009	112	6.51	0.25	103	5.84	0.29	followup	96.00	2.47	[2.12; 2.83]
Fann, JR, et al., 2009	112	6.33	0.22	103	5.72	0.26	followup	12.00	2.53	[2.17; 2.89]
Cheung YL, et al. 2002	29	7.51	0.98	30	4.96	0.66	followup	5.00	3.02	[2.26; 3.78]
Fann, JR, et al., 2009	112	6.67	0.23	103	5.95	0.24	followup	48.00	3.06	[2.66; 3.45]
Northouse, LL, et al., 2015	17	88.51	3.03	19	76.94	3.44	followup	2.00	3.48	[2.41; 4.55]
Fann, JR, et al., 2009	112	6.33	0.25	103	5.35	0.24	followup	72.00	3.98	[3.52; 4.45]
Random effect	7706			8192					0.51	[0.32; 0.70]
Prediction interval										[-0.73; 1.75]
group										
Heiney, SP, et al., 2003	33	6.00	1.70	33	6.40	1.40	baseline	0.00	-0.25	[-0.74; 0.23]
Liu, T, et al., 2019	49	66.61	10.85	53	69.54	15.27	baseline	0.00	-0.22	[-0.61; 0.17]
Lee, JT, et al., 2022	29	3.50	0.60	25	3.60	0.80	baseline	0.00	-0.14	[-0.68; 0.39]
Peng, L, et al., 2022	28	69.05	16.49	29	71.26	17.05	baseline	0.00	-0.13	[-0.65; 0.39]
Penedo, FJ, et al., 2007	41	-82.35	14.02	30	-80.57	13.21	baseline	0.00	-0.13	[-0.60; 0.34]
Von Ah, D, et al.,2012 I.	26	22.06	3.44	29	22.38	2.24	baseline	0.00	-0.11	[-0.64; 0.42]
Schellekens, MPJ, et al. 2016	69	95.43	21.60	70	97.09	23.54	baseline	0.00	-0.07	[-0.41; 0.26]
Compen F, et al. 2020 I.	77	0.75	0.21	78	0.76	0.17	baseline	0.00	-0.05	[-0.37; 0.26]
Reich, RR, et al. 2016	167	62.44	27.52	155	62.74	24.68	baseline	0.00	-0.01	[-0.23; 0.21]
Xia, S, et al., 2023	80	61.40	16.60	80	61.10	15.70	baseline	0.00	0.02	[-0.29; 0.33]
Nikbakhsh, N, et al., 2018	20	44.09	8.48	20	43.64	13.73	baseline	0.00	0.04	[-0.58; 0.66]
Compen F, et al. 2020 II.	90	0.77	0.19	78	0.76	0.17	baseline	0.00	0.05	[-0.25; 0.36]
Esplen MJ, et al., 2018	131	70.50	16.50	63	68.90	17.20	baseline	0.00	0.10	[-0.21; 0.40]
Mihuta, ME, et al., 2018	32	11.90	4.40	33	11.50	3.60	baseline	0.00	0.10	[-0.39; 0.59]
Hernandez, EG, et al. 2018	28	64.86	20.04	28	62.85	15.86	baseline	0.00	0.11	[-0.41; 0.63]
Von Ah, D, et al.,2012 II.	27	22.98	3.08	29	22.38	2.24	baseline	0.00	0.22	[-0.30; 0.75]
Rahmani, S, et al. 2015	12	41.66	6.15	12	39.58	7.22	baseline	0.00	0.30	[-0.51; 1.10]
Yoo, HJ, et al. 2004	30	93.41	16.90	30	87.88	14.96	baseline	0.00	0.34	[-0.17; 0.85]
Cengiz, HO, et al., 2023	32	63.06	19.74	33	55.22	18.26	baseline	0.00	0.41	[-0.08; 0.90]
Jelvehzadeh, F, et al., 2022	24	4.71	1.07	24	3.94	1.06	baseline	0.00	0.71	[0.13; 1.30]
Penedo, FJ, et al., 2007	41	-87.22	13.82	30	-79.63	14.56	followup	12.00	-0.53	[-1.01; -0.05]
Heiney, SP, et al., 2003	33	6.00	1.50	33	6.80	1.50	followup	16.00	-0.53	[-1.02; -0.04]
Von Ah, D, et al.,2012 I.	26	22.15	4.45	29	22.58	2.24	followup	8.00	-0.12	[-0.65; 0.41]
Peng, L, et al., 2022	28	66.67	24.00	29	69.25	22.17	followup	4.00	-0.11	[-0.63; 0.41]
Reich, RR, et al. 2016	167	68.43	27.76	155	70.36	22.70	followup	12.00	-0.08	[-0.29; 0.14]
Heiney, SP, et al., 2003	33	6.20	1.30	33	6.30	1.60	followup	6.00	-0.07	[-0.55; 0.41]
Esplen MJ, et al., 2018	131	73.40	16.70	63	74.30	17.70	followup	48.00	-0.05	[-0.35; 0.25]
Reich, RR, et al. 2016	167	65.76	26.18	155	66.24	24.76	followup	6.00	-0.02	[-0.24; 0.20]
Rahmani, S, et al. 2015	12	12.00	61.11	12	12.00	58.33	followup	16.00	0.00	[-0.80; 0.80]
Peng, L, et al., 2022	28	72.32	22.11	29	71.26	15.26	followup	0.10	0.06	[-0.46; 0.57]
Von Ah, D, et al.,2012 II.	27	22.71	2.08	29	22.58	2.24	followup	8.00	0.06	[-0.47; 0.58]
Lee, JT, et al., 2022	29	4.05	0.60	25	4.00	0.60	followup	12.00	0.08	[-0.45; 0.62]
Lee, JT, et al., 2022	29	4.05	0.60	25	4.00	0.60	followup	24.00	0.08	[-0.45; 0.62]
Esplen MJ, et al., 2018	131	14.90	9.40	63	14.00	9.40	followup	8.00	0.10	[-0.21; 0.40]
Xia, S, et al., 2023	80	67.70	16.20	80	65.80	14.40	followup	4.00	0.12	[-0.19; 0.43]
Esplen MJ, et al., 2018	131	72.60	16.60	63	70.30	18.00	followup	24.00	0.13	[-0.17; 0.44]
Schellekens, MPJ, et al. 2016	69	107.36	18.80	70	103.34	22.35	followup	8.00	0.19	[-0.14; 0.53]
Xia, S, et al., 2023	80	72.70	15.80	80	68.00	13.50	followup	12.00	0.32	[0.01; 0.63]
Xia, S, et al., 2023	80	78.00	14.30	80	72.90	13.40	followup	24.00	0.37	[0.05; 0.68]
Yoo, HJ, et al. 2004	30	97.01	18.24	30	88.13	16.18	followup	12.00	0.51	[-0.01; 1.02]
Hernandez, EG, et al. 2018	28	71.73	15.45	28	62.80	17.63	followup	24.00	0.53	[-0.00; 1.06]
Compen F, et al. 2020 II.	90	0.85	0.17	78	0.75	0.19	followup	8.00	0.55	[0.25; 0.86]
Mihuta, ME, et al., 2018	32	14.10	2.60	33	12.30	3.60	followup	4.00	0.56	[0.07; 1.06]
Compen F, et al. 2020 I.	77	0.86	0.13	78	0.75	0.19	followup	8.00	0.67	[0.35; 1.00]
Hernandez, EG, et al. 2018	28	74.38	11.48	28	64.40	14.73	followup	8.00	0.75	[0.20; 1.29]
Cengiz, HO, et al., 2023	32	66.98	17.72	33	53.03	18.65	followup	8.00	0.76	[0.25; 1.26]
Yoo, HJ, et al. 2004	30	102.00	19.94	30	88.18	14.02	followup	24.00	0.79	[0.26; 1.32]
Rahmani, S, et al. 2015	12	66.66	9.40	12	54.16	10.95	followup	8.00	1.18	[0.30; 2.06]
Liu, T, et al., 2019	49	62.67	10.32	53	49.47	10.92	followup	9.00	1.23	[0.81; 1.66]
Liu, T, et al., 2019	49	72.33	13.36	53	56.95	10.03	followup	12.00	1.30	[0.87; 1.73]
Jelvehzadeh, F, et al., 2022	24	6.05	1.00	24	4.65	0.80	followup	12.00	1.52	[0.87; 2.17]
Jelvehzadeh, F, et al., 2022	24	6.16	1.09	24	4.58	0.92	followup	8.00	1.54	[0.89; 2.19]
Nikbakhsh, N, et al., 2018	20	81.70	10.04	20	47.39	12.93	followup	12.00	2.91	[1.99; 3.82]
Random effect	2872			2539					0.44	[0.21; 0.67]
Prediction interval										[-0.83; 1.72]
self-help										
van den Berg, SW, et al. 2015 II.	70	66.79	16.57	80	69.79	17.91	baseline	0.00	-0.17	[-0.49; 0.15]
Wang, TJ, et al 2023	70	102.30	18.40	72	104.40	17.10	baseline	0.00	-0.12	[-0.45; 0.21]
van der Hout, AVD, et al. 2019	320	85.30	14.90	304	85.40	13.60	baseline	0.00	-0.01	[-0.16; 0.15]
Walker, LG, et al.,1998	48	3.25	0.70	48	3.25	0.66	baseline	0.00	0.00	[-0.40; 0.40]
Rosen, KD, et al., 2018	57	93.61	22.68	55	92.96	25.12	baseline	0.00	0.03	[-0.34; 0.40]
Wang, TJ, et al 2023	70	105.40	17.80	72	103.50	20.00	followup	8.00	0.10	[-0.23; 0.43]
van der Hout, AVD, et al. 2019	320	88.70	13.20	304	86.50	13.10	followup	12.00	0.17	[0.01; 0.32]
van der Hout, AVD, et al. 2019	320	88.40	12.10	304	86.20	12.80	followup	1.00	0.18	[0.02; 0.33]
van der Hout, AVD, et al. 2019	320	89.30	12.30	304	87.00	12.70	followup	24.00	0.18	[0.03; 0.34]
Wang, TJ, et al 2023	70	107.30	16.70	72	103.30	19.00	followup	16.00	0.22	[-0.11; 0.55]
Wang, TJ, et al 2023	70	106.90	16.60	72	102.70	20.00	followup	24.00	0.23	[-0.10; 0.56]
Walker, LG, et al.,1998	48	3.29	0.80	48	2.90	0.78	followup	15.00	0.49	[0.08; 0.90]
Rosen, KD, et al., 2018	57	107.35	17.79	55	96.04	23.96	followup	5.00	0.53	[0.16; 0.91]
van den Berg, SW, et al. 2015 II.	70	73.30	1.78	80	69.88	1.67	followup	4.00	1.97	[1.58; 2.36]
Random effect	1910			1870					0.42	[-0.00; 0.83]
Prediction interval										[-1.38; 2.21]
**Random effect**	**12488**			**12601**					**0.48**	**[0.33; 0.64]**
**Prediction interval**										**[-0.73; 1.70]**

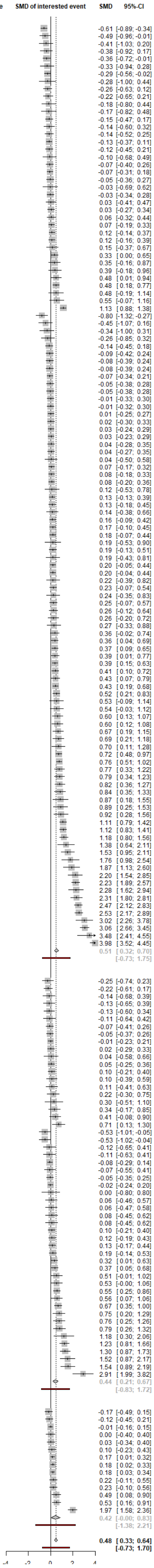

Figure S5.2. Forest plot represents the difference between the intervention vs. control group in the Global QoL domain with the type subgroups as predicted at week 12 (post-intervention). SMD - Standardized mean difference, CI - confidence interval.

Figure S5.3.T24

Study	Experimental			Control			follow-up	Follow-up time	SMD	95%-CI
	Patient N	Mean	SD	Patient N	Mean	SD				
individual										
Li, X, et al., 2017	102	56.32	7.80	108	62.38	11.46	baseline	0.00	-0.61	[-0.89; -0.34]
Garssen B, et al., 2012	34	74.40	4.20	36	76.50	4.30	baseline	0.00	-0.49	[-0.96; -0.01]
Rodin, G, et al., 2019	22	103.92	6.45	20	106.71	6.80	baseline	0.00	-0.41	[-1.03; 0.20]
Seliniotaki, T, et al., 2021	27	62.00	33.80	26	72.20	16.20	baseline	0.00	-0.38	[-0.92; 0.17]
Zhao,X, et al. 2015	62	7.85	2.52	62	8.75	2.38	baseline	0.00	-0.36	[-0.72; -0.01]
Serfaty M, et al., 2018	20	56.40	15.50	22	61.40	14.40	baseline	0.00	-0.33	[-0.94; 0.28]
Fann, JR, et al., 2009	112	5.39	0.21	103	5.45	0.20	baseline	0.00	-0.29	[-0.56; -0.02]
Elyasi, F, et al., 2021 I.	15	3.30	1.90	15	3.80	1.50	baseline	0.00	-0.28	[-1.00; 0.44]
Walczak, A, et al.,2017	61	71.84	16.33	49	75.99	15.72	baseline	0.00	-0.26	[-0.63; 0.12]
Chu, X. et al, 2020	42	65.20	16.77	42	68.91	16.63	baseline	0.00	-0.22	[-0.65; 0.21]
Elyasi, F, et al., 2021 II.	20	3.50	1.80	20	3.80	1.50	baseline	0.00	-0.18	[-0.80; 0.44]
Savard, J, et al., 2006	21	42.51	3.19	16	43.11	3.91	baseline	0.00	-0.17	[-0.82; 0.48]
Nápoles AM, et al. 2015	76	66.46	16.92	75	68.83	15.33	baseline	0.00	-0.15	[-0.47; 0.17]
Dirksen, S. et al, 2007	34	83.30	11.90	38	84.80	9.20	baseline	0.00	-0.14	[-0.60; 0.32]
Zhao, X, et al., 2021	52	53.70	3.14	51	54.69	9.61	baseline	0.00	-0.14	[-0.52; 0.25]
Qiu, H, et al., 2018 II.	98	76.78	19.91	196	79.49	21.60	baseline	0.00	-0.13	[-0.37; 0.11]
Sandsund, C, et al., 2017	72	64.20	21.80	70	66.70	19.70	baseline	0.00	-0.12	[-0.45; 0.21]
Hall S ,et al., 2011 I.	22	12.32	4.35	23	12.74	4.26	baseline	0.00	-0.10	[-0.68; 0.49]
Reese, JB, et al., 2021	73	60.80	16.70	71	61.90	14.30	baseline	0.00	-0.07	[-0.40; 0.26]
Qiu, H, et al., 2018 I.	98	78.09	18.86	196	79.49	21.60	baseline	0.00	-0.07	[-0.31; 0.18]
Chan, et al. 2005	80	58.29	50.71	75	60.44	40.96	baseline	0.00	-0.05	[-0.36; 0.27]
Marchioro, G, et al. 1996	18	-58.44	22.39	18	-57.72	20.25	baseline	0.00	-0.03	[-0.69; 0.62]
Breitbart, W, et al. 2018 II.	86	6.19	1.60	74	6.24	1.60	baseline	0.00	-0.03	[-0.34; 0.28]
Gao Q, et al. 2020	40	-68.20	8.25	40	-68.44	8.82	baseline	0.00	0.03	[-0.41; 0.47]
Breitbart, W, et al. 2018 I.	94	6.29	1.50	74	6.24	1.60	baseline	0.00	0.03	[-0.27; 0.34]
Kissane, DW, et al., 2023	55	25.80	8.30	52	25.30	8.90	baseline	0.00	0.06	[-0.32; 0.44]
Girgis, A, et al., 2009 I.	110	73.20	19.60	117	71.90	17.60	baseline	0.00	0.07	[-0.19; 0.33]
Girgis, A, et al., 2009 II.	120	74.00	18.20	117	71.90	17.60	baseline	0.00	0.12	[-0.14; 0.37]
Klinkhammer-Schalke, M et al., 2012	100	54.35	26.09	100	51.45	23.19	baseline	0.00	0.12	[-0.16; 0.39]
Powell, CB, et al., 2008	21	78.30	17.70	43	75.60	17.90	baseline	0.00	0.15	[-0.37; 0.67]
Thomas, ML, et al., 2012	64	68.80	15.90	88	63.60	15.60	baseline	0.00	0.33	[0.00; 0.65]
Cheung YL, et al. 2002	29	4.96	1.74	30	4.36	1.62	baseline	0.00	0.35	[-0.16; 0.87]
Peoples, AR, et al., 2016	24	78.90	15.30	24	71.90	19.70	baseline	0.00	0.39	[-0.18; 0.96]
Gaston-Johansson, F, et al., 2011	38	22.60	4.30	35	20.60	4.00	baseline	0.00	0.48	[0.01; 0.94]
van der Meulen, IC, et al., 2013	88	67.30	2.30	91	66.20	2.30	baseline	0.00	0.48	[0.18; 0.77]
Northouse, LL, et al., 2015	17	81.30	3.88	19	79.63	2.96	baseline	0.00	0.48	[-0.19; 1.14]
Ham, K, et al. 2019	21	47.69	18.42	21	38.22	15.47	baseline	0.00	0.55	[-0.07; 1.16]
Lu, Z, et al., 201	203	61.70	4.00	103	56.30	6.00	baseline	0.00	1.13	[0.88; 1.38]
Beatty, L. et al. 2015	30	56.22	3.88	30	59.37	3.92	followup	6.00	-0.80	[-1.32; -0.27]
Serfaty M, et al., 2018	20	63.80	19.80	22	72.20	16.60	followup	12.00	-0.45	[-1.07; 0.16]
Savard, J, et al., 2006	21	3.62	0.95	16	3.95	0.91	followup	8.00	-0.34	[-1.00; 0.31]
Hall S ,et al., 2011 I.	22	12.50	4.99	23	13.70	3.89	followup	4.00	-0.26	[-0.85; 0.32]
Chan, et al. 2005	80	60.31	41.32	75	66.46	47.98	followup	12.00	-0.14	[-0.45; 0.18]
Sandsund, C, et al., 2017	72	67.50	22.60	70	69.40	21.10	followup	24.00	-0.09	[-0.42; 0.24]
Chan, et al. 2005	80	71.69	74.33	75	77.23	63.91	followup	72.00	-0.08	[-0.39; 0.24]
Chan, et al. 2005	80	71.61	51.52	75	75.69	53.26	followup	60.00	-0.08	[-0.39; 0.24]
Klinkhammer-Schalke, M et al., 2012	100	53.62	22.46	100	55.07	20.29	followup	12.00	-0.07	[-0.34; 0.21]
Sandsund, C, et al., 2017	72	66.20	19.10	70	67.30	22.00	followup	12.00	-0.05	[-0.38; 0.28]
Reese, JB, et al., 2021	73	61.80	18.20	71	62.60	13.90	followup	8.00	-0.05	[-0.38; 0.28]
Chan, et al. 2005	80	73.13	34.88	75	73.75	47.11	followup	36.00	-0.01	[-0.33; 0.30]
Breitbart, W, et al. 2018 II.	86	6.47	1.20	74	6.48	1.60	followup	4.00	-0.01	[-0.32; 0.30]
Northhouse, LL, et al., 2007	107	87.00	10.80	121	86.90	10.60	followup	32.00	0.01	[-0.25; 0.27]
Chan, et al. 2005	80	67.38	46.78	75	66.55	51.44	followup	24.00	0.02	[-0.30; 0.33]
Northhouse, LL, et al., 2007	104	86.10	10.90	114	85.80	10.70	followup	48.00	0.03	[-0.24; 0.29]
Girgis, A, et al., 2009 I.	110	79.20	19.20	117	78.60	16.70	followup	24.00	0.03	[-0.23; 0.29]
Chan, et al. 2005	80	77.86	49.64	75	76.04	45.64	followup	48.00	0.04	[-0.28; 0.35]
Breitbart, W, et al. 2018 II.	86	6.55	1.60	74	6.49	1.50	followup	8.00	0.04	[-0.27; 0.35]
Seliniotaki, T, et al., 2021	27	65.00	28.00	26	64.00	20.50	followup	8.00	0.04	[-0.50; 0.58]
Qiu, H, et al., 2018 II.	98	80.28	26.77	196	78.03	31.55	followup	4.00	0.07	[-0.17; 0.32]
Girgis, A, et al., 2009 II.	120	79.90	17.40	117	78.60	16.70	followup	24.00	0.08	[-0.18; 0.33]
Klinkhammer-Schalke, M et al., 2012	100	64.13	23.56	100	62.32	21.38	followup	48.00	0.08	[-0.20; 0.36]
Marchioro, G, et al. 1996	18	-54.17	16.90	18	-56.33	17.72	followup	4.00	0.12	[-0.53; 0.78]
Girgis, A, et al., 2009 I.	110	78.30	19.40	117	76.00	16.30	followup	12.00	0.13	[-0.13; 0.39]
Breitbart, W, et al. 2018 II.	86	6.65	1.60	74	6.44	1.50	followup	16.00	0.13	[-0.18; 0.45]
Powell, CB, et al., 2008	21	80.40	26.30	43	77.10	21.20	followup	12.00	0.14	[-0.38; 0.66]
Northhouse, LL, et al., 2007	112	87.20	10.60	123	85.50	10.30	followup	16.00	0.16	[-0.09; 0.42]
Klinkhammer-Schalke, M et al., 2012	100	65.21	22.09	100	61.23	23.55	followup	36.00	0.17	[-0.10; 0.45]
Girgis, A, et al., 2009 II.	120	79.00	16.40	117	76.00	16.30	followup	12.00	0.18	[-0.07; 0.44]
Elyasi, F, et al., 2021 I.	15	4.30	1.00	15	4.10	1.10	followup	24.00	0.19	[-0.53; 0.90]
Nápoles AM, et al. 2015	76	77.24	15.13	75	74.39	15.34	followup	12.00	0.19	[-0.13; 0.51]
Elyasi, F, et al., 2021 II.	20	4.30	1.00	20	4.10	1.10	followup	24.00	0.19	[-0.43; 0.81]
Qiu, H, et al., 2018 II.	98	89.52	33.46	196	83.21	31.55	followup	12.00	0.20	[-0.05; 0.44]
Qiu, H, et al., 2018 II.	98	94.70	35.69	196	87.72	34.70	followup	24.00	0.20	[-0.04; 0.44]
Serfaty M, et al., 2018	20	67.70	23.80	22	63.20	16.60	followup	24.00	0.22	[-0.39; 0.82]
Breitbart, W, et al. 2018 I.	94	6.87	1.70	74	6.48	1.60	followup	4.00	0.23	[-0.07; 0.54]
Hall S ,et al., 2011 I.	22	13.17	4.75	23	12.00	4.85	followup	1.00	0.24	[-0.35; 0.83]
Nápoles AM, et al. 2015	76	80.64	13.64	75	77.02	15.62	followup	24.00	0.25	[-0.07; 0.57]
Kissane, DW, et al., 2023	55	27.40	7.70	52	25.40	7.80	followup	24.00	0.26	[-0.12; 0.64]
Dirksen, S. et al, 2007	34	91.60	15.00	38	87.70	14.70	followup	10.00	0.26	[-0.20; 0.72]
Serfaty M, et al., 2018	20	72.90	19.10	22	67.90	16.70	followup	18.00	0.27	[-0.33; 0.88]
Kissane, DW, et al., 2023	55	27.90	8.10	52	24.80	8.90	followup	12.00	0.36	[-0.02; 0.74]
Thomas, ML, et al., 2012	64	70.50	17.30	88	64.40	16.30	followup	12.00	0.36	[0.04; 0.69]
Klinkhammer-Schalke, M et al., 2012	100	63.41	22.11	100	55.07	23.19	followup	24.00	0.37	[0.09; 0.65]
Walczak, A, et al.,2017	61	77.76	18.80	49	70.87	16.33	followup	4.00	0.39	[0.01; 0.77]
Lu, Z, et al., 201	203	64.40	5.00	103	62.30	6.00	followup	9.00	0.39	[0.15; 0.63]
Breitbart, W, et al. 2018 I.	94	7.06	1.50	74	6.44	1.50	followup	16.00	0.41	[0.10; 0.72]
Zhang, J, et al., 2021	61	9.67	3.71	61	8.21	3.02	followup	12.00	0.43	[0.07; 0.79]
Qiu, H, et al., 2018 I.	98	91.32	29.00	196	78.03	31.55	followup	4.00	0.43	[0.19; 0.68]
Breitbart, W, et al. 2018 I.	94	7.33	1.70	74	6.49	1.50	followup	8.00	0.52	[0.21; 0.83]
Ham, K, et al. 2019	21	54.69	19.80	21	45.02	16.05	followup	10.00	0.53	[-0.09; 1.14]
Peoples, AR, et al., 2016	24	87.90	13.90	24	79.70	15.70	followup	7.00	0.54	[-0.03; 1.12]
Gaston-Johansson, F, et al., 2011	38	24.70	4.30	35	21.90	5.00	followup	48.00	0.60	[0.13; 1.07]
Garssen B, et al., 2012	34	78.40	3.70	36	76.40	2.90	followup	5.00	0.60	[0.12; 1.08]
Garssen B, et al., 2012	34	80.40	3.30	36	78.20	3.20	followup	2.00	0.67	[0.19; 1.15]
Garssen B, et al., 2012	34	75.50	3.60	36	72.90	3.80	followup	13.00	0.69	[0.21; 1.18]
Peoples, AR, et al., 2016	24	91.40	15.00	24	78.60	20.60	followup	12.00	0.70	[0.11; 1.28]
Qiu, H, et al., 2018 I.	98	113.18	35.69	196	87.72	34.70	followup	24.00	0.72	[0.48; 0.97]
Qiu, H, et al., 2018 I.	98	107.32	31.23	196	83.21	31.55	followup	12.00	0.76	[0.51; 1.02]
Chu, X. et al, 2020	42	85.41	16.08	42	71.90	18.50	followup	12.00	0.77	[0.33; 1.22]
Chu, X. et al, 2020	42	84.94	14.59	42	71.59	18.73	followup	8.00	0.79	[0.34; 1.23]
Gao Q, et al. 2020	40	-54.26	7.03	40	-60.17	7.28	followup	4.00	0.82	[0.36; 1.27]
Garssen B, et al., 2012	34	81.00	3.40	36	78.20	3.20	followup	4.00	0.84	[0.35; 1.33]
Marchioro, G, et al. 1996	18	-48.72	12.22	18	-60.78	14.85	followup	12.00	0.87	[0.18; 1.55]
Rodin, G, et al., 2019	22	110.14	6.83	20	103.57	7.71	followup	4.00	0.89	[0.25; 1.53]
Rodin, G, et al., 2019	22	110.41	7.29	20	103.70	7.00	followup	8.00	0.92	[0.28; 1.56]
van der Meulen, IC, et al., 2013	88	77.50	2.80	91	74.50	2.60	followup	48.00	1.11	[0.79; 1.42]
Li, X, et al., 2017	102	76.58	9.06	108	64.28	12.49	followup	12.00	1.12	[0.83; 1.41]
Zhao,X, et al. 2015	62	10.65	2.02	62	8.22	2.07	followup	0.30	1.18	[0.80; 1.56]
Marchioro, G, et al. 1996	18	-44.72	9.18	18	-60.78	13.26	followup	24.00	1.38	[0.64; 2.11]
Beatty, L. et al. 2015	30	68.50	3.64	30	62.85	3.64	followup	13.03	1.53	[0.95; 2.11]
Marchioro, G, et al. 1996	18	-41.17	6.91	18	-60.28	13.33	followup	36.00	1.76	[0.98; 2.54]
Rodin, G, et al., 2019	22	114.37	6.97	20	100.61	7.50	followup	12.00	1.87	[1.13; 2.60]
Cheung YL, et al. 2002	29	7.48	1.12	30	5.56	0.50	followup	10.00	2.20	[1.54; 2.85]
Fann, JR, et al., 2009	112	6.30	0.25	103	5.74	0.25	followup	24.00	2.23	[1.89; 2.57]
Beatty, L. et al. 2015	30	74.04	3.78	30	65.27	3.81	followup	26.07	2.28	[1.62; 2.94]
Zhao, X, et al., 2021	52	66.68	3.21	51	55.07	6.32	followup	12.00	2.31	[1.80; 2.81]
Fann, JR, et al., 2009	112	6.51	0.25	103	5.84	0.29	followup	96.00	2.47	[2.12; 2.83]
Fann, JR, et al., 2009	112	6.33	0.22	103	5.72	0.26	followup	12.00	2.53	[2.17; 2.89]
Cheung YL, et al. 2002	29	7.51	0.98	30	4.96	0.66	followup	5.00	3.02	[2.26; 3.78]
Fann, JR, et al., 2009	112	6.67	0.23	103	5.95	0.24	followup	48.00	3.06	[2.66; 3.45]
Northouse, LL, et al., 2015	17	88.51	3.03	19	76.94	3.44	followup	2.00	3.48	[2.41; 4.55]
Fann, JR, et al., 2009	112	6.33	0.25	103	5.35	0.24	followup	72.00	3.98	[3.52; 4.45]
Random effect	7706			8192					0.46	[0.11; 0.80]
Prediction interval										[-0.85; 1.77]
group										
Heiney, SP, et al., 2003	33	6.00	1.70	33	6.40	1.40	baseline	0.00	-0.25	[-0.74; 0.23]
Liu, T, et al., 2019	49	66.61	10.85	53	69.54	15.27	baseline	0.00	-0.22	[-0.61; 0.17]
Lee, JT, et al., 2022	29	3.50	0.60	25	3.60	0.80	baseline	0.00	-0.14	[-0.68; 0.39]
Peng, L, et al., 2022	28	69.05	16.49	29	71.26	17.05	baseline	0.00	-0.13	[-0.65; 0.39]
Penedo, FJ, et al., 2007	41	-82.35	14.02	30	-80.57	13.21	baseline	0.00	-0.13	[-0.60; 0.34]
Von Ah, D, et al.,2012 I.	26	22.06	3.44	29	22.38	2.24	baseline	0.00	-0.11	[-0.64; 0.42]
Schellekens, MPJ, et al. 2016	69	95.43	21.60	70	97.09	23.54	baseline	0.00	-0.07	[-0.41; 0.26]
Compen F, et al. 2020 I.	77	0.75	0.21	78	0.76	0.17	baseline	0.00	-0.05	[-0.37; 0.26]
Reich, RR, et al. 2016	167	62.44	27.52	155	62.74	24.68	baseline	0.00	-0.01	[-0.23; 0.21]
Xia, S, et al., 2023	80	61.40	16.60	80	61.10	15.70	baseline	0.00	0.02	[-0.29; 0.33]
Nikbakhsh, N, et al., 2018	20	44.09	8.48	20	43.64	13.73	baseline	0.00	0.04	[-0.58; 0.66]
Compen F, et al. 2020 II.	90	0.77	0.19	78	0.76	0.17	baseline	0.00	0.05	[-0.25; 0.36]
Esplen MJ, et al., 2018	131	70.50	16.50	63	68.90	17.20	baseline	0.00	0.10	[-0.21; 0.40]
Mihuta, ME, et al., 2018	32	11.90	4.40	33	11.50	3.60	baseline	0.00	0.10	[-0.39; 0.59]
Hernandez, EG, et al. 2018	28	64.86	20.04	28	62.85	15.86	baseline	0.00	0.11	[-0.41; 0.63]
Von Ah, D, et al.,2012 II.	27	22.98	3.08	29	22.38	2.24	baseline	0.00	0.22	[-0.30; 0.75]
Rahmani, S, et al. 2015	12	41.66	6.15	12	39.58	7.22	baseline	0.00	0.30	[-0.51; 1.10]
Yoo, HJ, et al. 2004	30	93.41	16.90	30	87.88	14.96	baseline	0.00	0.34	[-0.17; 0.85]
Cengiz, HO, et al., 2023	32	63.06	19.74	33	55.22	18.26	baseline	0.00	0.41	[-0.08; 0.90]
Jelvehzadeh, F, et al., 2022	24	4.71	1.07	24	3.94	1.06	baseline	0.00	0.71	[0.13; 1.30]
Penedo, FJ, et al., 2007	41	-87.22	13.82	30	-79.63	14.56	followup	12.00	-0.53	[-1.01; -0.05]
Heiney, SP, et al., 2003	33	6.00	1.50	33	6.80	1.50	followup	16.00	-0.53	[-1.02; -0.04]
Von Ah, D, et al.,2012 I.	26	22.15	4.45	29	22.58	2.24	followup	8.00	-0.12	[-0.65; 0.41]
Peng, L, et al., 2022	28	66.67	24.00	29	69.25	22.17	followup	4.00	-0.11	[-0.63; 0.41]
Reich, RR, et al. 2016	167	68.43	27.76	155	70.36	22.70	followup	12.00	-0.08	[-0.29; 0.14]
Heiney, SP, et al., 2003	33	6.20	1.30	33	6.30	1.60	followup	6.00	-0.07	[-0.55; 0.41]
Esplen MJ, et al., 2018	131	73.40	16.70	63	74.30	17.70	followup	48.00	-0.05	[-0.35; 0.25]
Reich, RR, et al. 2016	167	65.76	26.18	155	66.24	24.76	followup	6.00	-0.02	[-0.24; 0.20]
Rahmani, S, et al. 2015	12	12.00	61.11	12	12.00	58.33	followup	16.00	0.00	[-0.80; 0.80]
Peng, L, et al., 2022	28	72.32	22.11	29	71.26	15.26	followup	0.10	0.06	[-0.46; 0.57]
Von Ah, D, et al.,2012 II.	27	22.71	2.08	29	22.58	2.24	followup	8.00	0.06	[-0.47; 0.58]
Lee, JT, et al., 2022	29	4.05	0.60	25	4.00	0.60	followup	12.00	0.08	[-0.45; 0.62]
Lee, JT, et al., 2022	29	4.05	0.60	25	4.00	0.60	followup	24.00	0.08	[-0.45; 0.62]
Esplen MJ, et al., 2018	131	14.90	9.40	63	14.00	9.40	followup	8.00	0.10	[-0.21; 0.40]
Xia, S, et al., 2023	80	67.70	16.20	80	65.80	14.40	followup	4.00	0.12	[-0.19; 0.43]
Esplen MJ, et al., 2018	131	72.60	16.60	63	70.30	18.00	followup	24.00	0.13	[-0.17; 0.44]
Schellekens, MPJ, et al. 2016	69	107.36	18.80	70	103.34	22.35	followup	8.00	0.19	[-0.14; 0.53]
Xia, S, et al., 2023	80	72.70	15.80	80	68.00	13.50	followup	12.00	0.32	[0.01; 0.63]
Xia, S, et al., 2023	80	78.00	14.30	80	72.90	13.40	followup	24.00	0.37	[0.05; 0.68]
Yoo, HJ, et al. 2004	30	97.01	18.24	30	88.13	16.18	followup	12.00	0.51	[-0.01; 1.02]
Hernandez, EG, et al. 2018	28	71.73	15.45	28	62.80	17.63	followup	24.00	0.53	[-0.00; 1.06]
Compen F, et al. 2020 II.	90	0.85	0.17	78	0.75	0.19	followup	8.00	0.55	[0.25; 0.86]
Mihuta, ME, et al., 2018	32	14.10	2.60	33	12.30	3.60	followup	4.00	0.56	[0.07; 1.06]
Compen F, et al. 2020 I.	77	0.86	0.13	78	0.75	0.19	followup	8.00	0.67	[0.35; 1.00]
Hernandez, EG, et al. 2018	28	74.38	11.48	28	64.40	14.73	followup	8.00	0.75	[0.20; 1.29]
Cengiz, HO, et al., 2023	32	66.98	17.72	33	53.03	18.65	followup	8.00	0.76	[0.25; 1.26]
Yoo, HJ, et al. 2004	30	102.00	19.94	30	88.18	14.02	followup	24.00	0.79	[0.26; 1.32]
Rahmani, S, et al. 2015	12	66.66	9.40	12	54.16	10.95	followup	8.00	1.18	[0.30; 2.06]
Liu, T, et al., 2019	49	62.67	10.32	53	49.47	10.92	followup	9.00	1.23	[0.81; 1.66]
Liu, T, et al., 2019	49	72.33	13.36	53	56.95	10.03	followup	12.00	1.30	[0.87; 1.73]
Jelvehzadeh, F, et al., 2022	24	6.05	1.00	24	4.65	0.80	followup	12.00	1.52	[0.87; 2.17]
Jelvehzadeh, F, et al., 2022	24	6.16	1.09	24	4.58	0.92	followup	8.00	1.54	[0.89; 2.19]
Nikbakhsh, N, et al., 2018	20	81.70	10.04	20	47.39	12.93	followup	12.00	2.91	[1.99; 3.82]
Random effect	2872			2539					0.39	[0.06; 0.72]
Prediction interval										[-0.90; 1.68]
self-help										
van den Berg, SW, et al. 2015 II.	70	66.79	16.57	80	69.79	17.91	baseline	0.00	-0.17	[-0.49; 0.15]
Wang, TJ, et al 2023	70	102.30	18.40	72	104.40	17.10	baseline	0.00	-0.12	[-0.45; 0.21]
van der Hout, AVD, et al. 2019	320	85.30	14.90	304	85.40	13.60	baseline	0.00	-0.01	[-0.16; 0.15]
Walker, LG, et al.,1998	48	3.25	0.70	48	3.25	0.66	baseline	0.00	0.00	[-0.40; 0.40]
Rosen, KD, et al., 2018	57	93.61	22.68	55	92.96	25.12	baseline	0.00	0.03	[-0.34; 0.40]
Wang, TJ, et al 2023	70	105.40	17.80	72	103.50	20.00	followup	8.00	0.10	[-0.23; 0.43]
van der Hout, AVD, et al. 2019	320	88.70	13.20	304	86.50	13.10	followup	12.00	0.17	[0.01; 0.32]
van der Hout, AVD, et al. 2019	320	88.40	12.10	304	86.20	12.80	followup	1.00	0.18	[0.02; 0.33]
van der Hout, AVD, et al. 2019	320	89.30	12.30	304	87.00	12.70	followup	24.00	0.18	[0.03; 0.34]
Wang, TJ, et al 2023	70	107.30	16.70	72	103.30	19.00	followup	16.00	0.22	[-0.11; 0.55]
Wang, TJ, et al 2023	70	106.90	16.60	72	102.70	20.00	followup	24.00	0.23	[-0.10; 0.56]
Walker, LG, et al.,1998	48	3.29	0.80	48	2.90	0.78	followup	15.00	0.49	[0.08; 0.90]
Rosen, KD, et al., 2018	57	107.35	17.79	55	96.04	23.96	followup	5.00	0.53	[0.16; 0.91]
van den Berg, SW, et al. 2015 II.	70	73.30	1.78	80	69.88	1.67	followup	4.00	1.97	[1.58; 2.36]
Random effect	1910			1870					0.36	[-0.11; 0.83]
Prediction interval										[-1.29; 2.02]
**Random effect**	**12488**			**12601**					**0.44**	**[0.12; 0.75]**
**Prediction interval**										**[-0.86; 1.73]**

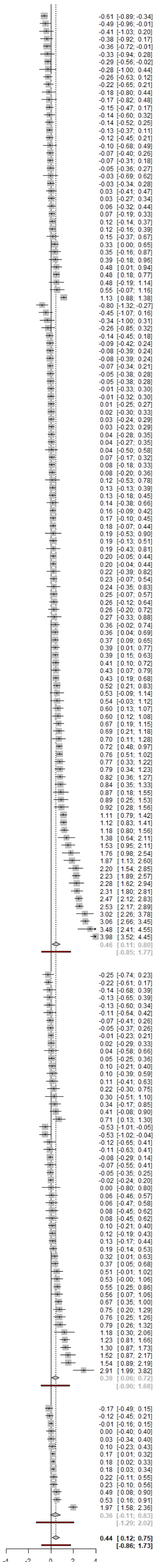

Figure S5.3. Forest plot represents the difference between the intervention vs. control group in the Global QoL domain with the type subgroups as predicted at week 24 (post-intervention). SMD - Standardized mean difference, CI - confidence interval.

Figure S5.4.T48

Study	Experimental Patient N	Experimental Mean	Experimental SD	Control Patient N	Control Mean	Control SD	follow-up	Follow-up time	SMD	95%-CI
**individual**										
Li, X, et al., 2017	102	56.32	7.80	108	62.38	11.46	baseline	0.00	-0.61	[-0.89; -0.34]
Garssen B, et al., 2012	34	74.40	4.20	36	76.50	4.30	baseline	0.00	-0.49	[-0.96; -0.01]
Rodin, G, et al., 2019	22	103.92	6.45	20	106.71	6.80	baseline	0.00	-0.41	[-1.03; 0.20]
Seliniotaki, T, et al., 2021	27	62.00	33.80	26	72.20	16.20	baseline	0.00	-0.38	[-0.92; 0.17]
Zhao,X, et al. 2015	62	7.85	2.52	62	8.75	2.38	baseline	0.00	-0.36	[-0.72; -0.01]
Serfaty M, et al., 2018	20	56.40	15.50	22	61.40	14.40	baseline	0.00	-0.33	[-0.94; 0.28]
Fann, JR, et al., 2009	112	5.39	0.21	103	5.45	0.20	baseline	0.00	-0.29	[-0.56; -0.02]
Elyasi, F, et al., 2021 I.	15	3.30	1.90	15	3.80	1.50	baseline	0.00	-0.28	[-1.00; 0.44]
Walczak, A, et al.,2017	61	71.84	16.33	49	75.99	15.72	baseline	0.00	-0.26	[-0.63; 0.12]
Chu, X. et al, 2020	42	65.20	16.77	42	68.91	16.63	baseline	0.00	-0.22	[-0.65; 0.21]
Elyasi, F, et al., 2021 II.	20	3.50	1.80	20	3.80	1.50	baseline	0.00	-0.18	[-0.80; 0.44]
Savard, J, et al., 2006	21	42.51	3.19	16	43.11	3.91	baseline	0.00	-0.17	[-0.82; 0.48]
Nápoles AM, et al. 2015	76	66.46	16.92	75	68.83	15.33	baseline	0.00	-0.15	[-0.47; 0.17]
Dirksen, S. et al, 2007	34	83.30	11.90	38	84.80	9.20	baseline	0.00	-0.14	[-0.60; 0.32]
Zhao, X, et al., 2021	52	53.70	3.14	51	54.69	9.61	baseline	0.00	-0.14	[-0.52; 0.25]
Qiu, H, et al., 2018 II.	98	76.78	19.91	196	79.49	21.60	baseline	0.00	-0.13	[-0.37; 0.11]
Sandsund, C, et al., 2017	72	64.20	21.80	70	66.70	19.70	baseline	0.00	-0.12	[-0.45; 0.21]
Hall S ,et al., 2011 I.	22	12.32	4.35	23	12.74	4.26	baseline	0.00	-0.10	[-0.68; 0.49]
Reese, JB, et al., 2021	73	60.80	16.70	71	61.90	14.30	baseline	0.00	-0.07	[-0.40; 0.26]
Qiu, H, et al., 2018 I.	98	78.09	18.86	196	79.49	21.60	baseline	0.00	-0.07	[-0.31; 0.18]
Chan, et al. 2005	80	58.29	50.71	75	60.44	40.96	baseline	0.00	-0.05	[-0.36; 0.27]
Marchioro, G, et al. 1996	18	-58.44	22.39	18	-57.72	20.25	baseline	0.00	-0.03	[-0.69; 0.62]
Breitbart, W, et al. 2018 II.	86	6.19	1.60	74	6.24	1.60	baseline	0.00	-0.03	[-0.34; 0.28]
Gao Q, et al. 2020	40	-68.20	8.25	40	-68.44	8.82	baseline	0.00	0.03	[-0.41; 0.47]
Breitbart, W, et al. 2018 I.	94	6.29	1.50	74	6.24	1.60	baseline	0.00	0.03	[-0.27; 0.34]
Kissane, DW, et al., 2023	55	25.80	8.30	52	25.30	8.90	baseline	0.00	0.06	[-0.32; 0.44]
Girgis, A, et al., 2009 I.	110	73.20	19.60	117	71.90	17.60	baseline	0.00	0.07	[-0.19; 0.33]
Girgis, A, et al., 2009 II.	120	74.00	18.20	117	71.90	17.60	baseline	0.00	0.12	[-0.14; 0.37]
Klinkhammer-Schalke, M et al., 2012	100	54.35	26.09	100	51.45	23.19	baseline	0.00	0.12	[-0.16; 0.39]
Powell, CB, et al., 2008	21	78.30	17.70	43	75.60	17.90	baseline	0.00	0.15	[-0.37; 0.67]
Thomas, ML, et al., 2012	64	68.80	15.90	88	63.60	15.60	baseline	0.00	0.33	[0.00; 0.65]
Cheung YL, et al. 2002	29	4.96	1.74	30	4.36	1.62	baseline	0.00	0.35	[-0.16; 0.87]
Peoples, AR, et al., 2016	24	78.90	15.30	24	71.90	19.70	baseline	0.00	0.39	[-0.18; 0.96]
Gaston-Johansson, F, et al., 2011	38	22.60	4.30	35	20.60	4.00	baseline	0.00	0.48	[0.01; 0.94]
van der Meulen, IC, et al., 2013	88	67.30	2.30	91	66.20	2.30	baseline	0.00	0.48	[0.18; 0.77]
Northouse, LL, et al., 2015	17	81.30	3.88	19	79.63	2.96	baseline	0.00	0.48	[-0.19; 1.14]
Ham, K, et al. 2019	21	47.69	18.42	21	38.22	15.47	baseline	0.00	0.55	[-0.07; 1.16]
Lu, Z, et al., 201	203	61.70	4.00	103	56.30	6.00	baseline	0.00	1.13	[0.88; 1.38]
Beatty, L. et al. 2015	30	56.22	3.88	30	59.37	3.92	followup	6.00	-0.80	[-1.32; -0.27]
Serfaty M, et al., 2018	20	63.80	19.80	22	72.20	16.60	followup	12.00	-0.45	[-1.07; 0.16]
Savard, J, et al., 2006	21	3.62	0.95	16	3.95	0.91	followup	8.00	-0.34	[-1.00; 0.31]
Hall S ,et al., 2011 I.	22	12.50	4.99	23	13.70	3.89	followup	4.00	-0.26	[-0.85; 0.32]
Chan, et al. 2005	80	60.31	41.32	75	66.46	47.98	followup	12.00	-0.14	[-0.45; 0.18]
Sandsund, C, et al., 2017	72	67.50	22.60	70	69.40	21.10	followup	24.00	-0.09	[-0.42; 0.24]
Chan, et al. 2005	80	71.69	74.33	75	77.23	63.91	followup	72.00	-0.08	[-0.39; 0.24]
Chan, et al. 2005	80	71.61	51.52	75	75.69	53.26	followup	60.00	-0.08	[-0.39; 0.24]
Klinkhammer-Schalke, M et al., 2012	100	53.62	22.46	100	55.07	20.29	followup	12.00	-0.07	[-0.34; 0.21]
Sandsund, C, et al., 2017	72	66.20	19.10	70	67.30	22.00	followup	12.00	-0.05	[-0.38; 0.28]
Reese, JB, et al., 2021	73	61.80	18.20	71	62.60	13.90	followup	8.00	-0.05	[-0.38; 0.28]
Chan, et al. 2005	80	73.13	34.88	75	73.75	47.11	followup	36.00	-0.01	[-0.33; 0.30]
Breitbart, W, et al. 2018 II.	86	6.47	1.20	74	6.48	1.60	followup	4.00	-0.01	[-0.32; 0.30]
Northhouse, LL, et al., 2007	107	87.00	10.80	121	86.90	10.60	followup	32.00	0.01	[-0.25; 0.27]
Chan, et al. 2005	80	67.38	46.78	75	66.55	51.44	followup	24.00	0.02	[-0.30; 0.33]
Northhouse, LL, et al., 2007	104	86.10	10.90	114	85.80	10.70	followup	48.00	0.03	[-0.24; 0.29]
Girgis, A, et al., 2009 I.	110	79.20	19.20	117	78.60	16.70	followup	24.00	0.03	[-0.23; 0.29]
Chan, et al. 2005	80	77.86	49.64	75	76.04	45.64	followup	48.00	0.04	[-0.28; 0.35]
Breitbart, W, et al. 2018 II.	86	6.55	1.60	74	6.49	1.50	followup	8.00	0.04	[-0.27; 0.35]
Seliniotaki, T, et al., 2021	27	65.00	28.00	26	64.00	20.50	followup	8.00	0.04	[-0.50; 0.58]
Qiu, H, et al., 2018 II.	98	80.28	26.77	196	78.03	31.55	followup	4.00	0.07	[-0.17; 0.32]
Girgis, A, et al., 2009 II.	120	79.90	17.40	117	78.60	16.70	followup	24.00	0.08	[-0.18; 0.33]
Klinkhammer-Schalke, M et al., 2012	100	64.13	23.56	100	62.32	21.38	followup	48.00	0.08	[-0.20; 0.36]
Marchioro, G, et al. 1996	18	-54.17	16.90	18	-56.33	17.72	followup	4.00	0.12	[-0.53; 0.78]
Girgis, A, et al., 2009 I.	110	78.30	19.40	117	76.00	16.30	followup	12.00	0.13	[-0.13; 0.39]
Breitbart, W, et al. 2018 II.	86	6.65	1.60	74	6.44	1.50	followup	16.00	0.13	[-0.18; 0.45]
Powell, CB, et al., 2008	21	80.40	26.30	43	77.10	21.20	followup	12.00	0.14	[-0.38; 0.66]
Northhouse, LL, et al., 2007	112	87.20	10.60	123	85.50	10.30	followup	16.00	0.16	[-0.09; 0.42]
Klinkhammer-Schalke, M et al., 2012	100	65.21	22.09	100	61.23	23.55	followup	36.00	0.17	[-0.10; 0.45]
Girgis, A, et al., 2009 II.	120	79.00	16.40	117	76.00	16.30	followup	12.00	0.18	[-0.07; 0.44]
Elyasi, F, et al., 2021 I.	15	4.30	1.00	15	4.10	1.10	followup	24.00	0.19	[-0.53; 0.90]
Nápoles AM, et al. 2015	76	77.24	15.13	75	74.39	15.34	followup	12.00	0.19	[-0.13; 0.51]
Elyasi, F, et al., 2021 II.	20	4.30	1.00	20	4.10	1.10	followup	24.00	0.19	[-0.43; 0.81]
Qiu, H, et al., 2018 II.	98	89.52	33.46	196	83.21	31.55	followup	12.00	0.20	[-0.05; 0.44]
Qiu, H, et al., 2018 II.	98	94.70	35.69	196	87.72	34.70	followup	24.00	0.20	[-0.04; 0.44]
Serfaty M, et al., 2018	20	67.70	23.80	22	63.20	16.60	followup	24.00	0.22	[-0.39; 0.82]
Breitbart, W, et al. 2018 I.	94	6.87	1.70	74	6.48	1.60	followup	4.00	0.23	[-0.07; 0.54]
Hall S ,et al., 2011 I.	22	13.17	4.75	23	12.00	4.85	followup	1.00	0.24	[-0.35; 0.83]
Nápoles AM, et al. 2015	76	80.64	13.64	75	77.02	15.62	followup	24.00	0.25	[-0.07; 0.57]
Kissane, DW, et al., 2023	55	27.40	7.70	52	25.40	7.80	followup	24.00	0.26	[-0.12; 0.64]
Dirksen, S. et al, 2007	34	91.60	15.00	38	87.70	14.70	followup	10.00	0.26	[-0.20; 0.72]
Serfaty M, et al., 2018	20	72.90	19.10	22	67.90	16.70	followup	18.00	0.27	[-0.33; 0.88]
Kissane, DW, et al., 2023	55	27.90	8.10	52	24.80	8.90	followup	12.00	0.36	[-0.02; 0.74]
Thomas, ML, et al., 2012	64	70.50	17.30	88	64.40	16.30	followup	12.00	0.36	[0.04; 0.69]
Klinkhammer-Schalke, M et al., 2012	100	63.41	22.11	100	55.07	23.19	followup	24.00	0.37	[0.09; 0.65]
Walczak, A, et al.,2017	61	77.76	18.80	49	70.87	16.33	followup	4.00	0.39	[0.01; 0.77]
Lu, Z, et al., 201	203	64.40	5.00	103	62.30	6.00	followup	9.00	0.39	[0.15; 0.63]
Breitbart, W, et al. 2018 I.	94	7.06	1.50	74	6.44	1.50	followup	16.00	0.41	[0.10; 0.72]
Zhang, J, et al., 2021	61	9.67	3.71	61	8.21	3.02	followup	12.00	0.43	[0.07; 0.79]
Qiu, H, et al., 2018 I.	98	91.32	29.00	196	78.03	31.55	followup	4.00	0.43	[0.19; 0.68]
Breitbart, W, et al. 2018 I.	94	7.33	1.70	74	6.49	1.50	followup	8.00	0.52	[0.21; 0.83]
Ham, K, et al. 2019	21	54.69	19.80	21	45.02	16.05	followup	10.00	0.53	[-0.09; 1.14]
Peoples, AR, et al., 2016	24	87.90	13.90	24	79.70	15.70	followup	7.00	0.54	[-0.03; 1.12]
Gaston-Johansson, F, et al., 2011	38	24.70	4.30	35	21.90	5.00	followup	48.00	0.60	[0.13; 1.07]
Garssen B, et al., 2012	34	78.40	3.70	36	76.40	2.90	followup	5.00	0.60	[0.12; 1.08]
Garssen B, et al., 2012	34	80.40	3.30	36	78.20	3.20	followup	2.00	0.67	[0.19; 1.15]
Garssen B, et al., 2012	34	75.50	3.60	36	72.90	3.80	followup	13.00	0.69	[0.21; 1.18]
Peoples, AR, et al., 2016	24	91.40	15.00	24	78.60	20.60	followup	12.00	0.70	[0.11; 1.28]
Qiu, H, et al., 2018 I.	98	113.18	35.69	196	87.72	34.70	followup	24.00	0.72	[0.48; 0.97]
Qiu, H, et al., 2018 I.	98	107.32	31.23	196	83.21	31.55	followup	12.00	0.76	[0.51; 1.02]
Chu, X. et al, 2020	42	85.41	16.08	42	71.90	18.50	followup	12.00	0.77	[0.33; 1.22]
Chu, X. et al, 2020	42	84.94	14.59	42	71.59	18.73	followup	8.00	0.79	[0.34; 1.23]
Gao Q, et al. 2020	40	-54.26	7.03	40	-60.17	7.28	followup	4.00	0.82	[0.36; 1.27]
Garssen B, et al., 2012	34	81.00	3.40	36	78.20	3.20	followup	4.00	0.84	[0.35; 1.33]
Marchioro, G, et al. 1996	18	-48.72	12.22	18	-60.78	14.85	followup	12.00	0.87	[0.18; 1.55]
Rodin, G, et al., 2019	22	110.14	6.83	20	103.57	7.71	followup	4.00	0.89	[0.25; 1.53]
Rodin, G, et al., 2019	22	110.41	7.29	20	103.70	7.00	followup	8.00	0.92	[0.28; 1.56]
van der Meulen, IC, et al., 2013	88	77.50	2.80	91	74.50	2.60	followup	48.00	1.11	[0.79; 1.42]
Li, X, et al., 2017	102	76.58	9.06	108	64.28	12.49	followup	12.00	1.12	[0.83; 1.41]
Zhao,X, et al. 2015	62	10.65	2.02	62	8.22	2.07	followup	0.30	1.18	[0.80; 1.56]
Marchioro, G, et al. 1996	18	-44.72	9.18	18	-60.78	13.26	followup	24.00	1.38	[0.64; 2.11]
Beatty, L. et al. 2015	30	68.50	3.64	30	62.85	3.64	followup	13.03	1.53	[0.95; 2.11]
Marchioro, G, et al. 1996	18	-41.17	6.91	18	-60.28	13.33	followup	36.00	1.76	[0.98; 2.54]
Rodin, G, et al., 2019	22	114.37	6.97	20	100.61	7.50	followup	12.00	1.87	[1.13; 2.60]
Cheung YL, et al. 2002	29	7.48	1.12	30	5.56	0.50	followup	10.00	2.20	[1.54; 2.85]
Fann, JR, et al., 2009	112	6.30	0.25	103	5.74	0.25	followup	24.00	2.23	[1.89; 2.57]
Beatty, L. et al. 2015	30	74.04	3.78	30	65.27	3.81	followup	26.07	2.28	[1.62; 2.94]
Zhao, X, et al., 2021	52	66.68	3.21	51	55.07	6.32	followup	12.00	2.31	[1.80; 2.81]
Fann, JR, et al., 2009	112	6.51	0.25	103	5.84	0.29	followup	96.00	2.47	[2.12; 2.83]
Fann, JR, et al., 2009	112	6.33	0.22	103	5.72	0.26	followup	12.00	2.53	[2.17; 2.89]
Cheung YL, et al. 2002	29	7.51	0.98	30	4.96	0.66	followup	5.00	3.02	[2.26; 3.78]
Fann, JR, et al., 2009	112	6.67	0.23	103	5.95	0.24	followup	48.00	3.06	[2.66; 3.45]
Northouse, LL, et al., 2015	17	88.51	3.03	19	76.94	3.44	followup	2.00	3.48	[2.41; 4.55]
Fann, JR, et al., 2009	112	6.33	0.25	103	5.35	0.24	followup	72.00	3.98	[3.52; 4.45]
Random effect	7706			8192					0.70	[-0.30; 1.69]
Prediction interval										[-0.99; 2.38]
**group**										
Heiney, SP, et al., 2003	33	6.00	1.70	33	6.40	1.40	baseline	0.00	-0.25	[-0.74; 0.23]
Liu, T, et al., 2019	49	66.61	10.85	53	69.54	15.27	baseline	0.00	-0.22	[-0.61; 0.17]
Lee, JT, et al., 2022	29	3.50	0.60	25	3.60	0.80	baseline	0.00	-0.14	[-0.68; 0.39]
Peng, L, et al., 2022	28	69.05	16.49	29	71.26	17.05	baseline	0.00	-0.13	[-0.65; 0.39]
Penedo, FJ, et al., 2007	41	-82.35	14.02	30	-80.57	13.21	baseline	0.00	-0.13	[-0.60; 0.34]
Von Ah, D, et al.,2012 I.	26	22.06	3.44	29	22.38	2.24	baseline	0.00	-0.11	[-0.64; 0.42]
Schellekens, MPJ, et al. 2016	69	95.43	21.60	70	97.09	23.54	baseline	0.00	-0.07	[-0.41; 0.26]
Compen F, et al. 2020 I.	77	0.75	0.21	78	0.76	0.17	baseline	0.00	-0.05	[-0.37; 0.26]
Reich, RR, et al. 2016	167	62.44	27.52	155	62.74	24.68	baseline	0.00	-0.01	[-0.23; 0.21]
Xia, S, et al., 2023	80	61.40	16.60	80	61.10	15.70	baseline	0.00	0.02	[-0.29; 0.33]
Nikbakhsh, N, et al., 2018	20	44.09	8.48	20	43.64	13.73	baseline	0.00	0.04	[-0.58; 0.66]
Compen F, et al. 2020 II.	90	0.77	0.19	78	0.76	0.17	baseline	0.00	0.05	[-0.25; 0.36]
Esplen MJ, et al., 2018	131	70.50	16.50	63	68.90	17.20	baseline	0.00	0.10	[-0.21; 0.40]
Mihuta, ME, et al., 2018	32	11.90	4.40	33	11.50	3.60	baseline	0.00	0.10	[-0.39; 0.59]
Hernandez, EG, et al. 2018	28	64.86	20.04	28	62.85	15.86	baseline	0.00	0.11	[-0.41; 0.63]
Von Ah, D, et al.,2012 II.	27	22.98	3.08	29	22.38	2.24	baseline	0.00	0.22	[-0.30; 0.75]
Rahmani, S, et al. 2015	12	41.66	6.15	12	39.58	7.22	baseline	0.00	0.30	[-0.51; 1.10]
Yoo, HJ, et al. 2004	30	93.41	16.90	30	87.88	14.96	baseline	0.00	0.34	[-0.17; 0.85]
Cengiz, HO, et al., 2023	32	63.06	19.74	33	55.22	18.26	baseline	0.00	0.41	[-0.08; 0.90]
Jelvehzadeh, F, et al., 2022	24	4.71	1.07	24	3.94	1.06	baseline	0.00	0.71	[0.13; 1.30]
Penedo, FJ, et al., 2007	41	-87.22	13.82	30	-79.63	14.56	followup	12.00	-0.53	[-1.01; -0.05]
Heiney, SP, et al., 2003	33	6.00	1.50	33	6.80	1.50	followup	16.00	-0.53	[-1.02; -0.04]
Von Ah, D, et al.,2012 I.	26	22.15	4.45	29	22.58	2.24	followup	8.00	-0.12	[-0.65; 0.41]
Peng, L, et al., 2022	28	66.67	24.00	29	69.25	22.17	followup	4.00	-0.11	[-0.63; 0.41]
Reich, RR, et al. 2016	167	68.43	27.76	155	70.36	22.70	followup	12.00	-0.08	[-0.29; 0.14]
Heiney, SP, et al., 2003	33	6.20	1.30	33	6.30	1.60	followup	6.00	-0.07	[-0.55; 0.41]
Esplen MJ, et al., 2018	131	73.40	16.70	63	74.30	17.70	followup	48.00	-0.05	[-0.35; 0.25]
Reich, RR, et al. 2016	167	65.76	26.18	155	66.24	24.76	followup	6.00	-0.02	[-0.24; 0.20]
Rahmani, S, et al. 2015	12	12.00	61.11	12	12.00	58.33	followup	16.00	0.00	[-0.80; 0.80]
Peng, L, et al., 2022	28	72.32	22.11	29	71.26	15.26	followup	0.10	0.06	[-0.46; 0.57]
Von Ah, D, et al.,2012 II.	27	22.71	2.08	29	22.58	2.24	followup	8.00	0.06	[-0.47; 0.58]
Lee, JT, et al., 2022	29	4.05	0.60	25	4.00	0.60	followup	12.00	0.08	[-0.45; 0.62]
Lee, JT, et al., 2022	29	4.05	0.60	25	4.00	0.60	followup	24.00	0.08	[-0.45; 0.62]
Esplen MJ, et al., 2018	131	14.90	9.40	63	14.00	9.40	followup	8.00	0.10	[-0.21; 0.40]
Xia, S, et al., 2023	80	67.70	16.20	80	65.80	14.40	followup	4.00	0.12	[-0.19; 0.43]
Esplen MJ, et al., 2018	131	72.60	16.60	63	70.30	18.00	followup	24.00	0.13	[-0.17; 0.44]
Schellekens, MPJ, et al. 2016	69	107.36	18.80	70	103.34	22.35	followup	8.00	0.19	[-0.14; 0.53]
Xia, S, et al., 2023	80	72.70	15.80	80	68.00	13.50	followup	12.00	0.32	[0.01; 0.63]
Xia, S, et al., 2023	80	78.00	14.30	80	72.90	13.40	followup	24.00	0.37	[0.05; 0.68]
Yoo, HJ, et al. 2004	30	97.01	18.24	30	88.13	16.18	followup	12.00	0.51	[-0.01; 1.02]
Hernandez, EG, et al. 2018	28	71.73	15.45	28	62.80	17.63	followup	24.00	0.53	[-0.00; 1.06]
Compen F, et al. 2020 II.	90	0.85	0.17	78	0.75	0.19	followup	8.00	0.55	[0.25; 0.86]
Mihuta, ME, et al., 2018	32	14.10	2.60	33	12.30	3.60	followup	4.00	0.56	[0.07; 1.06]
Compen F, et al. 2020 I.	77	0.86	0.13	78	0.75	0.19	followup	8.00	0.67	[0.35; 1.00]
Hernandez, EG, et al. 2018	28	74.38	11.48	28	64.40	14.73	followup	8.00	0.75	[0.20; 1.29]
Cengiz, HO, et al., 2023	32	66.98	17.72	33	53.03	18.65	followup	8.00	0.76	[0.25; 1.26]
Yoo, HJ, et al. 2004	30	102.00	19.94	30	88.18	14.02	followup	24.00	0.79	[0.26; 1.32]
Rahmani, S, et al. 2015	12	66.66	9.40	12	54.16	10.95	followup	8.00	1.18	[0.30; 2.06]
Liu, T, et al., 2019	49	62.67	10.32	53	49.47	10.92	followup	9.00	1.23	[0.81; 1.66]
Liu, T, et al., 2019	49	72.33	13.36	53	56.95	10.03	followup	12.00	1.30	[0.87; 1.73]
Jelvehzadeh, F, et al., 2022	24	6.05	1.00	24	4.65	0.80	followup	12.00	1.52	[0.87; 2.17]
Jelvehzadeh, F, et al., 2022	24	6.16	1.09	24	4.58	0.92	followup	8.00	1.54	[0.89; 2.19]
Nikbakhsh, N, et al., 2018	20	81.70	10.04	20	47.39	12.93	followup	12.00	2.91	[1.99; 3.82]
Random effect	2872			2539					0.63	[-0.25; 1.50]
Prediction interval										[-0.92; 2.17]
**self-help**										
van den Berg, SW, et al. 2015 II.	70	66.79	16.57	80	69.79	17.91	baseline	0.00	-0.17	[-0.49; 0.15]
Wang, TJ, et al 2023	70	102.30	18.40	72	104.40	17.10	baseline	0.00	-0.12	[-0.45; 0.21]
van der Hout, AVD, et al. 2019	320	85.30	14.90	304	85.40	13.60	baseline	0.00	-0.01	[-0.16; 0.15]
Walker, LG, et al.,1998	48	3.25	0.70	48	3.25	0.66	baseline	0.00	0.00	[-0.40; 0.40]
Rosen, KD, et al., 2018	57	93.61	22.68	55	92.96	25.12	baseline	0.00	0.03	[-0.34; 0.40]
Wang, TJ, et al 2023	70	105.40	17.80	72	103.50	20.00	followup	8.00	0.10	[-0.23; 0.43]
van der Hout, AVD, et al. 2019	320	88.70	13.20	304	86.50	13.10	followup	12.00	0.17	[0.01; 0.32]
van der Hout, AVD, et al. 2019	320	88.40	12.10	304	86.20	12.80	followup	1.00	0.18	[0.02; 0.33]
van der Hout, AVD, et al. 2019	320	89.30	12.30	304	87.00	12.70	followup	24.00	0.18	[0.03; 0.34]
Wang, TJ, et al 2023	70	107.30	16.70	72	103.30	19.00	followup	16.00	0.22	[-0.11; 0.55]
Wang, TJ, et al 2023	70	106.90	16.60	72	102.70	20.00	followup	24.00	0.23	[-0.10; 0.56]
Walker, LG, et al.,1998	48	3.29	0.80	48	2.90	0.78	followup	15.00	0.49	[0.08; 0.90]
Rosen, KD, et al., 2018	57	107.35	17.79	55	96.04	23.96	followup	5.00	0.53	[0.16; 0.91]
van den Berg, SW, et al. 2015 II.	70	73.30	1.78	80	69.88	1.67	followup	4.00	1.97	[1.58; 2.36]
Random effect	1910			1870					0.60	[-0.38; 1.58]
Prediction interval										[-1.10; 2.31]
**Random effect**	**12488**			**12601**					**0.69**	**[-0.29; 1.67]**
**Prediction interval**										**[-0.99; 2.36]**

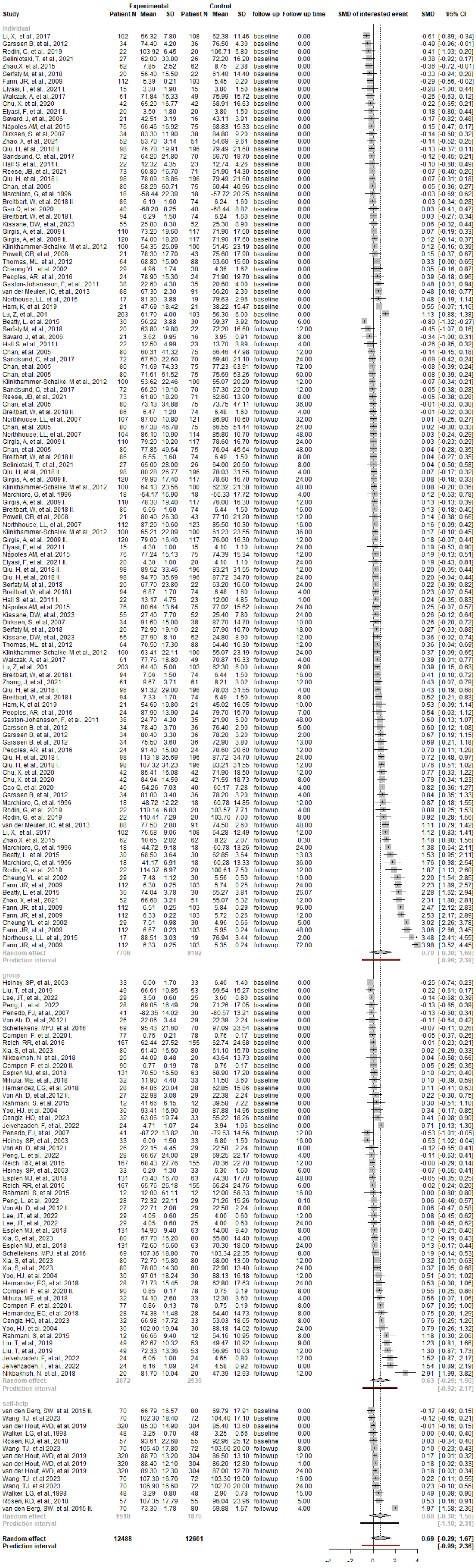

Figure S5.4. Forest plot represents the difference between the intervention vs. control group in the Global QoL domain with the type subgroups as predicted at week 48 (post-intervention). SMD - Standardized mean difference, CI - confidence interval.

S6. Subgroup analysis of Global QoL: Cancer Stage

Figure S6.1.T0

Study	Experimental Patient N	Experimental Mean	Experimental SD	Control Patient N	Control Mean	Control SD	follow-up	Follow-up time	SMD	95%-CI
**early**										
Zhao,X, et al. 2015	62	7.85	2.52	62	8.75	2.38	baseline	0.00	-0.36	[-0.72; -0.01]
Chu, X. et al, 2020	42	65.20	16.77	42	68.91	16.63	baseline	0.00	-0.22	[-0.65; 0.21]
Liu, T, et al., 2019	49	66.61	10.85	53	69.54	15.27	baseline	0.00	-0.22	[-0.61; 0.17]
Zhao, X, et al., 2021	52	53.70	3.14	51	54.69	9.61	baseline	0.00	-0.14	[-0.52; 0.25]
Wang, TJ, et al 2023	70	102.30	18.40	72	104.40	17.10	baseline	0.00	-0.12	[-0.45; 0.21]
Marchioro, G, et al. 1996	18	-58.44	22.39	18	-57.72	20.25	baseline	0.00	-0.03	[-0.69; 0.62]
Walker, LG, et al.,1998	48	3.25	0.70	48	3.25	0.66	baseline	0.00	0.00	[-0.40; 0.40]
Nikbakhsh, N, et al., 2018	20	44.09	8.48	20	43.64	13.73	baseline	0.00	0.04	[-0.58; 0.66]
Yoo, HJ, et al. 2004	30	93.41	16.90	30	87.88	14.96	baseline	0.00	0.34	[-0.17; 0.85]
Cengiz, HO, et al., 2023	32	63.06	19.74	33	55.22	18.26	baseline	0.00	0.41	[-0.08; 0.90]
Beatty, L. et al. 2015	30	56.22	3.88	30	59.37	3.92	followup	6.00	-0.80	[-1.32; -0.27]
Wang, TJ, et al 2023	70	105.40	17.80	72	103.50	20.00	followup	8.00	0.10	[-0.23; 0.43]
Marchioro, G, et al. 1996	18	-54.17	16.90	18	-56.33	17.72	followup	4.00	0.12	[-0.53; 0.78]
Wang, TJ, et al 2023	70	107.30	16.70	72	103.30	19.00	followup	16.00	0.22	[-0.11; 0.55]
Wang, TJ, et al 2023	70	106.90	16.60	72	102.70	20.00	followup	24.00	0.23	[-0.10; 0.56]
Walker, LG, et al.,1998	48	3.29	0.80	48	2.90	0.78	followup	15.00	0.49	[0.08; 0.90]
Yoo, HJ, et al. 2004	30	97.01	18.24	30	88.13	16.18	followup	12.00	0.51	[-0.01; 1.02]
Cengiz, HO, et al., 2023	32	66.98	17.72	33	53.03	18.65	followup	8.00	0.76	[0.25; 1.26]
Chu, X. et al, 2020	42	85.41	16.08	42	71.90	18.50	followup	12.00	0.77	[0.33; 1.22]
Chu, X. et al, 2020	42	84.94	14.59	42	71.59	18.73	followup	8.00	0.79	[0.34; 1.23]
Yoo, HJ, et al. 2004	30	102.00	19.94	30	88.18	14.02	followup	24.00	0.79	[0.26; 1.32]
Marchioro, G, et al. 1996	18	-48.72	12.22	18	-60.78	14.85	followup	12.00	0.87	[0.18; 1.55]
Zhao,X, et al. 2015	62	10.65	2.02	62	8.22	2.07	followup	0.30	1.18	[0.80; 1.56]
Liu, T, et al., 2019	49	62.67	10.32	53	49.47	10.92	followup	9.00	1.23	[0.81; 1.66]
Liu, T, et al., 2019	49	72.33	13.36	53	56.95	10.03	followup	12.00	1.30	[0.87; 1.73]
Marchioro, G, et al. 1996	18	-44.72	9.18	18	-60.78	13.26	followup	24.00	1.38	[0.64; 2.11]
Beatty, L. et al. 2015	30	68.50	3.64	30	62.85	3.64	followup	13.03	1.53	[0.95; 2.11]
Marchioro, G, et al. 1996	18	-41.17	6.91	18	-60.28	13.33	followup	36.00	1.76	[0.98; 2.54]
Beatty, L. et al. 2015	30	74.04	3.78	30	65.27	3.81	followup	26.07	2.28	[1.62; 2.94]
Zhao, X, et al., 2021	52	66.68	3.21	51	55.07	6.32	followup	12.00	2.31	[1.80; 2.81]
Nikbakhsh, N, et al., 2018	20	81.70	10.04	20	47.39	12.93	followup	12.00	2.91	[1.99; 3.82]
Random effect	1251			1271					0.66	[0.14; 1.17]
Prediction interval										[-0.65; 1.97]
**advanced**										
Rodin, G, et al., 2019	22	103.92	6.45	20	106.71	6.80	baseline	0.00	-0.41	[-1.03; 0.20]
Serfaty M, et al., 2018	20	56.40	15.50	22	61.40	14.40	baseline	0.00	-0.33	[-0.94; 0.28]
Walczak, A, et al.,2017	61	71.84	16.33	49	75.99	15.72	baseline	0.00	-0.26	[-0.63; 0.12]
Heiney, SP, et al., 2003	33	6.00	1.70	33	6.40	1.40	baseline	0.00	-0.25	[-0.74; 0.23]
Savard, J, et al., 2006	21	42.51	3.19	16	43.11	3.91	baseline	0.00	-0.17	[-0.82; 0.48]
Penedo, FJ, et al., 2007	41	-82.35	14.02	30	-80.57	13.21	baseline	0.00	-0.13	[-0.60; 0.34]
Kissane, DW, et al., 2023	55	25.80	8.30	52	25.30	8.90	baseline	0.00	0.06	[-0.32; 0.44]
Lu, Z, et al., 201	203	61.70	4.00	103	56.30	6.00	baseline	0.00	1.13	[0.88; 1.38]
Penedo, FJ, et al., 2007	41	-87.22	13.82	30	-79.63	14.56	followup	12.00	-0.53	[-1.01; -0.05]
Heiney, SP, et al., 2003	33	6.00	1.50	33	6.80	1.50	followup	16.00	-0.53	[-1.02; -0.04]
Serfaty M, et al., 2018	20	63.80	19.80	22	72.20	16.60	followup	12.00	-0.45	[-1.07; 0.16]
Savard, J, et al., 2006	21	3.62	0.95	16	3.95	0.91	followup	8.00	-0.34	[-1.00; 0.31]
Heiney, SP, et al., 2003	33	6.20	1.30	33	6.30	1.60	followup	6.00	-0.07	[-0.55; 0.41]
Serfaty M, et al., 2018	20	67.70	23.80	22	63.20	16.60	followup	24.00	0.22	[-0.39; 0.82]
Kissane, DW, et al., 2023	55	27.40	7.70	52	25.40	7.80	followup	24.00	0.26	[-0.12; 0.64]
Serfaty M, et al., 2018	20	72.90	19.10	22	67.90	16.70	followup	18.00	0.27	[-0.33; 0.88]
Kissane, DW, et al., 2023	55	27.90	8.10	52	24.80	8.90	followup	12.00	0.36	[-0.02; 0.74]
Walczak, A, et al.,2017	61	77.76	18.80	49	70.87	16.33	followup	4.00	0.39	[0.01; 0.77]
Lu, Z, et al., 201	203	64.40	5.00	103	62.30	6.00	followup	9.00	0.39	[0.15; 0.63]
Rodin, G, et al., 2019	22	110.14	6.83	20	103.57	7.71	followup	4.00	0.89	[0.25; 1.53]
Rodin, G, et al., 2019	22	110.41	7.29	20	103.70	7.00	followup	8.00	0.92	[0.28; 1.56]
Rodin, G, et al., 2019	22	114.37	6.97	20	100.61	7.50	followup	12.00	1.87	[1.13; 2.60]
Random effect	1084			819					0.21	[-0.44; 0.85]
Prediction interval										[-1.15; 1.56]
**survivor**										
van den Berg, SW, et al. 2015 II.	70	66.79	16.57	80	69.79	17.91	baseline	0.00	-0.17	[-0.49; 0.15]
Lee, JT, et al., 2022	29	3.50	0.60	25	3.60	0.80	baseline	0.00	-0.14	[-0.68; 0.39]
Peng, L, et al., 2022	28	69.05	16.49	29	71.26	17.05	baseline	0.00	-0.13	[-0.65; 0.39]
Qiu, H, et al., 2018 II.	98	76.78	19.91	196	79.49	21.60	baseline	0.00	-0.13	[-0.37; 0.11]
Von Ah, D, et al.,2012 I.	26	22.06	3.44	29	22.38	2.24	baseline	0.00	-0.11	[-0.64; 0.42]
Qiu, H, et al., 2018 I.	98	78.09	18.86	196	79.49	21.60	baseline	0.00	-0.07	[-0.31; 0.18]
Dieng M, et al. 2020	70	0.78	0.20	81	0.77	0.22	baseline	0.00	0.05	[-0.27; 0.37]
Von Ah, D, et al.,2012 II.	27	22.98	3.08	29	22.38	2.24	baseline	0.00	0.22	[-0.30; 0.75]
Peoples, AR, et al., 2016	24	78.90	15.30	24	71.90	19.70	baseline	0.00	0.39	[-0.18; 0.96]
McCaughan, E, et al., 2018	13	85.83	14.10	4	76.88	22.04	baseline	0.00	0.53	[-0.61; 1.67]
Jelvehzadeh, F, et al., 2022	24	4.71	1.07	24	3.94	1.06	baseline	0.00	0.71	[0.13; 1.30]
Von Ah, D, et al.,2012 I.	26	22.15	4.45	29	22.58	2.24	followup	8.00	-0.12	[-0.65; 0.41]
Peng, L, et al., 2022	28	66.67	24.00	29	69.25	22.17	followup	4.00	-0.11	[-0.63; 0.41]
Dieng M, et al. 2020	70	0.05	0.18	81	0.04	0.23	followup	48.00	0.05	[-0.27; 0.37]
Dieng M, et al. 2020	70	0.04	0.16	81	0.03	0.23	followup	26.00	0.05	[-0.27; 0.37]
Peng, L, et al., 2022	28	72.32	22.11	29	71.26	15.26	followup	0.10	0.06	[-0.46; 0.57]
Von Ah, D, et al.,2012 II.	27	22.71	2.08	29	22.58	2.24	followup	8.00	0.06	[-0.47; 0.58]
Qiu, H, et al., 2018 II.	98	80.28	26.77	196	78.03	31.55	followup	4.00	0.07	[-0.17; 0.32]
Lee, JT, et al., 2022	29	4.05	0.60	25	4.00	0.60	followup	12.00	0.08	[-0.45; 0.62]
Lee, JT, et al., 2022	29	4.05	0.60	25	4.00	0.60	followup	24.00	0.08	[-0.45; 0.62]
Qiu, H, et al., 2018 II.	98	89.52	33.46	196	83.21	31.55	followup	12.00	0.20	[-0.05; 0.44]
Qiu, H, et al., 2018 II.	98	94.70	35.69	196	87.72	34.70	followup	24.00	0.20	[-0.04; 0.44]
McCaughan, E, et al., 2018	13	85.76	17.07	4	80.22	22.75	followup	4.00	0.29	[-0.84; 1.41]
Qiu, H, et al., 2018 I.	98	91.32	29.00	196	78.03	31.55	followup	4.00	0.43	[0.19; 0.68]
Peoples, AR, et al., 2016	24	87.90	13.90	24	79.70	15.70	followup	7.00	0.54	[-0.03; 1.12]
Peoples, AR, et al., 2016	24	91.40	15.00	24	78.60	20.60	followup	12.00	0.70	[0.11; 1.28]
Qiu, H, et al., 2018 I.	98	113.18	35.69	196	87.72	34.70	followup	24.00	0.72	[0.48; 0.97]
Qiu, H, et al., 2018 I.	98	107.32	31.23	196	83.21	31.55	followup	12.00	0.76	[0.51; 1.02]
Jelvehzadeh, F, et al., 2022	24	6.05	1.00	24	4.65	0.80	followup	12.00	1.52	[0.87; 2.17]
Jelvehzadeh, F, et al., 2022	24	6.16	1.09	24	4.58	0.92	followup	8.00	1.54	[0.89; 2.19]
van den Berg, SW, et al. 2015 II.	70	73.30	1.78	80	69.88	1.67	followup	4.00	1.97	[1.58; 2.36]
Random effect	1581			2401					0.39	[-0.23; 1.01]
Prediction interval										[-0.97; 1.75]
**Random effect**	**3916**			**4491**					**0.41**	**[-0.16; 0.98]**
**Prediction interval**										**[-0.94; 1.76]**

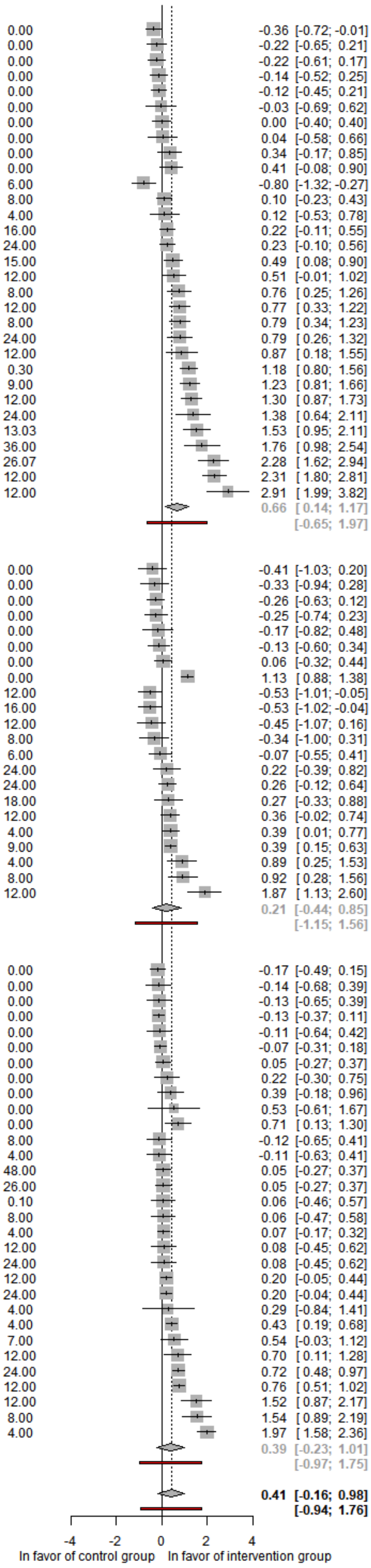

Figure S6.1. Subgroup analysis of the Global QoL. Forest plot represents the difference between the intervention vs. control group in the Global QoL domain with the cancer stage subgroups as predicted at week 0 (post-intervention). SMD - Standardized mean difference, CI - confidence interval.

Figure S6.2.T12

Study	Experimental Patient N	Experimental Mean	Experimental SD	Control Patient N	Control Mean	Control SD	follow-up	Follow-up time	SMD	95%-CI
early										
Zhao,X, et al. 2015	62	7.85	2.52	62	8.75	2.38	baseline	0.00	-0.36	[-0.72; -0.01]
Chu, X. et al, 2020	42	65.20	16.77	42	68.91	16.63	baseline	0.00	-0.22	[-0.65; 0.21]
Liu, T, et al., 2019	49	66.61	10.85	53	69.54	15.27	baseline	0.00	-0.22	[-0.61; 0.17]
Zhao, X, et al., 2021	52	53.70	3.14	51	54.69	9.61	baseline	0.00	-0.14	[-0.52; 0.25]
Wang, TJ, et al 2023	70	102.30	18.40	72	104.40	17.10	baseline	0.00	-0.12	[-0.45; 0.21]
Marchioro, G, et al. 1996	18	-58.44	22.39	18	-57.72	20.25	baseline	0.00	-0.03	[-0.69; 0.62]
Walker, LG, et al.,1998	48	3.25	0.70	48	3.25	0.66	baseline	0.00	0.00	[-0.40; 0.40]
Nikbakhsh, N, et al., 2018	20	44.09	8.48	20	43.64	13.73	baseline	0.00	0.04	[-0.58; 0.66]
Yoo, HJ, et al. 2004	30	93.41	16.90	30	87.88	14.96	baseline	0.00	0.34	[-0.17; 0.85]
Cengiz, HO, et al., 2023	32	63.06	19.74	33	55.22	18.26	baseline	0.00	0.41	[-0.08; 0.90]
Beatty, L. et al. 2015	30	56.22	3.88	30	59.37	3.92	followup	6.00	-0.80	[-1.32; -0.27]
Wang, TJ, et al 2023	70	105.40	17.80	72	103.50	20.00	followup	8.00	0.10	[-0.23; 0.43]
Marchioro, G, et al. 1996	18	-54.17	16.90	18	-56.33	17.72	followup	4.00	0.12	[-0.53; 0.78]
Wang, TJ, et al 2023	70	107.30	16.70	72	103.30	19.00	followup	16.00	0.22	[-0.11; 0.55]
Wang, TJ, et al 2023	70	106.90	16.60	72	102.70	20.00	followup	24.00	0.23	[-0.10; 0.56]
Walker, LG, et al.,1998	48	3.29	0.80	48	2.90	0.78	followup	15.00	0.49	[0.08; 0.90]
Yoo, HJ, et al. 2004	30	97.01	18.24	30	88.13	16.18	followup	12.00	0.51	[-0.01; 1.02]
Cengiz, HO, et al., 2023	32	66.98	17.72	33	53.03	18.65	followup	8.00	0.76	[0.25; 1.26]
Chu, X. et al, 2020	42	85.41	16.08	42	71.90	18.50	followup	12.00	0.77	[0.33; 1.22]
Chu, X. et al, 2020	42	84.94	14.59	42	71.59	18.73	followup	8.00	0.79	[0.34; 1.23]
Yoo, HJ, et al. 2004	30	102.00	19.94	30	88.18	14.02	followup	24.00	0.79	[0.26; 1.32]
Marchioro, G, et al. 1996	18	-48.72	12.22	18	-60.78	14.85	followup	12.00	0.87	[0.18; 1.55]
Zhao,X, et al. 2015	62	10.65	2.02	62	8.22	2.07	followup	0.30	1.18	[0.80; 1.56]
Liu, T, et al., 2019	49	62.67	10.32	53	49.47	10.92	followup	9.00	1.23	[0.81; 1.66]
Liu, T, et al., 2019	49	72.33	13.36	53	56.95	10.03	followup	12.00	1.30	[0.87; 1.73]
Marchioro, G, et al. 1996	18	-44.72	9.18	18	-60.78	13.26	followup	24.00	1.38	[0.64; 2.11]
Beatty, L. et al. 2015	30	68.50	3.64	30	62.85	3.64	followup	13.03	1.53	[0.95; 2.11]
Marchioro, G, et al. 1996	18	-41.17	6.91	18	-60.28	13.33	followup	36.00	1.76	[0.98; 2.54]
Beatty, L. et al. 2015	30	74.04	3.78	30	65.27	3.81	followup	26.07	2.28	[1.62; 2.94]
Zhao, X, et al., 2021	52	66.68	3.21	51	55.07	6.32	followup	12.00	2.31	[1.80; 2.81]
Nikbakhsh, N, et al., 2018	20	81.70	10.04	20	47.39	12.93	followup	12.00	2.91	[1.99; 3.82]
Random effect	1251			1271					0.82	[0.49; 1.14]
Prediction interval										[-0.45; 2.08]
advanced										
Rodin, G, et al., 2019	22	103.92	6.45	20	106.71	6.80	baseline	0.00	-0.41	[-1.03; 0.20]
Serfaty M, et al., 2018	20	56.40	15.50	22	61.40	14.40	baseline	0.00	-0.33	[-0.94; 0.28]
Walczak, A, et al.,2017	61	71.84	16.33	49	75.99	15.72	baseline	0.00	-0.26	[-0.63; 0.12]
Heiney, SP, et al., 2003	33	6.00	1.70	33	6.40	1.40	baseline	0.00	-0.25	[-0.74; 0.23]
Savard, J, et al., 2006	21	42.51	3.19	16	43.11	3.91	baseline	0.00	-0.17	[-0.82; 0.48]
Penedo, FJ, et al., 2007	41	-82.35	14.02	30	-80.57	13.21	baseline	0.00	-0.13	[-0.60; 0.34]
Kissane, DW, et al., 2023	55	25.80	8.30	52	25.30	8.90	baseline	0.00	0.06	[-0.32; 0.44]
Lu, Z, et al., 201	203	61.70	4.00	103	56.30	6.00	baseline	0.00	1.13	[0.88; 1.38]
Penedo, FJ, et al., 2007	41	-87.22	13.82	30	-79.63	14.56	followup	12.00	-0.53	[-1.01; -0.05]
Heiney, SP, et al., 2003	33	6.00	1.50	33	6.80	1.50	followup	16.00	-0.53	[-1.02; -0.04]
Serfaty M, et al., 2018	20	63.80	19.80	22	72.20	16.60	followup	12.00	-0.45	[-1.07; 0.16]
Savard, J, et al., 2006	21	3.62	0.95	16	3.95	0.91	followup	8.00	-0.34	[-1.00; 0.31]
Heiney, SP, et al., 2003	33	6.20	1.30	33	6.30	1.60	followup	6.00	-0.07	[-0.55; 0.41]
Serfaty M, et al., 2018	20	67.70	23.80	22	63.20	16.60	followup	24.00	0.22	[-0.39; 0.82]
Kissane, DW, et al., 2023	55	27.40	7.70	52	25.40	7.80	followup	24.00	0.26	[-0.12; 0.64]
Serfaty M, et al., 2018	20	72.90	19.10	22	67.90	16.70	followup	18.00	0.27	[-0.33; 0.88]
Kissane, DW, et al., 2023	55	27.90	8.10	52	24.80	8.90	followup	12.00	0.36	[-0.02; 0.74]
Walczak, A, et al.,2017	61	77.76	18.80	49	70.87	16.33	followup	4.00	0.39	[0.01; 0.77]
Lu, Z, et al., 201	203	64.40	5.00	103	62.30	6.00	followup	9.00	0.39	[0.15; 0.63]
Rodin, G, et al., 2019	22	110.14	6.83	20	103.57	7.71	followup	4.00	0.89	[0.25; 1.53]
Rodin, G, et al., 2019	22	110.41	7.29	20	103.70	7.00	followup	8.00	0.92	[0.28; 1.56]
Rodin, G, et al., 2019	22	114.37	6.97	20	100.61	7.50	followup	12.00	1.87	[1.13; 2.60]
Random effect	1084			819					0.36	[-0.07; 0.80]
Prediction interval										[-0.98; 1.71]
survivor										
van den Berg, SW, et al. 2015 II.	70	66.79	16.57	80	69.79	17.91	baseline	0.00	-0.17	[-0.49; 0.15]
Lee, JT, et al., 2022	29	3.50	0.60	25	3.60	0.80	baseline	0.00	-0.14	[-0.68; 0.39]
Peng, L, et al., 2022	28	69.05	16.49	29	71.26	17.05	baseline	0.00	-0.13	[-0.65; 0.39]
Qiu, H, et al., 2018 II.	98	76.78	19.91	196	79.49	21.60	baseline	0.00	-0.13	[-0.37; 0.11]
Von Ah, D, et al.,2012 I.	26	22.06	3.44	29	22.38	2.24	baseline	0.00	-0.11	[-0.64; 0.42]
Qiu, H, et al., 2018 I.	98	78.09	18.86	196	79.49	21.60	baseline	0.00	-0.07	[-0.31; 0.18]
Dieng M, et al. 2020	70	0.78	0.20	81	0.77	0.22	baseline	0.00	0.05	[-0.27; 0.37]
Von Ah, D, et al.,2012 II.	27	22.98	3.08	29	22.38	2.24	baseline	0.00	0.22	[-0.30; 0.75]
Peoples, AR, et al., 2016	24	78.90	15.30	24	71.90	19.70	baseline	0.00	0.39	[-0.18; 0.96]
McCaughan, E, et al., 2018	13	85.83	14.10	4	76.88	22.04	baseline	0.00	0.53	[-0.61; 1.67]
Jelvehzadeh, F, et al., 2022	24	4.71	1.07	24	3.94	1.06	baseline	0.00	0.71	[0.13; 1.30]
Von Ah, D, et al.,2012 I.	26	22.15	4.45	29	22.58	2.24	followup	8.00	-0.12	[-0.65; 0.41]
Peng, L, et al., 2022	28	66.67	24.00	29	69.25	22.17	followup	4.00	-0.11	[-0.63; 0.41]
Dieng M, et al. 2020	70	0.05	0.18	81	0.04	0.23	followup	48.00	0.05	[-0.27; 0.37]
Dieng M, et al. 2020	70	0.04	0.16	81	0.03	0.23	followup	26.00	0.05	[-0.27; 0.37]
Peng, L, et al., 2022	28	72.32	22.11	29	71.26	15.26	followup	0.10	0.06	[-0.46; 0.57]
Von Ah, D, et al.,2012 II.	27	22.71	2.08	29	22.58	2.24	followup	8.00	0.06	[-0.47; 0.58]
Qiu, H, et al., 2018 II.	98	80.28	26.77	196	78.03	31.55	followup	4.00	0.07	[-0.17; 0.32]
Lee, JT, et al., 2022	29	4.05	0.60	25	4.00	0.60	followup	12.00	0.08	[-0.45; 0.62]
Lee, JT, et al., 2022	29	4.05	0.60	25	4.00	0.60	followup	24.00	0.08	[-0.45; 0.62]
Qiu, H, et al., 2018 II.	98	89.52	33.46	196	83.21	31.55	followup	12.00	0.20	[-0.05; 0.44]
Qiu, H, et al., 2018 II.	98	94.70	35.69	196	87.72	34.70	followup	24.00	0.20	[-0.04; 0.44]
McCaughan, E, et al., 2018	13	85.76	17.07	4	80.22	22.75	followup	4.00	0.29	[-0.84; 1.41]
Qiu, H, et al., 2018 I.	98	91.32	29.00	196	78.03	31.55	followup	4.00	0.43	[0.19; 0.68]
Peoples, AR, et al., 2016	24	87.90	13.90	24	79.70	15.70	followup	7.00	0.54	[-0.03; 1.12]
Peoples, AR, et al., 2016	24	91.40	15.00	24	78.60	20.60	followup	12.00	0.70	[0.11; 1.28]
Qiu, H, et al., 2018 I.	98	113.18	35.69	196	87.72	34.70	followup	24.00	0.72	[0.48; 0.97]
Qiu, H, et al., 2018 I.	98	107.32	31.23	196	83.21	31.55	followup	12.00	0.76	[0.51; 1.02]
Jelvehzadeh, F, et al., 2022	24	6.05	1.00	24	4.65	0.80	followup	12.00	1.52	[0.87; 2.17]
Jelvehzadeh, F, et al., 2022	24	6.16	1.09	24	4.58	0.92	followup	8.00	1.54	[0.89; 2.19]
van den Berg, SW, et al. 2015 II.	70	73.30	1.78	80	69.88	1.67	followup	4.00	1.97	[1.58; 2.36]
Random effect	1581			2401					0.55	[0.20; 0.90]
Prediction interval										[-0.73; 1.83]
**Random effect**	**3916**			**4491**					**0.61**	**[0.34; 0.87]**
**Prediction interval**										**[-0.58; 1.79]**

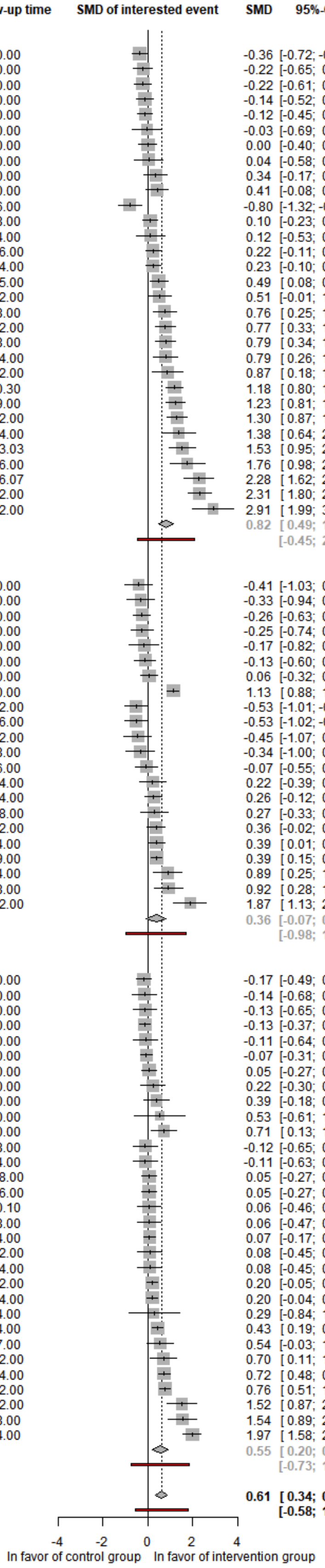

Figure S6.2. Forest plot represents the difference between the intervention vs. control group in the Global QoL domain with the cancer stage subgroups as predicted at week 12 (post-intervention). SMD - Standardized mean difference, CI - confidence interval.

Figure S6.3.T24

Study	Experimental Patient N	Experimental Mean	Experimental SD	Control Patient N	Control Mean	Control SD	follow-up	Follow-up time	SMD	95%-CI
**early**										
Zhao,X, et al. 2015	62	7.85	2.52	62	8.75	2.38	baseline	0.00	-0.36	[-0.72; -0.01]
Chu, X. et al, 2020	42	65.20	16.77	42	68.91	16.63	baseline	0.00	-0.22	[-0.65; 0.21]
Liu, T, et al., 2019	49	66.61	10.85	53	69.54	15.27	baseline	0.00	-0.22	[-0.61; 0.17]
Zhao, X, et al., 2021	52	53.70	3.14	51	54.69	9.61	baseline	0.00	-0.14	[-0.52; 0.25]
Wang, TJ, et al 2023	70	102.30	18.40	72	104.40	17.10	baseline	0.00	-0.12	[-0.45; 0.21]
Marchioro, G, et al. 1996	18	-58.44	22.39	18	-57.72	20.25	baseline	0.00	-0.03	[-0.69; 0.62]
Walker, LG, et al.,1998	48	3.25	0.70	48	3.25	0.66	baseline	0.00	0.00	[-0.40; 0.40]
Nikbakhsh, N, et al., 2018	20	44.09	8.48	20	43.64	13.73	baseline	0.00	0.04	[-0.58; 0.66]
Yoo, HJ, et al. 2004	30	93.41	16.90	30	87.88	14.96	baseline	0.00	0.34	[-0.17; 0.85]
Cengiz, HO, et al., 2023	32	63.06	19.74	33	55.22	18.26	baseline	0.00	0.41	[-0.08; 0.90]
Beatty, L. et al. 2015	30	56.22	3.88	30	59.37	3.92	followup	6.00	-0.80	[-1.32; -0.27]
Wang, TJ, et al 2023	70	105.40	17.80	72	103.50	20.00	followup	8.00	0.10	[-0.23; 0.43]
Marchioro, G, et al. 1996	18	-54.17	16.90	18	-56.33	17.72	followup	4.00	0.12	[-0.53; 0.78]
Wang, TJ, et al 2023	70	107.30	16.70	72	103.30	19.00	followup	16.00	0.22	[-0.11; 0.55]
Wang, TJ, et al 2023	70	106.90	16.60	72	102.70	20.00	followup	24.00	0.23	[-0.10; 0.56]
Walker, LG, et al.,1998	48	3.29	0.80	48	2.90	0.78	followup	15.00	0.49	[0.08; 0.90]
Yoo, HJ, et al. 2004	30	97.01	18.24	30	88.13	16.18	followup	12.00	0.51	[-0.01; 1.02]
Cengiz, HO, et al., 2023	32	66.98	17.72	33	53.03	18.65	followup	8.00	0.76	[0.25; 1.26]
Chu, X. et al, 2020	42	85.41	16.08	42	71.90	18.50	followup	12.00	0.77	[0.33; 1.22]
Chu, X. et al, 2020	42	84.94	14.59	42	71.59	18.73	followup	8.00	0.79	[0.34; 1.23]
Yoo, HJ, et al. 2004	30	102.00	19.94	30	88.18	14.02	followup	24.00	0.79	[0.26; 1.32]
Marchioro, G, et al. 1996	18	-48.72	12.22	18	-60.78	14.85	followup	12.00	0.87	[0.18; 1.55]
Zhao,X, et al. 2015	62	10.65	2.02	62	8.22	2.07	followup	0.30	1.18	[0.80; 1.56]
Liu, T, et al., 2019	49	62.67	10.32	53	49.47	10.92	followup	9.00	1.23	[0.81; 1.66]
Liu, T, et al., 2019	49	72.33	13.36	53	56.95	10.03	followup	12.00	1.30	[0.87; 1.73]
Marchioro, G, et al. 1996	18	-44.72	9.18	18	-60.78	13.26	followup	24.00	1.38	[0.64; 2.11]
Beatty, L. et al. 2015	30	68.50	3.64	30	62.85	3.64	followup	13.03	1.53	[0.95; 2.11]
Marchioro, G, et al. 1996	18	-41.17	6.91	18	-60.28	13.33	followup	36.00	1.76	[0.98; 2.54]
Beatty, L. et al. 2015	30	74.04	3.78	30	65.27	3.81	followup	26.07	2.28	[1.62; 2.94]
Zhao, X, et al., 2021	52	66.68	3.21	51	55.07	6.32	followup	12.00	2.31	[1.80; 2.81]
Nikbakhsh, N, et al., 2018	20	81.70	10.04	20	47.39	12.93	followup	12.00	2.91	[1.99; 3.82]
Random effect	1251			1271					0.85	[0.36; 1.35]
Prediction interval										[-0.45; 2.16]
**advanced**										
Rodin, G, et al., 2019	22	103.92	6.45	20	106.71	6.80	baseline	0.00	-0.41	[-1.03; 0.20]
Serfaty M, et al., 2018	20	56.40	15.50	22	61.40	14.40	baseline	0.00	-0.33	[-0.94; 0.28]
Walczak, A, et al.,2017	61	71.84	16.33	49	75.99	15.72	baseline	0.00	-0.26	[-0.63; 0.12]
Heiney, SP, et al., 2003	33	6.00	1.70	33	6.40	1.40	baseline	0.00	-0.25	[-0.74; 0.23]
Savard, J, et al., 2006	21	42.51	3.19	16	43.11	3.91	baseline	0.00	-0.17	[-0.82; 0.48]
Penedo, FJ, et al., 2007	41	-82.35	14.02	30	-80.57	13.21	baseline	0.00	-0.13	[-0.60; 0.34]
Kissane, DW, et al., 2023	55	25.80	8.30	52	25.30	8.90	baseline	0.00	0.06	[-0.32; 0.44]
Lu, Z, et al., 201	203	61.70	4.00	103	56.30	6.00	baseline	0.00	1.13	[0.88; 1.38]
Penedo, FJ, et al., 2007	41	-87.22	13.82	30	-79.63	14.56	followup	12.00	-0.53	[-1.01; -0.05]
Heiney, SP, et al., 2003	33	6.00	1.50	33	6.80	1.50	followup	16.00	-0.53	[-1.02; -0.04]
Serfaty M, et al., 2018	20	63.80	19.80	22	72.20	16.60	followup	12.00	-0.45	[-1.07; 0.16]
Savard, J, et al., 2006	21	3.62	0.95	16	3.95	0.91	followup	8.00	-0.34	[-1.00; 0.31]
Heiney, SP, et al., 2003	33	6.20	1.30	33	6.30	1.60	followup	6.00	-0.07	[-0.55; 0.41]
Serfaty M, et al., 2018	20	67.70	23.80	22	63.20	16.60	followup	24.00	0.22	[-0.39; 0.82]
Kissane, DW, et al., 2023	55	27.40	7.70	52	25.40	7.80	followup	24.00	0.26	[-0.12; 0.64]
Serfaty M, et al., 2018	20	72.90	19.10	22	67.90	16.70	followup	18.00	0.27	[-0.33; 0.88]
Kissane, DW, et al., 2023	55	27.90	8.10	52	24.80	8.90	followup	12.00	0.36	[-0.02; 0.74]
Walczak, A, et al.,2017	61	77.76	18.80	49	70.87	16.33	followup	4.00	0.39	[0.01; 0.77]
Lu, Z, et al., 201	203	64.40	5.00	103	62.30	6.00	followup	9.00	0.39	[0.15; 0.63]
Rodin, G, et al., 2019	22	110.14	6.83	20	103.57	7.71	followup	4.00	0.89	[0.25; 1.53]
Rodin, G, et al., 2019	22	110.41	7.29	20	103.70	7.00	followup	8.00	0.92	[0.28; 1.56]
Rodin, G, et al., 2019	22	114.37	6.97	20	100.61	7.50	followup	12.00	1.87	[1.13; 2.60]
Random effect	1084			819					0.40	[-0.12; 0.93]
Prediction interval										[-0.93; 1.74]
**survivor**										
van den Berg, SW, et al. 2015 II.	70	66.79	16.57	80	69.79	17.91	baseline	0.00	-0.17	[-0.49; 0.15]
Lee, JT, et al., 2022	29	3.50	0.60	25	3.60	0.80	baseline	0.00	-0.14	[-0.68; 0.39]
Peng, L, et al., 2022	28	69.05	16.49	29	71.26	17.05	baseline	0.00	-0.13	[-0.65; 0.39]
Qiu, H, et al., 2018 II.	98	76.78	19.91	196	79.49	21.60	baseline	0.00	-0.13	[-0.37; 0.11]
Von Ah, D, et al.,2012 I.	26	22.06	3.44	29	22.38	2.24	baseline	0.00	-0.11	[-0.64; 0.42]
Qiu, H, et al., 2018 I.	98	78.09	18.86	196	79.49	21.60	baseline	0.00	-0.07	[-0.31; 0.18]
Dieng M, et al. 2020	70	0.78	0.20	81	0.77	0.22	baseline	0.00	0.05	[-0.27; 0.37]
Von Ah, D, et al.,2012 II.	27	22.98	3.08	29	22.38	2.24	baseline	0.00	0.22	[-0.30; 0.75]
Peoples, AR, et al., 2016	24	78.90	15.30	24	71.90	19.70	baseline	0.00	0.39	[-0.18; 0.96]
McCaughan, E, et al., 2018	13	85.83	14.10	4	76.88	22.04	baseline	0.00	0.53	[-0.61; 1.67]
Jelvehzadeh, F, et al., 2022	24	4.71	1.07	24	3.94	1.06	baseline	0.00	0.71	[0.13; 1.30]
Von Ah, D, et al.,2012 I.	26	22.15	4.45	29	22.58	2.24	followup	8.00	-0.12	[-0.65; 0.41]
Peng, L, et al., 2022	28	66.67	24.00	29	69.25	22.17	followup	4.00	-0.11	[-0.63; 0.41]
Dieng M, et al. 2020	70	0.05	0.18	81	0.04	0.23	followup	48.00	0.05	[-0.27; 0.37]
Dieng M, et al. 2020	70	0.04	0.16	81	0.03	0.23	followup	26.00	0.05	[-0.27; 0.37]
Peng, L, et al., 2022	28	72.32	22.11	29	71.26	15.26	followup	0.10	0.06	[-0.46; 0.57]
Von Ah, D, et al.,2012 II.	27	22.71	2.08	29	22.58	2.24	followup	8.00	0.06	[-0.47; 0.58]
Qiu, H, et al., 2018 II.	98	80.28	26.77	196	78.03	31.55	followup	4.00	0.07	[-0.17; 0.32]
Lee, JT, et al., 2022	29	4.05	0.60	25	4.00	0.60	followup	12.00	0.08	[-0.45; 0.62]
Lee, JT, et al., 2022	29	4.05	0.60	25	4.00	0.60	followup	24.00	0.08	[-0.45; 0.62]
Qiu, H, et al., 2018 II.	98	89.52	33.46	196	83.21	31.55	followup	12.00	0.20	[-0.05; 0.44]
Qiu, H, et al., 2018 II.	98	94.70	35.69	196	87.72	34.70	followup	24.00	0.20	[-0.04; 0.44]
McCaughan, E, et al., 2018	13	85.76	17.07	4	80.22	22.75	followup	4.00	0.29	[-0.84; 1.41]
Qiu, H, et al., 2018 I.	98	91.32	29.00	196	78.03	31.55	followup	4.00	0.43	[0.19; 0.68]
Peoples, AR, et al., 2016	24	87.90	13.90	24	79.70	15.70	followup	7.00	0.54	[-0.03; 1.12]
Peoples, AR, et al., 2016	24	91.40	15.00	24	78.60	20.60	followup	12.00	0.70	[0.11; 1.28]
Qiu, H, et al., 2018 I.	98	113.18	35.69	196	87.72	34.70	followup	24.00	0.72	[0.48; 0.97]
Qiu, H, et al., 2018 I.	98	107.32	31.23	196	83.21	31.55	followup	12.00	0.76	[0.51; 1.02]
Jelvehzadeh, F, et al., 2022	24	6.05	1.00	24	4.65	0.80	followup	12.00	1.52	[0.87; 2.17]
Jelvehzadeh, F, et al., 2022	24	6.16	1.09	24	4.58	0.92	followup	8.00	1.54	[0.89; 2.19]
van den Berg, SW, et al. 2015 II.	70	73.30	1.78	80	69.88	1.67	followup	4.00	1.97	[1.58; 2.36]
Random effect	1581			2401					0.59	[0.12; 1.06]
Prediction interval										[-0.72; 1.89]
**Random effect**	**3916**			**4491**					**0.66**	**[0.18; 1.14]**
**Prediction interval**										**[-0.63; 1.95]**

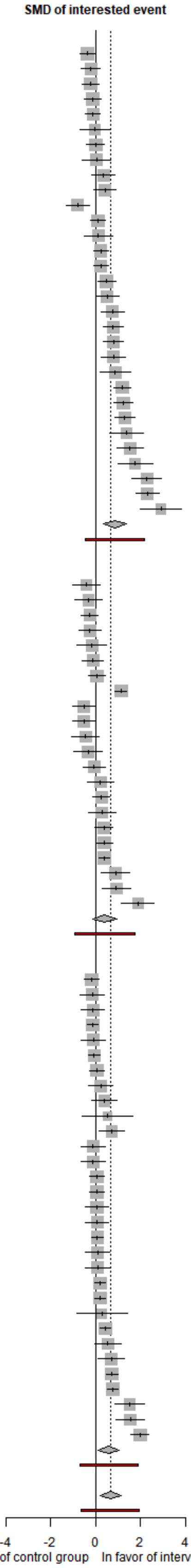

Figure S6.3.Subgroup analysis of the Global QoL Forest plot represents the difference between the intervention vs. control group in the Global QoL domain with the cancer stage subgroups as predicted at week 24 (post-intervention). SMD - Standardized mean difference, CI - confidence interval.

Figure S6.4.T48

Study	Experimental Patient N	Experimental Mean	Experimental SD	Control Patient N	Control Mean	Control SD	follow-up	Follow-up time	SMD	95%-CI
early										
Zhao,X, et al. 2015	62	7.85	2.52	62	8.75	2.38	baseline	0.00	-0.36	[-0.72; -0.01]
Chu, X. et al, 2020	42	65.20	16.77	42	68.91	16.63	baseline	0.00	-0.22	[-0.65; 0.21]
Liu, T, et al., 2019	49	66.61	10.85	53	69.54	15.27	baseline	0.00	-0.22	[-0.61; 0.17]
Zhao, X, et al., 2021	52	53.70	3.14	51	54.69	9.61	baseline	0.00	-0.14	[-0.52; 0.25]
Wang, TJ, et al 2023	70	102.30	18.40	72	104.40	17.10	baseline	0.00	-0.12	[-0.45; 0.21]
Marchioro, G, et al. 1996	18	-58.44	22.39	18	-57.72	20.25	baseline	0.00	-0.03	[-0.69; 0.62]
Walker, LG, et al.,1998	48	3.25	0.70	48	3.25	0.66	baseline	0.00	0.00	[-0.40; 0.40]
Nikbakhsh, N, et al., 2018	20	44.09	8.48	20	43.64	13.73	baseline	0.00	0.04	[-0.58; 0.66]
Yoo, HJ, et al. 2004	30	93.41	16.90	30	87.88	14.96	baseline	0.00	0.34	[-0.17; 0.85]
Cengiz, HO, et al., 2023	32	63.06	19.74	33	55.22	18.26	baseline	0.00	0.41	[-0.08; 0.90]
Beatty, L. et al. 2015	30	56.22	3.88	30	59.37	3.92	followup	6.00	-0.80	[-1.32; -0.27]
Wang, TJ, et al 2023	70	105.40	17.80	72	103.50	20.00	followup	8.00	0.10	[-0.23; 0.43]
Marchioro, G, et al. 1996	18	-54.17	16.90	18	-56.33	17.72	followup	4.00	0.12	[-0.53; 0.78]
Wang, TJ, et al 2023	70	107.30	16.70	72	103.30	19.00	followup	16.00	0.22	[-0.11; 0.55]
Wang, TJ, et al 2023	70	106.90	16.60	72	102.70	20.00	followup	24.00	0.23	[-0.10; 0.56]
Walker, LG, et al.,1998	48	3.29	0.80	48	2.90	0.78	followup	15.00	0.49	[0.08; 0.90]
Yoo, HJ, et al. 2004	30	97.01	18.24	30	88.13	16.18	followup	12.00	0.51	[-0.01; 1.02]
Cengiz, HO, et al., 2023	32	66.98	17.72	33	53.03	18.65	followup	8.00	0.76	[0.25; 1.26]
Chu, X. et al, 2020	42	85.41	16.08	42	71.90	18.50	followup	12.00	0.77	[0.33; 1.22]
Chu, X. et al, 2020	42	84.94	14.59	42	71.59	18.73	followup	8.00	0.79	[0.34; 1.23]
Yoo, HJ, et al. 2004	30	102.00	19.94	30	88.18	14.02	followup	24.00	0.79	[0.26; 1.32]
Marchioro, G, et al. 1996	18	-48.72	12.22	18	-60.78	14.85	followup	12.00	0.87	[0.18; 1.55]
Zhao,X, et al. 2015	62	10.65	2.02	62	8.22	2.07	followup	0.30	1.18	[0.80; 1.56]
Liu, T, et al., 2019	49	62.67	10.32	53	49.47	10.92	followup	9.00	1.23	[0.81; 1.66]
Liu, T, et al., 2019	49	72.33	13.36	53	56.95	10.03	followup	12.00	1.30	[0.87; 1.73]
Marchioro, G, et al. 1996	18	-44.72	9.18	18	-60.78	13.26	followup	24.00	1.38	[0.64; 2.11]
Beatty, L. et al. 2015	30	68.50	3.64	30	62.85	3.64	followup	13.03	1.53	[0.95; 2.11]
Marchioro, G, et al. 1996	18	-41.17	6.91	18	-60.28	13.33	followup	36.00	1.76	[0.98; 2.54]
Beatty, L. et al. 2015	30	74.04	3.78	30	65.27	3.81	followup	26.07	2.28	[1.62; 2.94]
Zhao, X, et al., 2021	52	66.68	3.21	51	55.07	6.32	followup	12.00	2.31	[1.80; 2.81]
Nikbakhsh, N, et al., 2018	20	81.70	10.04	20	47.39	12.93	followup	12.00	2.91	[1.99; 3.82]
Random effect	1251			1271					0.56	[-3.44; 4.57]
Prediction interval										[-4.69; 5.82]
advanced										
Rodin, G, et al., 2019	22	103.92	6.45	20	106.71	6.80	baseline	0.00	-0.41	[-1.03; 0.20]
Serfaty M, et al., 2018	20	56.40	15.50	22	61.40	14.40	baseline	0.00	-0.33	[-0.94; 0.28]
Walczak, A, et al.,2017	61	71.84	16.33	49	75.99	15.72	baseline	0.00	-0.26	[-0.63; 0.12]
Heiney, SP, et al., 2003	33	6.00	1.70	33	6.40	1.40	baseline	0.00	-0.25	[-0.74; 0.23]
Savard, J, et al., 2006	21	42.51	3.19	16	43.11	3.91	baseline	0.00	-0.17	[-0.82; 0.48]
Penedo, FJ, et al., 2007	41	-82.35	14.02	30	-80.57	13.21	baseline	0.00	-0.13	[-0.60; 0.34]
Kissane, DW, et al., 2023	55	25.80	8.30	52	25.30	8.90	baseline	0.00	0.06	[-0.32; 0.44]
Lu, Z, et al., 201	203	61.70	4.00	103	56.30	6.00	baseline	0.00	1.13	[0.88; 1.38]
Penedo, FJ, et al., 2007	41	-87.22	13.82	30	-79.63	14.56	followup	12.00	-0.53	[-1.01; -0.05]
Heiney, SP, et al., 2003	33	6.00	1.50	33	6.80	1.50	followup	16.00	-0.53	[-1.02; -0.04]
Serfaty M, et al., 2018	20	63.80	19.80	22	72.20	16.60	followup	12.00	-0.45	[-1.07; 0.16]
Savard, J, et al., 2006	21	3.62	0.95	16	3.95	0.91	followup	8.00	-0.34	[-1.00; 0.31]
Heiney, SP, et al., 2003	33	6.20	1.30	33	6.30	1.60	followup	6.00	-0.07	[-0.55; 0.41]
Serfaty M, et al., 2018	20	67.70	23.80	22	63.20	16.60	followup	24.00	0.22	[-0.39; 0.82]
Kissane, DW, et al., 2023	55	27.40	7.70	52	25.40	7.80	followup	24.00	0.26	[-0.12; 0.64]
Serfaty M, et al., 2018	20	72.90	19.10	22	67.90	16.70	followup	18.00	0.27	[-0.33; 0.88]
Kissane, DW, et al., 2023	55	27.90	8.10	52	24.80	8.90	followup	12.00	0.36	[-0.02; 0.74]
Walczak, A, et al.,2017	61	77.76	18.80	49	70.87	16.33	followup	4.00	0.39	[0.01; 0.77]
Lu, Z, et al., 201	203	64.40	5.00	103	62.30	6.00	followup	9.00	0.39	[0.15; 0.63]
Rodin, G, et al., 2019	22	110.14	6.83	20	103.57	7.71	followup	4.00	0.89	[0.25; 1.53]
Rodin, G, et al., 2019	22	110.41	7.29	20	103.70	7.00	followup	8.00	0.92	[0.28; 1.56]
Rodin, G, et al., 2019	22	114.37	6.97	20	100.61	7.50	followup	12.00	1.87	[1.13; 2.60]
Random effect	1084			819					0.11	[-3.67; 3.89]
Prediction interval										[-4.79; 5.01]
survivor										
van den Berg, SW, et al. 2015 II.	70	66.79	16.57	80	69.79	17.91	baseline	0.00	-0.17	[-0.49; 0.15]
Lee, JT, et al., 2022	29	3.50	0.60	25	3.60	0.80	baseline	0.00	-0.14	[-0.68; 0.39]
Peng, L, et al., 2022	28	69.05	16.49	29	71.26	17.05	baseline	0.00	-0.13	[-0.65; 0.39]
Qiu, H, et al., 2018 II.	98	76.78	19.91	196	79.49	21.60	baseline	0.00	-0.13	[-0.37; 0.11]
Von Ah, D, et al.,2012 I.	26	22.06	3.44	29	22.38	2.24	baseline	0.00	-0.11	[-0.64; 0.42]
Qiu, H, et al., 2018 I.	98	78.09	18.86	196	79.49	21.60	baseline	0.00	-0.07	[-0.31; 0.18]
Dieng M, et al. 2020	70	0.78	0.20	81	0.77	0.22	baseline	0.00	0.05	[-0.27; 0.37]
Von Ah, D, et al.,2012 II.	27	22.98	3.08	29	22.38	2.24	baseline	0.00	0.22	[-0.30; 0.75]
Peoples, AR, et al., 2016	24	78.90	15.30	24	71.90	19.70	baseline	0.00	0.39	[-0.18; 0.96]
McCaughan, E, et al., 2018	13	85.83	14.10	4	76.88	22.04	baseline	0.00	0.53	[-0.61; 1.67]
Jelvehzadeh, F, et al., 2022	24	4.71	1.07	24	3.94	1.06	baseline	0.00	0.71	[0.13; 1.30]
Von Ah, D, et al.,2012 I.	26	22.15	4.45	29	22.58	2.24	followup	8.00	-0.12	[-0.65; 0.41]
Peng, L, et al., 2022	28	66.67	24.00	29	69.25	22.17	followup	4.00	-0.11	[-0.63; 0.41]
Dieng M, et al. 2020	70	0.05	0.18	81	0.04	0.23	followup	48.00	0.05	[-0.27; 0.37]
Dieng M, et al. 2020	70	0.04	0.16	81	0.03	0.23	followup	26.00	0.05	[-0.27; 0.37]
Peng, L, et al., 2022	28	72.32	22.11	29	71.26	15.26	followup	0.10	0.06	[-0.46; 0.57]
Von Ah, D, et al.,2012 II.	27	22.71	2.08	29	22.58	2.24	followup	8.00	0.06	[-0.47; 0.58]
Qiu, H, et al., 2018 II.	98	80.28	26.77	196	78.03	31.55	followup	4.00	0.07	[-0.17; 0.32]
Lee, JT, et al., 2022	29	4.05	0.60	25	4.00	0.60	followup	12.00	0.08	[-0.45; 0.62]
Lee, JT, et al., 2022	29	4.05	0.60	25	4.00	0.60	followup	24.00	0.08	[-0.45; 0.62]
Qiu, H, et al., 2018 II.	98	89.52	33.46	196	83.21	31.55	followup	12.00	0.20	[-0.05; 0.44]
Qiu, H, et al., 2018 II.	98	94.70	35.69	196	87.72	34.70	followup	24.00	0.20	[-0.04; 0.44]
McCaughan, E, et al., 2018	13	85.76	17.07	4	80.22	22.75	followup	4.00	0.29	[-0.84; 1.41]
Qiu, H, et al., 2018 I.	98	91.32	29.00	196	78.03	31.55	followup	4.00	0.43	[0.19; 0.68]
Peoples, AR, et al., 2016	24	87.90	13.90	24	79.70	15.70	followup	7.00	0.54	[-0.03; 1.12]
Peoples, AR, et al., 2016	24	91.40	15.00	24	78.60	20.60	followup	12.00	0.70	[0.11; 1.28]
Qiu, H, et al., 2018 I.	98	113.18	35.69	196	87.72	34.70	followup	24.00	0.72	[0.48; 0.97]
Qiu, H, et al., 2018 I.	98	107.32	31.23	196	83.21	31.55	followup	12.00	0.76	[0.51; 1.02]
Jelvehzadeh, F, et al., 2022	24	6.05	1.00	24	4.65	0.80	followup	12.00	1.52	[0.87; 2.17]
Jelvehzadeh, F, et al., 2022	24	6.16	1.09	24	4.58	0.92	followup	8.00	1.54	[0.89; 2.19]
van den Berg, SW, et al. 2015 II.	70	73.30	1.78	80	69.88	1.67	followup	4.00	1.97	[1.58; 2.36]
Random effect	1581			2401					0.30	[-4.75; 5.34]
Prediction interval										[-6.31; 6.90]
**Random effect**	**3916**			**4491**					**0.35**	**[-5.65; 6.36]**
**Prediction interval**										**[-7.01; 7.71]**

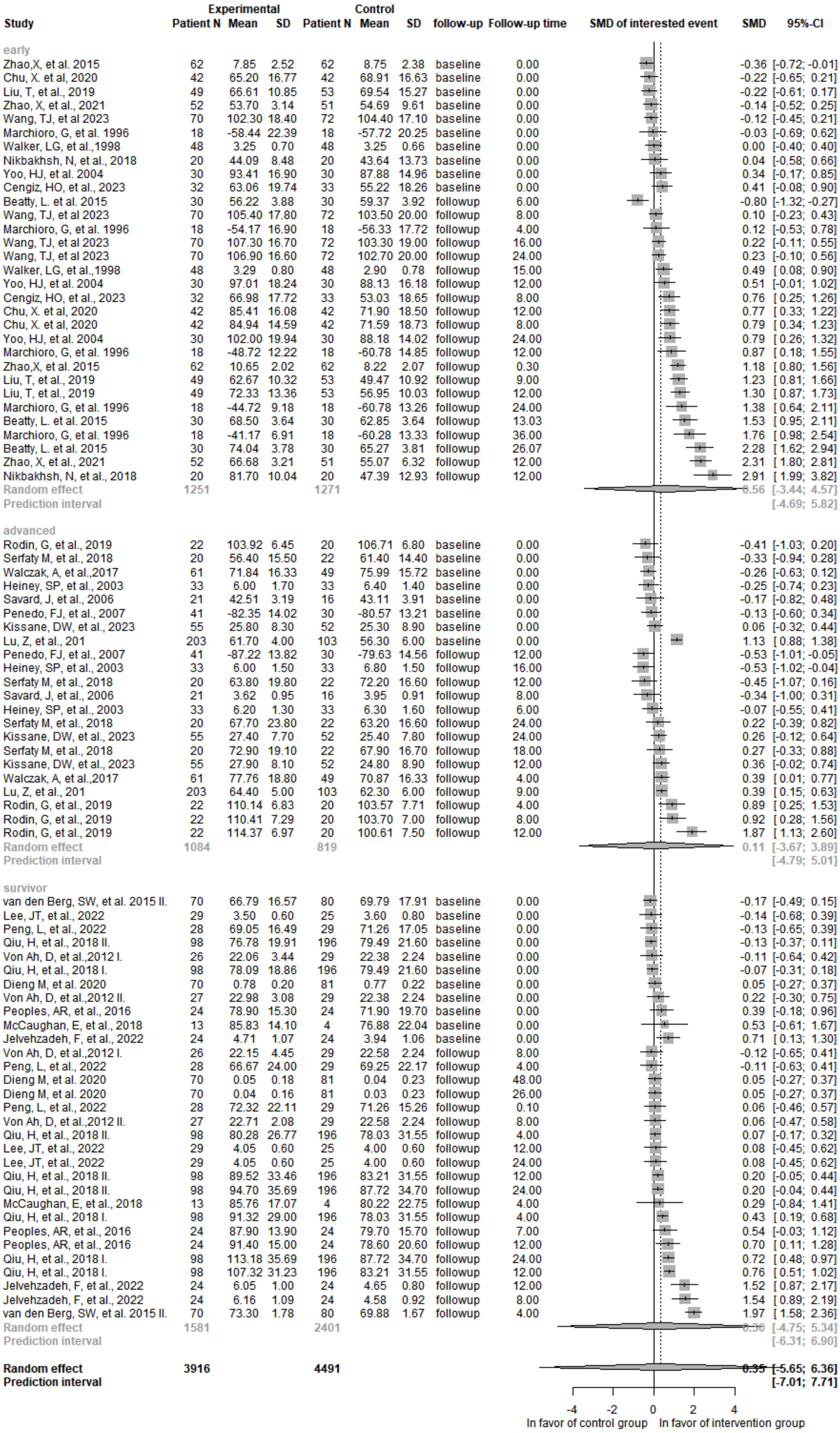

Figure S6.4.Subgroup analysis of the Global QoL Forest plot represents the difference between the intervention vs. control group in the Global QoL domain with the cancer stage subgroups as predicted at week 48 (post-intervention). SMD - Standardized mean difference, CI - confidence interval.

S7. Subgroup analysis of Global QoL: Cancer type

Figure S7.1.T0

Study	Experimental Patient N	Experimental Mean	Experimental SD	Control Patient N	Control Mean	Control SD	follow-up	Follow-up time	SMD	95%-CI
**breast**										
Garssen B, et al., 2012	34	74.40	4.20	36	76.50	4.30	baseline	0.00	-0.49	[-0.96; -0.01]
Seliniotaki, T, et al., 2021	27	62.00	33.80	26	72.20	16.20	baseline	0.00	-0.38	[-0.92; 0.17]
Elyasi, F, et al., 2021 I.	15	3.30	1.90	15	3.80	1.50	baseline	0.00	-0.28	[-1.00; 0.44]
Heiney, SP, et al., 2003	33	6.00	1.70	33	6.40	1.40	baseline	0.00	-0.25	[-0.74; 0.23]
Chu, X. et al, 2020	42	65.20	16.77	42	68.91	16.63	baseline	0.00	-0.22	[-0.65; 0.21]
Elyasi, F, et al., 2021 II.	20	3.50	1.80	20	3.80	1.50	baseline	0.00	-0.18	[-0.80; 0.44]
van den Berg, SW, et al. 2015 II.	70	66.79	16.57	80	69.79	17.91	baseline	0.00	-0.17	[-0.49; 0.15]
Savard, J, et al., 2006	21	42.51	3.19	16	43.11	3.91	baseline	0.00	-0.17	[-0.82; 0.48]
Nápoles AM, et al. 2015	76	66.46	16.92	75	68.83	15.33	baseline	0.00	-0.15	[-0.47; 0.17]
Dirksen, S. et al, 2007	34	83.30	11.90	38	84.80	9.20	baseline	0.00	-0.14	[-0.60; 0.32]
Peng, L, et al., 2022	28	69.05	16.49	29	71.26	17.05	baseline	0.00	-0.13	[-0.65; 0.39]
Qiu, H, et al., 2018 II.	98	76.78	19.91	196	79.49	21.60	baseline	0.00	-0.13	[-0.37; 0.11]
Von Ah, D, et al.,2012 I.	26	22.06	3.44	29	22.38	2.24	baseline	0.00	-0.11	[-0.64; 0.42]
Schellekens, MPJ, et al. 2016	69	95.43	21.60	70	97.09	23.54	baseline	0.00	-0.07	[-0.41; 0.26]
Reese, JB, et al., 2021	73	60.80	16.70	71	61.90	14.30	baseline	0.00	-0.07	[-0.40; 0.26]
Qiu, H, et al., 2018 I.	98	78.09	18.86	196	79.49	21.60	baseline	0.00	-0.07	[-0.31; 0.18]
Marchioro, G, et al. 1996	18	-58.44	22.39	18	-57.72	20.25	baseline	0.00	-0.03	[-0.69; 0.62]
Reich, RR, et al. 2016	167	62.44	27.52	155	62.74	24.68	baseline	0.00	-0.01	[-0.23; 0.21]
Hoffman, CJ, et al., 2012	101	96.57	17.22	106	96.68	21.05	baseline	0.00	-0.01	[-0.28; 0.27]
Walker, LG, et al.,1998	48	3.25	0.70	48	3.25	0.66	baseline	0.00	0.00	[-0.40; 0.40]
Rosen, KD, et al., 2018	57	93.61	22.68	55	92.96	25.12	baseline	0.00	0.03	[-0.34; 0.40]
Nikbakhsh, N, et al., 2018	20	44.09	8.48	20	43.64	13.73	baseline	0.00	0.04	[-0.58; 0.66]
Esplen MJ, et al., 2018	131	70.50	16.50	63	68.90	17.20	baseline	0.00	0.10	[-0.21; 0.40]
Klinkhammer-Schalke, M et al., 2012	100	54.35	26.09	100	51.45	23.19	baseline	0.00	0.12	[-0.16; 0.39]
Von Ah, D, et al.,2012 II.	27	22.98	3.08	29	22.38	2.24	baseline	0.00	0.22	[-0.30; 0.75]
Rahmani, S, et al. 2015	12	41.66	6.15	12	39.58	7.22	baseline	0.00	0.30	[-0.51; 1.10]
Yoo, HJ, et al. 2004	30	93.41	16.90	30	87.88	14.96	baseline	0.00	0.34	[-0.17; 0.85]
Cengiz, HO, et al., 2023	32	63.06	19.74	33	55.22	18.26	baseline	0.00	0.41	[-0.08; 0.90]
Gaston-Johansson, F, et al., 2011	38	22.60	4.30	35	20.60	4.00	baseline	0.00	0.48	[0.01; 0.94]
Jelvehzadeh, F, et al., 2022	24	4.71	1.07	24	3.94	1.06	baseline	0.00	0.71	[0.13; 1.30]
Heiney, SP, et al., 2003	33	6.00	1.50	33	6.80	1.50	followup	16.00	-0.53	[-1.02; -0.04]
Savard, J, et al., 2006	21	3.62	0.95	16	3.95	0.91	followup	8.00	-0.34	[-1.00; 0.31]
Von Ah, D, et al.,2012 I.	26	22.15	4.45	29	22.58	2.24	followup	8.00	-0.12	[-0.65; 0.41]
Peng, L, et al., 2022	28	66.67	24.00	29	69.25	22.17	followup	4.00	-0.11	[-0.63; 0.41]
Reich, RR, et al. 2016	167	68.43	27.76	155	70.36	22.70	followup	12.00	-0.08	[-0.29; 0.14]
Heiney, SP, et al., 2003	33	6.20	1.30	33	6.30	1.60	followup	6.00	-0.07	[-0.55; 0.41]
Klinkhammer-Schalke, M et al., 2012	100	53.62	22.46	100	55.07	20.29	followup	12.00	-0.07	[-0.34; 0.21]
Esplen MJ, et al., 2018	131	73.40	16.70	63	74.30	17.70	followup	48.00	-0.05	[-0.35; 0.25]
Reese, JB, et al., 2021	73	61.80	18.20	71	62.60	13.90	followup	8.00	-0.05	[-0.38; 0.28]
Reich, RR, et al. 2016	167	65.76	26.18	155	66.24	24.76	followup	6.00	-0.02	[-0.24; 0.20]
Rahmani, S, et al. 2015	12	12.00	61.11	12	12.00	58.33	followup	16.00	0.00	[-0.80; 0.80]
Seliniotaki, T, et al., 2021	27	65.00	28.00	26	64.00	20.50	followup	8.00	0.04	[-0.50; 0.58]
Peng, L, et al., 2022	28	72.32	22.11	29	71.26	15.26	followup	0.10	0.06	[-0.46; 0.57]
Von Ah, D, et al.,2012 II.	27	22.71	2.08	29	22.58	2.24	followup	8.00	0.06	[-0.47; 0.58]
Qiu, H, et al., 2018 II.	98	80.28	26.77	196	78.03	31.55	followup	4.00	0.07	[-0.17; 0.32]
Klinkhammer-Schalke, M et al., 2012	100	64.13	23.56	100	62.32	21.38	followup	48.00	0.08	[-0.20; 0.36]
Esplen MJ, et al., 2018	131	14.90	9.40	63	14.00	9.40	followup	8.00	0.10	[-0.21; 0.40]
Marchioro, G, et al. 1996	18	-54.17	16.90	18	-56.33	17.72	followup	4.00	0.12	[-0.53; 0.78]
Esplen MJ, et al., 2018	131	72.60	16.60	63	70.30	18.00	followup	24.00	0.13	[-0.17; 0.44]
Klinkhammer-Schalke, M et al., 2012	100	65.21	22.09	100	61.23	23.55	followup	36.00	0.17	[-0.10; 0.45]
Elyasi, F, et al., 2021 I.	15	4.30	1.00	15	4.10	1.10	followup	24.00	0.19	[-0.53; 0.90]
Nápoles AM, et al. 2015	76	77.24	15.13	75	74.39	15.34	followup	12.00	0.19	[-0.13; 0.51]
Elyasi, F, et al., 2021 II.	20	4.30	1.00	20	4.10	1.10	followup	24.00	0.19	[-0.43; 0.81]
Schellekens, MPJ, et al. 2016	69	107.36	18.80	70	103.34	22.35	followup	8.00	0.19	[-0.14; 0.53]
Qiu, H, et al., 2018 II.	98	89.52	33.46	196	83.21	31.55	followup	12.00	0.20	[-0.05; 0.44]
Qiu, H, et al., 2018 II.	98	94.70	35.69	196	87.72	34.70	followup	24.00	0.20	[-0.04; 0.44]
Nápoles AM, et al. 2015	76	80.64	13.64	75	77.02	15.62	followup	24.00	0.25	[-0.07; 0.57]
Dirksen, S. et al, 2007	34	91.60	15.00	38	87.70	14.70	followup	10.00	0.26	[-0.20; 0.72]
Hoffman, CJ, et al., 2012	101	103.56	17.91	106	96.84	21.14	followup	8.00	0.34	[0.07; 0.62]
Klinkhammer-Schalke, M et al., 2012	100	63.41	22.11	100	55.07	23.19	followup	24.00	0.37	[0.09; 0.65]
Hoffman, CJ, et al., 2012	101	103.78	17.85	106	96.22	19.43	followup	12.00	0.40	[0.13; 0.68]
Qiu, H, et al., 2018 I.	98	91.32	29.00	196	78.03	31.55	followup	4.00	0.43	[0.19; 0.68]
Walker, LG, et al.,1998	48	3.29	0.80	48	2.90	0.78	followup	15.00	0.49	[0.08; 0.90]
Yoo, HJ, et al. 2004	30	97.01	18.24	30	88.13	16.18	followup	12.00	0.51	[-0.01; 1.02]
Rosen, KD, et al., 2018	57	107.35	17.79	55	96.04	23.96	followup	5.00	0.53	[0.16; 0.91]
Gaston-Johansson, F, et al., 2011	38	24.70	4.30	35	21.90	5.00	followup	48.00	0.60	[0.13; 1.07]
Garssen B, et al., 2012	34	78.40	3.70	36	76.40	2.90	followup	5.00	0.60	[0.12; 1.08]
Garssen B, et al., 2012	34	80.40	3.30	36	78.20	3.20	followup	2.00	0.67	[0.19; 1.15]
Garssen B, et al., 2012	34	75.50	3.60	36	72.90	3.80	followup	13.00	0.69	[0.21; 1.18]
Qiu, H, et al., 2018 I.	98	113.18	35.69	196	87.72	34.70	followup	24.00	0.72	[0.48; 0.97]
Cengiz, HO, et al., 2023	32	66.98	17.72	33	53.03	18.65	followup	8.00	0.76	[0.25; 1.26]
Qiu, H, et al., 2018 I.	98	107.32	31.23	196	83.21	31.55	followup	12.00	0.76	[0.51; 1.02]
Chu, X. et al, 2020	42	85.41	16.08	42	71.90	18.50	followup	12.00	0.77	[0.33; 1.22]
Chu, X. et al, 2020	42	84.94	14.59	42	71.59	18.73	followup	8.00	0.79	[0.34; 1.23]
Yoo, HJ, et al. 2004	30	102.00	19.94	30	88.18	14.02	followup	24.00	0.79	[0.26; 1.32]
Garssen B, et al., 2012	34	81.00	3.40	36	78.20	3.20	followup	4.00	0.84	[0.35; 1.33]
Marchioro, G, et al. 1996	18	-48.72	12.22	18	-60.78	14.85	followup	12.00	0.87	[0.18; 1.55]
Rahmani, S, et al. 2015	12	66.66	9.40	12	54.16	10.95	followup	8.00	1.18	[0.30; 2.06]
Marchioro, G, et al. 1996	18	-44.72	9.18	18	-60.78	13.26	followup	24.00	1.38	[0.64; 2.11]
Jelvehzadeh, F, et al., 2022	24	6.05	1.00	24	4.65	0.80	followup	12.00	1.52	[0.87; 2.17]
Jelvehzadeh, F, et al., 2022	24	6.16	1.09	24	4.58	0.92	followup	8.00	1.54	[0.89; 2.19]
Marchioro, G, et al. 1996	18	-41.17	6.91	18	-60.28	13.33	followup	36.00	1.76	[0.98; 2.54]
van den Berg, SW, et al. 2015 II.	70	73.30	1.78	80	69.88	1.67	followup	4.00	1.97	[1.58; 2.36]
Nikbakhsh, N, et al., 2018	20	81.70	10.04	20	47.39	12.93	followup	12.00	2.91	[1.99; 3.82]
Random effect	4761			5278					0.53	[0.18; 0.87]
Prediction interval										[-0.47; 1.52]
**gastroenterological**										
Li, X, et al., 2017	102	56.32	7.80	108	62.38	11.46	baseline	0.00	-0.61	[-0.89; -0.34]
Wang, TJ, et al 2023	70	102.30	18.40	72	104.40	17.10	baseline	0.00	-0.12	[-0.45; 0.21]
Xia, S, et al., 2023	80	61.40	16.60	80	61.10	15.70	baseline	0.00	0.02	[-0.29; 0.33]
Gao Q, et al. 2020	40	-68.20	8.25	40	-68.44	8.82	baseline	0.00	0.03	[-0.41; 0.47]
Cheung YL, et al. 2002	29	4.96	1.74	30	4.36	1.62	baseline	0.00	0.35	[-0.16; 0.87]
Wang, TJ, et al 2023	70	105.40	17.80	72	103.50	20.00	followup	8.00	0.10	[-0.23; 0.43]
Xia, S, et al., 2023	80	67.70	16.20	80	65.80	14.40	followup	4.00	0.12	[-0.19; 0.43]
Wang, TJ, et al 2023	70	107.30	16.70	72	103.30	19.00	followup	16.00	0.22	[-0.11; 0.55]
Wang, TJ, et al 2023	70	106.90	16.60	72	102.70	20.00	followup	24.00	0.23	[-0.10; 0.56]
Xia, S, et al., 2023	80	72.70	15.80	80	68.00	13.50	followup	12.00	0.32	[0.01; 0.63]
Xia, S, et al., 2023	80	78.00	14.30	80	72.90	13.40	followup	24.00	0.37	[0.05; 0.68]
Zhang, J, et al., 2021	61	9.67	3.71	61	8.21	3.02	followup	12.00	0.43	[0.07; 0.79]
Gao Q, et al. 2020	40	-54.26	7.03	40	-60.17	7.28	followup	4.00	0.82	[0.36; 1.27]
Li, X, et al., 2017	102	76.58	9.06	108	64.28	12.49	followup	12.00	1.12	[0.83; 1.41]
Cheung YL, et al. 2002	29	7.48	1.12	30	5.56	0.50	followup	10.00	2.20	[1.54; 2.85]
Cheung YL, et al. 2002	29	7.51	0.98	30	4.96	0.66	followup	5.00	3.02	[2.26; 3.78]
Random effect	1032			1055					0.71	[-0.00; 1.42]
Prediction interval										[-0.55; 1.97]
**gynaecological**										
Sandsund, C, et al., 2017	72	64.20	21.80	70	66.70	19.70	baseline	0.00	-0.12	[-0.45; 0.21]
Chan, et al. 2005	80	58.29	50.71	75	60.44	40.96	baseline	0.00	-0.05	[-0.36; 0.27]
Powell, CB, et al., 2008	21	78.30	17.70	43	75.60	17.90	baseline	0.00	0.15	[-0.37; 0.67]
Chan, et al. 2005	80	60.31	41.32	75	66.46	47.98	followup	12.00	-0.14	[-0.45; 0.18]
Sandsund, C, et al., 2017	72	67.50	22.60	70	69.40	21.10	followup	24.00	-0.09	[-0.42; 0.24]
Chan, et al. 2005	80	71.69	74.33	75	77.23	63.91	followup	72.00	-0.08	[-0.39; 0.24]
Chan, et al. 2005	80	71.61	51.52	75	75.69	53.26	followup	60.00	-0.08	[-0.39; 0.24]
Sandsund, C, et al., 2017	72	66.20	19.10	70	67.30	22.00	followup	12.00	-0.05	[-0.38; 0.28]
Chan, et al. 2005	80	73.13	34.88	75	73.75	47.11	followup	36.00	-0.01	[-0.33; 0.30]
Chan, et al. 2005	80	67.38	46.78	75	66.55	51.44	followup	24.00	0.02	[-0.30; 0.33]
Chan, et al. 2005	80	77.86	49.64	75	76.04	45.64	followup	48.00	0.04	[-0.28; 0.35]
Powell, CB, et al., 2008	21	80.40	26.30	43	77.10	21.20	followup	12.00	0.14	[-0.38; 0.66]
Random effect	818			821					0.27	[-0.31; 0.85]
Prediction interval										[-1.27; 1.80]
**prostate**										
Penedo, FJ, et al., 2007	41	-82.35	14.02	30	-80.57	13.21	baseline	0.00	-0.13	[-0.60; 0.34]
Northouse, LL, et al., 2015	17	81.30	3.88	19	79.63	2.96	baseline	0.00	0.48	[-0.19; 1.14]
McCaughan, E, et al., 2018	13	85.83	14.10	4	76.88	22.04	baseline	0.00	0.53	[-0.61; 1.67]
Penedo, FJ, et al., 2007	41	-87.22	13.82	30	-79.63	14.56	followup	12.00	-0.53	[-1.01; -0.05]
Northhouse, LL, et al., 2007	107	87.00	10.80	121	86.90	10.60	followup	32.00	0.01	[-0.25; 0.27]
Northhouse, LL, et al., 2007	104	86.10	10.90	114	85.80	10.70	followup	48.00	0.03	[-0.24; 0.29]
Northhouse, LL, et al., 2007	112	87.20	10.60	123	85.50	10.30	followup	16.00	0.16	[-0.09; 0.42]
McCaughan, E, et al., 2018	13	85.76	17.07	4	80.22	22.75	followup	4.00	0.29	[-0.84; 1.41]
Northouse, LL, et al., 2015	17	88.51	3.03	19	76.94	3.44	followup	2.00	3.48	[2.41; 4.55]
Random effect	465			464					0.54	[-0.68; 1.76]
Prediction interval										[-1.20; 2.28]
**Random effect**	**7076**			**7618**					**0.54**	**[0.17; 0.91]**
**Prediction interval**										**[-0.45; 1.54]**

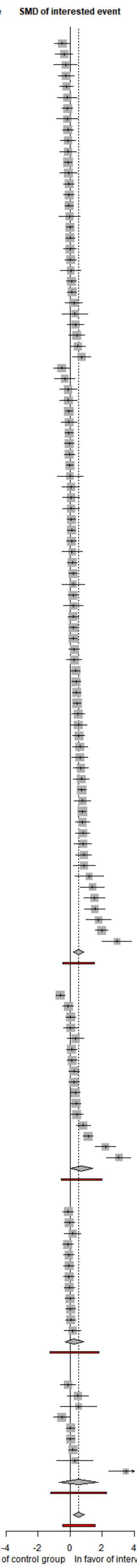

Figure S7.1. Subgroup analysis of the Global QoL. Forest plot represents the difference between the intervention vs. control group in the Global QoL domain with the cancer type subgroups as predicted at week 0 (post-intervention). SMD - Standardized mean difference, CI - confidence interval.

Figure S7.2.T12

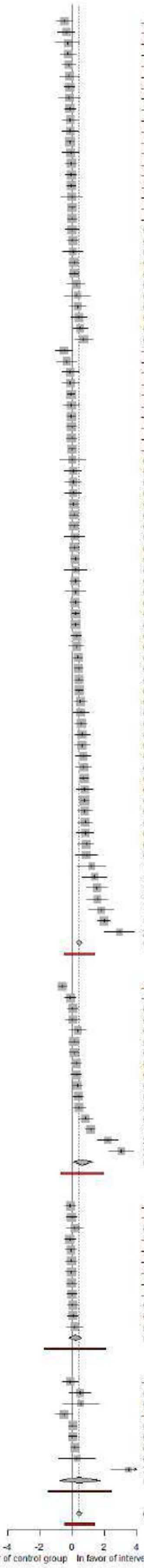

Study	Experimental Patient N	Experimental Mean	Experimental SD	Control Patient N	Control Mean	Control SD	follow-up	Follow-up time	SMD	95%-CI
breast										
Garssen B, et al., 2012	34	74.40	4.20	36	76.50	4.30	baseline	0.00	-0.49	[-0.96; -0.01]
Seliniotaki, T, et al., 2021	27	62.00	33.80	26	72.20	16.20	baseline	0.00	-0.38	[-0.92; 0.17]
Elyasi, F, et al., 2021 I.	15	3.30	1.90	15	3.80	1.50	baseline	0.00	-0.28	[-1.00; 0.44]
Heiney, SP, et al., 2003	33	6.00	1.70	33	6.40	1.40	baseline	0.00	-0.25	[-0.74; 0.23]
Chu, X. et al, 2020	42	65.20	16.77	42	68.91	16.63	baseline	0.00	-0.22	[-0.65; 0.21]
Elyasi, F, et al., 2021 II.	20	3.50	1.80	20	3.80	1.50	baseline	0.00	-0.18	[-0.80; 0.44]
van den Berg, SW, et al. 2015 II.	70	66.79	16.57	80	69.79	17.91	baseline	0.00	-0.17	[-0.49; 0.15]
Savard, J, et al., 2006	21	42.51	3.19	16	43.11	3.91	baseline	0.00	-0.17	[-0.82; 0.48]
Nápoles AM, et al. 2015	76	66.46	16.92	75	68.83	15.33	baseline	0.00	-0.15	[-0.47; 0.17]
Dirksen, S. et al, 2007	34	83.30	11.90	38	84.80	9.20	baseline	0.00	-0.14	[-0.60; 0.32]
Peng, L, et al., 2022	28	69.05	16.49	29	71.26	17.05	baseline	0.00	-0.13	[-0.65; 0.39]
Qiu, H, et al., 2018 II.	98	76.78	19.91	196	79.49	21.60	baseline	0.00	-0.13	[-0.37; 0.11]
Von Ah, D, et al.,2012 I.	26	22.06	3.44	29	22.38	2.24	baseline	0.00	-0.11	[-0.64; 0.42]
Schellekens, MPJ, et al. 2016	69	95.43	21.60	70	97.09	23.54	baseline	0.00	-0.07	[-0.41; 0.26]
Reese, JB, et al., 2021	73	60.80	15.70	71	61.90	14.30	baseline	0.00	-0.07	[-0.40; 0.26]
Qiu, H, et al., 2018 I.	98	78.09	18.86	196	79.49	21.60	baseline	0.00	-0.07	[-0.31; 0.18]
Marchioro, G, et al. 1996	18	-58.44	22.39	18	-57.72	20.25	baseline	0.00	-0.03	[-0.69; 0.62]
Reich, RR, et al. 2016	167	62.44	27.52	155	62.74	24.68	baseline	0.00	-0.01	[-0.23; 0.21]
Hoffman, CJ, et al., 2012	101	96.57	17.22	106	96.68	21.05	baseline	0.00	-0.01	[-0.28; 0.27]
Walker, LG, et al.,1998	48	3.25	0.70	48	3.25	0.66	baseline	0.00	0.00	[-0.40; 0.40]
Rosen, KD, et al., 2018	57	93.61	22.68	55	92.96	25.12	baseline	0.00	0.03	[-0.34; 0.40]
Nikbakhsh, N, et al., 2018	20	44.09	8.48	20	43.64	13.73	baseline	0.00	0.04	[-0.58; 0.66]
Esplen MJ, et al., 2018	131	70.50	16.50	63	68.90	17.20	baseline	0.00	0.10	[-0.21; 0.40]
Klinkhammer-Schalke, M et al., 2012	100	54.35	26.09	100	51.45	23.19	baseline	0.00	0.12	[-0.16; 0.39]
Von Ah, D, et al.,2012 II.	27	22.98	3.08	29	22.38	2.24	baseline	0.00	0.22	[-0.30; 0.75]
Rahmani, S, et al. 2015	12	41.66	6.15	12	39.58	7.22	baseline	0.00	0.30	[-0.51; 1.10]
Yoo, HJ, et al. 2004	30	93.41	16.90	30	87.88	14.96	baseline	0.00	0.34	[-0.17; 0.85]
Cengiz, HO, et al., 2023	32	63.06	19.74	33	55.22	18.26	baseline	0.00	0.41	[-0.08; 0.90]
Gaston-Johansson, F, et al., 2011	38	22.60	4.30	35	20.60	4.00	baseline	0.00	0.48	[0.01; 0.94]
Jelvehzadeh, F, et al., 2022	24	4.71	1.07	24	3.94	1.06	baseline	0.00	0.71	[0.13; 1.30]
Heiney, SP, et al., 2003	33	6.00	1.50	33	6.80	1.50	followup	16.00	-0.53	[-1.02; -0.04]
Savard, J, et al., 2006	21	3.62	0.95	16	3.95	0.91	followup	8.00	-0.34	[-1.00; 0.31]
Von Ah, D, et al.,2012 I.	26	22.15	4.45	29	22.58	2.24	followup	8.00	-0.12	[-0.65; 0.41]
Peng, L, et al., 2022	28	66.67	24.00	29	69.25	22.17	followup	4.00	-0.11	[-0.63; 0.41]
Reich, RR, et al. 2016	167	68.43	27.76	155	70.36	22.70	followup	12.00	-0.08	[-0.29; 0.14]
Heiney, SP, et al., 2003	33	6.20	1.30	33	6.30	1.60	followup	6.00	-0.07	[-0.55; 0.41]
Klinkhammer-Schalke, M et al., 2012	100	53.62	22.45	100	55.07	20.29	followup	12.00	-0.07	[-0.34; 0.21]
Esplen MJ, et al., 2018	131	73.40	16.70	63	74.30	17.70	followup	48.00	-0.05	[-0.35; 0.25]
Reese, JB, et al., 2021	73	61.80	18.20	71	62.60	13.90	followup	8.00	-0.05	[-0.36; 0.28]
Reich, RR, et al. 2016	167	65.76	26.18	155	66.24	24.76	followup	6.00	-0.02	[-0.24; 0.20]
Rahmani, S, et al. 2015	12	12.00	61.11	12	12.00	58.33	followup	16.00	0.00	[-0.80; 0.80]
Seliniotaki, T, et al., 2021	27	65.00	28.00	26	64.00	20.50	followup	8.00	0.04	[-0.50; 0.58]
Peng, L, et al., 2022	28	72.32	22.11	29	71.26	15.26	followup	0.10	0.06	[-0.46; 0.57]
Von Ah, D, et al.,2012 II.	27	22.71	2.08	29	22.58	2.24	followup	8.00	0.06	[-0.47; 0.58]
Qiu, H, et al., 2018 II.	98	80.28	26.77	196	78.03	31.55	followup	4.00	0.07	[-0.17; 0.32]
Klinkhammer-Schalke, M et al., 2012	100	64.13	23.56	100	62.32	21.38	followup	48.00	0.08	[-0.20; 0.36]
Esplen MJ, et al., 2018	131	14.90	9.40	63	14.00	9.40	followup	8.00	0.10	[-0.21; 0.40]
Marchioro, G, et al. 1996	18	-54.17	16.90	18	-56.33	17.72	followup	4.00	0.12	[-0.53; 0.78]
Esplen MJ, et al., 2018	131	72.60	16.60	63	70.30	18.00	followup	24.00	0.13	[-0.17; 0.44]
Klinkhammer-Schalke, M et al., 2012	100	65.21	22.09	100	61.23	23.55	followup	36.00	0.17	[-0.10; 0.45]
Elyasi, F, et al., 2021 I.	15	4.30	1.00	15	4.10	1.10	followup	24.00	0.19	[-0.53; 0.90]
Nápoles AM, et al. 2015	76	77.24	15.13	75	74.39	15.34	followup	12.00	0.19	[-0.13; 0.51]
Elyasi, F, et al., 2021 II.	20	4.30	1.00	20	4.10	1.10	followup	24.00	0.19	[-0.43; 0.81]
Schellekens, MPJ, et al. 2016	69	107.36	18.80	70	103.34	22.35	followup	8.00	0.19	[-0.14; 0.53]
Qiu, H, et al., 2018 II.	98	89.52	33.46	196	83.21	31.55	followup	12.00	0.20	[-0.05; 0.44]
Qiu, H, et al., 2018 II.	98	94.70	35.69	196	87.72	34.70	followup	24.00	0.20	[-0.04; 0.44]
Nápoles AM, et al. 2015	76	80.64	13.84	75	77.02	15.62	followup	24.00	0.25	[-0.07; 0.57]
Dirksen, S. et al, 2007	34	91.60	15.00	38	87.70	14.70	followup	10.00	0.26	[-0.20; 0.72]
Hoffman, CJ, et al., 2012	101	103.56	17.91	106	96.84	21.14	followup	8.00	0.34	[0.07; 0.62]
Klinkhammer-Schalke, M et al., 2012	100	63.41	22.11	100	55.07	23.19	followup	24.00	0.37	[0.09; 0.65]
Hoffman, CJ, et al., 2012	101	103.78	17.85	106	96.22	19.43	followup	12.00	0.40	[0.13; 0.68]
Qiu, H, et al., 2018 I.	98	91.32	29.00	196	78.03	31.55	followup	4.00	0.43	[0.19; 0.68]
Walker, LG, et al.,1998	48	3.29	0.80	48	2.90	0.78	followup	15.00	0.49	[0.08; 0.90]
Yoo, HJ, et al. 2004	30	97.01	18.24	30	88.13	16.18	followup	12.00	0.51	[-0.01; 1.02]
Rosen, KD, et al., 2018	57	107.35	17.79	55	96.04	23.96	followup	5.00	0.53	[0.16; 0.91]
Gaston-Johansson, F, et al., 2011	38	24.70	4.30	35	21.90	5.00	followup	48.00	0.60	[0.13; 1.07]
Garssen B, et al., 2012	34	78.40	3.70	36	76.40	2.90	followup	5.00	0.60	[0.12; 1.08]
Garssen B, et al., 2012	34	80.40	3.30	36	78.20	3.20	followup	2.00	0.67	[0.19; 1.15]
Garssen B, et al., 2012	34	75.50	3.60	36	72.90	3.80	followup	13.00	0.69	[0.21; 1.18]
Qiu, H, et al., 2018 I.	98	113.18	35.69	196	87.72	34.70	followup	24.00	0.72	[0.48; 0.97]
Cengiz, HO, et al., 2023	32	66.98	17.72	33	53.03	18.65	followup	8.00	0.76	[0.25; 1.26]
Qiu, H, et al., 2018 I.	98	107.32	31.23	196	83.21	31.55	followup	12.00	0.76	[0.51; 1.02]
Chu, X. et al, 2020	42	85.41	16.08	42	71.90	18.50	followup	12.00	0.77	[0.33; 1.22]
Chu, X. et al, 2020	42	84.94	14.59	42	71.59	18.73	followup	8.00	0.79	[0.34; 1.23]
Yoo, HJ, et al. 2004	30	102.00	19.94	30	88.18	14.02	followup	24.00	0.79	[0.26; 1.32]
Garssen B, et al., 2012	34	81.00	3.40	36	78.20	3.20	followup	4.00	0.84	[0.35; 1.33]
Marchioro, G, et al. 1996	18	-48.72	12.22	18	-60.78	14.85	followup	12.00	0.87	[0.18; 1.55]
Rahmani, S, et al. 2015	12	66.66	9.40	12	54.16	10.95	followup	8.00	1.18	[0.30; 2.06]
Marchioro, G, et al. 1996	18	-44.72	9.18	18	-60.78	13.26	followup	24.00	1.38	[0.64; 2.11]
Jelvehzadeh, F, et al., 2022	24	6.05	1.00	24	4.65	0.80	followup	12.00	1.52	[0.87; 2.17]
Jelvehzadeh, F, et al., 2022	24	6.16	1.09	24	4.58	0.92	followup	8.00	1.54	[0.89; 2.19]
Marchioro, G, et al. 1996	18	-41.17	6.91	18	-60.28	13.33	followup	36.00	1.76	[0.98; 2.54]
van den Berg, SW, et al. 2015 II.	70	73.30	1.78	80	69.88	1.67	followup	4.00	1.97	[1.58; 2.36]
Nikbakhsh, N, et al., 2018	20	81.70	10.04	20	47.39	12.93	followup	12.00	2.91	[1.99; 3.82]
Random effect	4761			5278					0.44	[0.26; 0.63]
Prediction interval										[-0.49; 1.38]
gastroenterological										
Li, X, et al., 2017	102	56.32	7.80	108	62.38	11.46	baseline	0.00	-0.61	[-0.89; -0.34]
Wang, TJ, et al 2023	70	102.30	18.40	72	104.40	17.10	baseline	0.00	-0.12	[-0.45; 0.21]
Xia, S, et al., 2023	80	61.40	16.60	80	61.10	15.70	baseline	0.00	0.02	[-0.29; 0.33]
Gao Q, et al. 2020	40	-58.20	8.25	40	-58.44	8.82	baseline	0.00	0.03	[-0.41; 0.47]
Cheung YL, et al. 2002	29	4.96	1.74	30	4.36	1.62	baseline	0.00	0.35	[-0.16; 0.87]
Wang, TJ, et al 2023	70	105.40	17.80	72	103.50	20.00	followup	8.00	0.10	[-0.23; 0.43]
Xia, S, et al., 2023	80	67.70	16.20	80	65.80	14.40	followup	4.00	0.12	[-0.19; 0.43]
Wang, TJ, et al 2023	70	107.30	16.70	72	103.30	19.00	followup	16.00	0.22	[-0.11; 0.55]
Wang, TJ, et al 2023	70	106.90	16.60	72	102.70	20.00	followup	24.00	0.23	[-0.10; 0.56]
Xia, S, et al., 2023	80	72.70	15.80	80	68.00	13.50	followup	12.00	0.32	[0.01; 0.63]
Xia, S, et al., 2023	80	78.00	14.30	80	72.90	13.40	followup	24.00	0.37	[0.05; 0.68]
Zhang, J. et al., 2021	61	9.67	3.71	61	8.21	3.02	followup	12.00	0.43	[0.07; 0.79]
Gao Q, et al. 2020	40	-54.26	7.03	40	-60.17	7.26	followup	4.00	0.82	[0.36; 1.27]
Li, X, et al., 2017	102	76.58	9.06	108	64.28	12.49	followup	12.00	1.12	[0.83; 1.41]
Cheung YL, et al. 2002	29	7.48	1.12	30	5.56	0.50	followup	10.00	2.20	[1.54; 2.85]
Cheung YL, et al. 2002	29	7.51	0.98	30	4.96	0.66	followup	5.00	3.02	[2.26; 3.78]
Random effect	1032			1055					0.63	[-0.00; 1.25]
Prediction interval										[-0.69; 1.94]
gynaecological										
Sandsund, C, et al., 2017	72	64.20	21.80	70	66.70	19.70	baseline	0.00	-0.12	[-0.45; 0.21]
Chan, et al. 2005	80	58.29	50.71	75	60.44	40.96	baseline	0.00	-0.05	[-0.36; 0.27]
Powell, CB, et al., 2008	21	78.30	17.70	43	75.60	17.90	baseline	0.00	0.15	[-0.37; 0.67]
Chan, et al. 2005	80	60.31	41.32	75	66.46	47.98	followup	12.00	-0.14	[-0.45; 0.18]
Sandsund, C, et al., 2017	72	67.50	22.60	70	69.40	21.10	followup	24.00	-0.09	[-0.42; 0.24]
Chan, et al. 2005	80	71.69	74.33	75	77.23	53.91	followup	72.00	-0.08	[-0.39; 0.24]
Chan, et al. 2005	80	71.61	51.52	75	75.69	53.26	followup	60.00	-0.08	[-0.39; 0.24]
Sandsund, C, et al., 2017	72	66.20	19.10	70	67.30	22.00	followup	12.00	-0.05	[-0.38; 0.28]
Chan, et al. 2005	80	73.13	34.88	75	73.75	47.11	followup	36.00	-0.01	[-0.33; 0.30]
Chan, et al. 2005	80	67.36	46.78	75	66.55	51.44	followup	24.00	0.02	[-0.30; 0.33]
Chan, et al. 2005	80	77.86	49.64	75	76.04	45.64	followup	48.00	0.04	[-0.28; 0.35]
Powell, CB, et al., 2008	21	80.40	26.30	43	77.10	21.20	followup	12.00	0.14	[-0.38; 0.66]
Random effect	818			821					0.18	[-0.20; 0.56]
Prediction interval										[-1.71; 2.07]
prostate										
Penedo, FJ, et al., 2007	41	-82.35	14.02	30	-80.57	13.21	baseline	0.00	-0.13	[-0.60; 0.34]
Northouse, LL, et al., 2015	17	81.30	3.88	19	79.63	2.96	baseline	0.00	0.48	[-0.19; 1.14]
McCaughan, E, et al., 2018	13	85.63	14.10	4	76.88	22.04	baseline	0.00	0.53	[-0.61; 1.67]
Penedo, FJ, et al., 2007	41	-87.22	13.82	30	-79.63	14.56	followup	12.00	-0.53	[-1.01; -0.05]
Northhouse, LL, et al., 2007	107	87.00	10.80	121	86.90	10.60	followup	32.00	0.01	[-0.25; 0.27]
Northhouse, LL, et al., 2007	104	86.10	10.90	114	85.80	10.70	followup	48.00	0.03	[-0.24; 0.29]
Northhouse, LL, et al., 2007	112	87.20	10.60	123	85.50	10.30	followup	16.00	0.16	[-0.09; 0.42]
McCaughan, E, et al., 2018	13	85.76	17.07	4	80.22	22.75	followup	4.00	0.29	[-0.84; 1.41]
Northouse, LL, et al., 2015	17	88.51	3.03	19	76.94	3.44	followup	2.00	3.48	[2.41; 4.55]
Random effect	465			464					0.45	[-0.81; 1.71]
Prediction interval										[-1.50; 2.41]
**Random effect**	**7076**			**7618**					**0.45**	**[0.26; 0.64]**
**Prediction interval**										**[-0.47; 1.37]**

Figure S7.2. Subgroup analysis of the Global QoL. Forest plot represents the difference between the intervention vs. control group in the Global QoL domain with the cancer type subgroups as predicted at week 12 (post-intervention). SMD - Standardized mean difference, CI - confidence interval.

Figure S7.3.T24

Study	Experimental Patient N	Experimental Mean	Experimental SD	Control Patient N	Control Mean	Control SD	follow-up	Follow-up time	SMD of interested event	SMD	95%-CI
breast											
Garssen B, et al., 2012	34	74.40	4.20	36	76.50	4.30	baseline	0.00		-0.49	[-0.96; -0.01]
Seliniotaki, T, et al., 2021	27	62.00	33.80	26	72.20	16.20	baseline	0.00		-0.38	[-0.92; 0.17]
Elyasi, F, et al., 2021 I.	15	3.30	1.90	15	3.80	1.50	baseline	0.00		-0.28	[-1.00; 0.44]
Heiney, SP, et al., 2003	33	6.00	1.70	33	6.40	1.40	baseline	0.00		-0.25	[-0.74; 0.23]
Chu, X. et al, 2020	42	65.20	16.77	42	68.91	16.63	baseline	0.00		-0.22	[-0.65; 0.21]
Elyasi, F, et al., 2021 II.	20	3.50	1.80	20	3.80	1.50	baseline	0.00		-0.18	[-0.80; 0.44]
van den Berg, SW, et al. 2015 II.	70	66.79	16.57	80	69.79	17.91	baseline	0.00		-0.17	[-0.49; 0.15]
Savard, J, et al., 2006	21	42.51	3.19	16	43.11	3.91	baseline	0.00		-0.17	[-0.82; 0.48]
Nápoles AM, et al. 2015	76	66.46	16.92	75	68.83	15.33	baseline	0.00		-0.15	[-0.47; 0.17]
Dirksen, S. et al, 2007	34	83.30	11.90	38	84.80	9.20	baseline	0.00		-0.14	[-0.60; 0.32]
Peng, L, et al., 2022	28	69.05	16.49	29	71.26	17.05	baseline	0.00		-0.13	[-0.65; 0.39]
Qiu, H, et al., 2018 II.	98	76.78	19.91	196	79.49	21.60	baseline	0.00		-0.13	[-0.37; 0.11]
Von Ah, D, et al.,2012 I.	26	22.06	3.44	29	22.38	2.24	baseline	0.00		-0.11	[-0.64; 0.42]
Schellekens, MPJ, et al. 2016	69	95.43	21.60	70	97.09	23.54	baseline	0.00		-0.07	[-0.41; 0.26]
Reese, JB, et al., 2021	73	60.80	16.70	71	61.90	14.30	baseline	0.00		-0.07	[-0.40; 0.26]
Qiu, H, et al., 2018 I.	98	78.09	18.86	196	79.49	21.60	baseline	0.00		-0.07	[-0.31; 0.18]
Marchioro, G, et al. 1996	18	-58.44	22.39	18	-57.72	20.25	baseline	0.00		-0.03	[-0.69; 0.62]
Reich, RR, et al. 2016	167	62.44	27.52	155	62.74	24.68	baseline	0.00		-0.01	[-0.23; 0.21]
Hoffman, CJ, et al., 2012	101	96.57	17.22	106	96.68	21.05	baseline	0.00		-0.01	[-0.28; 0.27]
Walker, LG, et al.,1998	48	3.25	0.70	48	3.25	0.66	baseline	0.00		0.00	[-0.40; 0.40]
Rosen, KD, et al., 2018	57	93.61	22.68	55	92.96	25.12	baseline	0.00		0.03	[-0.34; 0.40]
Nikbakhsh, N, et al., 2018	20	44.09	8.48	20	43.64	13.73	baseline	0.00		0.04	[-0.58; 0.66]
Esplen MJ, et al., 2018	131	70.50	16.50	63	68.90	17.20	baseline	0.00		0.10	[-0.21; 0.40]
Klinkhammer-Schalke, M et al., 2012	100	54.35	26.09	100	51.45	23.19	baseline	0.00		0.12	[-0.16; 0.39]
Von Ah, D, et al.,2012 II.	27	22.98	3.08	29	22.38	2.24	baseline	0.00		0.22	[-0.30; 0.75]
Rahmani, S, et al. 2015	12	41.66	6.15	12	39.58	7.22	baseline	0.00		0.30	[-0.51; 1.10]
Yoo, HJ, et al. 2004	30	93.41	16.90	30	87.88	14.96	baseline	0.00		0.34	[-0.17; 0.85]
Cengiz, HO, et al., 2023	32	63.06	19.74	33	55.22	18.26	baseline	0.00		0.41	[-0.08; 0.90]
Gaston-Johansson, F, et al., 2011	38	22.60	4.30	35	20.60	4.00	baseline	0.00		0.48	[0.01; 0.94]
Jelvehzadeh, F, et al., 2022	24	4.71	1.07	24	3.94	1.06	baseline	0.00		0.71	[0.13; 1.30]
Heiney, SP, et al., 2003	33	6.00	1.50	33	6.80	1.50	followup	16.00		-0.53	[-1.02; -0.04]
Savard, J, et al., 2006	21	3.62	0.95	16	3.95	0.91	followup	8.00		-0.34	[-1.00; 0.31]
Von Ah, D, et al.,2012 I.	26	22.15	4.45	29	22.58	2.24	followup	8.00		-0.12	[-0.65; 0.41]
Peng, L, et al., 2022	28	66.67	24.00	29	69.25	22.17	followup	4.00		-0.11	[-0.63; 0.41]
Reich, RR, et al. 2016	167	68.43	27.76	155	70.36	22.70	followup	12.00		-0.08	[-0.29; 0.14]
Heiney, SP, et al., 2003	33	6.20	1.30	33	6.30	1.60	followup	6.00		-0.07	[-0.55; 0.41]
Klinkhammer-Schalke, M et al., 2012	100	53.62	22.46	100	55.07	20.29	followup	12.00		-0.07	[-0.34; 0.21]
Esplen MJ, et al., 2018	131	73.40	16.70	63	74.30	17.70	followup	48.00		-0.05	[-0.35; 0.25]
Reese, JB, et al., 2021	73	61.80	18.20	71	62.60	13.90	followup	8.00		-0.05	[-0.38; 0.28]
Reich, RR, et al. 2016	167	65.76	26.18	155	66.24	24.76	followup	6.00		-0.02	[-0.24; 0.20]
Rahmani, S, et al. 2015	12	12.00	61.11	12	12.00	58.33	followup	16.00		0.00	[-0.80; 0.80]
Seliniotaki, T, et al., 2021	27	65.00	28.00	26	64.00	20.50	followup	8.00		0.04	[-0.50; 0.58]
Peng, L, et al., 2022	28	72.32	22.11	29	71.26	15.26	followup	0.10		0.06	[-0.46; 0.57]
Von Ah, D, et al.,2012 II.	27	22.71	2.08	29	22.58	2.24	followup	8.00		0.06	[-0.47; 0.58]
Qiu, H, et al., 2018 II.	98	80.28	26.77	196	78.03	31.55	followup	4.00		0.07	[-0.17; 0.32]
Klinkhammer-Schalke, M et al., 2012	100	64.13	23.56	100	62.32	21.38	followup	48.00		0.08	[-0.20; 0.36]
Esplen MJ, et al., 2018	131	14.90	9.40	63	14.00	9.40	followup	8.00		0.10	[-0.21; 0.40]
Marchioro, G, et al. 1996	18	-54.17	16.90	18	-56.33	17.72	followup	4.00		0.12	[-0.53; 0.78]
Esplen MJ, et al., 2018	131	72.60	16.60	63	70.30	18.00	followup	24.00		0.13	[-0.17; 0.44]
Klinkhammer-Schalke, M et al., 2012	100	65.21	22.09	100	61.23	23.55	followup	36.00		0.17	[-0.10; 0.45]
Elyasi, F, et al., 2021 I.	15	4.30	1.00	15	4.10	1.10	followup	24.00		0.19	[-0.53; 0.90]
Nápoles AM, et al. 2015	76	77.24	15.13	75	74.39	15.34	followup	12.00		0.19	[-0.13; 0.51]
Elyasi, F, et al., 2021 II.	20	4.30	1.00	20	4.10	1.10	followup	24.00		0.19	[-0.43; 0.81]
Schellekens, MPJ, et al. 2016	69	107.36	18.80	70	103.34	22.35	followup	8.00		0.19	[-0.14; 0.53]
Qiu, H, et al., 2018 II.	98	89.52	33.46	196	83.21	31.55	followup	12.00		0.20	[-0.05; 0.44]
Qiu, H, et al., 2018 II.	98	94.70	35.69	196	87.72	34.70	followup	24.00		0.20	[-0.04; 0.44]
Nápoles AM, et al. 2015	76	80.64	13.64	75	77.02	15.62	followup	24.00		0.25	[-0.07; 0.57]
Dirksen, S. et al, 2007	34	91.60	15.00	38	87.70	14.70	followup	10.00		0.26	[-0.20; 0.72]
Hoffman, CJ, et al., 2012	101	103.56	17.91	106	96.84	21.14	followup	8.00		0.34	[0.07; 0.62]
Klinkhammer-Schalke, M et al., 2012	100	63.41	22.11	100	55.07	23.19	followup	24.00		0.37	[0.09; 0.65]
Hoffman, CJ, et al., 2012	101	103.78	17.85	106	96.22	19.43	followup	12.00		0.40	[0.13; 0.68]
Qiu, H, et al., 2018 I.	98	91.32	29.00	196	78.03	31.55	followup	4.00		0.43	[0.19; 0.68]
Walker, LG, et al.,1998	48	3.29	0.80	48	2.90	0.78	followup	15.00		0.49	[0.08; 0.90]
Yoo, HJ, et al. 2004	30	97.01	18.24	30	88.13	16.18	followup	12.00		0.51	[-0.01; 1.02]
Rosen, KD, et al., 2018	57	107.35	17.79	55	96.04	23.96	followup	5.00		0.53	[0.16; 0.91]
Gaston-Johansson, F, et al., 2011	38	24.70	4.30	35	21.90	5.00	followup	48.00		0.60	[0.13; 1.07]
Garssen B, et al., 2012	34	78.40	3.70	36	76.40	2.90	followup	5.00		0.60	[0.12; 1.08]
Garssen B, et al., 2012	34	80.40	3.30	36	78.20	3.20	followup	2.00		0.67	[0.19; 1.15]
Garssen B, et al., 2012	34	75.50	3.60	36	72.90	3.80	followup	13.00		0.69	[0.21; 1.18]
Qiu, H, et al., 2018 I.	98	113.18	35.69	196	87.72	34.70	followup	24.00		0.72	[0.48; 0.97]
Cengiz, HO, et al., 2023	32	66.98	17.72	33	53.03	18.65	followup	8.00		0.76	[0.25; 1.26]
Qiu, H, et al., 2018 I.	98	107.32	31.23	196	83.21	31.55	followup	12.00		0.76	[0.51; 1.02]
Chu, X. et al, 2020	42	85.41	16.08	42	71.90	18.50	followup	12.00		0.77	[0.33; 1.22]
Chu, X. et al, 2020	42	84.94	14.59	42	71.59	18.73	followup	8.00		0.79	[0.34; 1.23]
Yoo, HJ, et al. 2004	30	102.00	19.94	30	88.18	14.02	followup	24.00		0.79	[0.26; 1.32]
Garssen B, et al., 2012	34	81.00	3.40	36	78.20	3.20	followup	4.00		0.84	[0.35; 1.33]
Marchioro, G, et al. 1996	18	-48.72	12.22	18	-60.78	14.85	followup	12.00		0.87	[0.18; 1.55]
Rahmani, S, et al. 2015	12	66.66	9.40	12	54.16	10.95	followup	8.00		1.18	[0.30; 2.06]
Marchioro, G, et al. 1996	18	-44.72	9.18	18	-60.78	13.26	followup	24.00		1.38	[0.64; 2.11]
Jelvehzadeh, F, et al., 2022	24	6.05	1.00	24	4.65	0.80	followup	12.00		1.52	[0.87; 2.17]
Jelvehzadeh, F, et al., 2022	24	6.16	1.09	24	4.58	0.92	followup	8.00		1.54	[0.89; 2.19]
Marchioro, G, et al. 1996	18	-41.17	6.91	18	-60.28	13.33	followup	36.00		1.76	[0.98; 2.54]
van den Berg, SW, et al. 2015 II.	70	73.30	1.78	80	69.88	1.67	followup	4.00		1.97	[1.58; 2.36]
Nikbakhsh, N, et al., 2018	20	81.70	10.04	20	47.39	12.93	followup	12.00		2.91	[1.99; 3.82]
Random effect	4761			5278						0.37	[0.14; 0.61]
Prediction interval											[-0.59; 1.34]
gastroenterological											
Li, X, et al., 2017	102	56.32	7.80	108	62.38	11.46	baseline	0.00		-0.61	[-0.89; -0.34]
Wang, TJ, et al 2023	70	102.30	18.40	72	104.40	17.10	baseline	0.00		-0.12	[-0.45; 0.21]
Xia, S, et al., 2023	80	61.40	16.60	80	61.10	15.70	baseline	0.00		0.02	[-0.29; 0.33]
Gao Q, et al. 2020	40	-68.20	8.25	40	-68.44	8.82	baseline	0.00		0.03	[-0.41; 0.47]
Cheung YL, et al. 2002	29	4.96	1.74	30	4.36	1.62	baseline	0.00		0.35	[-0.16; 0.87]
Wang, TJ, et al 2023	70	105.40	17.80	72	103.50	20.00	followup	8.00		0.10	[-0.23; 0.43]
Xia, S, et al., 2023	80	67.70	16.20	80	65.80	14.40	followup	4.00		0.12	[-0.19; 0.43]
Wang, TJ, et al 2023	70	107.30	16.70	72	103.30	19.00	followup	16.00		0.22	[-0.11; 0.55]
Wang, TJ, et al 2023	70	106.90	16.60	72	102.70	20.00	followup	24.00		0.23	[-0.10; 0.56]
Xia, S, et al., 2023	80	72.70	15.80	80	68.00	13.50	followup	12.00		0.32	[0.01; 0.63]
Xia, S, et al., 2023	80	78.00	14.30	80	72.90	13.40	followup	24.00		0.37	[0.05; 0.68]
Zhang, J, et al., 2021	61	9.67	3.71	61	8.21	3.02	followup	12.00		0.43	[0.07; 0.79]
Gao Q, et al. 2020	40	-54.26	7.03	40	-60.17	7.28	followup	4.00		0.82	[0.36; 1.27]
Li, X, et al., 2017	102	76.58	9.06	108	64.28	12.49	followup	12.00		1.12	[0.83; 1.41]
Cheung YL, et al. 2002	29	7.48	1.12	30	5.56	0.50	followup	10.00		2.20	[1.54; 2.85]
Cheung YL, et al. 2002	29	7.51	0.98	30	4.96	0.66	followup	5.00		3.02	[2.26; 3.78]
Random effect	1032			1055						0.56	[-0.01; 1.13]
Prediction interval											[-0.67; 1.79]
gynaecological											
Sandsund, C, et al., 2017	72	64.20	21.80	70	66.70	19.70	baseline	0.00		-0.12	[-0.45; 0.21]
Chan, et al. 2005	80	58.29	50.71	75	60.44	40.96	baseline	0.00		-0.05	[-0.36; 0.27]
Powell, CB, et al., 2008	21	78.30	17.70	43	75.60	17.90	baseline	0.00		0.15	[-0.37; 0.67]
Chan, et al. 2005	80	60.31	41.32	75	66.46	47.98	followup	12.00		-0.14	[-0.45; 0.18]
Sandsund, C, et al., 2017	72	67.50	22.60	70	69.40	21.10	followup	24.00		-0.09	[-0.42; 0.24]
Chan, et al. 2005	80	71.69	74.33	75	77.23	63.91	followup	72.00		-0.08	[-0.39; 0.24]
Chan, et al. 2005	80	71.61	51.52	75	75.69	53.26	followup	60.00		-0.08	[-0.39; 0.24]
Sandsund, C, et al., 2017	72	66.20	19.10	70	67.30	22.00	followup	12.00		-0.05	[-0.38; 0.28]
Chan, et al. 2005	80	73.13	34.88	75	73.75	47.11	followup	36.00		-0.01	[-0.33; 0.30]
Chan, et al. 2005	80	67.38	46.78	75	66.55	51.44	followup	24.00		0.02	[-0.30; 0.33]
Chan, et al. 2005	80	77.86	49.64	75	76.04	45.64	followup	48.00		0.04	[-0.28; 0.35]
Powell, CB, et al., 2008	21	80.40	26.30	43	77.10	21.20	followup	12.00		0.14	[-0.38; 0.66]
Random effect	818			821						0.11	[-0.28; 0.50]
Prediction interval											[-1.76; 1.99]
prostate											
Penedo, FJ, et al., 2007	41	-82.35	14.02	30	-80.57	13.21	baseline	0.00		-0.13	[-0.60; 0.34]
Northouse, LL, et al., 2015	17	81.30	3.88	19	79.63	2.96	baseline	0.00		0.48	[-0.19; 1.14]
McCaughan, E, et al., 2018	13	85.83	14.10	4	76.88	22.04	baseline	0.00		0.53	[-0.61; 1.67]
Penedo, FJ, et al., 2007	41	-87.22	13.82	30	-79.63	14.56	followup	12.00		-0.53	[-1.01; -0.05]
Northhouse, LL, et al., 2007	107	87.00	10.80	121	86.90	10.60	followup	32.00		0.01	[-0.25; 0.27]
Northhouse, LL, et al., 2007	104	86.10	10.90	114	85.80	10.70	followup	48.00		0.03	[-0.24; 0.29]
Northhouse, LL, et al., 2007	112	87.20	10.60	123	85.50	10.30	followup	16.00		0.16	[-0.09; 0.42]
McCaughan, E, et al., 2018	13	85.76	17.07	4	80.22	22.75	followup	4.00		0.29	[-0.84; 1.41]
Northouse, LL, et al., 2015	17	88.51	3.03	19	76.94	3.44	followup	2.00		3.48	[2.41; 4.55]
Random effect	465			464						0.38	[-0.78; 1.55]
Prediction interval											[-1.49; 2.26]
**Random effect**	**7076**			**7618**						**0.37**	**[0.17; 0.58]**
**Prediction interval**											**[-0.58; 1.33]**

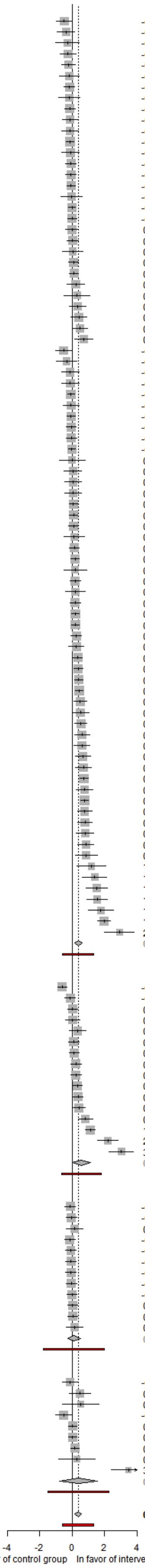

Figure S7.3. Subgroup analysis of the Global QoL. Forest plot represents the difference between the intervention vs. control group in the Global QoL domain with the cancer type subgroups as predicted at week 24 (post-intervention). SMD - Standardized mean difference, CI - confidence interval.

Figure S7.4.T48

Study	Experimental Patient N	Experimental Mean	Experimental SD	Control Patient N	Control Mean	Control SD	follow-up	Follow-up time	SMD	95%-CI
**breast**										
Garssen B, et al., 2012	34	74.40	4.20	36	76.50	4.30	baseline	0.00	-0.49	[-0.96; -0.01]
Seliniotaki, T, et al., 2021	27	62.00	33.80	26	72.20	16.20	baseline	0.00	-0.38	[-0.92; 0.17]
Elyasi, F, et al., 2021 I.	15	3.30	1.90	15	3.80	1.50	baseline	0.00	-0.28	[-1.00; 0.44]
Heiney, SP, et al., 2003	33	6.00	1.70	33	6.40	1.40	baseline	0.00	-0.25	[-0.74; 0.23]
Chu, X. et al, 2020	42	65.20	16.77	42	68.91	16.63	baseline	0.00	-0.22	[-0.65; 0.21]
Elyasi, F, et al., 2021 II.	20	3.50	1.80	20	3.80	1.50	baseline	0.00	-0.18	[-0.80; 0.44]
van den Berg, SW, et al. 2015 II.	70	66.79	16.57	80	69.79	17.91	baseline	0.00	-0.17	[-0.49; 0.15]
Savard, J, et al., 2006	21	42.51	3.19	16	43.11	3.91	baseline	0.00	-0.17	[-0.82; 0.48]
Nápoles AM, et al. 2015	76	66.46	16.92	75	68.83	15.33	baseline	0.00	-0.15	[-0.47; 0.17]
Dirksen, S. et al, 2007	34	83.30	11.90	38	84.80	9.20	baseline	0.00	-0.14	[-0.60; 0.32]
Peng, L, et al., 2022	28	69.05	16.49	29	71.26	17.05	baseline	0.00	-0.13	[-0.65; 0.39]
Qiu, H, et al., 2018 II.	98	76.78	19.91	196	79.49	21.60	baseline	0.00	-0.13	[-0.37; 0.11]
Von Ah, D, et al.,2012 I.	26	22.06	3.44	29	22.38	2.24	baseline	0.00	-0.11	[-0.64; 0.42]
Schellekens, MPJ, et al. 2016	69	95.43	21.60	70	97.09	23.54	baseline	0.00	-0.07	[-0.41; 0.26]
Reese, JB, et al., 2021	73	60.80	16.70	71	61.90	14.30	baseline	0.00	-0.07	[-0.40; 0.26]
Qiu, H, et al., 2018 I.	98	78.09	18.86	196	79.49	21.60	baseline	0.00	-0.07	[-0.31; 0.18]
Marchioro, G, et al. 1996	18	-58.44	22.39	18	-57.72	20.25	baseline	0.00	-0.03	[-0.69; 0.62]
Reich, RR, et al. 2016	167	62.44	27.52	155	62.74	24.68	baseline	0.00	-0.01	[-0.23; 0.21]
Hoffman, CJ, et al., 2012	101	96.57	17.22	106	96.68	21.05	baseline	0.00	-0.01	[-0.28; 0.27]
Walker, LG, et al.,1998	48	3.25	0.70	48	3.25	0.66	baseline	0.00	0.00	[-0.40; 0.40]
Rosen, KD, et al., 2018	57	93.61	22.68	55	92.96	25.12	baseline	0.00	0.03	[-0.34; 0.40]
Nikbakhsh, N, et al., 2018	20	44.09	8.48	20	43.64	13.73	baseline	0.00	0.04	[-0.58; 0.66]
Esplen MJ, et al., 2018	131	70.50	16.50	63	68.90	17.20	baseline	0.00	0.10	[-0.21; 0.40]
Klinkhammer-Schalke, M et al., 2012	100	54.35	26.09	100	51.45	23.19	baseline	0.00	0.12	[-0.16; 0.39]
Von Ah, D, et al.,2012 II.	27	22.98	3.08	29	22.38	2.24	baseline	0.00	0.22	[-0.30; 0.75]
Rahmani, S, et al. 2015	12	41.66	6.15	12	39.58	7.22	baseline	0.00	0.30	[-0.51; 1.10]
Yoo, HJ, et al. 2004	30	93.41	16.90	30	87.88	14.96	baseline	0.00	0.34	[-0.17; 0.85]
Cengiz, HO, et al., 2023	32	63.06	19.74	33	55.22	18.26	baseline	0.00	0.41	[-0.08; 0.90]
Gaston-Johansson, F, et al., 2011	38	22.60	4.30	35	20.60	4.00	baseline	0.00	0.48	[0.01; 0.94]
Jelvehzadeh, F, et al., 2022	24	4.71	1.07	24	3.94	1.06	baseline	0.00	0.71	[0.13; 1.30]
Heiney, SP, et al., 2003	33	6.00	1.50	33	6.80	1.50	followup	16.00	-0.53	[-1.02; -0.04]
Savard, J, et al., 2006	21	3.62	0.95	16	3.95	0.91	followup	8.00	-0.34	[-1.00; 0.31]
Von Ah, D, et al.,2012 I.	26	22.15	4.45	29	22.58	2.24	followup	8.00	-0.12	[-0.65; 0.41]
Peng, L, et al., 2022	28	66.67	24.00	29	69.25	22.17	followup	4.00	-0.11	[-0.63; 0.41]
Reich, RR, et al. 2016	167	68.43	27.76	155	70.36	22.70	followup	12.00	-0.08	[-0.29; 0.14]
Heiney, SP, et al., 2003	33	6.20	1.30	33	6.30	1.60	followup	6.00	-0.07	[-0.55; 0.41]
Klinkhammer-Schalke, M et al., 2012	100	53.62	22.46	100	55.07	20.29	followup	12.00	-0.07	[-0.34; 0.21]
Esplen MJ, et al., 2018	131	73.40	16.70	63	74.30	17.70	followup	48.00	-0.05	[-0.35; 0.25]
Reese, JB, et al., 2021	73	61.80	18.20	71	62.60	13.90	followup	8.00	-0.05	[-0.38; 0.28]
Reich, RR, et al. 2016	167	65.76	26.18	155	66.24	24.76	followup	6.00	-0.02	[-0.24; 0.20]
Rahmani, S, et al. 2015	12	12.00	61.11	12	12.00	58.33	followup	16.00	0.00	[-0.80; 0.80]
Seliniotaki, T, et al., 2021	27	65.00	28.00	26	64.00	20.50	followup	8.00	0.04	[-0.50; 0.58]
Peng, L, et al., 2022	28	72.32	22.11	29	71.26	15.26	followup	0.10	0.06	[-0.46; 0.57]
Von Ah, D, et al.,2012 II.	27	22.71	2.08	29	22.58	2.24	followup	8.00	0.06	[-0.47; 0.58]
Qiu, H, et al., 2018 II.	98	80.28	26.77	196	78.03	31.55	followup	4.00	0.07	[-0.17; 0.32]
Klinkhammer-Schalke, M et al., 2012	100	64.13	23.56	100	62.32	21.38	followup	48.00	0.08	[-0.20; 0.36]
Esplen MJ, et al., 2018	131	14.90	9.40	63	14.00	9.40	followup	8.00	0.10	[-0.21; 0.40]
Marchioro, G, et al. 1996	18	-54.17	16.90	18	-56.33	17.72	followup	4.00	0.12	[-0.53; 0.78]
Esplen MJ, et al., 2018	131	72.60	16.60	63	70.30	18.00	followup	24.00	0.13	[-0.17; 0.44]
Klinkhammer-Schalke, M et al., 2012	100	65.21	22.09	100	61.23	23.55	followup	36.00	0.17	[-0.10; 0.45]
Elyasi, F, et al., 2021 I.	15	4.30	1.00	15	4.10	1.10	followup	24.00	0.19	[-0.53; 0.90]
Nápoles AM, et al. 2015	76	77.24	15.13	75	74.39	15.34	followup	12.00	0.19	[-0.13; 0.51]
Elyasi, F, et al., 2021 II.	20	4.30	1.00	20	4.10	1.10	followup	24.00	0.19	[-0.43; 0.81]
Schellekens, MPJ, et al. 2016	69	107.36	18.80	70	103.34	22.35	followup	8.00	0.19	[-0.14; 0.53]
Qiu, H, et al., 2018 II.	98	89.52	33.46	196	83.21	31.55	followup	12.00	0.20	[-0.05; 0.44]
Qiu, H, et al., 2018 II.	98	94.70	35.69	196	87.72	34.70	followup	24.00	0.20	[-0.04; 0.44]
Nápoles AM, et al. 2015	76	80.64	13.64	75	77.02	15.62	followup	24.00	0.25	[-0.07; 0.57]
Dirksen, S. et al, 2007	34	91.60	15.00	38	87.70	14.70	followup	10.00	0.26	[-0.20; 0.72]
Hoffman, CJ, et al., 2012	101	103.56	17.91	106	96.84	21.14	followup	8.00	0.34	[0.07; 0.62]
Klinkhammer-Schalke, M et al., 2012	100	63.41	22.11	100	55.07	23.19	followup	24.00	0.37	[0.09; 0.65]
Hoffman, CJ, et al., 2012	101	103.78	17.85	106	96.22	19.43	followup	12.00	0.40	[0.13; 0.68]
Qiu, H, et al., 2018 I.	98	91.32	29.00	196	78.03	31.55	followup	4.00	0.43	[0.19; 0.68]
Walker, LG, et al.,1998	48	3.29	0.80	48	2.90	0.78	followup	15.00	0.49	[0.08; 0.90]
Yoo, HJ, et al. 2004	30	97.01	18.24	30	88.13	16.18	followup	12.00	0.51	[-0.01; 1.02]
Rosen, KD, et al., 2018	57	107.35	17.79	55	96.04	23.96	followup	5.00	0.53	[0.16; 0.91]
Gaston-Johansson, F, et al., 2011	38	24.70	4.30	35	21.90	5.00	followup	48.00	0.60	[0.13; 1.07]
Garssen B, et al., 2012	34	78.40	3.70	36	76.40	2.90	followup	5.00	0.60	[0.12; 1.08]
Garssen B, et al., 2012	34	80.40	3.30	36	78.20	3.20	followup	2.00	0.67	[0.19; 1.15]
Garssen B, et al., 2012	34	75.50	3.60	36	72.90	3.80	followup	13.00	0.69	[0.21; 1.18]
Qiu, H, et al., 2018 I.	98	113.18	35.69	196	87.72	34.70	followup	24.00	0.72	[0.48; 0.97]
Cengiz, HO, et al., 2023	32	66.98	17.72	33	53.03	18.65	followup	8.00	0.76	[0.25; 1.26]
Qiu, H, et al., 2018 I.	98	107.32	31.23	196	83.21	31.55	followup	12.00	0.76	[0.51; 1.02]
Chu, X. et al, 2020	42	85.41	16.08	42	71.90	18.50	followup	12.00	0.77	[0.33; 1.22]
Chu, X. et al, 2020	42	84.94	14.59	42	71.59	18.73	followup	8.00	0.79	[0.34; 1.23]
Yoo, HJ, et al. 2004	30	102.00	19.94	30	88.18	14.02	followup	24.00	0.79	[0.26; 1.32]
Garssen B, et al., 2012	34	81.00	3.40	36	78.20	3.20	followup	4.00	0.84	[0.35; 1.33]
Marchioro, G, et al. 1996	18	-48.72	12.22	18	-60.78	14.85	followup	12.00	0.87	[0.18; 1.55]
Rahmani, S, et al. 2015	12	66.66	9.40	12	54.16	10.95	followup	8.00	1.18	[0.30; 2.06]
Marchioro, G, et al. 1996	18	-44.72	9.18	18	-60.78	13.26	followup	24.00	1.38	[0.64; 2.11]
Jelvehzadeh, F, et al., 2022	24	6.05	1.00	24	4.65	0.80	followup	12.00	1.52	[0.87; 2.17]
Jelvehzadeh, F, et al., 2022	24	6.16	1.09	24	4.58	0.92	followup	8.00	1.54	[0.89; 2.19]
Marchioro, G, et al. 1996	18	-41.17	6.91	18	-60.28	13.33	followup	36.00	1.76	[0.98; 2.54]
van den Berg, SW, et al. 2015 II.	70	73.30	1.78	80	69.88	1.67	followup	4.00	1.97	[1.58; 2.36]
Nikbakhsh, N, et al., 2018	20	81.70	10.04	20	47.39	12.93	followup	12.00	2.91	[1.99; 3.82]
Random effect	4761			5278					0.28	[-0.03; 0.59]
Prediction interval										[-0.81; 1.37]
**gastroenterological**										
Li, X, et al., 2017	102	56.32	7.80	108	62.38	11.46	baseline	0.00	-0.61	[-0.89; -0.34]
Wang, TJ, et al 2023	70	102.30	18.40	72	104.40	17.10	baseline	0.00	-0.12	[-0.45; 0.21]
Xia, S, et al., 2023	80	61.40	16.60	80	61.10	15.70	baseline	0.00	0.02	[-0.29; 0.33]
Gao Q, et al. 2020	40	-68.20	8.25	40	-68.44	8.82	baseline	0.00	0.03	[-0.41; 0.47]
Cheung YL, et al. 2002	29	4.96	1.74	30	4.36	1.62	baseline	0.00	0.35	[-0.16; 0.87]
Wang, TJ, et al 2023	70	105.40	17.80	72	103.50	20.00	followup	8.00	0.10	[-0.23; 0.43]
Xia, S, et al., 2023	80	67.70	16.20	80	65.80	14.40	followup	4.00	0.12	[-0.19; 0.43]
Wang, TJ, et al 2023	70	107.30	16.70	72	103.30	19.00	followup	16.00	0.22	[-0.11; 0.55]
Wang, TJ, et al 2023	70	106.90	16.60	72	102.70	20.00	followup	24.00	0.23	[-0.10; 0.56]
Xia, S, et al., 2023	80	72.70	15.80	80	68.00	13.50	followup	12.00	0.32	[0.01; 0.63]
Xia, S, et al., 2023	80	78.00	14.30	80	72.90	13.40	followup	24.00	0.37	[0.05; 0.68]
Zhang, J, et al., 2021	61	9.67	3.71	61	8.21	3.02	followup	12.00	0.43	[0.07; 0.79]
Gao Q, et al. 2020	40	-54.26	7.03	40	-60.17	7.28	followup	4.00	0.82	[0.36; 1.27]
Li, X, et al., 2017	102	76.58	9.06	108	64.28	12.49	followup	12.00	1.12	[0.83; 1.41]
Cheung YL, et al. 2002	29	7.48	1.12	30	5.56	0.50	followup	10.00	2.20	[1.54; 2.85]
Cheung YL, et al. 2002	29	7.51	0.98	30	4.96	0.66	followup	5.00	3.02	[2.26; 3.78]
Random effect	1032			1055					0.46	[-0.08; 1.01]
Prediction interval										[-0.70; 1.62]
**gynaecological**										
Sandsund, C, et al., 2017	72	64.20	21.80	70	66.70	19.70	baseline	0.00	-0.12	[-0.45; 0.21]
Chan, et al. 2005	80	58.29	50.71	75	60.44	40.96	baseline	0.00	-0.05	[-0.36; 0.27]
Powell, CB, et al., 2008	21	78.30	17.70	43	75.60	17.90	baseline	0.00	0.15	[-0.37; 0.67]
Chan, et al. 2005	80	60.31	41.32	75	66.46	47.98	followup	12.00	-0.14	[-0.45; 0.18]
Sandsund, C, et al., 2017	72	67.50	22.60	70	69.40	21.10	followup	24.00	-0.09	[-0.42; 0.24]
Chan, et al. 2005	80	71.69	74.33	75	77.23	63.91	followup	72.00	-0.08	[-0.39; 0.24]
Chan, et al. 2005	80	71.61	51.52	75	75.69	53.26	followup	60.00	-0.08	[-0.39; 0.24]
Sandsund, C, et al., 2017	72	66.20	19.10	70	67.30	22.00	followup	12.00	-0.05	[-0.38; 0.28]
Chan, et al. 2005	80	73.13	34.88	75	73.75	47.11	followup	36.00	-0.01	[-0.33; 0.30]
Chan, et al. 2005	80	67.38	46.78	75	66.55	51.44	followup	24.00	0.02	[-0.30; 0.33]
Chan, et al. 2005	80	77.86	49.64	75	76.04	45.64	followup	48.00	0.04	[-0.28; 0.35]
Powell, CB, et al., 2008	21	80.40	26.30	43	77.10	21.20	followup	12.00	0.14	[-0.38; 0.66]
Random effect	818			821					0.02	[-0.41; 0.44]
Prediction interval										[-1.76; 1.80]
**prostate**										
Penedo, FJ, et al., 2007	41	-82.35	14.02	30	-80.57	13.21	baseline	0.00	-0.13	[-0.60; 0.34]
Northouse, LL, et al., 2015	17	81.30	3.88	19	79.63	2.96	baseline	0.00	0.48	[-0.19; 1.14]
McCaughan, E, et al., 2018	13	85.83	14.10	4	76.88	22.04	baseline	0.00	0.53	[-0.61; 1.67]
Penedo, FJ, et al., 2007	41	-87.22	13.82	30	-79.63	14.56	followup	12.00	-0.53	[-1.01; -0.05]
Northhouse, LL, et al., 2007	107	87.00	10.80	121	86.90	10.60	followup	32.00	0.01	[-0.25; 0.27]
Northhouse, LL, et al., 2007	104	86.10	10.90	114	85.80	10.70	followup	48.00	0.03	[-0.24; 0.29]
Northhouse, LL, et al., 2007	112	87.20	10.60	123	85.50	10.30	followup	16.00	0.16	[-0.09; 0.42]
McCaughan, E, et al., 2018	13	85.76	17.07	4	80.22	22.75	followup	4.00	0.29	[-0.84; 1.41]
Northouse, LL, et al., 2015	17	88.51	3.03	19	76.94	3.44	followup	2.00	3.48	[2.41; 4.55]
Random effect	465			464					0.29	[-0.75; 1.33]
Prediction interval										[-1.41; 1.99]
**Random effect**	**7076**			**7618**					**0.26**	**[-0.02; 0.54]**
**Prediction interval**										**[-0.89; 1.40]**

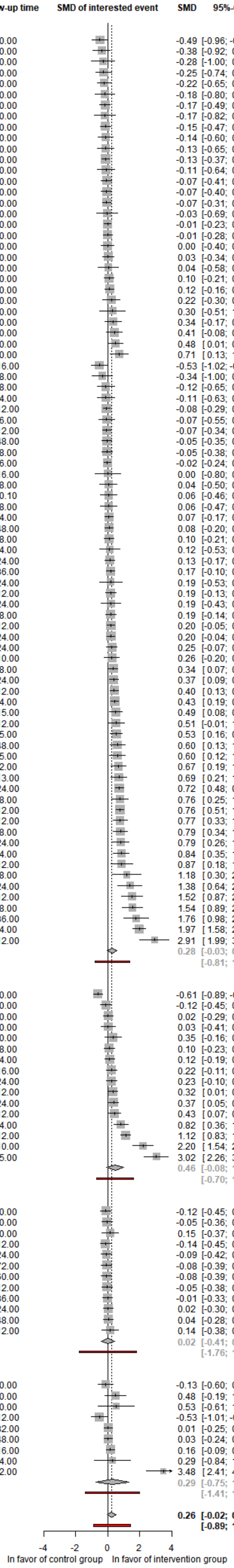

Figure S7.4. Subgroup analysis of the Global QoL. Forest plot represents the difference between the intervention vs. control group in the Global QoL domain with the cancer type subgroups as predicted at week 48 (post-intervention). SMD - Standardized mean difference, CI - confidence interval.

S8.Subgroup analysis of Emotional QoL: Provider

Figure S8.1.T0

Study	Experimental Patient N	Experimental Mean	Experimental SD	Control Patient N	Control Mean	Control SD	follow-up	Follow-up time	SMD	95%-CI
**psychologist**										
Seliniotaki, T, et al., 2021	27	56.10	29.10	26	74.20	20.90	baseline	0.00	-0.70	[-1.26; -0.15]
Reich, RR, et al. 2016	167	63.83	17.42	155	68.57	18.32	baseline	0.00	-0.26	[-0.48; -0.05]
Serfaty M, et al., 2018	20	12.30	5.20	22	13.60	4.90	baseline	0.00	-0.25	[-0.86; 0.36]
Peng, L, et al., 2022	28	69.94	16.25	29	72.41	16.08	baseline	0.00	-0.15	[-0.67; 0.37]
Rahmani, S, et al. 2015	12	11.11	8.94	12	12.50	9.05	baseline	0.00	-0.15	[-0.95; 0.65]
Qiu, H, et al., 2018 II.	98	11.50	6.30	196	12.22	6.56	baseline	0.00	-0.11	[-0.35; 0.13]
Qiu, H, et al., 2018 I.	98	11.72	6.65	196	12.22	6.56	baseline	0.00	-0.08	[-0.32; 0.17]
Hernandez, EG, et al. 2018	28	13.07	5.26	28	13.39	4.94	baseline	0.00	-0.06	[-0.59; 0.46]
Johansson, B, et al., 2008	128	74.00	23.00	116	75.00	23.00	baseline	0.00	-0.04	[-0.29; 0.21]
Powell, CB, et al., 2008	21	16.10	4.20	43	16.20	5.00	baseline	0.00	-0.02	[-0.54; 0.50]
Chan, et al. 2005	80	58.10	45.50	75	54.60	48.48	baseline	0.00	0.07	[-0.24; 0.39]
Liu, T, et al., 2019	49	71.18	18.38	53	69.23	22.04	baseline	0.00	0.10	[-0.29; 0.48]
Penedo, FJ, et al., 2007	41	-19.24	4.04	30	-19.63	3.43	baseline	0.00	0.10	[-0.37; 0.57]
Penedo, FJ, et al., 2020	95	-20.03	3.80	97	-20.46	3.74	baseline	0.00	0.11	[-0.17; 0.40]
Klinkhammer-Schalke, M et al., 2012	100	51.86	29.03	100	48.14	28.29	baseline	0.00	0.13	[-0.15; 0.41]
Arving, C, et al., 2007	47	69.00	25.00	38	63.00	18.00	baseline	0.00	0.27	[-0.16; 0.70]
Rodrigez, B, et al., 2014	8	88.54	22.24	7	79.76	36.28	baseline	0.00	0.28	[-0.74; 1.30]
Lu, Z, et al., 201	203	79.70	3.60	103	71.30	4.90	baseline	0.00	2.05	[1.76; 2.34]
Penedo, FJ, et al., 2007	41	-20.73	3.31	30	-19.20	3.62	followup	12.00	-0.44	[-0.92; 0.04]
Serfaty M, et al., 2018	20	15.40	4.50	22	16.60	4.90	followup	12.00	-0.25	[-0.86; 0.36]
Serfaty M, et al., 2018	20	15.60	4.20	22	16.50	3.70	followup	24.00	-0.22	[-0.83; 0.38]
Chan, et al. 2005	80	65.70	47.01	75	73.94	39.30	followup	12.00	-0.19	[-0.50; 0.13]
Klinkhammer-Schalke, M et al., 2012	100	55.34	28.29	100	58.56	28.29	followup	12.00	-0.11	[-0.39; 0.16]
Johansson, B, et al., 2008	128	83.00	18.00	116	85.00	19.00	followup	24.00	-0.11	[-0.36; 0.14]
Serfaty M, et al., 2018	20	16.60	5.50	22	17.10	4.40	followup	18.00	-0.10	[-0.70; 0.51]
Reich, RR, et al. 2016	167	71.33	19.41	155	72.71	19.13	followup	12.00	-0.07	[-0.29; 0.15]
Chan, et al. 2005	80	75.77	73.38	75	79.44	64.08	followup	72.00	-0.05	[-0.37; 0.26]
Chan, et al. 2005	80	76.73	57.02	75	78.59	56.21	followup	48.00	-0.03	[-0.35; 0.28]
Chan, et al. 2005	80	73.39	67.76	75	75.30	55.80	followup	24.00	-0.03	[-0.35; 0.28]
Chan, et al. 2005	80	68.59	48.12	75	69.81	48.88	followup	60.00	-0.03	[-0.34; 0.29]
Johansson, B, et al., 2008	128	85.00	17.00	116	85.00	17.00	followup	48.00	0.00	[-0.25; 0.25]
Rahmani, S, et al. 2015	12	12.00	48.61	12	12.00	23.61	followup	16.00	0.00	[-0.80; 0.80]
Qiu, H, et al., 2018 II.	98	12.32	9.06	196	11.90	7.89	followup	4.00	0.05	[-0.19; 0.29]
Johansson, B, et al., 2008	128	87.00	17.00	116	86.00	20.00	followup	96.00	0.05	[-0.20; 0.31]
Chan, et al. 2005	80	77.37	58.41	75	73.44	52.28	followup	36.00	0.07	[-0.24; 0.39]
Klinkhammer-Schalke, M et al., 2012	100	63.52	27.07	100	61.29	27.29	followup	48.00	0.08	[-0.20; 0.36]
Reich, RR, et al. 2016	167	70.87	18.02	155	69.36	18.78	followup	6.00	0.08	[-0.14; 0.30]
Powell, CB, et al., 2008	21	18.50	5.40	43	18.00	5.90	followup	12.00	0.09	[-0.44; 0.61]
Arving, C, et al., 2007	47	76.00	24.00	38	74.00	20.00	followup	12.00	0.09	[-0.34; 0.52]
Johansson, B, et al., 2008	128	83.00	18.00	116	81.00	19.00	followup	12.00	0.11	[-0.14; 0.36]
Penedo, FJ, et al., 2020	95	-20.18	3.80	97	-20.62	3.84	followup	24.00	0.11	[-0.17; 0.40]
Qiu, H, et al., 2018 II.	98	14.58	9.06	196	13.52	7.89	followup	24.00	0.13	[-0.12; 0.37]
Qiu, H, et al., 2018 II.	98	14.23	7.67	196	13.10	8.87	followup	12.00	0.13	[-0.11; 0.38]
Hernandez, EG, et al. 2018	28	14.00	4.69	28	13.32	5.32	followup	24.00	0.13	[-0.39; 0.66]
Klinkhammer-Schalke, M et al., 2012	100	66.50	26.80	100	59.06	29.53	followup	36.00	0.26	[-0.02; 0.54]
Rodrigez, B, et al., 2014	8	88.54	22.24	7	79.76	36.28	followup	0.10	0.28	[-0.74; 1.30]
Hernandez, EG, et al. 2018	28	16.12	4.09	28	14.72	4.63	followup	8.00	0.32	[-0.21; 0.84]
Penedo, FJ, et al., 2020	95	-19.20	3.90	97	-20.43	3.84	followup	48.00	0.32	[0.03; 0.60]
Arving, C, et al., 2007	47	82.00	20.00	38	75.00	24.00	followup	24.00	0.32	[-0.11; 0.75]
Klinkhammer-Schalke, M et al., 2012	100	66.25	25.31	100	56.58	28.54	followup	24.00	0.36	[0.08; 0.64]
Seliniotaki, T, et al., 2021	27	74.50	24.50	26	61.70	36.70	followup	8.00	0.41	[-0.14; 0.95]
Qiu, H, et al., 2018 I.	98	15.42	9.06	196	11.90	7.89	followup	4.00	0.42	[0.18; 0.67]
Arving, C, et al., 2007	47	80.00	21.00	38	69.00	23.00	followup	4.00	0.50	[0.06; 0.93]
Rodrigez, B, et al., 2014	8	93.33	10.87	7	80.56	26.79	followup	2.00	0.60	[-0.44; 1.65]
Qiu, H, et al., 2018 I.	98	19.23	9.76	196	13.10	8.87	followup	12.00	0.67	[0.42; 0.91]
Qiu, H, et al., 2018 I.	98	20.00	8.37	196	13.52	7.89	followup	24.00	0.80	[0.55; 1.05]
Liu, T, et al., 2019	49	63.49	13.78	53	51.93	14.62	followup	9.00	0.81	[0.40; 1.21]
Peng, L, et al., 2022	28	80.95	13.20	29	70.12	9.05	followup	4.00	0.95	[0.40; 1.50]
Peng, L, et al., 2022	28	83.33	14.87	29	69.25	8.94	followup	0.10	1.14	[0.57; 1.70]
Liu, T, et al., 2019	49	74.12	9.50	53	56.57	16.10	followup	12.00	1.31	[0.88; 1.73]
Lu, Z, et al., 201	203	85.40	3.50	103	77.60	5.60	followup	9.00	1.80	[1.52; 2.08]
Rahmani, S, et al. 2015	12	56.94	11.14	12	22.91	10.73	followup	8.00	3.00	[1.78; 4.23]
Random effect	4497			4960					0.61	[0.25; 0.97]
Prediction interval										[-0.66; 1.87]
**healthcare professional**										
Zaman, ACGNM, et al., 2021	42	44.00	9.90	46	45.30	9.80	baseline	0.00	-0.13	[-0.55; 0.29]
Berglund, G. et al. 2007	39	81.20	18.90	150	83.60	19.70	baseline	0.00	-0.12	[-0.47; 0.23]
Sandsund, C, et al., 2017	72	67.10	25.30	70	67.90	23.40	baseline	0.00	-0.03	[-0.36; 0.30]
Girgis, A, et al., 2009 II.	120	79.90	20.30	117	79.70	22.70	baseline	0.00	0.01	[-0.25; 0.26]
Girgis, A, et al., 2009 I.	110	80.70	21.60	117	79.70	22.70	baseline	0.00	0.04	[-0.22; 0.31]
Braeken APB, et al. 2013	136	78.40	20.77	144	76.12	24.16	baseline	0.00	0.10	[-0.13; 0.34]
Guo, Z, et al., 2013	89	72.57	12.51	89	70.77	14.71	baseline	0.00	0.13	[-0.16; 0.43]
Schofield, P, et al., 2013	55	77.12	22.92	53	72.93	23.08	baseline	0.00	0.18	[-0.20; 0.56]
Yun et al., 2017	134	76.80	19.40	72	73.00	23.00	baseline	0.00	0.18	[-0.10; 0.47]
Hoffman, CJ, et al., 2012	102	16.91	3.84	109	15.97	4.58	baseline	0.00	0.22	[-0.05; 0.49]
Elyasi, F, et al., 2021 II.	20	10.60	3.10	20	9.60	2.60	baseline	0.00	0.34	[-0.28; 0.97]
Elyasi, F, et al., 2021 I.	15	11.50	3.80	15	9.60	2.60	baseline	0.00	0.57	[-0.16; 1.30]
Cengiz, HO, et al., 2023	32	12.90	5.40	33	9.75	5.22	baseline	0.00	0.59	[0.09; 1.08]
Zaman, ACGNM, et al., 2021	42	47.70	9.70	46	50.80	7.50	followup	48.00	-0.36	[-0.78; 0.07]
Berglund, G. et al. 2007	39	81.60	20.10	150	87.80	18.00	followup	48.00	-0.33	[-0.69; 0.02]
Zaman, ACGNM, et al., 2021	42	46.20	10.70	46	49.40	8.30	followup	24.00	-0.33	[-0.75; 0.09]
Braeken APB, et al. 2013	136	78.38	22.75	144	79.46	20.68	followup	12.00	-0.05	[-0.28; 0.18]
Elyasi, F, et al., 2021 II.	20	9.40	2.10	20	9.50	2.60	followup	24.00	-0.04	[-0.66; 0.58]
Beatty, L. et al. 2015	30	69.03	4.81	30	69.16	4.79	followup	6.00	-0.03	[-0.53; 0.48]
Schofield, P, et al., 2013	55	75.31	22.19	53	75.51	22.22	followup	12.00	-0.01	[-0.39; 0.37]
Girgis, A, et al., 2009 II.	120	85.70	16.40	117	84.90	18.50	followup	12.00	0.05	[-0.21; 0.30]
Girgis, A, et al., 2009 I.	110	86.40	20.20	117	84.90	18.50	followup	12.00	0.08	[-0.18; 0.34]
Zaman, ACGNM, et al., 2021	42	47.60	8.80	46	46.80	10.40	followup	36.00	0.08	[-0.34; 0.50]
Yun et al., 2017	134	78.00	19.90	72	75.90	18.30	followup	48.00	0.11	[-0.18; 0.39]
Braeken APB, et al. 2013	136	83.66	20.80	144	81.23	20.60	followup	48.00	0.12	[-0.12; 0.35]
Girgis, A, et al., 2009 II.	120	86.70	18.70	117	84.40	18.90	followup	24.00	0.12	[-0.13; 0.38]
Sandsund, C, et al., 2017	72	70.00	23.90	70	66.40	26.20	followup	12.00	0.14	[-0.19; 0.47]
Zaman, ACGNM, et al., 2021	42	46.80	9.80	46	45.30	9.20	followup	12.00	0.16	[-0.26; 0.58]
Girgis, A, et al., 2009 I.	110	88.70	17.30	117	84.40	18.90	followup	24.00	0.24	[-0.02; 0.50]
Sandsund, C, et al., 2017	72	75.50	20.20	70	66.70	28.60	followup	24.00	0.35	[0.02; 0.69]
Hoffman, CJ, et al., 2012	102	18.14	3.82	109	16.59	4.40	followup	8.00	0.37	[0.10; 0.65]
Schofield, P, et al., 2013	55	81.43	20.19	53	73.23	20.40	followup	8.00	0.40	[0.02; 0.78]
Elyasi, F, et al., 2021 I.	15	11.00	3.40	15	9.50	2.60	followup	24.00	0.48	[-0.25; 1.21]
Hoffman, CJ, et al., 2012	102	18.59	3.75	109	16.28	4.42	followup	12.00	0.56	[0.28; 0.84]
McCusker, J, et al., 2021	99	43.30	9.20	119	37.40	9.80	followup	24.00	0.62	[0.34; 0.89]
Guo, Z, et al., 2013	89	74.18	11.43	89	66.03	14.00	followup	2.00	0.64	[0.33; 0.94]
Cengiz, HO, et al., 2023	32	13.56	5.78	33	8.75	5.14	followup	8.00	0.87	[0.36; 1.38]
Beatty, L. et al. 2015	30	72.59	4.87	30	67.43	4.89	followup	13.03	1.04	[0.50; 1.59]
Beatty, L. et al. 2015	30	76.54	4.22	30	71.38	4.21	followup	26.07	1.21	[0.66; 1.76]
Henderson, VP, et al., 2013	53	18.00	0.40	58	16.90	0.30	followup	16.00	3.11	[2.55; 3.67]
Random effect	2895			3085					0.69	[0.37; 1.02]
Prediction interval										[-0.56; 1.95]
**nurse**										
Zhao,X, et al. 2015	62	5.41	1.79	62	7.24	2.31	baseline	0.00	-0.88	[-1.25; -0.51]
van der Meulen, IC, et al., 2013	88	64.80	2.40	91	66.90	2.40	baseline	0.00	-0.87	[-1.18; -0.56]
McLachlan, SA, et al., 2001	296	46.00	29.20	154	61.10	22.00	baseline	0.00	-0.56	[-0.76; -0.36]
Walczak, A, et al.,2017	61	16.76	4.80	49	17.73	4.56	baseline	0.00	-0.21	[-0.58; 0.17]
Dirksen, S. et al, 2007	34	20.10	2.80	38	20.40	3.10	baseline	0.00	-0.10	[-0.56; 0.36]
Fang, P, et al.,2020	60	46.14	5.12	60	46.55	5.23	baseline	0.00	-0.08	[-0.44; 0.28]
Thomas, ML, et al., 2012	64	16.50	5.60	88	16.70	5.30	baseline	0.00	-0.04	[-0.36; 0.29]
Klafke, N, et al., 2019	120	50.00	26.00	114	50.70	26.50	baseline	0.00	-0.03	[-0.28; 0.23]
Wu, Q, et al., 2021	43	62.34	6.76	43	62.45	6.63	baseline	0.00	-0.02	[-0.44; 0.41]
Qin, X, et al., 2017	50	73.61	10.68	50	72.41	9.34	baseline	0.00	0.12	[-0.27; 0.51]
Kim, YH, et al., 2017	30	56.40	16.50	30	53.30	16.50	baseline	0.00	0.19	[-0.32; 0.69]
Gao Q, et al. 2020	40	-56.85	4.66	40	-58.20	4.78	baseline	0.00	0.28	[-0.16; 0.72]
Kim, SH, et al., 2021	47	72.00	17.70	47	65.40	21.00	baseline	0.00	0.34	[-0.07; 0.74]
Zhao, X, et al., 2021	52	64.03	4.42	51	58.12	0.42	baseline	0.00	1.86	[1.39; 2.32]
McLachlan, SA, et al., 2001	296	45.80	30.20	154	50.70	25.00	followup	24.00	-0.17	[-0.37; 0.02]
Klafke, N, et al., 2019	120	62.80	22.30	114	64.20	22.40	followup	12.00	-0.06	[-0.32; 0.19]
Zhao,X, et al. 2015	62	7.91	2.24	62	7.81	1.98	followup	0.30	0.05	[-0.31; 0.40]
Dirksen, S. et al, 2007	34	20.80	2.30	38	20.60	4.00	followup	10.00	0.06	[-0.40; 0.52]
Klafke, N, et al., 2019	120	59.10	25.40	114	56.50	23.00	followup	24.00	0.11	[-0.15; 0.36]
Thomas, ML, et al., 2012	64	17.60	5.30	88	16.80	4.90	followup	12.00	0.16	[-0.17; 0.48]
Kim, SH, et al., 2021	47	75.90	16.30	47	71.70	18.40	followup	20.00	0.24	[-0.17; 0.65]
Walczak, A, et al.,2017	61	17.88	4.63	49	16.61	4.80	followup	4.00	0.27	[-0.11; 0.65]
Klafke, N, et al., 2019	120	65.90	25.60	114	56.30	24.70	followup	48.00	0.38	[0.12; 0.64]
Kim, SH, et al., 2021	47	79.00	15.70	47	69.60	18.90	followup	8.00	0.54	[0.12; 0.95]
Kim, YH, et al., 2017	30	67.80	15.10	30	56.70	21.90	followup	6.00	0.58	[0.07; 1.10]
Kim, YH, et al., 2017	30	70.80	14.50	30	57.80	22.60	followup	9.00	0.68	[0.15; 1.20]
Wu, Q, et al., 2021	43	81.54	8.21	43	74.21	7.33	followup	12.00	0.93	[0.49; 1.38]
Gao Q, et al. 2020	40	-49.55	4.20	40	-53.56	3.25	followup	4.00	1.06	[0.59; 1.53]
Zhou, J, et al., 2020	59	64.81	9.32	59	55.54	7.29	followup	2.00	1.10	[0.71; 1.49]
Qin, X, et al., 2017	50	90.72	8.27	50	75.60	10.01	followup	2.00	1.63	[1.18; 2.09]
van der Meulen, IC, et al., 2013	88	84.90	3.00	91	77.20	2.80	followup	48.00	2.64	[2.24; 3.05]
Fang, P, et al.,2020	60	63.52	6.47	60	47.56	5.14	followup	4.00	2.71	[2.21; 3.21]
Zhao, X, et al., 2021	52	71.02	2.31	51	58.91	5.21	followup	12.00	2.99	[2.42; 3.56]
Random effect	2470			2198					0.86	[0.45; 1.28]
Prediction interval										[-0.42; 2.14]
**Random effect**	**9862**			**10243**					**0.71**	**[0.40; 1.03]**
**Prediction interval**										**[-0.54; 1.96]**

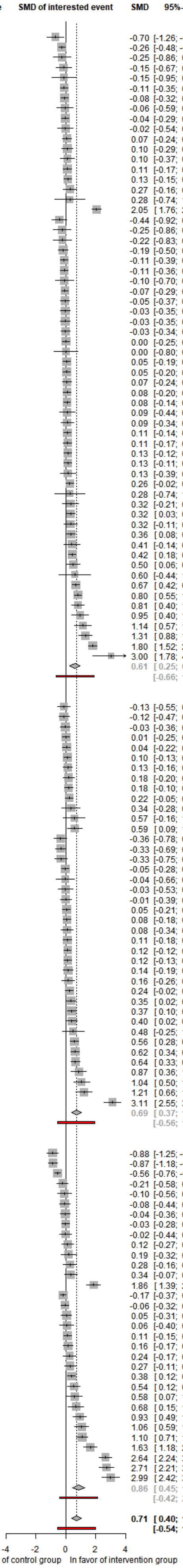

Figure S8.1. Forest plot represents the difference between the intervention vs. control group in the Emotional QoL domain with the provider subgroups as predicted at week 0 (post-intervention). SMD - Standardized mean difference, CI - confidence interval.

Figure S8.2.T12

Study	Experimental			Control						
	Patient N	Mean	SD	Patient N	Mean	SD	follow-up	Follow-up time	SMD	95%-CI
psychologist										
Seliniotaki, T, et al., 2021	27	56.10	29.10	26	74.20	20.90	baseline	0.00	-0.70	[-1.26; -0.15]
Reich, RR, et al. 2016	167	63.83	17.42	155	68.57	18.32	baseline	0.00	-0.26	[-0.48; -0.05]
Serfaty M, et al., 2018	20	12.30	5.20	22	13.60	4.90	baseline	0.00	-0.25	[-0.86; 0.36]
Peng, L, et al., 2022	28	69.94	16.25	29	72.41	16.08	baseline	0.00	-0.15	[-0.67; 0.37]
Rahmani, S, et al. 2015	12	11.11	8.94	12	12.50	9.05	baseline	0.00	-0.15	[-0.95; 0.65]
Qiu, H, et al., 2018 II.	98	11.50	6.30	196	12.22	6.56	baseline	0.00	-0.11	[-0.35; 0.13]
Qiu, H, et al., 2018 I.	98	11.72	6.65	196	12.22	6.56	baseline	0.00	-0.08	[-0.32; 0.17]
Hernandez, EG, et al. 2018	28	13.07	5.26	28	13.39	4.94	baseline	0.00	-0.06	[-0.59; 0.46]
Johansson, B, et al., 2008	128	74.00	23.00	116	75.00	23.00	baseline	0.00	-0.04	[-0.29; 0.21]
Powell, CB, et al., 2008	21	16.10	4.20	43	16.20	5.00	baseline	0.00	-0.02	[-0.54; 0.50]
Chan, et al. 2005	80	58.10	45.50	75	54.60	48.48	baseline	0.00	0.07	[-0.24; 0.39]
Liu, T, et al., 2019	49	71.18	18.38	53	69.23	22.04	baseline	0.00	0.10	[-0.29; 0.48]
Penedo, FJ, et al., 2007	41	-19.24	4.04	30	-19.63	3.43	baseline	0.00	0.10	[-0.37; 0.57]
Penedo, FJ, et al., 2020	95	-20.03	3.80	97	-20.46	3.74	baseline	0.00	0.11	[-0.17; 0.40]
Klinkhammer-Schalke, M et al., 2012	100	51.86	29.03	100	48.14	28.29	baseline	0.00	0.13	[-0.15; 0.41]
Arving, C, et al., 2007	47	69.00	25.00	38	63.00	18.00	baseline	0.00	0.27	[-0.16; 0.70]
Rodrigez, B, et al., 2014	8	88.54	22.24	7	79.76	36.28	baseline	0.00	0.28	[-0.74; 1.30]
Lu, Z, et al., 201	203	79.70	3.60	103	71.30	4.90	baseline	0.00	2.05	[1.76; 2.34]
Penedo, FJ, et al., 2007	41	-20.73	3.31	30	-19.20	3.62	followup	12.00	-0.44	[-0.92; 0.04]
Serfaty M, et al., 2018	20	15.40	4.50	22	16.60	4.90	followup	12.00	-0.25	[-0.86; 0.36]
Serfaty M, et al., 2018	20	15.60	4.20	22	16.50	3.70	followup	24.00	-0.22	[-0.83; 0.38]
Chan, et al. 2005	80	65.70	47.01	75	73.94	39.30	followup	12.00	-0.19	[-0.50; 0.13]
Klinkhammer-Schalke, M et al., 2012	100	55.34	28.29	100	58.56	28.29	followup	12.00	-0.11	[-0.39; 0.16]
Johansson, B, et al., 2008	128	83.00	18.00	116	85.00	19.00	followup	24.00	-0.11	[-0.36; 0.14]
Serfaty M, et al., 2018	20	16.60	5.50	22	17.10	4.40	followup	18.00	-0.10	[-0.70; 0.51]
Reich, RR, et al. 2016	167	71.33	19.41	155	72.71	19.13	followup	12.00	-0.07	[-0.29; 0.15]
Chan, et al. 2005	80	75.77	73.38	75	79.44	64.08	followup	72.00	-0.05	[-0.37; 0.26]
Chan, et al. 2005	80	76.73	57.02	75	78.59	56.21	followup	48.00	-0.03	[-0.35; 0.28]
Chan, et al. 2005	80	73.39	67.76	75	75.30	55.80	followup	60.00	-0.03	[-0.35; 0.28]
Chan, et al. 2005	80	68.59	48.12	75	69.81	48.88	followup	24.00	-0.03	[-0.34; 0.29]
Johansson, B, et al., 2008	128	85.00	17.00	116	85.00	17.00	followup	48.00	0.00	[-0.25; 0.25]
Rahmani, S, et al. 2015	12	12.00	48.61	12	12.00	23.61	followup	16.00	0.00	[-0.80; 0.80]
Qiu, H, et al., 2018 II.	98	12.32	9.06	196	11.90	7.89	followup	4.00	0.05	[-0.19; 0.29]
Johansson, B, et al., 2008	128	87.00	17.00	116	86.00	20.00	followup	96.00	0.05	[-0.20; 0.31]
Chan, et al. 2005	80	77.37	58.41	75	73.44	52.28	followup	36.00	0.07	[-0.24; 0.39]
Klinkhammer-Schalke, M et al., 2012	100	63.52	27.07	100	61.29	27.29	followup	48.00	0.08	[-0.20; 0.36]
Reich, RR, et al. 2016	167	70.87	18.02	155	69.36	18.78	followup	6.00	0.08	[-0.14; 0.30]
Powell, CB, et al., 2008	21	18.50	5.40	43	18.00	5.90	followup	12.00	0.09	[-0.44; 0.61]
Arving, C, et al., 2007	47	76.00	24.00	38	74.00	20.00	followup	12.00	0.09	[-0.34; 0.52]
Johansson, B, et al., 2008	128	83.00	18.00	116	81.00	19.00	followup	12.00	0.11	[-0.14; 0.36]
Penedo, FJ, et al., 2020	95	-20.18	3.80	97	-20.62	3.84	followup	24.00	0.11	[-0.17; 0.40]
Qiu, H, et al., 2018 II.	98	14.58	9.06	196	13.52	7.89	followup	24.00	0.13	[-0.12; 0.37]
Qiu, H, et al., 2018 II.	98	14.23	7.67	196	13.10	8.87	followup	12.00	0.13	[-0.11; 0.38]
Hernandez, EG, et al. 2018	28	14.00	4.69	28	13.32	5.32	followup	24.00	0.13	[-0.39; 0.66]
Klinkhammer-Schalke, M et al., 2012	100	66.50	26.80	100	59.06	29.53	followup	36.00	0.26	[-0.02; 0.54]
Rodrigez, B, et al., 2014	8	88.54	22.24	7	79.76	36.28	followup	0.10	0.28	[-0.74; 1.30]
Hernandez, EG, et al. 2018	28	16.12	4.09	28	14.72	4.63	followup	8.00	0.32	[-0.21; 0.84]
Penedo, FJ, et al., 2020	95	-19.20	3.90	97	-20.43	3.84	followup	48.00	0.32	[0.03; 0.60]
Arving, C, et al., 2007	47	82.00	20.00	38	75.00	24.00	followup	24.00	0.32	[-0.11; 0.75]
Klinkhammer-Schalke, M et al., 2012	100	66.25	25.31	100	56.58	28.54	followup	24.00	0.36	[0.08; 0.64]
Seliniotaki, T, et al., 2021	27	74.50	24.50	26	61.70	36.70	followup	8.00	0.41	[-0.14; 0.95]
Qiu, H, et al., 2018 I.	98	15.42	9.06	196	11.90	7.89	followup	4.00	0.42	[0.18; 0.67]
Arving, C, et al., 2007	47	80.00	21.00	38	69.00	23.00	followup	4.00	0.50	[0.06; 0.93]
Rodrigez, B, et al., 2014	8	93.33	10.87	7	80.56	26.79	followup	2.00	0.60	[-0.44; 1.65]
Qiu, H, et al., 2018 I.	98	19.23	9.76	196	13.10	8.87	followup	12.00	0.67	[0.42; 0.91]
Qiu, H, et al., 2018 I.	98	20.00	8.37	196	13.52	7.89	followup	24.00	0.80	[0.55; 1.05]
Liu, T, et al., 2019	49	63.49	13.78	53	51.93	14.62	followup	9.00	0.81	[0.40; 1.21]
Peng, L, et al., 2022	28	80.95	13.20	29	70.12	9.05	followup	4.00	0.95	[0.40; 1.50]
Peng, L, et al., 2022	28	83.33	14.87	29	69.25	8.94	followup	0.10	1.14	[0.57; 1.70]
Liu, T, et al., 2019	49	74.12	9.50	53	56.57	16.10	followup	12.00	1.31	[0.88; 1.73]
Lu, Z, et al., 201	203	85.40	3.50	103	77.60	5.60	followup	9.00	1.80	[1.52; 2.08]
Rahmani, S, et al. 2015	12	56.94	11.14	12	22.91	10.73	followup	8.00	3.00	[1.78; 4.23]
Random effect	4497			4960					0.40	[0.18; 0.61]
Prediction interval										[-0.85; 1.64]
healthcare professional										
Zaman, ACGNM, et al., 2021	42	44.00	9.90	46	45.30	9.80	baseline	0.00	-0.13	[-0.55; 0.29]
Berglund, G. et al. 2007	39	81.20	18.90	150	83.60	19.70	baseline	0.00	-0.12	[-0.47; 0.23]
Sandsund, C, et al., 2017	72	67.10	25.30	70	67.90	23.40	baseline	0.00	-0.03	[-0.36; 0.30]
Girgis, A, et al., 2009 II.	120	79.90	20.30	117	79.70	22.70	baseline	0.00	0.01	[-0.25; 0.26]
Girgis, A, et al., 2009 I.	110	80.70	21.60	117	79.70	22.70	baseline	0.00	0.04	[-0.22; 0.31]
Braeken APB, et al. 2013	136	78.40	20.77	144	76.12	24.16	baseline	0.00	0.10	[-0.13; 0.34]
Guo, Z, et al., 2013	89	72.57	12.51	89	70.77	14.71	baseline	0.00	0.13	[-0.16; 0.43]
Schofield, P, et al., 2013	55	77.12	22.92	53	72.93	23.08	baseline	0.00	0.18	[-0.20; 0.56]
Yun et al., 2017	134	76.80	19.40	72	73.00	23.00	baseline	0.00	0.18	[-0.10; 0.47]
Hoffman, CJ, et al., 2012	102	16.91	3.84	109	15.97	4.58	baseline	0.00	0.22	[-0.05; 0.49]
Elyasi, F, et al., 2021 II.	20	10.60	3.10	20	9.60	2.60	baseline	0.00	0.34	[-0.28; 0.97]
Elyasi, F, et al., 2021 I.	15	11.50	3.80	15	9.60	2.60	baseline	0.00	0.57	[-0.16; 1.30]
Cengiz, HO, et al., 2023	32	12.90	5.40	33	9.75	5.22	baseline	0.00	0.59	[0.09; 1.08]
Zaman, ACGNM, et al., 2021	42	47.70	9.70	46	50.80	7.50	followup	48.00	-0.36	[-0.78; 0.07]
Berglund, G. et al. 2007	39	81.60	20.10	150	87.80	18.00	followup	48.00	-0.33	[-0.69; 0.02]
Zaman, ACGNM, et al., 2021	42	46.20	10.70	46	49.40	8.30	followup	24.00	-0.33	[-0.75; 0.09]
Braeken APB, et al. 2013	136	78.38	22.75	144	79.46	20.68	followup	12.00	-0.05	[-0.28; 0.18]
Elyasi, F, et al., 2021 II.	20	9.40	2.10	20	9.50	2.60	followup	24.00	-0.04	[-0.66; 0.58]
Beatty, L. et al. 2015	30	69.03	4.81	30	69.16	4.79	followup	6.00	-0.03	[-0.53; 0.48]
Schofield, P, et al., 2013	55	75.31	22.19	53	75.51	22.22	followup	12.00	-0.01	[-0.39; 0.37]
Girgis, A, et al., 2009 II.	120	85.70	16.40	117	84.90	18.50	followup	12.00	0.05	[-0.21; 0.30]
Girgis, A, et al., 2009 I.	110	86.40	20.20	117	84.90	18.50	followup	12.00	0.08	[-0.18; 0.34]
Zaman, ACGNM, et al., 2021	42	47.60	8.80	46	46.80	10.40	followup	36.00	0.08	[-0.34; 0.50]
Yun et al., 2017	134	78.00	19.90	72	75.90	18.30	followup	48.00	0.11	[-0.18; 0.39]
Braeken APB, et al. 2013	136	83.66	20.80	144	81.23	20.60	followup	48.00	0.12	[-0.12; 0.35]
Girgis, A, et al., 2009 II.	120	86.70	18.70	117	84.40	18.90	followup	24.00	0.12	[-0.13; 0.38]
Sandsund, C, et al., 2017	72	70.00	23.90	70	66.40	26.20	followup	12.00	0.14	[-0.19; 0.47]
Zaman, ACGNM, et al., 2021	42	46.80	9.80	46	45.30	9.20	followup	12.00	0.16	[-0.26; 0.58]
Girgis, A, et al., 2009 I.	110	88.70	17.30	117	84.40	18.90	followup	24.00	0.24	[-0.02; 0.50]
Sandsund, C, et al., 2017	72	75.50	20.20	70	66.70	28.60	followup	24.00	0.35	[0.02; 0.69]
Hoffman, CJ, et al., 2012	102	18.14	3.82	109	16.59	4.40	followup	8.00	0.37	[0.10; 0.65]
Schofield, P, et al., 2013	55	81.43	20.19	53	73.23	20.40	followup	8.00	0.40	[0.02; 0.78]
Elyasi, F, et al., 2021 I.	15	11.00	3.40	15	9.50	2.60	followup	24.00	0.48	[-0.25; 1.21]
Hoffman, CJ, et al., 2012	102	18.59	3.75	109	16.28	4.42	followup	12.00	0.56	[0.28; 0.84]
McCusker, J, et al., 2021	99	43.30	9.20	119	37.40	9.80	followup	24.00	0.62	[0.34; 0.89]
Guo, Z, et al., 2013	89	74.18	11.43	89	66.03	14.00	followup	2.00	0.64	[0.33; 0.94]
Cengiz, HO, et al., 2023	32	13.56	5.78	33	8.75	5.14	followup	8.00	0.87	[0.36; 1.38]
Beatty, L. et al. 2015	30	72.59	4.87	30	67.43	4.89	followup	13.03	1.04	[0.50; 1.59]
Beatty, L. et al. 2015	30	76.54	4.22	30	71.38	4.21	followup	26.07	1.21	[0.66; 1.76]
Henderson, VP, et al., 2013	53	18.00	0.40	58	16.90	0.30	followup	16.00	3.11	[2.55; 3.67]
Random effect	2895			3085					0.48	[0.25; 0.71]
Prediction interval										[-0.77; 1.74]
nurse										
Zhao,X, et al. 2015	62	5.41	1.79	62	7.24	2.31	baseline	0.00	-0.88	[-1.25; -0.51]
van der Meulen, IC, et al., 2013	88	64.80	2.40	91	66.90	2.40	baseline	0.00	-0.87	[-1.18; -0.56]
McLachlan, SA, et al., 2001	296	46.00	29.20	154	61.10	22.00	baseline	0.00	-0.56	[-0.76; -0.36]
Walczak, A, et al.,2017	61	16.76	4.80	49	17.73	4.56	baseline	0.00	-0.21	[-0.58; 0.17]
Dirksen, S. et al, 2007	34	20.10	2.80	38	20.40	3.10	baseline	0.00	-0.10	[-0.56; 0.36]
Fang, P, et al.,2020	60	46.14	5.12	60	46.55	5.23	baseline	0.00	-0.08	[-0.44; 0.28]
Thomas, ML, et al., 2012	64	16.50	5.60	88	16.70	5.30	baseline	0.00	-0.04	[-0.36; 0.29]
Klafke, N, et al., 2019	120	50.00	26.00	114	50.70	26.50	baseline	0.00	-0.03	[-0.28; 0.23]
Wu, Q, et al., 2021	43	62.34	6.76	43	62.45	6.63	baseline	0.00	-0.02	[-0.44; 0.41]
Qin, X, et al., 2017	50	73.61	10.68	50	72.41	9.34	baseline	0.00	0.12	[-0.27; 0.51]
Kim, YH, et al., 2017	30	56.40	16.50	30	53.30	16.50	baseline	0.00	0.19	[-0.32; 0.69]
Gao Q, et al. 2020	40	-56.85	4.66	40	-58.20	4.78	baseline	0.00	0.28	[-0.16; 0.72]
Kim, SH, et al., 2021	47	72.00	17.70	47	65.40	21.00	baseline	0.00	0.34	[-0.07; 0.74]
Zhao, X, et al., 2021	52	64.03	4.42	51	58.12	0.42	baseline	0.00	1.86	[1.39; 2.32]
McLachlan, SA, et al., 2001	296	45.80	30.20	154	50.70	25.00	followup	24.00	-0.17	[-0.37; 0.02]
Klafke, N, et al., 2019	120	62.80	22.30	114	64.20	22.40	followup	12.00	-0.06	[-0.32; 0.19]
Zhao,X, et al. 2015	62	7.91	2.24	62	7.81	1.98	followup	0.30	0.05	[-0.31; 0.40]
Dirksen, S. et al, 2007	34	20.80	2.30	38	20.60	4.00	followup	10.00	0.06	[-0.40; 0.52]
Klafke, N, et al., 2019	120	59.10	25.40	114	56.50	23.00	followup	24.00	0.11	[-0.15; 0.36]
Thomas, ML, et al., 2012	64	17.60	5.30	88	16.80	4.90	followup	12.00	0.16	[-0.17; 0.48]
Kim, SH, et al., 2021	47	75.90	16.30	47	71.70	18.40	followup	20.00	0.24	[-0.17; 0.65]
Walczak, A, et al.,2017	61	17.88	4.63	49	16.61	4.80	followup	4.00	0.27	[-0.11; 0.65]
Klafke, N, et al., 2019	120	65.90	25.60	114	56.30	24.70	followup	48.00	0.38	[0.12; 0.64]
Kim, SH, et al., 2021	47	79.00	15.70	47	69.60	18.90	followup	8.00	0.54	[0.12; 0.95]
Kim, YH, et al., 2017	30	67.80	15.10	30	56.70	21.90	followup	6.00	0.58	[0.07; 1.10]
Kim, YH, et al., 2017	30	70.80	14.50	30	57.80	22.60	followup	9.00	0.68	[0.15; 1.20]
Wu, Q, et al., 2021	43	81.54	8.21	43	74.21	7.33	followup	12.00	0.93	[0.49; 1.38]
Gao Q, et al. 2020	40	-49.55	4.20	40	-53.56	3.25	followup	4.00	1.06	[0.59; 1.53]
Zhou, J, et al., 2020	59	64.81	9.32	59	55.54	7.29	followup	2.00	1.10	[0.71; 1.49]
Qin, X, et al., 2017	50	90.72	8.27	50	75.60	10.01	followup	2.00	1.63	[1.18; 2.09]
van der Meulen, IC, et al., 2013	88	84.90	3.00	91	77.20	2.80	followup	48.00	2.64	[2.24; 3.05]
Fang, P, et al.,2020	60	63.52	6.47	60	47.56	5.14	followup	4.00	2.71	[2.21; 3.21]
Zhao, X, et al., 2021	52	71.02	2.31	51	58.91	5.21	followup	12.00	2.99	[2.42; 3.56]
Random effect	2470			2198					0.65	[0.27; 1.04]
Prediction interval										[-0.65; 1.95]
**Random effect**	**9862**			**10243**					**0.49**	**[0.32; 0.66]**
**Prediction interval**										**[-0.71; 1.69]**

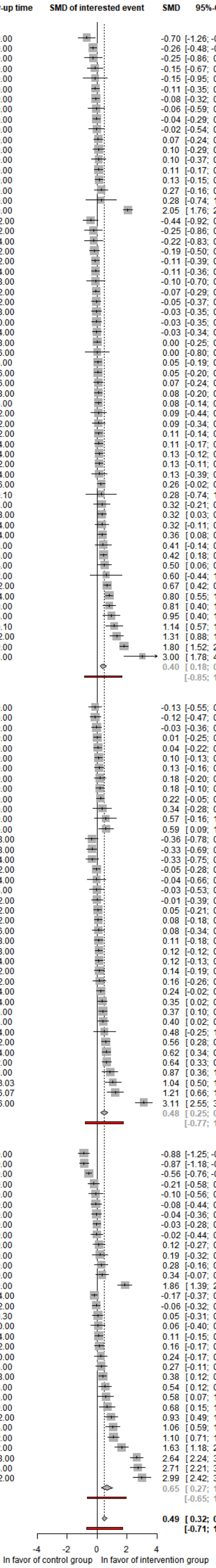

Figure S8.2. Forest plot represents the difference between the intervention vs. control group in the Emotional QoL domain with the provider subgroups as predicted at week 12 (post-intervention). SMD - Standardized mean difference, CI - confidence interval

Figure S8.3.T24

Study	Experimental Patient N	Experimental Mean	Experimental SD	Control Patient N	Control Mean	Control SD	follow-up	Follow-up time	SMD	95%-CI
psychologist										
Seliniotaki, T, et al., 2021	27	56.10	29.10	26	74.20	20.90	baseline	0.00	-0.70	[-1.26; -0.15]
Reich, RR, et al. 2016	167	63.83	17.42	155	68.57	18.32	baseline	0.00	-0.26	[-0.48; -0.05]
Serfaty M, et al., 2018	20	12.30	5.20	22	13.60	4.90	baseline	0.00	-0.25	[-0.86; 0.36]
Peng, L, et al., 2022	28	69.94	16.25	29	72.41	16.08	baseline	0.00	-0.15	[-0.67; 0.37]
Rahmani, S, et al. 2015	12	11.11	8.94	12	12.50	9.05	baseline	0.00	-0.15	[-0.95; 0.65]
Qiu, H, et al., 2018 II.	98	11.50	6.30	196	12.22	6.56	baseline	0.00	-0.11	[-0.35; 0.13]
Qiu, H, et al., 2018 I.	98	11.72	6.65	196	12.22	6.56	baseline	0.00	-0.08	[-0.32; 0.17]
Hernandez, EG, et al. 2018	28	13.07	5.26	28	13.39	4.94	baseline	0.00	-0.06	[-0.59; 0.46]
Johansson, B, et al., 2008	128	74.00	23.00	116	75.00	23.00	baseline	0.00	-0.04	[-0.29; 0.21]
Powell, CB, et al., 2008	21	16.10	4.20	43	16.20	5.00	baseline	0.00	-0.02	[-0.54; 0.50]
Chan, et al. 2005	80	58.10	45.50	75	54.60	48.48	baseline	0.00	0.07	[-0.24; 0.39]
Liu, T, et al., 2019	49	71.18	18.38	53	69.23	22.04	baseline	0.00	0.10	[-0.29; 0.48]
Penedo, FJ, et al., 2007	41	-19.24	4.04	30	-19.63	3.43	baseline	0.00	0.10	[-0.37; 0.57]
Penedo, FJ, et al., 2020	95	-20.03	3.80	97	-20.46	3.74	baseline	0.00	0.11	[-0.17; 0.40]
Klinkhammer-Schalke, M et al., 2012	100	51.86	29.03	100	48.14	28.29	baseline	0.00	0.13	[-0.15; 0.41]
Arving, C, et al., 2007	47	69.00	25.00	38	63.00	18.00	baseline	0.00	0.27	[-0.16; 0.70]
Rodrigez, B, et al., 2014	8	88.54	22.24	7	79.76	36.28	baseline	0.00	0.28	[-0.74; 1.30]
Lu, Z, et al., 201	203	79.70	3.60	103	71.30	4.90	baseline	0.00	2.05	[1.76; 2.34]
Penedo, FJ, et al., 2007	41	-20.73	3.31	30	-19.20	3.62	followup	12.00	-0.44	[-0.92; 0.04]
Serfaty M, et al., 2018	20	15.40	4.50	22	16.60	4.90	followup	12.00	-0.25	[-0.86; 0.36]
Serfaty M, et al., 2018	20	15.60	4.20	22	16.50	3.70	followup	24.00	-0.22	[-0.83; 0.38]
Chan, et al. 2005	80	65.70	47.01	75	73.94	39.30	followup	12.00	-0.19	[-0.50; 0.13]
Klinkhammer-Schalke, M et al., 2012	100	55.34	28.29	100	58.56	28.29	followup	12.00	-0.11	[-0.39; 0.16]
Johansson, B, et al., 2008	128	83.00	18.00	116	85.00	19.00	followup	24.00	-0.11	[-0.36; 0.14]
Serfaty M, et al., 2018	20	16.60	5.50	22	17.10	4.40	followup	18.00	-0.10	[-0.70; 0.51]
Reich, RR, et al. 2016	167	71.33	19.41	155	72.71	19.13	followup	12.00	-0.07	[-0.29; 0.15]
Chan, et al. 2005	80	75.77	73.38	75	79.44	64.08	followup	72.00	-0.05	[-0.37; 0.26]
Chan, et al. 2005	80	76.73	57.02	75	78.59	56.21	followup	48.00	-0.03	[-0.35; 0.28]
Chan, et al. 2005	80	73.39	67.76	75	75.30	55.80	followup	60.00	-0.03	[-0.35; 0.28]
Chan, et al. 2005	80	68.59	48.12	75	69.81	48.88	followup	24.00	-0.03	[-0.34; 0.29]
Johansson, B, et al., 2008	128	85.00	17.00	116	85.00	17.00	followup	48.00	0.00	[-0.25; 0.25]
Rahmani, S, et al. 2015	12	12.00	48.61	12	12.00	23.61	followup	16.00	0.00	[-0.80; 0.80]
Qiu, H, et al., 2018 II.	98	12.32	9.06	196	11.90	7.89	followup	4.00	0.05	[-0.19; 0.29]
Johansson, B, et al., 2008	128	87.00	17.00	116	86.00	20.00	followup	96.00	0.05	[-0.20; 0.31]
Chan, et al. 2005	80	77.37	58.41	75	73.44	52.28	followup	36.00	0.07	[-0.24; 0.39]
Klinkhammer-Schalke, M et al., 2012	100	63.52	27.07	100	61.29	27.29	followup	48.00	0.08	[-0.20; 0.36]
Reich, RR, et al. 2016	167	70.87	18.02	155	69.36	18.78	followup	6.00	0.08	[-0.14; 0.30]
Powell, CB, et al., 2008	21	18.50	5.40	43	18.00	5.90	followup	12.00	0.09	[-0.44; 0.61]
Arving, C, et al., 2007	47	76.00	24.00	38	74.00	20.00	followup	12.00	0.09	[-0.34; 0.52]
Johansson, B, et al., 2008	128	83.00	18.00	116	81.00	19.00	followup	12.00	0.11	[-0.14; 0.36]
Penedo, FJ, et al., 2020	95	-20.18	3.80	97	-20.62	3.84	followup	24.00	0.11	[-0.17; 0.40]
Qiu, H, et al., 2018 II.	98	14.58	9.06	196	13.52	7.89	followup	24.00	0.13	[-0.12; 0.37]
Qiu, H, et al., 2018 II.	98	14.23	7.67	196	13.10	8.87	followup	12.00	0.13	[-0.11; 0.38]
Hernandez, EG, et al. 2018	28	14.00	4.69	28	13.32	5.32	followup	24.00	0.13	[-0.39; 0.66]
Klinkhammer-Schalke, M et al., 2012	100	66.50	26.80	100	59.06	29.53	followup	36.00	0.26	[-0.02; 0.54]
Rodrigez, B, et al., 2014	8	88.54	22.24	7	79.76	36.28	followup	0.10	0.28	[-0.74; 1.30]
Hernandez, EG, et al. 2018	28	16.12	4.09	28	14.72	4.63	followup	8.00	0.32	[-0.21; 0.84]
Penedo, FJ, et al., 2020	95	-19.20	3.90	97	-20.43	3.84	followup	48.00	0.32	[0.03; 0.60]
Arving, C, et al., 2007	47	82.00	20.00	38	75.00	24.00	followup	24.00	0.32	[-0.11; 0.75]
Klinkhammer-Schalke, M et al., 2012	100	66.25	25.31	100	56.58	28.54	followup	24.00	0.36	[0.08; 0.64]
Seliniotaki, T, et al., 2021	27	74.50	24.50	26	61.70	36.70	followup	8.00	0.41	[-0.14; 0.95]
Qiu, H, et al., 2018 I.	98	15.42	9.06	196	11.90	7.89	followup	4.00	0.42	[0.18; 0.67]
Arving, C, et al., 2007	47	80.00	21.00	38	69.00	23.00	followup	4.00	0.50	[0.06; 0.93]
Rodrigez, B, et al., 2014	8	93.33	10.87	7	80.56	26.79	followup	2.00	0.60	[-0.44; 1.65]
Qiu, H, et al., 2018 I.	98	19.23	9.78	196	13.10	8.87	followup	12.00	0.67	[0.42; 0.91]
Qiu, H, et al., 2018 I.	98	20.00	8.37	196	13.52	7.89	followup	24.00	0.80	[0.55; 1.05]
Liu, T, et al., 2019	49	63.49	13.78	53	51.93	14.62	followup	9.00	0.81	[0.40; 1.21]
Peng, L, et al., 2022	28	80.95	13.20	29	70.12	9.05	followup	4.00	0.95	[0.40; 1.50]
Peng, L, et al., 2022	28	83.33	14.87	29	69.25	8.94	followup	0.10	1.14	[0.57; 1.70]
Liu, T, et al., 2019	49	74.12	9.50	53	56.57	16.10	followup	12.00	1.31	[0.88; 1.73]
Lu, Z, et al., 201	203	85.40	3.50	103	77.60	5.60	followup	9.00	1.80	[1.52; 2.08]
Rahmani, S, et al. 2015	12	56.94	11.14	12	22.91	10.73	followup	8.00	3.00	[1.78; 4.23]
Random effect	4497			4960					0.24	[0.03; 0.44]
Prediction interval										[-1.05; 1.52]
health care professional										
Zaman, ACGNM, et al., 2021	42	44.00	9.90	46	45.30	9.80	baseline	0.00	-0.13	[-0.55; 0.29]
Berglund, G. et al. 2007	39	81.20	18.90	150	83.60	19.70	baseline	0.00	-0.12	[-0.47; 0.23]
Sandsund, C, et al., 2017	72	67.10	25.30	70	67.90	23.40	baseline	0.00	-0.03	[-0.36; 0.30]
Girgis, A, et al., 2009 II.	120	79.90	20.30	117	79.70	22.70	baseline	0.00	0.01	[-0.25; 0.26]
Girgis, A, et al., 2009 I.	110	80.70	21.60	117	79.70	22.70	baseline	0.00	0.04	[-0.22; 0.31]
Braeken APB, et al. 2013	136	78.40	20.77	144	76.12	24.16	baseline	0.00	0.10	[-0.13; 0.34]
Guo, Z, et al., 2013	89	72.57	12.51	89	70.77	14.71	baseline	0.00	0.13	[-0.16; 0.43]
Schofield, P, et al., 2013	55	77.12	22.92	53	72.93	23.08	baseline	0.00	0.18	[-0.20; 0.56]
Yun et al., 2017	134	76.80	19.40	72	73.00	23.00	baseline	0.00	0.18	[-0.10; 0.47]
Hoffman, CJ, et al., 2012	102	16.91	3.84	109	15.97	4.58	baseline	0.00	0.22	[-0.05; 0.49]
Elyasi, F, et al., 2021 II.	20	10.60	3.10	20	9.60	2.60	baseline	0.00	0.34	[-0.28; 0.97]
Elyasi, F, et al., 2021 I.	15	11.50	3.80	15	9.60	2.60	baseline	0.00	0.57	[-0.16; 1.30]
Cengiz, HO, et al., 2023	32	12.90	5.40	33	9.75	5.22	baseline	0.00	0.59	[0.09; 1.08]
Zaman, ACGNM, et al., 2021	42	47.70	9.70	46	50.80	7.50	followup	48.00	-0.36	[-0.78; 0.07]
Berglund, G. et al. 2007	39	81.60	20.10	150	87.80	18.00	followup	48.00	-0.33	[-0.69; 0.02]
Zaman, ACGNM, et al., 2021	42	46.20	10.70	46	49.40	8.30	followup	24.00	-0.33	[-0.75; 0.09]
Braeken APB, et al. 2013	136	78.38	22.75	144	79.46	20.68	followup	12.00	-0.05	[-0.28; 0.18]
Elyasi, F, et al., 2021 II.	20	9.40	2.10	20	9.50	2.60	followup	24.00	-0.04	[-0.66; 0.58]
Beatty, L. et al. 2015	30	69.03	4.81	30	69.16	4.79	followup	6.00	-0.03	[-0.53; 0.48]
Schofield, P, et al., 2013	55	75.31	22.19	53	75.51	22.22	followup	12.00	-0.01	[-0.39; 0.37]
Girgis, A, et al., 2009 II.	120	85.70	16.40	117	84.90	18.50	followup	12.00	0.05	[-0.21; 0.30]
Girgis, A, et al., 2009 I.	110	86.40	20.20	117	84.90	18.50	followup	12.00	0.08	[-0.18; 0.34]
Zaman, ACGNM, et al., 2021	42	47.60	8.80	46	46.80	10.40	followup	36.00	0.08	[-0.34; 0.50]
Yun et al., 2017	134	78.00	19.90	72	75.90	18.30	followup	48.00	0.11	[-0.18; 0.39]
Braeken APB, et al. 2013	136	83.66	20.80	144	81.23	20.60	followup	48.00	0.12	[-0.12; 0.35]
Girgis, A, et al., 2009 II.	120	86.70	18.70	117	84.40	18.90	followup	24.00	0.12	[-0.13; 0.38]
Sandsund, C, et al., 2017	72	70.00	23.90	70	66.40	26.20	followup	12.00	0.14	[-0.19; 0.47]
Zaman, ACGNM, et al., 2021	42	46.80	9.80	46	45.30	9.20	followup	12.00	0.16	[-0.26; 0.58]
Girgis, A, et al., 2009 I.	110	88.70	17.30	117	84.40	18.90	followup	24.00	0.24	[-0.02; 0.50]
Sandsund, C, et al., 2017	72	75.50	20.20	70	66.70	28.60	followup	24.00	0.35	[0.02; 0.69]
Hoffman, CJ, et al., 2012	102	18.14	3.82	109	16.59	4.40	followup	8.00	0.37	[0.10; 0.65]
Schofield, P, et al., 2013	55	81.43	20.19	53	73.23	20.40	followup	8.00	0.40	[0.02; 0.78]
Elyasi, F, et al., 2021 I.	15	11.00	3.40	15	9.50	2.60	followup	24.00	0.48	[-0.25; 1.21]
Hoffman, CJ, et al., 2012	102	18.59	3.75	109	16.28	4.42	followup	12.00	0.56	[0.28; 0.84]
McCusker, J, et al., 2021	99	43.30	9.20	119	37.40	9.80	followup	24.00	0.62	[0.34; 0.89]
Guo, Z, et al., 2013	89	74.18	11.43	89	66.03	14.00	followup	2.00	0.64	[0.33; 0.94]
Cengiz, HO, et al., 2023	32	13.56	5.78	33	8.75	5.14	followup	8.00	0.87	[0.36; 1.38]
Beatty, L. et al. 2015	30	72.59	4.87	30	67.43	4.89	followup	13.03	1.04	[0.50; 1.59]
Beatty, L. et al. 2015	30	76.54	4.22	30	71.38	4.21	followup	26.07	1.21	[0.66; 1.76]
Henderson, VP, et al., 2013	53	18.00	0.40	58	16.90	0.30	followup	16.00	3.11	[2.55; 3.67]
Random effect	2895			3085					0.32	[0.06; 0.58]
Prediction interval										[-0.95; 1.59]
nurse										
Zhao,X, et al. 2015	62	5.41	1.79	62	7.24	2.31	baseline	0.00	-0.88	[-1.25; -0.51]
van der Meulen, IC, et al., 2013	88	64.80	2.40	91	66.90	2.40	baseline	0.00	-0.87	[-1.18; -0.56]
McLachlan, SA, et al., 2001	296	46.00	29.20	154	61.10	22.00	baseline	0.00	-0.56	[-0.76; -0.36]
Walczak, A, et al. ,2017	61	16.76	4.80	49	17.73	4.56	baseline	0.00	-0.21	[-0.58; 0.17]
Dirksen, S. et al, 2007	34	20.10	2.80	38	20.40	3.10	baseline	0.00	-0.10	[-0.56; 0.36]
Fang, P. et al.,2020	60	46.14	5.12	60	46.55	5.23	baseline	0.00	-0.08	[-0.44; 0.28]
Thomas, ML, et al., 2012	64	16.50	5.60	88	16.70	5.30	baseline	0.00	-0.04	[-0.36; 0.29]
Klafke, N, et al., 2019	120	50.00	26.00	114	50.70	26.50	baseline	0.00	-0.03	[-0.28; 0.23]
Wu, Q, et al., 2021	43	62.34	6.76	43	62.45	6.63	baseline	0.00	-0.02	[-0.44; 0.41]
Qin, X, et al., 2017	50	73.61	10.68	50	72.41	9.34	baseline	0.00	0.12	[-0.27; 0.51]
Kim, YH, et al., 2017	30	56.40	16.50	30	53.30	16.50	baseline	0.00	0.19	[-0.32; 0.69]
Gao Q, et al. 2020	40	-56.85	4.66	40	-58.20	4.78	baseline	0.00	0.28	[-0.16; 0.72]
Kim, SH, et al., 2021	47	72.00	17.70	47	65.40	21.00	baseline	0.00	0.34	[-0.07; 0.74]
Zhao, X, et al., 2021	52	64.03	4.42	51	58.12	0.42	baseline	0.00	1.86	[1.39; 2.32]
McLachlan, SA, et al., 2001	296	45.80	30.20	154	50.70	25.00	followup	24.00	-0.17	[-0.37; 0.02]
Klafke, N, et al., 2019	120	62.80	22.30	114	64.20	22.40	followup	12.00	-0.06	[-0.32; 0.19]
Zhao,X, et al. 2015	62	7.91	2.24	62	7.81	1.98	followup	0.30	0.05	[-0.31; 0.40]
Dirksen, S. et al, 2007	34	20.80	2.30	38	20.60	4.00	followup	10.00	0.06	[-0.40; 0.52]
Klafke, N, et al., 2019	120	59.10	25.40	114	56.50	23.00	followup	24.00	0.11	[-0.15; 0.36]
Thomas, ML, et al., 2012	64	17.60	5.30	88	16.80	4.90	followup	12.00	0.16	[-0.17; 0.48]
Kim, SH, et al., 2021	47	75.90	16.30	47	71.70	18.40	followup	20.00	0.24	[-0.17; 0.65]
Walczak, A, et al. ,2017	61	17.88	4.63	49	16.61	4.80	followup	4.00	0.27	[-0.11; 0.65]
Klafke, N, et al., 2019	120	65.90	25.60	114	56.30	24.70	followup	48.00	0.38	[0.12; 0.64]
Kim, SH, et al., 2021	47	79.00	15.70	47	69.60	18.90	followup	8.00	0.54	[0.12; 0.95]
Kim, YH, et al., 2017	30	67.80	15.10	30	56.70	21.90	followup	5.00	0.58	[0.07; 1.10]
Kim, YH, et al., 2017	30	70.80	14.50	30	57.80	22.60	followup	9.00	0.68	[0.15; 1.20]
Wu, Q, et al., 2021	43	81.54	8.21	43	74.21	7.33	followup	12.00	0.93	[0.49; 1.38]
Gao Q, et al. 2020	40	-49.55	4.20	40	-53.56	3.25	followup	4.00	1.06	[0.59; 1.53]
Zhou, J, et al., 2020	59	64.81	9.32	59	55.54	7.29	followup	2.00	1.10	[0.71; 1.49]
Qin, X, et al., 2017	50	90.72	8.27	50	75.60	10.01	followup	2.00	1.63	[1.18; 2.09]
van der Meulen, IC, et al., 2013	88	84.90	3.00	91	77.20	2.80	followup	48.00	2.64	[2.24; 3.05]
Fang, P. et al.,2020	60	63.52	6.47	60	47.56	5.14	followup	4.00	2.71	[2.21; 3.21]
Zhao, X, et al., 2021	52	71.02	2.31	51	58.91	5.21	followup	12.00	2.99	[2.42; 3.56]
Random effect	2470			2190					0.49	[0.06; 0.92]
Prediction interval										[-0.82; 1.80]
**Random effect**	**9862**			**10243**					**0.31**	**[0.12; 0.50]**
**Prediction interval**										**[-0.94; 1.56]**

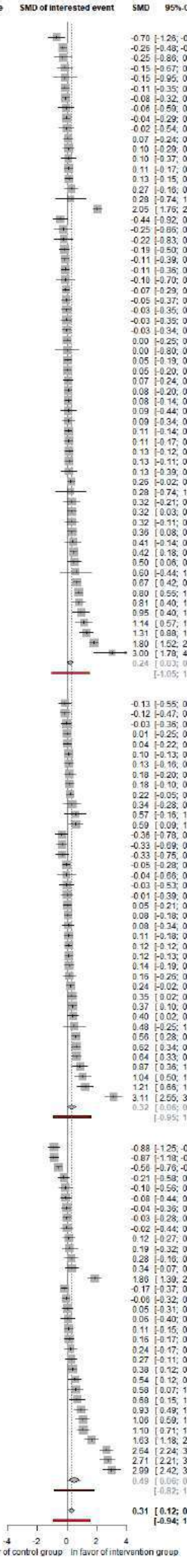

Figure S8.3. Forest plot represents the difference between the intervention vs. control group in the Emotional QoL domain with the provider subgroups as predicted at week 24 (post-intervention). SMD - Standardized mean difference, CI - confidence interval

Figure S8.4.T48

Study	Experimental Patient N	Experimental Mean	Experimental SD	Control Patient N	Control Mean	Control SD	follow-up	Follow-up time	SMD	95%-CI
**psychologist**										
Seliniotaki, T, et al., 2021	27	56.10	29.10	26	74.20	20.90	baseline	0.00	-0.70	[-1.26; -0.15]
Reich, RR, et al. 2016	167	63.83	17.42	155	68.57	18.32	baseline	0.00	-0.26	[-0.48; -0.05]
Serfaty M, et al., 2018	20	12.30	5.20	22	13.60	4.90	baseline	0.00	-0.25	[-0.86; 0.36]
Peng, L, et al., 2022	28	69.94	16.25	29	72.41	16.08	baseline	0.00	-0.15	[-0.67; 0.37]
Rahmani, S, et al. 2015	12	11.11	8.94	12	12.50	9.05	baseline	0.00	-0.15	[-0.95; 0.65]
Qiu, H, et al., 2018 II.	98	11.50	6.30	196	12.22	6.56	baseline	0.00	-0.11	[-0.35; 0.13]
Qiu, H, et al., 2018 I.	98	11.72	6.65	196	12.22	6.56	baseline	0.00	-0.08	[-0.32; 0.17]
Hernandez, EG, et al. 2018	28	13.07	5.26	28	13.39	4.94	baseline	0.00	-0.06	[-0.59; 0.46]
Johansson, B, et al., 2008	128	74.00	23.00	116	75.00	23.00	baseline	0.00	-0.04	[-0.29; 0.21]
Powell, CB, et al., 2008	21	16.10	4.20	43	16.20	5.00	baseline	0.00	-0.02	[-0.54; 0.50]
Chan, et al. 2005	80	58.10	45.50	75	54.60	48.48	baseline	0.00	0.07	[-0.24; 0.39]
Liu, T, et al., 2019	49	71.18	18.38	53	69.23	22.04	baseline	0.00	0.10	[-0.29; 0.48]
Penedo, FJ, et al., 2007	41	-19.24	4.04	30	-19.63	3.43	baseline	0.00	0.10	[-0.37; 0.57]
Penedo, FJ, et al., 2020	95	-20.03	3.80	97	-20.46	3.74	baseline	0.00	0.11	[-0.17; 0.40]
Klinkhammer-Schalke, M et al., 2012	100	51.86	29.03	100	48.14	28.29	baseline	0.00	0.13	[-0.15; 0.41]
Arving, C, et al., 2007	47	69.00	25.00	38	63.00	18.00	baseline	0.00	0.27	[-0.16; 0.70]
Rodrigez, B, et al., 2014	8	88.54	22.24	7	79.76	36.28	baseline	0.00	0.28	[-0.74; 1.30]
Lu, Z, et al., 201	203	79.70	3.60	103	71.30	4.90	baseline	0.00	2.05	[1.76; 2.34]
Penedo, FJ, et al., 2007	41	-20.73	3.31	30	-19.20	3.62	followup	12.00	-0.44	[-0.92; 0.04]
Serfaty M, et al., 2018	20	15.40	4.50	22	16.60	4.90	followup	12.00	-0.25	[-0.86; 0.36]
Serfaty M, et al., 2018	20	15.60	4.20	22	16.50	3.70	followup	24.00	-0.22	[-0.83; 0.38]
Chan, et al. 2005	80	65.70	47.01	75	73.94	39.30	followup	12.00	-0.19	[-0.50; 0.13]
Klinkhammer-Schalke, M et al., 2012	100	55.34	28.29	100	58.56	28.29	followup	12.00	-0.11	[-0.39; 0.18]
Johansson, B, et al., 2008	128	83.00	18.00	116	85.00	19.00	followup	24.00	-0.11	[-0.36; 0.14]
Serfaty M, et al., 2018	20	16.60	5.50	22	17.10	4.40	followup	18.00	-0.10	[-0.70; 0.51]
Reich, RR, et al. 2016	167	71.33	19.41	155	72.71	19.13	followup	12.00	-0.07	[-0.29; 0.15]
Chan, et al. 2005	80	75.77	73.38	75	79.44	64.08	followup	72.00	-0.05	[-0.37; 0.26]
Chan, et al. 2005	80	76.73	57.02	75	78.59	56.21	followup	48.00	-0.03	[-0.35; 0.28]
Chan, et al. 2005	80	73.39	67.76	75	75.30	55.80	followup	60.00	-0.03	[-0.35; 0.28]
Chan, et al. 2005	80	68.59	48.12	75	69.81	48.88	followup	24.00	-0.03	[-0.34; 0.29]
Johansson, B, et al., 2008	128	85.00	17.00	116	85.00	17.00	followup	48.00	0.00	[-0.25; 0.25]
Rahmani, S, et al. 2015	12	12.00	48.61	12	12.00	23.61	followup	16.00	0.00	[-0.80; 0.80]
Qiu, H, et al., 2018 II.	98	12.32	9.06	196	11.90	7.89	followup	4.00	0.05	[-0.19; 0.29]
Johansson, B, et al., 2008	128	87.00	17.00	116	86.00	20.00	followup	96.00	0.05	[-0.20; 0.31]
Chan, et al. 2005	80	77.37	58.41	75	73.44	52.26	followup	36.00	0.07	[-0.24; 0.39]
Klinkhammer-Schalke, M et al., 2012	100	63.52	27.07	100	61.29	27.29	followup	48.00	0.08	[-0.20; 0.36]
Reich, RR, et al. 2016	167	70.87	18.02	155	69.36	18.76	followup	6.00	0.08	[-0.14; 0.30]
Powell, CB, et al., 2008	21	18.50	5.40	43	18.00	5.90	followup	12.00	0.09	[-0.44; 0.61]
Arving, C, et al., 2007	47	76.00	24.00	38	74.00	20.00	followup	12.00	0.09	[-0.34; 0.52]
Johansson, B, et al., 2008	128	83.00	18.00	116	81.00	19.00	followup	12.00	0.11	[-0.14; 0.36]
Penedo, FJ, et al., 2020	95	-20.18	3.80	97	-20.62	3.84	followup	24.00	0.11	[-0.17; 0.40]
Qiu, H, et al., 2018 II.	98	14.58	9.06	196	13.52	7.89	followup	24.00	0.13	[-0.12; 0.37]
Qiu, H, et al., 2018 II.	98	14.23	7.67	196	13.10	8.87	followup	12.00	0.13	[-0.11; 0.38]
Hernandez, EG, et al. 2018	28	14.00	4.69	28	13.32	5.32	followup	24.00	0.13	[-0.39; 0.66]
Klinkhammer-Schalke, M et al., 2012	100	66.50	26.80	100	59.06	29.53	followup	36.00	0.26	[-0.02; 0.54]
Rodrigez, B, et al., 2014	8	88.54	22.24	7	79.76	36.28	followup	0.10	0.28	[-0.74; 1.30]
Hernandez, EG, et al. 2018	28	16.12	4.09	28	14.72	4.63	followup	8.00	0.32	[-0.21; 0.84]
Penedo, FJ, et al., 2020	95	-19.20	3.90	97	-20.43	3.84	followup	48.00	0.32	[0.03; 0.60]
Arving, C, et al., 2007	47	82.00	20.00	38	75.00	24.00	followup	24.00	0.32	[-0.11; 0.75]
Klinkhammer-Schalke, M et al., 2012	100	66.25	25.31	100	56.58	28.54	followup	24.00	0.36	[0.08; 0.64]
Seliniotaki, T, et al., 2021	27	74.50	24.50	26	61.70	36.70	followup	8.00	0.41	[-0.14; 0.95]
Qiu, H, et al., 2018 I.	98	15.42	9.06	196	11.90	7.89	followup	4.00	0.42	[0.18; 0.67]
Arving, C, et al., 2007	47	80.00	21.00	38	69.00	23.00	followup	4.00	0.50	[0.06; 0.93]
Rodrigez, B, et al., 2014	8	93.33	10.87	7	80.56	26.79	followup	2.00	0.60	[-0.44; 1.65]
Qiu, H, et al., 2018 I.	98	19.23	9.76	196	13.10	8.87	followup	12.00	0.67	[0.42; 0.91]
Qiu, H, et al., 2018 I.	98	20.00	8.37	196	13.52	7.89	followup	24.00	0.80	[0.55; 1.05]
Liu, T, et al., 2019	49	63.49	13.78	53	51.93	14.62	followup	9.00	0.81	[0.40; 1.21]
Peng, L, et al., 2022	28	80.95	13.20	29	70.12	9.05	followup	4.00	0.95	[0.40; 1.50]
Peng, L, et al., 2022	28	83.33	14.87	29	69.25	8.94	followup	0.10	1.14	[0.57; 1.70]
Liu, T, et al., 2019	49	74.12	9.50	53	56.57	16.10	followup	12.00	1.31	[0.88; 1.73]
Lu, Z, et al., 201	203	85.40	3.50	103	77.60	5.60	followup	9.00	1.80	[1.52; 2.08]
Rahmani, S, et al. 2015	12	56.94	11.14	12	22.91	10.73	followup	8.00	3.00	[1.78; 4.23]
Random effect	4497			4960					0.05	[-0.26; 0.36]
Prediction interval										[-1.31; 1.41]
**healthcare professional**										
Zaman, ACGNM, et al., 2021	42	44.00	9.90	46	45.30	9.80	baseline	0.00	-0.13	[-0.55; 0.29]
Berglund, G. et al. 2007	39	81.20	18.90	150	83.60	19.70	baseline	0.00	-0.12	[-0.47; 0.23]
Sandsund, C, et al., 2017	72	67.10	25.30	70	67.90	23.40	baseline	0.00	-0.03	[-0.36; 0.30]
Girgis, A, et al., 2009 II.	120	79.90	20.30	117	79.70	22.70	baseline	0.00	0.01	[-0.25; 0.26]
Girgis, A, et al., 2009 I.	110	80.70	21.60	117	79.70	22.70	baseline	0.00	0.04	[-0.22; 0.31]
Braeken APB, et al. 2013	136	78.40	20.77	144	76.12	24.16	baseline	0.00	0.10	[-0.13; 0.34]
Guo, Z, et al., 2013	89	72.57	12.51	89	70.77	14.71	baseline	0.00	0.13	[-0.16; 0.43]
Schofield, P, et al., 2013	55	77.12	22.92	53	72.93	23.08	baseline	0.00	0.18	[-0.20; 0.56]
Yun et al., 2017	134	76.80	19.40	72	73.00	23.00	baseline	0.00	0.18	[-0.10; 0.47]
Hoffman, CJ, et al., 2012	102	16.91	3.84	109	15.97	4.58	baseline	0.00	0.22	[-0.05; 0.49]
Elyasi, F, et al., 2021 II.	20	10.60	3.10	20	9.60	2.60	baseline	0.00	0.34	[-0.28; 0.97]
Elyasi, F, et al., 2021 I.	15	11.50	3.80	15	9.60	2.60	baseline	0.00	0.57	[-0.16; 1.30]
Cengiz, HO, et al., 2023	32	12.90	5.40	33	9.75	5.22	baseline	0.00	0.59	[0.09; 1.08]
Zaman, ACGNM, et al., 2021	42	47.70	9.70	46	50.60	7.50	followup	48.00	-0.36	[-0.78; 0.07]
Berglund, G. et al. 2007	39	81.60	20.10	150	87.80	18.00	followup	48.00	-0.33	[-0.69; 0.02]
Zaman, ACGNM, et al., 2021	42	46.20	10.70	46	49.40	8.30	followup	24.00	-0.33	[-0.75; 0.09]
Braeken APB, et al. 2013	136	78.38	22.75	144	79.46	20.68	followup	12.00	-0.05	[-0.28; 0.18]
Elyasi, F, et al., 2021 II.	20	9.40	2.10	20	9.50	2.60	followup	24.00	-0.04	[-0.66; 0.58]
Beatty, L. et al. 2015	30	69.03	4.81	30	69.16	4.79	followup	6.00	-0.03	[-0.53; 0.48]
Schofield, P, et al., 2013	55	75.31	22.19	53	75.51	22.22	followup	12.00	-0.01	[-0.39; 0.37]
Girgis, A, et al., 2009 II.	120	85.70	16.40	117	84.90	18.50	followup	12.00	0.05	[-0.21; 0.30]
Girgis, A, et al., 2009 I.	110	86.40	20.20	117	84.90	18.50	followup	12.00	0.08	[-0.18; 0.34]
Zaman, ACGNM, et al., 2021	42	47.60	8.80	46	46.80	10.40	followup	36.00	0.08	[-0.34; 0.50]
Yun et al., 2017	134	78.00	19.90	72	75.90	18.30	followup	48.00	0.11	[-0.18; 0.39]
Braeken APB, et al. 2013	136	83.66	20.80	144	81.23	20.60	followup	48.00	0.12	[-0.12; 0.35]
Girgis, A, et al., 2009 II.	120	86.70	18.70	117	84.40	18.90	followup	24.00	0.12	[-0.13; 0.38]
Sandsund, C, et al., 2017	72	70.00	23.90	70	66.40	26.20	followup	12.00	0.14	[-0.19; 0.47]
Zaman, ACGNM, et al., 2021	42	46.80	9.80	46	45.30	9.20	followup	12.00	0.16	[-0.26; 0.58]
Girgis, A, et al., 2009 I.	110	88.70	17.30	117	84.40	18.90	followup	24.00	0.24	[-0.02; 0.50]
Sandsund, C, et al., 2017	72	75.50	20.20	70	66.70	28.60	followup	24.00	0.35	[0.02; 0.69]
Hoffman, CJ, et al., 2012	102	18.14	3.82	109	16.59	4.40	followup	6.00	0.37	[0.10; 0.65]
Schofield, P, et al., 2013	55	81.43	20.19	53	73.23	20.40	followup	8.00	0.40	[0.02; 0.78]
Elyasi, F, et al., 2021 I.	15	11.00	3.40	15	9.50	2.60	followup	24.00	0.48	[-0.25; 1.21]
Hoffman, CJ, et al., 2012	102	18.59	3.75	109	16.28	4.42	followup	12.00	0.56	[0.28; 0.84]
McCusker, J, et al., 2021	99	43.30	9.20	119	37.40	9.80	followup	24.00	0.62	[0.34; 0.89]
Guo, Z, et al., 2013	89	74.18	11.43	89	66.03	14.00	followup	2.00	0.64	[0.33; 0.94]
Cengiz, HO, et al., 2023	32	13.56	5.78	33	8.75	5.14	followup	8.00	0.87	[0.36; 1.38]
Beatty, L. et al. 2015	30	72.59	4.87	30	67.43	4.89	followup	13.03	1.04	[0.50; 1.59]
Beatty, L. et al. 2015	30	76.54	4.22	30	71.38	4.21	followup	26.07	1.21	[0.66; 1.76]
Henderson, VP, et al., 2013	53	18.00	0.40	58	16.90	0.30	followup	16.00	3.11	[2.55; 3.67]
Random effect	2895			3005					0.13	[-0.23; 0.50]
Prediction interval										[-1.16; 1.43]
**nurse**										
Zhao,X, et al. 2015	62	5.41	1.79	62	7.24	2.31	baseline	0.00	-0.88	[-1.25; -0.51]
van der Meulen, IC, et al., 2013	88	64.80	2.40	91	66.90	2.40	baseline	0.00	-0.87	[-1.18; -0.56]
McLachlan, SA, et al., 2001	296	46.00	29.20	154	61.10	22.00	baseline	0.00	-0.56	[-0.76; -0.36]
Walczak, A, et al.,2017	61	16.76	4.80	49	17.73	4.56	baseline	0.00	-0.21	[-0.58; 0.17]
Dirksen, S. et al. 2007	34	20.10	2.80	38	20.40	3.10	baseline	0.00	-0.10	[-0.56; 0.36]
Fang, P, et al.,2020	60	46.14	5.12	60	46.55	5.23	baseline	0.00	-0.08	[-0.44; 0.28]
Thomas, ML, et al., 2012	64	16.50	5.60	88	16.70	5.30	baseline	0.00	-0.04	[-0.36; 0.29]
Klafke, N, et al., 2019	120	50.00	26.00	114	50.70	26.50	baseline	0.00	-0.03	[-0.28; 0.23]
Wu, Q, et al., 2021	43	62.34	6.76	43	62.45	6.63	baseline	0.00	-0.02	[-0.44; 0.41]
Qin, X, et al., 2017	50	73.61	10.68	50	72.41	9.34	baseline	0.00	0.12	[-0.27; 0.51]
Kim, YH, et al., 2017	30	56.40	16.50	30	53.30	16.50	baseline	0.00	0.19	[-0.32; 0.69]
Gao Q, et al. 2020	40	-56.85	4.66	40	-58.20	4.78	baseline	0.00	0.28	[-0.16; 0.72]
Kim, SH, et al., 2021	47	72.00	17.70	47	65.40	21.00	baseline	0.00	0.34	[-0.07; 0.74]
Zhao, X, et al., 2021	52	64.03	4.42	51	58.12	0.42	baseline	0.00	1.86	[1.39; 2.32]
McLachlan, SA, et al., 2001	296	45.80	30.20	154	50.70	25.00	followup	24.00	-0.17	[-0.37; 0.02]
Klafke, N, et al., 2019	120	62.80	22.30	114	64.20	22.40	followup	12.00	-0.06	[-0.32; 0.19]
Zhao,X, et al. 2015	62	7.91	2.24	62	7.81	1.98	followup	0.30	0.05	[-0.31; 0.40]
Dirksen, S. et al. 2007	34	20.80	2.30	38	20.60	4.00	followup	10.00	0.06	[-0.40; 0.52]
Klafke, N, et al., 2019	120	59.10	25.40	114	56.50	23.00	followup	24.00	0.11	[-0.15; 0.36]
Thomas, ML, et al., 2012	64	17.60	5.30	88	16.80	4.90	followup	12.00	0.16	[-0.17; 0.48]
Kim, SH, et al., 2021	47	75.90	16.30	47	71.70	18.40	followup	20.00	0.24	[-0.17; 0.65]
Walczak, A, et al.,2017	61	17.88	4.63	49	16.61	4.80	followup	4.00	0.27	[-0.11; 0.65]
Klafke, N, et al., 2019	120	65.90	25.60	114	56.30	24.70	followup	48.00	0.38	[0.12; 0.64]
Kim, SH, et al., 2021	47	79.00	15.70	47	69.60	18.90	followup	8.00	0.54	[0.12; 0.95]
Kim, YH, et al., 2017	30	67.80	15.10	30	56.70	21.90	followup	6.00	0.58	[0.07; 1.10]
Kim, YH, et al., 2017	30	70.80	14.50	30	57.80	22.60	followup	9.00	0.68	[0.15; 1.20]
Wu, Q, et al., 2021	43	81.54	8.21	43	74.21	7.33	followup	12.00	0.93	[0.49; 1.38]
Gao Q, et al. 2020	40	-49.55	4.20	40	-53.56	3.25	followup	4.00	1.06	[0.59; 1.53]
Zhou, J, et al., 2020	59	64.81	9.32	59	55.54	7.29	followup	2.00	1.10	[0.71; 1.49]
Qin, X, et al., 2017	50	90.72	8.27	50	75.60	10.01	followup	2.00	1.63	[1.18; 2.09]
van der Meulen, IC, et al., 2013	88	84.90	3.00	91	77.20	2.80	followup	48.00	2.64	[2.24; 3.05]
Fang, P, et al.,2020	60	63.52	6.47	60	47.56	5.14	followup	4.00	2.71	[2.21; 3.21]
Zhao, X, et al., 2021	52	71.02	2.31	51	58.91	5.21	followup	12.00	2.99	[2.42; 3.56]
Random effect	2470			2198					0.30	[-0.22; 0.83]
Prediction interval										[-1.04; 1.65]
**Random effect**	**9862**			**10243**					**0.10**	**[-0.23; 0.43]**
**Prediction interval**										**[-1.24; 1.44]**

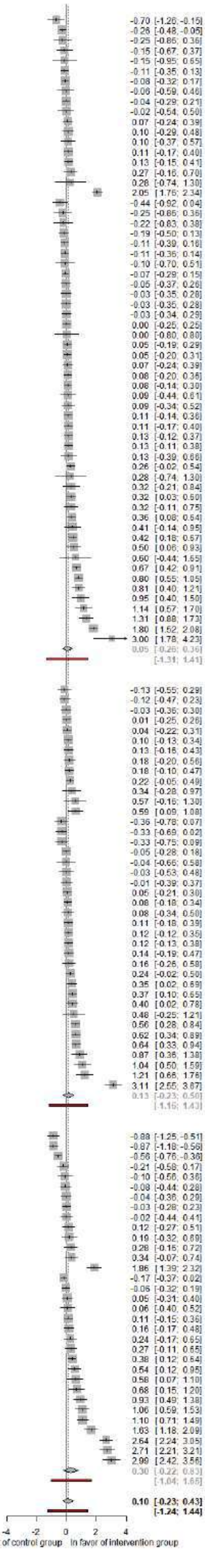

Figure S8.4. Forest plot represents the difference between the intervention vs. control group in the Emotional QoL domain with the provider subgroups as predicted at week 48 (post-intervention). SMD - Standardized mean difference, CI - confidence interval

S9.Subgroup analysis of Emotional QoL: Environment

Figure S9.1.T0

Study	Experimental			Control							
	Patient N	Mean	SD	Patient N	Mean	SD	follow-up	Follow-up time	SMD of interested event	SMD	95%-CI
face to face											
Zhao,X, et al. 2015	62	5.41	1.79	62	7.24	2.31	baseline	0.00		-0.88	[-1.25; -0.51]
van der Meulen, IC, et al., 2013	88	64.80	2.40	91	66.90	2.40	baseline	0.00		-0.87	[-1.18; -0.56]
Seliniotaki, T, et al., 2021	27	56.10	29.10	26	74.20	20.90	baseline	0.00		-0.70	[-1.26; -0.15]
McLachlan, SA, et al., 2001	296	46.00	29.20	154	61.10	22.00	baseline	0.00		-0.56	[-0.76; -0.36]
Reich, RR, et al. 2016	167	63.83	17.42	155	68.57	18.32	baseline	0.00		-0.26	[-0.48; -0.05]
Serfaty M, et al., 2018	20	12.30	5.20	22	13.60	4.90	baseline	0.00		-0.25	[-0.86; 0.36]
Nápoles AM, et al. 2015	76	12.07	4.91	75	12.86	5.14	baseline	0.00		-0.16	[-0.48; 0.16]
Rahmani, S, et al. 2015	12	11.11	8.94	12	12.50	9.05	baseline	0.00		-0.15	[-0.95; 0.65]
Berglund, G. et al. 2007	39	81.20	18.90	150	83.60	19.70	baseline	0.00		-0.12	[-0.47; 0.23]
Qiu, H, et al., 2018 II.	98	11.50	6.30	196	12.22	6.56	baseline	0.00		-0.11	[-0.35; 0.13]
Fang, P, et al.,2020	60	46.14	5.12	60	46.55	5.23	baseline	0.00		-0.08	[-0.44; 0.28]
Qiu, H, et al., 2018 I.	98	11.72	6.65	196	12.22	6.56	baseline	0.00		-0.08	[-0.32; 0.17]
Hernandez, EG, et al. 2018	28	13.07	5.26	28	13.39	4.94	baseline	0.00		-0.06	[-0.59; 0.46]
Klafke, N, et al., 2019	120	50.00	26.00	114	50.70	26.50	baseline	0.00		-0.03	[-0.28; 0.23]
Powell, CB, et al., 2008	21	16.10	4.20	43	16.20	5.00	baseline	0.00		-0.02	[-0.54; 0.50]
Chan, et al. 2005	80	58.10	45.50	75	54.60	48.48	baseline	0.00		0.07	[-0.24; 0.39]
Liu, T, et al., 2019	49	71.18	18.38	53	69.23	22.04	baseline	0.00		0.10	[-0.29; 0.48]
Braeken APB, et al. 2013	136	78.40	20.77	144	76.12	24.16	baseline	0.00		0.10	[-0.13; 0.34]
Penedo, FJ, et al., 2007	41	-19.24	4.04	30	-19.63	3.43	baseline	0.00		0.10	[-0.37; 0.57]
Qin, X, et al., 2017	50	73.61	10.68	50	72.41	9.34	baseline	0.00		0.12	[-0.27; 0.51]
Klinkhammer-Schalke, M et al., 2012	100	51.86	29.03	100	48.14	28.29	baseline	0.00		0.13	[-0.15; 0.41]
Guo, Z, et al., 2013	89	72.57	12.51	89	70.77	14.71	baseline	0.00		0.13	[-0.16; 0.43]
Schofield, P, et al., 2013	55	77.12	22.92	53	72.93	23.08	baseline	0.00		0.18	[-0.20; 0.56]
Arving, C, et al., 2007	47	69.00	25.00	38	63.00	18.00	baseline	0.00		0.27	[-0.16; 0.70]
Rodrigez, B, et al., 2014	8	88.54	22.24	7	79.76	36.28	baseline	0.00		0.28	[-0.74; 1.30]
Gao Q, et al. 2020	40	-56.85	4.66	40	-58.20	4.78	baseline	0.00		0.28	[-0.16; 0.72]
Elyasi, F, et al., 2021 II.	20	10.60	3.10	20	9.60	2.60	baseline	0.00		0.34	[-0.28; 0.97]
Elyasi, F, et al., 2021 I.	15	11.50	3.80	15	9.60	2.60	baseline	0.00		0.57	[-0.16; 1.30]
Zhao, X, et al., 2021	52	64.03	4.42	51	58.12	0.42	baseline	0.00		1.86	[1.39; 2.32]
Lu, Z, et al., 201	203	79.70	3.60	103	71.30	4.90	baseline	0.00		2.05	[1.76; 2.34]
Penedo, FJ, et al., 2007	41	-20.73	3.31	30	-19.20	3.62	followup	12.00		-0.44	[-0.92; 0.04]
Berglund, G. et al. 2007	39	81.60	20.10	150	87.80	18.00	followup	48.00		-0.33	[-0.69; 0.02]
Serfaty M, et al., 2018	20	15.40	4.50	22	16.60	4.90	followup	12.00		-0.25	[-0.86; 0.36]
Serfaty M, et al., 2018	20	15.60	4.20	22	16.50	3.70	followup	24.00		-0.22	[-0.83; 0.38]
Chan, et al. 2005	80	65.70	47.01	75	73.94	39.30	followup	12.00		-0.19	[-0.50; 0.13]
McLachlan, SA, et al., 2001	296	45.80	30.20	154	50.70	25.00	followup	24.00		-0.17	[-0.37; 0.02]
Klinkhammer-Schalke, M et al., 2012	100	55.34	28.29	100	58.56	28.29	followup	12.00		-0.11	[-0.39; 0.16]
Serfaty M, et al., 2018	20	16.60	5.50	22	17.10	4.40	followup	18.00		-0.10	[-0.70; 0.51]
Reich, RR, et al. 2016	167	71.33	19.41	155	72.71	19.13	followup	12.00		-0.07	[-0.29; 0.15]
Klafke, N, et al., 2019	120	62.80	22.30	114	64.20	22.40	followup	12.00		-0.06	[-0.32; 0.19]
Chan, et al. 2005	80	75.77	73.38	75	79.44	64.08	followup	72.00		-0.05	[-0.37; 0.26]
Braeken APB, et al. 2013	136	78.38	22.75	144	79.46	20.68	followup	12.00		-0.05	[-0.28; 0.18]
Elyasi, F, et al., 2021 II.	20	9.40	2.10	20	9.50	2.60	followup	24.00		-0.04	[-0.66; 0.58]
Chan, et al. 2005	80	76.73	57.02	75	78.59	56.21	followup	48.00		-0.03	[-0.35; 0.28]
Chan, et al. 2005	80	73.39	67.76	75	75.30	55.80	followup	60.00		-0.03	[-0.35; 0.28]
Chan, et al. 2005	80	68.59	48.12	75	69.81	48.88	followup	24.00		-0.03	[-0.34; 0.29]
Schofield, P, et al., 2013	55	75.31	22.19	53	75.51	22.22	followup	12.00		-0.01	[-0.39; 0.37]
Rahmani, S, et al. 2015	12	12.00	48.61	12	12.00	23.61	followup	16.00		0.00	[-0.80; 0.80]
Zhao,X, et al. 2015	62	7.91	2.24	62	7.81	1.98	followup	0.30		0.05	[-0.31; 0.40]
Qiu, H, et al., 2018 II.	98	12.32	9.06	196	11.90	7.89	followup	4.00		0.05	[-0.19; 0.29]
Chan, et al. 2005	80	77.37	58.41	75	73.44	52.28	followup	36.00		0.07	[-0.24; 0.39]
Klinkhammer-Schalke, M et al., 2012	100	63.52	27.07	100	61.29	27.29	followup	48.00		0.08	[-0.20; 0.36]
Reich, RR, et al. 2016	167	70.87	18.02	155	69.36	18.78	followup	6.00		0.08	[-0.14; 0.30]
Powell, CB, et al., 2008	21	18.50	5.40	43	18.00	5.90	followup	12.00		0.09	[-0.44; 0.61]
Arving, C, et al., 2007	47	76.00	24.00	38	74.00	20.00	followup	12.00		0.09	[-0.34; 0.52]
Klafke, N, et al., 2019	120	59.10	25.40	114	56.50	23.00	followup	24.00		0.11	[-0.15; 0.36]
Braeken APB, et al. 2013	136	83.66	20.80	144	81.23	20.60	followup	48.00		0.12	[-0.12; 0.35]
Qiu, H, et al., 2018 II.	98	14.58	9.06	196	13.52	7.89	followup	24.00		0.13	[-0.12; 0.37]
Qiu, H, et al., 2018 II.	98	14.23	7.67	196	13.10	8.87	followup	12.00		0.13	[-0.11; 0.38]
Hernandez, EG, et al. 2018	28	14.00	4.69	28	13.32	5.32	followup	24.00		0.13	[-0.39; 0.66]
Klinkhammer-Schalke, M et al., 2012	100	66.50	26.80	100	59.06	29.53	followup	36.00		0.26	[-0.02; 0.54]
Rodrigez, B, et al., 2014	8	88.54	22.24	7	79.76	36.28	followup	0.10		0.28	[-0.74; 1.30]
Nápoles AM, et al. 2015	76	15.93	3.52	75	14.73	4.33	followup	12.00		0.30	[-0.02; 0.62]
Hernandez, EG, et al. 2018	28	16.12	4.09	28	14.72	4.63	followup	8.00		0.32	[-0.21; 0.84]
Arving, C, et al., 2007	47	82.00	20.00	38	75.00	24.00	followup	24.00		0.32	[-0.11; 0.75]
Klinkhammer-Schalke, M et al., 2012	100	66.25	25.31	100	56.58	28.54	followup	24.00		0.36	[0.08; 0.64]
Klafke, N, et al., 2019	120	65.90	25.60	114	56.30	24.70	followup	48.00		0.38	[0.12; 0.64]
Schofield, P, et al., 2013	55	81.43	20.19	53	73.23	20.40	followup	8.00		0.40	[0.02; 0.78]
Seliniotaki, T, et al., 2021	27	74.50	24.50	26	61.70	36.70	followup	8.00		0.41	[-0.14; 0.95]
Nápoles AM, et al. 2015	76	16.39	3.30	75	14.89	3.95	followup	24.00		0.41	[0.09; 0.73]
Qiu, H, et al., 2018 I.	98	15.42	9.06	196	11.90	7.89	followup	4.00		0.42	[0.18; 0.67]
Elyasi, F, et al., 2021 I.	15	11.00	3.40	15	9.50	2.60	followup	24.00		0.48	[-0.25; 1.21]
Arving, C, et al., 2007	47	80.00	21.00	38	69.00	23.00	followup	4.00		0.50	[0.06; 0.93]
Rodrigez, B, et al., 2014	8	93.33	10.87	7	80.56	26.79	followup	2.00		0.60	[-0.44; 1.65]
Guo, Z, et al., 2013	89	74.18	11.43	89	66.03	14.00	followup	2.00		0.64	[0.33; 0.94]
Qiu, H, et al., 2018 I.	98	19.23	9.76	196	13.10	8.87	followup	12.00		0.67	[0.42; 0.91]
Qiu, H, et al., 2018 I.	98	20.00	8.37	196	13.52	7.89	followup	24.00		0.80	[0.55; 1.05]
Liu, T, et al., 2019	49	63.49	13.78	53	51.93	14.62	followup	9.00		0.81	[0.40; 1.21]
Gao Q, et al. 2020	40	-49.55	4.20	40	-53.56	3.25	followup	4.00		1.06	[0.59; 1.53]
Zhou, J, et al., 2020	59	64.81	9.32	59	55.54	7.29	followup	2.00		1.10	[0.71; 1.49]
Liu, T, et al., 2019	49	74.12	9.50	53	56.57	16.10	followup	12.00		1.31	[0.88; 1.73]
Qin, X, et al., 2017	50	90.72	8.27	50	75.60	10.01	followup	2.00		1.63	[1.18; 2.09]
Lu, Z, et al., 201	203	85.40	3.50	103	77.60	5.60	followup	9.00		1.80	[1.52; 2.08]
van der Meulen, IC, et al., 2013	88	84.90	3.00	91	77.20	2.80	followup	48.00		2.64	[2.24; 3.05]
Fang, P, et al.,2020	60	63.52	6.47	60	47.56	5.14	followup	4.00		2.71	[2.21; 3.21]
Zhao, X, et al., 2021	52	71.02	2.31	51	58.91	5.21	followup	12.00		2.99	[2.42; 3.56]
Rahmani, S, et al. 2015	12	56.94	11.14	12	22.91	10.73	followup	8.00		3.00	[1.78; 4.23]
Henderson, VP, et al., 2013	53	18.00	0.40	58	16.90	0.30	followup	16.00		3.11	[2.55; 3.67]
Random effect	6503			6955						0.77	[0.35; 1.19]
Prediction interval											[-0.65; 2.19]
telephone											
Dirksen, S. et al, 2007	34	20.10	2.80	38	20.40	3.10	baseline	0.00		-0.10	[-0.56; 0.36]
Thomas, ML, et al., 2012	64	16.50	5.60	88	16.70	5.30	baseline	0.00		-0.04	[-0.36; 0.29]
Girgis, A, et al., 2009 II.	120	79.90	20.30	117	79.70	22.70	baseline	0.00		0.01	[-0.25; 0.26]
Yun et al., 2017	134	76.80	19.40	72	73.00	23.00	baseline	0.00		0.18	[-0.10; 0.47]
Kim, SH, et al., 2021	47	72.00	17.70	47	65.40	21.00	baseline	0.00		0.34	[-0.07; 0.74]
Girgis, A, et al., 2009 II.	120	85.70	16.40	117	84.90	18.50	followup	12.00		0.05	[-0.21; 0.30]
Dirksen, S. et al, 2007	34	20.80	2.30	38	20.60	4.00	followup	10.00		0.06	[-0.40; 0.52]
Yun et al., 2017	134	78.00	19.90	72	75.90	18.30	followup	48.00		0.11	[-0.18; 0.39]
Girgis, A, et al., 2009 II.	120	86.70	18.70	117	84.40	18.90	followup	24.00		0.12	[-0.13; 0.38]
Thomas, ML, et al., 2012	64	17.60	5.30	88	16.80	4.90	followup	12.00		0.16	[-0.17; 0.48]
Kim, SH, et al., 2021	47	75.90	16.30	47	71.70	18.40	followup	20.00		0.24	[-0.17; 0.65]
Kim, SH, et al., 2021	47	79.00	15.70	47	69.60	18.90	followup	8.00		0.54	[0.12; 0.95]
McCusker, J, et al., 2021	99	43.30	9.20	119	37.40	9.80	followup	24.00		0.62	[0.34; 0.89]
Random effect	1064			1007						0.65	[0.24; 1.06]
Prediction interval											[-0.82; 2.11]
online											
Peng, L, et al., 2022	28	69.94	16.25	29	72.41	16.08	baseline	0.00		-0.15	[-0.67; 0.37]
Wu, Q, et al., 2021	43	62.34	6.76	43	62.45	6.63	baseline	0.00		-0.02	[-0.44; 0.41]
Penedo, FJ, et al., 2020	95	-20.03	3.80	97	-20.46	3.74	baseline	0.00		0.11	[-0.17; 0.40]
Cengiz, HO, et al., 2023	32	12.90	5.40	33	9.75	5.22	baseline	0.00		0.59	[0.09; 1.08]
Beatty, L. et al. 2015	30	69.03	4.81	30	69.16	4.79	followup	6.00		-0.03	[-0.53; 0.48]
Penedo, FJ, et al., 2020	95	-20.18	3.80	97	-20.62	3.84	followup	24.00		0.11	[-0.17; 0.40]
Penedo, FJ, et al., 2020	95	-19.20	3.90	97	-20.43	3.84	followup	48.00		0.32	[0.03; 0.60]
Cengiz, HO, et al., 2023	32	13.56	5.78	33	8.75	5.14	followup	8.00		0.87	[0.36; 1.38]
Wu, Q, et al., 2021	43	81.54	8.21	43	74.21	7.33	followup	12.00		0.93	[0.49; 1.38]
Peng, L, et al., 2022	28	80.95	13.20	29	70.12	9.05	followup	4.00		0.95	[0.40; 1.50]
Beatty, L. et al. 2015	30	72.59	4.87	30	67.43	4.89	followup	13.03		1.04	[0.50; 1.59]
Peng, L, et al., 2022	28	83.33	14.87	29	69.25	8.94	followup	0.10		1.14	[0.57; 1.70]
Beatty, L. et al. 2015	30	76.54	4.22	30	71.38	4.21	followup	26.07		1.21	[0.66; 1.76]
Random effect	609			620						0.94	[0.57; 1.30]
Prediction interval											[-0.60; 2.48]
**Random effect**	**8176**			**8582**						**0.79**	**[0.38; 1.19]**
**Prediction interval**											**[-0.63; 2.20]**

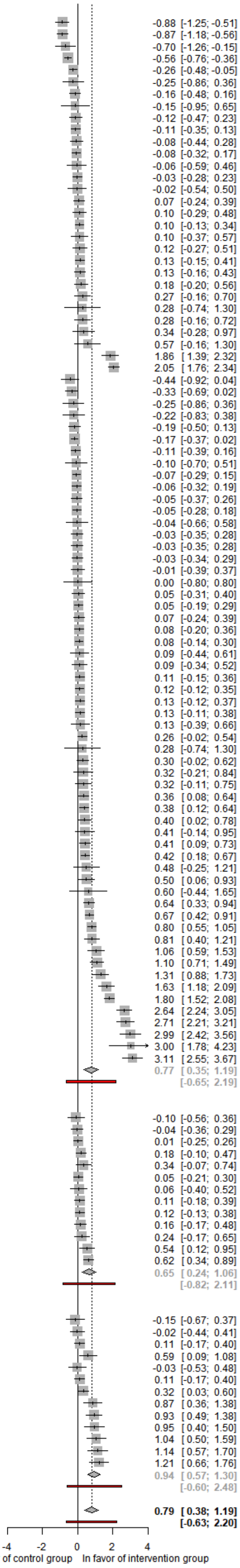

Figure S9.1. Forest plot represents the difference between the intervention vs. control group in the Emotional QoL domain with the environment subgroups as predicted at week 0 (post-intervention). SMD - Standardized mean difference, CI - confidence interval.

Figure S9.2.T12

Study	Experimental Patient N	Experimental Mean	Experimental SD	Control Patient N	Control Mean	Control SD	follow-up	Follow-up time	SMD	95%-CI
face to face										
Zhao,X, et al. 2015	62	5.41	1.79	62	7.24	2.31	baseline	0.00	-0.88	[-1.25; -0.51]
van der Meulen, IC, et al., 2013	88	64.80	2.40	91	66.90	2.40	baseline	0.00	-0.87	[-1.18; -0.56]
Seliniotaki, T, et al., 2021	27	56.10	29.10	26	74.20	20.90	baseline	0.00	-0.70	[-1.26; -0.15]
McLachlan, SA, et al., 2001	296	46.00	29.20	154	61.10	22.00	baseline	0.00	-0.56	[-0.76; -0.36]
Reich, RR, et al. 2016	167	63.83	17.42	155	68.57	18.32	baseline	0.00	-0.26	[-0.48; -0.05]
Serfaty M, et al., 2018	20	12.30	5.20	22	13.60	4.90	baseline	0.00	-0.25	[-0.86; 0.36]
Nápoles AM, et al. 2015	76	12.07	4.91	75	12.86	5.14	baseline	0.00	-0.16	[-0.48; 0.16]
Rahmani, S, et al. 2015	12	11.11	8.94	12	12.50	9.05	baseline	0.00	-0.15	[-0.95; 0.65]
Berglund, G. et al. 2007	39	81.20	18.90	150	83.60	19.70	baseline	0.00	-0.12	[-0.47; 0.23]
Qiu, H, et al., 2018 II.	98	11.50	6.30	196	12.22	6.56	baseline	0.00	-0.11	[-0.35; 0.13]
Fang, P, et al.,2020	60	46.14	5.12	60	46.55	5.23	baseline	0.00	-0.08	[-0.44; 0.28]
Qiu, H, et al., 2018 I.	98	11.72	6.65	196	12.22	6.56	baseline	0.00	-0.08	[-0.32; 0.17]
Hernandez, EG, et al. 2018	28	13.07	5.26	28	13.39	4.94	baseline	0.00	-0.06	[-0.59; 0.46]
Klafke, N, et al., 2019	120	50.00	26.00	114	50.70	26.50	baseline	0.00	-0.03	[-0.28; 0.23]
Powell, CB, et al., 2008	21	16.10	4.20	43	16.20	5.00	baseline	0.00	-0.02	[-0.54; 0.50]
Chan, et al. 2005	80	58.10	45.50	75	54.60	48.48	baseline	0.00	0.07	[-0.24; 0.39]
Liu, T, et al., 2019	49	71.18	18.38	53	69.23	22.04	baseline	0.00	0.10	[-0.29; 0.48]
Braeken APB, et al. 2013	136	78.40	20.77	144	76.12	24.16	baseline	0.00	0.10	[-0.13; 0.34]
Penedo, FJ, et al., 2007	41	-19.24	4.04	30	-19.63	3.43	baseline	0.00	0.10	[-0.37; 0.57]
Qin, X, et al., 2017	50	73.61	10.68	50	72.41	9.34	baseline	0.00	0.12	[-0.27; 0.51]
Klinkhammer-Schalke, M et al., 2012	100	51.86	29.03	100	48.14	28.29	baseline	0.00	0.13	[-0.15; 0.41]
Guo, Z, et al., 2013	89	72.57	12.51	89	70.77	14.71	baseline	0.00	0.13	[-0.16; 0.43]
Schofield, P, et al., 2013	55	77.12	22.92	53	72.93	23.08	baseline	0.00	0.18	[-0.20; 0.56]
Arving, C, et al., 2007	47	69.00	25.00	38	63.00	18.00	baseline	0.00	0.27	[-0.16; 0.70]
Rodrigez, B, et al., 2014	8	88.54	22.24	7	79.76	36.28	baseline	0.00	0.28	[-0.74; 1.30]
Gao Q, et al. 2020	40	-56.85	4.66	40	-58.20	4.78	baseline	0.00	0.28	[-0.16; 0.72]
Elyasi, F, et al., 2021 II.	20	10.60	3.10	20	9.60	2.60	baseline	0.00	0.34	[-0.28; 0.97]
Elyasi, F, et al., 2021 I.	15	11.50	3.80	15	9.60	2.60	baseline	0.00	0.57	[-0.16; 1.30]
Zhao, X, et al., 2021	52	64.03	4.42	51	58.12	0.42	baseline	0.00	1.86	[1.39; 2.32]
Lu, Z, et al., 201	203	79.70	3.60	103	71.30	4.90	baseline	0.00	2.05	[1.76; 2.34]
Penedo, FJ, et al., 2007	41	-20.73	3.31	30	-19.20	3.62	followup	12.00	-0.44	[-0.92; 0.04]
Berglund, G. et al. 2007	39	81.60	20.10	150	87.80	18.00	followup	48.00	-0.33	[-0.69; 0.02]
Serfaty M, et al., 2018	20	15.40	4.50	22	16.60	4.90	followup	12.00	-0.25	[-0.86; 0.36]
Serfaty M, et al., 2018	20	15.60	4.20	22	16.50	3.70	followup	24.00	-0.22	[-0.83; 0.38]
Chan, et al. 2005	80	65.70	47.01	75	73.94	39.30	followup	12.00	-0.19	[-0.50; 0.13]
McLachlan, SA, et al., 2001	296	45.80	30.20	154	50.70	25.00	followup	24.00	-0.17	[-0.37; 0.02]
Klinkhammer-Schalke, M et al., 2012	100	55.34	28.29	100	58.56	28.29	followup	12.00	-0.11	[-0.39; 0.16]
Serfaty M, et al., 2018	20	16.60	5.50	22	17.10	4.40	followup	18.00	-0.10	[-0.70; 0.51]
Reich, RR, et al. 2016	167	71.33	19.41	155	72.71	19.13	followup	12.00	-0.07	[-0.29; 0.15]
Klafke, N, et al., 2019	120	62.80	22.30	114	64.20	22.40	followup	12.00	-0.06	[-0.32; 0.19]
Chan, et al. 2005	80	75.77	73.38	75	79.44	64.08	followup	72.00	-0.05	[-0.37; 0.26]
Braeken APB, et al. 2013	136	78.38	22.75	144	79.46	20.68	followup	12.00	-0.05	[-0.28; 0.18]
Elyasi, F, et al., 2021 II.	20	9.40	2.10	20	9.50	2.60	followup	24.00	-0.04	[-0.66; 0.58]
Chan, et al. 2005	80	76.73	57.02	75	78.59	56.21	followup	48.00	-0.03	[-0.35; 0.28]
Chan, et al. 2005	80	73.39	67.76	75	75.30	55.80	followup	60.00	-0.03	[-0.35; 0.28]
Chan, et al. 2005	80	68.59	48.12	75	69.81	48.88	followup	24.00	-0.03	[-0.34; 0.29]
Schofield, P, et al., 2013	55	75.31	22.19	53	75.51	22.22	followup	12.00	-0.01	[-0.39; 0.37]
Rahmani, S, et al. 2015	12	12.00	48.61	12	12.00	23.61	followup	16.00	0.00	[-0.80; 0.80]
Zhao,X, et al. 2015	62	7.91	2.24	62	7.81	1.98	followup	0.30	0.05	[-0.31; 0.40]
Qiu, H, et al., 2018 II.	98	12.32	9.06	196	11.90	7.89	followup	4.00	0.05	[-0.19; 0.29]
Chan, et al. 2005	80	77.37	58.41	75	73.44	52.28	followup	36.00	0.07	[-0.24; 0.39]
Klinkhammer-Schalke, M et al., 2012	100	63.52	27.07	100	61.29	27.29	followup	48.00	0.08	[-0.20; 0.36]
Reich, RR, et al. 2016	167	70.87	18.02	155	69.36	18.78	followup	6.00	0.08	[-0.14; 0.30]
Powell, CB, et al., 2008	21	18.50	5.40	43	18.00	5.90	followup	12.00	0.09	[-0.44; 0.61]
Arving, C, et al., 2007	47	76.00	24.00	38	74.00	20.00	followup	12.00	0.09	[-0.34; 0.52]
Klafke, N, et al., 2019	120	59.10	25.40	114	56.50	23.00	followup	24.00	0.11	[-0.15; 0.36]
Braeken APB, et al. 2013	136	83.66	20.80	144	81.23	20.60	followup	48.00	0.12	[-0.12; 0.35]
Qiu, H, et al., 2018 II.	98	14.58	9.06	196	13.52	7.89	followup	24.00	0.13	[-0.12; 0.37]
Qiu, H, et al., 2018 II.	98	14.23	7.67	196	13.10	8.87	followup	12.00	0.13	[-0.11; 0.38]
Hernandez, EG, et al. 2018	28	14.00	4.69	28	13.32	5.32	followup	24.00	0.13	[-0.39; 0.66]
Klinkhammer-Schalke, M et al., 2012	100	66.50	26.80	100	59.06	29.53	followup	36.00	0.26	[-0.02; 0.54]
Rodrigez, B, et al., 2014	8	88.54	22.24	7	79.76	36.28	followup	0.10	0.28	[-0.74; 1.30]
Nápoles AM, et al. 2015	76	15.93	3.52	75	14.73	4.33	followup	12.00	0.30	[-0.02; 0.62]
Hernandez, EG, et al. 2018	28	16.12	4.09	28	14.72	4.63	followup	8.00	0.32	[-0.21; 0.84]
Arving, C, et al., 2007	47	82.00	20.00	38	75.00	24.00	followup	24.00	0.32	[-0.11; 0.75]
Klinkhammer-Schalke, M et al., 2012	100	66.25	25.31	100	56.58	28.54	followup	24.00	0.36	[0.08; 0.64]
Klafke, N, et al., 2019	120	65.90	25.60	114	56.30	24.70	followup	48.00	0.38	[0.12; 0.64]
Schofield, P, et al., 2013	55	81.43	20.19	53	73.23	20.40	followup	8.00	0.40	[0.02; 0.78]
Seliniotaki, T, et al., 2021	27	74.50	24.50	26	61.70	36.70	followup	8.00	0.41	[-0.14; 0.95]
Nápoles AM, et al. 2015	76	16.39	3.30	75	14.89	3.95	followup	24.00	0.41	[0.09; 0.73]
Qiu, H, et al., 2018 I.	98	15.42	9.06	196	11.90	7.89	followup	4.00	0.42	[0.18; 0.67]
Elyasi, F, et al., 2021 I.	15	11.00	3.40	15	9.50	2.60	followup	24.00	0.48	[-0.25; 1.21]
Arving, C, et al., 2007	47	80.00	21.00	38	69.00	23.00	followup	4.00	0.50	[0.06; 0.93]
Rodrigez, B, et al., 2014	8	93.33	10.87	7	80.56	26.79	followup	2.00	0.60	[-0.44; 1.65]
Guo, Z, et al., 2013	89	74.18	11.43	89	66.03	14.00	followup	2.00	0.64	[0.33; 0.94]
Qiu, H, et al., 2018 I.	98	19.23	9.76	196	13.10	8.87	followup	12.00	0.67	[0.42; 0.91]
Qiu, H, et al., 2018 I.	98	20.00	8.37	196	13.52	7.89	followup	24.00	0.80	[0.55; 1.05]
Liu, T, et al., 2019	49	63.49	13.78	53	51.93	14.62	followup	9.00	0.81	[0.40; 1.21]
Gao Q, et al. 2020	40	-49.55	4.20	40	-53.56	3.25	followup	4.00	1.06	[0.59; 1.53]
Zhou, J, et al., 2020	59	64.81	9.32	59	55.54	7.29	followup	2.00	1.10	[0.71; 1.49]
Liu, T, et al., 2019	49	74.12	9.50	53	56.57	16.10	followup	12.00	1.31	[0.88; 1.73]
Qin, X, et al., 2017	50	90.72	8.27	50	75.60	10.01	followup	2.00	1.63	[1.18; 2.09]
Lu, Z, et al., 201	203	85.40	3.50	103	77.60	5.60	followup	9.00	1.80	[1.52; 2.08]
van der Meulen, IC, et al., 2013	88	84.90	3.00	91	77.20	2.80	followup	48.00	2.64	[2.24; 3.05]
Fang, P, et al.,2020	60	63.52	6.47	60	47.56	5.14	followup	4.00	2.71	[2.21; 3.21]
Zhao, X, et al., 2021	52	71.02	2.31	51	58.91	5.21	followup	12.00	2.99	[2.42; 3.56]
Rahmani, S, et al. 2015	12	56.94	11.14	12	22.91	10.73	followup	8.00	3.00	[1.78; 4.23]
Henderson, VP, et al., 2013	53	18.00	0.40	58	16.90	0.30	followup	16.00	3.11	[2.55; 3.67]
Random effect	6503			6955					0.52	[0.28; 0.76]
Prediction interval										[-0.82; 1.86]
telephone										
Dirksen, S. et al, 2007	34	20.10	2.80	38	20.40	3.10	baseline	0.00	-0.10	[-0.56; 0.36]
Thomas, ML, et al., 2012	64	16.50	5.60	88	16.70	5.30	baseline	0.00	-0.04	[-0.36; 0.29]
Girgis, A, et al., 2009 II.	120	79.90	20.30	117	79.70	22.70	baseline	0.00	0.01	[-0.25; 0.26]
Yun et al., 2017	134	76.80	19.40	72	73.00	23.00	baseline	0.00	0.18	[-0.10; 0.47]
Kim, SH, et al., 2021	47	72.00	17.70	47	65.40	21.00	baseline	0.00	0.34	[-0.07; 0.74]
Girgis, A, et al., 2009 II.	120	85.70	16.40	117	84.90	18.50	followup	12.00	0.05	[-0.21; 0.30]
Dirksen, S. et al, 2007	34	20.80	2.30	38	20.60	4.00	followup	10.00	0.06	[-0.40; 0.52]
Yun et al., 2017	134	78.00	19.90	72	75.90	18.30	followup	48.00	0.11	[-0.18; 0.39]
Girgis, A, et al., 2009 II.	120	86.70	18.70	117	84.40	18.90	followup	24.00	0.12	[-0.13; 0.38]
Thomas, ML, et al., 2012	64	17.60	5.30	88	16.80	4.90	followup	12.00	0.16	[-0.17; 0.48]
Kim, SH, et al., 2021	47	75.90	16.30	47	71.70	18.40	followup	20.00	0.24	[-0.17; 0.65]
Kim, SH, et al., 2021	47	79.00	15.70	47	69.60	18.90	followup	8.00	0.54	[0.12; 0.95]
McCusker, J, et al., 2021	99	43.30	9.20	119	37.40	9.80	followup	24.00	0.62	[0.34; 0.89]
Random effect	1064			1007					0.40	[0.16; 0.63]
Prediction interval										[-1.24; 2.04]
online										
Peng, L, et al., 2022	28	69.94	16.25	29	72.41	16.08	baseline	0.00	-0.15	[-0.67; 0.37]
Wu, Q, et al., 2021	43	62.34	6.76	43	62.45	6.63	baseline	0.00	-0.02	[-0.44; 0.41]
Penedo, FJ, et al., 2020	95	-20.03	3.80	97	-20.46	3.74	baseline	0.00	0.11	[-0.17; 0.40]
Cengiz, HO, et al., 2023	32	12.90	5.40	33	9.75	5.22	baseline	0.00	0.59	[0.09; 1.08]
Beatty, L. et al. 2015	30	69.03	4.81	30	69.16	4.79	followup	6.00	-0.03	[-0.53; 0.48]
Penedo, FJ, et al., 2020	95	-20.18	3.80	97	-20.62	3.84	followup	24.00	0.11	[-0.17; 0.40]
Penedo, FJ, et al., 2020	95	-19.20	3.90	97	-20.43	3.84	followup	48.00	0.32	[0.03; 0.60]
Cengiz, HO, et al., 2023	32	13.56	5.78	33	8.75	5.14	followup	8.00	0.87	[0.36; 1.38]
Wu, Q, et al., 2021	43	81.54	8.21	43	74.21	7.33	followup	12.00	0.93	[0.49; 1.38]
Peng, L, et al., 2022	28	80.95	13.20	29	70.12	9.05	followup	4.00	0.95	[0.40; 1.50]
Beatty, L. et al. 2015	30	72.59	4.87	30	67.43	4.89	followup	13.03	1.04	[0.50; 1.59]
Peng, L, et al., 2022	28	83.33	14.87	29	69.25	8.94	followup	0.10	1.14	[0.57; 1.70]
Beatty, L. et al. 2015	30	76.54	4.22	30	71.38	4.21	followup	26.07	1.21	[0.66; 1.76]
Random effect	609			620					0.69	[0.45; 0.92]
Prediction interval										[-1.08; 2.45]
**Random effect**	**8176**			**8582**					**0.53**	**[0.33; 0.72]**
**Prediction interval**										**[-0.80; 1.85]**

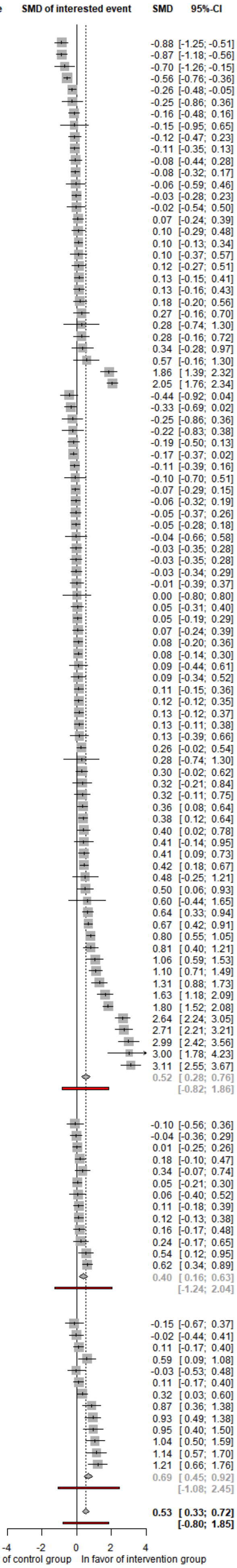

Figure S9.2. Forest plot represents the difference between the intervention vs. control group in the Emotional QoL domain with the environment subgroups as predicted at week 12 (post-intervention). SMD - Standardized mean difference, CI - confidence interval.

Figure S9.3.T24

Study	Experimental Patient N	Experimental Mean	Experimental SD	Control Patient N	Control Mean	Control SD	follow-up	Follow-up time	SMD	95%-CI
face to face										
Zhao,X, et al. 2015	62	5.41	1.79	62	7.24	2.31	baseline	0.00	-0.88	[-1.25; -0.51]
van der Meulen, IC, et al., 2013	88	64.80	2.40	91	66.90	2.40	baseline	0.00	-0.87	[-1.18; -0.56]
Seliniotaki, T, et al., 2021	27	56.10	29.10	26	74.20	20.90	baseline	0.00	-0.70	[-1.26; -0.15]
McLachlan, SA, et al., 2001	296	46.00	29.20	154	61.10	22.00	baseline	0.00	-0.56	[-0.76; -0.36]
Reich, RR, et al. 2016	167	63.83	17.42	155	68.57	18.32	baseline	0.00	-0.26	[-0.48; -0.05]
Serfaty M, et al., 2018	20	12.30	5.20	22	13.60	4.90	baseline	0.00	-0.25	[-0.86; 0.36]
Nápoles AM, et al. 2015	76	12.07	4.91	75	12.86	5.14	baseline	0.00	-0.16	[-0.48; 0.16]
Rahmani, S, et al. 2015	12	11.11	8.94	12	12.50	9.05	baseline	0.00	-0.15	[-0.95; 0.65]
Berglund, G. et al. 2007	39	81.20	18.90	150	83.60	19.70	baseline	0.00	-0.12	[-0.47; 0.23]
Qiu, H, et al., 2018 II.	98	11.50	6.30	196	12.22	6.56	baseline	0.00	-0.11	[-0.35; 0.13]
Fang, P, et al.,2020	60	46.14	5.12	60	46.55	5.23	baseline	0.00	-0.08	[-0.44; 0.28]
Qiu, H, et al., 2018 I.	98	11.72	6.65	196	12.22	6.56	baseline	0.00	-0.08	[-0.32; 0.17]
Hernandez, EG, et al. 2018	28	13.07	5.26	28	13.39	4.94	baseline	0.00	-0.06	[-0.59; 0.46]
Klafke, N, et al., 2019	120	50.00	26.00	114	50.70	26.50	baseline	0.00	-0.03	[-0.28; 0.23]
Powell, CB, et al., 2008	21	16.10	4.20	43	16.20	5.00	baseline	0.00	-0.02	[-0.54; 0.50]
Chan, et al. 2005	80	58.10	45.50	75	54.60	48.48	baseline	0.00	0.07	[-0.24; 0.39]
Liu, T, et al., 2019	49	71.18	18.38	53	69.23	22.04	baseline	0.00	0.10	[-0.29; 0.48]
Braeken APB, et al. 2013	136	78.40	20.77	144	76.12	24.16	baseline	0.00	0.10	[-0.13; 0.34]
Penedo, FJ, et al., 2007	41	-19.24	4.04	30	-19.63	3.43	baseline	0.00	0.10	[-0.37; 0.57]
Qin, X, et al., 2017	50	73.61	10.68	50	72.41	9.34	baseline	0.00	0.12	[-0.27; 0.51]
Klinkhammer-Schalke, M et al., 2012	100	51.86	29.03	100	48.14	28.29	baseline	0.00	0.13	[-0.15; 0.41]
Guo, Z, et al., 2013	89	72.57	12.51	89	70.77	14.71	baseline	0.00	0.13	[-0.16; 0.43]
Schofield, P, et al., 2013	55	77.12	22.92	53	72.93	23.08	baseline	0.00	0.18	[-0.20; 0.56]
Arving, C, et al., 2007	47	69.00	25.00	38	63.00	18.00	baseline	0.00	0.27	[-0.16; 0.70]
Rodrigez, B, et al., 2014	8	88.54	22.24	7	79.76	36.28	baseline	0.00	0.28	[-0.74; 1.30]
Gao Q, et al. 2020	40	-56.85	4.66	40	-58.20	4.78	baseline	0.00	0.28	[-0.16; 0.72]
Elyasi, F, et al., 2021 II.	20	10.60	3.10	20	9.60	2.60	baseline	0.00	0.34	[-0.28; 0.97]
Elyasi, F, et al., 2021 I.	15	11.50	3.80	15	9.60	2.60	baseline	0.00	0.57	[-0.16; 1.30]
Zhao, X, et al., 2021	52	64.03	4.42	51	58.12	0.42	baseline	0.00	1.86	[1.39; 2.32]
Lu, Z, et al., 201	203	79.70	3.60	103	71.30	4.90	baseline	0.00	2.05	[1.76; 2.34]
Penedo, FJ, et al., 2007	41	-20.73	3.31	30	-19.20	3.62	followup	12.00	-0.44	[-0.92; 0.04]
Berglund, G. et al. 2007	39	81.60	20.10	150	87.80	18.00	followup	48.00	-0.33	[-0.69; 0.02]
Serfaty M, et al., 2018	20	15.40	4.50	22	16.60	4.90	followup	12.00	-0.25	[-0.86; 0.36]
Serfaty M, et al., 2018	20	15.60	4.20	22	16.50	3.70	followup	24.00	-0.22	[-0.83; 0.38]
Chan, et al. 2005	80	65.70	47.01	75	73.94	39.30	followup	12.00	-0.19	[-0.50; 0.13]
McLachlan, SA, et al., 2001	296	45.80	30.20	154	50.70	25.00	followup	24.00	-0.17	[-0.37; 0.02]
Klinkhammer-Schalke, M et al., 2012	100	55.34	28.29	100	58.56	28.29	followup	12.00	-0.11	[-0.39; 0.16]
Serfaty M, et al., 2018	20	16.60	5.50	22	17.10	4.40	followup	18.00	-0.10	[-0.70; 0.51]
Reich, RR, et al. 2016	167	71.33	19.41	155	72.71	19.13	followup	12.00	-0.07	[-0.29; 0.15]
Klafke, N, et al., 2019	120	62.80	22.30	114	64.20	22.40	followup	12.00	-0.06	[-0.32; 0.19]
Chan, et al. 2005	80	75.77	73.38	75	79.44	64.08	followup	72.00	-0.05	[-0.37; 0.26]
Braeken APB, et al. 2013	136	78.38	22.75	144	79.46	20.68	followup	12.00	-0.05	[-0.28; 0.18]
Elyasi, F, et al., 2021 II.	20	9.40	2.10	20	9.50	2.60	followup	24.00	-0.04	[-0.66; 0.58]
Chan, et al. 2005	80	76.73	57.02	75	78.59	56.21	followup	48.00	-0.03	[-0.35; 0.28]
Chan, et al. 2005	80	73.39	67.76	75	75.30	55.80	followup	60.00	-0.03	[-0.35; 0.28]
Chan, et al. 2005	80	68.59	48.12	75	69.81	48.88	followup	24.00	-0.03	[-0.34; 0.29]
Schofield, P, et al., 2013	55	75.31	22.19	53	75.51	22.22	followup	12.00	-0.01	[-0.39; 0.37]
Rahmani, S, et al. 2015	12	12.00	48.61	12	12.00	23.61	followup	16.00	0.00	[-0.80; 0.80]
Zhao,X, et al. 2015	62	7.91	2.24	62	7.81	1.98	followup	0.30	0.05	[-0.31; 0.40]
Qiu, H, et al., 2018 II.	98	12.32	9.06	196	11.90	7.89	followup	4.00	0.05	[-0.19; 0.29]
Chan, et al. 2005	80	77.37	58.41	75	73.44	52.28	followup	36.00	0.07	[-0.24; 0.39]
Klinkhammer-Schalke, M et al., 2012	100	63.52	27.07	100	61.29	27.29	followup	48.00	0.08	[-0.20; 0.36]
Reich, RR, et al. 2016	167	70.87	18.02	155	69.36	18.78	followup	6.00	0.08	[-0.14; 0.30]
Powell, CB, et al., 2008	21	18.50	5.40	43	18.00	5.90	followup	12.00	0.09	[-0.44; 0.61]
Arving, C, et al., 2007	47	76.00	24.00	38	74.00	20.00	followup	12.00	0.09	[-0.34; 0.52]
Klafke, N, et al., 2019	120	59.10	25.40	114	56.50	23.00	followup	24.00	0.11	[-0.15; 0.36]
Braeken APB, et al. 2013	136	83.66	20.80	144	81.23	20.60	followup	48.00	0.12	[-0.12; 0.35]
Qiu, H, et al., 2018 II.	98	14.58	9.06	196	13.52	7.89	followup	24.00	0.13	[-0.12; 0.37]
Qiu, H, et al., 2018 II.	98	14.23	7.67	196	13.10	8.87	followup	12.00	0.13	[-0.11; 0.38]
Hernandez, EG, et al. 2018	28	14.00	4.69	28	13.32	5.32	followup	24.00	0.13	[-0.39; 0.66]
Klinkhammer-Schalke, M et al., 2012	100	66.50	26.80	100	59.06	29.53	followup	36.00	0.26	[-0.02; 0.54]
Rodrigez, B, et al., 2014	8	88.54	22.24	7	79.76	36.28	followup	0.10	0.28	[-0.74; 1.30]
Nápoles AM, et al. 2015	76	15.93	3.52	75	14.73	4.33	followup	12.00	0.30	[-0.02; 0.62]
Hernandez, EG, et al. 2018	28	16.12	4.09	28	14.72	4.63	followup	8.00	0.32	[-0.21; 0.84]
Arving, C, et al., 2007	47	82.00	20.00	38	75.00	24.00	followup	24.00	0.32	[-0.11; 0.75]
Klinkhammer-Schalke, M et al., 2012	100	66.25	25.31	100	56.58	28.54	followup	24.00	0.36	[0.08; 0.64]
Klafke, N, et al., 2019	120	65.90	25.60	114	56.30	24.70	followup	48.00	0.38	[0.12; 0.64]
Schofield, P, et al., 2013	55	81.43	20.19	53	73.23	20.40	followup	8.00	0.40	[0.02; 0.78]
Seliniotaki, T, et al., 2021	27	74.50	24.50	26	61.70	36.70	followup	8.00	0.41	[-0.14; 0.95]
Nápoles AM, et al. 2015	76	16.39	3.30	75	14.89	3.95	followup	24.00	0.41	[0.09; 0.73]
Qiu, H, et al., 2018 I.	98	15.42	9.06	196	11.90	7.89	followup	4.00	0.42	[0.18; 0.67]
Elyasi, F, et al., 2021 I.	15	11.00	3.40	15	9.50	2.60	followup	24.00	0.48	[-0.25; 1.21]
Arving, C, et al., 2007	47	80.00	21.00	38	69.00	23.00	followup	4.00	0.50	[0.06; 0.93]
Rodrigez, B, et al., 2014	8	93.33	10.87	7	80.56	26.79	followup	2.00	0.60	[-0.44; 1.65]
Guo, Z, et al., 2013	89	74.18	11.43	89	66.03	14.00	followup	2.00	0.64	[0.33; 0.94]
Qiu, H, et al., 2018 I.	98	19.23	9.76	196	13.10	8.87	followup	12.00	0.67	[0.42; 0.91]
Qiu, H, et al., 2018 I.	98	20.00	8.37	196	13.52	7.89	followup	24.00	0.80	[0.55; 1.05]
Liu, T, et al., 2019	49	63.49	13.78	53	51.93	14.62	followup	9.00	0.81	[0.40; 1.21]
Gao Q, et al. 2020	40	-49.55	4.20	40	-53.56	3.25	followup	4.00	1.06	[0.59; 1.53]
Zhou, J, et al., 2020	59	64.81	9.32	59	55.54	7.29	followup	2.00	1.10	[0.71; 1.49]
Liu, T, et al., 2019	49	74.12	9.50	53	56.57	16.10	followup	12.00	1.31	[0.88; 1.73]
Qin, X, et al., 2017	50	90.72	8.27	50	75.60	10.01	followup	2.00	1.63	[1.18; 2.09]
Lu, Z, et al., 201	203	85.40	3.50	103	77.60	5.60	followup	9.00	1.80	[1.52; 2.08]
van der Meulen, IC, et al., 2013	88	84.90	3.00	91	77.20	2.80	followup	48.00	2.64	[2.24; 3.05]
Fang, P, et al.,2020	60	63.52	6.47	60	47.56	5.14	followup	4.00	2.71	[2.21; 3.21]
Zhao, X, et al., 2021	52	71.02	2.31	51	58.91	5.21	followup	12.00	2.99	[2.42; 3.56]
Rahmani, S, et al. 2015	12	56.94	11.14	12	22.91	10.73	followup	8.00	3.00	[1.78; 4.23]
Henderson, VP, et al., 2013	53	18.00	0.40	58	16.90	0.30	followup	16.00	3.11	[2.55; 3.67]
Random effect	6503			6955					0.34	[0.07; 0.61]
Prediction interval										[-1.05; 1.73]
telephone										
Dirksen, S. et al, 2007	34	20.10	2.80	38	20.40	3.10	baseline	0.00	-0.10	[-0.56; 0.36]
Thomas, ML, et al., 2012	64	16.50	5.60	88	16.70	5.30	baseline	0.00	-0.04	[-0.36; 0.29]
Girgis, A, et al., 2009 II.	120	79.90	20.30	117	79.70	22.70	baseline	0.00	0.01	[-0.25; 0.26]
Yun et al., 2017	134	76.80	19.40	72	73.00	23.00	baseline	0.00	0.18	[-0.10; 0.47]
Kim, SH, et al., 2021	47	72.00	17.70	47	65.40	21.00	baseline	0.00	0.34	[-0.07; 0.74]
Girgis, A, et al., 2009 II.	120	85.70	16.40	117	84.90	18.50	followup	12.00	0.05	[-0.21; 0.30]
Dirksen, S. et al, 2007	34	20.80	2.30	38	20.60	4.00	followup	10.00	0.06	[-0.40; 0.52]
Yun et al., 2017	134	78.00	19.90	72	75.90	18.30	followup	48.00	0.11	[-0.18; 0.39]
Girgis, A, et al., 2009 II.	120	86.70	18.70	117	84.40	18.90	followup	24.00	0.12	[-0.13; 0.38]
Thomas, ML, et al., 2012	64	17.60	5.30	88	16.80	4.90	followup	12.00	0.16	[-0.17; 0.48]
Kim, SH, et al., 2021	47	75.90	16.30	47	71.70	18.40	followup	20.00	0.24	[-0.17; 0.65]
Kim, SH, et al., 2021	47	79.00	15.70	47	69.60	18.90	followup	8.00	0.54	[0.12; 0.95]
McCusker, J, et al., 2021	99	43.30	9.20	119	37.40	9.80	followup	24.00	0.62	[0.34; 0.89]
Random effect	1064			1007					0.22	[-0.04; 0.48]
Prediction interval										[-1.35; 1.79]
online										
Peng, L, et al., 2022	28	69.94	16.25	29	72.41	16.08	baseline	0.00	-0.15	[-0.67; 0.37]
Wu, Q, et al., 2021	43	62.34	6.76	43	62.45	6.63	baseline	0.00	-0.02	[-0.44; 0.41]
Penedo, FJ, et al., 2020	95	-20.03	3.80	97	-20.46	3.74	baseline	0.00	0.11	[-0.17; 0.40]
Cengiz, HO, et al., 2023	32	12.90	5.40	33	9.75	5.22	baseline	0.00	0.59	[0.09; 1.08]
Beatty, L. et al. 2015	30	69.03	4.81	30	69.16	4.79	followup	6.00	-0.03	[-0.53; 0.48]
Penedo, FJ, et al., 2020	95	-20.18	3.80	97	-20.62	3.84	followup	24.00	0.11	[-0.17; 0.40]
Penedo, FJ, et al., 2020	95	-19.20	3.90	97	-20.43	3.84	followup	48.00	0.32	[0.03; 0.60]
Cengiz, HO, et al., 2023	32	13.56	5.78	33	8.75	5.14	followup	8.00	0.87	[0.36; 1.38]
Wu, Q, et al., 2021	43	81.54	8.21	43	74.21	7.33	followup	12.00	0.93	[0.49; 1.38]
Peng, L, et al., 2022	28	80.95	13.20	29	70.12	9.05	followup	4.00	0.95	[0.40; 1.50]
Beatty, L. et al. 2015	30	72.59	4.87	30	67.43	4.89	followup	13.03	1.04	[0.50; 1.59]
Peng, L, et al., 2022	28	83.33	14.87	29	69.25	8.94	followup	0.10	1.14	[0.57; 1.70]
Beatty, L. et al. 2015	30	76.54	4.22	30	71.38	4.21	followup	26.07	1.21	[0.66; 1.76]
Random effect	609			620					0.51	[0.19; 0.83]
Prediction interval										[-1.17; 2.19]
**Random effect**	**8176**			**8582**					**0.34**	**[0.11; 0.57]**
**Prediction interval**										**[-1.03; 1.71]**

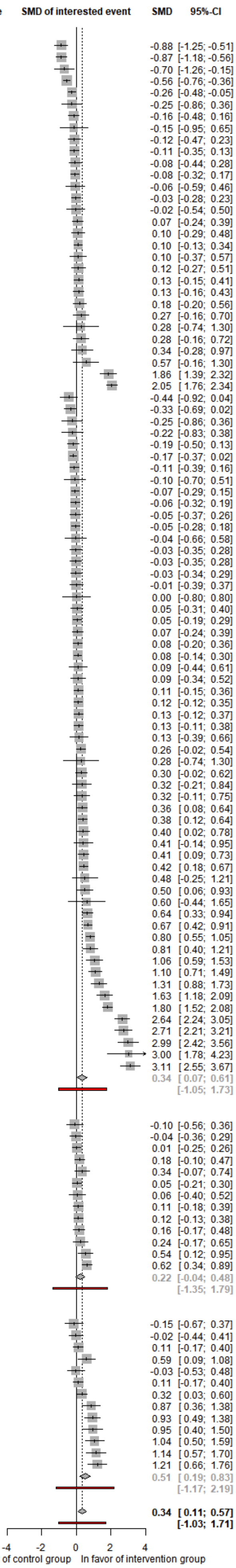

Figure S9.3. Forest plot represents the difference between the intervention vs. control group in the Emotional QoL domain with the environment subgroups as predicted at week 24 (post-intervention). SMD - Standardized mean difference, CI - confidence interval.

Figure S9.4.T48

Study	Experimental Patient N	Experimental Mean	Experimental SD	Control Patient N	Control Mean	Control SD	follow-up	Follow-up time	SMD	95%-CI
**face to face**										
Zhao,X, et al. 2015	62	5.41	1.79	62	7.24	2.31	baseline	0.00	-0.88	[-1.25; -0.51]
van der Meulen, IC, et al., 2013	88	64.80	2.40	91	66.90	2.40	baseline	0.00	-0.87	[-1.18; -0.56]
Seliniotaki, T, et al., 2021	27	56.10	29.10	26	74.20	20.90	baseline	0.00	-0.70	[-1.26; -0.15]
McLachlan, SA, et al., 2001	296	46.00	29.20	154	61.10	22.00	baseline	0.00	-0.56	[-0.76; -0.36]
Reich, RR, et al. 2016	167	63.83	17.42	155	68.57	18.32	baseline	0.00	-0.26	[-0.48; -0.05]
Serfaty M, et al., 2018	20	12.30	5.20	22	13.60	4.90	baseline	0.00	-0.25	[-0.86; 0.36]
Nápoles AM, et al. 2015	76	12.07	4.91	75	12.86	5.14	baseline	0.00	-0.16	[-0.48; 0.16]
Rahmani, S, et al. 2015	12	11.11	8.94	12	12.50	9.05	baseline	0.00	-0.15	[-0.95; 0.65]
Berglund, G. et al. 2007	39	81.20	18.90	150	83.60	19.70	baseline	0.00	-0.12	[-0.47; 0.23]
Qiu, H, et al., 2018 II.	98	11.50	6.30	196	12.22	6.56	baseline	0.00	-0.11	[-0.35; 0.13]
Fang, P, et al.,2020	60	46.14	5.12	60	46.55	5.23	baseline	0.00	-0.08	[-0.44; 0.28]
Qiu, H, et al., 2018 I.	98	11.72	6.65	196	12.22	6.56	baseline	0.00	-0.08	[-0.32; 0.17]
Hernandez, EG, et al. 2018	28	13.07	5.26	28	13.39	4.94	baseline	0.00	-0.06	[-0.59; 0.46]
Klafke, N, et al., 2019	120	50.00	26.00	114	50.70	26.50	baseline	0.00	-0.03	[-0.28; 0.23]
Powell, CB, et al., 2008	21	16.10	4.20	43	16.20	5.00	baseline	0.00	-0.02	[-0.54; 0.50]
Chan, et al. 2005	80	58.10	45.50	75	54.60	48.48	baseline	0.00	0.07	[-0.24; 0.39]
Liu, T, et al., 2019	49	71.18	18.38	53	69.23	22.04	baseline	0.00	0.10	[-0.29; 0.48]
Braeken APB, et al. 2013	136	78.40	20.77	144	76.12	24.16	baseline	0.00	0.10	[-0.13; 0.34]
Penedo, FJ, et al., 2007	41	-19.24	4.04	30	-19.63	3.43	baseline	0.00	0.10	[-0.37; 0.57]
Qin, X, et al., 2017	50	73.61	10.68	50	72.41	9.34	baseline	0.00	0.12	[-0.27; 0.51]
Klinkhammer-Schalke, M et al., 2012	100	51.86	29.03	100	48.14	28.29	baseline	0.00	0.13	[-0.15; 0.41]
Guo, Z, et al., 2013	89	72.57	12.51	89	70.77	14.71	baseline	0.00	0.13	[-0.16; 0.43]
Schofield, P, et al., 2013	55	77.12	22.92	53	72.93	23.08	baseline	0.00	0.18	[-0.20; 0.56]
Arving, C, et al., 2007	47	69.00	25.00	38	63.00	18.00	baseline	0.00	0.27	[-0.16; 0.70]
Rodrigez, B, et al., 2014	8	88.54	22.24	7	79.76	36.28	baseline	0.00	0.28	[-0.74; 1.30]
Gao Q, et al. 2020	40	-56.85	4.66	40	-58.20	4.78	baseline	0.00	0.28	[-0.16; 0.72]
Elyasi, F, et al., 2021 II.	20	10.60	3.10	20	9.60	2.60	baseline	0.00	0.34	[-0.28; 0.97]
Elyasi, F, et al., 2021 I.	15	11.50	3.80	15	9.60	2.60	baseline	0.00	0.57	[-0.16; 1.30]
Zhao, X, et al., 2021	52	64.03	4.42	51	58.12	0.42	baseline	0.00	1.86	[1.39; 2.32]
Lu, Z, et al., 201	203	79.70	3.60	103	71.30	4.90	baseline	0.00	2.05	[1.76; 2.34]
Penedo, FJ, et al., 2007	41	-20.73	3.31	30	-19.20	3.62	followup	12.00	-0.44	[-0.92; 0.04]
Berglund, G. et al. 2007	39	81.60	20.10	150	87.80	18.00	followup	48.00	-0.33	[-0.69; 0.02]
Serfaty M, et al., 2018	20	15.40	4.50	22	16.60	4.90	followup	12.00	-0.25	[-0.86; 0.36]
Serfaty M, et al., 2018	20	15.60	4.20	22	16.50	3.70	followup	24.00	-0.22	[-0.83; 0.38]
Chan, et al. 2005	80	65.70	47.01	75	73.94	39.30	followup	12.00	-0.19	[-0.50; 0.13]
McLachlan, SA, et al., 2001	296	45.80	30.20	154	50.70	25.00	followup	24.00	-0.17	[-0.37; 0.02]
Klinkhammer-Schalke, M et al., 2012	100	55.34	28.29	100	58.56	28.29	followup	12.00	-0.11	[-0.39; 0.16]
Serfaty M, et al., 2018	20	16.60	5.50	22	17.10	4.40	followup	18.00	-0.10	[-0.70; 0.51]
Reich, RR, et al. 2016	167	71.33	19.41	155	72.71	19.13	followup	12.00	-0.07	[-0.29; 0.15]
Klafke, N, et al., 2019	120	62.80	22.30	114	64.20	22.40	followup	12.00	-0.06	[-0.32; 0.19]
Chan, et al. 2005	80	75.77	73.38	75	79.44	64.08	followup	72.00	-0.05	[-0.37; 0.26]
Braeken APB, et al. 2013	136	78.38	22.75	144	79.46	20.68	followup	12.00	-0.05	[-0.28; 0.18]
Elyasi, F, et al., 2021 II.	20	9.40	2.10	20	9.50	2.60	followup	24.00	-0.04	[-0.66; 0.58]
Chan, et al. 2005	80	76.73	57.02	75	78.59	56.21	followup	48.00	-0.03	[-0.35; 0.28]
Chan, et al. 2005	80	73.39	67.76	75	75.30	55.80	followup	60.00	-0.03	[-0.35; 0.28]
Chan, et al. 2005	80	68.59	48.12	75	69.81	48.88	followup	24.00	-0.03	[-0.34; 0.29]
Schofield, P, et al., 2013	55	75.31	22.19	53	75.51	22.22	followup	12.00	-0.01	[-0.39; 0.37]
Rahmani, S, et al. 2015	12	12.00	48.61	12	12.00	23.61	followup	16.00	0.00	[-0.80; 0.80]
Zhao,X, et al. 2015	62	7.91	2.24	62	7.81	1.98	followup	0.30	0.05	[-0.31; 0.40]
Qiu, H, et al., 2018 II.	98	12.32	9.06	196	11.90	7.89	followup	4.00	0.05	[-0.19; 0.29]
Chan, et al. 2005	80	77.37	58.41	75	73.44	52.28	followup	36.00	0.07	[-0.24; 0.39]
Klinkhammer-Schalke, M et al., 2012	100	63.52	27.07	100	61.29	27.29	followup	48.00	0.08	[-0.20; 0.36]
Reich, RR, et al. 2016	167	70.87	18.02	155	69.36	18.78	followup	6.00	0.08	[-0.14; 0.30]
Powell, CB, et al., 2008	21	18.50	5.40	43	18.00	5.90	followup	12.00	0.09	[-0.44; 0.61]
Arving, C, et al., 2007	47	76.00	24.00	38	74.00	20.00	followup	12.00	0.09	[-0.34; 0.52]
Klafke, N, et al., 2019	120	59.10	25.40	114	56.50	23.00	followup	24.00	0.11	[-0.15; 0.36]
Braeken APB, et al. 2013	136	83.66	20.80	144	81.23	20.60	followup	48.00	0.12	[-0.12; 0.35]
Qiu, H, et al., 2018 II.	98	14.58	9.06	196	13.52	7.89	followup	24.00	0.13	[-0.12; 0.37]
Qiu, H, et al., 2018 II.	98	14.23	7.67	196	13.10	8.87	followup	12.00	0.13	[-0.11; 0.38]
Hernandez, EG, et al. 2018	28	14.00	4.69	28	13.32	5.32	followup	24.00	0.13	[-0.39; 0.66]
Klinkhammer-Schalke, M et al., 2012	100	66.50	26.80	100	59.06	29.53	followup	36.00	0.26	[-0.02; 0.54]
Rodrigez, B, et al., 2014	8	88.54	22.24	7	79.76	36.28	followup	0.10	0.28	[-0.74; 1.30]
Nápoles AM, et al. 2015	76	15.93	3.52	75	14.73	4.33	followup	12.00	0.30	[-0.02; 0.62]
Hernandez, EG, et al. 2018	28	16.12	4.09	28	14.72	4.63	followup	8.00	0.32	[-0.21; 0.84]
Arving, C, et al., 2007	47	82.00	20.00	38	75.00	24.00	followup	24.00	0.32	[-0.11; 0.75]
Klinkhammer-Schalke, M et al., 2012	100	66.25	25.31	100	56.58	28.54	followup	24.00	0.36	[0.08; 0.64]
Klafke, N, et al., 2019	120	65.90	25.60	114	56.30	24.70	followup	48.00	0.38	[0.12; 0.64]
Schofield, P, et al., 2013	55	81.43	20.19	53	73.23	20.40	followup	8.00	0.40	[0.02; 0.78]
Seliniotaki, T, et al., 2021	27	74.50	24.50	26	61.70	36.70	followup	8.00	0.41	[-0.14; 0.95]
Nápoles AM, et al. 2015	76	16.39	3.30	75	14.89	3.95	followup	24.00	0.41	[0.09; 0.73]
Qiu, H, et al., 2018 I.	98	15.42	9.06	196	11.90	7.89	followup	4.00	0.42	[0.18; 0.67]
Elyasi, F, et al., 2021 I.	15	11.00	3.40	15	9.50	2.60	followup	24.00	0.48	[-0.25; 1.21]
Arving, C, et al., 2007	47	80.00	21.00	38	69.00	23.00	followup	4.00	0.50	[0.06; 0.93]
Rodrigez, B, et al., 2014	8	93.33	10.87	7	80.56	26.79	followup	2.00	0.60	[-0.44; 1.65]
Guo, Z, et al., 2013	89	74.18	11.43	89	66.03	14.00	followup	2.00	0.64	[0.33; 0.94]
Qiu, H, et al., 2018 I.	98	19.23	9.76	196	13.10	8.87	followup	12.00	0.67	[0.42; 0.91]
Qiu, H, et al., 2018 I.	98	20.00	8.37	196	13.52	7.89	followup	24.00	0.80	[0.55; 1.05]
Liu, T, et al., 2019	49	63.49	13.78	53	51.93	14.62	followup	9.00	0.81	[0.40; 1.21]
Gao Q, et al. 2020	40	-49.55	4.20	40	-53.56	3.25	followup	4.00	1.06	[0.59; 1.53]
Zhou, J, et al., 2020	59	64.81	9.32	59	55.54	7.29	followup	2.00	1.10	[0.71; 1.49]
Liu, T, et al., 2019	49	74.12	9.50	53	56.57	16.10	followup	12.00	1.31	[0.88; 1.73]
Qin, X, et al., 2017	50	90.72	8.27	50	75.60	10.01	followup	2.00	1.63	[1.18; 2.09]
Lu, Z, et al., 201	203	85.40	3.50	103	77.60	5.60	followup	9.00	1.80	[1.52; 2.08]
van der Meulen, IC, et al., 2013	88	84.90	3.00	91	77.20	2.80	followup	48.00	2.64	[2.24; 3.05]
Fang, P, et al.,2020	60	63.52	6.47	60	47.56	5.14	followup	4.00	2.71	[2.21; 3.21]
Zhao, X, et al., 2021	52	71.02	2.31	51	58.91	5.21	followup	12.00	2.99	[2.42; 3.56]
Rahmani, S, et al. 2015	12	56.94	11.14	12	22.91	10.73	followup	8.00	3.00	[1.78; 4.23]
Henderson, VP, et al., 2013	53	18.00	0.40	58	16.90	0.30	followup	16.00	3.11	[2.55; 3.67]
Random effect	6503			6955					0.19	[-0.41; 0.80]
Prediction interval										[-1.60; 1.99]
**telephone**										
Dirksen, S. et al, 2007	34	20.10	2.80	38	20.40	3.10	baseline	0.00	-0.10	[-0.56; 0.36]
Thomas, ML, et al., 2012	64	16.50	5.60	88	16.70	5.30	baseline	0.00	-0.04	[-0.36; 0.29]
Girgis, A, et al., 2009 II.	120	79.90	20.30	117	79.70	22.70	baseline	0.00	0.01	[-0.25; 0.26]
Yun et al., 2017	134	76.80	19.40	72	73.00	23.00	baseline	0.00	0.18	[-0.10; 0.47]
Kim, SH, et al., 2021	47	72.00	17.70	47	65.40	21.00	baseline	0.00	0.34	[-0.07; 0.74]
Girgis, A, et al., 2009 II.	120	85.70	16.40	117	84.90	18.50	followup	12.00	0.05	[-0.21; 0.30]
Dirksen, S. et al, 2007	34	20.80	2.30	38	20.60	4.00	followup	10.00	0.06	[-0.40; 0.52]
Yun et al., 2017	134	78.00	19.90	72	75.90	18.30	followup	48.00	0.11	[-0.18; 0.39]
Girgis, A, et al., 2009 II.	120	86.70	18.70	117	84.40	18.90	followup	24.00	0.12	[-0.13; 0.38]
Thomas, ML, et al., 2012	64	17.60	5.30	88	16.80	4.90	followup	12.00	0.16	[-0.17; 0.48]
Kim, SH, et al., 2021	47	75.90	16.30	47	71.70	18.40	followup	20.00	0.24	[-0.17; 0.65]
Kim, SH, et al., 2021	47	79.00	15.70	47	69.60	18.90	followup	8.00	0.54	[0.12; 0.95]
McCusker, J, et al., 2021	99	43.30	9.20	119	37.40	9.80	followup	24.00	0.62	[0.34; 0.89]
Random effect	1064			1007					0.07	[-0.44; 0.59]
Prediction interval										[-1.51; 1.65]
**online**										
Peng, L, et al., 2022	28	69.94	16.25	29	72.41	16.08	baseline	0.00	-0.15	[-0.67; 0.37]
Wu, Q, et al., 2021	43	62.34	6.76	43	62.45	6.63	baseline	0.00	-0.02	[-0.44; 0.41]
Penedo, FJ, et al., 2020	95	-20.03	3.80	97	-20.46	3.74	baseline	0.00	0.11	[-0.17; 0.40]
Cengiz, HO, et al., 2023	32	12.90	5.40	33	9.75	5.22	baseline	0.00	0.59	[0.09; 1.08]
Beatty, L. et al. 2015	30	69.03	4.81	30	69.16	4.79	followup	6.00	-0.03	[-0.53; 0.48]
Penedo, FJ, et al., 2020	95	-20.18	3.80	97	-20.62	3.84	followup	24.00	0.11	[-0.17; 0.40]
Penedo, FJ, et al., 2020	95	-19.20	3.90	97	-20.43	3.84	followup	48.00	0.32	[0.03; 0.60]
Cengiz, HO, et al., 2023	32	13.56	5.78	33	8.75	5.14	followup	8.00	0.87	[0.36; 1.38]
Wu, Q, et al., 2021	43	81.54	8.21	43	74.21	7.33	followup	12.00	0.93	[0.49; 1.38]
Peng, L, et al., 2022	28	80.95	13.20	29	70.12	9.05	followup	4.00	0.95	[0.40; 1.50]
Beatty, L. et al. 2015	30	72.59	4.87	30	67.43	4.89	followup	13.03	1.04	[0.50; 1.59]
Peng, L, et al., 2022	28	83.33	14.87	29	69.25	8.94	followup	0.10	1.14	[0.57; 1.70]
Beatty, L. et al. 2015	30	76.54	4.22	30	71.38	4.21	followup	26.07	1.21	[0.66; 1.76]
Random effect	609			620					0.36	[-0.19; 0.92]
Prediction interval										[-1.29; 2.02]
**Random effect**	**8176**			**8582**					**0.19**	**[-0.40; 0.79]**
**Prediction interval**										**[-1.60; 1.98]**

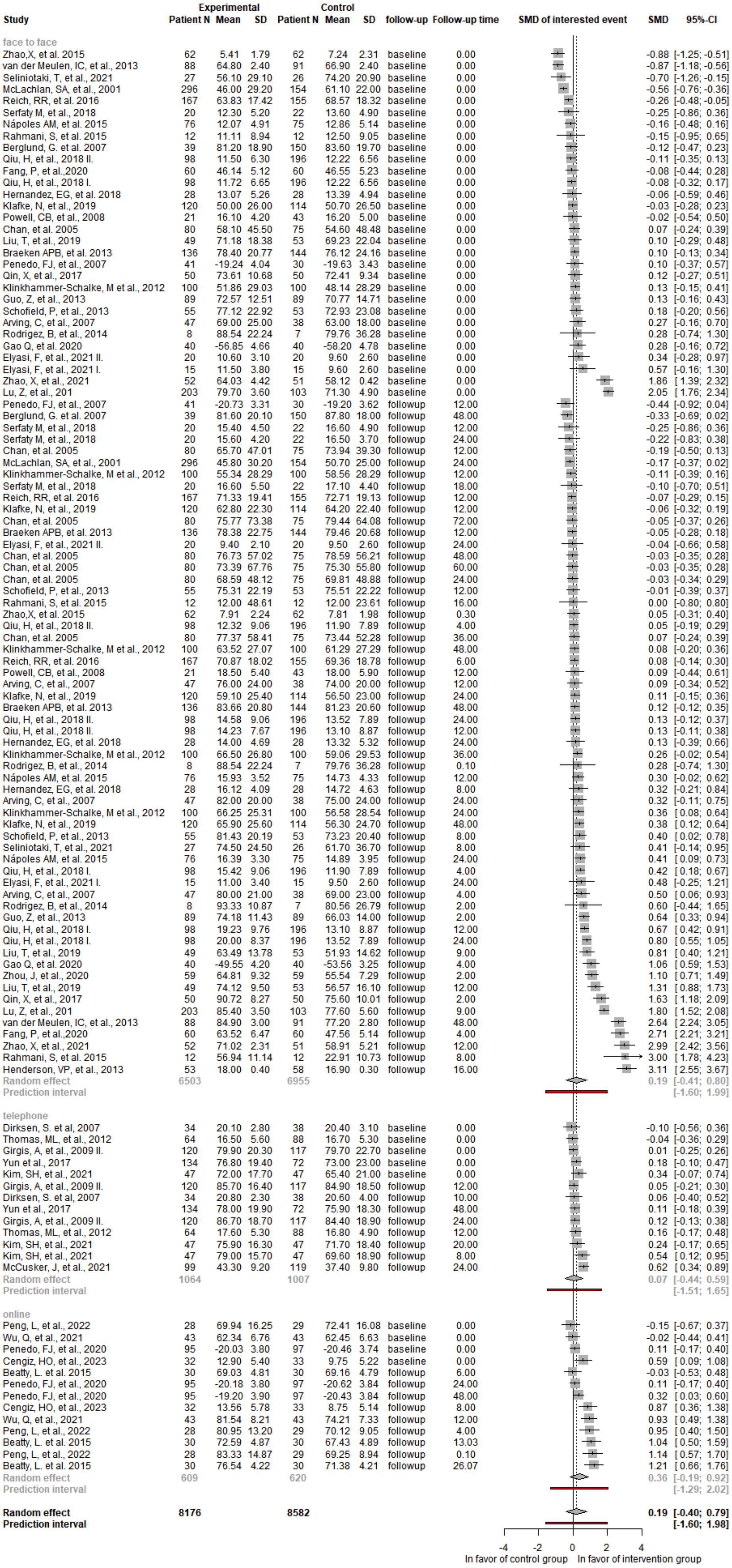

Figure S9.4. Forest plot represents the difference between the intervention vs. control group in the Emotional QoL domain with the environment subgroups as predicted at week 48 (post-intervention). SMD - Standardized mean difference, CI - confidence interval.

S10.Subgroup analysis of Emotional QoL: Type

Figure S10.1.T0

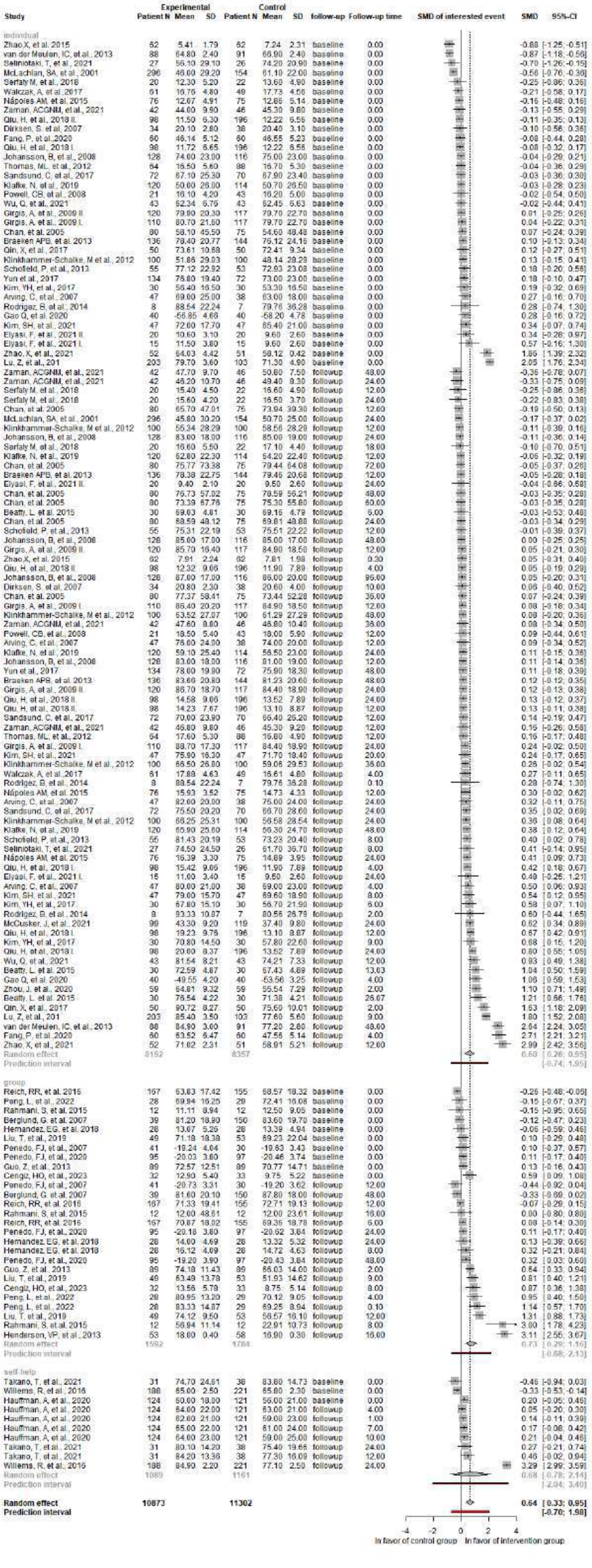

Figure S10.1. Forest plot represents the difference between the intervention vs. control group in the Emotional QoL domain with the type subgroups as predicted at week 0 (post-intervention). SMD - Standardized mean difference, CI - confidence interval.

Figure S10.2.T12

Study	Experimental Patient N	Experimental Mean	Experimental SD	Control Patient N	Control Mean	Control SD	follow-up	Follow-up time	SMD	95%-CI
individual										
Zhao,X, et al. 2015	62	5.41	1.79	62	7.24	2.31	baseline	0.00	-0.88	[-1.25; -0.51]
van der Meulen, IC, et al., 2013	88	64.80	2.40	91	66.90	2.40	baseline	0.00	-0.87	[-1.18; -0.56]
Seliniotaki, T, et al., 2021	27	56.10	29.10	26	74.20	20.90	baseline	0.00	-0.70	[-1.26; -0.15]
McLachlan, SA, et al., 2001	298	46.00	29.20	154	61.10	22.00	baseline	0.00	-0.56	[-0.76; -0.36]
Serfaty M, et al., 2018	20	12.30	5.20	22	13.60	4.90	baseline	0.00	-0.25	[-0.86; 0.36]
Walczak, A, et al.,2017	61	16.76	4.80	49	17.73	4.56	baseline	0.00	-0.21	[-0.58; 0.17]
Nápoles AM, et al. 2015	76	12.07	4.91	75	12.86	5.14	baseline	0.00	-0.16	[-0.48; 0.16]
Zaman, ACGNM, et al., 2021	42	44.00	9.90	46	45.30	9.80	baseline	0.00	-0.13	[-0.55; 0.29]
Qiu, H, et al., 2018 II.	98	11.50	6.30	196	12.22	6.56	baseline	0.00	-0.11	[-0.35; 0.13]
Dirksen, S. et al, 2007	34	20.10	2.80	38	20.40	3.10	baseline	0.00	-0.10	[-0.56; 0.36]
Fang, P, et al.,2020	60	46.14	5.12	60	46.55	5.23	baseline	0.00	-0.08	[-0.44; 0.28]
Qiu, H, et al., 2018 I.	98	11.72	6.65	196	12.22	6.56	baseline	0.00	-0.08	[-0.32; 0.17]
Johansson, B, et al., 2008	128	74.00	23.00	116	75.00	23.00	baseline	0.00	-0.04	[-0.29; 0.21]
Thomas, ML, et al., 2012	64	16.50	5.60	88	16.70	5.30	baseline	0.00	-0.04	[-0.36; 0.29]
Sandsund, C, et al., 2017	72	67.10	25.30	70	67.90	23.40	baseline	0.00	-0.03	[-0.36; 0.30]
Klafke, N, et al., 2019	120	50.00	26.00	114	50.70	26.50	baseline	0.00	-0.03	[-0.28; 0.23]
Powell, CB, et al., 2008	21	16.10	4.20	43	16.20	5.00	baseline	0.00	-0.02	[-0.54; 0.50]
Wu, Q. et al., 2021	43	62.34	6.76	43	62.45	6.63	baseline	0.00	-0.02	[-0.44; 0.41]
Girgis, A, et al., 2009 II.	120	79.90	20.30	117	79.70	22.70	baseline	0.00	0.01	[-0.25; 0.26]
Girgis, A, et al., 2009 I.	110	80.70	21.60	117	79.70	22.70	baseline	0.00	0.04	[-0.22; 0.31]
Chan, et al. 2005	80	58.10	45.50	75	54.60	48.48	baseline	0.00	0.07	[-0.24; 0.39]
Braeken APB, et al. 2013	136	76.40	20.77	144	76.12	24.16	baseline	0.00	0.10	[-0.13; 0.34]
Qin, X, et al., 2017	50	73.61	10.68	50	72.41	9.34	baseline	0.00	0.12	[-0.27; 0.51]
Klinkhammer-Schalke, M et al., 2012	100	51.86	29.03	100	48.14	28.29	baseline	0.00	0.13	[-0.15; 0.41]
Schofield, P, et al., 2013	55	77.12	22.92	53	72.93	23.08	baseline	0.00	0.18	[-0.20; 0.56]
Yun et al., 2017	134	76.80	19.40	72	73.00	23.00	baseline	0.00	0.18	[-0.10; 0.47]
Kim, YH, et al., 2017	30	56.40	16.50	30	53.30	16.50	baseline	0.00	0.19	[-0.32; 0.69]
Arving, C. et al., 2007	47	69.00	25.00	38	63.00	18.00	baseline	0.00	0.27	[-0.16; 0.70]
Rodrigez, B, et al., 2014	8	86.54	22.24	7	79.76	36.28	baseline	0.00	0.28	[-0.74; 1.30]
Gao Q, et al. 2020	40	-56.85	4.66	40	-58.20	4.78	baseline	0.00	0.28	[-0.16; 0.72]
Kim, SH, et al., 2021	47	72.00	17.70	47	65.40	21.00	baseline	0.00	0.34	[-0.07; 0.74]
Elyasi, F, et al., 2021 II.	20	10.60	3.10	20	9.60	2.60	baseline	0.00	0.34	[-0.28; 0.97]
Elyasi, F, et al., 2021 I.	15	11.50	3.80	15	9.60	2.60	baseline	0.00	0.57	[-0.16; 1.30]
Zhao, X, et al., 2021	52	64.03	4.42	51	58.12	0.42	baseline	0.00	1.86	[1.39; 2.32]
Lu, Z, et al., 201	203	79.70	3.60	103	71.30	4.90	baseline	0.00	2.05	[1.76; 2.34]
Zaman, ACGNM, et al., 2021	42	47.70	9.70	46	50.80	7.50	followup	48.00	-0.36	[-0.78; 0.07]
Zaman, ACGNM, et al., 2021	42	46.20	10.70	46	49.40	8.30	followup	24.00	-0.33	[-0.75; 0.09]
Serfaty M, et al., 2018	20	15.40	4.50	22	16.60	4.90	followup	12.00	-0.25	[-0.86; 0.36]
Serfaty M, et al., 2018	20	15.60	4.20	22	16.50	3.70	followup	24.00	-0.22	[-0.83; 0.38]
Chan, et al. 2005	80	65.70	47.01	75	73.94	39.30	followup	12.00	-0.19	[-0.50; 0.13]
McLachlan, SA, et al., 2001	298	45.80	30.20	154	50.70	25.00	followup	24.00	-0.17	[-0.37; 0.02]
Klinkhammer-Schalke, M et al., 2012	100	55.34	28.29	100	58.56	28.29	followup	12.00	-0.11	[-0.39; 0.16]
Johansson, B, et al., 2008	128	83.00	18.00	116	85.00	19.00	followup	24.00	-0.11	[-0.36; 0.14]
Serfaty M, et al., 2018	20	16.60	5.50	22	17.10	4.40	followup	18.00	-0.10	[-0.70; 0.51]
Klafke, N, et al., 2019	120	62.80	22.30	114	64.20	22.40	followup	12.00	-0.06	[-0.32; 0.19]
Chan, et al. 2005	80	75.77	73.38	75	79.44	64.08	followup	72.00	-0.05	[-0.37; 0.26]
Braeken APB, et al. 2013	136	78.38	22.75	144	79.46	20.68	followup	12.00	-0.05	[-0.28; 0.18]
Elyasi, F, et al., 2021 II.	20	9.40	2.10	20	9.50	2.60	followup	24.00	-0.04	[-0.66; 0.58]
Chan, et al. 2005	80	76.73	57.02	75	78.59	56.21	followup	48.00	-0.03	[-0.35; 0.28]
Chan, et al. 2005	80	73.39	67.76	75	75.30	55.80	followup	60.00	-0.03	[-0.35; 0.28]
Beatty, L et al. 2015	30	69.03	4.81	30	69.16	4.79	followup	6.00	-0.03	[-0.53; 0.48]
Chan, et al. 2005	80	68.59	48.12	75	69.81	46.86	followup	24.00	-0.03	[-0.34; 0.29]
Schofield, P, et al., 2013	55	75.31	22.19	53	75.51	22.22	followup	12.00	-0.01	[-0.39; 0.37]
Johansson, B, et al., 2008	128	85.00	17.00	116	85.00	17.00	followup	48.00	0.00	[-0.25; 0.25]
Girgis, A, et al., 2009 II.	120	85.70	16.40	117	84.90	18.50	followup	12.00	0.05	[-0.21; 0.30]
Zhao,X, et al. 2015	62	7.91	2.24	62	7.81	1.98	followup	0.30	0.05	[-0.31; 0.40]
Qiu, H, et al., 2018 II.	98	12.32	9.06	196	11.90	7.89	followup	4.00	0.05	[-0.19; 0.29]
Johansson, B, et al., 2008	128	87.00	17.00	116	86.00	20.00	followup	96.00	0.05	[-0.20; 0.31]
Dirksen, S. et al, 2007	34	20.80	2.30	38	20.60	4.00	followup	10.00	0.06	[-0.40; 0.52]
Chan, et al. 2005	80	77.37	58.41	75	73.44	52.28	followup	36.00	0.07	[-0.24; 0.39]
Girgis, A, et al., 2009 I.	110	86.40	20.20	117	84.90	18.50	followup	12.00	0.08	[-0.18; 0.34]
Klinkhammer-Schalke, M et al., 2012	100	63.52	27.07	100	61.29	27.29	followup	48.00	0.08	[-0.20; 0.36]
Zaman, ACGNM, et al., 2021	42	47.60	8.80	46	46.80	10.40	followup	36.00	0.08	[-0.34; 0.50]
Powell, CB, et al., 2008	21	18.50	5.40	43	18.00	5.90	followup	12.00	0.09	[-0.44; 0.61]
Arving, C. et al., 2007	47	76.00	24.00	38	74.00	20.00	followup	12.00	0.09	[-0.34; 0.52]
Klafke, N, et al., 2019	120	59.10	25.40	114	56.50	23.00	followup	24.00	0.11	[-0.15; 0.36]
Johansson, B, et al., 2008	128	83.00	18.00	116	81.00	19.00	followup	12.00	0.11	[-0.14; 0.36]
Yun et al., 2017	134	78.00	19.90	72	75.90	18.30	followup	48.00	0.11	[-0.18; 0.39]
Braeken APB, et al. 2013	136	83.66	20.80	144	81.23	20.60	followup	48.00	0.12	[-0.12; 0.35]
Girgis, A, et al., 2009 II.	120	86.70	18.70	117	84.40	18.90	followup	24.00	0.12	[-0.13; 0.38]
Qiu, H, et al., 2018 II.	98	14.58	9.06	196	13.52	7.89	followup	24.00	0.13	[-0.12; 0.37]
Qiu, H, et al., 2018 II.	98	14.23	7.67	196	13.10	8.87	followup	12.00	0.13	[-0.11; 0.38]
Sandsund, C, et al., 2017	72	70.00	23.90	70	66.40	26.20	followup	12.00	0.14	[-0.19; 0.47]
Zaman, ACGNM, et al., 2021	42	46.80	9.80	46	45.30	9.20	followup	12.00	0.16	[-0.26; 0.58]
Thomas, ML, et al., 2012	64	17.60	5.30	88	16.80	4.90	followup	12.00	0.16	[-0.17; 0.48]
Girgis, A, et al., 2009 I.	110	88.70	17.30	117	84.40	18.90	followup	24.00	0.24	[-0.02; 0.50]
Kim, SH, et al., 2021	47	75.90	16.30	47	71.70	18.40	followup	20.00	0.24	[-0.17; 0.65]
Klinkhammer-Schalke, M et al., 2012	100	66.50	26.80	100	59.06	29.53	followup	36.00	0.26	[-0.02; 0.54]
Walczak, A, et al.,2017	61	17.88	4.63	49	16.61	4.80	followup	4.00	0.27	[-0.11; 0.65]
Rodrigez, B, et al., 2014	8	86.54	22.24	7	79.76	36.28	followup	0.10	0.28	[-0.74; 1.30]
Nápoles AM, et al. 2015	76	15.93	3.52	75	14.73	4.33	followup	12.00	0.30	[-0.02; 0.62]
Arving, C, et al., 2007	47	82.00	20.00	38	75.00	24.00	followup	24.00	0.32	[-0.11; 0.75]
Sandsund, C, et al., 2017	72	75.50	20.20	70	66.70	28.60	followup	24.00	0.35	[0.02; 0.69]
Klinkhammer-Schalke, M et al., 2012	100	66.25	25.31	100	56.58	28.54	followup	24.00	0.36	[0.08; 0.64]
Klafke, N, et al., 2019	120	65.90	25.60	114	56.30	24.70	followup	48.00	0.38	[0.12; 0.64]
Schofield, P, et al., 2013	55	81.43	20.19	53	73.23	20.40	followup	8.00	0.40	[0.02; 0.78]
Seliniotaki, T, et al., 2021	27	74.50	24.50	26	61.70	36.70	followup	8.00	0.41	[-0.14; 0.95]
Nápoles AM, et al. 2015	76	16.39	3.30	75	14.89	3.95	followup	24.00	0.41	[0.09; 0.73]
Qiu, H, et al., 2018 I.	98	15.42	9.06	196	11.90	7.89	followup	4.00	0.42	[0.18; 0.67]
Elyasi, F, et al., 2021 I.	15	11.00	3.40	15	9.50	2.60	followup	24.00	0.48	[-0.25; 1.21]
Arving, C. et al., 2007	47	80.00	21.00	38	69.00	23.00	followup	4.00	0.50	[0.06; 0.93]
Kim, SH, et al., 2021	47	79.00	15.70	47	69.60	18.90	followup	8.00	0.54	[0.12; 0.95]
Kim, YH, et al., 2017	30	67.80	15.10	30	56.70	21.90	followup	6.00	0.58	[0.07; 1.10]
Rodrigez, B, et al., 2014	8	93.33	10.87	7	80.56	26.79	followup	2.00	0.60	[-0.44; 1.65]
McCusker, J, et al., 2021	99	43.30	9.20	119	37.40	9.80	followup	24.00	0.62	[0.34; 0.89]
Qiu, H, et al., 2018 I.	98	19.23	9.76	196	13.10	8.87	followup	12.00	0.67	[0.42; 0.91]
Kim, YH, et al., 2017	30	70.80	14.50	30	57.80	22.60	followup	9.00	0.68	[0.15; 1.20]
Qiu, H, et al., 2018 I.	98	20.00	8.37	196	13.52	7.89	followup	24.00	0.80	[0.55; 1.05]
Wu, Q, et al., 2021	43	81.54	8.21	43	74.21	7.33	followup	12.00	0.93	[0.49; 1.38]
Beatty, L et al. 2015	30	72.59	4.87	30	67.43	4.89	followup	13.03	1.04	[0.50; 1.59]
Gao Q, et al. 2020	40	-49.55	4.20	40	-53.56	3.25	followup	4.00	1.06	[0.59; 1.53]
Zhou, J, et al., 2020	59	64.81	9.32	59	55.54	7.29	followup	2.00	1.10	[0.71; 1.49]
Beatty, L et al. 2015	30	76.54	4.22	30	71.38	4.21	followup	26.07	1.21	[0.66; 1.76]
Qin, X, et al., 2017	50	90.72	8.27	50	75.60	10.01	followup	2.00	1.63	[1.18; 2.09]
Lu, Z, et al., 201	203	85.40	3.50	103	77.60	5.60	followup	9.00	1.80	[1.52; 2.08]
van der Meulen, IC, et al., 2013	88	84.90	3.00	91	77.20	2.90	followup	49.00	2.64	[2.24; 3.05]
Fang, P, et al.,2020	60	63.52	6.47	60	47.56	5.14	followup	4.00	2.71	[2.21; 3.21]
Zhao, X, et al., 2021	52	71.02	2.31	51	58.91	5.21	followup	12.00	2.99	[2.42; 3.56]
Random effect	8102			8357					0.46	[0.28; 0.64]
Prediction interval										[-0.84; 1.76]
group										
Reich, RR, et al. 2016	167	63.83	17.42	155	68.57	18.32	baseline	0.00	-0.26	[-0.48; -0.05]
Peng, L, et al., 2022	28	69.94	16.25	29	72.41	16.08	baseline	0.00	-0.15	[-0.67; 0.37]
Rahmani, S, et al. 2015	12	11.11	8.94	12	12.50	9.05	baseline	0.00	-0.15	[-0.95; 0.65]
Berglund, G. et al. 2007	39	81.20	18.90	150	83.60	19.70	baseline	0.00	-0.12	[-0.47; 0.23]
Hernandez, EG, et al. 2018	28	13.07	5.26	28	13.39	4.94	baseline	0.00	-0.06	[-0.59; 0.46]
Liu, T, et al., 2019	49	71.18	18.38	53	69.23	22.04	baseline	0.00	0.10	[-0.29; 0.48]
Penedo, FJ, et al., 2007	41	-19.24	4.04	30	-19.63	3.43	baseline	0.00	0.10	[-0.37; 0.57]
Penedo, FJ, et al., 2020	95	-20.03	3.80	97	-20.46	3.74	baseline	0.00	0.11	[-0.17; 0.40]
Guo, Z, et al., 2013	89	72.57	12.51	89	70.77	14.71	baseline	0.00	0.13	[-0.16; 0.43]
Cengiz, HO, et al., 2023	32	12.90	5.40	33	9.75	5.22	baseline	0.00	0.59	[0.09; 1.08]
Penedo, FJ, et al., 2007	41	-20.73	3.31	30	-19.20	3.62	followup	12.00	-0.44	[-0.92; 0.04]
Berglund, G. et al. 2007	39	81.60	20.10	150	87.80	18.00	followup	48.00	-0.33	[-0.69; 0.02]
Reich, RR, et al. 2016	167	71.33	19.41	155	72.71	19.13	followup	12.00	-0.07	[-0.29; 0.15]
Rahmani, S, et al. 2015	12	12.00	48.61	12	12.00	23.61	followup	15.00	0.00	[-0.80; 0.80]
Reich, RR, et al. 2016	167	70.87	18.02	155	69.36	18.78	followup	6.00	0.08	[-0.14; 0.30]
Penedo, FJ, et al., 2020	95	-20.18	3.80	97	-20.62	3.84	followup	24.00	0.11	[-0.17; 0.40]
Hernandez, EG, et al. 2018	28	14.00	4.69	28	13.32	5.32	followup	24.00	0.13	[-0.39; 0.66]
Hernandez, EG, et al. 2018	28	16.12	4.09	28	14.72	4.63	followup	8.00	0.32	[-0.21; 0.84]
Penedo, FJ, et al., 2020	95	-19.20	3.90	97	-20.43	3.84	followup	48.00	0.32	[0.03; 0.60]
Guo, Z, et al., 2013	89	74.18	11.43	89	66.03	14.00	followup	2.00	0.64	[0.33; 0.94]
Liu, T, et al., 2019	49	63.49	13.78	53	51.93	14.62	followup	9.00	0.81	[0.40; 1.21]
Cengiz, HO, et al., 2023	32	13.56	5.78	33	8.75	5.14	followup	8.00	0.87	[0.36; 1.38]
Peng, L, et al., 2022	28	80.95	13.20	29	70.12	9.05	followup	4.00	0.95	[0.40; 1.50]
Peng, L, et al., 2022	28	83.33	14.87	29	69.25	8.94	followup	0.10	1.14	[0.57; 1.70]
Liu, T, et al., 2019	49	74.12	9.50	53	56.57	16.10	followup	12.00	1.31	[0.88; 1.73]
Rahmani, S, et al. 2015	12	56.94	11.14	12	22.91	10.73	followup	8.00	3.00	[1.78; 4.23]
Henderson, VP, et al., 2013	53	18.00	0.40	58	16.90	0.30	followup	16.00	3.11	[2.55; 3.67]
Random effect	1592			1784					0.58	[0.21; 0.95]
Prediction interval										[-0.86; 2.03]
self-help										
Takano, T, et al., 2021	31	74.70	24.61	38	83.80	14.73	baseline	0.00	-0.46	[-0.94; 0.03]
Willems, R, et al., 2016	188	65.00	2.50	221	65.80	2.30	baseline	0.00	-0.33	[-0.53; -0.14]
Hauffman, A, et al., 2020	124	60.00	18.00	121	56.00	21.00	baseline	0.00	0.20	[-0.05; 0.46]
Hauffman, A, et al., 2020	124	64.00	22.00	121	63.00	21.00	followup	4.00	0.05	[-0.20; 0.30]
Hauffman, A, et al., 2020	124	62.00	21.00	121	59.00	23.00	followup	1.00	0.14	[-0.11; 0.39]
Hauffman, A, et al., 2020	124	65.00	22.00	121	61.00	24.00	followup	7.00	0.17	[-0.08; 0.42]
Hauffman, A, et al., 2020	124	64.00	23.00	121	59.00	25.00	followup	10.00	0.21	[-0.04; 0.46]
Takano, T, et al., 2021	31	90.10	14.20	38	75.40	19.66	followup	24.00	0.27	[-0.21; 0.74]
Takano, T, et al., 2021	31	84.20	13.36	38	77.30	16.09	followup	12.00	0.46	[-0.02; 0.94]
Willems, R, et al., 2016	188	84.90	2.20	221	77.10	2.50	followup	24.00	3.29	[2.99; 3.59]
Random effect	1089			1161					0.53	[-1.36; 2.43]
Prediction interval										[-2.96; 4.03]
**Random effect**	**10873**			**11302**					**0.49**	**[0.31; 0.67]**
**Prediction interval**										**[-0.80; 1.78]**

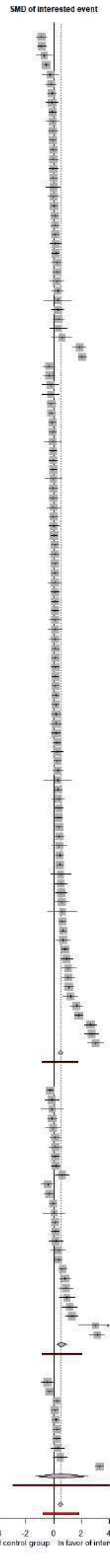

Figure S10.2. Forest plot represents the difference between the intervention vs. control group in the Emotional QoL domain with the type subgroups as predicted at week 12 (post-intervention). SMD - Standardized mean difference, CI - confidence interval.

Figure S10.3.T24

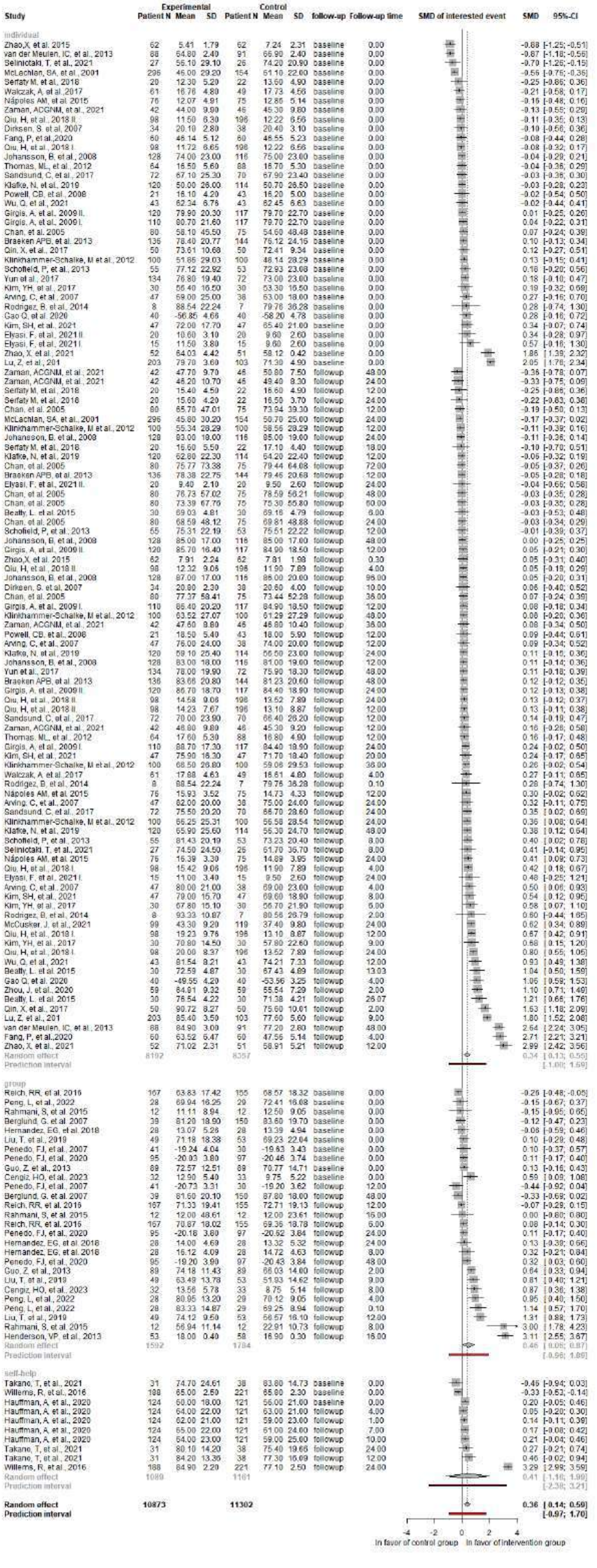

Figure S10.3. Forest plot represents the difference between the intervention vs. control group in the Emotional QoL domain with the type subgroups as predicted at week 24 (post-intervention). SMD - Standardized mean difference, CI - confidence interval.

Figure S10.4.T48

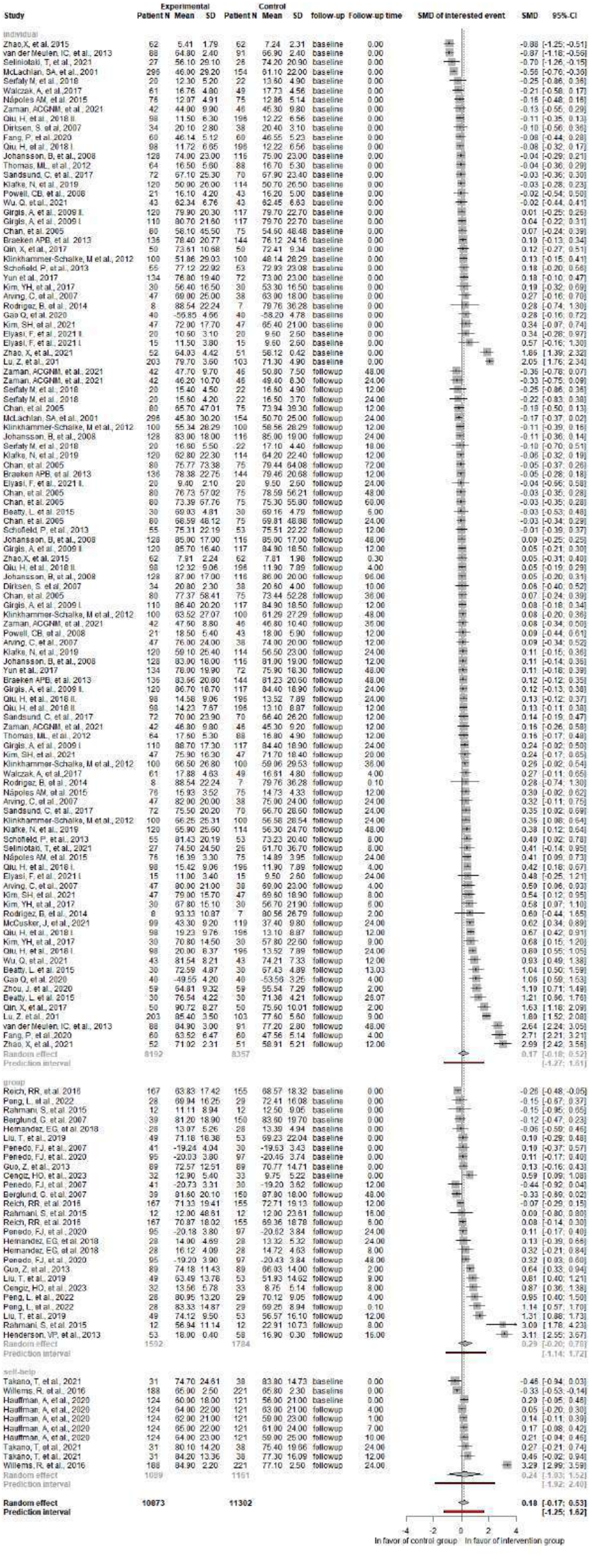

Figure S10.4. Forest plot represents the difference between the intervention vs. control group in the Emotional QoL domain with the type subgroups as predicted at week 48 (post-intervention). SMD - Standardized mean difference, CI - confidence interval

S11.Subgroup analysis of Emotional QoL: Cancer stage

Figure S11.1.T0

Study	Experimental Patient N	Experimental Mean	Experimental SD	Control Patient N	Control Mean	Control SD	follow-up	Follow-up time	SMD	95%-CI
**early**										
Zhao,X, et al. 2015	62	5.41	1.79	62	7.24	2.31	baseline	0.00	-0.88	[-1.25; -0.51]
Takano, T, et al., 2021	31	74.70	24.61	38	83.80	14.73	baseline	0.00	-0.46	[-0.94; 0.03]
Willems, R, et al., 2016	188	65.00	2.50	221	65.80	2.30	baseline	0.00	-0.33	[-0.53; -0.14]
Fang, P, et al.,2020	60	46.14	5.12	60	46.55	5.23	baseline	0.00	-0.08	[-0.44; 0.28]
Wu, Q, et al., 2021	43	62.34	6.76	43	62.45	6.63	baseline	0.00	-0.02	[-0.44; 0.41]
Liu, T, et al., 2019	49	71.18	18.38	53	69.23	22.04	baseline	0.00	0.10	[-0.29; 0.48]
Yun et al., 2017	134	76.80	19.40	72	73.00	23.00	baseline	0.00	0.18	[-0.10; 0.47]
Rodrigez, B, et al., 2014	8	88.54	22.24	7	79.76	36.28	baseline	0.00	0.28	[-0.74; 1.30]
Zhao, X, et al., 2021	52	64.03	4.42	51	58.12	0.42	baseline	0.00	1.86	[1.39; 2.32]
Beatty, L. et al. 2015	30	69.03	4.81	30	69.16	4.79	followup	6.00	-0.03	[-0.53; 0.48]
Zhao,X, et al. 2015	62	7.91	2.24	62	7.81	1.98	followup	0.30	0.05	[-0.31; 0.40]
Yun et al., 2017	134	78.00	19.90	72	75.90	18.30	followup	48.00	0.11	[-0.18; 0.39]
Takano, T, et al., 2021	31	80.10	14.20	38	75.40	19.66	followup	24.00	0.27	[-0.21; 0.74]
Rodrigez, B, et al., 2014	8	88.54	22.24	7	79.76	36.28	followup	0.10	0.28	[-0.74; 1.30]
Takano, T, et al., 2021	31	84.20	13.36	38	77.30	16.09	followup	12.00	0.46	[-0.02; 0.94]
Rodrigez, B, et al., 2014	8	93.33	10.87	7	80.56	26.79	followup	2.00	0.60	[-0.44; 1.65]
Liu, T, et al., 2019	49	63.49	13.78	53	51.93	14.62	followup	9.00	0.81	[0.40; 1.21]
Wu, Q, et al., 2021	43	81.54	8.21	43	74.21	7.33	followup	12.00	0.93	[0.49; 1.38]
Beatty, L. et al. 2015	30	72.59	4.87	30	67.43	4.89	followup	13.03	1.04	[0.50; 1.59]
Zhou, J, et al., 2020	59	64.81	9.32	59	55.54	7.29	followup	2.00	1.10	[0.71; 1.49]
Beatty, L. et al. 2015	30	76.54	4.22	30	71.38	4.21	followup	26.07	1.21	[0.66; 1.76]
Liu, T, et al., 2019	49	74.12	9.50	53	56.57	16.10	followup	12.00	1.31	[0.88; 1.73]
Fang, P, et al.,2020	60	63.52	6.47	60	47.56	5.14	followup	4.00	2.71	[2.21; 3.21]
Zhao, X, et al., 2021	52	71.02	2.31	51	58.91	5.21	followup	12.00	2.99	[2.42; 3.56]
Henderson, VP, et al., 2013	53	18.00	0.40	58	16.90	0.30	followup	16.00	3.11	[2.55; 3.67]
Willems, R, et al., 2016	188	84.90	2.20	221	77.10	2.50	followup	24.00	3.29	[2.99; 3.59]
Random effect	1544			1519					1.03	[0.24; 1.83]
Prediction interval										[-0.96; 3.03]
**advanced**										
Serfaty M, et al., 2018	20	12.30	5.20	22	13.60	4.90	baseline	0.00	-0.25	[-0.86; 0.36]
Walczak, A, et al.,2017	61	16.76	4.80	49	17.73	4.56	baseline	0.00	-0.21	[-0.58; 0.17]
Penedo, FJ, et al., 2007	41	-19.24	4.04	30	-19.63	3.43	baseline	0.00	0.10	[-0.37; 0.57]
Penedo, FJ, et al., 2020	95	-20.03	3.80	97	-20.46	3.74	baseline	0.00	0.11	[-0.17; 0.40]
Schofield, P, et al., 2013	55	77.12	22.92	53	72.93	23.08	baseline	0.00	0.18	[-0.20; 0.56]
Lu, Z, et al., 201	203	79.70	3.60	103	71.30	4.90	baseline	0.00	2.05	[1.76; 2.34]
Penedo, FJ, et al., 2007	41	-20.73	3.31	30	-19.20	3.62	followup	12.00	-0.44	[-0.92; 0.04]
Serfaty M, et al., 2018	20	15.40	4.50	22	16.60	4.90	followup	12.00	-0.25	[-0.86; 0.36]
Serfaty M, et al., 2018	20	15.60	4.20	22	16.50	3.70	followup	24.00	-0.22	[-0.83; 0.38]
Serfaty M, et al., 2018	20	16.60	5.50	22	17.10	4.40	followup	18.00	-0.10	[-0.70; 0.51]
Schofield, P, et al., 2013	55	75.31	22.19	53	75.51	22.22	followup	12.00	-0.01	[-0.39; 0.37]
Penedo, FJ, et al., 2020	95	-20.18	3.80	97	-20.62	3.84	followup	24.00	0.11	[-0.17; 0.40]
Walczak, A, et al.,2017	61	17.88	4.63	49	16.61	4.80	followup	4.00	0.27	[-0.11; 0.65]
Penedo, FJ, et al., 2020	95	-19.20	3.90	97	-20.43	3.84	followup	48.00	0.32	[0.03; 0.60]
Schofield, P, et al., 2013	55	81.43	20.19	53	73.23	20.40	followup	8.00	0.40	[0.02; 0.78]
Lu, Z, et al., 201	203	85.40	3.50	103	77.60	5.60	followup	9.00	1.80	[1.52; 2.08]
Random effect	1140			902					0.47	[-0.49; 1.43]
Prediction interval										[-1.57; 2.50]
**survivor**										
McLachlan, SA, et al., 2001	296	46.00	29.20	154	61.10	22.00	baseline	0.00	-0.56	[-0.76; -0.36]
Peng, L, et al., 2022	28	69.94	16.25	29	72.41	16.08	baseline	0.00	-0.15	[-0.67; 0.37]
Qiu, H, et al., 2018 II.	98	11.50	6.30	196	12.22	6.56	baseline	0.00	-0.11	[-0.35; 0.13]
Qiu, H, et al., 2018 I.	98	11.72	6.65	196	12.22	6.56	baseline	0.00	-0.08	[-0.32; 0.17]
Kim, SH, et al., 2021	47	72.00	17.70	47	65.40	21.00	baseline	0.00	0.34	[-0.07; 0.74]
McLachlan, SA, et al., 2001	296	45.80	30.20	154	50.70	25.00	followup	24.00	-0.17	[-0.37; 0.02]
Qiu, H, et al., 2018 II.	98	12.32	9.06	196	11.90	7.89	followup	4.00	0.05	[-0.19; 0.29]
Qiu, H, et al., 2018 II.	98	14.58	9.06	196	13.52	7.89	followup	24.00	0.13	[-0.12; 0.37]
Qiu, H, et al., 2018 II.	98	14.23	7.67	196	13.10	8.87	followup	12.00	0.13	[-0.11; 0.38]
Kim, SH, et al., 2021	47	75.90	16.30	47	71.70	18.40	followup	20.00	0.24	[-0.17; 0.65]
Qiu, H, et al., 2018 I.	98	15.42	9.06	196	11.90	7.89	followup	4.00	0.42	[0.18; 0.67]
Kim, SH, et al., 2021	47	79.00	15.70	47	69.60	18.90	followup	8.00	0.54	[0.12; 0.95]
McCusker, J, et al., 2021	99	43.30	9.20	119	37.40	9.80	followup	24.00	0.62	[0.34; 0.89]
Qiu, H, et al., 2018 I.	98	19.23	9.76	196	13.10	8.87	followup	12.00	0.67	[0.42; 0.91]
Qiu, H, et al., 2018 I.	98	20.00	8.37	196	13.52	7.89	followup	24.00	0.80	[0.55; 1.05]
Peng, L, et al., 2022	28	80.95	13.20	29	70.12	9.05	followup	4.00	0.95	[0.40; 1.50]
Peng, L, et al., 2022	28	83.33	14.87	29	69.25	8.94	followup	0.10	1.14	[0.57; 1.70]
Random effect	1700			2223					0.39	[-0.50; 1.27]
Prediction interval										[-1.75; 2.52]
**Random effect**	**4384**			**4644**					**0.73**	**[0.07; 1.40]**
**Prediction interval**										**[-1.35; 2.82]**

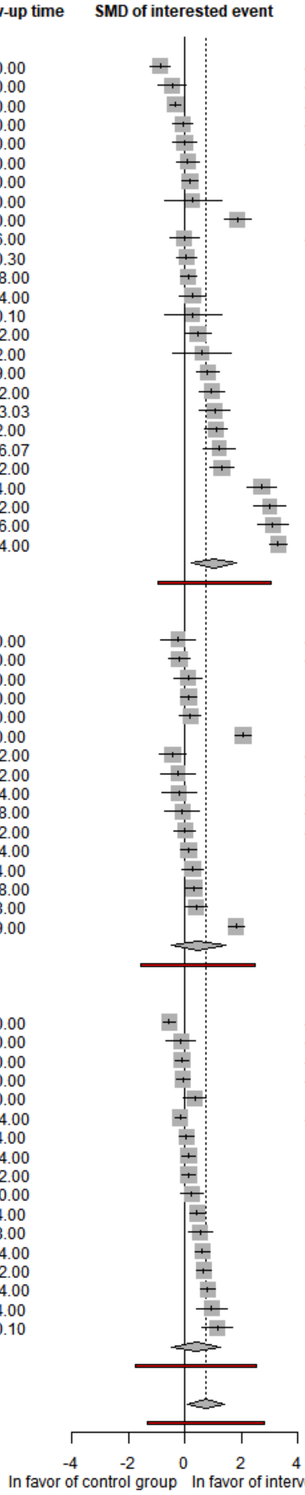

Figure S11.1. Forest plot represents the difference between the intervention vs. control group in the Emotional QoL domain with the cancer stage subgroups as predicted at week 0 (post-intervention). SMD - Standardized mean difference, CI - confidence interval.

Figure S11.2.T12

Study	Experimental Patient N	Experimental Mean	Experimental SD	Control Patient N	Control Mean	Control SD	follow-up	Follow-up time	SMD	95%-CI
early										
Zhao,X, et al. 2015	62	5.41	1.79	62	7.24	2.31	baseline	0.00	-0.88	[-1.25; -0.51]
Takano, T, et al., 2021	31	74.70	24.61	38	83.80	14.73	baseline	0.00	-0.46	[-0.94; 0.03]
Willems, R, et al., 2016	188	65.00	2.50	221	65.80	2.30	baseline	0.00	-0.33	[-0.53; -0.14]
Fang, P, et al.,2020	60	46.14	5.12	60	46.55	5.23	baseline	0.00	-0.08	[-0.44; 0.28]
Wu, Q, et al., 2021	43	62.34	6.76	43	62.45	6.63	baseline	0.00	-0.02	[-0.44; 0.41]
Liu, T, et al., 2019	49	71.18	18.38	53	69.23	22.04	baseline	0.00	0.10	[-0.29; 0.48]
Yun et al., 2017	134	76.80	19.40	72	73.00	23.00	baseline	0.00	0.18	[-0.10; 0.47]
Rodrigez, B, et al., 2014	8	88.54	22.24	7	79.76	36.28	baseline	0.00	0.28	[-0.74; 1.30]
Zhao, X, et al., 2021	52	64.03	4.42	51	58.12	0.42	baseline	0.00	1.86	[1.39; 2.32]
Beatty, L. et al. 2015	30	69.03	4.81	30	69.16	4.79	followup	6.00	-0.03	[-0.53; 0.48]
Zhao,X, et al. 2015	62	7.91	2.24	62	7.81	1.98	followup	0.30	0.05	[-0.31; 0.40]
Yun et al., 2017	134	78.00	19.90	72	75.90	18.30	followup	48.00	0.11	[-0.18; 0.39]
Takano, T, et al., 2021	31	80.10	14.20	38	75.40	19.66	followup	24.00	0.27	[-0.21; 0.74]
Rodrigez, B, et al., 2014	8	88.54	22.24	7	79.76	36.28	followup	0.10	0.28	[-0.74; 1.30]
Takano, T, et al., 2021	31	84.20	13.36	38	77.30	16.09	followup	12.00	0.46	[-0.02; 0.94]
Rodrigez, B, et al., 2014	8	93.33	10.87	7	80.56	26.79	followup	2.00	0.60	[-0.44; 1.65]
Liu, T, et al., 2019	49	63.49	13.78	53	51.93	14.62	followup	9.00	0.81	[0.40; 1.21]
Wu, Q, et al., 2021	43	81.54	8.21	43	74.21	7.33	followup	12.00	0.93	[0.49; 1.38]
Beatty, L. et al. 2015	30	72.59	4.87	30	67.43	4.89	followup	13.03	1.04	[0.50; 1.59]
Zhou, J, et al., 2020	59	64.81	9.32	59	55.54	7.29	followup	2.00	1.10	[0.71; 1.49]
Beatty, L. et al. 2015	30	76.54	4.22	30	71.38	4.21	followup	26.07	1.21	[0.66; 1.76]
Liu, T, et al., 2019	49	74.12	9.50	53	56.57	16.10	followup	12.00	1.31	[0.88; 1.73]
Fang, P, et al.,2020	60	63.52	6.47	60	47.56	5.14	followup	4.00	2.71	[2.21; 3.21]
Zhao, X, et al., 2021	52	71.02	2.31	51	58.91	5.21	followup	12.00	2.99	[2.42; 3.56]
Henderson, VP, et al., 2013	53	18.00	0.40	58	16.90	0.30	followup	16.00	3.11	[2.55; 3.67]
Willems, R, et al., 2016	188	84.90	2.20	221	77.10	2.50	followup	24.00	3.29	[2.99; 3.59]
Random effect	1544			1519					1.15	[0.50; 1.80]
Prediction interval										[-0.76; 3.06]
advanced										
Serfaty M, et al., 2018	20	12.30	5.20	22	13.60	4.90	baseline	0.00	-0.25	[-0.86; 0.36]
Walczak, A, et al.,2017	61	16.76	4.80	49	17.73	4.56	baseline	0.00	-0.21	[-0.58; 0.17]
Penedo, FJ, et al., 2007	41	-19.24	4.04	30	-19.63	3.43	baseline	0.00	0.10	[-0.37; 0.57]
Penedo, FJ, et al., 2020	95	-20.03	3.80	97	-20.46	3.74	baseline	0.00	0.11	[-0.17; 0.40]
Schofield, P, et al., 2013	55	77.12	22.92	53	72.93	23.08	baseline	0.00	0.18	[-0.20; 0.56]
Lu, Z, et al., 201	203	79.70	3.60	103	71.30	4.90	baseline	0.00	2.05	[1.76; 2.34]
Penedo, FJ, et al., 2007	41	-20.73	3.31	30	-19.20	3.62	followup	12.00	-0.44	[-0.92; 0.04]
Serfaty M, et al., 2018	20	15.40	4.50	22	16.60	4.90	followup	12.00	-0.25	[-0.86; 0.36]
Serfaty M, et al., 2018	20	15.60	4.20	22	16.50	3.70	followup	24.00	-0.22	[-0.83; 0.38]
Serfaty M, et al., 2018	20	16.60	5.50	22	17.10	4.40	followup	18.00	-0.10	[-0.70; 0.51]
Schofield, P, et al., 2013	55	75.31	22.19	53	75.51	22.22	followup	12.00	-0.01	[-0.39; 0.37]
Penedo, FJ, et al., 2020	95	-20.18	3.80	97	-20.62	3.84	followup	24.00	0.11	[-0.17; 0.40]
Walczak, A, et al.,2017	61	17.88	4.63	49	16.61	4.80	followup	4.00	0.27	[-0.11; 0.65]
Penedo, FJ, et al., 2020	95	-19.20	3.90	97	-20.43	3.84	followup	48.00	0.32	[0.03; 0.60]
Schofield, P, et al., 2013	55	81.43	20.19	53	73.23	20.40	followup	8.00	0.40	[0.02; 0.78]
Lu, Z, et al., 201	203	85.40	3.50	103	77.60	5.60	followup	9.00	1.80	[1.52; 2.08]
Random effect	1140			902					0.58	[-0.23; 1.40]
Prediction interval										[-1.57; 2.74]
survivor										
McLachlan, SA, et al., 2001	296	46.00	29.20	154	61.10	22.00	baseline	0.00	-0.56	[-0.76; -0.36]
Peng, L, et al., 2022	28	69.94	16.25	29	72.41	16.08	baseline	0.00	-0.15	[-0.67; 0.37]
Qiu, H, et al., 2018 II.	98	11.50	6.30	196	12.22	6.56	baseline	0.00	-0.11	[-0.35; 0.13]
Qiu, H, et al., 2018 I.	98	11.72	6.65	196	12.22	6.56	baseline	0.00	-0.08	[-0.32; 0.17]
Kim, SH, et al., 2021	47	72.00	17.70	47	65.40	21.00	baseline	0.00	0.34	[-0.07; 0.74]
McLachlan, SA, et al., 2001	296	45.80	30.20	154	50.70	25.00	followup	24.00	-0.17	[-0.37; 0.02]
Qiu, H, et al., 2018 II.	98	12.32	9.06	196	11.90	7.89	followup	4.00	0.05	[-0.19; 0.29]
Qiu, H, et al., 2018 II.	98	14.58	9.06	196	13.52	7.89	followup	24.00	0.13	[-0.12; 0.37]
Qiu, H, et al., 2018 II.	98	14.23	7.67	196	13.10	8.87	followup	12.00	0.13	[-0.11; 0.38]
Kim, SH, et al., 2021	47	75.90	16.30	47	71.70	18.40	followup	20.00	0.24	[-0.17; 0.65]
Qiu, H, et al., 2018 I.	98	15.42	9.06	196	11.90	7.89	followup	4.00	0.42	[0.18; 0.67]
Kim, SH, et al., 2021	47	79.00	15.70	47	69.60	18.90	followup	8.00	0.54	[0.12; 0.95]
McCusker, J, et al., 2021	99	43.30	9.20	119	37.40	9.80	followup	24.00	0.62	[0.34; 0.89]
Qiu, H, et al., 2018 I.	98	19.23	9.76	196	13.10	8.87	followup	12.00	0.67	[0.42; 0.91]
Qiu, H, et al., 2018 I.	98	20.00	8.37	196	13.52	7.89	followup	24.00	0.80	[0.55; 1.05]
Peng, L, et al., 2022	28	80.95	13.20	29	70.12	9.05	followup	4.00	0.95	[0.40; 1.50]
Peng, L, et al., 2022	28	83.33	14.87	29	69.25	8.94	followup	0.10	1.14	[0.57; 1.70]
Random effect	1700			2223					0.50	[0.10; 0.91]
Prediction interval										[-1.62; 2.62]
**Random effect**	**4384**			**4644**					**0.78**	**[0.34; 1.21]**
**Prediction interval**										**[-1.07; 2.63]**

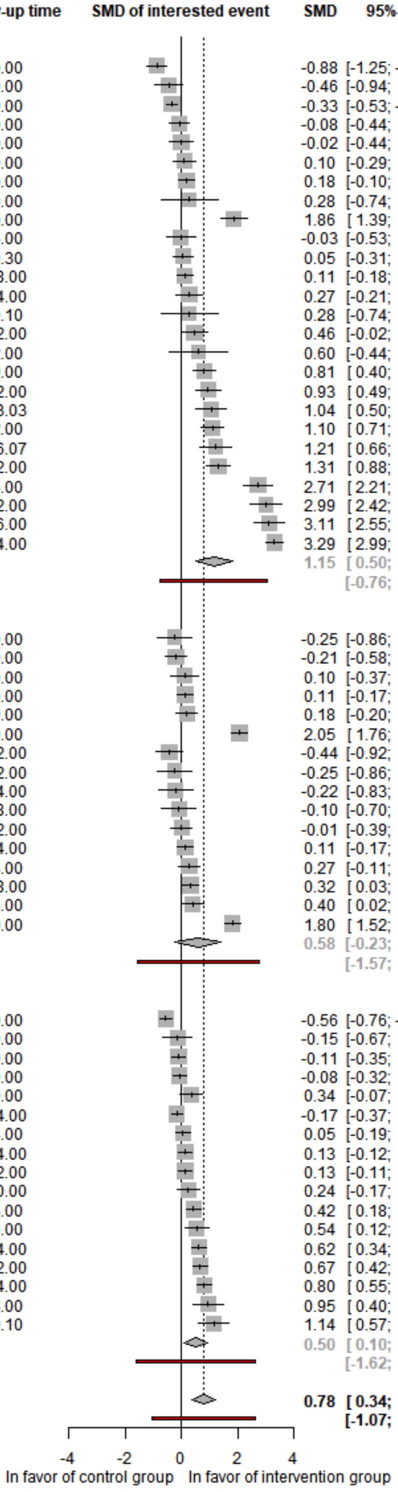

Figure S11.2. Forest plot represents the difference between the intervention vs. control group in the Emotional QoL domain with the cancer stage subgroups as predicted at week 12 (post-intervention). SMD - Standardized mean difference, CI - confidence interval.

Figure S11.3.T24

Study	Experimental Patient N	Experimental Mean	Experimental SD	Control Patient N	Control Mean	Control SD	follow-up	Follow-up time	SMD	95%-CI
early										
Zhao,X, et al. 2015	62	5.41	1.79	62	7.24	2.31	baseline	0.00	-0.88	[-1.25; -0.51]
Takano, T, et al., 2021	31	74.70	24.61	38	83.80	14.73	baseline	0.00	-0.46	[-0.94; 0.03]
Willems, R, et al., 2016	188	65.00	2.50	221	65.80	2.30	baseline	0.00	-0.33	[-0.53; -0.14]
Fang, P, et al.,2020	60	46.14	5.12	60	46.55	5.23	baseline	0.00	-0.08	[-0.44; 0.28]
Wu, Q, et al., 2021	43	62.34	6.76	43	62.45	6.63	baseline	0.00	-0.02	[-0.44; 0.41]
Liu, T, et al., 2019	49	71.18	18.38	53	69.23	22.04	baseline	0.00	0.10	[-0.29; 0.48]
Yun et al., 2017	134	76.80	19.40	72	73.00	23.00	baseline	0.00	0.18	[-0.10; 0.47]
Rodrigez, B, et al., 2014	8	88.54	22.24	7	79.76	36.28	baseline	0.00	0.28	[-0.74; 1.30]
Zhao, X, et al., 2021	52	64.03	4.42	51	58.12	0.42	baseline	0.00	1.86	[1.39; 2.32]
Beatty, L. et al. 2015	30	69.03	4.81	30	69.16	4.79	followup	6.00	-0.03	[-0.53; 0.48]
Zhao,X, et al. 2015	62	7.91	2.24	62	7.81	1.98	followup	0.30	0.05	[-0.31; 0.40]
Yun et al., 2017	134	78.00	19.90	72	75.90	18.30	followup	48.00	0.11	[-0.18; 0.39]
Takano, T, et al., 2021	31	80.10	14.20	38	75.40	19.66	followup	24.00	0.27	[-0.21; 0.74]
Rodrigez, B, et al., 2014	8	88.54	22.24	7	79.76	36.28	followup	0.10	0.28	[-0.74; 1.30]
Takano, T, et al., 2021	31	84.20	13.36	38	77.30	16.09	followup	12.00	0.46	[-0.02; 0.94]
Rodrigez, B, et al., 2014	8	93.33	10.87	7	80.56	26.79	followup	2.00	0.60	[-0.44; 1.65]
Liu, T, et al., 2019	49	63.49	13.78	53	51.93	14.62	followup	9.00	0.81	[0.40; 1.21]
Wu, Q, et al., 2021	43	81.54	8.21	43	74.21	7.33	followup	12.00	0.93	[0.49; 1.38]
Beatty, L. et al. 2015	30	72.59	4.87	30	67.43	4.89	followup	13.03	1.04	[0.50; 1.59]
Zhou, J, et al., 2020	59	64.81	9.32	59	55.54	7.29	followup	2.00	1.10	[0.71; 1.49]
Beatty, L. et al. 2015	30	76.54	4.22	30	71.38	4.21	followup	26.07	1.21	[0.66; 1.76]
Liu, T, et al., 2019	49	74.12	9.50	53	56.57	16.10	followup	12.00	1.31	[0.88; 1.73]
Fang, P, et al.,2020	60	63.52	6.47	60	47.56	5.14	followup	4.00	2.71	[2.21; 3.21]
Zhao, X, et al., 2021	52	71.02	2.31	51	58.91	5.21	followup	12.00	2.99	[2.42; 3.56]
Henderson, VP, et al., 2013	53	18.00	0.40	58	16.90	0.30	followup	16.00	3.11	[2.55; 3.67]
Willems, R, et al., 2016	188	84.90	2.20	221	77.10	2.50	followup	24.00	3.29	[2.99; 3.59]
Random effect	1544			1519					1.10	[0.30; 1.89]
Prediction interval										[-0.84; 3.04]
advanced										
Serfaty M, et al., 2018	20	12.30	5.20	22	13.60	4.90	baseline	0.00	-0.25	[-0.86; 0.36]
Walczak, A, et al.,2017	61	16.76	4.80	49	17.73	4.56	baseline	0.00	-0.21	[-0.58; 0.17]
Penedo, FJ, et al., 2007	41	-19.24	4.04	30	-19.63	3.43	baseline	0.00	0.10	[-0.37; 0.57]
Penedo, FJ, et al., 2020	95	-20.03	3.80	97	-20.46	3.74	baseline	0.00	0.11	[-0.17; 0.40]
Schofield, P, et al., 2013	55	77.12	22.92	53	72.93	23.08	baseline	0.00	0.18	[-0.20; 0.56]
Lu, Z, et al., 201	203	79.70	3.60	103	71.30	4.90	baseline	0.00	2.05	[1.76; 2.34]
Penedo, FJ, et al., 2007	41	-20.73	3.31	30	-19.20	3.62	followup	12.00	-0.44	[-0.92; 0.04]
Serfaty M, et al., 2018	20	15.40	4.50	22	16.60	4.90	followup	12.00	-0.25	[-0.86; 0.36]
Serfaty M, et al., 2018	20	15.60	4.20	22	16.50	3.70	followup	24.00	-0.22	[-0.83; 0.38]
Serfaty M, et al., 2018	20	16.60	5.50	22	17.10	4.40	followup	18.00	-0.10	[-0.70; 0.51]
Schofield, P, et al., 2013	55	75.31	22.19	53	75.51	22.22	followup	12.00	-0.01	[-0.39; 0.37]
Penedo, FJ, et al., 2020	95	-20.18	3.80	97	-20.62	3.84	followup	24.00	0.11	[-0.17; 0.40]
Walczak, A, et al.,2017	61	17.88	4.63	49	16.61	4.80	followup	4.00	0.27	[-0.11; 0.65]
Penedo, FJ, et al., 2020	95	-19.20	3.90	97	-20.43	3.84	followup	48.00	0.32	[0.03; 0.60]
Schofield, P, et al., 2013	55	81.43	20.19	53	73.23	20.40	followup	8.00	0.40	[0.02; 0.78]
Lu, Z, et al., 201	203	85.40	3.50	103	77.60	5.60	followup	9.00	1.80	[1.52; 2.08]
Random effect	1140			902					0.53	[-0.32; 1.38]
Prediction interval										[-1.55; 2.61]
survivor										
McLachlan, SA, et al., 2001	296	46.00	29.20	154	61.10	22.00	baseline	0.00	-0.56	[-0.76; -0.36]
Peng, L, et al., 2022	28	69.94	16.25	29	72.41	16.08	baseline	0.00	-0.15	[-0.67; 0.37]
Qiu, H, et al., 2018 II.	98	11.50	6.30	196	12.22	6.56	baseline	0.00	-0.11	[-0.35; 0.13]
Qiu, H, et al., 2018 I.	98	11.72	6.65	196	12.22	6.56	baseline	0.00	-0.08	[-0.32; 0.17]
Kim, SH, et al., 2021	47	72.00	17.70	47	65.40	21.00	baseline	0.00	0.34	[-0.07; 0.74]
McLachlan, SA, et al., 2001	296	45.80	30.20	154	50.70	25.00	followup	24.00	-0.17	[-0.37; 0.02]
Qiu, H, et al., 2018 II.	98	12.32	9.06	196	11.90	7.89	followup	4.00	0.05	[-0.19; 0.29]
Qiu, H, et al., 2018 II.	98	14.58	9.06	196	13.52	7.89	followup	24.00	0.13	[-0.12; 0.37]
Qiu, H, et al., 2018 II.	98	14.23	7.67	196	13.10	8.87	followup	12.00	0.13	[-0.11; 0.38]
Kim, SH, et al., 2021	47	75.90	16.30	47	71.70	18.40	followup	20.00	0.24	[-0.17; 0.65]
Qiu, H, et al., 2018 I.	98	15.42	9.06	196	11.90	7.89	followup	4.00	0.42	[0.18; 0.67]
Kim, SH, et al., 2021	47	79.00	15.70	47	69.60	18.90	followup	8.00	0.54	[0.12; 0.95]
McCusker, J, et al., 2021	99	43.30	9.20	119	37.40	9.80	followup	24.00	0.62	[0.34; 0.89]
Qiu, H, et al., 2018 I.	98	19.23	9.76	196	13.10	8.87	followup	12.00	0.67	[0.42; 0.91]
Qiu, H, et al., 2018 I.	98	20.00	8.37	196	13.52	7.89	followup	24.00	0.80	[0.55; 1.05]
Peng, L, et al., 2022	28	80.95	13.20	29	70.12	9.05	followup	4.00	0.95	[0.40; 1.50]
Peng, L, et al., 2022	28	83.33	14.87	29	69.25	8.94	followup	0.10	1.14	[0.57; 1.70]
Random effect	1700			2223					0.45	[-0.07; 0.97]
Prediction interval										[-1.52; 2.42]
**Random effect**	**4384**			**4644**					**0.70**	**[0.08; 1.32]**
**Prediction interval**										**[-1.23; 2.63]**

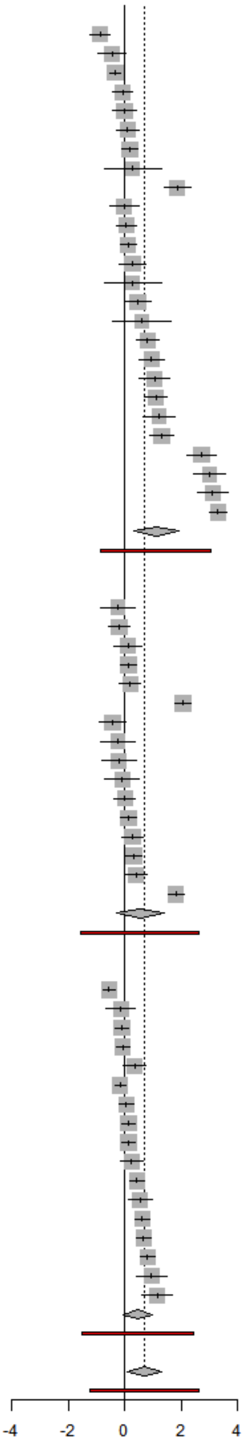

Figure S11.3. Forest plot represents the difference between the intervention vs. control group in the Emotional QoL domain with the cancer stage subgroups as predicted at week 24 (post-intervention). SMD - Standardized mean difference, CI - confidence interval.

Figure S11.4.T48

Study	Experimental Patient N	Experimental Mean	Experimental SD	Control Patient N	Control Mean	Control SD	follow-up	Follow-up time	SMD	95%-CI
**early**										
Zhao,X, et al. 2015	62	5.41	1.79	62	7.24	2.31	baseline	0.00	-0.88	[-1.25; -0.51]
Takano, T, et al., 2021	31	74.70	24.61	38	83.80	14.73	baseline	0.00	-0.46	[-0.94; 0.03]
Willems, R, et al., 2016	188	65.00	2.50	221	65.80	2.30	baseline	0.00	-0.33	[-0.53; -0.14]
Fang, P, et al.,2020	60	46.14	5.12	60	46.55	5.23	baseline	0.00	-0.08	[-0.44; 0.28]
Wu, Q, et al., 2021	43	62.34	6.76	43	62.45	6.63	baseline	0.00	-0.02	[-0.44; 0.41]
Liu, T, et al., 2019	49	71.18	18.38	53	69.23	22.04	baseline	0.00	0.10	[-0.29; 0.48]
Yun et al., 2017	134	76.80	19.40	72	73.00	23.00	baseline	0.00	0.18	[-0.10; 0.47]
Rodrigez, B, et al., 2014	8	88.54	22.24	7	79.76	36.28	baseline	0.00	0.28	[-0.74; 1.30]
Zhao, X, et al., 2021	52	64.03	4.42	51	58.12	0.42	baseline	0.00	1.86	[1.39; 2.32]
Beatty, L. et al. 2015	30	69.03	4.81	30	69.16	4.79	followup	6.00	-0.03	[-0.53; 0.48]
Zhao,X, et al. 2015	62	7.91	2.24	62	7.81	1.98	followup	0.30	0.05	[-0.31; 0.40]
Yun et al., 2017	134	78.00	19.90	72	75.90	18.30	followup	48.00	0.11	[-0.18; 0.39]
Takano, T, et al., 2021	31	80.10	14.20	38	75.40	19.66	followup	24.00	0.27	[-0.21; 0.74]
Rodrigez, B, et al., 2014	8	88.54	22.24	7	79.76	36.28	followup	0.10	0.28	[-0.74; 1.30]
Takano, T, et al., 2021	31	84.20	13.36	38	77.30	16.09	followup	12.00	0.46	[-0.02; 0.94]
Rodrigez, B, et al., 2014	8	93.33	10.87	7	80.56	26.79	followup	2.00	0.60	[-0.44; 1.65]
Liu, T, et al., 2019	49	63.49	13.78	53	51.93	14.62	followup	9.00	0.81	[0.40; 1.21]
Wu, Q, et al., 2021	43	81.54	8.21	43	74.21	7.33	followup	12.00	0.93	[0.49; 1.38]
Beatty, L. et al. 2015	30	72.59	4.87	30	67.43	4.89	followup	13.03	1.04	[0.50; 1.59]
Zhou, J, et al., 2020	59	64.81	9.32	59	55.54	7.29	followup	2.00	1.10	[0.71; 1.49]
Beatty, L. et al. 2015	30	76.54	4.22	30	71.38	4.21	followup	26.07	1.21	[0.66; 1.76]
Liu, T, et al., 2019	49	74.12	9.50	53	56.57	16.10	followup	12.00	1.31	[0.88; 1.73]
Fang, P, et al.,2020	60	63.52	6.47	60	47.56	5.14	followup	4.00	2.71	[2.21; 3.21]
Zhao, X, et al., 2021	52	71.02	2.31	51	58.91	5.21	followup	12.00	2.99	[2.42; 3.56]
Henderson, VP, et al., 2013	53	18.00	0.40	58	16.90	0.30	followup	16.00	3.11	[2.55; 3.67]
Willems, R, et al., 2016	188	84.90	2.20	221	77.10	2.50	followup	24.00	3.29	[2.99; 3.59]
Random effect	1544			1519					0.50	[-2.63; 3.63]
Prediction interval										[-6.16; 7.16]
**advanced**										
Serfaty M, et al., 2018	20	12.30	5.20	22	13.60	4.90	baseline	0.00	-0.25	[-0.86; 0.36]
Walczak, A, et al.,2017	61	16.76	4.80	49	17.73	4.56	baseline	0.00	-0.21	[-0.58; 0.17]
Penedo, FJ, et al., 2007	41	-19.24	4.04	30	-19.63	3.43	baseline	0.00	0.10	[-0.37; 0.57]
Penedo, FJ, et al., 2020	95	-20.03	3.80	97	-20.46	3.74	baseline	0.00	0.11	[-0.17; 0.40]
Schofield, P, et al., 2013	55	77.12	22.92	53	72.93	23.08	baseline	0.00	0.18	[-0.20; 0.56]
Lu, Z, et al., 201	203	79.70	3.60	103	71.30	4.90	baseline	0.00	2.05	[1.76; 2.34]
Penedo, FJ, et al., 2007	41	-20.73	3.31	30	-19.20	3.62	followup	12.00	-0.44	[-0.92; 0.04]
Serfaty M, et al., 2018	20	15.40	4.50	22	16.60	4.90	followup	12.00	-0.25	[-0.86; 0.36]
Serfaty M, et al., 2018	20	15.60	4.20	22	16.50	3.70	followup	24.00	-0.22	[-0.83; 0.38]
Serfaty M, et al., 2018	20	16.60	5.50	22	17.10	4.40	followup	18.00	-0.10	[-0.70; 0.51]
Schofield, P, et al., 2013	55	75.31	22.19	53	75.51	22.22	followup	12.00	-0.01	[-0.39; 0.37]
Penedo, FJ, et al., 2020	95	-20.18	3.80	97	-20.62	3.84	followup	24.00	0.11	[-0.17; 0.40]
Walczak, A, et al.,2017	61	17.88	4.63	49	16.61	4.80	followup	4.00	0.27	[-0.11; 0.65]
Penedo, FJ, et al., 2020	95	-19.20	3.90	97	-20.43	3.84	followup	48.00	0.32	[0.03; 0.60]
Schofield, P, et al., 2013	55	81.43	20.19	53	73.23	20.40	followup	8.00	0.40	[0.02; 0.78]
Lu, Z, et al., 201	203	85.40	3.50	103	77.60	5.60	followup	9.00	1.80	[1.52; 2.08]
Random effect	1140			902					-0.07	[-3.24; 3.10]
Prediction interval										[-6.94; 6.80]
**survivor**										
McLachlan, SA, et al., 2001	296	46.00	29.20	154	61.10	22.00	baseline	0.00	-0.56	[-0.76; -0.36]
Peng, L, et al., 2022	28	69.94	16.25	29	72.41	16.08	baseline	0.00	-0.15	[-0.67; 0.37]
Qiu, H, et al., 2018 II.	98	11.50	6.30	196	12.22	6.56	baseline	0.00	-0.11	[-0.35; 0.13]
Qiu, H, et al., 2018 I.	98	11.72	6.65	196	12.22	6.56	baseline	0.00	-0.08	[-0.32; 0.17]
Kim, SH, et al., 2021	47	72.00	17.70	47	65.40	21.00	baseline	0.00	0.34	[-0.07; 0.74]
McLachlan, SA, et al., 2001	296	45.80	30.20	154	50.70	25.00	followup	24.00	-0.17	[-0.37; 0.02]
Qiu, H, et al., 2018 II.	98	12.32	9.06	196	11.90	7.89	followup	4.00	0.05	[-0.19; 0.29]
Qiu, H, et al., 2018 II.	98	14.58	9.06	196	13.52	7.89	followup	24.00	0.13	[-0.12; 0.37]
Qiu, H, et al., 2018 II.	98	14.23	7.67	196	13.10	8.87	followup	12.00	0.13	[-0.11; 0.38]
Kim, SH, et al., 2021	47	75.90	16.30	47	71.70	18.40	followup	20.00	0.24	[-0.17; 0.65]
Qiu, H, et al., 2018 I.	98	15.42	9.06	196	11.90	7.89	followup	4.00	0.42	[0.18; 0.67]
Kim, SH, et al., 2021	47	79.00	15.70	47	69.60	18.90	followup	8.00	0.54	[0.12; 0.95]
McCusker, J, et al., 2021	99	43.30	9.20	119	37.40	9.80	followup	24.00	0.62	[0.34; 0.89]
Qiu, H, et al., 2018 I.	98	19.23	9.76	196	13.10	8.87	followup	12.00	0.67	[0.42; 0.91]
Qiu, H, et al., 2018 I.	98	20.00	8.37	196	13.52	7.89	followup	24.00	0.80	[0.55; 1.05]
Peng, L, et al., 2022	28	80.95	13.20	29	70.12	9.05	followup	4.00	0.95	[0.40; 1.50]
Peng, L, et al., 2022	28	83.33	14.87	29	69.25	8.94	followup	0.10	1.14	[0.57; 1.70]
Random effect	1700			2223					-0.15	[-2.49; 2.19]
Prediction interval										[-4.90; 4.60]
**Random effect**	**4384**			**4644**					**0.20**	**[-1.13; 1.53]**
**Prediction interval**										**[-7.33; 7.73]**

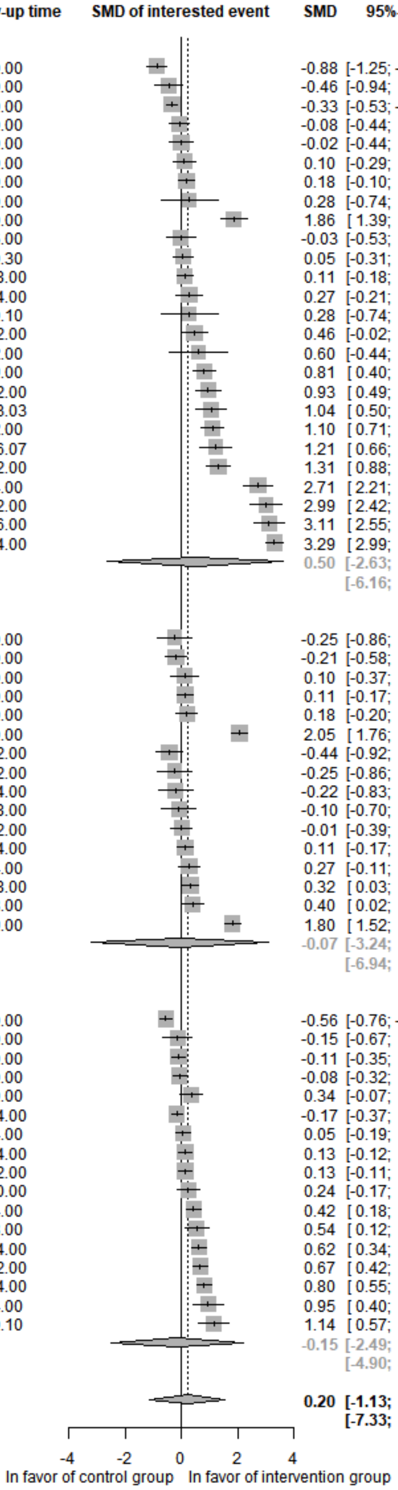

Figure S11.4. Forest plot represents the difference between the intervention vs. control group in the Emotional QoL domain with the cancer stage subgroups as predicted at week 48 (post-intervention). SMD - Standardized mean difference, CI - confidence interval.

S12.Subgroup analysis of Emotional QoL: Cancer type

Figure S12.1.T0

Study	Experimental Patient N	Experimental Mean	Experimental SD	Control Patient N	Control Mean	Control SD	follow-up	Follow-up time	SMD	95%-CI
breast										
Seliniotaki, T, et al., 2021	27	56.10	29.10	26	74.20	20.90	baseline	0.00	-0.70	[-1.26; -0.15]
Reich, RR, et al. 2016	167	63.83	17.42	155	68.57	18.32	baseline	0.00	-0.26	[-0.48; -0.05]
Nápoles AM, et al. 2015	76	12.07	4.91	75	12.86	5.14	baseline	0.00	-0.16	[-0.48; 0.16]
Peng, L, et al., 2022	28	69.94	16.25	29	72.41	16.08	baseline	0.00	-0.15	[-0.67; 0.37]
Rahmani, S, et al. 2015	12	11.11	8.94	12	12.50	9.05	baseline	0.00	-0.15	[-0.95; 0.65]
Qiu, H, et al., 2018 II.	98	11.50	6.30	196	12.22	6.56	baseline	0.00	-0.11	[-0.35; 0.13]
Dirksen, S. et al, 2007	34	20.10	2.80	38	20.40	3.10	baseline	0.00	-0.10	[-0.56; 0.36]
Qiu, H, et al., 2018 I.	98	11.72	6.65	196	12.22	6.56	baseline	0.00	-0.08	[-0.32; 0.17]
Hernandez, EG, et al. 2018	28	13.07	5.26	28	13.39	4.94	baseline	0.00	-0.06	[-0.59; 0.46]
Klafke, N, et al., 2019	120	50.00	26.00	114	50.70	26.50	baseline	0.00	-0.03	[-0.28; 0.23]
Wu, Q, et al., 2021	43	62.34	6.76	43	62.45	6.63	baseline	0.00	-0.02	[-0.44; 0.41]
Klinkhammer-Schalke, M et al., 2012	100	51.86	29.03	100	48.14	28.29	baseline	0.00	0.13	[-0.15; 0.41]
Kim, YH, et al., 2017	30	56.40	16.50	30	53.30	16.50	baseline	0.00	0.19	[-0.32; 0.69]
Hoffman, CJ, et al., 2012	102	16.91	3.84	109	15.97	4.58	baseline	0.00	0.22	[-0.05; 0.49]
Arving, C, et al., 2007	47	69.00	25.00	38	63.00	18.00	baseline	0.00	0.27	[-0.16; 0.70]
Kim, SH, et al., 2021	47	72.00	17.70	47	65.40	21.00	baseline	0.00	0.34	[-0.07; 0.74]
Elyasi, F, et al., 2021 II.	20	10.60	3.10	20	9.60	2.60	baseline	0.00	0.34	[-0.28; 0.97]
Elyasi, F, et al., 2021 I.	15	11.50	3.80	15	9.60	2.60	baseline	0.00	0.57	[-0.16; 1.30]
Cengiz, HO, et al., 2023	32	12.90	5.40	33	9.75	5.22	baseline	0.00	0.59	[0.09; 1.08]
Klinkhammer-Schalke, M et al., 2012	100	55.34	28.29	100	58.56	28.29	followup	12.00	-0.11	[-0.39; 0.16]
Reich, RR, et al. 2016	167	71.33	19.41	155	72.71	19.13	followup	12.00	-0.07	[-0.29; 0.15]
Klafke, N, et al., 2019	120	62.80	22.30	114	64.20	22.40	followup	12.00	-0.06	[-0.32; 0.19]
Elyasi, F, et al., 2021 II.	20	9.40	2.10	20	9.50	2.60	followup	24.00	-0.04	[-0.66; 0.58]
Rahmani, S, et al. 2015	12	12.00	48.61	12	12.00	23.61	followup	16.00	0.00	[-0.80; 0.80]
Qiu, H, et al., 2018 II.	98	12.32	9.06	196	11.90	7.89	followup	4.00	0.05	[-0.19; 0.29]
Dirksen, S. et al, 2007	34	20.80	2.30	38	20.60	4.00	followup	10.00	0.06	[-0.40; 0.52]
Klinkhammer-Schalke, M et al., 2012	100	63.52	27.07	100	61.29	27.29	followup	48.00	0.08	[-0.20; 0.36]
Reich, RR, et al. 2016	167	70.87	18.02	155	69.36	18.78	followup	6.00	0.08	[-0.14; 0.30]
Arving, C, et al., 2007	47	76.00	24.00	38	74.00	20.00	followup	12.00	0.09	[-0.34; 0.52]
Klafke, N, et al., 2019	120	59.10	25.40	114	56.50	23.00	followup	24.00	0.11	[-0.15; 0.36]
Qiu, H, et al., 2018 II.	98	14.58	9.06	196	13.52	7.89	followup	24.00	0.13	[-0.12; 0.37]
Qiu, H, et al., 2018 II.	98	14.23	7.67	196	13.10	8.87	followup	12.00	0.13	[-0.11; 0.38]
Hernandez, EG, et al. 2018	28	14.00	4.69	28	13.32	5.32	followup	24.00	0.13	[-0.39; 0.66]
Kim, SH, et al., 2021	47	75.90	16.30	47	71.70	18.40	followup	20.00	0.24	[-0.17; 0.65]
Klinkhammer-Schalke, M et al., 2012	100	66.50	26.80	100	59.06	29.53	followup	36.00	0.26	[-0.02; 0.54]
Nápoles AM, et al. 2015	76	15.93	3.52	75	14.73	4.33	followup	12.00	0.30	[-0.02; 0.62]
Hernandez, EG, et al. 2018	28	16.12	4.09	28	14.72	4.63	followup	8.00	0.32	[-0.21; 0.84]
Arving, C, et al., 2007	47	82.00	20.00	38	75.00	24.00	followup	24.00	0.32	[-0.11; 0.75]
Klinkhammer-Schalke, M et al., 2012	100	66.25	25.31	100	56.58	28.54	followup	24.00	0.36	[0.08; 0.64]
Hoffman, CJ, et al., 2012	102	18.14	3.82	109	16.59	4.40	followup	8.00	0.37	[0.10; 0.65]
Klafke, N, et al., 2019	120	65.90	25.60	114	56.30	24.70	followup	48.00	0.38	[0.12; 0.64]
Seliniotaki, T, et al., 2021	27	74.50	24.50	26	61.70	36.70	followup	8.00	0.41	[-0.14; 0.95]
Nápoles AM, et al. 2015	76	16.39	3.30	75	14.89	3.95	followup	24.00	0.41	[0.09; 0.73]
Qiu, H, et al., 2018 I.	98	15.42	9.06	196	11.90	7.89	followup	4.00	0.42	[0.18; 0.67]
Elyasi, F, et al., 2021 I.	15	11.00	3.40	15	9.50	2.60	followup	24.00	0.48	[-0.25; 1.21]
Arving, C, et al., 2007	47	80.00	21.00	38	69.00	23.00	followup	4.00	0.50	[0.06; 0.93]
Kim, SH, et al., 2021	47	79.00	15.70	47	69.60	18.90	followup	8.00	0.54	[0.12; 0.95]
Hoffman, CJ, et al., 2012	102	18.59	3.75	109	16.28	4.42	followup	12.00	0.56	[0.28; 0.84]
Kim, YH, et al., 2017	30	67.80	15.10	30	56.70	21.90	followup	6.00	0.58	[0.07; 1.10]
Qiu, H, et al., 2018 I.	98	19.23	9.76	196	13.10	8.87	followup	12.00	0.67	[0.42; 0.91]
Kim, YH, et al., 2017	30	70.80	14.50	30	57.80	22.60	followup	9.00	0.68	[0.15; 1.20]
Qiu, H, et al., 2018 I.	98	20.00	8.37	196	13.52	7.89	followup	24.00	0.80	[0.55; 1.05]
Cengiz, HO, et al., 2023	32	13.56	5.78	33	8.75	5.14	followup	8.00	0.87	[0.36; 1.38]
Wu, Q, et al., 2021	43	81.54	8.21	43	74.21	7.33	followup	12.00	0.93	[0.49; 1.38]
Peng, L, et al., 2022	28	80.95	13.20	29	70.12	9.05	followup	4.00	0.95	[0.40; 1.50]
Peng, L, et al., 2022	28	83.33	14.87	29	69.25	8.94	followup	0.10	1.14	[0.57; 1.70]
Rahmani, S, et al. 2015	12	56.94	11.14	12	22.91	10.73	followup	8.00	3.00	[1.78; 4.23]
Henderson, VP, et al., 2013	53	18.00	0.40	58	16.90	0.30	followup	16.00	3.11	[2.55; 3.67]
Random effect	3817			4539					0.77	[0.40; 1.15]
Prediction interval										[-0.21; 1.76]
gastroenterological										
Fang, P, et al.,2020	60	46.14	5.12	60	46.55	5.23	baseline	0.00	-0.08	[-0.44; 0.28]
Qin, X, et al., 2017	50	73.61	10.68	50	72.41	9.34	baseline	0.00	0.12	[-0.27; 0.51]
Gao Q, et al. 2020	40	-56.85	4.66	40	-58.20	4.78	baseline	0.00	0.28	[-0.16; 0.72]
Gao Q, et al. 2020	40	-49.55	4.20	40	-53.56	3.25	followup	4.00	1.06	[0.59; 1.53]
Qin, X, et al., 2017	50	90.72	8.27	50	75.60	10.01	followup	2.00	1.63	[1.18; 2.09]
Fang, P, et al.,2020	60	63.52	6.47	60	47.56	5.14	followup	4.00	2.71	[2.21; 3.21]
Random effect	300			300					1.34	[0.54; 2.14]
Prediction interval										[-0.27; 2.95]
gynaecological										
Sandsund, C, et al., 2017	72	67.10	25.30	70	67.90	23.40	baseline	0.00	-0.03	[-0.36; 0.30]
Powell, CB, et al., 2008	21	16.10	4.20	43	16.20	5.00	baseline	0.00	-0.02	[-0.54; 0.50]
Chan, et al. 2005	80	58.10	45.50	75	54.60	48.48	baseline	0.00	0.07	[-0.24; 0.39]
Chan, et al. 2005	80	65.70	47.01	75	73.94	39.30	followup	12.00	-0.19	[-0.50; 0.13]
Chan, et al. 2005	80	75.77	73.38	75	79.44	64.08	followup	72.00	-0.05	[-0.37; 0.26]
Chan, et al. 2005	80	76.73	57.02	75	78.59	56.21	followup	48.00	-0.03	[-0.35; 0.28]
Chan, et al. 2005	80	73.39	67.76	75	75.30	55.80	followup	60.00	-0.03	[-0.35; 0.28]
Chan, et al. 2005	80	68.59	48.12	75	69.81	48.88	followup	24.00	-0.03	[-0.34; 0.29]
Chan, et al. 2005	80	77.37	58.41	75	73.44	52.28	followup	36.00	0.07	[-0.24; 0.39]
Powell, CB, et al., 2008	21	18.50	5.40	43	18.00	5.90	followup	12.00	0.09	[-0.44; 0.61]
Sandsund, C, et al., 2017	72	70.00	23.90	70	66.40	26.20	followup	12.00	0.14	[-0.19; 0.47]
Sandsund, C, et al., 2017	72	75.50	20.20	70	66.70	28.60	followup	24.00	0.35	[0.02; 0.69]
Zhou, J, et al., 2020	59	64.81	9.32	59	55.54	7.29	followup	2.00	1.10	[0.71; 1.49]
Random effect	877			880					0.62	[0.13; 1.11]
Prediction interval										[-0.51; 1.75]
prostate										
Berglund, G. et al. 2007	39	81.20	18.90	150	83.60	19.70	baseline	0.00	-0.12	[-0.47; 0.23]
Penedo, FJ, et al., 2007	41	-19.24	4.04	30	-19.63	3.43	baseline	0.00	0.10	[-0.37; 0.57]
Penedo, FJ, et al., 2020	95	-20.03	3.80	97	-20.46	3.74	baseline	0.00	0.11	[-0.17; 0.40]
Penedo, FJ, et al., 2007	41	-20.73	3.31	30	-19.20	3.62	followup	12.00	-0.44	[-0.92; 0.04]
Berglund, G. et al. 2007	39	81.60	20.10	150	87.80	18.00	followup	48.00	-0.33	[-0.69; 0.02]
Penedo, FJ, et al., 2020	95	-20.18	3.80	97	-20.62	3.84	followup	24.00	0.11	[-0.17; 0.40]
Penedo, FJ, et al., 2020	95	-19.20	3.90	97	-20.43	3.84	followup	48.00	0.32	[0.03; 0.60]
Random effect	445			651					0.56	[-0.04; 1.16]
Prediction interval										[-0.66; 1.78]
**Random effect**	**5439**			**6370**					**0.85**	**[0.41; 1.29]**
**Prediction interval**										**[-0.23; 1.93]**

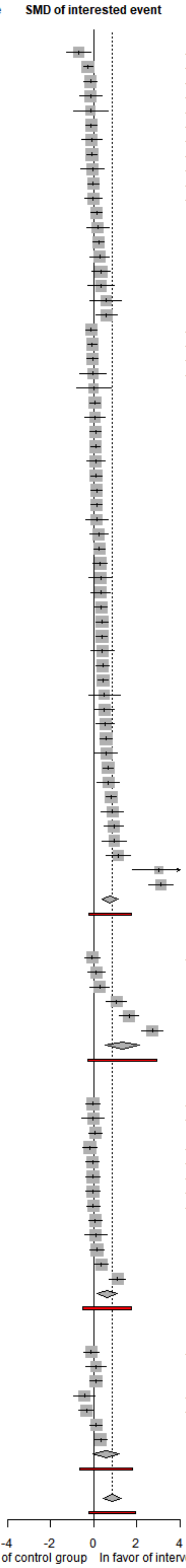

Figure S12.1. Subgroup analysis of the Emotional QoL. Forest plot represents the difference between the intervention vs. control group in the Emotional QoL domain with the cancer type subgroups as predicted at week 0 (post-intervention). SMD - Standardized mean difference, CI - confidence interval.

Figure S12.2.T12

Study	Experimental Patient N	Experimental Mean	Experimental SD	Control Patient N	Control Mean	Control SD	follow-up	Follow-up time	SMD	95%-CI
breast										
Seliniotaki, T, et al., 2021	27	56.10	29.10	26	74.20	20.90	baseline	0.00	-0.70	[-1.26; -0.15]
Reich, RR, et al. 2016	167	63.83	17.42	155	68.57	18.32	baseline	0.00	-0.26	[-0.48; -0.05]
Nápoles AM, et al. 2015	76	12.07	4.91	75	12.86	5.14	baseline	0.00	-0.16	[-0.48; 0.16]
Peng, L, et al., 2022	28	69.94	16.25	29	72.41	16.08	baseline	0.00	-0.15	[-0.67; 0.37]
Rahmani, S, et al. 2015	12	11.11	8.94	12	12.50	9.05	baseline	0.00	-0.15	[-0.95; 0.65]
Qiu, H, et al., 2018 II.	98	11.50	6.30	196	12.22	6.56	baseline	0.00	-0.11	[-0.35; 0.13]
Dirksen, S. et al, 2007	34	20.10	2.80	38	20.40	3.10	baseline	0.00	-0.10	[-0.56; 0.36]
Qiu, H, et al., 2018 I.	98	11.72	6.65	196	12.22	6.56	baseline	0.00	-0.08	[-0.32; 0.17]
Hernandez, EG, et al. 2018	28	13.07	5.26	28	13.39	4.94	baseline	0.00	-0.06	[-0.59; 0.46]
Klafke, N, et al., 2019	120	50.00	26.00	114	50.70	26.50	baseline	0.00	-0.03	[-0.28; 0.23]
Wu, Q, et al., 2021	43	62.34	6.76	43	62.45	6.63	baseline	0.00	-0.02	[-0.44; 0.41]
Klinkhammer-Schalke, M et al., 2012	100	51.86	29.03	100	48.14	28.29	baseline	0.00	0.13	[-0.15; 0.41]
Kim, YH, et al., 2017	30	56.40	16.50	30	53.30	16.50	baseline	0.00	0.19	[-0.32; 0.69]
Hoffman, CJ, et al., 2012	102	16.91	3.84	109	15.97	4.58	baseline	0.00	0.22	[-0.05; 0.49]
Arving, C, et al., 2007	47	69.00	25.00	38	63.00	18.00	baseline	0.00	0.27	[-0.16; 0.70]
Kim, SH, et al., 2021	47	72.00	17.70	47	65.40	21.00	baseline	0.00	0.34	[-0.07; 0.74]
Elyasi, F, et al., 2021 II.	20	10.60	3.10	20	9.60	2.60	baseline	0.00	0.34	[-0.28; 0.97]
Elyasi, F, et al., 2021 I.	15	11.50	3.80	15	9.60	2.60	baseline	0.00	0.57	[-0.16; 1.30]
Cengiz, HO, et al., 2023	32	12.90	5.40	33	9.75	5.22	baseline	0.00	0.59	[0.09; 1.08]
Klinkhammer-Schalke, M et al., 2012	100	55.34	28.29	100	58.56	28.29	followup	12.00	-0.11	[-0.39; 0.16]
Reich, RR, et al. 2016	167	71.33	19.41	155	72.71	19.13	followup	12.00	-0.07	[-0.29; 0.15]
Klafke, N, et al., 2019	120	62.80	22.30	114	64.20	22.40	followup	12.00	-0.06	[-0.32; 0.19]
Elyasi, F, et al., 2021 II.	20	9.40	2.10	20	9.50	2.60	followup	24.00	-0.04	[-0.66; 0.58]
Rahmani, S, et al. 2015	12	12.00	48.61	12	12.00	23.61	followup	16.00	0.00	[-0.80; 0.80]
Qiu, H, et al., 2018 II.	98	12.32	9.06	196	11.90	7.89	followup	4.00	0.05	[-0.19; 0.29]
Dirksen, S. et al, 2007	34	20.80	2.30	38	20.60	4.00	followup	10.00	0.06	[-0.40; 0.52]
Klinkhammer-Schalke, M et al., 2012	100	63.52	27.07	100	61.29	27.29	followup	48.00	0.08	[-0.20; 0.36]
Reich, RR, et al. 2016	167	70.87	18.02	155	69.36	18.78	followup	6.00	0.08	[-0.14; 0.30]
Arving, C, et al., 2007	47	76.00	24.00	38	74.00	20.00	followup	12.00	0.09	[-0.34; 0.52]
Klafke, N, et al., 2019	120	59.10	25.40	114	56.50	23.00	followup	24.00	0.11	[-0.15; 0.36]
Qiu, H, et al., 2018 II.	98	14.58	9.06	196	13.52	7.89	followup	24.00	0.13	[-0.12; 0.37]
Qiu, H, et al., 2018 II.	98	14.23	7.67	196	13.10	8.87	followup	12.00	0.13	[-0.11; 0.38]
Hernandez, EG, et al. 2018	28	14.00	4.69	28	13.32	5.32	followup	24.00	0.13	[-0.39; 0.66]
Kim, SH, et al., 2021	47	75.90	16.30	47	71.70	18.40	followup	20.00	0.24	[-0.17; 0.65]
Klinkhammer-Schalke, M et al., 2012	100	66.50	26.80	100	59.06	29.53	followup	36.00	0.26	[-0.02; 0.54]
Nápoles AM, et al. 2015	76	15.93	3.52	75	14.73	4.33	followup	12.00	0.30	[-0.02; 0.62]
Hernandez, EG, et al. 2018	28	16.12	4.09	28	14.72	4.63	followup	8.00	0.32	[-0.21; 0.84]
Arving, C, et al., 2007	47	82.00	20.00	38	75.00	24.00	followup	24.00	0.32	[-0.11; 0.75]
Klinkhammer-Schalke, M et al., 2012	100	66.25	25.31	100	56.58	28.54	followup	24.00	0.36	[0.08; 0.64]
Hoffman, CJ, et al., 2012	102	18.14	3.82	109	16.59	4.40	followup	8.00	0.37	[0.10; 0.65]
Klafke, N, et al., 2019	120	65.90	25.60	114	56.30	24.70	followup	48.00	0.38	[0.12; 0.64]
Seliniotaki, T, et al., 2021	27	74.50	24.50	26	61.70	36.70	followup	8.00	0.41	[-0.14; 0.95]
Nápoles AM, et al. 2015	76	16.39	3.30	75	14.89	3.95	followup	24.00	0.41	[0.09; 0.73]
Qiu, H, et al., 2018 I.	98	15.42	9.06	196	11.90	7.89	followup	4.00	0.42	[0.18; 0.67]
Elyasi, F, et al., 2021 I.	15	11.00	3.40	15	9.50	2.60	followup	24.00	0.48	[-0.25; 1.21]
Arving, C, et al., 2007	47	80.00	21.00	38	69.00	23.00	followup	4.00	0.50	[0.06; 0.93]
Kim, SH, et al., 2021	47	79.00	15.70	47	69.60	18.90	followup	8.00	0.54	[0.12; 0.95]
Hoffman, CJ, et al., 2012	102	18.59	3.75	109	16.28	4.42	followup	12.00	0.56	[0.28; 0.84]
Kim, YH, et al., 2017	30	67.80	15.10	30	56.70	21.90	followup	6.00	0.58	[0.07; 1.10]
Qiu, H, et al., 2018 I.	98	19.23	9.76	196	13.10	8.87	followup	12.00	0.67	[0.42; 0.91]
Kim, YH, et al., 2017	30	70.80	14.50	30	57.80	22.60	followup	9.00	0.68	[0.15; 1.20]
Qiu, H, et al., 2018 I.	98	20.00	8.37	196	13.52	7.89	followup	24.00	0.80	[0.55; 1.05]
Cengiz, HO, et al., 2023	32	13.56	5.78	33	8.75	5.14	followup	8.00	0.87	[0.36; 1.38]
Wu, Q, et al., 2021	43	81.54	8.21	43	74.21	7.33	followup	12.00	0.93	[0.49; 1.38]
Peng, L, et al., 2022	28	80.95	13.20	29	70.12	9.05	followup	4.00	0.95	[0.40; 1.50]
Peng, L, et al., 2022	28	83.33	14.87	29	69.25	8.94	followup	0.10	1.14	[0.57; 1.70]
Rahmani, S, et al. 2015	12	56.94	11.14	12	22.91	10.73	followup	8.00	3.00	[1.78; 4.23]
Henderson, VP, et al., 2013	53	18.00	0.40	58	16.90	0.30	followup	16.00	3.11	[2.55; 3.67]
Random effect	3817			4539					0.50	[0.29; 0.71]
Prediction interval										[-0.41; 1.42]
gastroenterological										
Fang, P, et al.,2020	60	46.14	5.12	60	46.55	5.23	baseline	0.00	-0.08	[-0.44; 0.28]
Qin, X, et al., 2017	50	73.61	10.68	50	72.41	9.34	baseline	0.00	0.12	[-0.27; 0.51]
Gao Q, et al. 2020	40	-56.85	4.66	40	-58.20	4.78	baseline	0.00	0.28	[-0.16; 0.72]
Gao Q, et al. 2020	40	-49.55	4.20	40	-53.56	3.25	followup	4.00	1.06	[0.59; 1.53]
Qin, X, et al., 2017	50	90.72	8.27	50	75.60	10.01	followup	2.00	1.63	[1.18; 2.09]
Fang, P, et al.,2020	60	63.52	6.47	60	47.56	5.14	followup	4.00	2.71	[2.21; 3.21]
Random effect	300			300					1.06	[0.37; 1.75]
Prediction interval										[-0.64; 2.77]
gynaecological										
Sandsund, C, et al., 2017	72	67.10	25.30	70	67.90	23.40	baseline	0.00	-0.03	[-0.36; 0.30]
Powell, CB, et al., 2008	21	16.10	4.20	43	16.20	5.00	baseline	0.00	-0.02	[-0.54; 0.50]
Chan, et al. 2005	80	58.10	45.50	75	54.60	48.48	baseline	0.00	0.07	[-0.24; 0.39]
Chan, et al. 2005	80	65.70	47.01	75	73.94	39.30	followup	12.00	-0.19	[-0.50; 0.13]
Chan, et al. 2005	80	75.77	73.38	75	79.44	64.08	followup	72.00	-0.05	[-0.37; 0.26]
Chan, et al. 2005	80	76.73	57.02	75	78.59	56.21	followup	48.00	-0.03	[-0.35; 0.28]
Chan, et al. 2005	80	73.39	67.76	75	75.30	55.80	followup	60.00	-0.03	[-0.35; 0.28]
Chan, et al. 2005	80	68.59	48.12	75	69.81	48.88	followup	24.00	-0.03	[-0.34; 0.29]
Chan, et al. 2005	80	77.37	58.41	75	73.44	52.28	followup	36.00	0.07	[-0.24; 0.39]
Powell, CB, et al., 2008	21	18.50	5.40	43	18.00	5.90	followup	12.00	0.09	[-0.44; 0.61]
Sandsund, C, et al., 2017	72	70.00	23.90	70	66.40	26.20	followup	12.00	0.14	[-0.19; 0.47]
Sandsund, C, et al., 2017	72	75.50	20.20	70	66.70	28.60	followup	24.00	0.35	[0.02; 0.69]
Zhou, J, et al., 2020	59	64.81	9.32	59	55.54	7.29	followup	2.00	1.10	[0.71; 1.49]
Random effect	877			880					0.35	[-0.00; 0.70]
Prediction interval										[-1.07; 1.76]
prostate										
Berglund, G. et al. 2007	39	81.20	18.90	150	83.60	19.70	baseline	0.00	-0.12	[-0.47; 0.23]
Penedo, FJ, et al., 2007	41	-19.24	4.04	30	-19.63	3.43	baseline	0.00	0.10	[-0.37; 0.57]
Penedo, FJ, et al., 2020	95	-20.03	3.80	97	-20.46	3.74	baseline	0.00	0.11	[-0.17; 0.40]
Penedo, FJ, et al., 2007	41	-20.73	3.31	30	-19.20	3.62	followup	12.00	-0.44	[-0.92; 0.04]
Berglund, G. et al. 2007	39	81.60	20.10	150	87.80	18.00	followup	48.00	-0.33	[-0.69; 0.02]
Penedo, FJ, et al., 2020	95	-20.18	3.80	97	-20.62	3.84	followup	24.00	0.11	[-0.17; 0.40]
Penedo, FJ, et al., 2020	95	-19.20	3.90	97	-20.43	3.84	followup	48.00	0.32	[0.03; 0.60]
Random effect	445			651					0.29	[-0.32; 0.90]
Prediction interval										[-1.33; 1.91]
**Random effect**	**5439**			**6370**					**0.53**	**[0.31; 0.74]**
**Prediction interval**										**[-0.46; 1.51]**

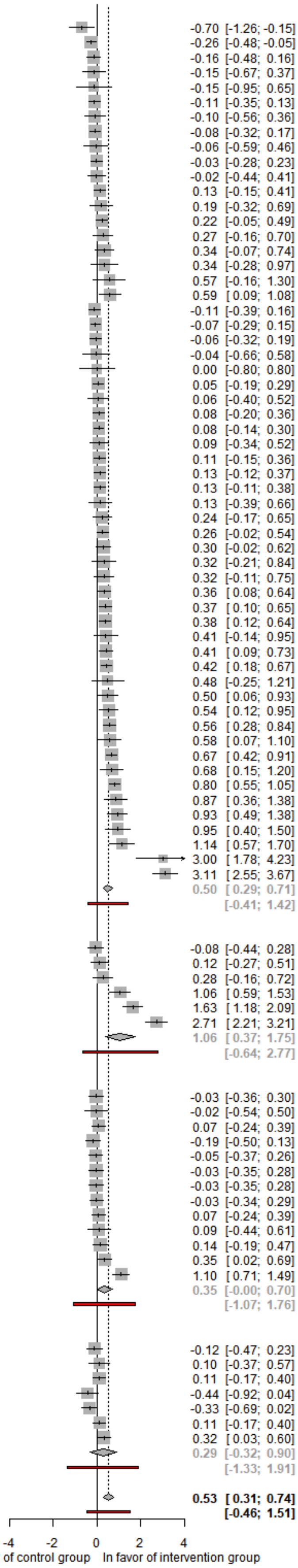

Figure S12.2. Subgroup analysis of the Emotional QoL. Forest plot represents the difference between the intervention vs. control group in the Emotional QoL domain with the cancer type subgroups as predicted at week 12 (post-intervention). SMD - Standardized mean difference, CI - confidence interval.

Figure S12.3.T24

Study	Experimental Patient N	Experimental Mean	Experimental SD	Control Patient N	Control Mean	Control SD	follow-up	Follow-up time	SMD	95%-CI
**breast**										
Seliniotaki, T, et al., 2021	27	56.10	29.10	26	74.20	20.90	baseline	0.00	-0.70	[-1.26; -0.15]
Reich, RR, et al. 2016	167	63.83	17.42	155	68.57	18.32	baseline	0.00	-0.26	[-0.48; -0.05]
Nápoles AM, et al. 2015	76	12.07	4.91	75	12.86	5.14	baseline	0.00	-0.16	[-0.48; 0.16]
Peng, L, et al., 2022	28	69.94	16.25	29	72.41	16.08	baseline	0.00	-0.15	[-0.67; 0.37]
Rahmani, S, et al. 2015	12	11.11	8.94	12	12.50	9.05	baseline	0.00	-0.15	[-0.95; 0.65]
Qiu, H, et al., 2018 II.	98	11.50	6.30	196	12.22	6.56	baseline	0.00	-0.11	[-0.35; 0.13]
Dirksen, S. et al, 2007	34	20.10	2.80	38	20.40	3.10	baseline	0.00	-0.10	[-0.56; 0.36]
Qiu, H, et al., 2018 I.	98	11.72	6.65	196	12.22	6.56	baseline	0.00	-0.08	[-0.32; 0.17]
Hernandez, EG, et al. 2018	28	13.07	5.26	28	13.39	4.94	baseline	0.00	-0.06	[-0.59; 0.46]
Klafke, N, et al., 2019	120	50.00	26.00	114	50.70	26.50	baseline	0.00	-0.03	[-0.28; 0.23]
Wu, Q, et al., 2021	43	62.34	6.76	43	62.45	6.63	baseline	0.00	-0.02	[-0.44; 0.41]
Klinkhammer-Schalke, M et al., 2012	100	51.86	29.03	100	48.14	28.29	baseline	0.00	0.13	[-0.15; 0.41]
Kim, YH, et al., 2017	30	56.40	16.50	30	53.30	16.50	baseline	0.00	0.19	[-0.32; 0.69]
Hoffman, CJ, et al., 2012	102	16.91	3.84	109	15.97	4.58	baseline	0.00	0.22	[-0.05; 0.49]
Arving, C, et al., 2007	47	69.00	25.00	38	63.00	18.00	baseline	0.00	0.27	[-0.16; 0.70]
Kim, SH, et al., 2021	47	72.00	17.70	47	65.40	21.00	baseline	0.00	0.34	[-0.07; 0.74]
Elyasi, F, et al., 2021 II.	20	10.60	3.10	20	9.60	2.60	baseline	0.00	0.34	[-0.28; 0.97]
Elyasi, F, et al., 2021 I.	15	11.50	3.80	15	9.60	2.60	baseline	0.00	0.57	[-0.16; 1.30]
Cengiz, HO, et al., 2023	32	12.90	5.40	33	9.75	5.22	baseline	0.00	0.59	[0.09; 1.08]
Klinkhammer-Schalke, M et al., 2012	100	55.34	28.29	100	58.56	28.29	followup	12.00	-0.11	[-0.39; 0.16]
Reich, RR, et al. 2016	167	71.33	19.41	155	72.71	19.13	followup	12.00	-0.07	[-0.29; 0.15]
Klafke, N, et al., 2019	120	62.80	22.30	114	64.20	22.40	followup	12.00	-0.06	[-0.32; 0.19]
Elyasi, F, et al., 2021 II.	20	9.40	2.10	20	9.50	2.60	followup	24.00	-0.04	[-0.66; 0.58]
Rahmani, S, et al. 2015	12	12.00	48.61	12	12.00	23.61	followup	16.00	0.00	[-0.80; 0.80]
Qiu, H, et al., 2018 II.	98	12.32	9.06	196	11.90	7.89	followup	4.00	0.05	[-0.19; 0.29]
Dirksen, S. et al, 2007	34	20.80	2.30	38	20.60	4.00	followup	10.00	0.06	[-0.40; 0.52]
Klinkhammer-Schalke, M et al., 2012	100	63.52	27.07	100	61.29	27.29	followup	48.00	0.08	[-0.20; 0.36]
Reich, RR, et al. 2016	167	70.87	18.02	155	69.36	18.78	followup	6.00	0.08	[-0.14; 0.30]
Arving, C, et al., 2007	47	76.00	24.00	38	74.00	20.00	followup	12.00	0.09	[-0.34; 0.52]
Klafke, N, et al., 2019	120	59.10	25.40	114	56.50	23.00	followup	24.00	0.11	[-0.15; 0.36]
Qiu, H, et al., 2018 II.	98	14.58	9.06	196	13.52	7.89	followup	24.00	0.13	[-0.12; 0.37]
Qiu, H, et al., 2018 II.	98	14.23	7.67	196	13.10	8.87	followup	12.00	0.13	[-0.11; 0.38]
Hernandez, EG, et al. 2018	28	14.00	4.69	28	13.32	5.32	followup	24.00	0.13	[-0.39; 0.66]
Kim, SH, et al., 2021	47	75.90	16.30	47	71.70	18.40	followup	20.00	0.24	[-0.17; 0.65]
Klinkhammer-Schalke, M et al., 2012	100	66.50	26.80	100	59.06	29.53	followup	36.00	0.26	[-0.02; 0.54]
Nápoles AM, et al. 2015	76	15.93	3.52	75	14.73	4.33	followup	12.00	0.30	[-0.02; 0.62]
Hernandez, EG, et al. 2018	28	16.12	4.09	28	14.72	4.63	followup	8.00	0.32	[-0.21; 0.84]
Arving, C, et al., 2007	47	82.00	20.00	38	75.00	24.00	followup	24.00	0.32	[-0.11; 0.75]
Klinkhammer-Schalke, M et al., 2012	100	66.25	25.31	100	56.58	28.54	followup	24.00	0.36	[0.08; 0.64]
Hoffman, CJ, et al., 2012	102	18.14	3.82	109	16.59	4.40	followup	8.00	0.37	[0.10; 0.65]
Klafke, N, et al., 2019	120	65.90	25.60	114	56.30	24.70	followup	48.00	0.38	[0.12; 0.64]
Seliniotaki, T, et al., 2021	27	74.50	24.50	26	61.70	36.70	followup	8.00	0.41	[-0.14; 0.95]
Nápoles AM, et al. 2015	76	16.39	3.30	75	14.89	3.95	followup	24.00	0.41	[0.09; 0.73]
Qiu, H, et al., 2018 I.	98	15.42	9.06	196	11.90	7.89	followup	4.00	0.42	[0.18; 0.67]
Elyasi, F, et al., 2021 I.	15	11.00	3.40	15	9.50	2.60	followup	24.00	0.48	[-0.25; 1.21]
Arving, C, et al., 2007	47	80.00	21.00	38	69.00	23.00	followup	4.00	0.50	[0.06; 0.93]
Kim, SH, et al., 2021	47	79.00	15.70	47	69.60	18.90	followup	8.00	0.54	[0.12; 0.95]
Hoffman, CJ, et al., 2012	102	18.59	3.75	109	16.28	4.42	followup	12.00	0.56	[0.28; 0.84]
Kim, YH, et al., 2017	30	67.80	15.10	30	56.70	21.90	followup	6.00	0.58	[0.07; 1.10]
Qiu, H, et al., 2018 I.	98	19.23	9.76	196	13.10	8.87	followup	12.00	0.67	[0.42; 0.91]
Kim, YH, et al., 2017	30	70.80	14.50	30	57.80	22.60	followup	9.00	0.68	[0.15; 1.20]
Qiu, H, et al., 2018 I.	98	20.00	8.37	196	13.52	7.89	followup	24.00	0.80	[0.55; 1.05]
Cengiz, HO, et al., 2023	32	13.56	5.78	33	8.75	5.14	followup	8.00	0.87	[0.36; 1.38]
Wu, Q, et al., 2021	43	81.54	8.21	43	74.21	7.33	followup	12.00	0.93	[0.49; 1.38]
Peng, L, et al., 2022	28	80.95	13.20	29	70.12	9.05	followup	4.00	0.95	[0.40; 1.50]
Peng, L, et al., 2022	28	83.33	14.87	29	69.25	8.94	followup	0.10	1.14	[0.57; 1.70]
Rahmani, S, et al. 2015	12	56.94	11.14	12	22.91	10.73	followup	8.00	3.00	[1.78; 4.23]
Henderson, VP, et al., 2013	53	18.00	0.40	58	16.90	0.30	followup	16.00	3.11	[2.55; 3.67]
Random effect	3817			4539					0.31	[0.08; 0.53]
Prediction interval										[-0.63; 1.25]
**gastroenterological**										
Fang, P, et al.,2020	60	46.14	5.12	60	46.55	5.23	baseline	0.00	-0.08	[-0.44; 0.28]
Qin, X, et al., 2017	50	73.61	10.68	50	72.41	9.34	baseline	0.00	0.12	[-0.27; 0.51]
Gao Q, et al. 2020	40	-56.85	4.66	40	-58.20	4.78	baseline	0.00	0.28	[-0.16; 0.72]
Gao Q, et al. 2020	40	-49.55	4.20	40	-53.56	3.25	followup	4.00	1.06	[0.59; 1.53]
Qin, X, et al., 2017	50	90.72	8.27	50	75.60	10.01	followup	2.00	1.63	[1.18; 2.09]
Fang, P, et al.,2020	60	63.52	6.47	60	47.56	5.14	followup	4.00	2.71	[2.21; 3.21]
Random effect	300			300					0.87	[0.33; 1.41]
Prediction interval										[-0.48; 2.23]
**gynaecological**										
Sandsund, C, et al., 2017	72	67.10	25.30	70	67.90	23.40	baseline	0.00	-0.03	[-0.36; 0.30]
Powell, CB, et al., 2008	21	16.10	4.20	43	16.20	5.00	baseline	0.00	-0.02	[-0.54; 0.50]
Chan, et al. 2005	80	58.10	45.50	75	54.60	48.48	baseline	0.00	0.07	[-0.24; 0.39]
Chan, et al. 2005	80	65.70	47.01	75	73.94	39.30	followup	12.00	-0.19	[-0.50; 0.13]
Chan, et al. 2005	80	75.77	73.38	75	79.44	64.08	followup	72.00	-0.05	[-0.37; 0.26]
Chan, et al. 2005	80	76.73	57.02	75	78.59	56.21	followup	48.00	-0.03	[-0.35; 0.28]
Chan, et al. 2005	80	73.39	67.76	75	75.30	55.80	followup	60.00	-0.03	[-0.35; 0.28]
Chan, et al. 2005	80	68.59	48.12	75	69.81	48.88	followup	24.00	-0.03	[-0.34; 0.29]
Chan, et al. 2005	80	77.37	58.41	75	73.44	52.28	followup	36.00	0.07	[-0.24; 0.39]
Powell, CB, et al., 2008	21	18.50	5.40	43	18.00	5.90	followup	12.00	0.09	[-0.44; 0.61]
Sandsund, C, et al., 2017	72	70.00	23.90	70	66.40	26.20	followup	12.00	0.14	[-0.19; 0.47]
Sandsund, C, et al., 2017	72	75.50	20.20	70	66.70	28.60	followup	24.00	0.35	[0.02; 0.69]
Zhou, J, et al., 2020	59	64.81	9.32	59	55.54	7.29	followup	2.00	1.10	[0.71; 1.49]
Random effect	877			880					0.15	[-0.15; 0.46]
Prediction interval										[-1.29; 1.60]
**prostate**										
Berglund, G. et al. 2007	39	81.20	18.90	150	83.60	19.70	baseline	0.00	-0.12	[-0.47; 0.23]
Penedo, FJ, et al., 2007	41	-19.24	4.04	30	-19.63	3.43	baseline	0.00	0.10	[-0.37; 0.57]
Penedo, FJ, et al., 2020	95	-20.03	3.80	97	-20.46	3.74	baseline	0.00	0.11	[-0.17; 0.40]
Penedo, FJ, et al., 2007	41	-20.73	3.31	30	-19.20	3.62	followup	12.00	-0.44	[-0.92; 0.04]
Berglund, G. et al. 2007	39	81.60	20.10	150	87.80	18.00	followup	48.00	-0.33	[-0.69; 0.02]
Penedo, FJ, et al., 2020	95	-20.18	3.80	97	-20.62	3.84	followup	24.00	0.11	[-0.17; 0.40]
Penedo, FJ, et al., 2020	95	-19.20	3.90	97	-20.43	3.84	followup	48.00	0.32	[0.03; 0.60]
Random effect	445			651					0.10	[-0.51; 0.70]
Prediction interval										[-1.59; 1.78]
**Random effect**	**5439**			**6370**					**0.30**	**[0.09; 0.51]**
**Prediction interval**										**[-0.72; 1.32]**

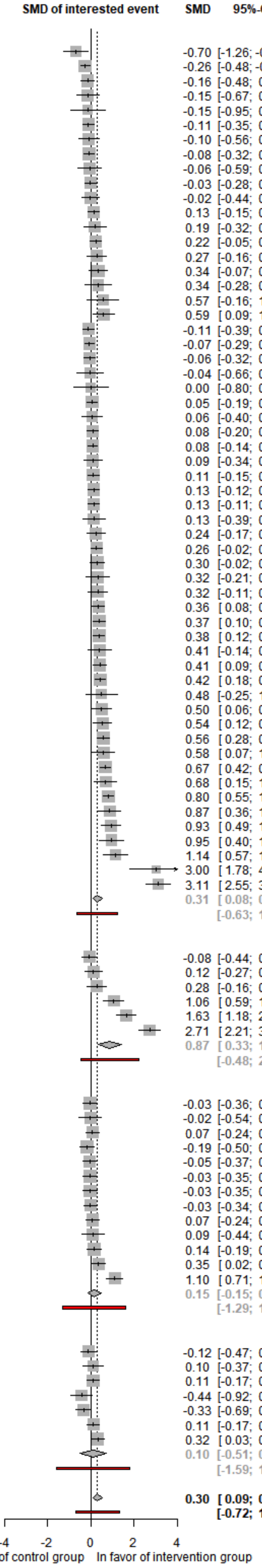

Figure S12.3.Subgroup analysis of the Emotional QoL. Forest plot represents the difference between the intervention vs. control group in the Emotional QoL domain with the cancer type subgroups as predicted at week 24 (post-intervention). SMD - Standardized mean difference, CI - confidence interval.

Figure S12.4.T48

Study	Experimental Patient N	Experimental Mean	Experimental SD	Control Patient N	Control Mean	Control SD	follow-up	Follow-up time	SMD	95%-CI
breast										
Seliniotaki, T, et al., 2021	27	56.10	29.10	26	74.20	20.90	baseline	0.00	-0.70	[-1.26; -0.15]
Reich, RR, et al. 2016	167	63.83	17.42	155	68.57	18.32	baseline	0.00	-0.26	[-0.48; -0.05]
Nápoles AM, et al. 2015	76	12.07	4.91	75	12.86	5.14	baseline	0.00	-0.16	[-0.48; 0.16]
Peng, L, et al., 2022	28	69.94	16.25	29	72.41	16.08	baseline	0.00	-0.15	[-0.67; 0.37]
Rahmani, S, et al. 2015	12	11.11	8.94	12	12.50	9.05	baseline	0.00	-0.15	[-0.95; 0.65]
Qiu, H, et al., 2018 II.	98	11.50	6.30	196	12.22	6.56	baseline	0.00	-0.11	[-0.35; 0.13]
Dirksen, S. et al, 2007	34	20.10	2.80	38	20.40	3.10	baseline	0.00	-0.10	[-0.56; 0.36]
Qiu, H, et al., 2018 I.	98	11.72	6.65	196	12.22	6.56	baseline	0.00	-0.08	[-0.32; 0.17]
Hernandez, EG, et al. 2018	28	13.07	5.26	28	13.39	4.94	baseline	0.00	-0.06	[-0.59; 0.46]
Klafke, N, et al., 2019	120	50.00	26.00	114	50.70	26.50	baseline	0.00	-0.03	[-0.28; 0.23]
Wu, Q, et al., 2021	43	62.34	6.76	43	62.45	6.63	baseline	0.00	-0.02	[-0.44; 0.41]
Klinkhammer-Schalke, M et al., 2012	100	51.86	29.03	100	48.14	28.29	baseline	0.00	0.13	[-0.15; 0.41]
Kim, YH, et al., 2017	30	56.40	16.50	30	53.30	16.50	baseline	0.00	0.19	[-0.32; 0.69]
Hoffman, CJ, et al., 2012	102	16.91	3.84	109	15.97	4.58	baseline	0.00	0.22	[-0.05; 0.49]
Arving, C, et al., 2007	47	69.00	25.00	38	63.00	18.00	baseline	0.00	0.27	[-0.16; 0.70]
Kim, SH, et al., 2021	47	72.00	17.70	47	65.40	21.00	baseline	0.00	0.34	[-0.07; 0.74]
Elyasi, F, et al., 2021 II.	20	10.60	3.10	20	9.60	2.60	baseline	0.00	0.34	[-0.28; 0.97]
Elyasi, F, et al., 2021 I.	15	11.50	3.80	15	9.60	2.60	baseline	0.00	0.57	[-0.16; 1.30]
Cengiz, HO, et al., 2023	32	12.90	5.40	33	9.75	5.22	baseline	0.00	0.59	[0.09; 1.08]
Klinkhammer-Schalke, M et al., 2012	100	55.34	28.29	100	58.56	28.29	followup	12.00	-0.11	[-0.39; 0.16]
Reich, RR, et al. 2016	167	71.33	19.41	155	72.71	19.13	followup	12.00	-0.07	[-0.29; 0.15]
Klafke, N, et al., 2019	120	62.80	22.30	114	64.20	22.40	followup	12.00	-0.06	[-0.32; 0.19]
Elyasi, F, et al., 2021 II.	20	9.40	2.10	20	9.50	2.60	followup	24.00	-0.04	[-0.66; 0.58]
Rahmani, S, et al. 2015	12	12.00	48.61	12	12.00	23.61	followup	16.00	0.00	[-0.80; 0.80]
Qiu, H, et al., 2018 II.	98	12.32	9.06	196	11.90	7.89	followup	4.00	0.05	[-0.19; 0.29]
Dirksen, S. et al, 2007	34	20.80	2.30	38	20.60	4.00	followup	10.00	0.06	[-0.40; 0.52]
Klinkhammer-Schalke, M et al., 2012	100	63.52	27.07	100	61.29	27.29	followup	48.00	0.08	[-0.20; 0.36]
Reich, RR, et al. 2016	167	70.87	18.02	155	69.36	18.78	followup	6.00	0.08	[-0.14; 0.30]
Arving, C, et al., 2007	47	76.00	24.00	38	74.00	20.00	followup	12.00	0.09	[-0.34; 0.52]
Klafke, N, et al., 2019	120	59.10	25.40	114	56.50	23.00	followup	24.00	0.11	[-0.15; 0.36]
Qiu, H, et al., 2018 II.	98	14.58	9.06	196	13.52	7.89	followup	24.00	0.13	[-0.12; 0.37]
Qiu, H, et al., 2018 II.	98	14.23	7.67	196	13.10	8.87	followup	12.00	0.13	[-0.11; 0.38]
Hernandez, EG, et al. 2018	28	14.00	4.69	28	13.32	5.32	followup	24.00	0.13	[-0.39; 0.66]
Kim, SH, et al., 2021	47	75.90	16.30	47	71.70	18.40	followup	20.00	0.24	[-0.17; 0.65]
Klinkhammer-Schalke, M et al., 2012	100	66.50	26.80	100	59.06	29.53	followup	36.00	0.26	[-0.02; 0.54]
Nápoles AM, et al. 2015	76	15.93	3.52	75	14.73	4.33	followup	12.00	0.30	[-0.02; 0.62]
Hernandez, EG, et al. 2018	28	16.12	4.09	28	14.72	4.63	followup	8.00	0.32	[-0.21; 0.84]
Arving, C, et al., 2007	47	82.00	20.00	38	75.00	24.00	followup	24.00	0.32	[-0.11; 0.75]
Klinkhammer-Schalke, M et al., 2012	100	66.25	25.31	100	56.58	28.54	followup	24.00	0.36	[0.08; 0.64]
Hoffman, CJ, et al., 2012	102	18.14	3.82	109	16.59	4.40	followup	8.00	0.37	[0.10; 0.65]
Klafke, N, et al., 2019	120	65.90	25.60	114	56.30	24.70	followup	48.00	0.38	[0.12; 0.64]
Seliniotaki, T, et al., 2021	27	74.50	24.50	26	61.70	36.70	followup	8.00	0.41	[-0.14; 0.95]
Nápoles AM, et al. 2015	76	16.39	3.30	75	14.89	3.95	followup	24.00	0.41	[0.09; 0.73]
Qiu, H, et al., 2018 I.	98	15.42	9.06	196	11.90	7.89	followup	4.00	0.42	[0.18; 0.67]
Elyasi, F, et al., 2021 I.	15	11.00	3.40	15	9.50	2.60	followup	24.00	0.48	[-0.25; 1.21]
Arving, C, et al., 2007	47	80.00	21.00	38	69.00	23.00	followup	4.00	0.50	[0.06; 0.93]
Kim, SH, et al., 2021	47	79.00	15.70	47	69.60	18.90	followup	8.00	0.54	[0.12; 0.95]
Hoffman, CJ, et al., 2012	102	18.59	3.75	109	16.28	4.42	followup	12.00	0.56	[0.28; 0.84]
Kim, YH, et al., 2017	30	67.80	15.10	30	56.70	21.90	followup	6.00	0.58	[0.07; 1.10]
Qiu, H, et al., 2018 I.	98	19.23	9.76	196	13.10	8.87	followup	12.00	0.67	[0.42; 0.91]
Kim, YH, et al., 2017	30	70.80	14.50	30	57.80	22.60	followup	9.00	0.68	[0.15; 1.20]
Qiu, H, et al., 2018 I.	98	20.00	8.37	196	13.52	7.89	followup	24.00	0.80	[0.55; 1.05]
Cengiz, HO, et al., 2023	32	13.56	5.78	33	8.75	5.14	followup	8.00	0.87	[0.36; 1.38]
Wu, Q, et al., 2021	43	81.54	8.21	43	74.21	7.33	followup	12.00	0.93	[0.49; 1.38]
Peng, L, et al., 2022	28	80.95	13.20	29	70.12	9.05	followup	4.00	0.95	[0.40; 1.50]
Peng, L, et al., 2022	28	83.33	14.87	29	69.25	8.94	followup	0.10	1.14	[0.57; 1.70]
Rahmani, S, et al. 2015	12	56.94	11.14	12	22.91	10.73	followup	8.00	3.00	[1.78; 4.23]
Henderson, VP, et al., 2013	53	18.00	0.40	58	16.90	0.30	followup	16.00	3.11	[2.55; 3.67]
Random effect	3817			4539					0.17	[-0.12; 0.46]
Prediction interval										[-0.91; 1.25]
gastroenterological										
Fang, P, et al.,2020	60	46.14	5.12	60	46.55	5.23	baseline	0.00	-0.08	[-0.44; 0.28]
Qin, X, et al., 2017	50	73.61	10.68	50	72.41	9.34	baseline	0.00	0.12	[-0.27; 0.51]
Gao Q, et al. 2020	40	-56.85	4.66	40	-58.20	4.78	baseline	0.00	0.28	[-0.16; 0.72]
Gao Q, et al. 2020	40	-49.55	4.20	40	-53.56	3.25	followup	4.00	1.06	[0.59; 1.53]
Qin, X, et al., 2017	50	90.72	8.27	50	75.60	10.01	followup	2.00	1.63	[1.18; 2.09]
Fang, P, et al.,2020	60	63.52	6.47	60	47.56	5.14	followup	4.00	2.71	[2.21; 3.21]
Random effect	300			300					0.73	[0.23; 1.24]
Prediction interval										[-0.48; 1.95]
gynaecological										
Sandsund, C, et al., 2017	72	67.10	25.30	70	67.90	23.40	baseline	0.00	-0.03	[-0.36; 0.30]
Powell, CB, et al., 2008	21	16.10	4.20	43	16.20	5.00	baseline	0.00	-0.02	[-0.54; 0.50]
Chan, et al. 2005	80	58.10	45.50	75	54.60	48.48	baseline	0.00	0.07	[-0.24; 0.39]
Chan, et al. 2005	80	65.70	47.01	75	73.94	39.30	followup	12.00	-0.19	[-0.50; 0.13]
Chan, et al. 2005	80	75.77	73.38	75	79.44	64.08	followup	72.00	-0.05	[-0.37; 0.26]
Chan, et al. 2005	80	76.73	57.02	75	78.59	56.21	followup	48.00	-0.03	[-0.35; 0.28]
Chan, et al. 2005	80	73.39	67.76	75	75.30	55.80	followup	60.00	-0.03	[-0.35; 0.28]
Chan, et al. 2005	80	68.59	48.12	75	69.81	48.88	followup	24.00	-0.03	[-0.34; 0.29]
Chan, et al. 2005	80	77.37	58.41	75	73.44	52.28	followup	36.00	0.07	[-0.24; 0.39]
Powell, CB, et al., 2008	21	18.50	5.40	43	18.00	5.90	followup	12.00	0.09	[-0.44; 0.61]
Sandsund, C, et al., 2017	72	70.00	23.90	70	66.40	26.20	followup	12.00	0.14	[-0.19; 0.47]
Sandsund, C, et al., 2017	72	75.50	20.20	70	66.70	28.60	followup	24.00	0.35	[0.02; 0.69]
Zhou, J, et al., 2020	59	64.81	9.32	59	55.54	7.29	followup	2.00	1.10	[0.71; 1.49]
Random effect	877			880					0.02	[-0.46; 0.49]
Prediction interval										[-1.47; 1.50]
prostate										
Berglund, G. et al. 2007	39	81.20	18.90	150	83.60	19.70	baseline	0.00	-0.12	[-0.47; 0.23]
Penedo, FJ, et al., 2007	41	-19.24	4.04	30	-19.63	3.43	baseline	0.00	0.10	[-0.37; 0.57]
Penedo, FJ, et al., 2020	95	-20.03	3.80	97	-20.46	3.74	baseline	0.00	0.11	[-0.17; 0.40]
Penedo, FJ, et al., 2007	41	-20.73	3.31	30	-19.20	3.62	followup	12.00	-0.44	[-0.92; 0.04]
Berglund, G. et al. 2007	39	81.60	20.10	150	87.80	18.00	followup	48.00	-0.33	[-0.69; 0.02]
Penedo, FJ, et al., 2020	95	-20.18	3.80	97	-20.62	3.84	followup	24.00	0.11	[-0.17; 0.40]
Penedo, FJ, et al., 2020	95	-19.20	3.90	97	-20.43	3.84	followup	48.00	0.32	[0.03; 0.60]
Random effect	445			651					-0.04	[-0.65; 0.56]
Prediction interval										[-1.61; 1.53]
**Random effect**	**5439**			**6370**					**0.15**	**[-0.18; 0.47]**
**Prediction interval**										**[-1.07; 1.37]**

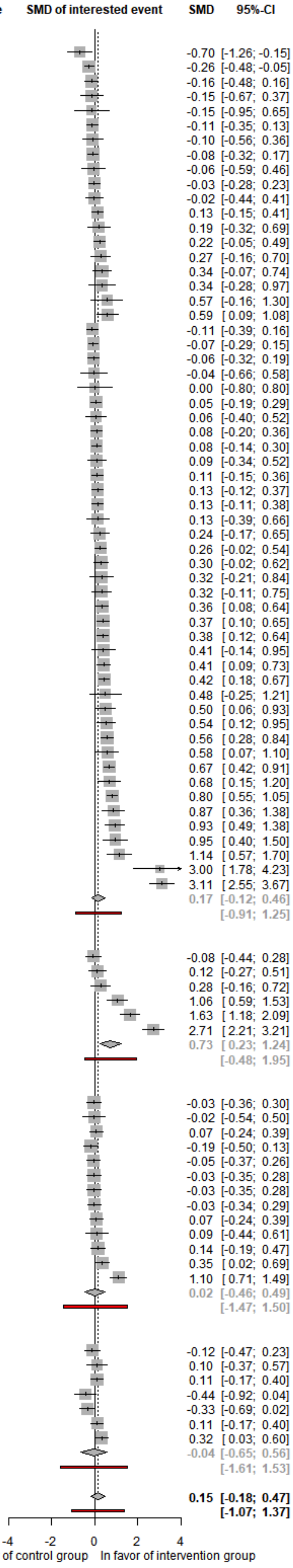

Figure S12.4. Subgroup analysis of the Emotional QoL. Forest plot represents the difference between the intervention vs. control group in the Emotional QoL domain with the cancer type subgroups as predicted at week 48 (post-intervention). SMD - Standardized mean difference, CI - confidence interval.

S13.Subgroup analysis of Social QoL: Provider

Figure S13.1.T0

Study	Experimental Patient N	Experimental Mean	Experimental SD	Control Patient N	Control Mean	Control SD	follow-up	Follow-up time	SMD	95%-CI
psychologist										
Rodríguez Vega, B, et al., 2010	39	3.31	3.32	33	6.08	3.62	baseline	0.00	-0.79	[-1.27; -0.31]
Peng, L, et al., 2022	28	76.79	15.27	29	81.61	16.87	baseline	0.00	-0.30	[-0.82; 0.23]
Serfaty M, et al., 2018	20	16.40	6.10	22	18.10	7.80	baseline	0.00	-0.24	[-0.84; 0.37]
Qiu, H, et al., 2018 II.	98	17.84	5.10	196	18.85	4.22	baseline	0.00	-0.22	[-0.47; 0.02]
Heiney, SP, et al., 2003	33	5.60	2.00	33	5.90	1.90	baseline	0.00	-0.15	[-0.64; 0.33]
Qiu, H, et al., 2018 I.	98	18.13	5.70	196	18.85	4.22	baseline	0.00	-0.15	[-0.39; 0.09]
Ruiz-Vozmediano, J, et al.,2020	31	69.40	29.80	32	73.40	28.40	baseline	0.00	-0.14	[-0.63; 0.36]
Seliniotaki, T, et al., 2021	27	74.40	29.90	26	77.70	29.30	baseline	0.00	-0.11	[-0.65; 0.43]
Antoni, MH, et al., 2006	92	877.61	158.55	107	891.17	152.16	baseline	0.00	-0.09	[-0.37; 0.19]
Hernandez, EG, et al. 2018	28	17.11	6.05	28	17.56	5.46	baseline	0.00	-0.08	[-0.60; 0.45]
Liu, T, et al., 2019	49	63.77	24.95	53	65.22	23.71	baseline	0.00	-0.06	[-0.45; 0.33]
Chan, et al. 2005	80	54.76	60.02	75	56.28	54.76	baseline	0.00	-0.03	[-0.34; 0.29]
Johansson, B, et al., 2008	128	84.00	24.00	116	82.00	25.00	baseline	0.00	0.08	[-0.17; 0.33]
Klinkhammer-Schalke, M et al., 2012	100	71.21	33.50	100	67.99	34.25	baseline	0.00	0.09	[-0.18; 0.37]
Arving, C, et al., 2007	47	78.00	27.00	38	75.00	19.00	baseline	0.00	0.13	[-0.30; 0.55]
Rahmani, S, et al. 2015	12	34.72	13.22	12	33.33	7.11	baseline	0.00	0.13	[-0.67; 0.93]
Rodrigez, B, et al., 2014	8	95.83	11.79	7	90.48	25.20	baseline	0.00	0.26	[-0.76; 1.28]
Penedo, FJ, et al., 2020	95	-19.85	4.09	97	-21.16	4.24	baseline	0.00	0.31	[0.03; 0.60]
Powell, CB, et al., 2008	21	22.20	5.40	43	19.70	6.00	baseline	0.00	0.42	[-0.10; 0.95]
Lu, Z, et al., 201	203	75.30	4.00	103	71.30	6.50	baseline	0.00	0.80	[0.56; 1.05]
Antoni, MH, et al., 2006	92	788.15	133.08	107	855.60	126.82	followup	48.00	-0.52	[-0.80; -0.23]
Heiney, SP, et al., 2003	33	5.80	2.00	33	6.55	1.80	followup	16.00	-0.39	[-0.88; 0.10]
Antoni, MH, et al., 2006	92	832.88	119.31	107	873.38	114.05	followup	24.00	-0.35	[-0.63; -0.07]
Chan, et al. 2005	80	76.19	54.76	75	87.23	54.76	followup	72.00	-0.20	[-0.52; 0.12]
Chan, et al. 2005	80	65.58	54.76	75	75.11	54.76	followup	24.00	-0.17	[-0.49; 0.14]
Chan, et al. 2005	80	76.62	54.76	75	85.06	54.76	followup	60.00	-0.15	[-0.47; 0.16]
Chan, et al. 2005	80	61.47	54.76	75	69.26	54.76	followup	12.00	-0.14	[-0.46; 0.17]
Serfaty M, et al., 2018	20	18.70	5.30	22	19.40	6.20	followup	12.00	-0.12	[-0.72; 0.49]
Peng, L, et al., 2022	28	80.36	20.09	29	82.18	19.38	followup	0.10	-0.09	[-0.61; 0.43]
Klinkhammer-Schalke, M et al., 2012	100	78.90	27.54	100	81.14	24.07	followup	48.00	-0.09	[-0.36; 0.19]
Rodrigez, B, et al., 2014	8	93.33	14.91	7	94.44	9.62	followup	2.00	-0.08	[-1.10; 0.93]
Chan, et al. 2005	80	80.52	54.76	75	83.77	54.76	followup	48.00	-0.06	[-0.37; 0.26]
Heiney, SP, et al., 2003	33	5.80	2.00	33	5.90	1.90	followup	6.00	-0.05	[-0.53; 0.43]
Peng, L, et al., 2022	28	79.17	16.49	29	79.89	20.11	followup	4.00	-0.04	[-0.56; 0.48]
Klinkhammer-Schalke, M et al., 2012	100	66.00	33.00	100	66.74	31.76	followup	12.00	-0.02	[-0.30; 0.25]
Qiu, H, et al., 2018 II.	98	17.98	8.92	196	17.98	8.52	followup	4.00	0.00	[-0.24; 0.24]
Rahmani, S, et al. 2015	12	12.00	54.16	12	12.00	34.72	followup	16.00	0.00	[-0.80; 0.80]
Seliniotaki, T, et al., 2021	27	74.60	35.90	26	74.60	29.30	followup	8.00	0.00	[-0.54; 0.54]
Qiu, H, et al., 2018 II.	98	18.97	8.92	196	18.75	9.46	followup	24.00	0.02	[-0.22; 0.27]
Klinkhammer-Schalke, M et al., 2012	100	72.20	28.78	100	71.46	29.03	followup	24.00	0.03	[-0.25; 0.30]
Arving, C, et al., 2007	47	76.00	25.00	38	75.00	19.00	followup	12.00	0.04	[-0.38; 0.47]
Johansson, B, et al., 2008	128	91.00	18.00	116	90.00	20.00	followup	96.00	0.05	[-0.20; 0.30]
Chan, et al. 2005	80	80.95	54.76	75	77.92	54.76	followup	36.00	0.06	[-0.26; 0.37]
Johansson, B, et al., 2008	128	92.00	15.00	116	91.00	18.00	followup	48.00	0.06	[-0.19; 0.31]
Qiu, H, et al., 2018 II.	98	18.77	8.03	196	18.25	5.05	followup	12.00	0.08	[-0.16; 0.33]
Arving, C, et al., 2007	47	81.00	22.00	38	79.00	25.00	followup	4.00	0.08	[-0.34; 0.51]
Arving, C, et al., 2007	47	83.00	21.00	38	80.00	26.00	followup	24.00	0.13	[-0.30; 0.56]
Klinkhammer-Schalke, M et al., 2012	100	77.91	27.05	100	73.69	30.77	followup	36.00	0.15	[-0.13; 0.42]
Ruiz-Vozmediano, J, et al.,2020	31	83.30	24.70	32	78.70	28.50	followup	24.00	0.17	[-0.32; 0.67]
Johansson, B, et al., 2008	128	90.00	17.00	116	86.00	22.00	followup	24.00	0.20	[-0.05; 0.46]
Johansson, B, et al., 2008	128	87.00	20.00	116	82.00	24.00	followup	12.00	0.23	[-0.03; 0.48]
Qiu, H, et al., 2018 I.	98	20.01	9.15	196	17.98	8.52	followup	4.00	0.23	[-0.01; 0.47]
Serfaty M, et al., 2018	20	21.20	4.30	22	19.70	6.70	followup	18.00	0.26	[-0.35; 0.87]
Rodrigez, B, et al., 2014	8	95.83	11.79	7	90.48	25.20	followup	0.10	0.26	[-0.76; 1.28]
Penedo, FJ, et al., 2020	95	-20.74	4.29	97	-22.05	4.43	followup	48.00	0.30	[0.01; 0.58]
Penedo, FJ, et al., 2020	95	-20.61	4.19	97	-21.91	4.33	followup	24.00	0.30	[0.02; 0.59]
Powell, CB, et al., 2008	21	21.30	5.60	43	19.10	7.30	followup	12.00	0.32	[-0.21; 0.84]
Qiu, H, et al., 2018 I.	98	21.75	8.70	196	18.75	9.46	followup	24.00	0.32	[0.08; 0.57]
Hernandez, EG, et al. 2018	28	18.82	5.79	28	16.40	5.07	followup	24.00	0.44	[-0.09; 0.97]
Serfaty M, et al., 2018	20	19.50	7.20	22	16.00	7.60	followup	24.00	0.46	[-0.15; 1.08]
Hernandez, EG, et al. 2018	28	19.23	5.51	28	16.60	5.39	followup	8.00	0.48	[-0.06; 1.01]
Qiu, H, et al., 2018 I.	98	21.39	8.03	196	18.25	5.05	followup	12.00	0.50	[0.26; 0.75]
Liu, T, et al., 2019	49	65.81	18.49	53	56.11	17.08	followup	9.00	0.54	[0.15; 0.94]
Liu, T, et al., 2019	49	74.35	17.68	53	62.05	14.34	followup	12.00	0.76	[0.36; 1.16]
Lu, Z, et al., 201	203	76.10	5.20	103	71.10	8.20	followup	9.00	0.78	[0.54; 1.03]
Rahmani, S, et al. 2015	12	51.38	11.14	12	29.16	16.09	followup	8.00	1.55	[0.62; 2.48]
Rodríguez Vega, B, et al., 2010	39	49.04	3.32	33	29.42	3.62	followup	12.00	> 5.61	[4.56; 6.66]
Rodríguez Vega, B, et al., 2010	39	61.86	3.32	33	34.47	3.62	followup	24.00	> 7.83	[6.44; 9.22]
Random effect	4468			5018					0.32	[0.06; 0.58]
Prediction interval										[-0.92; 1.56]
healthcare professional										
Ferguson, RJ, et al., 2012	19	6.26	2.05	21	7.31	1.21	baseline	0.00	-0.62	[-1.26; 0.02]
Hoffman, CJ, et al., 2012	102	17.59	5.91	109	18.78	6.01	baseline	0.00	-0.20	[-0.47; 0.07]
Berglund, G. et al. 2007	39	80.30	22.60	150	84.00	22.10	baseline	0.00	-0.17	[-0.52; 0.19]
Braeken APB, et al. 2013	136	84.94	21.44	144	85.46	22.52	baseline	0.00	-0.02	[-0.26; 0.21]
Schofield, P, et al., 2013	55	70.90	26.77	53	70.75	26.72	baseline	0.00	0.01	[-0.37; 0.38]
Girgis, A, et al., 2009 II.	120	80.80	25.60	117	79.50	25.10	baseline	0.00	0.05	[-0.20; 0.31]
Elyasi, F, et al., 2021 I.	15	2.00	1.20	15	1.90	0.90	baseline	0.00	0.09	[-0.62; 0.81]
Yun et al., 2017	134	75.80	26.80	72	73.10	23.40	baseline	0.00	0.10	[-0.18; 0.39]
Girgis, A, et al., 2009 I.	110	82.10	24.00	117	79.50	25.10	baseline	0.00	0.11	[-0.16; 0.37]
Guo, Z, et al., 2013	89	75.00	13.82	89	73.43	12.31	baseline	0.00	0.12	[-0.17; 0.41]
Sandsund, C, et al., 2017	72	74.10	26.70	70	70.00	29.00	baseline	0.00	0.15	[-0.18; 0.48]
Cheung YL, et al. 2002	29	11.00	0.70	30	10.16	2.26	baseline	0.00	0.49	[-0.03; 1.01]
Trask, PC, et al. 2003	25	71.50	22.00	23	58.20	29.25	baseline	0.00	0.51	[-0.07; 1.08]
Elyasi, F, et al., 2021 II.	20	2.50	1.10	20	1.90	0.90	baseline	0.00	0.59	[-0.05; 1.22]
Beatty, L. et al. 2015	30	57.26	5.49	30	66.56	5.41	followup	6.00	-1.68	[-2.28; -1.09]
Ferguson, RJ, et al., 2012	19	6.75	1.78	21	7.47	1.59	followup	8.00	-0.42	[-1.05; 0.21]
Berglund, G. et al. 2007	39	77.00	23.70	150	83.50	23.70	followup	48.00	-0.27	[-0.63; 0.08]
Schofield, P, et al., 2013	55	65.03	28.36	53	71.29	28.69	followup	12.00	-0.22	[-0.60; 0.16]
Girgis, A, et al., 2009 I.	110	88.20	19.30	117	89.90	17.00	followup	12.00	-0.09	[-0.35; 0.17]
Schofield, P, et al., 2013	55	68.28	26.22	53	70.58	26.38	followup	8.00	-0.09	[-0.46; 0.29]
Girgis, A, et al., 2009 II.	120	89.20	20.30	117	89.90	17.00	followup	12.00	-0.04	[-0.29; 0.22]
Hoffman, CJ, et al., 2012	102	18.09	5.81	109	18.30	5.75	followup	12.00	-0.04	[-0.31; 0.23]
Braeken APB, et al. 2013	136	86.99	20.73	144	87.55	19.10	followup	48.00	-0.03	[-0.26; 0.21]
Elyasi, F, et al., 2021 I.	15	1.80	0.90	15	1.80	0.90	followup	24.00	0.00	[-0.72; 0.72]
Girgis, A, et al., 2009 II.	120	91.90	17.60	117	91.90	17.40	followup	24.00	0.00	[-0.25; 0.25]
Hoffman, CJ, et al., 2012	102	18.36	5.65	109	18.26	5.88	followup	8.00	0.02	[-0.25; 0.29]
Girgis, A, et al., 2009 I.	110	92.20	15.00	117	91.90	17.40	followup	24.00	0.02	[-0.24; 0.28]
Braeken APB, et al. 2013	136	83.46	23.57	144	81.81	22.37	followup	12.00	0.07	[-0.16; 0.31]
Guan, S, et al., 2019	50	69.05	7.24	50	68.19	8.05	followup	8.00	0.11	[-0.28; 0.50]
Guo, Z, et al., 2013	89	74.35	10.67	89	71.33	11.88	followup	2.00	0.27	[-0.03; 0.56]
Trask, PC, et al. 2003	25	86.10	23.50	23	79.40	20.62	followup	24.00	0.30	[-0.27; 0.87]
Yun et al., 2017	134	85.30	19.50	72	78.20	22.40	followup	48.00	0.34	[0.06; 0.63]
Sandsund, C, et al., 2017	72	85.00	21.40	70	74.90	29.80	followup	12.00	0.39	[0.06; 0.72]
Sandsund, C, et al., 2017	72	86.00	18.90	70	74.20	28.90	followup	24.00	0.48	[0.15; 0.82]
Beatty, L. et al. 2015	30	77.87	4.62	30	75.08	4.61	followup	13.03	0.60	[0.08; 1.11]
Trask, PC, et al. 2003	25	85.90	24.00	23	70.10	27.34	followup	8.00	0.61	[0.03; 1.19]
Elyasi, F, et al., 2021 II.	20	2.40	1.00	20	1.80	0.90	followup	24.00	0.62	[-0.02; 1.25]
Cheung YL, et al. 2002	29	10.55	0.50	30	9.64	1.56	followup	5.00	0.77	[0.24; 1.30]
Cheung YL, et al. 2002	29	10.37	0.49	30	9.47	1.43	followup	10.00	0.83	[0.29; 1.36]
Beatty, L. et al. 2015	30	87.98	4.26	30	83.84	4.75	followup	26.07	0.91	[0.37; 1.44]
Random effect	2719			2863					0.22	[-0.04; 0.48]
Prediction interval										[-1.02; 1.46]
nurse										
Walczak, A, et al.,2017	61	21.16	4.48	49	22.71	4.99	baseline	0.00	-0.33	[-0.71; 0.05]
Chen, et al., 2017	58	58.30	6.20	65	59.60	6.90	baseline	0.00	-0.20	[-0.55; 0.16]
Fang, P, et al.,2020	60	48.50	4.11	60	49.08	5.01	baseline	0.00	-0.13	[-0.48; 0.23]
Wu, Q, et al., 2021	43	53.77	5.51	43	54.34	5.53	baseline	0.00	-0.10	[-0.53; 0.32]
McLachlan, SA, et al., 2001	296	41.20	22.60	154	42.80	18.70	baseline	0.00	-0.07	[-0.27; 0.12]
Li, X, et al., 2017	102	13.58	2.23	108	13.67	2.21	baseline	0.00	-0.04	[-0.31; 0.23]
Kim, SH, et al., 2021	47	73.70	81.90	47	75.50	22.00	baseline	0.00	-0.03	[-0.43; 0.37]
Dirksen, S. et al, 2007	34	22.10	4.90	38	22.20	6.10	baseline	0.00	-0.02	[-0.48; 0.44]
van der Meulen, IC, et al., 2013	88	83.10	2.50	91	83.10	2.50	baseline	0.00	0.00	[-0.29; 0.29]
Klafke, N, et al., 2019	120	59.30	31.30	113	58.60	29.60	baseline	0.00	0.02	[-0.23; 0.28]
Qin, X, et al., 2017	50	77.68	15.31	50	77.26	16.04	baseline	0.00	0.03	[-0.37; 0.42]
Baoyindeligeer, L.Z. et al. 2020	65	66.59	14.95	65	65.59	15.24	baseline	0.00	0.07	[-0.28; 0.41]
Zhao,X, et al. 2015	62	7.01	2.18	62	6.76	2.39	baseline	0.00	0.11	[-0.24; 0.46]
Thomas, ML, et al., 2012	64	21.10	5.40	88	19.00	6.30	baseline	0.00	0.35	[0.03; 0.68]
Kim, YH, et al., 2017	30	72.80	25.30	30	62.80	20.80	baseline	0.00	0.43	[-0.09; 0.94]
Zhao, X, et al., 2021	52	58.02	3.41	51	54.01	4.12	baseline	0.00	1.05	[0.64; 1.47]
McLachlan, SA, et al., 2001	296	42.00	23.70	154	47.50	17.90	followup	24.00	-0.25	[-0.45; -0.06]
Walczak, A, et al.,2017	61	20.94	5.40	49	21.43	4.47	followup	4.00	-0.10	[-0.47; 0.28]
Klafke, N, et al., 2019	120	54.50	29.60	113	55.10	29.50	followup	24.00	-0.02	[-0.28; 0.24]
Kim, SH, et al., 2021	47	80.30	17.60	47	80.40	18.50	followup	20.00	-0.01	[-0.41; 0.40]
Qin, X, et al., 2017	50	80.24	13.46	50	79.67	13.13	followup	2.00	0.04	[-0.35; 0.43]
Klafke, N, et al., 2019	120	62.10	29.40	113	59.70	27.60	followup	12.00	0.08	[-0.17; 0.34]
Zhao, X, et al., 2021	52	58.62	6.31	51	58.02	3.21	followup	12.00	0.12	[-0.27; 0.51]
Klafke, N, et al., 2019	120	69.10	27.90	113	63.60	26.60	followup	48.00	0.20	[-0.06; 0.46]
Thomas, ML, et al., 2012	64	20.50	6.10	88	19.00	6.40	followup	12.00	0.24	[-0.09; 0.56]
Li, X, et al., 2017	102	15.45	2.67	108	14.52	3.28	followup	12.00	0.31	[0.04; 0.58]
Kim, SH, et al., 2021	47	81.90	15.20	47	74.60	23.00	followup	8.00	0.37	[-0.04; 0.78]
Dirksen, S. et al, 2007	34	23.30	3.90	38	21.40	5.90	followup	10.00	0.37	[-0.10; 0.84]
Zhou, J, et al., 2020	59	72.34	11.43	59	65.87	10.26	followup	2.00	0.59	[0.22; 0.96]
Zhao,X, et al. 2015	62	8.19	2.10	62	6.87	2.21	followup	0.30	0.61	[0.25; 0.97]
Kim, YH, et al., 2017	30	75.60	26.20	30	58.30	27.20	followup	6.00	0.64	[0.12; 1.16]
Chen, et al., 2017	58	47.60	7.50	65	42.60	7.20	followup	8.00	0.68	[0.31; 1.04]
Chen, et al., 2017	58	46.80	7.60	65	41.50	7.50	followup	24.00	0.70	[0.33; 1.06]
Kim, YH, et al., 2017	30	75.00	23.10	30	54.40	28.00	followup	9.00	0.79	[0.27; 1.32]
van der Meulen, IC, et al., 2013	88	88.50	3.20	91	85.10	3.00	followup	48.00	1.09	[0.78; 1.41]
Wu, Q, et al., 2021	43	81.65	8.30	43	72.36	7.51	followup	12.00	1.16	[0.70; 1.62]
Baoyindeligeer, L.Z. et al. 2020	65	88.33	12.38	65	71.36	13.84	followup	2.00	1.28	[0.91; 1.66]
Fang, P, et al.,2020	60	65.47	6.98	60	49.45	5.65	followup	4.00	2.51	[2.03; 2.99]
Random effect	2898			2655					0.43	[0.19; 0.68]
Prediction interval										[-0.81; 1.68]
**Random effect**	**10085**			**10536**					**0.33**	**[0.13; 0.54]**
**Prediction interval**										**[-0.89; 1.55]**

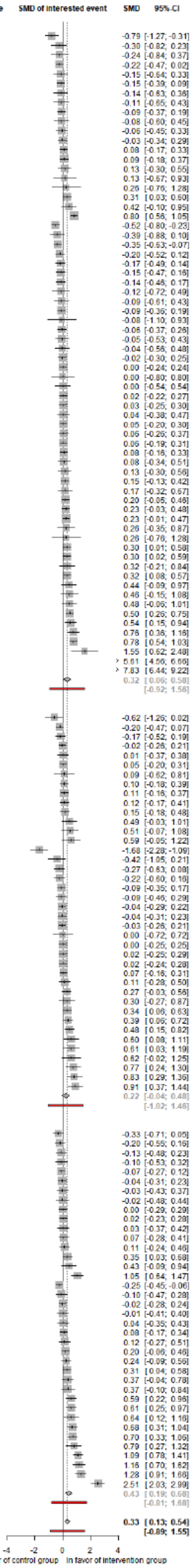

Figure S13.1. Forest plot represents the difference between the intervention vs. control group in the Social QoL domain with the provider subgroups as predicted at week 0 (post-intervention). SMD - Standardized mean difference, CI - confidence interval.

Figure S13.2.T12

Study	Experimental Patient N	Experimental Mean	Experimental SD	Control Patient N	Control Mean	Control SD	follow-up	Follow-up time	SMD	95%-CI
**psychologist**										
Rodríguez Vega, B, et al., 2010	39	3.31	3.32	33	6.06	3.62	baseline	0.00	-0.79	[-1.27; -0.31]
Peng, L, et al., 2022	28	76.79	15.27	29	81.61	16.87	baseline	0.00	-0.30	[-0.82; 0.23]
Serfaty M, et al., 2018	20	16.40	6.10	22	18.10	7.80	baseline	0.00	-0.24	[-0.84; 0.37]
Qiu, H, et al., 2018 II.	98	17.84	5.10	196	18.85	4.22	baseline	0.00	-0.22	[-0.47; 0.02]
Heiney, SP, et al., 2003	33	5.60	2.00	33	5.90	1.90	baseline	0.00	-0.15	[-0.64; 0.33]
Qiu, H, et al., 2018 I.	98	18.13	5.70	196	18.85	4.22	baseline	0.00	-0.15	[-0.39; 0.09]
Ruiz-Vozmediano, J, et al.,2020	31	69.40	29.80	32	73.40	28.40	baseline	0.00	-0.14	[-0.63; 0.36]
Seliniotaki, T, et al., 2021	27	74.40	29.90	26	77.70	29.30	baseline	0.00	-0.11	[-0.65; 0.43]
Antoni, MH, et al., 2006	92	877.61	158.55	107	891.17	152.16	baseline	0.00	-0.09	[-0.37; 0.19]
Hernandez, EG, et al. 2018	28	17.11	6.05	28	17.56	5.46	baseline	0.00	-0.08	[-0.60; 0.45]
Liu, T, et al., 2019	49	63.77	24.95	53	65.22	23.71	baseline	0.00	-0.06	[-0.45; 0.33]
Chan, et al. 2005	80	54.76	60.02	75	56.28	54.76	baseline	0.00	-0.03	[-0.34; 0.29]
Johansson, B, et al., 2008	128	84.00	24.00	116	82.00	25.00	baseline	0.00	0.08	[-0.17; 0.33]
Klinkhammer-Schalke, M et al., 2012	100	71.21	33.50	100	67.99	34.25	baseline	0.00	0.09	[-0.18; 0.37]
Arving, C, et al., 2007	47	78.00	27.00	38	75.00	19.00	baseline	0.00	0.13	[-0.30; 0.55]
Rahmani, S, et al. 2015	12	34.72	13.22	12	33.33	7.11	baseline	0.00	0.13	[-0.67; 0.93]
Rodrigez, B, et al., 2014	8	95.83	11.79	7	90.48	25.20	baseline	0.00	0.26	[-0.76; 1.28]
Penedo, FJ, et al., 2020	95	-19.85	4.09	97	-21.16	4.24	baseline	0.00	0.31	[0.03; 0.60]
Powell, CB, et al., 2008	21	22.20	5.40	43	19.70	6.00	baseline	0.00	0.42	[-0.10; 0.95]
Lu, Z, et al., 201	203	75.30	4.00	103	71.30	6.50	baseline	0.00	0.80	[0.56; 1.05]
Antoni, MH, et al., 2006	92	786.15	133.08	107	855.60	126.82	followup	48.00	-0.52	[-0.80; -0.23]
Heiney, SP, et al., 2003	33	5.80	2.00	33	6.55	1.80	followup	16.00	-0.39	[-0.88; 0.10]
Antoni, MH, et al., 2006	92	832.88	119.31	107	873.38	114.05	followup	24.00	-0.35	[-0.63; -0.07]
Chan, et al. 2005	80	76.19	54.76	75	87.23	54.76	followup	72.00	-0.20	[-0.52; 0.12]
Chan, et al. 2005	80	65.58	54.76	75	75.11	54.76	followup	24.00	-0.17	[-0.49; 0.14]
Chan, et al. 2005	80	76.62	54.76	75	85.06	54.76	followup	60.00	-0.15	[-0.47; 0.16]
Chan, et al. 2005	80	61.47	54.76	75	69.26	54.76	followup	12.00	-0.14	[-0.46; 0.17]
Serfaty M, et al., 2018	20	18.70	5.30	22	19.40	6.20	followup	12.00	-0.12	[-0.72; 0.49]
Peng, L, et al., 2022	28	80.36	20.09	29	82.18	19.38	followup	0.10	-0.09	[-0.61; 0.43]
Klinkhammer-Schalke, M et al., 2012	100	78.90	27.54	100	81.14	24.07	followup	48.00	-0.09	[-0.36; 0.19]
Rodrigez, B, et al., 2014	8	93.33	14.91	7	94.44	9.62	followup	2.00	-0.08	[-1.10; 0.93]
Chan, et al. 2005	80	80.52	54.76	75	83.77	54.76	followup	48.00	-0.06	[-0.37; 0.26]
Heiney, SP, et al., 2003	33	5.80	2.00	33	5.90	1.90	followup	6.00	-0.05	[-0.53; 0.43]
Peng, L, et al., 2022	28	79.17	16.49	29	79.89	20.11	followup	4.00	-0.04	[-0.56; 0.48]
Klinkhammer-Schalke, M et al., 2012	100	66.00	33.00	100	66.74	31.76	followup	12.00	-0.02	[-0.30; 0.25]
Qiu, H, et al., 2018 II.	98	17.98	8.92	196	17.98	8.52	followup	4.00	0.00	[-0.24; 0.24]
Rahmani, S, et al. 2015	12	12.00	54.16	12	12.00	34.72	followup	16.00	0.00	[-0.80; 0.80]
Seliniotaki, T, et al., 2021	27	74.60	35.90	26	74.60	29.30	followup	8.00	0.00	[-0.54; 0.54]
Qiu, H, et al., 2018 II.	98	18.97	8.92	196	18.75	9.46	followup	24.00	0.02	[-0.22; 0.27]
Klinkhammer-Schalke, M et al., 2012	100	72.20	28.78	100	71.46	29.03	followup	24.00	0.03	[-0.25; 0.30]
Arving, C, et al., 2007	47	76.00	25.00	38	75.00	19.00	followup	12.00	0.04	[-0.38; 0.47]
Johansson, B, et al., 2008	128	91.00	18.00	116	90.00	20.00	followup	96.00	0.05	[-0.20; 0.30]
Chan, et al. 2005	80	80.95	54.76	75	77.92	54.76	followup	36.00	0.06	[-0.26; 0.37]
Johansson, B, et al., 2008	128	92.00	15.00	116	91.00	18.00	followup	48.00	0.06	[-0.19; 0.31]
Qiu, H, et al., 2018 II.	98	18.77	8.03	196	18.25	5.05	followup	12.00	0.08	[-0.16; 0.33]
Arving, C, et al., 2007	47	81.00	22.00	38	79.00	25.00	followup	4.00	0.08	[-0.34; 0.51]
Arving, C, et al., 2007	47	83.00	21.00	38	80.00	26.00	followup	24.00	0.13	[-0.30; 0.56]
Klinkhammer-Schalke, M et al., 2012	100	77.91	27.05	100	73.69	30.77	followup	36.00	0.15	[-0.13; 0.42]
Ruiz-Vozmediano, J, et al.,2020	31	83.30	24.70	32	78.70	28.50	followup	24.00	0.17	[-0.32; 0.67]
Johansson, B, et al., 2008	128	90.00	17.00	116	86.00	22.00	followup	24.00	0.20	[-0.05; 0.46]
Johansson, B, et al., 2008	128	87.00	20.00	116	82.00	24.00	followup	12.00	0.23	[-0.03; 0.48]
Qiu, H, et al., 2018 I.	98	20.01	9.15	196	17.98	8.52	followup	4.00	0.23	[-0.01; 0.47]
Serfaty M, et al., 2018	20	21.20	4.30	22	19.70	6.70	followup	18.00	0.26	[-0.35; 0.87]
Rodrigez, B, et al., 2014	8	95.83	11.79	7	90.48	25.20	followup	0.10	0.26	[-0.76; 1.28]
Penedo, FJ, et al., 2020	95	-20.74	4.29	97	-22.05	4.43	followup	48.00	0.30	[0.01; 0.58]
Penedo, FJ, et al., 2020	95	-20.61	4.19	97	-21.91	4.33	followup	24.00	0.30	[0.02; 0.59]
Powell, CB, et al., 2008	21	21.30	5.60	43	19.10	7.30	followup	12.00	0.32	[-0.21; 0.84]
Qiu, H, et al., 2018 I.	98	21.75	8.70	196	18.75	9.46	followup	24.00	0.32	[0.08; 0.57]
Hernandez, EG, et al. 2018	28	18.82	5.79	28	16.40	5.07	followup	24.00	0.44	[-0.09; 0.97]
Serfaty M, et al., 2018	20	19.50	7.20	22	16.00	7.60	followup	24.00	0.46	[-0.15; 1.08]
Hernandez, EG, et al. 2018	28	19.23	5.51	28	16.60	5.39	followup	8.00	0.48	[-0.06; 1.01]
Qiu, H, et al., 2018 I.	98	21.39	8.03	196	18.25	5.05	followup	12.00	0.50	[0.26; 0.75]
Liu, T, et al., 2019	49	65.81	18.49	53	56.11	17.08	followup	9.00	0.54	[0.15; 0.94]
Liu, T, et al., 2019	49	74.35	17.68	53	62.05	14.34	followup	12.00	0.76	[0.36; 1.16]
Lu, Z, et al., 201	203	76.10	5.20	103	71.10	8.20	followup	9.00	0.78	[0.54; 1.03]
Rahmani, S, et al. 2015	12	51.38	11.14	12	29.16	16.09	followup	8.00	1.55	[0.62; 2.48]
Rodríguez Vega, B, et al., 2010	39	49.04	3.32	33	29.42	3.62	followup	12.00	5.61	[4.56; 6.66]
Rodríguez Vega, B, et al., 2010	39	61.86	3.32	33	34.47	3.62	followup	24.00	7.83	[6.44; 9.22]
Random effect	4468			5018					0.33	[0.02; 0.65]
Prediction interval										[-0.94; 1.61]
**healthcare professional**										
Ferguson, RJ, et al., 2012	19	6.26	2.05	21	7.31	1.21	baseline	0.00	-0.62	[-1.26; 0.02]
Hoffman, CJ, et al., 2012	102	17.59	5.91	109	18.78	6.01	baseline	0.00	-0.20	[-0.47; 0.07]
Berglund, G. et al. 2007	39	80.30	22.60	150	84.00	22.10	baseline	0.00	-0.17	[-0.52; 0.19]
Braeken APB, et al. 2013	136	84.94	21.44	144	85.46	22.52	baseline	0.00	-0.02	[-0.26; 0.21]
Schofield, P, et al., 2013	55	70.90	26.77	53	70.75	26.72	baseline	0.00	0.01	[-0.37; 0.38]
Girgis, A, et al., 2009 II.	120	80.80	25.60	117	79.50	25.10	baseline	0.00	0.05	[-0.20; 0.31]
Elyasi, F, et al., 2021 I.	15	2.00	1.20	15	1.90	0.90	baseline	0.00	0.09	[-0.62; 0.81]
Yun et al., 2017	134	75.80	26.80	72	73.10	23.40	baseline	0.00	0.10	[-0.18; 0.39]
Girgis, A, et al., 2009 I.	110	82.10	24.00	117	79.50	25.10	baseline	0.00	0.11	[-0.16; 0.37]
Guo, Z, et al., 2013	89	75.00	13.82	89	73.43	12.31	baseline	0.00	0.12	[-0.17; 0.41]
Sandsund, C, et al., 2017	72	74.10	26.70	70	70.00	29.00	baseline	0.00	0.15	[-0.18; 0.48]
Cheung YL, et al. 2002	29	11.00	0.70	30	10.16	2.26	baseline	0.00	0.49	[-0.03; 1.01]
Trask, PC, et al. 2003	25	71.50	22.00	23	58.20	29.25	baseline	0.00	0.51	[-0.07; 1.08]
Elyasi, F, et al., 2021 II.	20	2.50	1.10	20	1.90	0.90	baseline	0.00	0.59	[-0.05; 1.22]
Beatty, L. et al. 2015	30	57.26	5.49	30	66.56	5.41	followup	6.00	-1.68	[-2.28; -1.09]
Ferguson, RJ, et al., 2012	19	6.75	1.78	21	7.47	1.59	followup	8.00	-0.42	[-1.05; 0.21]
Berglund, G. et al. 2007	39	77.00	23.70	150	83.50	23.70	followup	48.00	-0.27	[-0.63; 0.08]
Schofield, P, et al., 2013	55	65.03	28.36	53	71.29	28.69	followup	12.00	-0.22	[-0.60; 0.16]
Girgis, A, et al., 2009 I.	110	88.20	19.30	117	89.90	17.00	followup	12.00	-0.09	[-0.35; 0.17]
Schofield, P, et al., 2013	55	68.28	26.22	53	70.58	26.38	followup	8.00	-0.09	[-0.46; 0.29]
Girgis, A, et al., 2009 II.	120	89.20	20.30	117	89.90	17.00	followup	12.00	-0.04	[-0.29; 0.22]
Hoffman, CJ, et al., 2012	102	18.09	5.81	109	18.30	5.75	followup	12.00	-0.04	[-0.31; 0.23]
Braeken APB, et al. 2013	136	86.99	20.73	144	87.55	19.10	followup	48.00	-0.03	[-0.26; 0.21]
Elyasi, F, et al., 2021 I.	15	1.80	0.90	15	1.80	0.90	followup	24.00	0.00	[-0.72; 0.72]
Girgis, A, et al., 2009 II.	120	91.90	17.60	117	91.90	17.40	followup	24.00	0.00	[-0.25; 0.25]
Hoffman, CJ, et al., 2012	102	18.36	5.65	109	18.26	5.88	followup	8.00	0.02	[-0.25; 0.29]
Girgis, A, et al., 2009 I.	110	92.20	15.00	117	91.90	17.40	followup	24.00	0.02	[-0.24; 0.28]
Braeken APB, et al. 2013	136	83.46	23.57	144	81.81	22.37	followup	12.00	0.07	[-0.16; 0.31]
Guan, S, et al., 2019	50	69.05	7.24	50	68.19	8.05	followup	8.00	0.11	[-0.28; 0.50]
Guo, Z, et al., 2013	89	74.35	10.67	89	71.33	11.88	followup	2.00	0.27	[-0.03; 0.56]
Trask, PC, et al. 2003	25	86.10	23.50	23	79.40	20.62	followup	24.00	0.30	[-0.27; 0.87]
Yun et al., 2017	134	85.30	19.50	72	78.20	22.40	followup	48.00	0.34	[0.06; 0.63]
Sandsund, C, et al., 2017	72	85.00	21.40	70	74.90	29.80	followup	12.00	0.39	[0.06; 0.72]
Sandsund, C, et al., 2017	72	86.00	18.90	70	74.20	28.90	followup	24.00	0.48	[0.15; 0.82]
Beatty, L. et al. 2015	30	77.87	4.62	30	75.08	4.61	followup	13.03	0.60	[0.08; 1.11]
Trask, PC, et al. 2003	25	85.90	24.00	23	70.10	27.34	followup	8.00	0.61	[0.03; 1.19]
Elyasi, F, et al., 2021 II.	20	2.40	1.00	20	1.80	0.90	followup	24.00	0.62	[-0.02; 1.25]
Cheung YL, et al. 2002	29	10.55	0.50	30	9.64	1.56	followup	5.00	0.77	[0.24; 1.30]
Cheung YL, et al. 2002	29	10.37	0.49	30	9.47	1.43	followup	10.00	0.83	[0.29; 1.36]
Beatty, L. et al. 2015	30	87.98	4.26	30	83.84	4.75	followup	26.07	0.91	[0.37; 1.44]
Random effect	2719			2863					0.23	[0.06; 0.40]
Prediction interval										[-1.03; 1.49]
**nurse**										
Walczak, A, et al.,2017	61	21.16	4.48	49	22.71	4.99	baseline	0.00	-0.33	[-0.71; 0.05]
Chen, et al., 2017	58	58.30	6.20	65	59.60	6.90	baseline	0.00	-0.20	[-0.55; 0.16]
Fang, P, et al.,2020	60	48.50	4.11	60	49.08	5.01	baseline	0.00	-0.13	[-0.48; 0.23]
Wu, Q, et al., 2021	43	53.77	5.51	43	54.34	5.53	baseline	0.00	-0.10	[-0.53; 0.32]
McLachlan, SA, et al., 2001	296	41.20	22.60	154	42.80	18.70	baseline	0.00	-0.07	[-0.27; 0.12]
Li, X, et al., 2017	102	13.58	2.23	108	13.67	2.21	baseline	0.00	-0.04	[-0.31; 0.23]
Kim, SH, et al., 2021	47	73.70	81.90	47	75.50	22.00	baseline	0.00	-0.03	[-0.43; 0.37]
Dirksen, S. et al, 2007	34	22.10	4.90	38	22.20	6.10	baseline	0.00	-0.02	[-0.48; 0.44]
van der Meulen, IC, et al., 2013	88	83.10	2.50	91	83.10	2.50	baseline	0.00	0.00	[-0.29; 0.29]
Klafke, N, et al., 2019	120	59.30	31.30	113	58.60	29.60	baseline	0.00	0.02	[-0.23; 0.28]
Qin, X, et al., 2017	50	77.68	15.31	50	77.26	16.04	baseline	0.00	0.03	[-0.37; 0.42]
Baoyindeligeer, L.Z. et al. 2020	65	66.59	14.95	65	65.59	15.24	baseline	0.00	0.07	[-0.28; 0.41]
Zhao,X, et al. 2015	62	7.01	2.18	62	6.76	2.39	baseline	0.00	0.11	[-0.24; 0.46]
Thomas, ML, et al., 2012	64	21.10	5.40	88	19.00	6.30	baseline	0.00	0.35	[0.03; 0.68]
Kim, YH, et al., 2017	30	72.80	25.30	30	62.80	20.80	baseline	0.00	0.43	[-0.09; 0.94]
Zhao, X, et al., 2021	52	58.02	3.41	51	54.01	4.12	baseline	0.00	1.05	[0.64; 1.47]
McLachlan, SA, et al., 2001	296	42.00	23.70	154	47.50	17.90	followup	24.00	-0.25	[-0.45; -0.06]
Walczak, A, et al.,2017	61	20.94	5.40	49	21.43	4.47	followup	4.00	-0.10	[-0.47; 0.28]
Klafke, N, et al., 2019	120	54.50	29.60	113	55.10	29.50	followup	24.00	-0.02	[-0.28; 0.24]
Kim, SH, et al., 2021	47	80.30	17.60	47	80.40	18.50	followup	20.00	-0.01	[-0.41; 0.40]
Qin, X, et al., 2017	50	80.24	13.46	50	79.67	13.13	followup	2.00	0.04	[-0.35; 0.43]
Klafke, N, et al., 2019	120	62.10	29.40	113	59.70	27.60	followup	12.00	0.08	[-0.17; 0.34]
Zhao, X, et al., 2021	52	58.62	6.31	51	58.02	3.21	followup	12.00	0.12	[-0.27; 0.51]
Klafke, N, et al., 2019	120	69.10	27.90	113	63.60	26.60	followup	48.00	0.20	[-0.06; 0.46]
Thomas, ML, et al., 2012	64	20.50	6.10	88	19.00	6.40	followup	12.00	0.24	[-0.09; 0.56]
Li, X, et al., 2017	102	15.45	2.67	108	14.52	3.28	followup	12.00	0.31	[0.04; 0.58]
Kim, SH, et al., 2021	47	81.90	15.20	47	74.60	23.00	followup	8.00	0.37	[-0.04; 0.78]
Dirksen, S. et al, 2007	34	23.30	3.90	38	21.40	5.90	followup	10.00	0.37	[-0.10; 0.84]
Zhou, J, et al., 2020	59	72.34	11.43	59	65.87	10.26	followup	2.00	0.59	[0.22; 0.96]
Zhao,X, et al. 2015	62	8.19	2.10	62	6.87	2.21	followup	0.30	0.61	[0.25; 0.97]
Kim, YH, et al., 2017	30	75.60	26.20	30	58.30	27.20	followup	6.00	0.64	[0.12; 1.16]
Chen, et al., 2017	58	47.60	7.50	65	42.60	7.20	followup	8.00	0.68	[0.31; 1.04]
Chen, et al., 2017	58	46.80	7.60	65	41.50	7.50	followup	24.00	0.70	[0.33; 1.06]
Kim, YH, et al., 2017	30	75.00	23.10	30	54.40	28.00	followup	9.00	0.79	[0.27; 1.32]
van der Meulen, IC, et al., 2013	88	88.50	3.20	91	85.10	3.00	followup	48.00	1.09	[0.78; 1.41]
Wu, Q, et al., 2021	43	81.65	8.30	43	72.36	7.51	followup	12.00	1.16	[0.70; 1.62]
Baoyindeligeer, L.Z. et al. 2020	65	88.33	12.38	65	71.36	13.84	followup	2.00	1.28	[0.91; 1.66]
Fang, P, et al.,2020	60	65.47	6.96	60	49.45	5.65	followup	4.00	2.51	[2.03; 2.99]
Random effect	2898			2655					0.44	[0.23; 0.66]
Prediction interval										[-0.82; 1.71]
**Random effect**	**10085**			**10536**					**0.33**	**[0.15; 0.51]**
**Prediction interval**										**[-0.86; 1.52]**

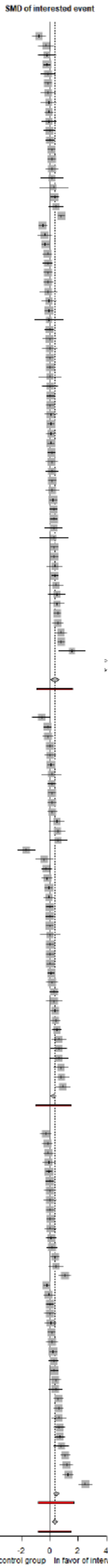

Figure S13.2. Forest plot represents the difference between the intervention vs. control group in the Social QoL domain with the provider subgroups as predicted at week 12 (post-intervention). SMD - Standardized mean difference, CI - confidence interval

Figure S13.3.T24

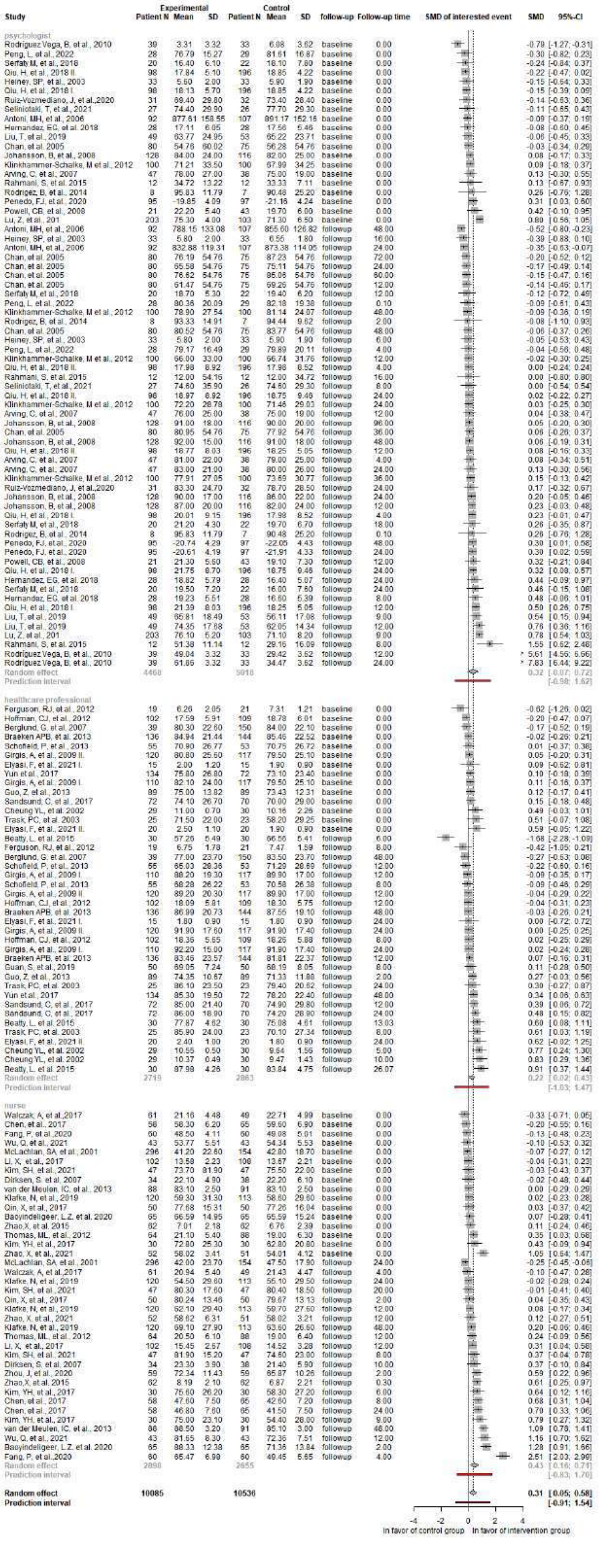

Figure S13.3.. Forest plot represents the difference between the intervention vs. control group in the Social QoL domain with the provider subgroups as predicted at week 24 (post-intervention). SMD - Standardized mean difference, CI - confidence interval

Figure S13.4.T48

Study	Experimental Patient N	Experimental Mean	Experimental SD	Control Patient N	Control Mean	Control SD	follow-up	Follow-up time	SMD	95%-CI
psychologist										
Rodriguez Vega, B, et al., 2010	39	3.31	3.32	33	6.08	3.62	baseline	0.00	-0.79	[-1.27; -0.31]
Peng, L, et al., 2022	28	76.79	15.27	29	81.61	16.87	baseline	0.00	-0.30	[-0.82; 0.23]
Serfaty M, et al., 2018	20	16.40	6.10	22	18.10	7.80	baseline	0.00	-0.24	[-0.84; 0.37]
Qiu, H, et al., 2018 II.	98	17.84	5.10	196	18.85	4.22	baseline	0.00	-0.22	[-0.47; 0.02]
Heiney, SP, et al., 2003	33	5.60	2.00	33	5.90	1.90	baseline	0.00	-0.15	[-0.64; 0.33]
Qiu, H, et al., 2018 I.	98	18.13	5.70	196	18.85	4.22	baseline	0.00	-0.15	[-0.39; 0.09]
Ruiz-Vozmediano, J, et al.,2020	31	69.40	29.80	32	73.40	28.40	baseline	0.00	-0.14	[-0.63; 0.36]
Seliniotaki, T, et al., 2021	27	74.40	29.90	26	77.70	29.30	baseline	0.00	-0.11	[-0.65; 0.43]
Antoni, MH, et al., 2006	92	877.61	158.55	107	891.17	152.16	baseline	0.00	-0.09	[-0.37; 0.19]
Hernandez, EG, et al. 2018	28	17.11	6.05	28	17.56	5.46	baseline	0.00	-0.08	[-0.60; 0.45]
Liu, T, et al., 2019	49	63.77	24.95	53	65.22	23.71	baseline	0.00	-0.06	[-0.45; 0.33]
Chan, et al. 2005	80	54.76	60.02	75	56.28	54.76	baseline	0.00	-0.03	[-0.34; 0.29]
Johansson, B, et al., 2008	128	84.00	24.00	116	82.00	25.00	baseline	0.00	0.08	[-0.17; 0.33]
Klinkhammer-Schalke, M et al., 2012	100	71.21	33.50	100	67.99	34.25	baseline	0.00	0.09	[-0.18; 0.37]
Arving, C, et al., 2007	47	78.00	27.00	38	75.00	19.00	baseline	0.00	0.13	[-0.30; 0.55]
Rahmani, S, et al. 2015	12	34.72	13.22	12	33.33	7.11	baseline	0.00	0.13	[-0.67; 0.93]
Rodrigez, B, et al., 2014	8	95.83	11.79	7	90.48	25.20	baseline	0.00	0.26	[-0.76; 1.28]
Penedo, FJ, et al., 2020	95	-19.85	4.09	97	-21.16	4.24	baseline	0.00	0.31	[0.03; 0.60]
Powell, CB, et al., 2008	21	22.20	5.40	43	19.70	6.00	baseline	0.00	0.42	[-0.10; 0.95]
Lu, Z, et al., 201	203	75.30	4.00	103	71.30	6.50	baseline	0.00	0.80	[0.56; 1.05]
Antoni, MH, et al., 2006	92	788.15	133.08	107	855.60	126.82	followup	48.00	-0.52	[-0.80; -0.23]
Heiney, SP, et al., 2003	33	5.80	2.00	33	6.55	1.80	followup	16.00	-0.39	[-0.88; 0.10]
Antoni, MH, et al., 2006	92	832.88	119.31	107	873.38	114.05	followup	24.00	-0.35	[-0.63; -0.07]
Chan, et al. 2005	80	76.19	54.76	75	87.23	54.75	followup	72.00	-0.20	[-0.52; 0.12]
Chan, et al. 2005	80	65.58	54.76	75	75.11	54.75	followup	24.00	-0.17	[-0.49; 0.14]
Chan, et al. 2005	80	76.62	54.76	75	85.06	54.76	followup	60.00	-0.15	[-0.47; 0.16]
Chan, et al. 2005	80	61.47	54.76	75	69.26	54.76	followup	12.00	-0.14	[-0.46; 0.17]
Serfaty M, et al., 2018	20	18.70	5.30	22	19.40	6.20	followup	12.00	-0.12	[-0.72; 0.49]
Peng, L, et al., 2022	28	80.36	20.09	29	82.18	19.38	followup	0.10	-0.09	[-0.61; 0.43]
Klinkhammer-Schalke, M et al., 2012	100	78.90	27.54	100	81.14	24.07	followup	48.00	-0.09	[-0.36; 0.19]
Rodrigez, B, et al., 2014	8	93.33	14.91	7	94.44	9.62	followup	2.00	-0.08	[-1.10; 0.93]
Chan, et al. 2005	80	80.52	54.76	75	83.77	54.76	followup	48.00	-0.06	[-0.37; 0.26]
Heiney, SP, et al., 2003	33	5.80	2.00	33	5.90	1.90	followup	6.00	-0.05	[-0.53; 0.43]
Peng, L, et al., 2022	28	79.17	16.49	29	79.89	20.11	followup	4.00	-0.04	[-0.56; 0.48]
Klinkhammer-Schalke, M et al., 2012	100	66.00	33.00	100	66.74	31.76	followup	12.00	-0.02	[-0.30; 0.25]
Qiu, H, et al., 2018 II.	98	17.98	8.92	196	17.98	8.52	followup	4.00	0.00	[-0.24; 0.24]
Rahmani, S, et al. 2015	12	12.00	54.16	12	12.00	34.72	followup	16.00	0.00	[-0.80; 0.80]
Seliniotaki, T, et al., 2021	27	74.60	35.90	26	74.60	29.30	followup	8.00	0.00	[-0.54; 0.54]
Qiu, H, et al., 2018 II.	98	18.97	8.92	196	18.75	9.46	followup	24.00	0.02	[-0.22; 0.27]
Klinkhammer-Schalke, M et al., 2012	100	72.20	28.78	100	71.46	29.03	followup	24.00	0.03	[-0.25; 0.30]
Arving, C, et al., 2007	47	76.00	25.00	38	75.00	19.00	followup	12.00	0.04	[-0.38; 0.47]
Johansson, B, et al., 2008	128	91.00	18.00	116	90.00	20.00	followup	96.00	0.05	[-0.20; 0.30]
Chan, et al. 2005	80	80.95	54.76	75	77.92	54.76	followup	36.00	0.06	[-0.26; 0.37]
Johansson, B, et al., 2008	128	92.00	15.00	116	91.00	18.00	followup	48.00	0.06	[-0.19; 0.31]
Qiu, H, et al., 2018 II.	98	18.77	8.03	196	18.25	5.05	followup	12.00	0.08	[-0.16; 0.33]
Arving, C, et al., 2007	47	81.00	22.00	38	79.00	25.00	followup	4.00	0.08	[-0.34; 0.51]
Arving, C, et al., 2007	47	83.00	21.00	38	80.00	26.00	followup	24.00	0.13	[-0.30; 0.56]
Klinkhammer-Schalke, M et al., 2012	100	77.91	27.05	100	73.69	30.77	followup	36.00	0.15	[-0.13; 0.42]
Ruiz-Vozmediano, J, et al.,2020	31	83.30	24.70	32	78.70	28.50	followup	24.00	0.17	[-0.32; 0.67]
Johansson, B, et al., 2008	128	90.00	17.00	116	86.00	22.00	followup	24.00	0.20	[-0.05; 0.46]
Johansson, B, et al., 2008	128	87.00	20.00	116	82.00	24.00	followup	12.00	0.23	[-0.03; 0.48]
Qiu, H, et al., 2018 I.	98	20.01	9.15	196	17.98	8.52	followup	4.00	0.23	[-0.01; 0.47]
Serfaty M, et al., 2018	20	21.20	4.30	22	19.70	6.70	followup	18.00	0.26	[-0.35; 0.87]
Rodrigez, B, et al., 2014	8	95.83	11.79	7	90.48	25.20	followup	0.10	0.26	[-0.76; 1.28]
Penedo, FJ, et al., 2020	95	-20.74	4.29	97	-22.05	4.43	followup	48.00	0.30	[0.01; 0.58]
Penedo, FJ, et al., 2020	95	-20.61	4.19	97	-21.91	4.33	followup	24.00	0.30	[0.02; 0.59]
Powell, CB, et al., 2008	21	21.30	5.50	43	19.10	7.30	followup	12.00	0.32	[-0.21; 0.84]
Qiu, H, et al., 2018 I.	98	21.75	8.70	196	18.75	9.46	followup	24.00	0.32	[0.08; 0.57]
Hernandez, EG, et al. 2018	28	18.82	5.79	28	16.40	5.07	followup	24.00	0.44	[-0.09; 0.97]
Serfaty M, et al., 2018	20	19.50	7.20	22	16.00	7.60	followup	24.00	0.46	[-0.15; 1.08]
Hernandez, EG, et al. 2018	28	19.23	5.51	28	16.60	5.39	followup	8.00	0.48	[-0.06; 1.01]
Qiu, H, et al., 2018 I.	98	21.39	8.03	196	18.25	5.05	followup	12.00	0.50	[0.26; 0.75]
Liu, T, et al., 2019	49	65.81	18.49	53	56.11	17.08	followup	9.00	0.54	[0.15; 0.94]
Liu, T, et al., 2019	49	74.35	17.68	53	62.05	14.34	followup	12.00	0.76	[0.36; 1.16]
Lu, Z, et al., 201	203	76.10	5.20	103	71.10	8.20	followup	9.00	0.78	[0.54; 1.03]
Rahmani, S, et al. 2015	12	51.38	11.14	12	29.16	16.09	followup	8.00	1.55	[0.62; 2.48]
Rodriguez Vega, B, et al., 2010	39	49.04	3.32	33	29.42	3.62	followup	12.00	5.61	[4.56; 6.66]
Rodriguez Vega, B, et al., 2010	39	61.86	3.32	33	34.47	3.62	followup	24.00	7.83	[6.44; 9.22]
Random effect	4468			5018					0.23	[-0.19; 0.65]
Prediction interval										[-1.12; 1.58]
healthcare professional										
Ferguson, RJ, et al., 2012	19	6.26	2.05	21	7.31	1.21	baseline	0.00	-0.62	[-1.26; 0.02]
Hoffman, CJ, et al., 2012	102	17.59	5.91	109	18.78	6.01	baseline	0.00	-0.20	[-0.47; 0.07]
Berglund, G. et al. 2007	39	80.30	22.60	150	84.00	22.10	baseline	0.00	-0.17	[-0.52; 0.19]
Braeken APB, et al. 2013	136	84.94	21.44	144	85.46	22.52	baseline	0.00	-0.02	[-0.26; 0.21]
Schofield, P, et al., 2013	55	70.90	26.77	53	70.75	26.72	baseline	0.00	0.01	[-0.37; 0.38]
Girgis, A, et al., 2009 II.	120	80.80	25.50	117	79.50	25.10	baseline	0.00	0.05	[-0.20; 0.31]
Elyasi, F, et al., 2021 I.	15	2.00	1.20	15	1.90	0.90	baseline	0.00	0.09	[-0.62; 0.81]
Yun et al., 2017	134	75.80	26.80	72	73.10	23.40	baseline	0.00	0.10	[-0.18; 0.39]
Girgis, A, et al., 2009 I.	110	82.10	24.00	117	79.50	25.10	baseline	0.00	0.11	[-0.16; 0.37]
Guo, Z, et al., 2013	89	75.00	13.82	89	73.43	12.31	baseline	0.00	0.12	[-0.17; 0.41]
Sandsund, C, et al., 2017	72	74.10	26.70	70	70.00	29.00	baseline	0.00	0.15	[-0.18; 0.48]
Cheung YL, et al. 2002	29	11.00	0.70	30	10.16	2.26	baseline	0.00	0.49	[-0.03; 1.01]
Trask, PC, et al. 2003	25	71.50	22.00	23	58.20	29.25	baseline	0.00	0.51	[-0.07; 1.08]
Elyasi, F, et al., 2021 II.	20	2.50	1.10	20	1.90	0.90	baseline	0.00	0.59	[-0.05; 1.22]
Beatty, L. et al. 2015	30	57.26	5.49	30	66.56	5.41	followup	6.00	-1.68	[-2.28; -1.09]
Ferguson, RJ, et al., 2012	19	6.75	1.78	21	7.47	1.59	followup	8.00	-0.42	[-1.05; 0.21]
Berglund, G. et al. 2007	39	77.00	23.70	150	83.50	23.70	followup	48.00	-0.27	[-0.63; 0.08]
Schofield, P, et al., 2013	55	65.03	28.36	53	71.29	28.69	followup	12.00	-0.22	[-0.60; 0.16]
Girgis, A, et al., 2009 I.	110	88.20	19.30	117	89.90	17.00	followup	12.00	-0.09	[-0.35; 0.17]
Schofield, P, et al., 2013	55	68.28	26.22	53	70.58	26.38	followup	8.00	-0.09	[-0.46; 0.29]
Girgis, A, et al., 2009 II.	120	89.20	20.30	117	89.90	17.00	followup	12.00	-0.04	[-0.29; 0.22]
Hoffman, CJ, et al., 2012	102	18.09	5.81	109	18.30	5.75	followup	12.00	-0.04	[-0.31; 0.23]
Braeken APB, et al. 2013	136	86.99	20.73	144	87.55	19.10	followup	48.00	-0.03	[-0.26; 0.21]
Elyasi, F, et al., 2021 I.	15	1.80	0.90	15	1.80	0.90	followup	24.00	0.00	[-0.72; 0.72]
Girgis, A, et al., 2009 II.	120	91.90	17.60	117	91.90	17.40	followup	24.00	0.00	[-0.25; 0.25]
Hoffman, CJ, et al., 2012	102	18.36	5.65	109	18.26	5.88	followup	8.00	0.02	[-0.25; 0.29]
Girgis, A, et al., 2009 I.	110	92.20	15.00	117	91.90	17.40	followup	24.00	0.02	[-0.24; 0.28]
Braeken APB, et al. 2013	136	83.46	23.57	144	81.81	22.37	followup	12.00	0.07	[-0.16; 0.31]
Guan, S, et al., 2019	50	69.05	7.24	50	68.19	8.05	followup	8.00	0.11	[-0.28; 0.50]
Guo, Z, et al., 2013	89	74.35	10.67	89	71.33	11.88	followup	2.00	0.27	[-0.03; 0.56]
Trask, PC, et al. 2003	25	86.10	23.50	23	79.40	20.62	followup	24.00	0.30	[-0.27; 0.87]
Yun et al., 2017	134	85.30	19.50	72	78.20	22.40	followup	48.00	0.34	[0.06; 0.63]
Sandsund, C, et al., 2017	72	85.00	21.40	70	74.90	29.80	followup	12.00	0.39	[0.06; 0.72]
Sandsund, C, et al., 2017	72	86.00	18.90	70	74.20	28.90	followup	24.00	0.48	[0.15; 0.82]
Beatty, L. et al. 2015	30	77.87	4.62	30	75.08	4.61	followup	13.03	0.60	[0.08; 1.11]
Trask, PC, et al. 2003	25	85.90	24.00	23	70.10	27.34	followup	8.00	0.61	[0.03; 1.19]
Elyasi, F, et al., 2021 II.	20	2.40	1.00	20	1.80	0.90	followup	24.00	0.62	[-0.02; 1.25]
Cheung YL, et al. 2002	29	10.55	0.50	30	9.64	1.56	followup	5.00	0.77	[0.24; 1.30]
Cheung YL, et al. 2002	29	10.37	0.49	30	9.47	1.43	followup	10.00	0.83	[0.29; 1.36]
Beatty, L. et al. 2015	30	87.98	4.26	30	83.84	4.75	followup	26.07	0.91	[0.37; 1.44]
Random effect	2719			2863					0.13	[-0.12; 0.37]
Prediction interval										[-1.13; 1.38]
nurse										
Walczak, A, et al.,2017	61	21.16	4.48	49	22.71	4.99	baseline	0.00	-0.33	[-0.71; 0.05]
Chen, et al., 2017	58	58.30	6.20	65	59.60	6.90	baseline	0.00	-0.20	[-0.55; 0.16]
Fang, P, et al.,2020	60	48.50	4.11	60	49.08	5.01	baseline	0.00	-0.13	[-0.48; 0.23]
Wu, Q, et al., 2021	43	53.77	5.51	43	54.34	5.53	baseline	0.00	-0.10	[-0.53; 0.32]
McLachlan, SA, et al., 2001	296	41.20	22.60	154	42.80	18.70	baseline	0.00	-0.07	[-0.27; 0.12]
Li, X, et al., 2017	102	13.58	2.23	108	13.67	2.21	baseline	0.00	-0.04	[-0.31; 0.23]
Kim, SH, et al., 2021	47	73.70	81.90	47	75.50	22.00	baseline	0.00	-0.03	[-0.43; 0.37]
Dirksen, S. et al. 2007	34	22.10	4.90	38	22.20	6.10	baseline	0.00	-0.02	[-0.48; 0.44]
van der Meulen, IC, et al., 2013	88	83.10	2.50	91	83.10	2.50	baseline	0.00	0.00	[-0.29; 0.29]
Klafke, N, et al., 2019	120	59.30	31.30	113	58.60	29.60	baseline	0.00	0.02	[-0.23; 0.28]
Qin, X, et al., 2017	50	77.68	15.31	50	77.26	16.04	baseline	0.00	0.03	[-0.37; 0.42]
Baoyindeligeer, L.Z. et al. 2020	65	66.59	14.95	65	65.59	15.24	baseline	0.00	0.07	[-0.28; 0.41]
Zhao X, et al. 2015	62	7.01	2.18	62	6.76	2.39	baseline	0.00	0.11	[-0.24; 0.46]
Thomas, ML, et al., 2012	64	21.10	5.40	88	19.00	6.30	baseline	0.00	0.35	[0.03; 0.68]
Kim, YH, et al., 2017	30	72.80	25.30	30	62.80	20.80	baseline	0.00	0.43	[-0.09; 0.94]
Zhao, X, et al., 2021	52	58.02	3.41	51	54.01	4.12	baseline	0.00	1.05	[0.64; 1.47]
McLachlan, SA, et al., 2001	296	42.00	23.70	154	47.50	17.90	followup	24.00	-0.25	[-0.45; -0.06]
Walczak, A, et al.,2017	61	20.94	5.40	49	21.43	4.47	followup	4.00	-0.10	[-0.47; 0.28]
Klafke, N, et al., 2019	120	54.50	29.60	113	55.10	29.50	followup	24.00	-0.02	[-0.28; 0.24]
Kim, SH, et al., 2021	47	80.30	17.50	47	80.40	18.50	followup	20.00	-0.01	[-0.41; 0.40]
Qin, X, et al., 2017	50	80.24	13.46	50	79.67	13.13	followup	2.00	0.04	[-0.35; 0.43]
Klafke, N, et al., 2019	120	62.10	29.40	113	59.70	27.60	followup	12.00	0.08	[-0.17; 0.34]
Zhao, X, et al., 2021	52	58.62	6.31	51	58.02	3.21	followup	12.00	0.12	[-0.27; 0.51]
Klafke, N, et al., 2019	120	69.10	27.90	113	63.60	26.60	followup	48.00	0.20	[-0.06; 0.46]
Thomas, ML, et al., 2012	64	20.50	6.10	88	19.00	6.40	followup	12.00	0.24	[-0.09; 0.56]
Li, X, et al., 2017	102	15.45	2.67	108	14.52	3.28	followup	12.00	0.31	[0.04; 0.58]
Kim, SH, et al., 2021	47	81.90	15.20	47	74.60	23.00	followup	8.00	0.37	[-0.04; 0.78]
Dirksen, S. et al. 2007	34	23.30	3.90	38	21.40	5.90	followup	10.00	0.37	[-0.10; 0.84]
Zhou, J, et al., 2020	59	72.34	11.43	59	65.87	10.25	followup	2.00	0.59	[0.22; 0.96]
Zhao X, et al. 2015	62	8.19	2.10	62	6.97	2.21	followup	0.30	0.61	[0.25; 0.97]
Kim, YH, et al., 2017	30	75.60	26.20	30	58.30	27.20	followup	6.00	0.64	[0.12; 1.16]
Chen, et al., 2017	58	47.60	7.50	65	42.60	7.20	followup	8.00	0.68	[0.31; 1.04]
Chen, et al., 2017	58	46.80	7.60	65	41.50	7.50	followup	24.00	0.70	[0.33; 1.06]
Kim, YH, et al., 2017	30	75.00	23.10	30	54.40	29.00	followup	9.00	0.79	[0.27; 1.32]
van der Meulen, IC, et al., 2013	88	88.50	3.20	91	85.10	3.00	followup	48.00	1.09	[0.78; 1.41]
Wu, Q, et al., 2021	43	81.65	8.30	43	72.36	7.51	followup	12.00	1.16	[0.70; 1.62]
Baoyindeligeer, L.Z. et al. 2020	65	88.33	12.38	65	71.36	13.84	followup	2.00	1.28	[0.91; 1.66]
Fang, P, et al.,2020	60	65.47	6.98	60	49.45	5.65	followup	4.00	2.51	[2.03; 2.99]
Random effect	2898			2655					0.34	[0.03; 0.65]
Prediction interval										[-0.93; 1.61]
**Random effect**	**10085**			**10536**					**0.21**	**[-0.11; 0.54]**
**Prediction interval**										**[-1.09; 1.52]**

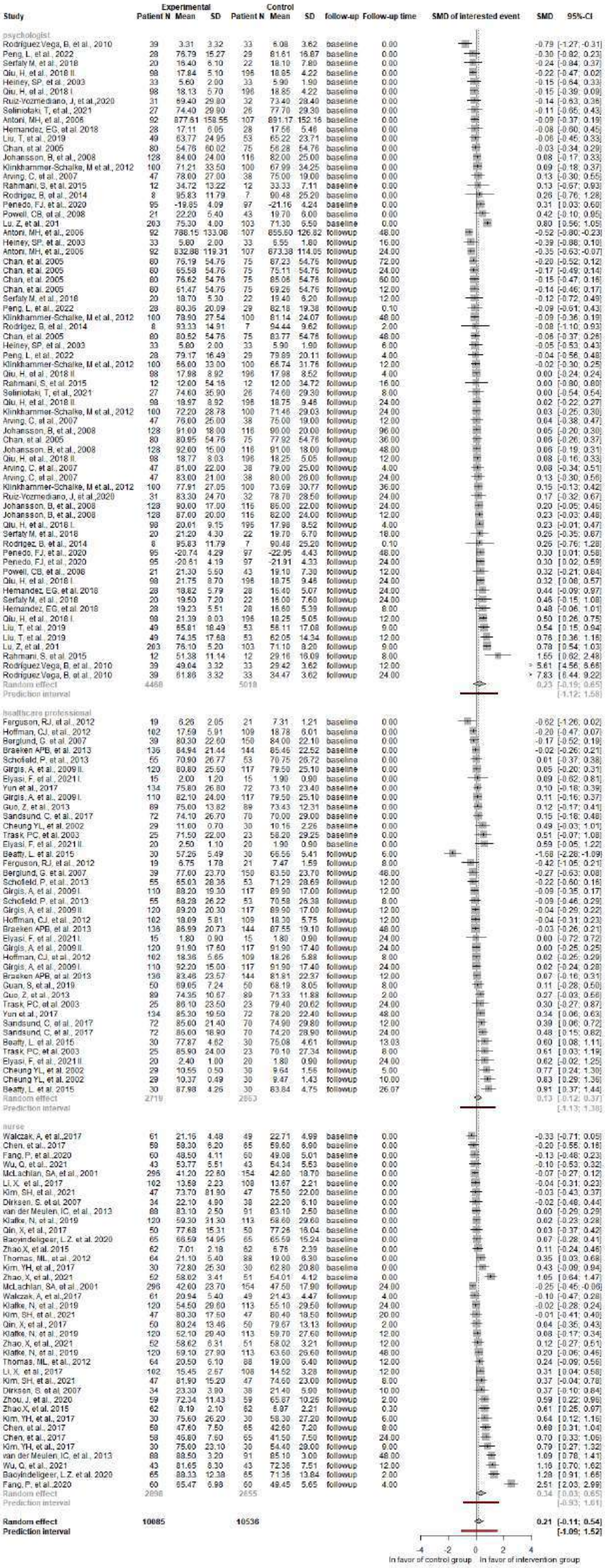

Figure S13.4.. Forest plot represents the difference between the intervention vs. control group in the Social QoL domain with the provider subgroups as predicted at week 48 (post-intervention). SMD - Standardized mean difference, CI - confidence interval

S14.Subgroup analysis of Social QoL: Environment

Figure S14.1.T0

Study	Experimental Patient N	Experimental Mean	Experimental SD	Control Patient N	Control Mean	Control SD	follow-up	Follow-up time	SMD of interested event	SMD	95%-CI
face to face											
Rodríguez Vega, B, et al., 2010	39	3.31	3.32	33	6.08	3.62	baseline	0.00		-0.79	[-1.27; -0.31]
Ferguson, RJ, et al., 2012	19	6.26	2.05	21	7.31	1.21	baseline	0.00		-0.62	[-1.26; 0.02]
Serfaty M, et al., 2018	20	16.40	6.10	22	18.10	7.80	baseline	0.00		-0.24	[-0.84; 0.37]
Qiu, H, et al., 2018 II.	98	17.84	5.10	196	18.85	4.22	baseline	0.00		-0.22	[-0.47; 0.02]
Chen, et al., 2017	58	58.30	6.20	65	59.60	6.90	baseline	0.00		-0.20	[-0.55; 0.16]
Berglund, G. et al. 2007	39	80.30	22.60	150	84.00	22.10	baseline	0.00		-0.17	[-0.52; 0.19]
Qiu, H, et al., 2018 I.	98	18.13	5.70	196	18.85	4.22	baseline	0.00		-0.15	[-0.39; 0.09]
Ruiz-Vozmediano, J, et al.,2020	31	69.40	29.80	32	73.40	28.40	baseline	0.00		-0.14	[-0.63; 0.36]
Fang, P, et al.,2020	60	48.50	4.11	60	49.08	5.01	baseline	0.00		-0.13	[-0.48; 0.23]
Seliniotaki, T, et al., 2021	27	74.40	29.90	26	77.70	29.30	baseline	0.00		-0.11	[-0.65; 0.43]
Antoni, MH, et al., 2006	92	877.61	158.55	107	891.17	152.16	baseline	0.00		-0.09	[-0.37; 0.19]
Hernandez, EG, et al. 2018	28	17.11	6.05	28	17.56	5.46	baseline	0.00		-0.08	[-0.60; 0.45]
McLachlan, SA, et al., 2001	296	41.20	22.60	154	42.80	18.70	baseline	0.00		-0.07	[-0.27; 0.12]
Liu, T, et al., 2019	49	63.77	24.95	53	65.22	23.71	baseline	0.00		-0.06	[-0.45; 0.33]
Li, X, et al., 2017	102	13.58	2.23	108	13.67	2.21	baseline	0.00		-0.04	[-0.31; 0.23]
Chan, et al. 2005	80	54.76	60.02	75	56.28	54.76	baseline	0.00		-0.03	[-0.34; 0.29]
Braeken APB, et al. 2013	136	84.94	21.44	144	85.46	22.52	baseline	0.00		-0.02	[-0.26; 0.21]
van der Meulen, IC, et al., 2013	88	83.10	2.50	91	83.10	2.50	baseline	0.00		0.00	[-0.29; 0.29]
Schofield, P, et al., 2013	55	70.90	26.77	53	70.75	26.72	baseline	0.00		0.01	[-0.37; 0.38]
Klafke, N, et al., 2019	120	59.30	31.30	113	58.60	29.60	baseline	0.00		0.02	[-0.23; 0.28]
Qin, X, et al., 2017	50	77.68	15.31	50	77.26	16.04	baseline	0.00		0.03	[-0.37; 0.42]
Baoyindeligeer, L.Z. et al. 2020	65	66.59	14.95	65	65.59	15.24	baseline	0.00		0.07	[-0.28; 0.41]
Elyasi, F, et al., 2021 I.	15	2.00	1.20	15	1.90	0.90	baseline	0.00		0.09	[-0.62; 0.81]
Klinkhammer-Schalke, M et al., 2012	100	71.21	33.50	100	67.99	34.25	baseline	0.00		0.09	[-0.18; 0.37]
Zhao,X, et al. 2015	62	7.01	2.18	62	6.76	2.39	baseline	0.00		0.11	[-0.24; 0.46]
Guo, Z, et al., 2013	89	75.00	13.82	89	73.43	12.31	baseline	0.00		0.12	[-0.17; 0.41]
Arving, C, et al., 2007	47	78.00	27.00	38	75.00	19.00	baseline	0.00		0.13	[-0.30; 0.55]
Rahmani, S, et al. 2015	12	34.72	13.22	12	33.33	7.11	baseline	0.00		0.13	[-0.67; 0.93]
Rodrigez, B, et al., 2014	8	95.83	11.79	7	90.48	25.20	baseline	0.00		0.26	[-0.76; 1.28]
Powell, CB, et al., 2008	21	22.20	5.40	43	19.70	6.00	baseline	0.00		0.42	[-0.10; 0.95]
Trask, PC, et al. 2003	25	71.50	22.00	23	58.20	29.25	baseline	0.00		0.51	[-0.07; 1.08]
Elyasi, F, et al., 2021 II.	20	2.50	1.10	20	1.90	0.90	baseline	0.00		0.59	[-0.05; 1.22]
Lu, Z, et al., 201	203	75.30	4.00	103	71.30	6.50	baseline	0.00		0.80	[0.56; 1.05]
Zhao, X, et al., 2021	52	58.02	3.41	51	54.01	4.12	baseline	0.00		1.05	[0.64; 1.47]
Antoni, MH, et al., 2006	92	788.15	133.08	107	855.60	126.82	followup	48.00		-0.52	[-0.80; -0.23]
Ferguson, RJ, et al., 2012	19	6.75	1.78	21	7.47	1.59	followup	8.00		-0.42	[-1.05; 0.21]
Antoni, MH, et al., 2006	92	832.88	119.31	107	873.38	114.05	followup	24.00		-0.35	[-0.63; -0.07]
Berglund, G. et al. 2007	39	77.00	23.70	150	83.50	23.70	followup	48.00		-0.27	[-0.63; 0.08]
McLachlan, SA, et al., 2001	296	42.00	23.70	154	47.50	17.90	followup	24.00		-0.25	[-0.45; -0.06]
Schofield, P, et al., 2013	55	65.03	28.36	53	71.29	28.69	followup	12.00		-0.22	[-0.60; 0.16]
Chan, et al. 2005	80	76.19	54.76	75	87.23	54.76	followup	72.00		-0.20	[-0.52; 0.12]
Chan, et al. 2005	80	65.58	54.76	75	75.11	54.76	followup	24.00		-0.17	[-0.49; 0.14]
Chan, et al. 2005	80	76.62	54.76	75	85.06	54.76	followup	60.00		-0.15	[-0.47; 0.16]
Chan, et al. 2005	80	61.47	54.76	75	69.26	54.76	followup	12.00		-0.14	[-0.46; 0.17]
Serfaty M, et al., 2018	20	18.70	5.30	22	19.40	6.20	followup	12.00		-0.12	[-0.72; 0.49]
Schofield, P, et al., 2013	55	68.28	26.22	53	70.58	26.38	followup	8.00		-0.09	[-0.46; 0.29]
Klinkhammer-Schalke, M et al., 2012	100	78.90	27.54	100	81.14	24.07	followup	48.00		-0.09	[-0.36; 0.19]
Rodrigez, B, et al., 2014	8	93.33	14.91	7	94.44	9.62	followup	2.00		-0.08	[-1.10; 0.93]
Chan, et al. 2005	80	80.52	54.76	75	83.77	54.76	followup	48.00		-0.06	[-0.37; 0.26]
Braeken APB, et al. 2013	136	86.99	20.73	144	87.55	19.10	followup	48.00		-0.03	[-0.26; 0.21]
Klinkhammer-Schalke, M et al., 2012	100	66.00	33.00	100	66.74	31.76	followup	12.00		-0.02	[-0.30; 0.25]
Klafke, N, et al., 2019	120	54.50	29.60	113	55.10	29.50	followup	24.00		-0.02	[-0.28; 0.24]
Elyasi, F, et al., 2021 I.	15	1.80	0.90	15	1.80	0.90	followup	24.00		0.00	[-0.72; 0.72]
Qiu, H, et al., 2018 II.	98	17.98	8.92	196	17.98	8.52	followup	4.00		0.00	[-0.24; 0.24]
Rahmani, S, et al. 2015	12	12.00	54.16	12	12.00	34.72	followup	16.00		0.00	[-0.80; 0.80]
Seliniotaki, T, et al., 2021	27	74.60	35.90	26	74.60	29.30	followup	8.00		0.00	[-0.54; 0.54]
Qiu, H, et al., 2018 II.	98	18.97	8.92	196	18.75	9.46	followup	24.00		0.02	[-0.22; 0.27]
Klinkhammer-Schalke, M et al., 2012	100	72.20	28.78	100	71.46	29.03	followup	24.00		0.03	[-0.25; 0.30]
Qin, X, et al., 2017	50	80.24	13.46	50	79.67	13.13	followup	2.00		0.04	[-0.35; 0.43]
Arving, C, et al., 2007	47	76.00	25.00	38	75.00	19.00	followup	12.00		0.04	[-0.38; 0.47]
Chan, et al. 2005	80	80.95	54.76	75	77.92	54.76	followup	36.00		0.06	[-0.26; 0.37]
Braeken APB, et al. 2013	136	83.46	23.57	144	81.81	22.37	followup	12.00		0.07	[-0.16; 0.31]
Qiu, H, et al., 2018 II.	98	18.77	8.03	196	18.25	5.05	followup	12.00		0.08	[-0.16; 0.33]
Klafke, N, et al., 2019	120	62.10	29.40	113	59.70	27.60	followup	12.00		0.08	[-0.17; 0.34]
Arving, C, et al., 2007	47	81.00	22.00	38	79.00	25.00	followup	4.00		0.08	[-0.34; 0.51]
Zhao, X, et al., 2021	52	58.62	6.31	51	58.02	3.21	followup	12.00		0.12	[-0.27; 0.51]
Arving, C, et al., 2007	47	83.00	21.00	38	80.00	26.00	followup	24.00		0.13	[-0.30; 0.56]
Klinkhammer-Schalke, M et al., 2012	100	77.91	27.05	100	73.69	30.77	followup	36.00		0.15	[-0.13; 0.42]
Ruiz-Vozmediano, J, et al.,2020	31	83.30	24.70	32	78.70	28.50	followup	24.00		0.17	[-0.32; 0.67]
Klafke, N, et al., 2019	120	69.10	27.90	113	63.60	26.60	followup	48.00		0.20	[-0.06; 0.46]
Qiu, H, et al., 2018 I.	98	20.01	9.15	196	17.98	8.52	followup	4.00		0.23	[-0.01; 0.47]
Serfaty M, et al., 2018	20	21.20	4.30	22	19.70	6.70	followup	18.00		0.26	[-0.35; 0.87]
Rodrigez, B, et al., 2014	8	95.83	11.79	7	90.48	25.20	followup	0.10		0.26	[-0.76; 1.28]
Guo, Z, et al., 2013	89	74.35	10.67	89	71.33	11.88	followup	2.00		0.27	[-0.03; 0.56]
Trask, PC, et al. 2003	25	86.10	23.50	23	79.40	20.62	followup	24.00		0.30	[-0.27; 0.87]
Li, X, et al., 2017	102	15.45	2.67	108	14.52	3.28	followup	12.00		0.31	[0.04; 0.58]
Powell, CB, et al., 2008	21	21.30	5.60	43	19.10	7.30	followup	12.00		0.32	[-0.21; 0.84]
Qiu, H, et al., 2018 I.	98	21.75	8.70	196	18.75	9.46	followup	24.00		0.32	[0.08; 0.57]
Hernandez, EG, et al. 2018	28	18.82	5.79	28	16.40	5.07	followup	24.00		0.44	[-0.09; 0.97]
Serfaty M, et al., 2018	20	19.50	7.20	22	16.00	7.60	followup	24.00		0.46	[-0.15; 1.08]
Hernandez, EG, et al. 2018	28	19.23	5.51	28	16.60	5.39	followup	8.00		0.48	[-0.06; 1.01]
Qiu, H, et al., 2018 I.	98	21.39	8.03	196	18.25	5.05	followup	12.00		0.50	[0.26; 0.75]
Liu, T, et al., 2019	49	65.81	18.49	53	56.11	17.08	followup	9.00		0.54	[0.15; 0.94]
Zhou, J, et al., 2020	59	72.34	11.43	59	65.87	10.26	followup	2.00		0.59	[0.22; 0.96]
Trask, PC, et al. 2003	25	85.90	24.00	23	70.10	27.34	followup	8.00		0.61	[0.03; 1.19]
Zhao,X, et al. 2015	62	8.19	2.10	62	6.87	2.21	followup	0.30		0.61	[0.25; 0.97]
Elyasi, F, et al., 2021 II.	20	2.40	1.00	20	1.80	0.90	followup	24.00		0.62	[-0.02; 1.25]
Chen, et al., 2017	58	47.60	7.50	65	42.60	7.20	followup	8.00		0.68	[0.31; 1.04]
Chen, et al., 2017	58	46.80	7.60	65	41.50	7.50	followup	24.00		0.70	[0.33; 1.06]
Liu, T, et al., 2019	49	74.35	17.68	53	62.05	14.34	followup	12.00		0.76	[0.36; 1.16]
Lu, Z, et al., 201	203	76.10	5.20	103	71.10	8.20	followup	9.00		0.78	[0.54; 1.03]
van der Meulen, IC, et al., 2013	88	88.50	3.20	91	85.10	3.00	followup	48.00		1.09	[0.78; 1.41]
Baoyindeligeer, L.Z. et al. 2020	65	88.33	12.38	65	71.36	13.84	followup	2.00		1.28	[0.91; 1.66]
Rahmani, S, et al. 2015	12	51.38	11.14	12	29.16	16.09	followup	8.00		1.55	[0.62; 2.48]
Fang, P, et al.,2020	60	65.47	6.98	60	49.45	5.65	followup	4.00		2.51	[2.03; 2.99]
Rodríguez Vega, B, et al., 2010	39	49.04	3.32	33	29.42	3.62	followup	12.00	>	5.61	[4.56; 6.66]
Rodríguez Vega, B, et al., 2010	39	61.86	3.32	33	34.47	3.62	followup	24.00	>	7.83	[6.44; 9.22]
Random effect	6635			7199						0.32	[0.10; 0.54]
Prediction interval											[-1.11; 1.76]
telephone											
Heiney, SP, et al., 2003	33	5.60	2.00	33	5.90	1.90	baseline	0.00		-0.15	[-0.64; 0.33]
Kim, SH, et al., 2021	47	73.70	81.90	47	75.50	22.00	baseline	0.00		-0.03	[-0.43; 0.37]
Dirksen, S. et al, 2007	34	22.10	4.90	38	22.20	6.10	baseline	0.00		-0.02	[-0.48; 0.44]
Girgis, A, et al., 2009 II.	120	80.80	25.60	117	79.50	25.10	baseline	0.00		0.05	[-0.20; 0.31]
Yun et al., 2017	134	75.80	26.80	72	73.10	23.40	baseline	0.00		0.10	[-0.18; 0.39]
Thomas, ML, et al., 2012	64	21.10	5.40	88	19.00	6.30	baseline	0.00		0.35	[0.03; 0.68]
Heiney, SP, et al., 2003	33	5.80	2.00	33	6.55	1.80	followup	16.00		-0.39	[-0.88; 0.10]
Heiney, SP, et al., 2003	33	5.80	2.00	33	5.90	1.90	followup	6.00		-0.05	[-0.53; 0.43]
Girgis, A, et al., 2009 II.	120	89.20	20.30	117	89.90	17.00	followup	12.00		-0.04	[-0.29; 0.22]
Kim, SH, et al., 2021	47	80.30	17.60	47	80.40	18.50	followup	20.00		-0.01	[-0.41; 0.40]
Girgis, A, et al., 2009 II.	120	91.90	17.60	117	91.90	17.40	followup	24.00		0.00	[-0.25; 0.25]
Thomas, ML, et al., 2012	64	20.50	6.10	88	19.00	6.40	followup	12.00		0.24	[-0.09; 0.56]
Yun et al., 2017	134	85.30	19.50	72	78.20	22.40	followup	48.00		0.34	[0.06; 0.63]
Kim, SH, et al., 2021	47	81.90	15.20	47	74.60	23.00	followup	8.00		0.37	[-0.04; 0.78]
Dirksen, S. et al, 2007	34	23.30	3.90	38	21.40	5.90	followup	10.00		0.37	[-0.10; 0.84]
Random effect	1064			987						0.17	[-0.19; 0.53]
Prediction interval											[-1.50; 1.84]
online											
Peng, L, et al., 2022	28	76.79	15.27	29	81.61	16.87	baseline	0.00		-0.30	[-0.82; 0.23]
Wu, Q, et al., 2021	43	53.77	5.51	43	54.34	5.53	baseline	0.00		-0.10	[-0.53; 0.32]
Cengiz, HO, et al., 2023	32	20.16	5.97	33	19.13	5.49	baseline	0.00		0.18	[-0.31; 0.66]
Penedo, FJ, et al., 2020	95	-19.85	4.09	97	-21.16	4.24	baseline	0.00		0.31	[0.03; 0.60]
Beatty, L. et al. 2015	30	57.26	5.49	30	66.56	5.41	followup	6.00		-1.68	[-2.28; -1.09]
Peng, L, et al., 2022	28	80.36	20.09	29	82.18	19.38	followup	0.10		-0.09	[-0.61; 0.43]
Peng, L, et al., 2022	28	79.17	16.49	29	79.89	20.11	followup	4.00		-0.04	[-0.56; 0.48]
Cengiz, HO, et al., 2023	32	20.36	5.38	33	19.06	5.74	followup	8.00		0.23	[-0.26; 0.72]
Penedo, FJ, et al., 2020	95	-20.74	4.29	97	-22.05	4.43	followup	48.00		0.30	[0.01; 0.58]
Penedo, FJ, et al., 2020	95	-20.61	4.19	97	-21.91	4.33	followup	24.00		0.30	[0.02; 0.59]
Beatty, L. et al. 2015	30	77.87	4.62	30	75.08	4.61	followup	13.03		0.60	[0.08; 1.11]
Beatty, L. et al. 2015	30	87.98	4.26	30	83.84	4.75	followup	26.07		0.91	[0.37; 1.44]
Wu, Q, et al., 2021	43	81.65	8.30	43	72.36	7.51	followup	12.00		1.16	[0.70; 1.62]
Random effect	609			620						0.25	[-0.28; 0.78]
Prediction interval											[-1.61; 2.11]
**Random effect**	**8308**			**8806**						**0.29**	**[0.07; 0.52]**
**Prediction interval**											**[-1.12; 1.71]**

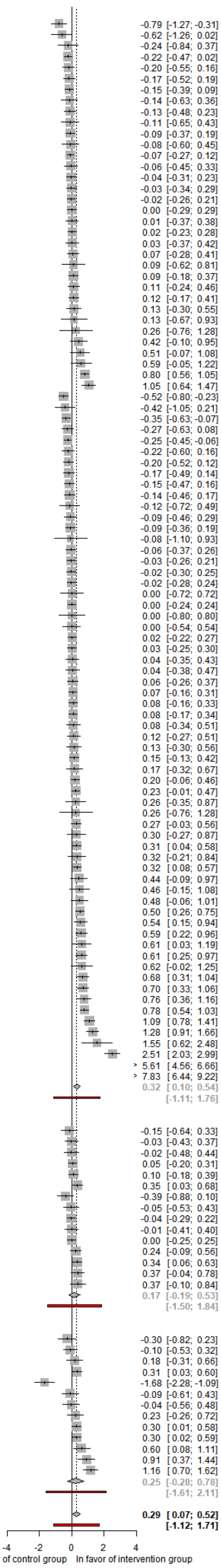

Figure S14.1. Forest plot represents the difference between the intervention vs. control group in the Social QoL domain with the environment subgroups as predicted at week 0 (post-intervention). SMD - Standardized mean difference, CI - confidence interval

Figure S14.2.T12

Study	Experimental Patient N	Experimental Mean	Experimental SD	Control Patient N	Control Mean	Control SD	follow-up	Follow-up time	SMD	95%-CI
face to face										
Rodríguez Vega, B, et al., 2010	39	3.31	3.32	33	6.08	3.62	baseline	0.00	-0.79	[-1.27; -0.31]
Ferguson, RJ, et al., 2012	19	6.26	2.05	21	7.31	1.21	baseline	0.00	-0.62	[-1.26; 0.02]
Serfaty M, et al., 2018	20	16.40	6.10	22	18.10	7.80	baseline	0.00	-0.24	[-0.84; 0.37]
Qiu, H, et al., 2018 II.	98	17.84	5.10	196	18.85	4.22	baseline	0.00	-0.22	[-0.47; 0.02]
Chen, et al., 2017	58	58.30	6.20	65	59.60	6.90	baseline	0.00	-0.20	[-0.55; 0.16]
Berglund, G. et al. 2007	39	80.30	22.60	150	84.00	22.10	baseline	0.00	-0.17	[-0.52; 0.19]
Qiu, H, et al., 2018 I.	98	18.13	5.70	196	18.85	4.22	baseline	0.00	-0.15	[-0.39; 0.09]
Ruiz-Vozmediano, J, et al.,2020	31	69.40	29.80	32	73.40	28.40	baseline	0.00	-0.14	[-0.63; 0.36]
Fang, P, et al.,2020	60	48.50	4.11	60	49.08	5.01	baseline	0.00	-0.13	[-0.48; 0.23]
Seliniotaki, T, et al., 2021	27	74.40	29.90	26	77.70	29.30	baseline	0.00	-0.11	[-0.65; 0.43]
Antoni, MH, et al., 2006	92	877.61	158.55	107	891.17	152.16	baseline	0.00	-0.09	[-0.37; 0.19]
Hernandez, EG, et al. 2018	28	17.11	6.05	28	17.56	5.46	baseline	0.00	-0.08	[-0.60; 0.45]
McLachlan, SA, et al., 2001	296	41.20	22.60	154	42.80	18.70	baseline	0.00	-0.07	[-0.27; 0.12]
Liu, T, et al., 2019	49	63.77	24.95	53	65.22	23.71	baseline	0.00	-0.06	[-0.45; 0.33]
Li, X, et al., 2017	102	13.58	2.23	108	13.67	2.21	baseline	0.00	-0.04	[-0.31; 0.23]
Chan, et al. 2005	80	54.76	60.02	75	56.28	54.76	baseline	0.00	-0.03	[-0.34; 0.29]
Braeken APB, et al. 2013	136	84.94	21.44	144	85.46	22.52	baseline	0.00	-0.02	[-0.26; 0.21]
van der Meulen, IC, et al., 2013	88	83.10	2.50	91	83.10	2.50	baseline	0.00	0.00	[-0.29; 0.29]
Schofield, P, et al., 2013	55	70.90	26.77	53	70.75	26.72	baseline	0.00	0.01	[-0.37; 0.38]
Klafke, N, et al., 2019	120	59.30	31.30	113	58.60	29.60	baseline	0.00	0.02	[-0.23; 0.28]
Qin, X, et al., 2017	50	77.68	15.31	50	77.26	16.04	baseline	0.00	0.03	[-0.37; 0.42]
Baoyindeligeer, L.Z. et al. 2020	65	66.59	14.95	65	65.59	15.24	baseline	0.00	0.07	[-0.28; 0.41]
Elyasi, F, et al., 2021 I.	15	2.00	1.20	15	1.90	0.90	baseline	0.00	0.09	[-0.62; 0.81]
Klinkhammer-Schalke, M et al., 2012	100	71.21	33.50	100	67.99	34.25	baseline	0.00	0.09	[-0.18; 0.37]
Zhao,X, et al. 2015	62	7.01	2.18	62	6.76	2.39	baseline	0.00	0.11	[-0.24; 0.46]
Guo, Z, et al., 2013	89	75.00	13.82	89	73.43	12.31	baseline	0.00	0.12	[-0.17; 0.41]
Arving, C, et al., 2007	47	78.00	27.00	38	75.00	19.00	baseline	0.00	0.13	[-0.30; 0.55]
Rahmani, S, et al. 2015	12	34.72	13.22	12	33.33	7.11	baseline	0.00	0.13	[-0.67; 0.93]
Rodrigez, B, et al., 2014	8	95.83	11.79	7	90.48	25.20	baseline	0.00	0.26	[-0.76; 1.28]
Powell, CB, et al., 2008	21	22.20	5.40	43	19.70	6.00	baseline	0.00	0.42	[-0.10; 0.95]
Trask, PC, et al. 2003	25	71.50	22.00	23	58.20	29.25	baseline	0.00	0.51	[-0.07; 1.08]
Elyasi, F, et al., 2021 II.	20	2.50	1.10	20	1.90	0.90	baseline	0.00	0.59	[-0.05; 1.22]
Lu, Z, et al., 201	203	75.30	4.00	103	71.30	6.50	baseline	0.00	0.80	[0.56; 1.05]
Zhao, X, et al., 2021	52	58.02	3.41	51	54.01	4.12	baseline	0.00	1.05	[0.64; 1.47]
Antoni, MH, et al., 2006	92	788.15	133.08	107	855.60	126.82	followup	48.00	-0.52	[-0.80; -0.23]
Ferguson, RJ, et al., 2012	19	6.75	1.78	21	7.47	1.59	followup	8.00	-0.42	[-1.05; 0.21]
Antoni, MH, et al., 2006	92	832.88	119.31	107	873.38	114.05	followup	24.00	-0.35	[-0.63; -0.07]
Berglund, G. et al. 2007	39	77.00	23.70	150	83.50	23.70	followup	48.00	-0.27	[-0.63; 0.08]
McLachlan, SA, et al., 2001	296	42.00	23.70	154	47.50	17.90	followup	24.00	-0.25	[-0.45; -0.06]
Schofield, P, et al., 2013	55	65.03	28.36	53	71.29	28.69	followup	12.00	-0.22	[-0.60; 0.16]
Chan, et al. 2005	80	76.19	54.76	75	87.23	54.76	followup	72.00	-0.20	[-0.52; 0.12]
Chan, et al. 2005	80	65.58	54.76	75	75.11	54.76	followup	24.00	-0.17	[-0.49; 0.14]
Chan, et al. 2005	80	76.62	54.76	75	85.06	54.76	followup	60.00	-0.15	[-0.47; 0.16]
Chan, et al. 2005	80	61.47	54.76	75	69.26	54.76	followup	12.00	-0.14	[-0.46; 0.17]
Serfaty M, et al., 2018	20	18.70	5.30	22	19.40	6.20	followup	12.00	-0.12	[-0.72; 0.49]
Schofield, P, et al., 2013	55	68.28	26.22	53	70.58	26.38	followup	8.00	-0.09	[-0.46; 0.29]
Klinkhammer-Schalke, M et al., 2012	100	78.90	27.54	100	81.14	24.07	followup	48.00	-0.09	[-0.36; 0.19]
Rodrigez, B, et al., 2014	8	93.33	14.91	7	94.44	9.62	followup	2.00	-0.08	[-1.10; 0.93]
Chan, et al. 2005	80	80.52	54.76	75	83.77	54.76	followup	48.00	-0.06	[-0.37; 0.26]
Braeken APB, et al. 2013	136	86.99	20.73	144	87.55	19.10	followup	48.00	-0.03	[-0.26; 0.21]
Klinkhammer-Schalke, M et al., 2012	100	66.00	33.00	100	66.74	31.76	followup	12.00	-0.02	[-0.30; 0.25]
Klafke, N, et al., 2019	120	54.50	29.60	113	55.10	29.50	followup	24.00	-0.02	[-0.28; 0.24]
Elyasi, F, et al., 2021 I.	15	1.80	0.90	15	1.80	0.90	followup	24.00	0.00	[-0.72; 0.72]
Qiu, H, et al., 2018 II.	98	17.98	8.92	196	17.98	8.52	followup	4.00	0.00	[-0.24; 0.24]
Rahmani, S, et al. 2015	12	12.00	54.16	12	12.00	34.72	followup	16.00	0.00	[-0.80; 0.80]
Seliniotaki, T, et al., 2021	27	74.60	35.90	26	74.60	29.30	followup	8.00	0.00	[-0.54; 0.54]
Qiu, H, et al., 2018 II.	98	18.97	8.92	196	18.75	9.46	followup	24.00	0.02	[-0.22; 0.27]
Klinkhammer-Schalke, M et al., 2012	100	72.20	28.78	100	71.46	29.03	followup	24.00	0.03	[-0.25; 0.30]
Qin, X, et al., 2017	50	80.24	13.46	50	79.67	13.13	followup	2.00	0.04	[-0.35; 0.43]
Arving, C, et al., 2007	47	76.00	25.00	38	75.00	19.00	followup	12.00	0.04	[-0.38; 0.47]
Chan, et al. 2005	80	80.95	54.76	75	77.92	54.76	followup	36.00	0.06	[-0.26; 0.37]
Braeken APB, et al. 2013	136	83.46	23.57	144	81.81	22.37	followup	12.00	0.07	[-0.16; 0.31]
Qiu, H, et al., 2018 II.	98	18.77	8.03	196	18.25	5.05	followup	12.00	0.08	[-0.16; 0.33]
Klafke, N, et al., 2019	120	62.10	29.40	113	59.70	27.60	followup	12.00	0.08	[-0.17; 0.34]
Arving, C, et al., 2007	47	81.00	22.00	38	79.00	25.00	followup	4.00	0.08	[-0.34; 0.51]
Zhao, X, et al., 2021	52	58.62	6.31	51	58.02	3.21	followup	12.00	0.12	[-0.27; 0.51]
Arving, C, et al., 2007	47	83.00	21.00	38	80.00	26.00	followup	24.00	0.13	[-0.30; 0.56]
Klinkhammer-Schalke, M et al., 2012	100	77.91	27.05	100	73.69	30.77	followup	36.00	0.15	[-0.13; 0.42]
Ruiz-Vozmediano, J, et al.,2020	31	83.30	24.70	32	78.70	28.50	followup	24.00	0.17	[-0.32; 0.67]
Klafke, N, et al., 2019	120	69.10	27.90	113	63.60	26.60	followup	48.00	0.20	[-0.06; 0.46]
Qiu, H, et al., 2018 I.	98	20.01	9.15	196	17.98	8.52	followup	4.00	0.23	[-0.01; 0.47]
Serfaty M, et al., 2018	20	21.20	4.30	22	19.70	6.70	followup	18.00	0.26	[-0.35; 0.87]
Rodrigez, B, et al., 2014	8	95.83	11.79	7	90.48	25.20	followup	0.10	0.26	[-0.76; 1.28]
Guo, Z, et al., 2013	89	74.35	10.67	89	71.33	11.88	followup	2.00	0.27	[-0.03; 0.56]
Trask, PC, et al. 2003	25	86.10	23.50	23	79.40	20.62	followup	24.00	0.30	[-0.27; 0.87]
Li, X, et al., 2017	102	15.45	2.67	108	14.52	3.28	followup	12.00	0.31	[0.04; 0.58]
Powell, CB, et al., 2008	21	21.30	5.60	43	19.10	7.30	followup	12.00	0.32	[-0.21; 0.84]
Qiu, H, et al., 2018 I.	98	21.75	8.70	196	18.75	9.46	followup	24.00	0.32	[0.08; 0.57]
Hernandez, EG, et al. 2018	28	18.82	5.79	28	16.40	5.07	followup	24.00	0.44	[-0.09; 0.97]
Serfaty M, et al., 2018	20	19.50	7.20	22	16.00	7.60	followup	24.00	0.46	[-0.15; 1.08]
Hernandez, EG, et al. 2018	28	19.23	5.51	28	16.60	5.39	followup	8.00	0.48	[-0.06; 1.01]
Qiu, H, et al., 2018 I.	98	21.39	8.03	196	18.25	5.05	followup	12.00	0.50	[0.26; 0.75]
Liu, T, et al., 2019	49	65.81	18.49	53	56.11	17.08	followup	9.00	0.54	[0.15; 0.94]
Zhou, J, et al., 2020	59	72.34	11.43	59	65.87	10.26	followup	2.00	0.59	[0.22; 0.96]
Trask, PC, et al. 2003	25	85.90	24.00	23	70.10	27.34	followup	8.00	0.61	[0.03; 1.19]
Zhao,X, et al. 2015	62	8.19	2.10	62	6.87	2.21	followup	0.30	0.61	[0.25; 0.97]
Elyasi, F, et al., 2021 II.	20	2.40	1.00	20	1.80	0.90	followup	24.00	0.62	[-0.02; 1.25]
Chen, et al., 2017	58	47.60	7.50	65	42.60	7.20	followup	8.00	0.68	[0.31; 1.04]
Chen, et al., 2017	58	46.80	7.60	65	41.50	7.50	followup	24.00	0.70	[0.33; 1.06]
Liu, T, et al., 2019	49	74.35	17.68	53	62.05	14.34	followup	12.00	0.76	[0.36; 1.16]
Lu, Z, et al., 201	203	76.10	5.20	103	71.10	8.20	followup	9.00	0.78	[0.54; 1.03]
van der Meulen, IC, et al., 2013	88	88.50	3.20	91	85.10	3.00	followup	48.00	1.09	[0.78; 1.41]
Baoyindeligeer, L.Z. et al. 2020	65	88.33	12.38	65	71.36	13.84	followup	2.00	1.28	[0.91; 1.66]
Rahmani, S, et al. 2015	12	51.38	11.14	12	29.16	16.09	followup	8.00	1.55	[0.62; 2.48]
Fang, P, et al.,2020	60	65.47	6.98	60	49.45	5.65	followup	4.00	2.51	[2.03; 2.99]
Rodríguez Vega, B, et al., 2010	39	49.04	3.32	33	29.42	3.62	followup	12.00	5.61	[4.56; 6.66]
Rodríguez Vega, B, et al., 2010	39	61.86	3.32	33	34.47	3.62	followup	24.00	7.83	[6.44; 9.22]
Random effect	6635			7199					0.41	[0.11; 0.72]
Prediction interval										[-1.03; 1.85]
telephone										
Heiney, SP, et al., 2003	33	5.60	2.00	33	5.90	1.90	baseline	0.00	-0.15	[-0.64; 0.33]
Kim, SH, et al., 2021	47	73.70	81.90	47	75.50	22.00	baseline	0.00	-0.03	[-0.43; 0.37]
Dirksen, S. et al, 2007	34	22.10	4.90	38	22.20	6.10	baseline	0.00	-0.02	[-0.48; 0.44]
Girgis, A, et al., 2009 II.	120	80.80	25.60	117	79.50	25.10	baseline	0.00	0.05	[-0.20; 0.31]
Yun et al., 2017	134	75.80	26.80	72	73.10	23.40	baseline	0.00	0.10	[-0.18; 0.39]
Thomas, ML, et al., 2012	64	21.10	5.40	88	19.00	6.30	baseline	0.00	0.35	[0.03; 0.68]
Heiney, SP, et al., 2003	33	5.80	2.00	33	6.55	1.80	followup	16.00	-0.39	[-0.88; 0.10]
Heiney, SP, et al., 2003	33	5.80	2.00	33	5.90	1.90	followup	6.00	-0.05	[-0.53; 0.43]
Girgis, A, et al., 2009 II.	120	89.20	20.30	117	89.90	17.00	followup	12.00	-0.04	[-0.29; 0.22]
Kim, SH, et al., 2021	47	80.30	17.60	47	80.40	18.50	followup	20.00	-0.01	[-0.41; 0.40]
Girgis, A, et al., 2009 II.	120	91.90	17.60	117	91.90	17.40	followup	24.00	0.00	[-0.25; 0.25]
Thomas, ML, et al., 2012	64	20.50	6.10	88	19.00	6.40	followup	12.00	0.24	[-0.09; 0.56]
Yun et al., 2017	134	85.30	19.50	72	78.20	22.40	followup	48.00	0.34	[0.06; 0.63]
Kim, SH, et al., 2021	47	81.90	15.20	47	74.60	23.00	followup	8.00	0.37	[-0.04; 0.78]
Dirksen, S. et al, 2007	34	23.30	3.90	38	21.40	5.90	followup	10.00	0.37	[-0.10; 0.84]
Random effect	1064			987					0.26	[-0.02; 0.55]
Prediction interval										[-1.49; 2.02]
online										
Peng, L, et al., 2022	28	76.79	15.27	29	81.61	16.87	baseline	0.00	-0.30	[-0.82; 0.23]
Wu, Q, et al., 2021	43	53.77	5.51	43	54.34	5.53	baseline	0.00	-0.10	[-0.53; 0.32]
Cengiz, HO, et al., 2023	32	20.16	5.97	33	19.13	5.49	baseline	0.00	0.18	[-0.31; 0.66]
Penedo, FJ, et al., 2020	95	-19.85	4.09	97	-21.16	4.24	baseline	0.00	0.31	[0.03; 0.60]
Beatty, L. et al. 2015	30	57.26	5.49	30	66.56	5.41	followup	6.00	-1.68	[-2.28; -1.09]
Peng, L, et al., 2022	28	80.36	20.09	29	82.18	19.38	followup	0.10	-0.09	[-0.61; 0.43]
Peng, L, et al., 2022	28	79.17	16.49	29	79.89	20.11	followup	4.00	-0.04	[-0.56; 0.48]
Cengiz, HO, et al., 2023	32	20.36	5.38	33	19.06	5.74	followup	8.00	0.23	[-0.26; 0.72]
Penedo, FJ, et al., 2020	95	-20.74	4.29	97	-22.05	4.43	followup	48.00	0.30	[0.01; 0.58]
Penedo, FJ, et al., 2020	95	-20.61	4.19	97	-21.91	4.33	followup	24.00	0.30	[0.02; 0.59]
Beatty, L. et al. 2015	30	77.87	4.62	30	75.08	4.61	followup	13.03	0.60	[0.08; 1.11]
Beatty, L. et al. 2015	30	87.98	4.26	30	83.84	4.75	followup	26.07	0.91	[0.37; 1.44]
Wu, Q, et al., 2021	43	81.65	8.30	43	72.36	7.51	followup	12.00	1.16	[0.70; 1.62]
Random effect	609			620					0.34	[-0.07; 0.76]
Prediction interval										[-1.64; 2.32]
**Random effect**	**8308**			**8806**					**0.38**	**[0.12; 0.64]**
**Prediction interval**										**[-1.01; 1.78]**

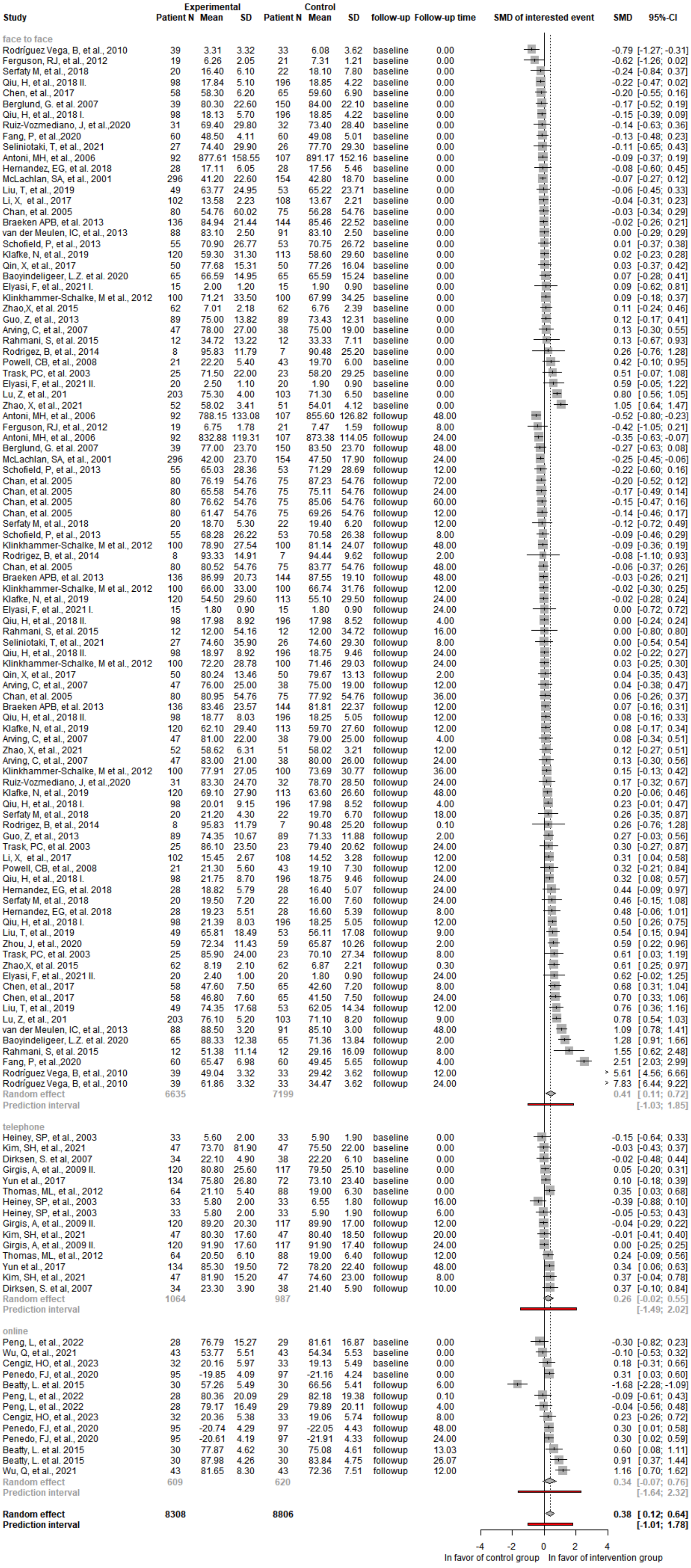

Figure S14.2. Forest plot represents the difference between the intervention vs. control group in the Social QoL domain with the environment subgroups as predicted at week 12 (post-intervention). SMD - Standardized mean difference, CI - confidence interval.

Figure S14.3.T24

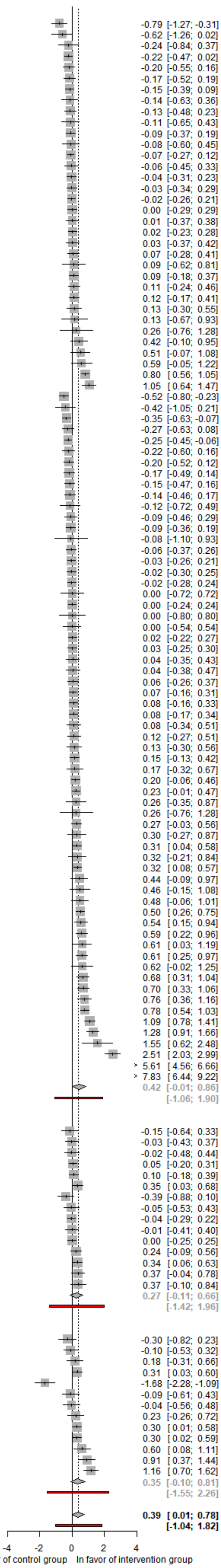

Study	Experimental Patient N	Experimental Mean	Experimental SD	Control Patient N	Control Mean	Control SD	follow-up	Follow-up time	SMD	95%-CI
**face to face**										
Rodríguez Vega, B, et al., 2010	39	3.31	3.32	33	6.08	3.62	baseline	0.00	-0.79	[-1.27; -0.31]
Ferguson, RJ, et al., 2012	19	6.26	2.05	21	7.31	1.21	baseline	0.00	-0.62	[-1.26; 0.02]
Serfaty M, et al., 2018	20	16.40	6.10	22	18.10	7.80	baseline	0.00	-0.24	[-0.84; 0.37]
Qiu, H, et al., 2018 II.	98	17.84	5.10	196	18.85	4.22	baseline	0.00	-0.22	[-0.47; 0.02]
Chen, et al., 2017	58	58.30	6.20	65	59.60	6.90	baseline	0.00	-0.20	[-0.55; 0.16]
Berglund, G. et al. 2007	39	80.30	22.60	150	84.00	22.10	baseline	0.00	-0.17	[-0.52; 0.19]
Qiu, H, et al., 2018 I.	98	18.13	5.70	196	18.85	4.22	baseline	0.00	-0.15	[-0.39; 0.09]
Ruiz-Vozmediano, J, et al.,2020	31	69.40	29.80	32	73.40	28.40	baseline	0.00	-0.14	[-0.63; 0.36]
Fang, P, et al.,2020	60	48.50	4.11	60	49.08	5.01	baseline	0.00	-0.13	[-0.48; 0.23]
Seliniotaki, T, et al., 2021	27	74.40	29.90	26	77.70	29.30	baseline	0.00	-0.11	[-0.65; 0.43]
Antoni, MH, et al., 2006	92	877.61	158.55	107	891.17	152.16	baseline	0.00	-0.09	[-0.37; 0.19]
Hernandez, EG, et al. 2018	28	17.11	6.05	28	17.56	5.46	baseline	0.00	-0.08	[-0.60; 0.45]
McLachlan, SA, et al., 2001	296	41.20	22.60	154	42.80	18.70	baseline	0.00	-0.07	[-0.27; 0.12]
Liu, T, et al., 2019	49	63.77	24.95	53	65.22	23.71	baseline	0.00	-0.06	[-0.45; 0.33]
Li, X, et al., 2017	102	13.58	2.23	108	13.67	2.21	baseline	0.00	-0.04	[-0.31; 0.23]
Chan, et al. 2005	80	54.76	60.02	75	56.28	54.76	baseline	0.00	-0.03	[-0.34; 0.29]
Braeken APB, et al. 2013	136	84.94	21.44	144	85.46	22.52	baseline	0.00	-0.02	[-0.26; 0.21]
van der Meulen, IC, et al., 2013	88	83.10	2.50	91	83.10	2.50	baseline	0.00	0.00	[-0.29; 0.29]
Schofield, P, et al., 2013	55	70.90	26.77	53	70.75	26.72	baseline	0.00	0.01	[-0.37; 0.38]
Klafke, N, et al., 2019	120	59.30	31.30	113	58.60	29.60	baseline	0.00	0.02	[-0.23; 0.28]
Qin, X, et al., 2017	50	77.68	15.31	50	77.26	16.04	baseline	0.00	0.03	[-0.37; 0.42]
Baoyindeligeer, L.Z. et al. 2020	65	66.59	14.95	65	65.59	15.24	baseline	0.00	0.07	[-0.28; 0.41]
Elyasi, F, et al., 2021 I.	15	2.00	1.20	15	1.90	0.90	baseline	0.00	0.09	[-0.62; 0.81]
Klinkhammer-Schalke, M et al., 2012	100	71.21	33.50	100	67.99	34.25	baseline	0.00	0.09	[-0.18; 0.37]
Zhao,X, et al. 2015	62	7.01	2.18	62	6.76	2.39	baseline	0.00	0.11	[-0.24; 0.46]
Guo, Z, et al., 2013	89	75.00	13.82	89	73.43	12.31	baseline	0.00	0.12	[-0.17; 0.41]
Arving, C, et al., 2007	47	78.00	27.00	38	75.00	19.00	baseline	0.00	0.13	[-0.30; 0.55]
Rahmani, S, et al. 2015	12	34.72	13.22	12	33.33	7.11	baseline	0.00	0.13	[-0.67; 0.93]
Rodrigez, B, et al., 2014	8	95.83	11.79	7	90.48	25.20	baseline	0.00	0.26	[-0.76; 1.28]
Powell, CB, et al., 2008	21	22.20	5.40	43	19.70	6.00	baseline	0.00	0.42	[-0.10; 0.95]
Trask, PC, et al. 2003	25	71.50	22.00	23	58.20	29.25	baseline	0.00	0.51	[-0.07; 1.08]
Elyasi, F, et al., 2021 II.	20	2.50	1.10	20	1.90	0.90	baseline	0.00	0.59	[-0.05; 1.22]
Lu, Z, et al., 201	203	75.30	4.00	103	71.30	6.50	baseline	0.00	0.80	[0.56; 1.05]
Zhao, X, et al., 2021	52	58.02	3.41	51	54.01	4.12	baseline	0.00	1.05	[0.64; 1.47]
Antoni, MH, et al., 2006	92	788.15	133.08	107	855.60	126.82	followup	48.00	-0.52	[-0.80; -0.23]
Ferguson, RJ, et al., 2012	19	6.75	1.78	21	7.47	1.59	followup	8.00	-0.42	[-1.05; 0.21]
Antoni, MH, et al., 2006	92	832.88	119.31	107	873.38	114.05	followup	24.00	-0.35	[-0.63; -0.07]
Berglund, G. et al. 2007	39	77.00	23.70	150	83.50	23.70	followup	48.00	-0.27	[-0.63; 0.08]
McLachlan, SA, et al., 2001	296	42.00	23.70	154	47.50	17.90	followup	24.00	-0.25	[-0.45; -0.06]
Schofield, P, et al., 2013	55	65.03	28.36	53	71.29	28.69	followup	12.00	-0.22	[-0.60; 0.16]
Chan, et al. 2005	80	76.19	54.76	75	87.23	54.76	followup	72.00	-0.20	[-0.52; 0.12]
Chan, et al. 2005	80	65.58	54.76	75	75.11	54.76	followup	24.00	-0.17	[-0.49; 0.14]
Chan, et al. 2005	80	76.62	54.76	75	85.06	54.76	followup	60.00	-0.15	[-0.47; 0.16]
Chan, et al. 2005	80	61.47	54.76	75	69.26	54.76	followup	12.00	-0.14	[-0.46; 0.17]
Serfaty M, et al., 2018	20	18.70	5.30	22	19.40	6.20	followup	12.00	-0.12	[-0.72; 0.49]
Schofield, P, et al., 2013	55	68.28	26.22	53	70.58	26.38	followup	8.00	-0.09	[-0.46; 0.29]
Klinkhammer-Schalke, M et al., 2012	100	78.90	27.54	100	81.14	24.07	followup	48.00	-0.09	[-0.36; 0.19]
Rodrigez, B, et al., 2014	8	93.33	14.91	7	94.44	9.62	followup	2.00	-0.08	[-1.10; 0.93]
Chan, et al. 2005	80	80.52	54.76	75	83.77	54.76	followup	48.00	-0.06	[-0.37; 0.26]
Braeken APB, et al. 2013	136	86.99	20.73	144	87.55	19.10	followup	48.00	-0.03	[-0.26; 0.21]
Klinkhammer-Schalke, M et al., 2012	100	66.00	33.00	100	66.74	31.76	followup	12.00	-0.02	[-0.30; 0.25]
Klafke, N, et al., 2019	120	54.50	29.60	113	55.10	29.50	followup	24.00	-0.02	[-0.28; 0.24]
Elyasi, F, et al., 2021 I.	15	1.80	0.90	15	1.80	0.90	followup	24.00	0.00	[-0.72; 0.72]
Qiu, H, et al., 2018 II.	98	17.98	8.92	196	17.98	8.52	followup	4.00	0.00	[-0.24; 0.24]
Rahmani, S, et al. 2015	12	12.00	54.16	12	12.00	34.72	followup	16.00	0.00	[-0.80; 0.80]
Seliniotaki, T, et al., 2021	27	74.60	35.90	26	74.60	29.30	followup	8.00	0.00	[-0.54; 0.54]
Qiu, H, et al., 2018 II.	98	18.97	8.92	196	18.75	9.46	followup	24.00	0.02	[-0.22; 0.27]
Klinkhammer-Schalke, M et al., 2012	100	72.20	28.78	100	71.46	29.03	followup	24.00	0.03	[-0.25; 0.30]
Qin, X, et al., 2017	50	80.24	13.46	50	79.67	13.13	followup	2.00	0.04	[-0.35; 0.43]
Arving, C, et al., 2007	47	76.00	25.00	38	75.00	19.00	followup	12.00	0.04	[-0.38; 0.47]
Chan, et al. 2005	80	80.95	54.76	75	77.92	54.76	followup	36.00	0.06	[-0.26; 0.37]
Braeken APB, et al. 2013	136	83.46	23.57	144	81.81	22.37	followup	12.00	0.07	[-0.16; 0.31]
Qiu, H, et al., 2018 II.	98	18.77	8.03	196	18.25	5.05	followup	12.00	0.08	[-0.16; 0.33]
Klafke, N, et al., 2019	120	62.10	29.40	113	59.70	27.60	followup	12.00	0.08	[-0.17; 0.34]
Arving, C, et al., 2007	47	81.00	22.00	38	79.00	25.00	followup	4.00	0.08	[-0.34; 0.51]
Zhao, X, et al., 2021	52	58.62	6.31	51	58.02	3.21	followup	12.00	0.12	[-0.27; 0.51]
Arving, C, et al., 2007	47	83.00	21.00	38	80.00	26.00	followup	24.00	0.13	[-0.30; 0.56]
Klinkhammer-Schalke, M et al., 2012	100	77.91	27.05	100	73.69	30.77	followup	36.00	0.15	[-0.13; 0.42]
Ruiz-Vozmediano, J, et al.,2020	31	83.30	24.70	32	78.70	28.50	followup	24.00	0.17	[-0.32; 0.67]
Klafke, N, et al., 2019	120	69.10	27.90	113	63.60	26.60	followup	48.00	0.20	[-0.06; 0.46]
Qiu, H, et al., 2018 I.	98	20.01	9.15	196	17.98	8.52	followup	4.00	0.23	[-0.01; 0.47]
Serfaty M, et al., 2018	20	21.20	4.30	22	19.70	6.70	followup	18.00	0.26	[-0.35; 0.87]
Rodrigez, B, et al., 2014	8	95.83	11.79	7	90.48	25.20	followup	0.10	0.26	[-0.76; 1.28]
Guo, Z, et al., 2013	89	74.35	10.67	89	71.33	11.88	followup	2.00	0.27	[-0.03; 0.56]
Trask, PC, et al. 2003	25	86.10	23.50	23	79.40	20.62	followup	24.00	0.30	[-0.27; 0.87]
Li, X, et al., 2017	102	15.45	2.67	108	14.52	3.28	followup	12.00	0.31	[0.04; 0.58]
Powell, CB, et al., 2008	21	21.30	5.60	43	19.10	7.30	followup	12.00	0.32	[-0.21; 0.84]
Qiu, H, et al., 2018 I.	98	21.75	8.70	196	18.75	9.46	followup	24.00	0.32	[0.08; 0.57]
Hernandez, EG, et al. 2018	28	18.82	5.79	28	16.40	5.07	followup	24.00	0.44	[-0.09; 0.97]
Serfaty M, et al., 2018	20	19.50	7.20	22	16.00	7.60	followup	24.00	0.46	[-0.15; 1.08]
Hernandez, EG, et al. 2018	28	19.23	5.51	28	16.60	5.39	followup	8.00	0.48	[-0.06; 1.01]
Qiu, H, et al., 2018 I.	98	21.39	8.03	196	18.25	5.05	followup	12.00	0.50	[0.26; 0.75]
Liu, T, et al., 2019	49	65.81	18.49	53	56.11	17.08	followup	9.00	0.54	[0.15; 0.94]
Zhou, J, et al., 2020	59	72.34	11.43	59	65.87	10.26	followup	2.00	0.59	[0.22; 0.96]
Trask, PC, et al. 2003	25	85.90	24.00	23	70.10	27.34	followup	8.00	0.61	[0.03; 1.19]
Zhao,X, et al. 2015	62	8.19	2.10	62	6.87	2.21	followup	0.30	0.61	[0.25; 0.97]
Elyasi, F, et al., 2021 II.	20	2.40	1.00	20	1.80	0.90	followup	24.00	0.62	[-0.02; 1.25]
Chen, et al., 2017	58	47.60	7.50	65	42.60	7.20	followup	8.00	0.68	[0.31; 1.04]
Chen, et al., 2017	58	46.80	7.60	65	41.50	7.50	followup	24.00	0.70	[0.33; 1.06]
Liu, T, et al., 2019	49	74.35	17.68	53	62.05	14.34	followup	12.00	0.76	[0.36; 1.16]
Lu, Z, et al., 201	203	76.10	5.20	103	71.10	8.20	followup	9.00	0.78	[0.54; 1.03]
van der Meulen, IC, et al., 2013	88	88.50	3.20	91	85.10	3.00	followup	48.00	1.09	[0.78; 1.41]
Baoyindeligeer, L.Z. et al. 2020	65	88.33	12.38	65	71.36	13.84	followup	2.00	1.28	[0.91; 1.66]
Rahmani, S, et al. 2015	12	51.38	11.14	12	29.16	16.09	followup	8.00	1.55	[0.62; 2.48]
Fang, P, et al.,2020	60	65.47	6.98	60	49.45	5.65	followup	4.00	2.51	[2.03; 2.99]
Rodríguez Vega, B, et al., 2010	39	49.04	3.32	33	29.42	3.62	followup	12.00	5.61	[4.56; 6.66]
Rodríguez Vega, B, et al., 2010	39	61.86	3.32	33	34.47	3.62	followup	24.00	7.83	[6.44; 9.22]
Random effect	6635			7199					0.42	[-0.01; 0.86]
Prediction interval										[-1.06; 1.90]
**telephone**										
Heiney, SP, et al., 2003	33	5.60	2.00	33	5.90	1.90	baseline	0.00	-0.15	[-0.64; 0.33]
Kim, SH, et al., 2021	47	73.70	81.90	47	75.50	22.00	baseline	0.00	-0.03	[-0.43; 0.37]
Dirksen, S. et al, 2007	34	22.10	4.90	38	22.20	6.10	baseline	0.00	-0.02	[-0.48; 0.44]
Girgis, A, et al., 2009 II.	120	80.80	25.60	117	79.50	25.10	baseline	0.00	0.05	[-0.20; 0.31]
Yun et al., 2017	134	75.80	26.80	72	73.10	23.40	baseline	0.00	0.10	[-0.18; 0.39]
Thomas, ML, et al., 2012	64	21.10	5.40	88	19.00	6.30	baseline	0.00	0.35	[0.03; 0.68]
Heiney, SP, et al., 2003	33	5.80	2.00	33	6.55	1.80	followup	16.00	-0.39	[-0.88; 0.10]
Heiney, SP, et al., 2003	33	5.80	2.00	33	5.90	1.90	followup	6.00	-0.05	[-0.53; 0.43]
Girgis, A, et al., 2009 II.	120	89.20	20.30	117	89.90	17.00	followup	12.00	-0.04	[-0.29; 0.22]
Kim, SH, et al., 2021	47	80.30	17.60	47	80.40	18.50	followup	20.00	-0.01	[-0.41; 0.40]
Girgis, A, et al., 2009 II.	120	91.90	17.60	117	91.90	17.40	followup	24.00	0.00	[-0.25; 0.25]
Thomas, ML, et al., 2012	64	20.50	6.10	88	19.00	6.40	followup	12.00	0.24	[-0.09; 0.56]
Yun et al., 2017	134	85.30	19.50	72	78.20	22.40	followup	48.00	0.34	[0.06; 0.63]
Kim, SH, et al., 2021	47	81.90	15.20	47	74.60	23.00	followup	8.00	0.37	[-0.04; 0.78]
Dirksen, S. et al, 2007	34	23.30	3.90	38	21.40	5.90	followup	10.00	0.37	[-0.10; 0.84]
Random effect	1064			987					0.27	[-0.11; 0.66]
Prediction interval										[-1.42; 1.96]
**online**										
Peng, L, et al., 2022	28	76.79	15.27	29	81.61	16.87	baseline	0.00	-0.30	[-0.82; 0.23]
Wu, Q, et al., 2021	43	53.77	5.51	43	54.34	5.53	baseline	0.00	-0.10	[-0.53; 0.32]
Cengiz, HO, et al., 2023	32	20.16	5.97	33	19.13	5.49	baseline	0.00	0.18	[-0.31; 0.66]
Penedo, FJ, et al., 2020	95	-19.85	4.09	97	-21.16	4.24	baseline	0.00	0.31	[0.03; 0.60]
Beatty, L. et al. 2015	30	57.26	5.49	30	66.56	5.41	followup	6.00	-1.68	[-2.28; -1.09]
Peng, L, et al., 2022	28	80.36	20.09	29	82.18	19.38	followup	0.10	-0.09	[-0.61; 0.43]
Peng, L, et al., 2022	28	79.17	16.49	29	79.89	20.11	followup	4.00	-0.04	[-0.56; 0.48]
Cengiz, HO, et al., 2023	32	20.36	5.38	33	19.06	5.74	followup	8.00	0.23	[-0.26; 0.72]
Penedo, FJ, et al., 2020	95	-20.74	4.29	97	-22.05	4.43	followup	48.00	0.30	[0.01; 0.58]
Penedo, FJ, et al., 2020	95	-20.61	4.19	97	-21.91	4.33	followup	24.00	0.30	[0.02; 0.59]
Beatty, L. et al. 2015	30	77.87	4.62	30	75.08	4.61	followup	13.03	0.60	[0.08; 1.11]
Beatty, L. et al. 2015	30	87.98	4.26	30	83.84	4.75	followup	26.07	0.91	[0.37; 1.44]
Wu, Q, et al., 2021	43	81.65	8.30	43	72.36	7.51	followup	12.00	1.16	[0.70; 1.62]
Random effect	609			620					0.35	[-0.10; 0.81]
Prediction interval										[-1.55; 2.26]
**Random effect**	**8308**			**8806**					**0.39**	**[0.01; 0.78]**
**Prediction interval**										**[-1.04; 1.82]**

Figure S14.3. Forest plot represents the difference between the intervention vs. control group in the Social QoL domain with the environment subgroups as predicted at week 24 (post-intervention). SMD - Standardized mean difference, CI - confidence interval.

Figure S14.4.T48

Study	Experimental Patient N	Experimental Mean	Experimental SD	Control Patient N	Control Mean	Control SD	follow-up	Follow-up time	SMD	95%-CI
face to face										
Rodríguez Vega, B, et al., 2010	39	3.31	3.32	33	6.08	3.62	baseline	0.00	-0.79	[-1.27; -0.31]
Ferguson, RJ, et al., 2012	19	6.26	2.05	21	7.31	1.21	baseline	0.00	-0.62	[-1.26; 0.02]
Serfaty M, et al., 2018	20	16.40	6.10	22	18.10	7.80	baseline	0.00	-0.24	[-0.84; 0.37]
Qiu, H, et al., 2018 II.	98	17.84	5.10	196	18.85	4.22	baseline	0.00	-0.22	[-0.47; 0.02]
Chen, et al., 2017	58	58.30	6.20	65	59.60	6.90	baseline	0.00	-0.20	[-0.55; 0.16]
Berglund, G. et al. 2007	39	80.30	22.60	150	84.00	22.10	baseline	0.00	-0.17	[-0.52; 0.19]
Qiu, H, et al., 2018 I.	98	18.13	5.70	196	18.85	4.22	baseline	0.00	-0.15	[-0.39; 0.09]
Ruiz-Vozmediano, J, et al.,2020	31	69.40	29.80	32	73.40	28.40	baseline	0.00	-0.14	[-0.63; 0.36]
Fang, P, et al.,2020	60	48.50	4.11	60	49.08	5.01	baseline	0.00	-0.13	[-0.48; 0.23]
Seliniotaki, T, et al., 2021	27	74.40	29.90	26	77.70	29.30	baseline	0.00	-0.11	[-0.65; 0.43]
Antoni, MH, et al., 2006	92	877.61	158.55	107	891.17	152.16	baseline	0.00	-0.09	[-0.37; 0.19]
Hernandez, EG, et al. 2018	28	17.11	6.05	28	17.56	5.46	baseline	0.00	-0.08	[-0.60; 0.45]
McLachlan, SA, et al., 2001	296	41.20	22.60	154	42.80	18.70	baseline	0.00	-0.07	[-0.27; 0.12]
Liu, T, et al., 2019	49	63.77	24.95	53	65.22	23.71	baseline	0.00	-0.06	[-0.45; 0.33]
Li, X, et al., 2017	102	13.58	2.23	108	13.67	2.21	baseline	0.00	-0.04	[-0.31; 0.23]
Chan, et al. 2005	80	54.76	60.02	75	56.28	54.76	baseline	0.00	-0.03	[-0.34; 0.29]
Braeken APB, et al. 2013	136	84.94	21.44	144	85.46	22.52	baseline	0.00	-0.02	[-0.26; 0.21]
van der Meulen, IC, et al., 2013	88	83.10	2.50	91	83.10	2.50	baseline	0.00	0.00	[-0.29; 0.29]
Schofield, P, et al., 2013	55	70.90	26.77	53	70.75	26.72	baseline	0.00	0.01	[-0.37; 0.38]
Klafke, N, et al., 2019	120	59.30	31.30	113	58.60	29.60	baseline	0.00	0.02	[-0.23; 0.28]
Qin, X, et al., 2017	50	77.68	15.31	50	77.26	16.04	baseline	0.00	0.03	[-0.37; 0.42]
Baoyindeligeer, L.Z. et al. 2020	65	66.59	14.95	65	65.59	15.24	baseline	0.00	0.07	[-0.28; 0.41]
Elyasi, F, et al., 2021 I.	15	2.00	1.20	15	1.90	0.90	baseline	0.00	0.09	[-0.62; 0.81]
Klinkhammer-Schalke, M et al., 2012	100	71.21	33.50	100	67.99	34.25	baseline	0.00	0.09	[-0.18; 0.37]
Zhao,X, et al. 2015	62	7.01	2.18	62	6.76	2.39	baseline	0.00	0.11	[-0.24; 0.46]
Guo, Z, et al., 2013	89	75.00	13.82	89	73.43	12.31	baseline	0.00	0.12	[-0.17; 0.41]
Arving, C, et al., 2007	47	78.00	27.00	38	75.00	19.00	baseline	0.00	0.13	[-0.30; 0.55]
Rahmani, S, et al. 2015	12	34.72	13.22	12	33.33	7.11	baseline	0.00	0.13	[-0.67; 0.93]
Rodrigez, B, et al., 2014	8	95.83	11.79	7	90.48	25.20	baseline	0.00	0.26	[-0.76; 1.28]
Powell, CB, et al., 2008	21	22.20	5.40	43	19.70	6.00	baseline	0.00	0.42	[-0.10; 0.95]
Trask, PC, et al. 2003	25	71.50	22.00	23	58.20	29.25	baseline	0.00	0.51	[-0.07; 1.08]
Elyasi, F, et al., 2021 II.	20	2.50	1.10	20	1.90	0.90	baseline	0.00	0.59	[-0.05; 1.22]
Lu, Z, et al., 201	203	75.30	4.00	103	71.30	6.50	baseline	0.00	0.80	[0.56; 1.05]
Zhao, X, et al., 2021	52	58.02	3.41	51	54.01	4.12	baseline	0.00	1.05	[0.64; 1.47]
Antoni, MH, et al., 2006	92	788.15	133.08	107	855.60	126.82	followup	48.00	-0.52	[-0.80; -0.23]
Ferguson, RJ, et al., 2012	19	6.75	1.78	21	7.47	1.59	followup	8.00	-0.42	[-1.05; 0.21]
Antoni, MH, et al., 2006	92	832.88	119.31	107	873.38	114.05	followup	24.00	-0.35	[-0.63; -0.07]
Berglund, G. et al. 2007	39	77.00	23.70	150	83.50	23.70	followup	48.00	-0.27	[-0.63; 0.08]
McLachlan, SA, et al., 2001	296	42.00	23.70	154	47.50	17.90	followup	24.00	-0.25	[-0.45; -0.06]
Schofield, P, et al., 2013	55	65.03	28.36	53	71.29	28.69	followup	12.00	-0.22	[-0.60; 0.16]
Chan, et al. 2005	80	76.19	54.76	75	87.23	54.76	followup	72.00	-0.20	[-0.52; 0.12]
Chan, et al. 2005	80	65.58	54.76	75	75.11	54.76	followup	24.00	-0.17	[-0.49; 0.14]
Chan, et al. 2005	80	76.62	54.76	75	85.06	54.76	followup	60.00	-0.15	[-0.47; 0.16]
Chan, et al. 2005	80	61.47	54.76	75	69.26	54.76	followup	12.00	-0.14	[-0.46; 0.17]
Serfaty M, et al., 2018	20	18.70	5.30	22	19.40	6.20	followup	12.00	-0.12	[-0.72; 0.49]
Schofield, P, et al., 2013	55	68.28	26.22	53	70.58	26.38	followup	8.00	-0.09	[-0.46; 0.29]
Klinkhammer-Schalke, M et al., 2012	100	78.90	27.54	100	81.14	24.07	followup	48.00	-0.09	[-0.36; 0.19]
Rodrigez, B, et al., 2014	8	93.33	14.91	7	94.44	9.62	followup	2.00	-0.08	[-1.10; 0.93]
Chan, et al. 2005	80	80.52	54.76	75	83.77	54.76	followup	48.00	-0.06	[-0.37; 0.26]
Braeken APB, et al. 2013	136	86.99	20.73	144	87.55	19.10	followup	48.00	-0.03	[-0.26; 0.21]
Klinkhammer-Schalke, M et al., 2012	100	66.00	33.00	100	66.74	31.76	followup	12.00	-0.02	[-0.30; 0.25]
Klafke, N, et al., 2019	120	54.50	29.60	113	55.10	29.50	followup	24.00	-0.02	[-0.28; 0.24]
Elyasi, F, et al., 2021 I.	15	1.80	0.90	15	1.80	0.90	followup	24.00	0.00	[-0.72; 0.72]
Qiu, H, et al., 2018 II.	98	17.98	8.92	196	17.98	8.52	followup	4.00	0.00	[-0.24; 0.24]
Rahmani, S, et al. 2015	12	12.00	54.16	12	12.00	34.72	followup	16.00	0.00	[-0.80; 0.80]
Seliniotaki, T, et al., 2021	27	74.60	35.90	26	74.60	29.30	followup	8.00	0.00	[-0.54; 0.54]
Qiu, H, et al., 2018 II.	98	18.97	8.92	196	18.75	9.46	followup	24.00	0.02	[-0.22; 0.27]
Klinkhammer-Schalke, M et al., 2012	100	72.20	28.78	100	71.46	29.03	followup	24.00	0.03	[-0.25; 0.30]
Qin, X, et al., 2017	50	80.24	13.46	50	79.67	13.13	followup	2.00	0.04	[-0.35; 0.43]
Arving, C, et al., 2007	47	76.00	25.00	38	75.00	19.00	followup	12.00	0.04	[-0.38; 0.47]
Chan, et al. 2005	80	80.95	54.76	75	77.92	54.76	followup	36.00	0.06	[-0.26; 0.37]
Braeken APB, et al. 2013	136	83.46	23.57	144	81.81	22.37	followup	12.00	0.07	[-0.16; 0.31]
Qiu, H, et al., 2018 II.	98	18.77	8.03	196	18.25	5.05	followup	12.00	0.08	[-0.16; 0.33]
Klafke, N, et al., 2019	120	62.10	29.40	113	59.70	27.60	followup	12.00	0.08	[-0.17; 0.34]
Arving, C, et al., 2007	47	81.00	22.00	38	79.00	25.00	followup	4.00	0.08	[-0.34; 0.51]
Zhao, X, et al., 2021	52	58.62	6.31	51	58.02	3.21	followup	12.00	0.12	[-0.27; 0.51]
Arving, C, et al., 2007	47	83.00	21.00	38	80.00	26.00	followup	24.00	0.13	[-0.30; 0.56]
Klinkhammer-Schalke, M et al., 2012	100	77.91	27.05	100	73.69	30.77	followup	36.00	0.15	[-0.13; 0.42]
Ruiz-Vozmediano, J, et al.,2020	31	83.30	24.70	32	78.70	28.50	followup	24.00	0.17	[-0.32; 0.67]
Klafke, N, et al., 2019	120	69.10	27.90	113	63.60	26.60	followup	48.00	0.20	[-0.06; 0.46]
Qiu, H, et al., 2018 I.	98	20.01	9.15	196	17.98	8.52	followup	4.00	0.23	[-0.01; 0.47]
Serfaty M, et al., 2018	20	21.20	4.30	22	19.70	6.70	followup	18.00	0.26	[-0.35; 0.87]
Rodrigez, B, et al., 2014	8	95.83	11.79	7	90.48	25.20	followup	0.10	0.26	[-0.76; 1.28]
Guo, Z, et al., 2013	89	74.35	10.67	89	71.33	11.88	followup	2.00	0.27	[-0.03; 0.56]
Trask, PC, et al. 2003	25	86.10	23.50	23	79.40	20.62	followup	24.00	0.30	[-0.27; 0.87]
Li, X, et al., 2017	102	15.45	2.67	108	14.52	3.28	followup	12.00	0.31	[0.04; 0.58]
Powell, CB, et al., 2008	21	21.30	5.60	43	19.10	7.30	followup	12.00	0.32	[-0.21; 0.84]
Qiu, H, et al., 2018 I.	98	21.75	8.70	196	18.75	9.46	followup	24.00	0.32	[0.08; 0.57]
Hernandez, EG, et al. 2018	28	18.82	5.79	28	16.40	5.07	followup	24.00	0.44	[-0.09; 0.97]
Serfaty M, et al., 2018	20	19.50	7.20	22	16.00	7.60	followup	24.00	0.46	[-0.15; 1.08]
Hernandez, EG, et al. 2018	28	19.23	5.51	28	16.60	5.39	followup	8.00	0.48	[-0.06; 1.01]
Qiu, H, et al., 2018 I.	98	21.39	8.03	196	18.25	5.05	followup	12.00	0.50	[0.26; 0.75]
Liu, T, et al., 2019	49	65.81	18.49	53	56.11	17.08	followup	9.00	0.54	[0.15; 0.94]
Zhou, J, et al., 2020	59	72.34	11.43	59	65.87	10.26	followup	2.00	0.59	[0.22; 0.96]
Trask, PC, et al. 2003	25	85.90	24.00	23	70.10	27.34	followup	8.00	0.61	[0.03; 1.19]
Zhao,X, et al. 2015	62	8.19	2.10	62	6.87	2.21	followup	0.30	0.61	[0.25; 0.97]
Elyasi, F, et al., 2021 II.	20	2.40	1.00	20	1.80	0.90	followup	24.00	0.62	[-0.02; 1.25]
Chen, et al., 2017	58	47.60	7.50	65	42.60	7.20	followup	8.00	0.68	[0.31; 1.04]
Chen, et al., 2017	58	46.80	7.60	65	41.50	7.50	followup	24.00	0.70	[0.33; 1.06]
Liu, T, et al., 2019	49	74.35	17.68	53	62.05	14.34	followup	12.00	0.76	[0.36; 1.16]
Lu, Z, et al., 201	203	76.10	5.20	103	71.10	8.20	followup	9.00	0.78	[0.54; 1.03]
van der Meulen, IC, et al., 2013	88	88.50	3.20	91	85.10	3.00	followup	48.00	1.09	[0.78; 1.41]
Baoyindeligeer, L.Z. et al. 2020	65	88.33	12.38	65	71.36	13.84	followup	2.00	1.28	[0.91; 1.66]
Rahmani, S, et al. 2015	12	51.38	11.14	12	29.16	16.09	followup	8.00	1.55	[0.62; 2.48]
Fang, P, et al.,2020	60	65.47	6.98	60	49.45	5.65	followup	4.00	2.51	[2.03; 2.99]
Rodríguez Vega, B, et al., 2010	39	49.04	3.32	33	29.42	3.62	followup	12.00	5.61	[4.56; 6.66]
Rodríguez Vega, B, et al., 2010	39	61.86	3.32	33	34.47	3.62	followup	24.00	7.83	[6.44; 9.22]
Random effect	6635			7199					0.20	[-0.18; 0.58]
Prediction interval										[-1.56; 1.95]
telephone										
Heiney, SP, et al., 2003	33	5.60	2.00	33	5.90	1.90	baseline	0.00	-0.15	[-0.64; 0.33]
Kim, SH, et al., 2021	47	73.70	81.90	47	75.50	22.00	baseline	0.00	-0.03	[-0.43; 0.37]
Dirksen, S. et al, 2007	34	22.10	4.90	38	22.20	6.10	baseline	0.00	-0.02	[-0.48; 0.44]
Girgis, A, et al., 2009 II.	120	80.80	25.60	117	79.50	25.10	baseline	0.00	0.05	[-0.20; 0.31]
Yun et al., 2017	134	75.80	26.80	72	73.10	23.40	baseline	0.00	0.10	[-0.18; 0.39]
Thomas, ML, et al., 2012	64	21.10	5.40	88	19.00	6.30	baseline	0.00	0.35	[0.03; 0.68]
Heiney, SP, et al., 2003	33	5.80	2.00	33	6.55	1.80	followup	16.00	-0.39	[-0.88; 0.10]
Heiney, SP, et al., 2003	33	5.80	2.00	33	5.90	1.90	followup	6.00	-0.05	[-0.53; 0.43]
Girgis, A, et al., 2009 II.	120	89.20	20.30	117	89.90	17.00	followup	12.00	-0.04	[-0.29; 0.22]
Kim, SH, et al., 2021	47	80.30	17.60	47	80.40	18.50	followup	20.00	-0.01	[-0.41; 0.40]
Girgis, A, et al., 2009 II.	120	91.90	17.60	117	91.90	17.40	followup	24.00	0.00	[-0.25; 0.25]
Thomas, ML, et al., 2012	64	20.50	6.10	88	19.00	6.40	followup	12.00	0.24	[-0.09; 0.56]
Yun et al., 2017	134	85.30	19.50	72	78.20	22.40	followup	48.00	0.34	[0.06; 0.63]
Kim, SH, et al., 2021	47	81.90	15.20	47	74.60	23.00	followup	8.00	0.37	[-0.04; 0.78]
Dirksen, S. et al, 2007	34	23.30	3.90	38	21.40	5.90	followup	10.00	0.37	[-0.10; 0.84]
Random effect	1064			987					0.05	[-0.28; 0.37]
Prediction interval										[-1.56; 1.66]
online										
Peng, L, et al., 2022	28	76.79	15.27	29	81.61	16.87	baseline	0.00	-0.30	[-0.82; 0.23]
Wu, Q, et al., 2021	43	53.77	5.51	43	54.34	5.53	baseline	0.00	-0.10	[-0.53; 0.32]
Cengiz, HO, et al., 2023	32	20.16	5.97	33	19.13	5.49	baseline	0.00	0.18	[-0.31; 0.66]
Penedo, FJ, et al., 2020	95	-19.85	4.09	97	-21.16	4.24	baseline	0.00	0.31	[0.03; 0.60]
Beatty, L. et al. 2015	30	57.26	5.49	30	66.56	5.41	followup	6.00	-1.68	[-2.28; -1.09]
Peng, L, et al., 2022	28	80.36	20.09	29	82.18	19.38	followup	0.10	-0.09	[-0.61; 0.43]
Peng, L, et al., 2022	28	79.17	16.49	29	79.89	20.11	followup	4.00	-0.04	[-0.56; 0.48]
Cengiz, HO, et al., 2023	32	20.36	5.38	33	19.06	5.74	followup	8.00	0.23	[-0.26; 0.72]
Penedo, FJ, et al., 2020	95	-20.74	4.29	97	-22.05	4.43	followup	48.00	0.30	[0.01; 0.58]
Penedo, FJ, et al., 2020	95	-20.61	4.19	97	-21.91	4.33	followup	24.00	0.30	[0.02; 0.59]
Beatty, L. et al. 2015	30	77.87	4.62	30	75.08	4.61	followup	13.03	0.60	[0.08; 1.11]
Beatty, L. et al. 2015	30	87.98	4.26	30	83.84	4.75	followup	26.07	0.91	[0.37; 1.44]
Wu, Q, et al., 2021	43	81.65	8.30	43	72.36	7.51	followup	12.00	1.16	[0.70; 1.62]
Random effect	609			620					0.13	[-0.24; 0.50]
Prediction interval										[-1.63; 1.88]
**Random effect**	**8308**			**8806**					**0.17**	**[-0.16; 0.51]**
**Prediction interval**										**[-1.56; 1.90]**

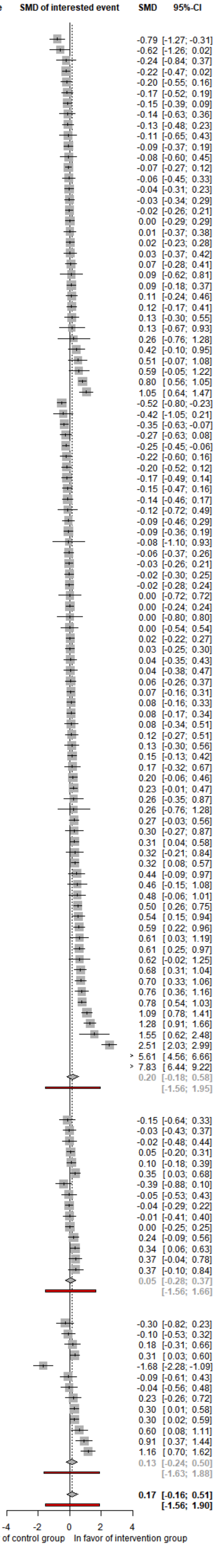

Figure S14.4. Forest plot represents the difference between the intervention vs. control group in the Social QoL domain with the environment subgroups as predicted at week 48 (post-intervention). SMD - Standardized mean difference, CI - confidence interval.

S15.Subgroup analysis of Social QoL: Type

Figure S15.1.T0

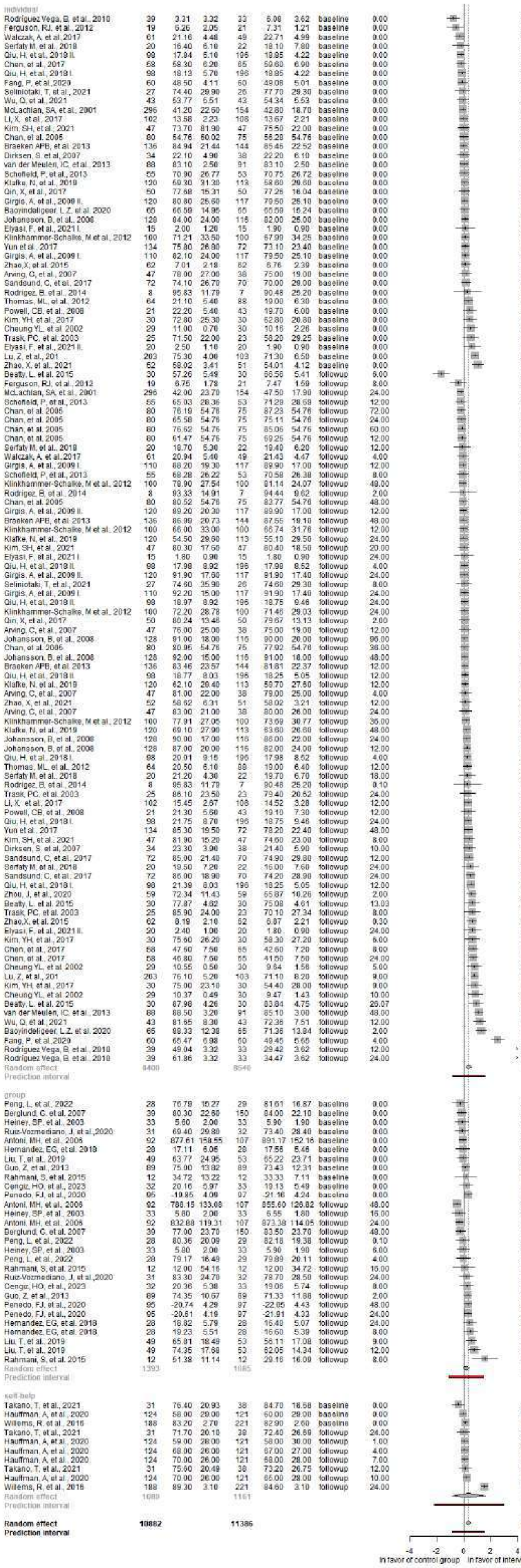

Study	Experimental Patient N	Experimental Mean	Experimental SD	Control Patient N	Control Mean	Control SD	follow-up	Follow-up time	SMD	95%-CI
individual										
Rodriguez Vega, B, et al., 2010	39	3.31	3.32	33	6.08	3.62	baseline	0.00	-0.79	[-1.27; -0.31]
Ferguson, RJ. et al., 2012	19	6.26	2.05	21	7.31	1.21	baseline	0.00	-0.62	[-1.26; 0.02]
Walczak, A, et al.,2017	61	21.16	4.48	49	22.71	4.99	baseline	0.00	-0.33	[-0.71; 0.05]
Serfaty M, et al., 2018	20	16.40	6.10	22	18.10	7.80	baseline	0.00	-0.24	[-0.84; 0.37]
Qiu, H, et al., 2018 II.	98	17.84	5.10	196	18.85	4.22	baseline	0.00	-0.22	[-0.47; 0.02]
Chen, et al., 2017	58	58.30	6.20	65	59.60	6.90	baseline	0.00	-0.20	[-0.55; 0.16]
Qiu, H, et al., 2018 I.	98	18.13	5.70	196	18.85	4.22	baseline	0.00	-0.15	[-0.39; 0.09]
Fang, P, et al.,2020	60	48.50	4.11	60	49.08	5.01	baseline	0.00	-0.13	[-0.48; 0.23]
Seliniotaki, T, et al., 2021	27	74.40	29.90	26	77.70	29.30	baseline	0.00	-0.11	[-0.65; 0.43]
Wu, Q, et al., 2021	43	53.77	5.51	43	54.34	5.53	baseline	0.00	-0.10	[-0.53; 0.32]
McLachlan, SA, et al., 2001	296	41.20	22.60	154	42.80	18.70	baseline	0.00	-0.07	[-0.27; 0.12]
Li, X, et al., 2017	102	13.58	2.23	108	13.67	2.21	baseline	0.00	-0.04	[-0.31; 0.23]
Kim, SH, et al., 2021	47	73.70	81.90	47	75.50	22.00	baseline	0.00	-0.03	[-0.43; 0.37]
Chan, et al. 2005	80	54.76	60.02	75	56.28	54.76	baseline	0.00	-0.03	[-0.34; 0.29]
Braeken APB, et al. 2013	136	84.94	21.44	144	85.46	22.52	baseline	0.00	-0.02	[-0.26; 0.21]
Dirksen, S. et al, 2007	34	22.10	4.90	38	22.20	6.10	baseline	0.00	-0.02	[-0.48; 0.44]
van der Meulen, IC, et al., 2013	88	83.10	2.50	91	83.10	2.50	baseline	0.00	0.00	[-0.29; 0.29]
Schofield, P, et al., 2013	55	70.90	26.77	53	70.75	26.72	baseline	0.00	0.01	[-0.37; 0.38]
Klafke, N, et al., 2019	120	59.30	31.30	113	58.60	29.60	baseline	0.00	0.02	[-0.23; 0.28]
Qin, X, et al., 2017	50	77.68	15.31	50	77.26	16.04	baseline	0.00	0.03	[-0.37; 0.42]
Girgis, A, et al., 2009 II.	120	80.80	25.60	117	79.50	25.10	baseline	0.00	0.05	[-0.20; 0.31]
Baoyindeligeer, L.Z. et al. 2020	65	66.59	14.95	65	65.59	15.24	baseline	0.00	0.07	[-0.28; 0.41]
Johansson, B, et al., 2008	128	84.00	24.00	116	82.00	25.00	baseline	0.00	0.08	[-0.17; 0.33]
Elyasi, F, et al., 2021 I.	15	2.00	1.20	15	1.90	0.90	baseline	0.00	0.09	[-0.62; 0.81]
Klinkhammer-Schalke, M.et al., 2012	100	71.21	33.50	100	67.99	34.25	baseline	0.00	0.09	[-0.18; 0.37]
Yun et al., 2017	134	75.80	26.80	72	73.10	23.40	baseline	0.00	0.10	[-0.18; 0.39]
Girgis, A, et al., 2009 I.	110	82.10	24.00	117	79.50	25.10	baseline	0.00	0.11	[-0.16; 0.37]
Zhao,X, et al. 2015	62	7.01	2.18	62	6.76	2.39	baseline	0.00	0.11	[-0.24; 0.46]
Arving, C, et al., 2007	47	78.00	27.00	38	75.00	19.00	baseline	0.00	0.13	[-0.30; 0.55]
Sandsund, C, et al., 2017	72	74.10	26.70	70	70.00	28.00	baseline	0.00	0.15	[-0.18; 0.48]
Rodrigez, B. et al., 2014	8	95.83	11.79	7	90.48	25.20	baseline	0.00	0.26	[-0.76; 1.28]
Thomas, ML, et al., 2012	64	21.10	5.40	88	19.00	6.30	baseline	0.00	0.35	[0.03; 0.68]
Powell, CB, et al., 2008	21	22.20	5.40	43	19.70	6.00	baseline	0.00	0.42	[-0.10; 0.95]
Kim, YH, et al., 2017	30	72.80	25.30	30	62.80	20.80	baseline	0.00	0.43	[-0.09; 0.94]
Cheung YL, et al. 2002	29	11.00	0.70	30	10.16	2.26	baseline	0.00	0.49	[-0.03; 1.01]
Trask, PC, et al. 2003	25	71.50	22.00	23	58.20	29.25	baseline	0.00	0.51	[-0.07; 1.08]
Elyasi, F, et al., 2021 II.	20	2.50	1.10	20	1.90	0.90	baseline	0.00	0.59	[-0.05; 1.22]
Lu, Z, et al., 201	203	75.30	4.00	103	71.30	6.50	baseline	0.00	0.80	[0.56; 1.05]
Zhao, X, et al., 2021	52	58.02	3.41	51	54.01	4.12	baseline	0.00	1.05	[0.64; 1.47]
Beatty, L. et al. 2015	30	57.26	5.49	30	66.56	5.41	followup	6.00	-1.68	[-2.28; -1.09]
Ferguson, RJ, et al., 2012	19	6.75	1.78	21	7.47	1.59	followup	8.00	-0.42	[-1.05; 0.21]
McLachlan, SA, et al., 2001	296	42.00	23.70	154	47.50	17.90	followup	24.00	-0.25	[-0.45; -0.06]
Schofield, P, et al., 2013	55	65.03	28.36	53	71.29	28.69	followup	12.00	-0.22	[-0.60; 0.16]
Chan, et al. 2005	80	76.19	54.78	75	87.23	54.76	followup	72.00	-0.20	[-0.52; 0.12]
Chan, et al. 2005	80	65.58	54.76	75	75.11	54.76	followup	24.00	-0.17	[-0.49; 0.14]
Chan, et al. 2005	80	76.62	54.76	75	85.06	54.76	followup	60.00	-0.15	[-0.47; 0.16]
Chan, et al. 2005	80	61.47	54.76	75	69.25	54.76	followup	12.00	-0.14	[-0.46; 0.17]
Serfaty M, et al., 2018	20	18.70	5.30	22	19.40	6.20	followup	12.00	-0.12	[-0.72; 0.49]
Walczak, A, et al.,2017	61	20.94	5.40	49	21.43	4.47	followup	4.00	-0.10	[-0.47; 0.28]
Girgis, A, et al., 2009 I.	110	88.20	19.30	117	89.90	17.00	followup	12.00	-0.09	[-0.35; 0.17]
Schofield, P, et al., 2013	55	68.28	26.22	53	70.58	26.38	followup	8.00	-0.09	[-0.46; 0.29]
Klinkhammer-Schalke, M et al., 2012	100	78.90	27.54	100	81.14	24.07	followup	48.00	-0.09	[-0.36; 0.19]
Rodrigez, B. et al., 2014	8	93.33	14.91	7	94.44	9.62	followup	2.00	-0.08	[-1.10; 0.93]
Chan, et al. 2005	80	80.52	54.76	75	83.77	54.76	followup	48.00	-0.06	[-0.37; 0.26]
Girgis, A, et al., 2009 II.	120	89.20	20.30	117	89.90	17.00	followup	12.00	-0.04	[-0.29; 0.22]
Braeken APB, et al. 2013	136	86.99	20.73	144	87.55	19.10	followup	48.00	-0.03	[-0.26; 0.21]
Klinkhammer-Schalke, M et al., 2012	100	66.00	33.00	100	66.74	31.76	followup	12.00	-0.02	[-0.30; 0.25]
Klafke, N, et al., 2019	120	54.50	29.60	113	55.10	29.50	followup	24.00	-0.02	[-0.28; 0.24]
Kim, SH, et al., 2021	47	80.30	17.60	47	80.40	18.50	followup	20.00	-0.01	[-0.41; 0.40]
Elyasi, F, et al., 2021 I.	15	1.80	0.90	15	1.80	0.90	followup	24.00	0.00	[-0.72; 0.72]
Qiu, H, et al., 2018 II.	98	17.98	8.92	196	17.98	8.52	followup	4.00	0.00	[-0.24; 0.24]
Girgis, A, et al., 2009 II.	120	91.90	17.60	117	91.90	17.40	followup	24.00	0.00	[-0.25; 0.25]
Seliniotaki, T, et al., 2021	27	74.60	35.90	26	74.60	29.30	followup	8.00	0.00	[-0.54; 0.54]
Girgis, A, et al., 2009 I.	110	92.20	15.00	117	91.90	17.40	followup	24.00	0.02	[-0.24; 0.28]
Qiu, H, et al., 2018 II.	98	18.97	8.92	196	18.75	9.46	followup	24.00	0.02	[-0.22; 0.27]
Klinkhammer-Schalke, M et al., 2012	100	72.20	28.78	100	71.46	29.03	followup	24.00	0.03	[-0.25; 0.30]
Qin, X, et al., 2017	50	80.24	13.48	50	79.67	13.13	followup	2.00	0.04	[-0.35; 0.43]
Arving, C, et al., 2007	47	76.00	25.00	38	75.00	19.00	followup	12.00	0.04	[-0.38; 0.47]
Johansson, B, et al., 2008	128	91.00	18.00	116	90.00	20.00	followup	96.00	0.05	[-0.20; 0.30]
Chan, et al. 2005	80	80.95	54.78	75	77.92	54.76	followup	36.00	0.06	[-0.26; 0.37]
Johansson, B, et al., 2008	128	92.00	15.00	116	91.00	18.00	followup	48.00	0.06	[-0.19; 0.31]
Braeken APB, et al. 2013	136	83.46	23.57	144	81.81	22.37	followup	12.00	0.07	[-0.16; 0.31]
Qiu, H, et al., 2018 II.	98	18.77	8.03	196	18.25	5.05	followup	12.00	0.08	[-0.16; 0.33]
Klafke, N, et al., 2019	120	62.10	29.40	113	59.70	27.60	followup	12.00	0.08	[-0.17; 0.34]
Arving, C, et al., 2007	47	81.00	22.00	38	79.00	25.00	followup	4.00	0.08	[-0.34; 0.51]
Zhao, X, et al., 2021	52	58.62	6.31	51	58.02	3.21	followup	12.00	0.12	[-0.27; 0.51]
Arving, C, et al., 2007	47	83.00	21.00	38	80.00	26.00	followup	24.00	0.13	[-0.30; 0.56]
Klinkhammer-Schalke, M et al., 2012	100	77.81	27.05	100	73.69	30.77	followup	36.00	0.15	[-0.13; 0.42]
Klafke, N, et al., 2019	120	69.10	27.90	113	63.60	26.60	followup	48.00	0.20	[-0.06; 0.46]
Johansson, B, et al., 2008	128	90.00	17.00	116	86.00	22.00	followup	24.00	0.20	[-0.05; 0.46]
Johansson, B, et al., 2008	128	87.00	20.00	116	82.00	24.00	followup	12.00	0.23	[-0.03; 0.48]
Qiu, H, et al., 2018 I.	98	20.01	9.15	196	17.98	8.52	followup	4.00	0.23	[-0.01; 0.47]
Thomas, ML, et al., 2012	64	20.50	6.10	88	19.00	6.40	followup	12.00	0.24	[-0.09; 0.56]
Serfaty M, et al., 2018	20	21.20	4.30	22	19.70	6.70	followup	18.00	0.25	[-0.35; 0.87]
Rodrigez, B. et al., 2014	8	95.83	11.79	7	90.48	25.20	followup	0.10	0.26	[-0.76; 1.28]
Trask, PC, et al. 2003	25	86.10	23.50	23	79.40	20.62	followup	24.00	0.30	[-0.27; 0.87]
Li, X, et al., 2017	102	15.45	2.67	108	14.52	3.28	followup	12.00	0.31	[0.04; 0.58]
Powell, CB, et al., 2008	21	21.30	5.60	43	19.10	7.30	followup	12.00	0.32	[-0.21; 0.84]
Qiu, H, et al., 2018 I.	98	21.75	8.70	196	18.75	9.46	followup	24.00	0.32	[0.08; 0.57]
Yun et al., 2017	134	85.30	19.50	72	78.20	22.40	followup	48.00	0.34	[0.06; 0.63]
Kim, SH, et al., 2021	47	81.90	15.20	47	74.60	23.00	followup	8.00	0.37	[-0.04; 0.78]
Dirksen, S. et al, 2007	34	23.30	3.90	38	21.40	5.90	followup	10.00	0.37	[-0.10; 0.84]
Sandsund, C, et al., 2017	72	85.00	21.40	70	74.90	29.80	followup	12.00	0.39	[0.06; 0.72]
Serfaty M, et al., 2018	20	19.50	7.20	22	16.00	7.60	followup	24.00	0.46	[-0.15; 1.08]
Sandsund, C, et al., 2017	72	86.00	18.90	70	74.20	28.90	followup	24.00	0.48	[0.15; 0.82]
Qiu, H, et al., 2018 I.	98	21.39	8.03	196	18.25	5.05	followup	12.00	0.50	[0.26; 0.75]
Zhou, J, et al., 2020	59	72.34	11.43	59	65.87	10.26	followup	2.00	0.59	[0.22; 0.96]
Beatty, L. et al. 2015	30	77.87	4.62	30	75.08	4.61	followup	13.03	0.60	[0.08; 1.11]
Trask, PC, et al. 2003	25	85.90	24.00	23	70.10	27.34	followup	8.00	0.61	[0.03; 1.19]
Zhao,X, et al. 2015	62	8.19	2.10	62	6.87	2.21	followup	0.30	0.61	[0.25; 0.97]
Elyasi, F, et al., 2021 II.	20	2.40	1.00	20	1.80	0.90	followup	24.00	0.62	[-0.02; 1.25]
Kim, YH, et al., 2017	30	75.60	26.20	30	58.30	27.20	followup	6.00	0.64	[0.12; 1.16]
Chen, et al., 2017	58	47.60	7.50	65	42.60	7.20	followup	8.00	0.68	[0.31; 1.04]
Chen, et al., 2017	58	46.80	7.60	65	41.50	7.50	followup	24.00	0.70	[0.33; 1.06]
Cheung YL, et al. 2002	29	10.55	0.50	30	9.64	1.56	followup	5.00	0.77	[0.24; 1.30]
Lu, Z, et al., 201	203	76.10	5.20	103	71.10	8.20	followup	9.00	0.78	[0.54; 1.03]
Kim, YH, et al., 2017	30	75.00	23.10	30	54.40	28.00	followup	9.00	0.79	[0.27; 1.32]
Cheung YL, et al. 2002	29	10.37	0.49	30	9.47	1.43	followup	10.00	0.83	[0.29; 1.36]
Beatty, L. et al. 2015	30	87.96	4.26	30	83.84	4.75	followup	26.07	0.91	[0.37; 1.44]
van der Meulen, IC, et al., 2013	88	88.50	3.20	91	85.10	3.00	followup	48.00	1.09	[0.78; 1.41]
Wu, Q, et al., 2021	43	81.65	8.30	43	72.38	7.51	followup	12.00	1.16	[0.70; 1.62]
Baoyindeligeer, L.Z. et al. 2020	65	88.33	12.38	65	71.36	13.84	followup	2.00	1.28	[0.91; 1.66]
Fang, P, et al.,2020	60	65.47	6.98	60	49.45	5.65	followup	4.00	2.51	[2.03; 2.99]
Rodriguez Vega, B, et al., 2010	39	49.04	3.32	33	29.42	3.62	followup	12.00	5.61	[4.56; 6.66]
Rodriguez Vega, B, et al., 2010	39	61.86	3.32	33	34.47	3.62	followup	24.00	7.83	[6.44; 9.22]
Random effect	8400			8540					0.35	[0.15; 0.55]
Prediction interval										[-0.86; 1.57]
group										
Peng, L, et al., 2022	28	76.79	15.27	29	81.61	16.87	baseline	0.00	-0.30	[-0.82; 0.23]
Berglund, G. et al. 2007	39	80.30	22.60	150	84.00	22.10	baseline	0.00	-0.17	[-0.52; 0.19]
Heiney, SP, et al., 2003	33	5.60	2.00	33	5.90	1.90	baseline	0.00	-0.15	[-0.64; 0.33]
Ruiz-Vozmediano, J, et al.,2020	31	69.40	29.80	32	73.40	28.40	baseline	0.00	-0.14	[-0.63; 0.36]
Antoni, MH, et al., 2006	92	877.61	158.55	107	891.17	152.16	baseline	0.00	-0.09	[-0.37; 0.19]
Hernandez, EG, et al. 2018	28	17.11	6.06	28	17.56	5.46	baseline	0.00	-0.08	[-0.60; 0.45]
Liu, T, et al., 2019	49	63.77	24.95	53	65.22	23.71	baseline	0.00	-0.06	[-0.45; 0.33]
Guo, Z, et al., 2013	89	75.00	13.82	89	73.43	12.31	baseline	0.00	0.12	[-0.17; 0.41]
Rahmani, S, et al. 2015	12	34.72	13.22	12	33.33	7.11	baseline	0.00	0.13	[-0.67; 0.93]
Cengiz, HO, et al., 2023	32	20.16	5.97	33	19.13	5.49	baseline	0.00	0.18	[-0.31; 0.66]
Penedo, FJ, et al., 2020	95	-19.85	4.09	97	-21.16	4.24	baseline	0.00	0.31	[0.03; 0.60]
Antoni, MH, et al., 2006	92	788.15	133.08	107	855.60	126.82	followup	48.00	-0.52	[-0.80; -0.23]
Heiney, SP, et al., 2003	33	5.80	2.00	33	6.55	1.80	followup	16.00	-0.39	[-0.88; 0.10]
Antoni, MH, et al., 2006	92	832.88	119.31	107	873.38	114.05	followup	24.00	-0.35	[-0.63; -0.07]
Berglund, G. et al. 2007	39	77.00	23.70	150	83.50	23.70	followup	48.00	-0.27	[-0.63; 0.08]
Peng, L, et al., 2022	28	80.36	20.09	29	82.18	19.38	followup	0.10	-0.09	[-0.61; 0.43]
Heiney, SP, et al., 2003	33	5.80	2.00	33	5.90	1.90	followup	6.00	-0.05	[-0.53; 0.43]
Peng, L, et al., 2022	28	79.17	16.49	29	79.89	20.11	followup	4.00	-0.04	[-0.56; 0.48]
Rahmani, S, et al. 2015	12	12.00	54.16	12	12.00	34.72	followup	16.00	0.00	[-0.80; 0.80]
Ruiz-Vozmediano, J, et al.,2020	31	83.30	24.70	32	78.70	28.50	followup	24.00	0.17	[-0.32; 0.67]
Cengiz, HO, et al., 2023	32	20.36	5.38	33	19.06	5.74	followup	8.00	0.23	[-0.26; 0.72]
Guo, Z, et al., 2013	89	74.35	10.67	89	71.33	11.68	followup	2.00	0.27	[-0.03; 0.56]
Penedo, FJ, et al., 2020	95	-20.74	4.29	97	-22.05	4.43	followup	48.00	0.30	[0.01; 0.58]
Penedo, FJ, et al., 2020	95	-20.61	4.19	97	-21.91	4.33	followup	24.00	0.30	[0.02; 0.59]
Hernandez, EG, et al. 2018	28	18.82	5.79	28	16.40	5.07	followup	24.00	0.44	[-0.09; 0.97]
Hernandez, EG, et al. 2018	28	19.23	5.51	28	16.60	5.39	followup	8.00	0.48	[-0.06; 1.01]
Liu, T, et al., 2019	49	65.81	18.49	53	56.11	17.08	followup	9.00	0.54	[0.15; 0.94]
Liu, T, et al., 2019	49	74.35	17.68	53	62.05	14.34	followup	12.00	0.76	[0.36; 1.16]
Rahmani, S, et al. 2015	12	51.38	11.14	12	29.16	16.09	followup	8.00	1.55	[0.62; 2.48]
Random effect	1393			1685					0.17	[-0.10; 0.44]
Prediction interval										[-1.12; 1.46]
self-help										
Takano, T, et al., 2021	31	76.40	20.93	38	84.70	16.58	baseline	0.00	-0.42	[-0.90; 0.06]
Hauffman, A, et al., 2020	124	58.00	29.00	121	60.00	29.00	baseline	0.00	-0.07	[-0.32; 0.18]
Willems, R, et al., 2016	188	83.20	2.70	221	82.90	2.60	baseline	0.00	0.11	[-0.08; 0.31]
Takano, T, et al., 2021	31	71.70	20.10	38	72.40	26.69	followup	24.00	-0.03	[-0.50; 0.45]
Hauffman, A, et al., 2020	124	59.00	28.00	121	58.00	30.00	followup	1.00	0.03	[-0.22; 0.28]
Hauffman, A, et al., 2020	124	68.00	26.00	121	67.00	27.00	followup	4.00	0.04	[-0.21; 0.29]
Hauffman, A, et al., 2020	124	70.00	26.00	121	68.00	28.00	followup	7.00	0.07	[-0.18; 0.32]
Takano, T, et al., 2021	31	75.60	20.49	38	73.20	26.75	followup	12.00	0.10	[-0.38; 0.57]
Hauffman, A, et al., 2020	124	70.00	26.00	121	65.00	28.00	followup	10.00	0.18	[-0.07; 0.44]
Willems, R, et al., 2016	188	89.30	3.10	221	84.60	3.10	followup	24.00	1.51	[1.29; 1.73]
Random effect	1089			1161					0.35	[-0.90; 1.60]
Prediction interval										[-2.22; 2.92]
**Random effect**	**10882**			**11386**					**0.32**	**[0.13; 0.51]**
**Prediction interval**										**[-0.89; 1.53]**

Figure S15.1. Forest plot represents the difference between the intervention vs. control group in the Social QoL domain with the type subgroups as predicted at week 0 (post-intervention). SMD - Standardized mean difference, CI - confidence interval.

Figure S15.2.T12

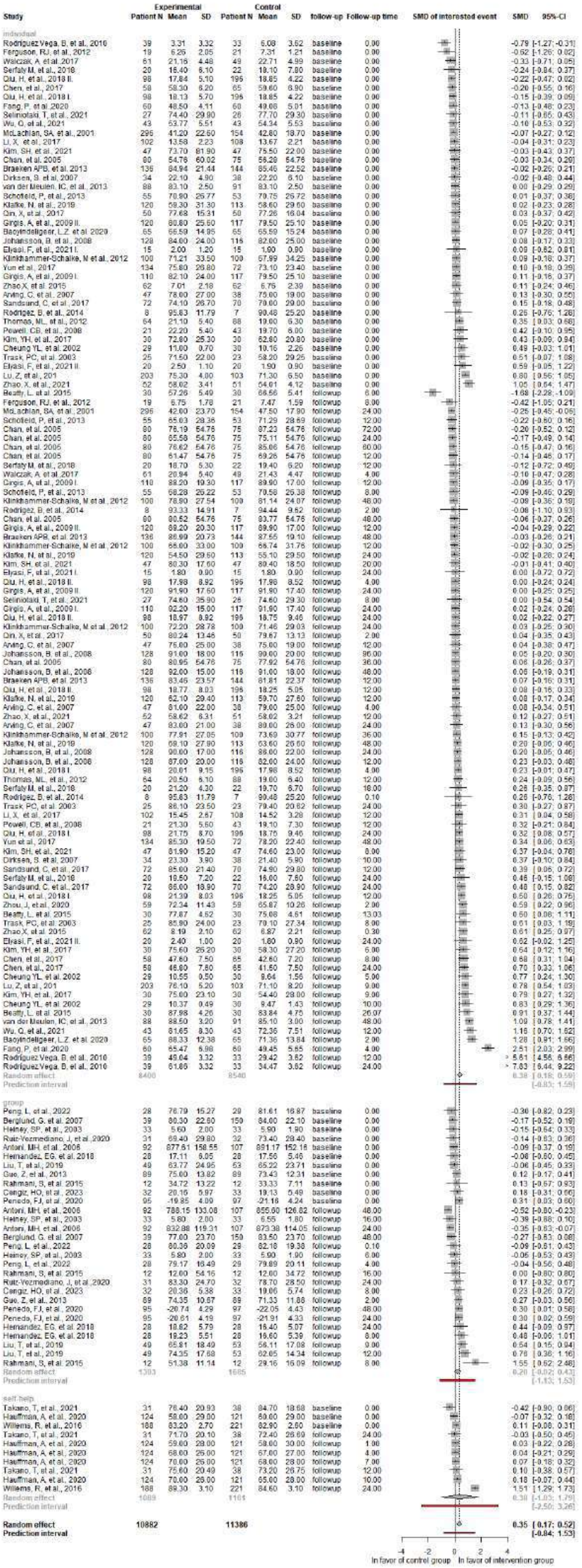

Figure S15.2. Forest plot represents the difference between the intervention vs. control group in the Social QoL domain with the type subgroups as predicted at week 12 (post-intervention). SMD - Standardized mean difference, CI - confidence interval.

Figure S15.3.T24

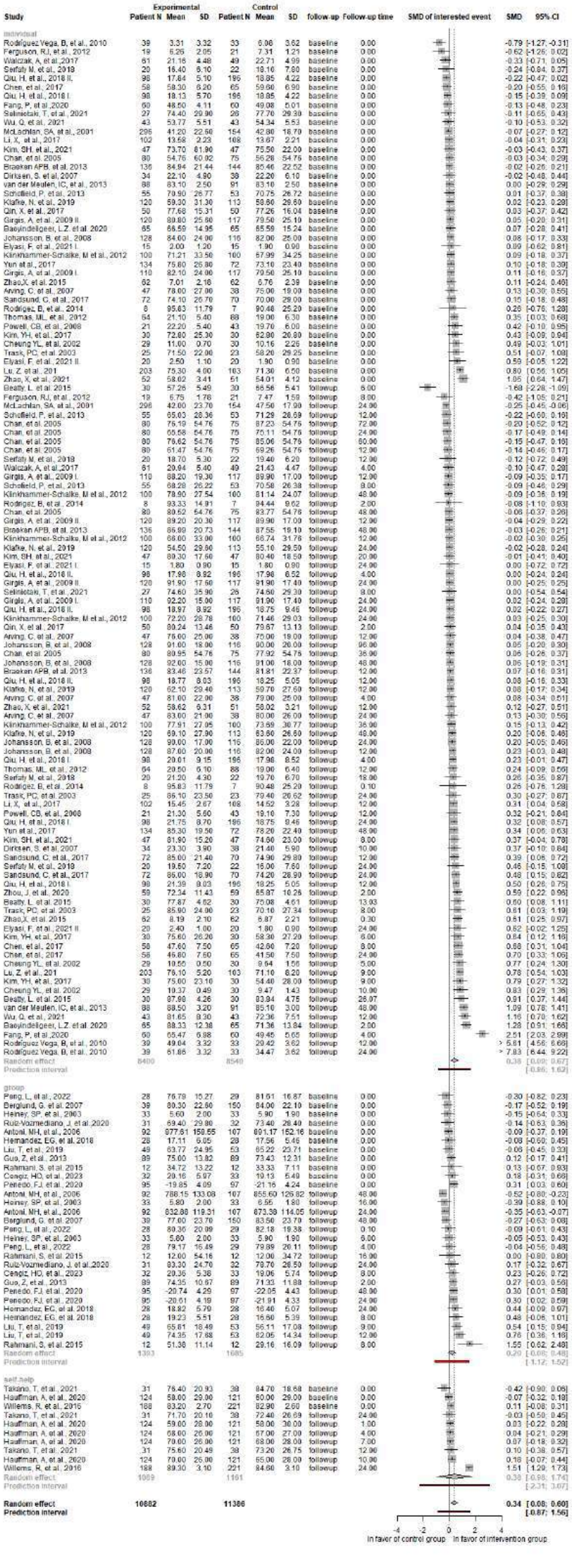

Figure S15.3. Forest plot represents the difference between the intervention vs. control group in the Social QoL domain with the type subgroups as predicted at week 24 (post-intervention). SMD - Standardized mean difference, CI - confidence interval.

Figure S15.4.T48

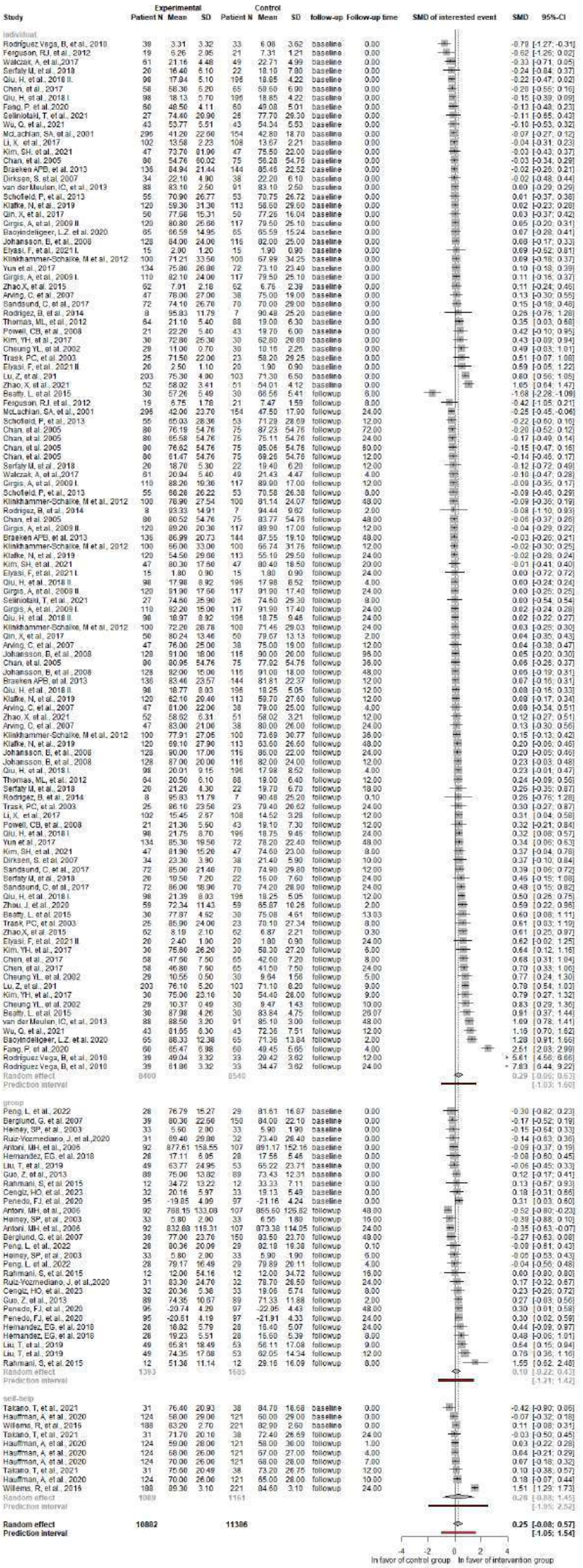

Figure S15.4. Forest plot represents the difference between the intervention vs. control group in the Social QoL domain with the type subgroups as predicted at week 48 (post-intervention). SMD - Standardized mean difference, CI - confidence interval.

S16.Subgroup analysis of Social QoL: Cancer stage

Figure S16.1.T0

Study	Experimental Patient N	Experimental Mean	Experimental SD	Control Patient N	Control Mean	Control SD	follow-up	Follow-up time	SMD	95%-CI
early										
Rodríguez Vega, B, et al., 2010	39	3.31	3.32	33	6.08	3.62	baseline	0.00	-0.79	[-1.27; -0.31]
Ferguson, RJ, et al., 2012	19	6.26	2.05	21	7.31	1.21	baseline	0.00	-0.62	[-1.26; 0.02]
Takano, T, et al., 2021	31	76.40	20.93	38	84.70	18.68	baseline	0.00	-0.42	[-0.90; 0.06]
Ruiz-Vozmediano, J, et al.,2020	31	69.40	29.80	32	73.40	28.40	baseline	0.00	-0.14	[-0.63; 0.36]
Fang, P, et al.,2020	60	48.50	4.11	60	49.08	5.01	baseline	0.00	-0.13	[-0.48; 0.23]
Wu, Q, et al., 2021	43	53.77	5.51	43	54.34	5.53	baseline	0.00	-0.10	[-0.53; 0.32]
Liu, T, et al., 2019	49	63.77	24.95	53	65.22	23.71	baseline	0.00	-0.06	[-0.45; 0.33]
Yun et al., 2017	134	75.80	26.80	72	73.10	23.40	baseline	0.00	0.10	[-0.18; 0.39]
Zhao,X, et al. 2015	62	7.01	2.18	62	6.76	2.39	baseline	0.00	0.11	[-0.24; 0.46]
Willems, R, et al., 2016	188	83.20	2.70	221	82.90	2.60	baseline	0.00	0.11	[-0.08; 0.31]
Cengiz, HO, et al., 2023	32	20.16	5.97	33	19.13	5.49	baseline	0.00	0.18	[-0.31; 0.66]
Rodrigez, B, et al., 2014	8	95.83	11.79	7	90.48	25.20	baseline	0.00	0.26	[-0.76; 1.28]
Zhao, X, et al., 2021	52	58.02	3.41	51	54.01	4.12	baseline	0.00	1.05	[0.64; 1.47]
Beatty, L. et al. 2015	30	57.26	5.49	30	66.56	5.41	followup	6.00	-1.68	[-2.28; -1.09]
Ferguson, RJ, et al., 2012	19	6.75	1.78	21	7.47	1.59	followup	8.00	-0.42	[-1.05; 0.21]
Rodrigez, B, et al., 2014	8	93.33	14.91	7	94.44	9.62	followup	2.00	-0.08	[-1.10; 0.93]
Takano, T, et al., 2021	31	71.70	20.10	38	72.40	26.69	followup	24.00	-0.03	[-0.50; 0.45]
Takano, T, et al., 2021	31	75.60	20.49	38	73.20	26.75	followup	12.00	0.10	[-0.38; 0.57]
Guan, S, et al., 2019	50	69.05	7.24	50	68.19	8.05	followup	8.00	0.11	[-0.28; 0.50]
Zhao, X, et al., 2021	52	58.62	6.31	51	58.02	3.21	followup	12.00	0.12	[-0.27; 0.51]
Ruiz-Vozmediano, J, et al.,2020	31	83.30	24.70	32	78.70	28.50	followup	24.00	0.17	[-0.32; 0.67]
Cengiz, HO, et al., 2023	32	20.36	5.38	33	19.06	5.74	followup	8.00	0.23	[-0.26; 0.72]
Rodrigez, B, et al., 2014	8	95.83	11.79	7	90.48	25.20	followup	0.10	0.26	[-0.76; 1.28]
Yun et al., 2017	134	85.30	19.50	72	78.20	22.40	followup	48.00	0.34	[0.06; 0.63]
Liu, T, et al., 2019	49	65.81	18.49	53	56.11	17.08	followup	9.00	0.54	[0.15; 0.94]
Beatty, L. et al. 2015	30	77.87	4.62	30	75.08	4.61	followup	13.03	0.60	[0.08; 1.11]
Zhao,X, et al. 2015	62	8.19	2.10	62	6.87	2.21	followup	0.30	0.61	[0.25; 0.97]
Liu, T, et al., 2019	49	74.35	17.68	53	62.05	14.34	followup	12.00	0.76	[0.36; 1.16]
Beatty, L. et al. 2015	30	87.98	4.26	30	83.84	4.75	followup	26.07	0.91	[0.37; 1.44]
Wu, Q, et al., 2021	43	81.65	8.30	43	72.36	7.51	followup	12.00	1.16	[0.70; 1.62]
Willems, R, et al., 2016	188	89.30	3.10	221	84.60	3.10	followup	24.00	1.51	[1.29; 1.73]
Fang, P, et al.,2020	60	65.47	6.98	60	49.45	5.65	followup	4.00	2.51	[2.03; 2.99]
Rodríguez Vega, B, et al., 2010	39	49.04	3.32	33	29.42	3.62	followup	12.00	5.61	[4.56; 6.66]
Rodríguez Vega, B, et al., 2010	39	61.86	3.32	33	34.47	3.62	followup	24.00	7.83	[6.44; 9.22]
Random effect	1763			1723					0.28	[-0.11; 0.67]
Prediction interval										[-2.01; 2.58]
advanced										
Walczak, A, et al.,2017	61	21.16	4.48	49	22.71	4.99	baseline	0.00	-0.33	[-0.71; 0.05]
Serfaty M, et al., 2018	20	16.40	6.10	22	18.10	7.80	baseline	0.00	-0.24	[-0.84; 0.37]
Heiney, SP, et al., 2003	33	5.60	2.00	33	5.90	1.90	baseline	0.00	-0.15	[-0.64; 0.33]
Schofield, P, et al., 2013	55	70.90	26.77	53	70.75	26.72	baseline	0.00	0.01	[-0.37; 0.38]
Penedo, FJ, et al., 2020	95	-19.85	4.09	97	-21.16	4.24	baseline	0.00	0.31	[0.03; 0.60]
Lu, Z, et al., 201	203	75.30	4.00	103	71.30	6.50	baseline	0.00	0.80	[0.56; 1.05]
Heiney, SP, et al., 2003	33	5.80	2.00	33	6.55	1.80	followup	16.00	-0.39	[-0.88; 0.10]
Schofield, P, et al., 2013	55	65.03	28.36	53	71.29	28.69	followup	12.00	-0.22	[-0.60; 0.16]
Serfaty M, et al., 2018	20	18.70	5.30	22	19.40	6.20	followup	12.00	-0.12	[-0.72; 0.49]
Walczak, A, et al.,2017	61	20.94	5.40	49	21.43	4.47	followup	4.00	-0.10	[-0.47; 0.28]
Schofield, P, et al., 2013	55	68.28	26.22	53	70.58	26.38	followup	8.00	-0.09	[-0.46; 0.29]
Heiney, SP, et al., 2003	33	5.80	2.00	33	5.90	1.90	followup	6.00	-0.05	[-0.53; 0.43]
Serfaty M, et al., 2018	20	21.20	4.30	22	19.70	6.70	followup	18.00	0.26	[-0.35; 0.87]
Penedo, FJ, et al., 2020	95	-20.74	4.29	97	-22.05	4.43	followup	48.00	0.30	[0.01; 0.58]
Penedo, FJ, et al., 2020	95	-20.61	4.19	97	-21.91	4.33	followup	24.00	0.30	[0.02; 0.59]
Serfaty M, et al., 2018	20	19.50	7.20	22	16.00	7.60	followup	24.00	0.46	[-0.15; 1.08]
Lu, Z, et al., 201	203	76.10	5.20	103	71.10	8.20	followup	9.00	0.78	[0.54; 1.03]
Random effect	1157			941					-0.11	[-0.74; 0.53]
Prediction interval										[-2.74; 2.53]
survivor										
Peng, L, et al., 2022	28	76.79	15.27	29	81.61	16.87	baseline	0.00	-0.30	[-0.82; 0.23]
Qiu, H, et al., 2018 II.	98	17.84	5.10	196	18.85	4.22	baseline	0.00	-0.22	[-0.47; 0.02]
Chen, et al., 2017	58	58.30	6.20	65	59.60	6.90	baseline	0.00	-0.20	[-0.55; 0.16]
Qiu, H, et al., 2018 I.	98	18.13	5.70	196	18.85	4.22	baseline	0.00	-0.15	[-0.39; 0.09]
Kim, SH, et al., 2021	47	73.70	81.90	47	75.50	22.00	baseline	0.00	-0.03	[-0.43; 0.37]
Peng, L, et al., 2022	28	80.36	20.09	29	82.18	19.38	followup	0.10	-0.09	[-0.61; 0.43]
Peng, L, et al., 2022	28	79.17	16.49	29	79.89	20.11	followup	4.00	-0.04	[-0.56; 0.48]
Kim, SH, et al., 2021	47	80.30	17.60	47	80.40	18.50	followup	20.00	-0.01	[-0.41; 0.40]
Qiu, H, et al., 2018 II.	98	17.98	8.92	196	17.98	8.52	followup	4.00	0.00	[-0.24; 0.24]
Qiu, H, et al., 2018 II.	98	18.97	8.92	196	18.75	9.46	followup	24.00	0.02	[-0.22; 0.27]
Qiu, H, et al., 2018 II.	98	18.77	8.03	196	18.25	5.05	followup	12.00	0.08	[-0.16; 0.33]
Qiu, H, et al., 2018 I.	98	20.01	9.15	196	17.98	8.52	followup	4.00	0.23	[-0.01; 0.47]
Qiu, H, et al., 2018 I.	98	21.75	8.70	196	18.75	9.46	followup	24.00	0.32	[0.08; 0.57]
Kim, SH, et al., 2021	47	81.90	15.20	47	74.60	23.00	followup	8.00	0.37	[-0.04; 0.78]
Qiu, H, et al., 2018 I.	98	21.39	8.03	196	18.25	5.05	followup	12.00	0.50	[0.26; 0.75]
Chen, et al., 2017	58	47.60	7.50	65	42.60	7.20	followup	8.00	0.68	[0.31; 1.04]
Chen, et al., 2017	58	46.80	7.60	65	41.50	7.50	followup	24.00	0.70	[0.33; 1.06]
Random effect	1183			1991					-0.21	[-0.88; 0.47]
Prediction interval										[-3.01; 2.60]
**Random effect**	**4103**			**4655**					**0.10**	**[-0.21; 0.40]**
**Prediction interval**										**[-2.15; 2.34]**

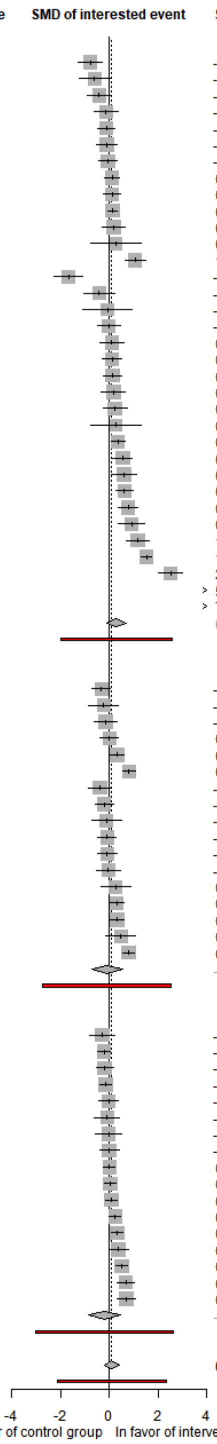

Figure S16.1. Forest plot represents the difference between the intervention vs. control group in the Social QoL domain with the cancer stage subgroups as predicted at week 0 (post-intervention). SMD - Standardized mean difference, CI - confidence interval.

Figure S16.2.T12

Study	Experimental Patient N	Experimental Mean	Experimental SD	Control Patient N	Control Mean	Control SD	follow-up	Follow-up time	SMD	95%-CI
early										
Rodríguez Vega, B, et al., 2010	39	3.31	3.32	33	6.08	3.62	baseline	0.00	-0.79	[-1.27; -0.31]
Ferguson, RJ, et al., 2012	19	6.26	2.05	21	7.31	1.21	baseline	0.00	-0.62	[-1.26; 0.02]
Takano, T, et al., 2021	31	76.40	20.93	38	84.70	18.68	baseline	0.00	-0.42	[-0.90; 0.06]
Ruiz-Vozmediano, J, et al.,2020	31	69.40	29.80	32	73.40	28.40	baseline	0.00	-0.14	[-0.63; 0.36]
Fang, P, et al.,2020	60	48.50	4.11	60	49.08	5.01	baseline	0.00	-0.13	[-0.48; 0.23]
Wu, Q, et al., 2021	43	53.77	5.51	43	54.34	5.53	baseline	0.00	-0.10	[-0.53; 0.32]
Liu, T, et al., 2019	49	63.77	24.95	53	65.22	23.71	baseline	0.00	-0.06	[-0.45; 0.33]
Yun et al., 2017	134	75.80	26.80	72	73.10	23.40	baseline	0.00	0.10	[-0.18; 0.39]
Zhao,X, et al. 2015	62	7.01	2.18	62	6.76	2.39	baseline	0.00	0.11	[-0.24; 0.46]
Willems, R, et al., 2016	188	83.20	2.70	221	82.90	2.60	baseline	0.00	0.11	[-0.08; 0.31]
Cengiz, HO, et al., 2023	32	20.16	5.97	33	19.13	5.49	baseline	0.00	0.18	[-0.31; 0.66]
Rodrigez, B, et al., 2014	8	95.83	11.79	7	90.48	25.20	baseline	0.00	0.26	[-0.76; 1.28]
Zhao, X, et al., 2021	52	58.02	3.41	51	54.01	4.12	baseline	0.00	1.05	[0.64; 1.47]
Beatty, L. et al. 2015	30	57.26	5.49	30	66.56	5.41	followup	6.00	-1.68	[-2.28; -1.09]
Ferguson, RJ, et al., 2012	19	6.75	1.78	21	7.47	1.59	followup	8.00	-0.42	[-1.05; 0.21]
Rodrigez, B, et al., 2014	8	93.33	14.91	7	94.44	9.62	followup	2.00	-0.08	[-1.10; 0.93]
Takano, T, et al., 2021	31	71.70	20.10	38	72.40	26.69	followup	24.00	-0.03	[-0.50; 0.45]
Takano, T, et al., 2021	31	75.60	20.49	38	73.20	26.75	followup	12.00	0.10	[-0.38; 0.57]
Guan, S, et al., 2019	50	69.05	7.24	50	68.19	8.05	followup	8.00	0.11	[-0.28; 0.50]
Zhao, X, et al., 2021	52	58.62	6.31	51	58.02	3.21	followup	12.00	0.12	[-0.27; 0.51]
Ruiz-Vozmediano, J, et al.,2020	31	83.30	24.70	32	78.70	28.50	followup	24.00	0.17	[-0.32; 0.67]
Cengiz, HO, et al., 2023	32	20.36	5.38	33	19.06	5.74	followup	8.00	0.23	[-0.26; 0.72]
Rodrigez, B, et al., 2014	8	95.83	11.79	7	90.48	25.20	followup	0.10	0.26	[-0.76; 1.28]
Yun et al., 2017	134	85.30	19.50	72	78.20	22.40	followup	48.00	0.34	[0.06; 0.63]
Liu, T, et al., 2019	49	65.81	18.49	53	56.11	17.08	followup	9.00	0.54	[0.15; 0.94]
Beatty, L. et al. 2015	30	77.87	4.62	30	75.08	4.61	followup	13.03	0.60	[0.08; 1.11]
Zhao,X, et al. 2015	62	8.19	2.10	62	6.87	2.21	followup	0.30	0.61	[0.25; 0.97]
Liu, T, et al., 2019	49	74.35	17.68	53	62.05	14.34	followup	12.00	0.76	[0.36; 1.16]
Beatty, L. et al. 2015	30	87.98	4.26	30	83.84	4.75	followup	26.07	0.91	[0.37; 1.44]
Wu, Q, et al., 2021	43	81.65	8.30	43	72.36	7.51	followup	12.00	1.16	[0.70; 1.62]
Willems, R, et al., 2016	188	89.30	3.10	221	84.60	3.10	followup	24.00	1.51	[1.29; 1.73]
Fang, P, et al.,2020	60	65.47	6.98	60	49.45	5.65	followup	4.00	2.51	[2.03; 2.99]
Rodríguez Vega, B, et al., 2010	39	49.04	3.32	33	29.42	3.62	followup	12.00	5.61	[4.56; 6.66]
Rodríguez Vega, B, et al., 2010	39	61.86	3.32	33	34.47	3.62	followup	24.00	7.83	[6.44; 9.22]
Random effect	1763			1723					0.84	[-0.06; 1.74]
Prediction interval										[-1.61; 3.30]
advanced										
Walczak, A, et al.,2017	61	21.16	4.48	49	22.71	4.99	baseline	0.00	-0.33	[-0.71; 0.05]
Serfaty M, et al., 2018	20	16.40	6.10	22	18.10	7.80	baseline	0.00	-0.24	[-0.84; 0.37]
Heiney, SP, et al., 2003	33	5.60	2.00	33	5.90	1.90	baseline	0.00	-0.15	[-0.64; 0.33]
Schofield, P, et al., 2013	55	70.90	26.77	53	70.75	26.72	baseline	0.00	0.01	[-0.37; 0.38]
Penedo, FJ, et al., 2020	95	-19.85	4.09	97	-21.16	4.24	baseline	0.00	0.31	[0.03; 0.60]
Lu, Z, et al., 201	203	75.30	4.00	103	71.30	6.50	baseline	0.00	0.80	[0.56; 1.05]
Heiney, SP, et al., 2003	33	5.80	2.00	33	6.55	1.80	followup	16.00	-0.39	[-0.88; 0.10]
Schofield, P, et al., 2013	55	65.03	28.36	53	71.29	28.69	followup	12.00	-0.22	[-0.60; 0.16]
Serfaty M, et al., 2018	20	18.70	5.30	22	19.40	6.20	followup	12.00	-0.12	[-0.72; 0.49]
Walczak, A, et al.,2017	61	20.94	5.40	49	21.43	4.47	followup	4.00	-0.10	[-0.47; 0.28]
Schofield, P, et al., 2013	55	68.28	26.22	53	70.58	26.38	followup	8.00	-0.09	[-0.46; 0.29]
Heiney, SP, et al., 2003	33	5.80	2.00	33	5.90	1.90	followup	6.00	-0.05	[-0.53; 0.43]
Serfaty M, et al., 2018	20	21.20	4.30	22	19.70	6.70	followup	18.00	0.26	[-0.35; 0.87]
Penedo, FJ, et al., 2020	95	-20.74	4.29	97	-22.05	4.43	followup	48.00	0.30	[0.01; 0.58]
Penedo, FJ, et al., 2020	95	-20.61	4.19	97	-21.91	4.33	followup	24.00	0.30	[0.02; 0.59]
Serfaty M, et al., 2018	20	19.50	7.20	22	16.00	7.60	followup	24.00	0.46	[-0.15; 1.08]
Lu, Z, et al., 201	203	76.10	5.20	103	71.10	8.20	followup	9.00	0.78	[0.54; 1.03]
Random effect	1157			941					0.45	[-0.11; 1.02]
Prediction interval										[-2.26; 3.16]
survivor										
Peng, L, et al., 2022	28	76.79	15.27	29	81.61	16.87	baseline	0.00	-0.30	[-0.82; 0.23]
Qiu, H, et al., 2018 II.	98	17.84	5.10	196	18.85	4.22	baseline	0.00	-0.22	[-0.47; 0.02]
Chen, et al., 2017	58	58.30	6.20	65	59.60	6.90	baseline	0.00	-0.20	[-0.55; 0.16]
Qiu, H, et al., 2018 I.	98	18.13	5.70	196	18.85	4.22	baseline	0.00	-0.15	[-0.39; 0.09]
Kim, SH, et al., 2021	47	73.70	81.90	47	75.50	22.00	baseline	0.00	-0.03	[-0.43; 0.37]
Peng, L, et al., 2022	28	80.36	20.09	29	82.18	19.38	followup	0.10	-0.09	[-0.61; 0.43]
Peng, L, et al., 2022	28	79.17	16.49	29	79.89	20.11	followup	4.00	-0.04	[-0.56; 0.48]
Kim, SH, et al., 2021	47	80.30	17.60	47	80.40	18.50	followup	20.00	-0.01	[-0.41; 0.40]
Qiu, H, et al., 2018 II.	98	17.98	8.92	196	17.98	8.52	followup	4.00	0.00	[-0.24; 0.24]
Qiu, H, et al., 2018 II.	98	18.97	8.92	196	18.75	9.46	followup	24.00	0.02	[-0.22; 0.27]
Qiu, H, et al., 2018 II.	98	18.77	8.03	196	18.25	5.05	followup	12.00	0.08	[-0.16; 0.33]
Qiu, H, et al., 2018 I.	98	20.01	9.15	196	17.98	8.52	followup	4.00	0.23	[-0.01; 0.47]
Qiu, H, et al., 2018 I.	98	21.75	8.70	196	18.75	9.46	followup	24.00	0.32	[0.08; 0.57]
Kim, SH, et al., 2021	47	81.90	15.20	47	74.60	23.00	followup	8.00	0.37	[-0.04; 0.78]
Qiu, H, et al., 2018 I.	98	21.39	8.03	196	18.25	5.05	followup	12.00	0.50	[0.26; 0.75]
Chen, et al., 2017	58	47.60	7.50	65	42.60	7.20	followup	8.00	0.68	[0.31; 1.04]
Chen, et al., 2017	58	46.80	7.60	65	41.50	7.50	followup	24.00	0.70	[0.33; 1.06]
Random effect	1183			1991					0.35	[-0.04; 0.75]
Prediction interval										[-2.60; 3.31]
**Random effect**	**4103**			**4655**					**0.64**	**[0.02; 1.25]**
**Prediction interval**										**[-1.66; 2.93]**

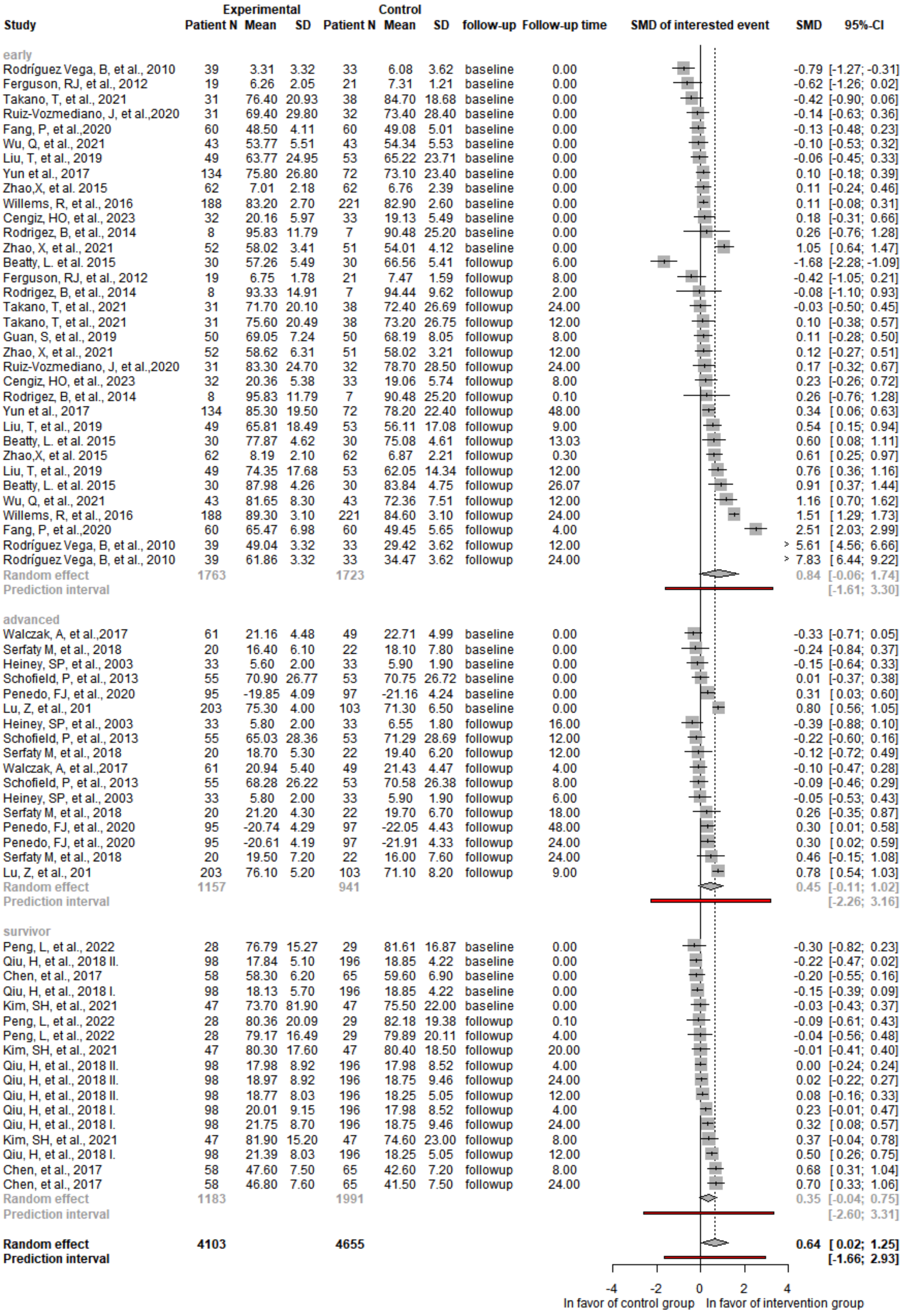

Figure S16.2. Forest plot represents the difference between the intervention vs. control group in the Social QoL domain with the cancer stage subgroups as predicted at week 12 (post-intervention). SMD - Standardized mean difference, CI - confidence interval.

Figure S16.3.T24

Study	Experimental Patient N	Experimental Mean	Experimental SD	Control Patient N	Control Mean	Control SD	follow-up	Follow-up time	SMD	95%-CI
early										
Rodríguez Vega, B, et al., 2010	39	3.31	3.32	33	6.08	3.62	baseline	0.00	-0.79	[-1.27; -0.31]
Ferguson, RJ, et al., 2012	19	6.26	2.05	21	7.31	1.21	baseline	0.00	-0.62	[-1.26; 0.02]
Takano, T, et al., 2021	31	76.40	20.93	38	84.70	18.68	baseline	0.00	-0.42	[-0.90; 0.06]
Ruiz-Vozmediano, J, et al.,2020	31	69.40	29.80	32	73.40	28.40	baseline	0.00	-0.14	[-0.63; 0.36]
Fang, P, et al.,2020	60	48.50	4.11	60	49.08	5.01	baseline	0.00	-0.13	[-0.48; 0.23]
Wu, Q, et al., 2021	43	53.77	5.51	43	54.34	5.53	baseline	0.00	-0.10	[-0.53; 0.32]
Liu, T, et al., 2019	49	63.77	24.95	53	65.22	23.71	baseline	0.00	-0.06	[-0.45; 0.33]
Yun et al., 2017	134	75.80	26.80	72	73.10	23.40	baseline	0.00	0.10	[-0.18; 0.39]
Zhao,X, et al. 2015	62	7.01	2.18	62	6.76	2.39	baseline	0.00	0.11	[-0.24; 0.46]
Willems, R, et al., 2016	188	83.20	2.70	221	82.90	2.60	baseline	0.00	0.11	[-0.08; 0.31]
Cengiz, HO, et al., 2023	32	20.16	5.97	33	19.13	5.49	baseline	0.00	0.18	[-0.31; 0.66]
Rodrigez, B, et al., 2014	8	95.83	11.79	7	90.48	25.20	baseline	0.00	0.26	[-0.76; 1.28]
Zhao, X, et al., 2021	52	58.02	3.41	51	54.01	4.12	baseline	0.00	1.05	[0.64; 1.47]
Beatty, L. et al. 2015	30	57.26	5.49	30	66.56	5.41	followup	6.00	-1.68	[-2.28; -1.09]
Ferguson, RJ, et al., 2012	19	6.75	1.78	21	7.47	1.59	followup	8.00	-0.42	[-1.05; 0.21]
Rodrigez, B, et al., 2014	8	93.33	14.91	7	94.44	9.62	followup	2.00	-0.08	[-1.10; 0.93]
Takano, T, et al., 2021	31	71.70	20.10	38	72.40	26.69	followup	24.00	-0.03	[-0.50; 0.45]
Takano, T, et al., 2021	31	75.60	20.49	38	73.20	26.75	followup	12.00	0.10	[-0.38; 0.57]
Guan, S, et al., 2019	50	69.05	7.24	50	68.19	8.05	followup	8.00	0.11	[-0.28; 0.50]
Zhao, X, et al., 2021	52	58.62	6.31	51	58.02	3.21	followup	12.00	0.12	[-0.27; 0.51]
Ruiz-Vozmediano, J, et al.,2020	31	83.30	24.70	32	78.70	28.50	followup	24.00	0.17	[-0.32; 0.67]
Cengiz, HO, et al., 2023	32	20.36	5.38	33	19.06	5.74	followup	8.00	0.23	[-0.26; 0.72]
Rodrigez, B, et al., 2014	8	95.83	11.79	7	90.48	25.20	followup	0.10	0.26	[-0.76; 1.28]
Yun et al., 2017	134	85.30	19.50	72	78.20	22.40	followup	48.00	0.34	[0.06; 0.63]
Liu, T, et al., 2019	49	65.81	18.49	53	56.11	17.08	followup	9.00	0.54	[0.15; 0.94]
Beatty, L. et al. 2015	30	77.87	4.62	30	75.08	4.61	followup	13.03	0.60	[0.08; 1.11]
Zhao,X, et al. 2015	62	8.19	2.10	62	6.87	2.21	followup	0.30	0.61	[0.25; 0.97]
Liu, T, et al., 2019	49	74.35	17.68	53	62.05	14.34	followup	12.00	0.76	[0.36; 1.16]
Beatty, L. et al. 2015	30	87.98	4.26	30	83.84	4.75	followup	26.07	0.91	[0.37; 1.44]
Wu, Q, et al., 2021	43	81.65	8.30	43	72.36	7.51	followup	12.00	1.16	[0.70; 1.62]
Willems, R, et al., 2016	188	89.30	3.10	221	84.60	3.10	followup	24.00	1.51	[1.29; 1.73]
Fang, P, et al.,2020	60	65.47	6.98	60	49.45	5.65	followup	4.00	2.51	[2.03; 2.99]
Rodríguez Vega, B, et al., 2010	39	49.04	3.32	33	29.42	3.62	followup	12.00	5.61	[4.56; 6.66]
Rodríguez Vega, B, et al., 2010	39	61.86	3.32	33	34.47	3.62	followup	24.00	7.83	[6.44; 9.22]
Random effect	1763			1723					1.09	[-0.09; 2.27]
Prediction interval										[-1.48; 3.65]
advanced										
Walczak, A, et al.,2017	61	21.16	4.48	49	22.71	4.99	baseline	0.00	-0.33	[-0.71; 0.05]
Serfaty M, et al., 2018	20	16.40	6.10	22	18.10	7.80	baseline	0.00	-0.24	[-0.84; 0.37]
Heiney, SP, et al., 2003	33	5.60	2.00	33	5.90	1.90	baseline	0.00	-0.15	[-0.64; 0.33]
Schofield, P, et al., 2013	55	70.90	26.77	53	70.75	26.72	baseline	0.00	0.01	[-0.37; 0.38]
Penedo, FJ, et al., 2020	95	-19.85	4.09	97	-21.16	4.24	baseline	0.00	0.31	[0.03; 0.60]
Lu, Z, et al., 201	203	75.30	4.00	103	71.30	6.50	baseline	0.00	0.80	[0.56; 1.05]
Heiney, SP, et al., 2003	33	5.80	2.00	33	6.55	1.80	followup	16.00	-0.39	[-0.88; 0.10]
Schofield, P, et al., 2013	55	65.03	28.36	53	71.29	28.69	followup	12.00	-0.22	[-0.60; 0.16]
Serfaty M, et al., 2018	20	18.70	5.30	22	19.40	6.20	followup	12.00	-0.12	[-0.72; 0.49]
Walczak, A, et al.,2017	61	20.94	5.40	49	21.43	4.47	followup	4.00	-0.10	[-0.47; 0.28]
Schofield, P, et al., 2013	55	68.28	26.22	53	70.58	26.38	followup	8.00	-0.09	[-0.46; 0.29]
Heiney, SP, et al., 2003	33	5.80	2.00	33	5.90	1.90	followup	6.00	-0.05	[-0.53; 0.43]
Serfaty M, et al., 2018	20	21.20	4.30	22	19.70	6.70	followup	18.00	0.26	[-0.35; 0.87]
Penedo, FJ, et al., 2020	95	-20.74	4.29	97	-22.05	4.43	followup	48.00	0.30	[0.01; 0.58]
Penedo, FJ, et al., 2020	95	-20.61	4.19	97	-21.91	4.33	followup	24.00	0.30	[0.02; 0.59]
Serfaty M, et al., 2018	20	19.50	7.20	22	16.00	7.60	followup	24.00	0.46	[-0.15; 1.08]
Lu, Z, et al., 201	203	76.10	5.20	103	71.10	8.20	followup	9.00	0.78	[0.54; 1.03]
Random effect	1157			941					0.70	[-0.12; 1.51]
Prediction interval										[-1.99; 3.39]
survivor										
Peng, L, et al., 2022	28	76.79	15.27	29	81.61	16.87	baseline	0.00	-0.30	[-0.82; 0.23]
Qiu, H, et al., 2018 II.	98	17.84	5.10	196	18.85	4.22	baseline	0.00	-0.22	[-0.47; 0.02]
Chen, et al., 2017	58	58.30	6.20	65	59.60	6.90	baseline	0.00	-0.20	[-0.55; 0.16]
Qiu, H, et al., 2018 I.	98	18.13	5.70	196	18.85	4.22	baseline	0.00	-0.15	[-0.39; 0.09]
Kim, SH, et al., 2021	47	73.70	81.90	47	75.50	22.00	baseline	0.00	-0.03	[-0.43; 0.37]
Peng, L, et al., 2022	28	80.36	20.09	29	82.18	19.38	followup	0.10	-0.09	[-0.61; 0.43]
Peng, L, et al., 2022	28	79.17	16.49	29	79.89	20.11	followup	4.00	-0.04	[-0.56; 0.48]
Kim, SH, et al., 2021	47	80.30	17.60	47	80.40	18.50	followup	20.00	-0.01	[-0.41; 0.40]
Qiu, H, et al., 2018 II.	98	17.98	8.92	196	17.98	8.52	followup	4.00	0.00	[-0.24; 0.24]
Qiu, H, et al., 2018 II.	98	18.97	8.92	196	18.75	9.46	followup	24.00	0.02	[-0.22; 0.27]
Qiu, H, et al., 2018 II.	98	18.77	8.03	196	18.25	5.05	followup	12.00	0.08	[-0.16; 0.33]
Qiu, H, et al., 2018 I.	98	20.01	9.15	196	17.98	8.52	followup	4.00	0.23	[-0.01; 0.47]
Qiu, H, et al., 2018 I.	98	21.75	8.70	196	18.75	9.46	followup	24.00	0.32	[0.08; 0.57]
Kim, SH, et al., 2021	47	81.90	15.20	47	74.60	23.00	followup	8.00	0.37	[-0.04; 0.78]
Qiu, H, et al., 2018 I.	98	21.39	8.03	196	18.25	5.05	followup	12.00	0.50	[0.26; 0.75]
Chen, et al., 2017	58	47.60	7.50	65	42.60	7.20	followup	8.00	0.68	[0.31; 1.04]
Chen, et al., 2017	58	46.80	7.60	65	41.50	7.50	followup	24.00	0.70	[0.33; 1.06]
Random effect	1183			1991					0.60	[-0.11; 1.31]
Prediction interval										[-2.25; 3.44]
**Random effect**	**4103**			**4655**					**0.87**	**[-0.04; 1.78]**
**Prediction interval**										**[-1.55; 3.29]**

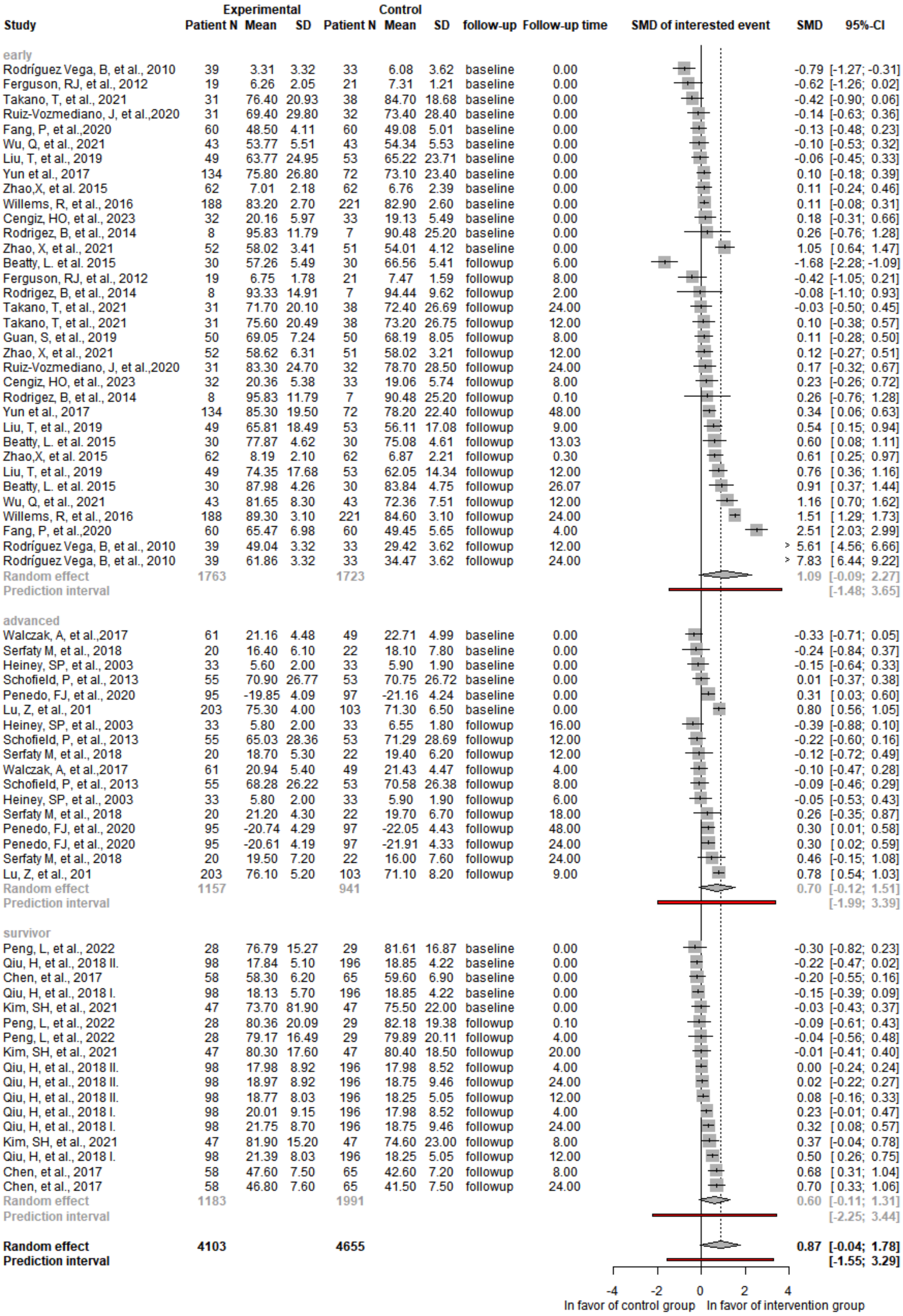

Figure S16.3. Forest plot represents the difference between the intervention vs. control group in the Social QoL domain with the cancer stage subgroups as predicted at week 24 (post-intervention). SMD - Standardized mean difference, CI - confidence interval.

Figure S16.4.T48

Study	Experimental Patient N	Experimental Mean	Experimental SD	Control Patient N	Control Mean	Control SD	follow-up	Follow-up time	SMD	95%-CI
**early**										
Rodríguez Vega, B, et al., 2010	39	3.31	3.32	33	6.08	3.62	baseline	0.00	-0.79	[-1.27; -0.31]
Ferguson, RJ, et al., 2012	19	6.26	2.05	21	7.31	1.21	baseline	0.00	-0.62	[-1.26; 0.02]
Takano, T, et al., 2021	31	76.40	20.93	38	84.70	18.68	baseline	0.00	-0.42	[-0.90; 0.06]
Ruiz-Vozmediano, J, et al.,2020	31	69.40	29.80	32	73.40	28.40	baseline	0.00	-0.14	[-0.63; 0.36]
Fang, P, et al.,2020	60	48.50	4.11	60	49.08	5.01	baseline	0.00	-0.13	[-0.48; 0.23]
Wu, Q, et al., 2021	43	53.77	5.51	43	54.34	5.53	baseline	0.00	-0.10	[-0.53; 0.32]
Liu, T, et al., 2019	49	63.77	24.95	53	65.22	23.71	baseline	0.00	-0.06	[-0.45; 0.33]
Yun et al., 2017	134	75.80	26.80	72	73.10	23.40	baseline	0.00	0.10	[-0.18; 0.39]
Zhao,X, et al. 2015	62	7.01	2.18	62	6.76	2.39	baseline	0.00	0.11	[-0.24; 0.46]
Willems, R, et al., 2016	188	83.20	2.70	221	82.90	2.60	baseline	0.00	0.11	[-0.08; 0.31]
Cengiz, HO, et al., 2023	32	20.16	5.97	33	19.13	5.49	baseline	0.00	0.18	[-0.31; 0.66]
Rodrigez, B, et al., 2014	8	95.83	11.79	7	90.48	25.20	baseline	0.00	0.26	[-0.76; 1.28]
Zhao, X, et al., 2021	52	58.02	3.41	51	54.01	4.12	baseline	0.00	1.05	[0.64; 1.47]
Beatty, L. et al. 2015	30	57.26	5.49	30	66.56	5.41	followup	6.00	-1.68	[-2.28; -1.09]
Ferguson, RJ, et al., 2012	19	6.75	1.78	21	7.47	1.59	followup	8.00	-0.42	[-1.05; 0.21]
Rodrigez, B, et al., 2014	8	93.33	14.91	7	94.44	9.62	followup	2.00	-0.08	[-1.10; 0.93]
Takano, T, et al., 2021	31	71.70	20.10	38	72.40	26.69	followup	24.00	-0.03	[-0.50; 0.45]
Takano, T, et al., 2021	31	75.60	20.49	38	73.20	26.75	followup	12.00	0.10	[-0.38; 0.57]
Guan, S, et al., 2019	50	69.05	7.24	50	68.19	8.05	followup	8.00	0.11	[-0.28; 0.50]
Zhao, X, et al., 2021	52	58.62	6.31	51	58.02	3.21	followup	12.00	0.12	[-0.27; 0.51]
Ruiz-Vozmediano, J, et al.,2020	31	83.30	24.70	32	78.70	28.50	followup	24.00	0.17	[-0.32; 0.67]
Cengiz, HO, et al., 2023	32	20.36	5.38	33	19.06	5.74	followup	8.00	0.23	[-0.26; 0.72]
Rodrigez, B, et al., 2014	8	95.83	11.79	7	90.48	25.20	followup	0.10	0.26	[-0.76; 1.28]
Yun et al., 2017	134	85.30	19.50	72	78.20	22.40	followup	48.00	0.34	[0.06; 0.63]
Liu, T, et al., 2019	49	65.81	18.49	53	56.11	17.08	followup	9.00	0.54	[0.15; 0.94]
Beatty, L. et al. 2015	30	77.87	4.62	30	75.08	4.61	followup	13.03	0.60	[0.08; 1.11]
Zhao,X, et al. 2015	62	8.19	2.10	62	6.87	2.21	followup	0.30	0.61	[0.25; 0.97]
Liu, T, et al., 2019	49	74.35	17.68	53	62.05	14.34	followup	12.00	0.76	[0.36; 1.16]
Beatty, L. et al. 2015	30	87.98	4.26	30	83.84	4.75	followup	26.07	0.91	[0.37; 1.44]
Wu, Q, et al., 2021	43	81.65	8.30	43	72.36	7.51	followup	12.00	1.16	[0.70; 1.62]
Willems, R, et al., 2016	188	89.30	3.10	221	84.60	3.10	followup	24.00	1.51	[1.29; 1.73]
Fang, P, et al.,2020	60	65.47	6.98	60	49.45	5.65	followup	4.00	2.51	[2.03; 2.99]
Rodríguez Vega, B, et al., 2010	39	49.04	3.32	33	29.42	3.62	followup	12.00	5.61	[4.56; 6.66]
Rodríguez Vega, B, et al., 2010	39	61.86	3.32	33	34.47	3.62	followup	24.00	7.83	[6.44; 9.22]
Random effect	1763			1723					0.62	[-1.08; 2.32]
Prediction interval										[-5.33; 6.57]
**advanced**										
Walczak, A, et al.,2017	61	21.16	4.48	49	22.71	4.99	baseline	0.00	-0.33	[-0.71; 0.05]
Serfaty M, et al., 2018	20	16.40	6.10	22	18.10	7.80	baseline	0.00	-0.24	[-0.84; 0.37]
Heiney, SP, et al., 2003	33	5.60	2.00	33	5.90	1.90	baseline	0.00	-0.15	[-0.64; 0.33]
Schofield, P, et al., 2013	55	70.90	26.77	53	70.75	26.72	baseline	0.00	0.01	[-0.37; 0.38]
Penedo, FJ, et al., 2020	95	-19.85	4.09	97	-21.16	4.24	baseline	0.00	0.31	[0.03; 0.60]
Lu, Z, et al., 201	203	75.30	4.00	103	71.30	6.50	baseline	0.00	0.80	[0.56; 1.05]
Heiney, SP, et al., 2003	33	6.55	1.80	33	6.55	1.80	followup	16.00	-0.39	[-0.88; 0.10]
Schofield, P, et al., 2013	55	65.03	28.36	53	71.29	28.69	followup	12.00	-0.22	[-0.60; 0.16]
Serfaty M, et al., 2018	20	18.70	5.30	22	19.40	6.20	followup	12.00	-0.12	[-0.72; 0.49]
Walczak, A, et al.,2017	61	20.94	5.40	49	21.43	4.47	followup	4.00	-0.10	[-0.47; 0.28]
Schofield, P, et al., 2013	55	68.28	26.22	53	70.58	26.38	followup	8.00	-0.09	[-0.46; 0.29]
Heiney, SP, et al., 2003	33	5.80	2.00	33	5.90	1.90	followup	6.00	-0.05	[-0.53; 0.43]
Serfaty M, et al., 2018	20	21.20	4.30	22	19.70	6.70	followup	18.00	0.26	[-0.35; 0.87]
Penedo, FJ, et al., 2020	95	-20.74	4.29	97	-22.05	4.43	followup	48.00	0.30	[0.01; 0.58]
Penedo, FJ, et al., 2020	95	-20.61	4.19	97	-21.91	4.33	followup	24.00	0.30	[0.02; 0.59]
Serfaty M, et al., 2018	20	19.50	7.20	22	16.00	7.60	followup	24.00	0.46	[-0.15; 1.08]
Lu, Z, et al., 201	203	76.10	5.20	103	71.10	8.20	followup	9.00	0.78	[0.54; 1.03]
Random effect	1157			941					0.23	[-1.05; 1.51]
Prediction interval										[-5.50; 5.96]
**survivor**										
Peng, L, et al., 2022	28	76.79	15.27	29	81.61	16.87	baseline	0.00	-0.30	[-0.82; 0.23]
Qiu, H, et al., 2018 II.	98	17.84	5.10	196	18.85	4.22	baseline	0.00	-0.22	[-0.47; 0.02]
Chen, et al., 2017	58	58.30	6.20	65	59.60	6.90	baseline	0.00	-0.20	[-0.55; 0.16]
Qiu, H, et al., 2018 I.	98	18.13	5.70	196	18.85	4.22	baseline	0.00	-0.15	[-0.39; 0.09]
Kim, SH, et al., 2021	47	73.70	81.90	47	75.50	22.00	baseline	0.00	-0.03	[-0.43; 0.37]
Peng, L, et al., 2022	28	80.36	20.09	29	82.18	19.38	followup	0.10	-0.09	[-0.61; 0.43]
Peng, L, et al., 2022	28	79.17	16.49	29	79.89	20.11	followup	4.00	-0.04	[-0.56; 0.48]
Kim, SH, et al., 2021	47	80.30	17.60	47	80.40	18.50	followup	20.00	-0.01	[-0.41; 0.40]
Qiu, H, et al., 2018 II.	98	17.98	8.92	196	17.98	8.52	followup	4.00	0.00	[-0.24; 0.24]
Qiu, H, et al., 2018 II.	98	18.97	8.92	196	18.75	9.46	followup	24.00	0.02	[-0.22; 0.27]
Qiu, H, et al., 2018 II.	98	18.77	8.03	196	18.25	5.05	followup	12.00	0.08	[-0.16; 0.33]
Qiu, H, et al., 2018 I.	98	20.01	9.15	196	17.98	8.52	followup	4.00	0.23	[-0.01; 0.47]
Qiu, H, et al., 2018 I.	98	21.75	8.70	196	18.75	9.46	followup	24.00	0.32	[0.08; 0.57]
Kim, SH, et al., 2021	47	81.90	15.20	47	74.60	23.00	followup	8.00	0.37	[-0.04; 0.78]
Qiu, H, et al., 2018 I.	98	21.39	8.03	196	18.25	5.05	followup	12.00	0.50	[0.26; 0.75]
Chen, et al., 2017	58	47.60	7.50	65	42.60	7.20	followup	8.00	0.68	[0.31; 1.04]
Chen, et al., 2017	58	46.80	7.60	65	41.50	7.50	followup	24.00	0.70	[0.33; 1.06]
Random effect	1183			1991					0.13	[-0.81; 1.07]
Prediction interval										[-3.74; 4.00]
**Random effect**	**4103**			**4655**					**0.43**	**[-0.65; 1.50]**
**Prediction interval**										**[-6.36; 7.21]**

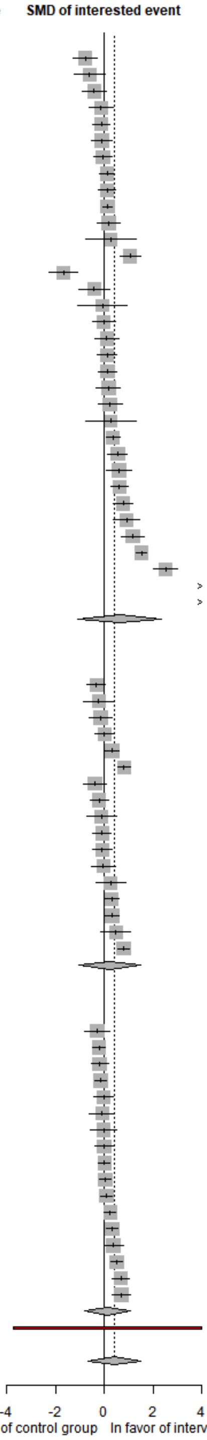

Figure S16.4. Forest plot represents the difference between the intervention vs. control group in the Social QoL domain with the cancer stage subgroups as predicted at week 48 (post-intervention). SMD - Standardized mean difference, CI - confidence interval.

S17.Subgroup analysis of Social QoL: Cancer type

Figure S17.1.T0

Study	Experimental Patient N	Experimental Mean	Experimental SD	Control Patient N	Control Mean	Control SD	follow-up	Follow-up time	SMD	95%-CI
**breast**										
Ferguson, RJ, et al., 2012	19	6.26	2.05	21	7.31	1.21	baseline	0.00	-0.62	[-1.26; 0.02]
Peng, L, et al., 2022	28	76.79	15.27	29	81.61	16.87	baseline	0.00	-0.30	[-0.82; 0.23]
Qiu, H, et al., 2018 II.	98	17.84	5.10	196	18.85	4.22	baseline	0.00	-0.22	[-0.47; 0.02]
Hoffman, CJ, et al., 2012	102	17.59	5.91	109	18.78	6.01	baseline	0.00	-0.20	[-0.47; 0.07]
Heiney, SP, et al., 2003	33	5.60	2.00	33	5.90	1.90	baseline	0.00	-0.15	[-0.64; 0.33]
Qiu, H, et al., 2018 I.	98	18.13	5.70	196	18.85	4.22	baseline	0.00	-0.15	[-0.39; 0.09]
Ruiz-Vozmediano, J, et al.,2020	31	69.40	29.80	32	73.40	28.40	baseline	0.00	-0.14	[-0.63; 0.36]
Seliniotaki, T, et al., 2021	27	74.40	29.90	26	77.70	29.30	baseline	0.00	-0.11	[-0.65; 0.43]
Wu, Q, et al., 2021	43	53.77	5.51	43	54.34	5.53	baseline	0.00	-0.10	[-0.53; 0.32]
Antoni, MH, et al., 2006	92	877.61	158.55	107	891.17	152.16	baseline	0.00	-0.09	[-0.37; 0.19]
Hernandez, EG, et al. 2018	28	17.11	6.05	28	17.56	5.46	baseline	0.00	-0.08	[-0.60; 0.45]
Kim, SH, et al., 2021	47	73.70	81.90	47	75.50	22.00	baseline	0.00	-0.03	[-0.43; 0.37]
Dirksen, S. et al, 2007	34	22.10	4.90	38	22.20	6.10	baseline	0.00	-0.02	[-0.48; 0.44]
Klafke, N, et al., 2019	120	59.30	31.30	113	58.60	29.60	baseline	0.00	0.02	[-0.23; 0.28]
Elyasi, F, et al., 2021 I.	15	2.00	1.20	15	1.90	0.90	baseline	0.00	0.09	[-0.62; 0.81]
Klinkhammer-Schalke, M et al., 2012	100	71.21	33.50	100	67.99	34.25	baseline	0.00	0.09	[-0.18; 0.37]
Arving, C, et al., 2007	47	78.00	27.00	38	75.00	19.00	baseline	0.00	0.13	[-0.30; 0.55]
Rahmani, S, et al. 2015	12	34.72	13.22	12	33.33	7.11	baseline	0.00	0.13	[-0.67; 0.93]
Cengiz, HO, et al., 2023	32	20.16	5.97	33	19.13	5.49	baseline	0.00	0.18	[-0.31; 0.66]
Kim, YH, et al., 2017	30	72.80	25.30	30	62.80	20.80	baseline	0.00	0.43	[-0.09; 0.94]
Elyasi, F, et al., 2021 II.	20	2.50	1.10	20	1.90	0.90	baseline	0.00	0.59	[-0.05; 1.22]
Antoni, MH, et al., 2006	92	788.15	133.08	107	855.60	126.82	followup	48.00	-0.52	[-0.80; -0.23]
Ferguson, RJ, et al., 2012	19	6.75	1.78	21	7.47	1.59	followup	8.00	-0.42	[-1.05; 0.21]
Heiney, SP, et al., 2003	33	5.80	2.00	33	6.55	1.80	followup	16.00	-0.39	[-0.88; 0.10]
Antoni, MH, et al., 2006	92	832.88	119.31	107	873.38	114.05	followup	24.00	-0.35	[-0.63; -0.07]
Peng, L, et al., 2022	28	80.36	20.09	29	82.18	19.38	followup	0.10	-0.09	[-0.61; 0.43]
Klinkhammer-Schalke, M et al., 2012	100	78.90	27.54	100	81.14	24.07	followup	48.00	-0.09	[-0.36; 0.19]
Heiney, SP, et al., 2003	33	5.80	2.00	33	5.90	1.90	followup	6.00	-0.05	[-0.53; 0.43]
Peng, L, et al., 2022	28	79.17	16.49	29	79.89	20.11	followup	4.00	-0.04	[-0.56; 0.48]
Hoffman, CJ, et al., 2012	102	18.09	5.81	109	18.30	5.75	followup	12.00	-0.04	[-0.31; 0.23]
Klinkhammer-Schalke, M et al., 2012	100	66.00	33.00	100	66.74	31.76	followup	12.00	-0.02	[-0.30; 0.25]
Klafke, N, et al., 2019	120	54.50	29.60	113	55.10	29.50	followup	24.00	-0.02	[-0.28; 0.24]
Kim, SH, et al., 2021	47	80.30	17.60	47	80.40	18.50	followup	20.00	-0.01	[-0.41; 0.40]
Elyasi, F, et al., 2021 I.	15	1.80	0.90	15	1.80	0.90	followup	24.00	0.00	[-0.72; 0.72]
Qiu, H, et al., 2018 II.	98	17.98	8.92	196	17.98	8.52	followup	4.00	0.00	[-0.24; 0.24]
Rahmani, S, et al. 2015	12	12.00	54.16	12	12.00	34.72	followup	16.00	0.00	[-0.80; 0.80]
Seliniotaki, T, et al., 2021	27	74.60	35.90	26	74.60	29.30	followup	8.00	0.00	[-0.54; 0.54]
Hoffman, CJ, et al., 2012	102	18.36	5.65	109	18.26	5.88	followup	8.00	0.02	[-0.25; 0.29]
Qiu, H, et al., 2018 II.	98	18.97	8.92	196	18.75	9.46	followup	24.00	0.02	[-0.22; 0.27]
Klinkhammer-Schalke, M et al., 2012	100	72.20	28.78	100	71.46	29.03	followup	24.00	0.03	[-0.25; 0.30]
Arving, C, et al., 2007	47	76.00	25.00	38	75.00	19.00	followup	12.00	0.04	[-0.38; 0.47]
Qiu, H, et al., 2018 II.	98	18.77	8.03	196	18.25	5.05	followup	12.00	0.08	[-0.16; 0.33]
Klafke, N, et al., 2019	120	62.10	29.40	113	59.70	27.60	followup	12.00	0.08	[-0.17; 0.34]
Arving, C, et al., 2007	47	81.00	22.00	38	79.00	25.00	followup	4.00	0.08	[-0.34; 0.51]
Arving, C, et al., 2007	47	83.00	21.00	38	80.00	26.00	followup	24.00	0.13	[-0.30; 0.56]
Klinkhammer-Schalke, M et al., 2012	100	77.91	27.05	100	73.69	30.77	followup	36.00	0.15	[-0.13; 0.42]
Ruiz-Vozmediano, J, et al.,2020	31	83.30	24.70	32	78.70	28.50	followup	24.00	0.17	[-0.32; 0.67]
Klafke, N, et al., 2019	120	69.10	27.90	113	63.60	26.60	followup	48.00	0.20	[-0.06; 0.46]
Cengiz, HO, et al., 2023	32	20.36	5.38	33	19.06	5.74	followup	8.00	0.23	[-0.26; 0.72]
Qiu, H, et al., 2018 I.	98	20.01	9.15	196	17.98	8.52	followup	4.00	0.23	[-0.01; 0.47]
Qiu, H, et al., 2018 I.	98	21.75	8.70	196	18.75	9.46	followup	24.00	0.32	[0.08; 0.57]
Kim, SH, et al., 2021	47	81.90	15.20	47	74.60	23.00	followup	8.00	0.37	[-0.04; 0.78]
Dirksen, S. et al, 2007	34	23.30	3.90	38	21.40	5.90	followup	10.00	0.37	[-0.10; 0.84]
Hernandez, EG, et al. 2018	28	18.82	5.79	28	16.40	5.07	followup	24.00	0.44	[-0.09; 0.97]
Hernandez, EG, et al. 2018	28	19.23	5.51	28	16.60	5.39	followup	8.00	0.48	[-0.06; 1.01]
Qiu, H, et al., 2018 I.	98	21.39	8.03	196	18.25	5.05	followup	12.00	0.50	[0.26; 0.75]
Elyasi, F, et al., 2021 II.	20	2.40	1.00	20	1.80	0.90	followup	24.00	0.62	[-0.02; 1.25]
Kim, YH, et al., 2017	30	75.60	26.20	30	58.30	27.20	followup	6.00	0.64	[0.12; 1.16]
Kim, YH, et al., 2017	30	75.00	23.10	30	54.40	28.00	followup	9.00	0.79	[0.27; 1.32]
Wu, Q, et al., 2021	43	81.65	8.30	43	72.36	7.51	followup	12.00	1.16	[0.70; 1.62]
Rahmani, S, et al. 2015	12	51.38	11.14	12	29.16	16.09	followup	8.00	1.55	[0.62; 2.48]
Random effect	3510			4313					0.26	[0.04; 0.48]
Prediction interval										[-0.30; 0.82]
**gastroenterological**										
Qin, X, et al., 2017	50	77.68	15.31	50	77.26	16.04	baseline	0.00	0.03	[-0.37; 0.42]
Baoyindeligeer, L.Z. et al. 2020	65	66.59	14.95	65	65.59	15.24	baseline	0.00	0.07	[-0.28; 0.41]
Cheung YL, et al. 2002	29	11.00	0.70	30	10.16	2.26	baseline	0.00	0.49	[-0.03; 1.01]
Qin, X, et al., 2017	50	80.24	13.46	50	79.67	13.13	followup	2.00	0.04	[-0.35; 0.43]
Cheung YL, et al. 2002	29	10.55	0.50	30	9.64	1.56	followup	5.00	0.77	[0.24; 1.30]
Cheung YL, et al. 2002	29	10.37	0.49	30	9.47	1.43	followup	10.00	0.83	[0.29; 1.36]
Baoyindeligeer, L.Z. et al. 2020	65	88.33	12.38	65	71.36	13.84	followup	2.00	1.28	[0.91; 1.66]
Random effect	317			320					0.61	[-0.29; 1.50]
Prediction interval										[-0.67; 1.88]
**gynaecological**										
Chan, et al. 2005	80	54.76	60.02	75	56.28	54.76	baseline	0.00	-0.03	[-0.34; 0.29]
Sandsund, C, et al., 2017	72	74.10	26.70	70	70.00	29.00	baseline	0.00	0.15	[-0.18; 0.48]
Powell, CB, et al., 2008	21	22.20	5.40	43	19.70	6.00	baseline	0.00	0.42	[-0.10; 0.95]
Chan, et al. 2005	80	76.19	54.76	75	87.23	54.76	followup	72.00	-0.20	[-0.52; 0.12]
Chan, et al. 2005	80	65.58	54.76	75	75.11	54.76	followup	24.00	-0.17	[-0.49; 0.14]
Chan, et al. 2005	80	76.62	54.76	75	85.06	54.76	followup	60.00	-0.15	[-0.47; 0.16]
Chan, et al. 2005	80	61.47	54.76	75	69.26	54.76	followup	12.00	-0.14	[-0.46; 0.17]
Chan, et al. 2005	80	80.52	54.76	75	83.77	54.76	followup	48.00	-0.06	[-0.37; 0.26]
Chan, et al. 2005	80	80.95	54.76	75	77.92	54.76	followup	36.00	0.06	[-0.26; 0.37]
Powell, CB, et al., 2008	21	21.30	5.60	43	19.10	7.30	followup	12.00	0.32	[-0.21; 0.84]
Sandsund, C, et al., 2017	72	85.00	21.40	70	74.90	29.80	followup	12.00	0.39	[0.06; 0.72]
Sandsund, C, et al., 2017	72	86.00	18.90	70	74.20	28.90	followup	24.00	0.48	[0.15; 0.82]
Random effect	818			821					0.37	[-0.20; 0.94]
Prediction interval										[-0.59; 1.33]
**prostate**										
Berglund, G. et al. 2007	39	80.30	22.60	150	84.00	22.10	baseline	0.00	-0.17	[-0.52; 0.19]
Penedo, FJ, et al., 2020	95	-19.85	4.09	97	-21.16	4.24	baseline	0.00	0.31	[0.03; 0.60]
Berglund, G. et al. 2007	39	77.00	23.70	150	83.50	23.70	followup	48.00	-0.27	[-0.63; 0.08]
Penedo, FJ, et al., 2020	95	-20.74	4.29	97	-22.05	4.43	followup	48.00	0.30	[0.01; 0.58]
Penedo, FJ, et al., 2020	95	-20.61	4.19	97	-21.91	4.33	followup	24.00	0.30	[0.02; 0.59]
Random effect	363			591					0.33	[-1.12; 1.77]
Prediction interval										[-1.64; 2.29]
**Random effect**	**5008**			**6045**					**0.33**	**[0.07; 0.58]**
**Prediction interval**										**[-0.26; 0.91]**

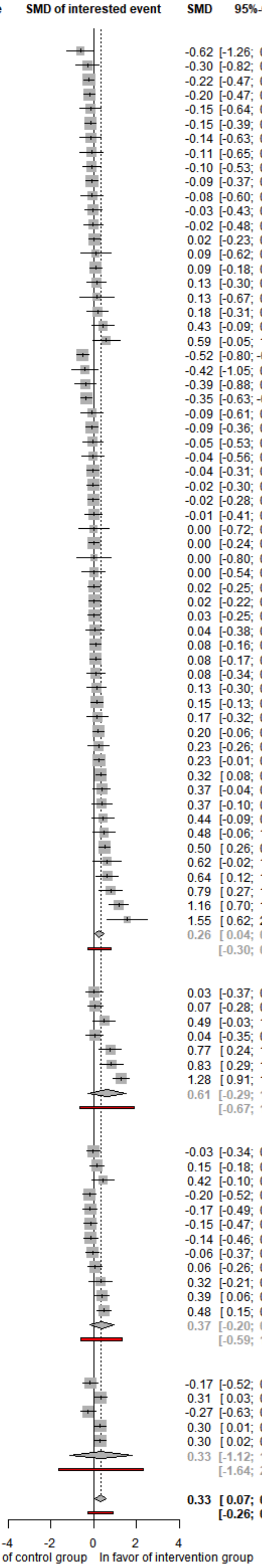

Figure S17.1. Subgroup analysis of the Social QoL. Forest plot represents the difference between the intervention vs. control group in the Social QoL domain with the cancer type subgroups as predicted at week 0 (post-intervention). SMD - Standardized mean difference, CI - confidence interval.

Figure S17.2.T12

Study	Experimental Patient N	Experimental Mean	Experimental SD	Control Patient N	Control Mean	Control SD	follow-up	Follow-up time	SMD	95%-CI
**breast**										
Ferguson, RJ, et al., 2012	19	6.26	2.05	21	7.31	1.21	baseline	0.00	-0.62	[-1.26; 0.02]
Peng, L, et al., 2022	28	76.79	15.27	29	81.61	16.87	baseline	0.00	-0.30	[-0.82; 0.23]
Qiu, H, et al., 2018 II.	98	17.84	5.10	196	18.85	4.22	baseline	0.00	-0.22	[-0.47; 0.02]
Hoffman, CJ, et al., 2012	102	17.59	5.91	109	18.78	6.01	baseline	0.00	-0.20	[-0.47; 0.07]
Heiney, SP, et al., 2003	33	5.60	2.00	33	5.90	1.90	baseline	0.00	-0.15	[-0.64; 0.33]
Qiu, H, et al., 2018 I.	98	18.13	5.70	196	18.85	4.22	baseline	0.00	-0.15	[-0.39; 0.09]
Ruiz-Vozmediano, J, et al.,2020	31	69.40	29.80	32	73.40	28.40	baseline	0.00	-0.14	[-0.63; 0.36]
Seliniotaki, T, et al., 2021	27	74.40	29.90	26	77.70	29.30	baseline	0.00	-0.11	[-0.65; 0.43]
Wu, Q, et al., 2021	43	53.77	5.51	43	54.34	5.53	baseline	0.00	-0.10	[-0.53; 0.32]
Antoni, MH, et al., 2006	92	877.61	158.55	107	891.17	152.16	baseline	0.00	-0.09	[-0.37; 0.19]
Hernandez, EG, et al. 2018	28	17.11	6.05	28	17.56	5.46	baseline	0.00	-0.08	[-0.60; 0.45]
Kim, SH, et al., 2021	47	73.70	81.90	47	75.50	22.00	baseline	0.00	-0.03	[-0.43; 0.37]
Dirksen, S. et al, 2007	34	22.10	4.90	38	22.20	6.10	baseline	0.00	-0.02	[-0.48; 0.44]
Klafke, N, et al., 2019	120	59.30	31.30	113	58.60	29.60	baseline	0.00	0.02	[-0.23; 0.28]
Elyasi, F, et al., 2021 I.	15	2.00	1.20	15	1.90	0.90	baseline	0.00	0.09	[-0.62; 0.81]
Klinkhammer-Schalke, M et al., 2012	100	71.21	33.50	100	67.99	34.25	baseline	0.00	0.09	[-0.18; 0.37]
Arving, C, et al., 2007	47	78.00	27.00	38	75.00	19.00	baseline	0.00	0.13	[-0.30; 0.55]
Rahmani, S, et al. 2015	12	34.72	13.22	12	33.33	7.11	baseline	0.00	0.13	[-0.67; 0.93]
Cengiz, HO, et al., 2023	32	20.16	5.97	33	19.13	5.49	baseline	0.00	0.18	[-0.31; 0.66]
Kim, YH, et al., 2017	30	72.80	25.30	30	62.80	20.80	baseline	0.00	0.43	[-0.09; 0.94]
Elyasi, F, et al., 2021 II.	20	2.50	1.10	20	1.90	0.90	baseline	0.00	0.59	[-0.05; 1.22]
Antoni, MH, et al., 2006	92	788.15	133.08	107	855.60	126.82	followup	48.00	-0.52	[-0.80; -0.23]
Ferguson, RJ, et al., 2012	19	6.75	1.78	21	7.47	1.59	followup	8.00	-0.42	[-1.05; 0.21]
Heiney, SP, et al., 2003	33	5.80	2.00	33	6.55	1.80	followup	16.00	-0.39	[-0.88; 0.10]
Antoni, MH, et al., 2006	92	832.88	119.31	107	873.38	114.05	followup	24.00	-0.35	[-0.63; -0.07]
Peng, L, et al., 2022	28	80.36	20.09	29	82.18	19.38	followup	0.10	-0.09	[-0.61; 0.43]
Klinkhammer-Schalke, M et al., 2012	100	78.90	27.54	100	81.14	24.07	followup	48.00	-0.09	[-0.36; 0.19]
Heiney, SP, et al., 2003	33	5.80	2.00	33	5.90	1.90	followup	6.00	-0.05	[-0.53; 0.43]
Peng, L, et al., 2022	28	79.17	16.49	29	79.89	20.11	followup	4.00	-0.04	[-0.56; 0.48]
Hoffman, CJ, et al., 2012	102	18.09	5.81	109	18.30	5.75	followup	12.00	-0.04	[-0.31; 0.23]
Klinkhammer-Schalke, M et al., 2012	100	66.00	33.00	100	66.74	31.76	followup	12.00	-0.02	[-0.30; 0.25]
Klafke, N, et al., 2019	120	54.50	29.60	113	55.10	29.50	followup	24.00	-0.02	[-0.28; 0.24]
Kim, SH, et al., 2021	47	80.30	17.60	47	80.40	18.50	followup	20.00	-0.01	[-0.41; 0.40]
Elyasi, F, et al., 2021 I.	15	1.80	0.90	15	1.80	0.90	followup	24.00	0.00	[-0.72; 0.72]
Qiu, H, et al., 2018 II.	98	17.98	8.92	196	17.98	8.52	followup	4.00	0.00	[-0.24; 0.24]
Rahmani, S, et al. 2015	12	12.00	54.16	12	12.00	34.72	followup	16.00	0.00	[-0.80; 0.80]
Seliniotaki, T, et al., 2021	27	74.60	35.90	26	74.60	29.30	followup	8.00	0.00	[-0.54; 0.54]
Hoffman, CJ, et al., 2012	102	18.36	5.65	109	18.26	5.88	followup	8.00	0.02	[-0.25; 0.29]
Qiu, H, et al., 2018 II.	98	18.97	8.92	196	18.75	9.46	followup	24.00	0.02	[-0.22; 0.27]
Klinkhammer-Schalke, M et al., 2012	100	72.20	28.78	100	71.46	29.03	followup	24.00	0.03	[-0.25; 0.30]
Arving, C, et al., 2007	47	76.00	25.00	38	75.00	19.00	followup	12.00	0.04	[-0.38; 0.47]
Qiu, H, et al., 2018 II.	98	18.77	8.03	196	18.25	5.05	followup	12.00	0.08	[-0.16; 0.33]
Klafke, N, et al., 2019	120	62.10	29.40	113	59.70	27.60	followup	12.00	0.08	[-0.17; 0.34]
Arving, C, et al., 2007	47	81.00	22.00	38	79.00	25.00	followup	4.00	0.08	[-0.34; 0.51]
Arving, C, et al., 2007	47	83.00	21.00	38	80.00	26.00	followup	24.00	0.13	[-0.30; 0.56]
Klinkhammer-Schalke, M et al., 2012	100	77.91	27.05	100	73.69	30.77	followup	36.00	0.15	[-0.13; 0.42]
Ruiz-Vozmediano, J, et al.,2020	31	83.30	24.70	32	78.70	28.50	followup	24.00	0.17	[-0.32; 0.67]
Klafke, N, et al., 2019	120	69.10	27.90	113	63.60	26.60	followup	48.00	0.20	[-0.06; 0.46]
Cengiz, HO, et al., 2023	32	20.36	5.38	33	19.06	5.74	followup	8.00	0.23	[-0.26; 0.72]
Qiu, H, et al., 2018 I.	98	20.01	9.15	196	17.98	8.52	followup	4.00	0.23	[-0.01; 0.47]
Qiu, H, et al., 2018 I.	98	21.75	8.70	196	18.75	9.46	followup	24.00	0.32	[0.08; 0.57]
Kim, SH, et al., 2021	47	81.90	15.20	47	74.60	23.00	followup	8.00	0.37	[-0.04; 0.78]
Dirksen, S. et al, 2007	34	23.30	3.90	38	21.40	5.90	followup	10.00	0.37	[-0.10; 0.84]
Hernandez, EG, et al. 2018	28	18.82	5.79	28	16.40	5.07	followup	24.00	0.44	[-0.09; 0.97]
Hernandez, EG, et al. 2018	28	19.23	5.51	28	16.60	5.39	followup	8.00	0.48	[-0.06; 1.01]
Qiu, H, et al., 2018 I.	98	21.39	8.03	196	18.25	5.05	followup	12.00	0.50	[0.26; 0.75]
Elyasi, F, et al., 2021 II.	20	2.40	1.00	20	1.80	0.90	followup	24.00	0.62	[-0.02; 1.25]
Kim, YH, et al., 2017	30	75.60	26.20	30	58.30	27.20	followup	6.00	0.64	[0.12; 1.16]
Kim, YH, et al., 2017	30	75.00	23.10	30	54.40	28.00	followup	9.00	0.79	[0.27; 1.32]
Wu, Q, et al., 2021	43	81.65	8.30	43	72.36	7.51	followup	12.00	1.16	[0.70; 1.62]
Rahmani, S, et al. 2015	12	51.38	11.14	12	29.16	16.09	followup	8.00	1.55	[0.62; 2.48]
Random effect	3510			4313					0.18	[0.04; 0.32]
Prediction interval										[-0.34; 0.70]
**gastroenterological**										
Qin, X, et al., 2017	50	77.68	15.31	50	77.26	16.04	baseline	0.00	0.03	[-0.37; 0.42]
Baoyindeligeer, L.Z. et al. 2020	65	66.59	14.95	65	65.59	15.24	baseline	0.00	0.07	[-0.28; 0.41]
Cheung YL, et al. 2002	29	11.00	0.70	30	10.16	2.26	baseline	0.00	0.49	[-0.03; 1.01]
Qin, X, et al., 2017	50	80.24	13.46	50	79.67	13.13	followup	2.00	0.04	[-0.35; 0.43]
Cheung YL, et al. 2002	29	10.55	0.50	30	9.64	1.56	followup	5.00	0.77	[0.24; 1.30]
Cheung YL, et al. 2002	29	10.37	0.49	30	9.47	1.43	followup	10.00	0.83	[0.29; 1.36]
Baoyindeligeer, L.Z. et al. 2020	65	88.33	12.38	65	71.36	13.84	followup	2.00	1.28	[0.91; 1.66]
Random effect	317			320					0.52	[-0.31; 1.36]
Prediction interval										[-0.75; 1.80]
**gynaecological**										
Chan, et al. 2005	80	54.76	60.02	75	56.28	54.76	baseline	0.00	-0.03	[-0.34; 0.29]
Sandsund, C, et al., 2017	72	74.10	26.70	70	70.00	29.00	baseline	0.00	0.15	[-0.18; 0.48]
Powell, CB, et al., 2008	21	22.20	5.40	43	19.70	6.00	baseline	0.00	0.42	[-0.10; 0.95]
Chan, et al. 2005	80	76.19	54.76	75	87.23	54.76	followup	72.00	-0.20	[-0.52; 0.12]
Chan, et al. 2005	80	65.58	54.76	75	75.11	54.76	followup	24.00	-0.17	[-0.49; 0.14]
Chan, et al. 2005	80	76.62	54.76	75	85.06	54.76	followup	60.00	-0.15	[-0.47; 0.16]
Chan, et al. 2005	80	61.47	54.76	75	69.26	54.76	followup	12.00	-0.14	[-0.46; 0.17]
Chan, et al. 2005	80	80.52	54.76	75	83.77	54.76	followup	48.00	-0.06	[-0.37; 0.26]
Chan, et al. 2005	80	80.95	54.76	75	77.92	54.76	followup	36.00	0.06	[-0.26; 0.37]
Powell, CB, et al., 2008	21	21.30	5.60	43	19.10	7.30	followup	12.00	0.32	[-0.21; 0.84]
Sandsund, C, et al., 2017	72	85.00	21.40	70	74.90	29.80	followup	12.00	0.39	[0.06; 0.72]
Sandsund, C, et al., 2017	72	86.00	18.90	70	74.20	28.90	followup	24.00	0.48	[0.15; 0.82]
Random effect	818			821					0.29	[-0.38; 0.96]
Prediction interval										[-0.92; 1.50]
**prostate**										
Berglund, G. et al. 2007	39	80.30	22.60	150	84.00	22.10	baseline	0.00	-0.17	[-0.52; 0.19]
Penedo, FJ, et al., 2020	95	-19.85	4.09	97	-21.16	4.24	baseline	0.00	0.31	[0.03; 0.60]
Berglund, G. et al. 2007	39	77.00	23.70	150	83.50	23.70	followup	48.00	-0.27	[-0.63; 0.08]
Penedo, FJ, et al., 2020	95	-20.74	4.29	97	-22.05	4.43	followup	48.00	0.30	[0.01; 0.58]
Penedo, FJ, et al., 2020	95	-20.61	4.19	97	-21.91	4.33	followup	24.00	0.30	[0.02; 0.59]
Random effect	363			591					0.25	[-2.08; 2.57]
Prediction interval										[-3.03; 3.52]
**Random effect**	**5008**			**6045**					**0.23**	**[0.09; 0.37]**
**Prediction interval**										**[-0.30; 0.76]**

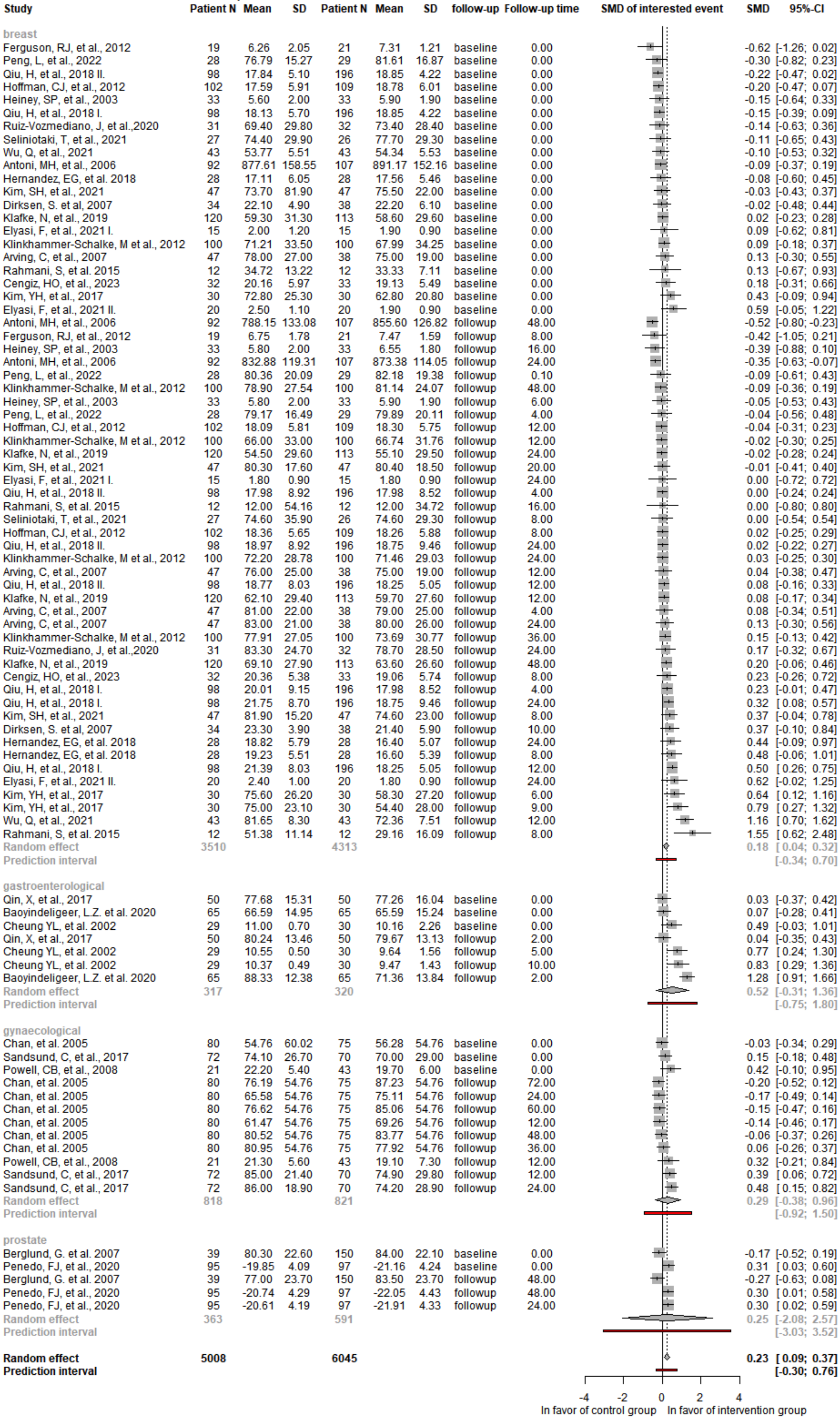

Figure S17.2. Subgroup analysis of the Social QoL. Forest plot represents the difference between the intervention vs. control group in the Social QoL domain with the cancer type subgroups as predicted at week 12 (post-intervention). SMD - Standardized mean difference, CI - confidence interval.

Figure S17.3.T24

Study	Experimental Patient N	Experimental Mean	Experimental SD	Control Patient N	Control Mean	Control SD	follow-up	Follow-up time	SMD	95%-CI
breast										
Ferguson, RJ, et al., 2012	19	6.26	2.05	21	7.31	1.21	baseline	0.00	-0.62	[-1.26; 0.02]
Peng, L, et al., 2022	28	76.79	15.27	29	81.61	16.87	baseline	0.00	-0.30	[-0.82; 0.23]
Qiu, H, et al., 2018 II.	98	17.84	5.10	196	18.85	4.22	baseline	0.00	-0.22	[-0.47; 0.02]
Hoffman, CJ, et al., 2012	102	17.59	5.91	109	18.78	6.01	baseline	0.00	-0.20	[-0.47; 0.07]
Heiney, SP, et al., 2003	33	5.60	2.00	33	5.90	1.90	baseline	0.00	-0.15	[-0.64; 0.33]
Qiu, H, et al., 2018 I.	98	18.13	5.70	196	18.85	4.22	baseline	0.00	-0.15	[-0.39; 0.09]
Ruiz-Vozmediano, J, et al.,2020	31	69.40	29.80	32	73.40	28.40	baseline	0.00	-0.14	[-0.63; 0.36]
Seliniotaki, T, et al., 2021	27	74.40	29.90	26	77.70	29.30	baseline	0.00	-0.11	[-0.65; 0.43]
Wu, Q, et al., 2021	43	53.77	5.51	43	54.34	5.53	baseline	0.00	-0.10	[-0.53; 0.32]
Antoni, MH, et al., 2006	92	877.61	158.55	107	891.17	152.16	baseline	0.00	-0.09	[-0.37; 0.19]
Hernandez, EG, et al. 2018	28	17.11	6.05	28	17.56	5.46	baseline	0.00	-0.08	[-0.60; 0.45]
Kim, SH, et al., 2021	47	73.70	81.90	47	75.50	22.00	baseline	0.00	-0.03	[-0.43; 0.37]
Dirksen, S. et al, 2007	34	22.10	4.90	38	22.20	6.10	baseline	0.00	-0.02	[-0.48; 0.44]
Klafke, N, et al., 2019	120	59.30	31.30	113	58.60	29.60	baseline	0.00	0.02	[-0.23; 0.28]
Elyasi, F, et al., 2021 I.	15	2.00	1.20	15	1.90	0.90	baseline	0.00	0.09	[-0.62; 0.81]
Klinkhammer-Schalke, M et al., 2012	100	71.21	33.50	100	67.99	34.25	baseline	0.00	0.09	[-0.18; 0.37]
Arving, C, et al., 2007	47	78.00	27.00	38	75.00	19.00	baseline	0.00	0.13	[-0.30; 0.55]
Rahmani, S, et al. 2015	12	34.72	13.22	12	33.33	7.11	baseline	0.00	0.13	[-0.67; 0.93]
Cengiz, HO, et al., 2023	32	20.16	5.97	33	19.13	5.49	baseline	0.00	0.18	[-0.31; 0.66]
Kim, YH, et al., 2017	30	72.80	25.30	30	62.80	20.80	baseline	0.00	0.43	[-0.09; 0.94]
Elyasi, F, et al., 2021 II.	20	2.50	1.10	20	1.90	0.90	baseline	0.00	0.59	[-0.05; 1.22]
Antoni, MH, et al., 2006	92	788.15	133.08	107	855.60	126.82	followup	48.00	-0.52	[-0.80; -0.23]
Ferguson, RJ, et al., 2012	19	6.75	1.78	21	7.47	1.59	followup	8.00	-0.42	[-1.05; 0.21]
Heiney, SP, et al., 2003	33	5.80	2.00	33	6.55	1.80	followup	16.00	-0.39	[-0.88; 0.10]
Antoni, MH, et al., 2006	92	832.88	119.31	107	873.38	114.05	followup	24.00	-0.35	[-0.63; -0.07]
Peng, L, et al., 2022	28	80.36	20.09	29	82.18	19.38	followup	0.10	-0.09	[-0.61; 0.43]
Klinkhammer-Schalke, M et al., 2012	100	78.90	27.54	100	81.14	24.07	followup	48.00	-0.09	[-0.36; 0.19]
Heiney, SP, et al., 2003	33	5.80	2.00	33	5.90	1.90	followup	6.00	-0.05	[-0.53; 0.43]
Peng, L, et al., 2022	28	79.17	16.49	29	79.89	20.11	followup	4.00	-0.04	[-0.56; 0.48]
Hoffman, CJ, et al., 2012	102	18.09	5.81	109	18.30	5.75	followup	12.00	-0.04	[-0.31; 0.23]
Klinkhammer-Schalke, M et al., 2012	100	66.00	33.00	100	66.74	31.76	followup	12.00	-0.02	[-0.30; 0.25]
Klafke, N, et al., 2019	120	54.50	29.60	113	55.10	29.50	followup	24.00	-0.02	[-0.28; 0.24]
Kim, SH, et al., 2021	47	80.30	17.60	47	80.40	18.50	followup	20.00	-0.01	[-0.41; 0.40]
Elyasi, F, et al., 2021 I.	15	1.80	0.90	15	1.80	0.90	followup	24.00	0.00	[-0.72; 0.72]
Qiu, H, et al., 2018 II.	98	17.98	8.92	196	17.98	8.52	followup	4.00	0.00	[-0.24; 0.24]
Rahmani, S, et al. 2015	12	12.00	54.16	12	12.00	34.72	followup	16.00	0.00	[-0.80; 0.80]
Seliniotaki, T, et al., 2021	27	74.60	35.90	26	74.60	29.30	followup	8.00	0.00	[-0.54; 0.54]
Hoffman, CJ, et al., 2012	102	18.36	5.65	109	18.26	5.88	followup	8.00	0.02	[-0.25; 0.29]
Qiu, H, et al., 2018 II.	98	18.97	8.92	196	18.75	9.46	followup	24.00	0.02	[-0.22; 0.27]
Klinkhammer-Schalke, M et al., 2012	100	72.20	28.78	100	71.46	29.03	followup	24.00	0.03	[-0.25; 0.30]
Arving, C, et al., 2007	47	76.00	25.00	38	75.00	19.00	followup	12.00	0.04	[-0.38; 0.47]
Qiu, H, et al., 2018 II.	98	18.77	8.03	196	18.25	5.05	followup	12.00	0.08	[-0.16; 0.33]
Klafke, N, et al., 2019	120	62.10	29.40	113	59.70	27.60	followup	12.00	0.08	[-0.17; 0.34]
Arving, C, et al., 2007	47	81.00	22.00	38	79.00	25.00	followup	4.00	0.08	[-0.34; 0.51]
Arving, C, et al., 2007	47	83.00	21.00	38	80.00	26.00	followup	24.00	0.13	[-0.30; 0.56]
Klinkhammer-Schalke, M et al., 2012	100	77.91	27.05	100	73.69	30.77	followup	36.00	0.15	[-0.13; 0.42]
Ruiz-Vozmediano, J, et al.,2020	31	83.30	24.70	32	78.70	28.50	followup	24.00	0.17	[-0.32; 0.67]
Klafke, N, et al., 2019	120	69.10	27.90	113	63.60	26.60	followup	48.00	0.20	[-0.06; 0.46]
Cengiz, HO, et al., 2023	32	20.36	5.38	33	19.06	5.74	followup	8.00	0.23	[-0.26; 0.72]
Qiu, H, et al., 2018 I.	98	20.01	9.15	196	17.98	8.52	followup	4.00	0.23	[-0.01; 0.47]
Qiu, H, et al., 2018 I.	98	21.75	8.70	196	18.75	9.46	followup	24.00	0.32	[0.08; 0.57]
Kim, SH, et al., 2021	47	81.90	15.20	47	74.60	23.00	followup	8.00	0.37	[-0.04; 0.78]
Dirksen, S. et al, 2007	34	23.30	3.90	38	21.40	5.90	followup	10.00	0.37	[-0.10; 0.84]
Hernandez, EG, et al. 2018	28	18.82	5.79	28	16.40	5.07	followup	24.00	0.44	[-0.09; 0.97]
Hernandez, EG, et al. 2018	28	19.23	5.51	28	16.60	5.39	followup	8.00	0.48	[-0.06; 1.01]
Qiu, H, et al., 2018 I.	98	21.39	8.03	196	18.25	5.05	followup	12.00	0.50	[0.26; 0.75]
Elyasi, F, et al., 2021 II.	20	2.40	1.00	20	1.80	0.90	followup	24.00	0.62	[-0.02; 1.25]
Kim, YH, et al., 2017	30	75.60	26.20	30	58.30	27.20	followup	6.00	0.64	[0.12; 1.16]
Kim, YH, et al., 2017	30	75.00	23.10	30	54.40	28.00	followup	9.00	0.79	[0.27; 1.32]
Wu, Q, et al., 2021	43	81.65	8.30	43	72.36	7.51	followup	12.00	1.16	[0.70; 1.62]
Rahmani, S, et al. 2015	12	51.38	11.14	12	29.16	16.09	followup	8.00	1.55	[0.62; 2.48]
Random effect	3510			4313					0.11	[-0.05; 0.26]
Prediction interval										[-0.42; 0.64]
gastroenterological										
Qin, X, et al., 2017	50	77.68	15.31	50	77.26	16.04	baseline	0.00	0.03	[-0.37; 0.42]
Baoyindeligeer, L.Z. et al. 2020	65	66.59	14.95	65	65.59	15.24	baseline	0.00	0.07	[-0.28; 0.41]
Cheung YL, et al. 2002	29	11.00	0.70	30	10.16	2.26	baseline	0.00	0.49	[-0.03; 1.01]
Qin, X, et al., 2017	50	80.24	13.46	50	79.67	13.13	followup	2.00	0.04	[-0.35; 0.43]
Cheung YL, et al. 2002	29	10.55	0.50	30	9.64	1.56	followup	5.00	0.77	[0.24; 1.30]
Cheung YL, et al. 2002	29	10.37	0.49	30	9.47	1.43	followup	10.00	0.83	[0.29; 1.36]
Baoyindeligeer, L.Z. et al. 2020	65	88.33	12.38	65	71.36	13.84	followup	2.00	1.28	[0.91; 1.66]
Random effect	317			320					0.45	[-0.23; 1.13]
Prediction interval										[-0.62; 1.53]
gynaecological										
Chan, et al. 2005	80	54.76	60.02	75	56.28	54.76	baseline	0.00	-0.03	[-0.34; 0.29]
Sandsund, C, et al., 2017	72	74.10	26.70	70	70.00	29.00	baseline	0.00	0.15	[-0.18; 0.48]
Powell, CB, et al., 2008	21	22.20	5.40	43	19.70	6.00	baseline	0.00	0.42	[-0.10; 0.95]
Chan, et al. 2005	80	76.19	54.76	75	87.23	54.76	followup	72.00	-0.20	[-0.52; 0.12]
Chan, et al. 2005	80	65.58	54.76	75	75.11	54.76	followup	24.00	-0.17	[-0.49; 0.14]
Chan, et al. 2005	80	76.62	54.76	75	85.06	54.76	followup	60.00	-0.15	[-0.47; 0.16]
Chan, et al. 2005	80	61.47	54.76	75	69.26	54.76	followup	12.00	-0.14	[-0.46; 0.17]
Chan, et al. 2005	80	80.52	54.76	75	83.77	54.76	followup	48.00	-0.06	[-0.37; 0.26]
Chan, et al. 2005	80	80.95	54.76	75	77.92	54.76	followup	36.00	0.06	[-0.26; 0.37]
Powell, CB, et al., 2008	21	21.30	5.60	43	19.10	7.30	followup	12.00	0.32	[-0.21; 0.84]
Sandsund, C, et al., 2017	72	85.00	21.40	70	74.90	29.80	followup	12.00	0.39	[0.06; 0.72]
Sandsund, C, et al., 2017	72	86.00	18.90	70	74.20	28.90	followup	24.00	0.48	[0.15; 0.82]
Random effect	818			821					0.22	[-0.44; 0.88]
Prediction interval										[-0.99; 1.43]
prostate										
Berglund, G. et al. 2007	39	80.30	22.60	150	84.00	22.10	baseline	0.00	-0.17	[-0.52; 0.19]
Penedo, FJ, et al., 2020	95	-19.85	4.09	97	-21.16	4.24	baseline	0.00	0.31	[0.03; 0.60]
Berglund, G. et al. 2007	39	77.00	23.70	150	83.50	23.70	followup	48.00	-0.27	[-0.63; 0.08]
Penedo, FJ, et al., 2020	95	-20.74	4.29	97	-22.05	4.43	followup	48.00	0.30	[0.01; 0.58]
Penedo, FJ, et al., 2020	95	-20.61	4.19	97	-21.91	4.33	followup	24.00	0.30	[0.02; 0.59]
Random effect	363			591					0.17	[-2.36; 2.71]
Prediction interval										[-3.43; 3.77]
**Random effect**	**5008**			**6045**					**0.15**	**[0.01; 0.29]**
**Prediction interval**										**[-0.39; 0.69]**

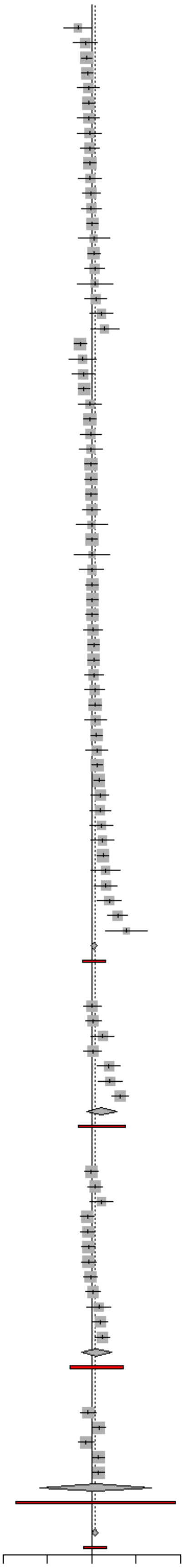

Figure S17.1. Subgroup analysis of the Social QoL. Forest plot represents the difference between the intervention vs. control group in the Social QoL domain with the cancer type subgroups as predicted at week 0 (post-intervention). SMD - Standardized mean difference, CI - confidence interval.

Figure S17.4.T48

Study	Experimental Patient N	Experimental Mean	Experimental SD	Control Patient N	Control Mean	Control SD	follow-up	Follow-up time	SMD	95%-CI
**breast**										
Ferguson, RJ, et al., 2012	19	6.26	2.05	21	7.31	1.21	baseline	0.00	-0.62	[-1.26; 0.02]
Peng, L, et al., 2022	28	76.79	15.27	29	81.61	16.87	baseline	0.00	-0.30	[-0.82; 0.23]
Qiu, H, et al., 2018 II.	98	17.84	5.10	196	18.85	4.22	baseline	0.00	-0.22	[-0.47; 0.02]
Hoffman, CJ, et al., 2012	102	17.59	5.91	109	18.78	6.01	baseline	0.00	-0.20	[-0.47; 0.07]
Heiney, SP, et al., 2003	33	5.60	2.00	33	5.90	1.90	baseline	0.00	-0.15	[-0.64; 0.33]
Qiu, H, et al., 2018 I.	98	18.13	5.70	196	18.85	4.22	baseline	0.00	-0.15	[-0.39; 0.09]
Ruiz-Vozmediano, J, et al.,2020	31	69.40	29.80	32	73.40	28.40	baseline	0.00	-0.14	[-0.63; 0.36]
Seliniotaki, T, et al., 2021	27	74.40	29.90	26	77.70	29.30	baseline	0.00	-0.11	[-0.65; 0.43]
Wu, Q, et al., 2021	43	53.77	5.51	43	54.34	5.53	baseline	0.00	-0.10	[-0.53; 0.32]
Antoni, MH, et al., 2006	92	877.61	158.55	107	891.17	152.16	baseline	0.00	-0.09	[-0.37; 0.19]
Hernandez, EG, et al. 2018	28	17.11	6.05	28	17.56	5.46	baseline	0.00	-0.08	[-0.60; 0.45]
Kim, SH, et al., 2021	47	73.70	81.90	47	75.50	22.00	baseline	0.00	-0.03	[-0.43; 0.37]
Dirksen, S. et al, 2007	34	22.10	4.90	38	22.20	6.10	baseline	0.00	-0.02	[-0.48; 0.44]
Klafke, N, et al., 2019	120	59.30	31.30	113	58.60	29.60	baseline	0.00	0.02	[-0.23; 0.28]
Elyasi, F, et al., 2021 I.	15	2.00	1.20	15	1.90	0.90	baseline	0.00	0.09	[-0.62; 0.81]
Klinkhammer-Schalke, M et al., 2012	100	71.21	33.50	100	67.99	34.25	baseline	0.00	0.09	[-0.18; 0.37]
Arving, C, et al., 2007	47	78.00	27.00	38	75.00	19.00	baseline	0.00	0.13	[-0.30; 0.55]
Rahmani, S, et al. 2015	12	34.72	13.22	12	33.33	7.11	baseline	0.00	0.13	[-0.67; 0.93]
Cengiz, HO, et al., 2023	32	20.16	5.97	33	19.13	5.49	baseline	0.00	0.18	[-0.31; 0.66]
Kim, YH, et al., 2017	30	72.80	25.30	30	62.80	20.80	baseline	0.00	0.43	[-0.09; 0.94]
Elyasi, F, et al., 2021 II.	20	2.50	1.10	20	1.90	0.90	baseline	0.00	0.59	[-0.05; 1.22]
Antoni, MH, et al., 2006	92	788.15	133.08	107	855.60	126.82	followup	48.00	-0.52	[-0.80; -0.23]
Ferguson, RJ, et al., 2012	19	6.75	1.78	21	7.47	1.59	followup	8.00	-0.42	[-1.05; 0.21]
Heiney, SP, et al., 2003	33	5.80	2.00	33	6.55	1.80	followup	16.00	-0.39	[-0.88; 0.10]
Antoni, MH, et al., 2006	92	832.88	119.31	107	873.38	114.05	followup	24.00	-0.35	[-0.63; -0.07]
Peng, L, et al., 2022	28	80.36	20.09	29	82.18	19.38	followup	0.10	-0.09	[-0.61; 0.43]
Klinkhammer-Schalke, M et al., 2012	100	78.90	27.54	100	81.14	24.07	followup	48.00	-0.09	[-0.36; 0.19]
Heiney, SP, et al., 2003	33	5.80	2.00	33	5.90	1.90	followup	6.00	-0.05	[-0.53; 0.43]
Peng, L, et al., 2022	28	79.17	16.49	29	79.89	20.11	followup	4.00	-0.04	[-0.56; 0.48]
Hoffman, CJ, et al., 2012	102	18.09	5.81	109	18.30	5.75	followup	12.00	-0.04	[-0.31; 0.23]
Klinkhammer-Schalke, M et al., 2012	100	66.00	33.00	100	66.74	31.76	followup	12.00	-0.02	[-0.30; 0.25]
Klafke, N, et al., 2019	120	54.50	29.60	113	55.10	29.50	followup	24.00	-0.02	[-0.28; 0.24]
Kim, SH, et al., 2021	47	80.30	17.60	47	80.40	18.50	followup	20.00	-0.01	[-0.41; 0.40]
Elyasi, F, et al., 2021 I.	15	1.80	0.90	15	1.80	0.90	followup	24.00	0.00	[-0.72; 0.72]
Qiu, H, et al., 2018 II.	98	17.98	8.92	196	17.98	8.52	followup	4.00	0.00	[-0.24; 0.24]
Rahmani, S, et al. 2015	12	12.00	54.16	12	12.00	34.72	followup	16.00	0.00	[-0.80; 0.80]
Seliniotaki, T, et al., 2021	27	74.60	35.90	26	74.60	29.30	followup	8.00	0.00	[-0.54; 0.54]
Hoffman, CJ, et al., 2012	102	18.36	5.65	109	18.26	5.88	followup	8.00	0.02	[-0.25; 0.29]
Qiu, H, et al., 2018 II.	98	18.97	8.92	196	18.75	9.46	followup	24.00	0.02	[-0.22; 0.27]
Klinkhammer-Schalke, M et al., 2012	100	72.20	28.78	100	71.46	29.03	followup	24.00	0.03	[-0.25; 0.30]
Arving, C, et al., 2007	47	76.00	25.00	38	75.00	19.00	followup	12.00	0.04	[-0.38; 0.47]
Qiu, H, et al., 2018 II.	98	18.77	8.03	196	18.25	5.05	followup	12.00	0.08	[-0.16; 0.33]
Klafke, N, et al., 2019	120	62.10	29.40	113	59.70	27.60	followup	12.00	0.08	[-0.17; 0.34]
Arving, C, et al., 2007	47	81.00	22.00	38	79.00	25.00	followup	4.00	0.08	[-0.34; 0.51]
Arving, C, et al., 2007	47	83.00	21.00	38	80.00	26.00	followup	24.00	0.13	[-0.30; 0.56]
Klinkhammer-Schalke, M et al., 2012	100	77.91	27.05	100	73.69	30.77	followup	36.00	0.15	[-0.13; 0.42]
Ruiz-Vozmediano, J, et al.,2020	31	83.30	24.70	32	78.70	28.50	followup	24.00	0.17	[-0.32; 0.67]
Klafke, N, et al., 2019	120	69.10	27.90	113	63.60	26.60	followup	48.00	0.20	[-0.06; 0.46]
Cengiz, HO, et al., 2023	32	20.36	5.38	33	19.06	5.74	followup	8.00	0.23	[-0.26; 0.72]
Qiu, H, et al., 2018 I.	98	20.01	9.15	196	17.98	8.52	followup	4.00	0.23	[-0.01; 0.47]
Qiu, H, et al., 2018 I.	98	21.75	8.70	196	18.75	9.46	followup	24.00	0.32	[0.08; 0.57]
Kim, SH, et al., 2021	47	81.90	15.20	47	74.60	23.00	followup	8.00	0.37	[-0.04; 0.78]
Dirksen, S. et al, 2007	34	23.30	3.90	38	21.40	5.90	followup	10.00	0.37	[-0.10; 0.84]
Hernandez, EG, et al. 2018	28	18.82	5.79	28	16.40	5.07	followup	24.00	0.44	[-0.09; 0.97]
Hernandez, EG, et al. 2018	28	19.23	5.51	28	16.60	5.39	followup	8.00	0.48	[-0.06; 1.01]
Qiu, H, et al., 2018 I.	98	21.39	8.03	196	18.25	5.05	followup	12.00	0.50	[0.26; 0.75]
Elyasi, F, et al., 2021 II.	20	2.40	1.00	20	1.80	0.90	followup	24.00	0.62	[-0.02; 1.25]
Kim, YH, et al., 2017	30	75.60	26.20	30	58.30	27.20	followup	6.00	0.64	[0.12; 1.16]
Kim, YH, et al., 2017	30	75.00	23.10	30	54.40	28.00	followup	9.00	0.79	[0.27; 1.32]
Wu, Q, et al., 2021	43	81.65	8.30	43	72.36	7.51	followup	12.00	1.16	[0.70; 1.62]
Rahmani, S, et al. 2015	12	51.38	11.14	12	29.16	16.09	followup	8.00	1.55	[0.62; 2.48]
Random effect	3510			4313					-0.02	[-0.21; 0.18]
Prediction interval										[-0.59; 0.56]
**gastroenterological**										
Qin, X, et al., 2017	50	77.68	15.31	50	77.26	16.04	baseline	0.00	0.03	[-0.37; 0.42]
Baoyindeligeer, L.Z. et al. 2020	65	66.59	14.95	65	65.59	15.24	baseline	0.00	0.07	[-0.28; 0.41]
Cheung YL, et al. 2002	29	11.00	0.70	30	10.16	2.26	baseline	0.00	0.49	[-0.03; 1.01]
Qin, X, et al., 2017	50	80.24	13.46	50	79.67	13.13	followup	2.00	0.04	[-0.35; 0.43]
Cheung YL, et al. 2002	29	10.55	0.50	30	9.64	1.56	followup	5.00	0.77	[0.24; 1.30]
Cheung YL, et al. 2002	29	10.37	0.49	30	9.47	1.43	followup	10.00	0.83	[0.29; 1.36]
Baoyindeligeer, L.Z. et al. 2020	65	88.33	12.38	65	71.36	13.84	followup	2.00	1.28	[0.91; 1.66]
Random effect	317			320					0.33	[-0.28; 0.94]
Prediction interval										[-0.63; 1.29]
**gynaecological**										
Chan, et al. 2005	80	54.76	60.02	75	56.28	54.76	baseline	0.00	-0.03	[-0.34; 0.29]
Sandsund, C, et al., 2017	72	74.10	26.70	70	70.00	29.00	baseline	0.00	0.15	[-0.18; 0.48]
Powell, CB, et al., 2008	21	22.20	5.40	43	19.70	6.00	baseline	0.00	0.42	[-0.10; 0.95]
Chan, et al. 2005	80	76.19	54.76	75	87.23	54.76	followup	72.00	-0.20	[-0.52; 0.12]
Chan, et al. 2005	80	65.58	54.76	75	75.11	54.76	followup	24.00	-0.17	[-0.49; 0.14]
Chan, et al. 2005	80	76.62	54.76	75	85.06	54.76	followup	60.00	-0.15	[-0.47; 0.16]
Chan, et al. 2005	80	61.47	54.76	75	69.26	54.76	followup	12.00	-0.14	[-0.46; 0.17]
Chan, et al. 2005	80	80.52	54.76	75	83.77	54.76	followup	48.00	-0.06	[-0.37; 0.26]
Chan, et al. 2005	80	80.95	54.76	75	77.92	54.76	followup	36.00	0.06	[-0.26; 0.37]
Powell, CB, et al., 2008	21	21.30	5.60	43	19.10	7.30	followup	12.00	0.32	[-0.21; 0.84]
Sandsund, C, et al., 2017	72	85.00	21.40	70	74.90	29.80	followup	12.00	0.39	[0.06; 0.72]
Sandsund, C, et al., 2017	72	86.00	18.90	70	74.20	28.90	followup	24.00	0.48	[0.15; 0.82]
Random effect	818			821					0.10	[-0.50; 0.69]
Prediction interval										[-1.03; 1.23]
**prostate**										
Berglund, G. et al. 2007	39	80.30	22.60	150	84.00	22.10	baseline	0.00	-0.17	[-0.52; 0.19]
Penedo, FJ, et al., 2020	95	-19.85	4.09	97	-21.16	4.24	baseline	0.00	0.31	[0.03; 0.60]
Berglund, G. et al. 2007	39	77.00	23.70	150	83.50	23.70	followup	48.00	-0.27	[-0.63; 0.08]
Penedo, FJ, et al., 2020	95	-20.74	4.29	97	-22.05	4.43	followup	48.00	0.30	[0.01; 0.58]
Penedo, FJ, et al., 2020	95	-20.61	4.19	97	-21.91	4.33	followup	24.00	0.30	[0.02; 0.59]
Random effect	363			591					0.05	[-2.47; 2.57]
Prediction interval										[-3.48; 3.59]
**Random effect**	**5008**			**6045**					**0.03**	**[-0.15; 0.21]**
**Prediction interval**										**[-0.57; 0.63]**

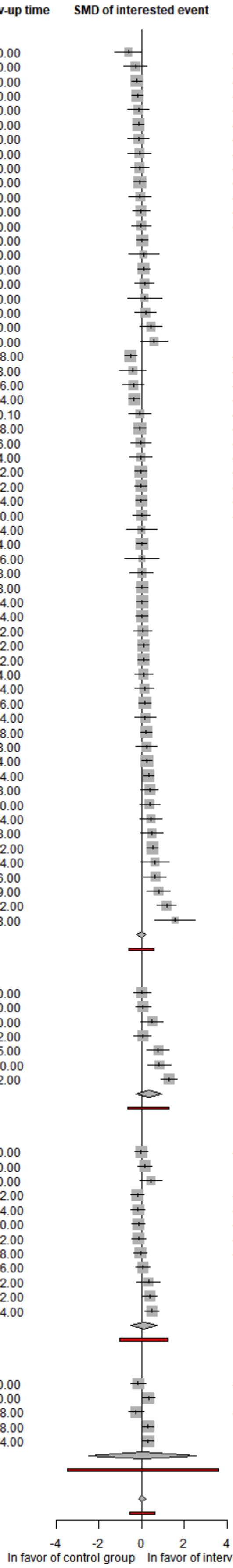

Figure S17.4. Subgroup analysis of the Social QoL. Forest plot represents the difference between the intervention vs. control group in the Social QoL domain with the cancer type subgroups as predicted at week 48 (post-intervention). SMD - Standardized mean difference, CI - confidence interval.

S18. Subgroup analysis of Physical QoL: Provider

Figure S18.1.T0

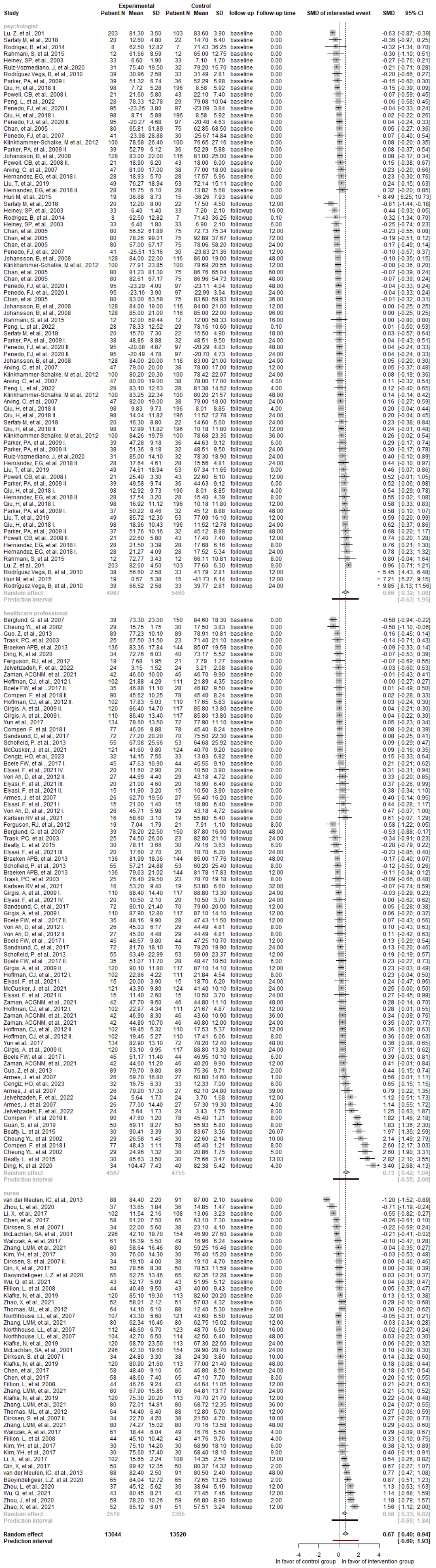

Figure S18.1. Forest plot represents the difference between the intervention vs. control group in the Physical QoL domain with the provider subgroups as predicted at week 0 (post-intervention). SMD - Standardized mean difference, CI - confidence interval.

Figure S18.2.T12

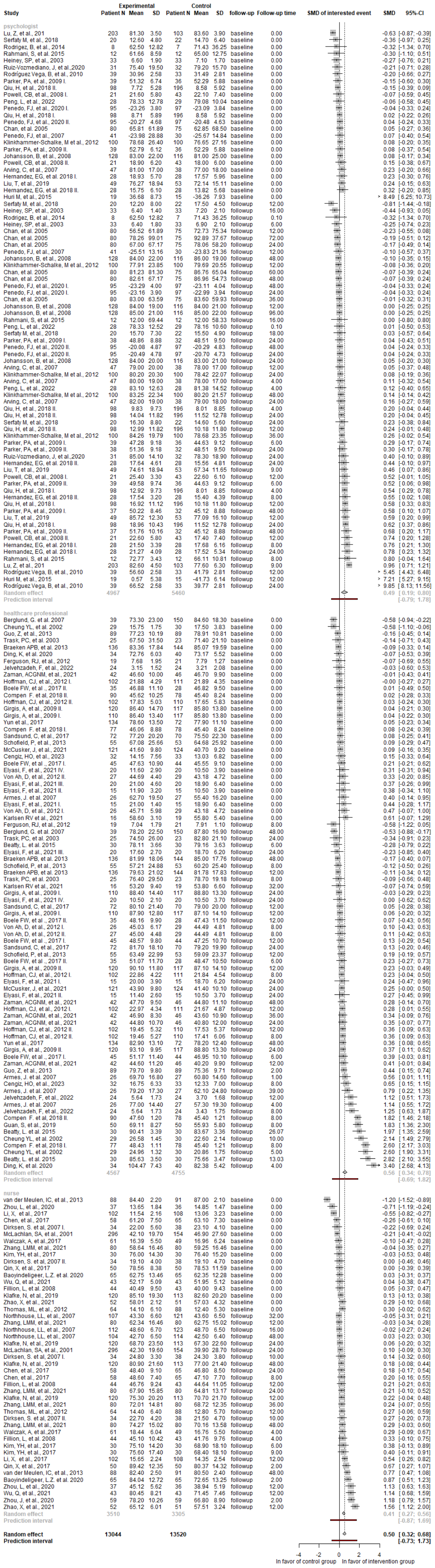

Figure S18.2. Forest plot represents the difference between the intervention vs. control group in the Physical QoL domain with the provider subgroups as predicted at week 12 (post-intervention). SMD - Standardized mean difference, CI - confidence interval

Figure S18.3.T24

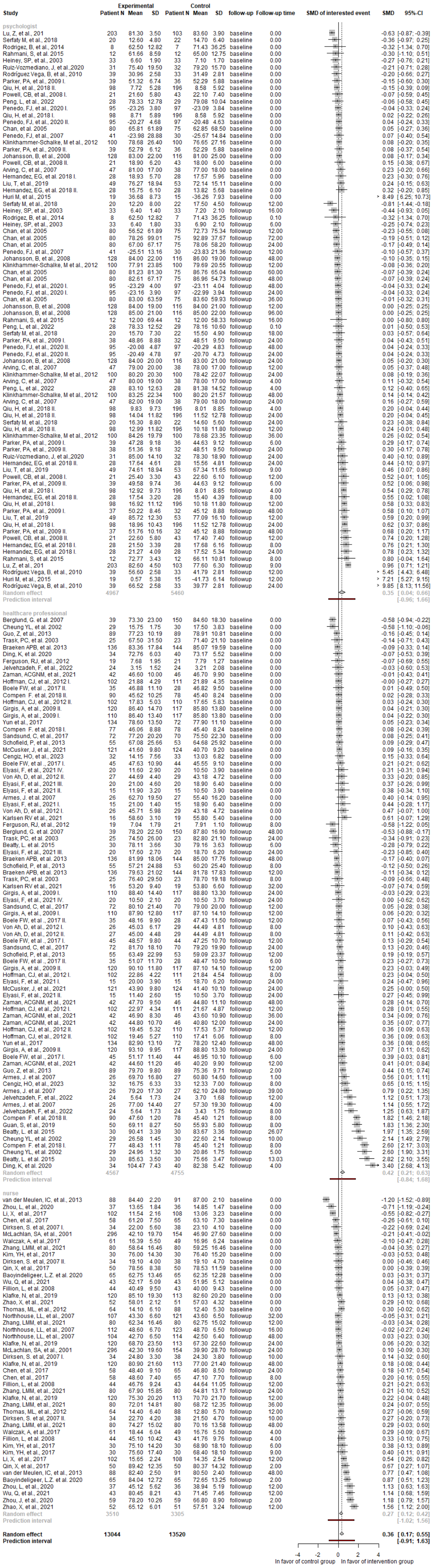

Figure S18.3. Forest plot represents the difference between the intervention vs. control group in the Physical QoL domain with the provider subgroups as predicted at week 24 (post-intervention). SMD - Standardized mean difference, CI - confidence interval

Figure S18.4.T48

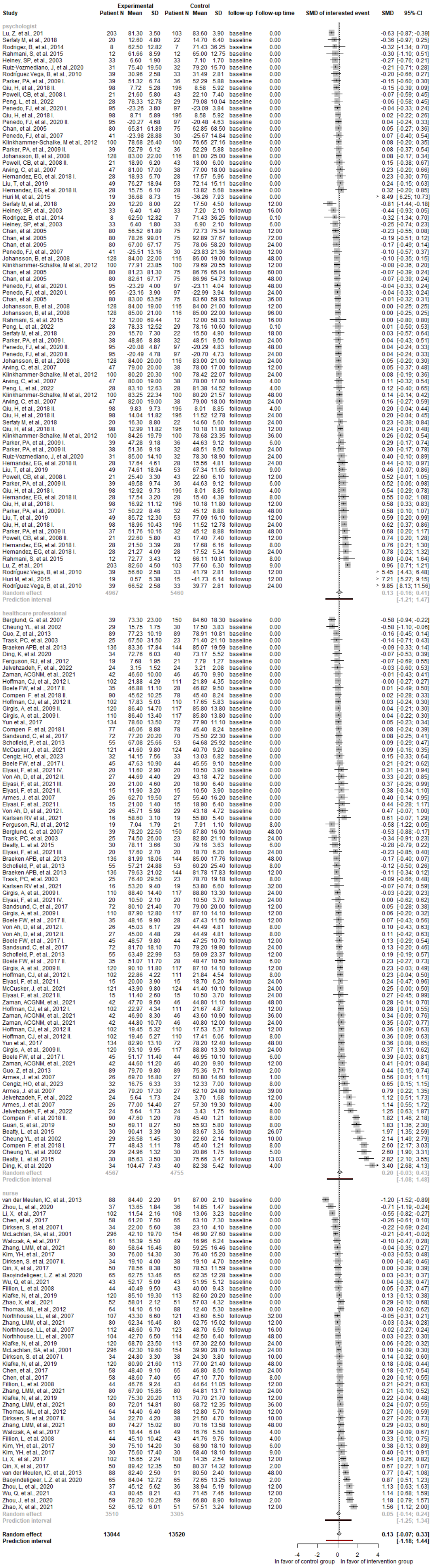

Figure S18.4. Forest plot represents the difference between the intervention vs. control group in the Physical QoL domain with the provider subgroups as predicted at week 48 (post-intervention). SMD - Standardized mean difference, CI - confidence interval

S19. Subgroup analysis of Physical QoL: Environment

Figure S19.1.T0

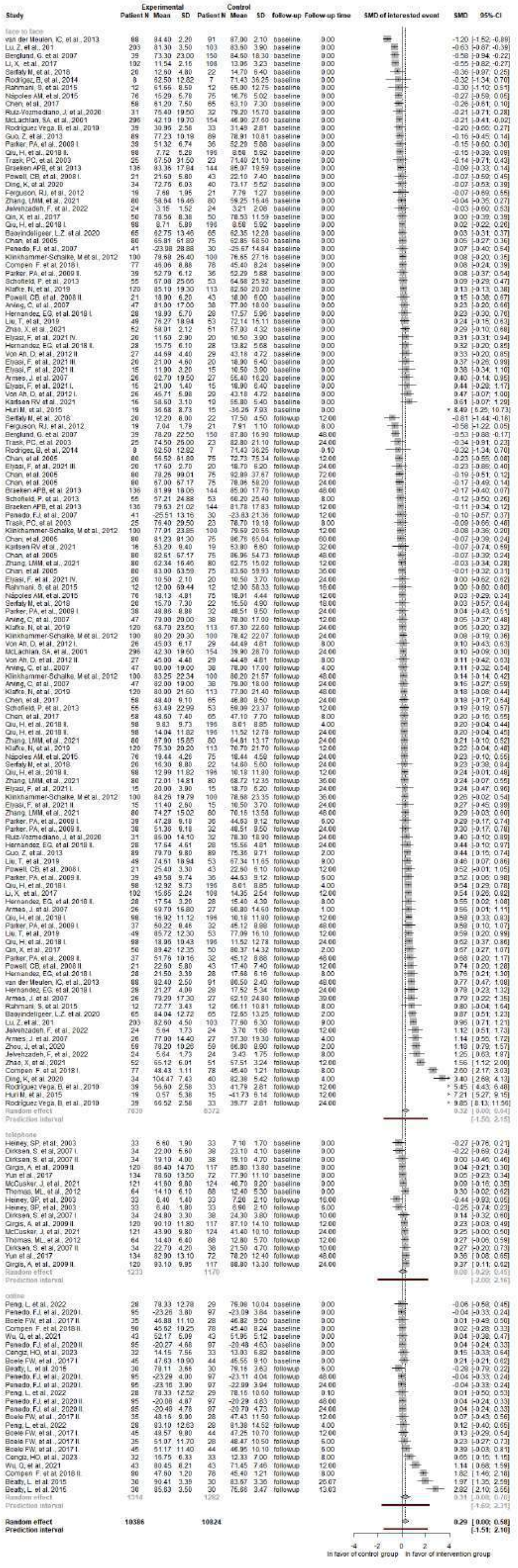

Figure S19.1. Forest plot represents the difference between the intervention vs. control group in the Physical QoL domain with the environment subgroups as predicted at week 0 (post-intervention). SMD - Standardized mean difference, CI - confidence interval.

Figure S19.2.T12

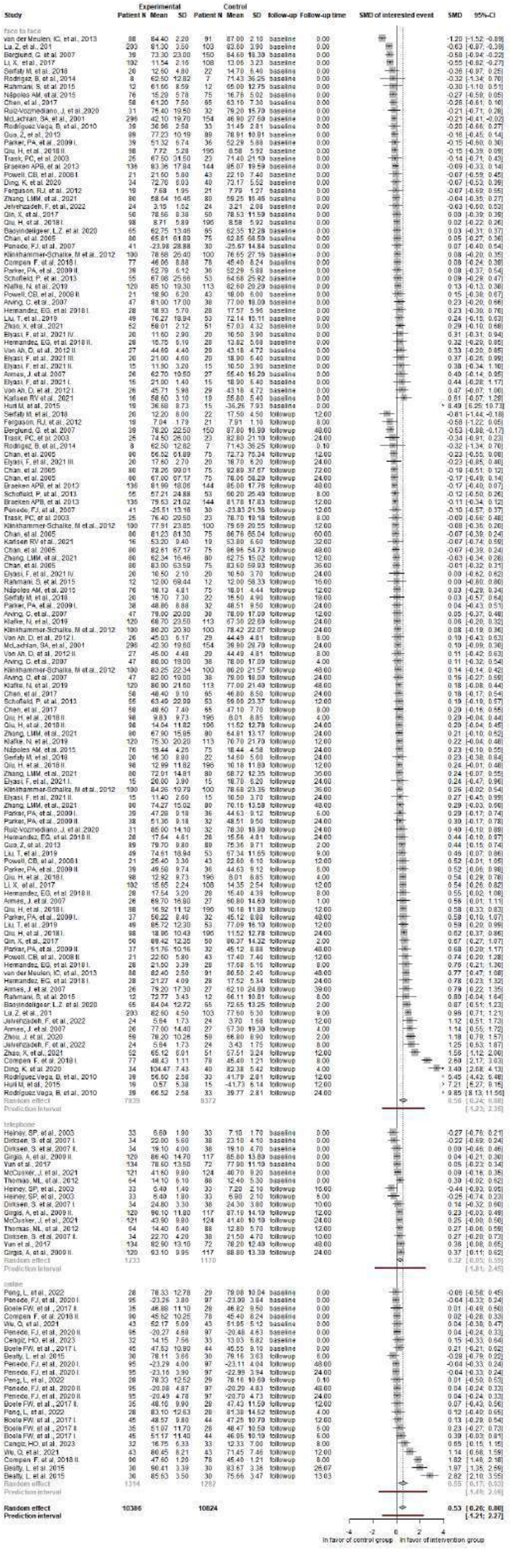

Figure S19.2. Forest plot represents the difference between the intervention vs. control group in the Physical QoL domain with the environment subgroups as predicted at week 12 (post-intervention). SMD - Standardized mean difference, CI - confidence interval.

Figure S19.3.T24

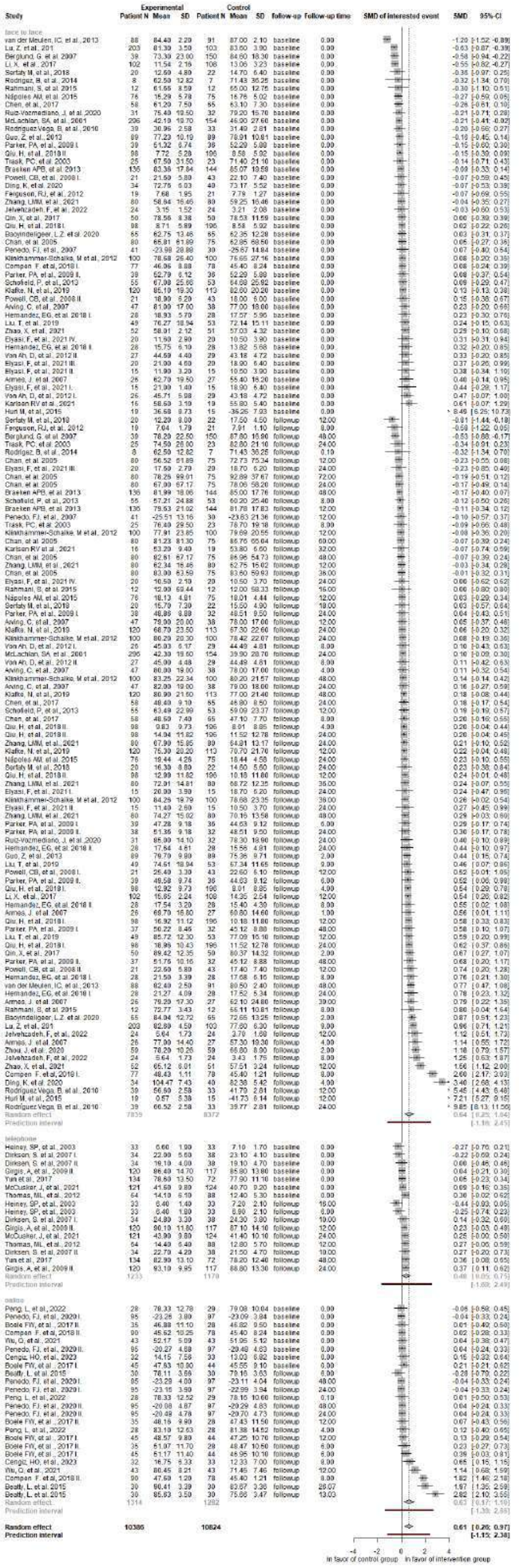

Figure S19.3. Forest plot represents the difference between the intervention vs. control group in the Physical QoL domain with the environment subgroups as predicted at week 24 (post-intervention). SMD - Standardized mean difference, CI - confidence interval.

Figure S19.4.T48

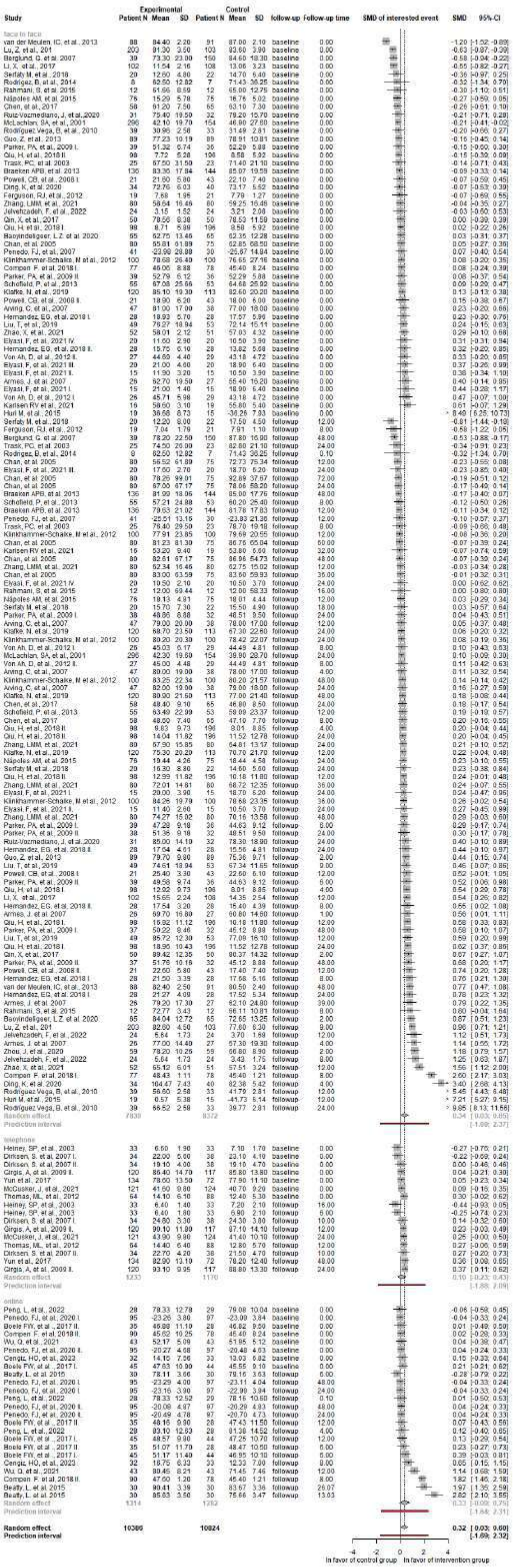

Figure S19.4. Forest plot represents the difference between the intervention vs. control group in the Physical QoL domain with the environment subgroups as predicted at week 48 (post-intervention). SMD - Standardized mean difference, CI - confidence interval.

S20. Subgroup analysis of Physical QoL: Type

Figure S20.1.T0

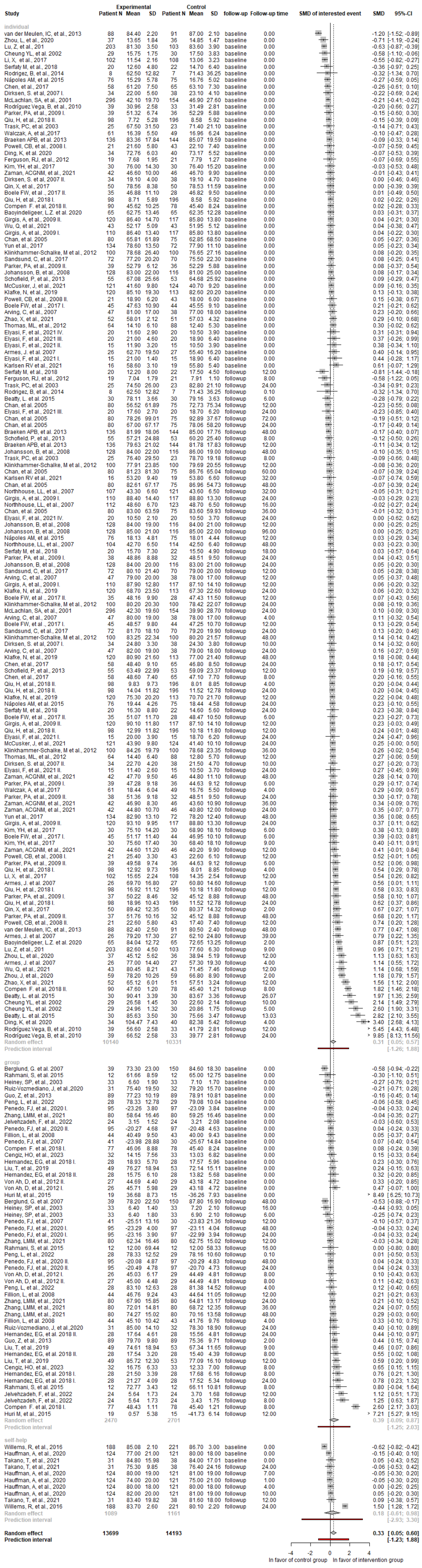

Figure S20.1. Forest plot represents the difference between the intervention vs. control group in the Physical QoL domain with the type subgroups as predicted at week 0 (post-intervention). SMD - Standardized mean difference, CI - confidence interval.

Figure S20.2.T12

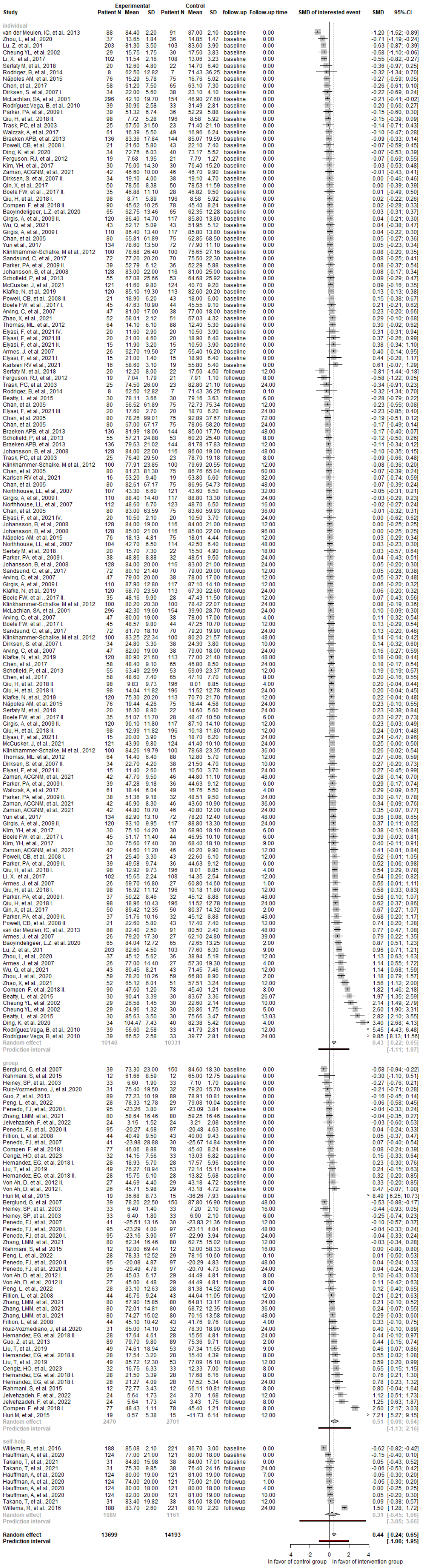

Figure S20.2. Forest plot represents the difference between the intervention vs. control group in the Physical QoL domain with the type subgroups as predicted at week 12 (post-intervention). SMD - Standardized mean difference, CI - confidence interval.

Figure S20.3.T24

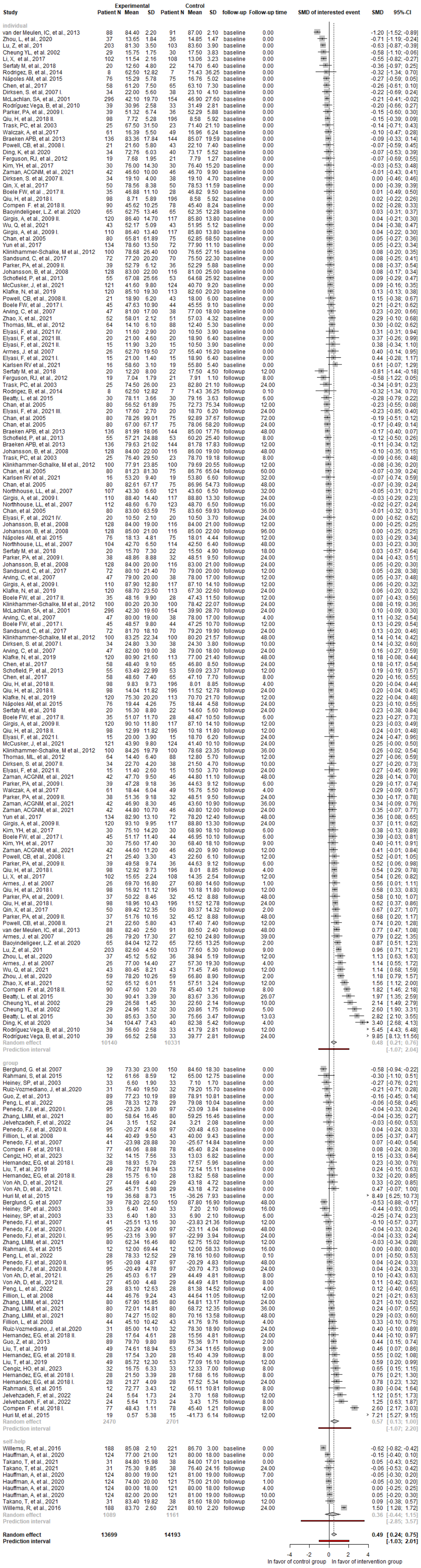

Figure S20.3. Forest plot represents the difference between the intervention vs. control group in the Physical QoL domain with the type subgroups as predicted at week 24 (post-intervention). SMD - Standardized mean difference, CI - confidence interval.

Figure S20.4.T48

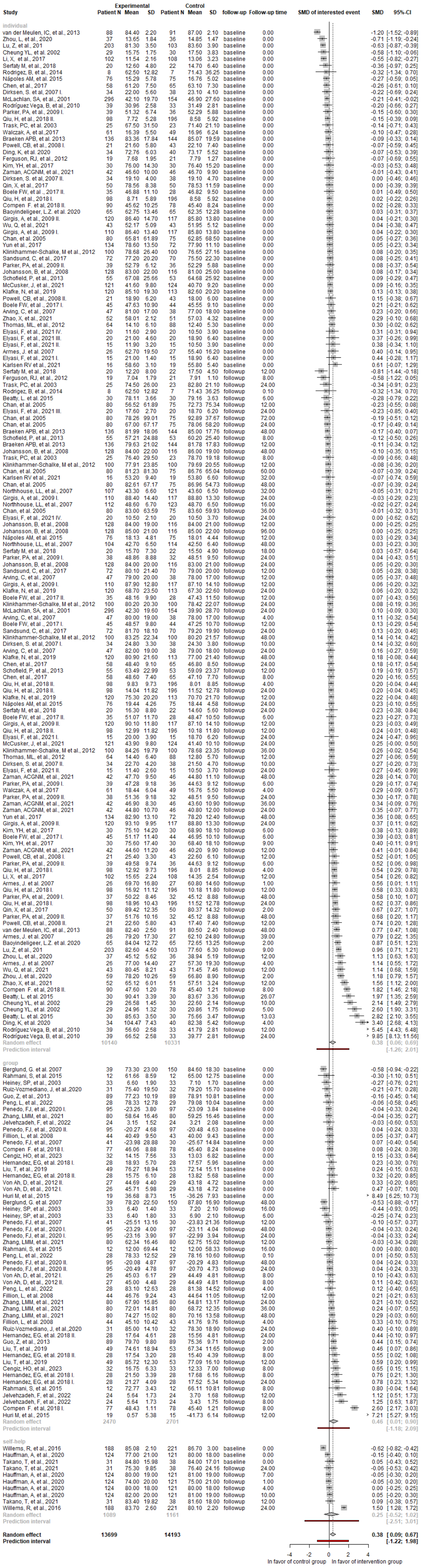

Figure S20.4. Forest plot represents the difference between the intervention vs. control group in the Physical QoL domain with the type subgroups as predicted at week 48 (post-intervention). SMD - Standardized mean difference, CI - confidence interval.

S21. Subgroup analysis of Physical QoL: Cancer stage

Figure S21.1.T0

Study	Experimental Patient N	Experimental Mean	Experimental SD	Control Patient N	Control Mean	Control SD	follow-up	Follow-up time	SMD	95%-CI
early										
Willems, R, et al., 2016	188	85.08	2.10	221	86.70	3.00	baseline	0.00	-0.62	[-0.82; -0.42]
Rodrigez, B, et al., 2014	8	62.50	12.82	7	71.43	36.25	baseline	0.00	-0.32	[-1.34; 0.70]
Ruiz-Vozmediano, J, et al.,2020	31	75.40	19.50	32	79.20	15.70	baseline	0.00	-0.21	[-0.71; 0.28]
Rodríguez Vega, B, et al., 2010	39	30.96	2.58	33	31.49	2.81	baseline	0.00	-0.20	[-0.66; 0.27]
Parker, PA, et al., 2009 I.	39	51.32	6.74	36	52.29	5.88	baseline	0.00	-0.15	[-0.60; 0.30]
Ferguson, RJ, et al., 2012	19	7.68	1.95	21	7.79	1.27	baseline	0.00	-0.07	[-0.69; 0.55]
Zhang, LMM, et al., 2021	80	58.64	16.46	80	59.25	16.46	baseline	0.00	-0.04	[-0.35; 0.27]
Wu, Q, et al., 2021	43	52.17	5.09	43	51.95	5.12	baseline	0.00	0.04	[-0.38; 0.47]
Takano, T, et al., 2021	31	84.80	15.98	38	84.00	17.01	baseline	0.00	0.05	[-0.43; 0.52]
Fillion, L, et al. 2008	44	40.49	9.50	43	40.00	9.43	baseline	0.00	0.05	[-0.37; 0.47]
Yun et al., 2017	134	78.60	13.50	72	77.90	11.10	baseline	0.00	0.05	[-0.23; 0.34]
Parker, PA, et al., 2009 II.	39	52.79	6.12	36	52.29	5.88	baseline	0.00	0.08	[-0.37; 0.54]
Cengiz, HO, et al., 2023	32	14.15	7.56	33	13.03	6.82	baseline	0.00	0.15	[-0.33; 0.64]
Liu, T, et al., 2019	49	76.27	18.94	53	72.14	15.11	baseline	0.00	0.24	[-0.15; 0.63]
Zhao, X, et al., 2021	52	58.01	2.12	51	57.03	4.32	baseline	0.00	0.29	[-0.10; 0.68]
Ferguson, RJ, et al., 2012	19	7.04	1.79	21	7.91	1.10	followup	8.00	-0.58	[-1.22; 0.05]
Rodrigez, B, et al., 2014	8	62.50	12.82	7	71.43	36.25	followup	0.10	-0.32	[-1.34; 0.70]
Beatty, L. et al. 2015	30	78.11	3.66	30	79.16	3.63	followup	6.00	-0.28	[-0.79; 0.22]
Takano, T, et al., 2021	31	75.30	9.85	38	76.40	24.16	followup	24.00	-0.06	[-0.53; 0.42]
Zhang, LMM, et al., 2021	80	62.34	16.46	80	62.75	15.02	followup	12.00	-0.03	[-0.34; 0.28]
Parker, PA, et al., 2009 I.	38	48.86	8.88	32	48.51	9.50	followup	24.00	0.04	[-0.43; 0.51]
Takano, T, et al., 2021	31	83.40	19.82	38	81.60	18.00	followup	12.00	0.09	[-0.38; 0.57]
Fillion, L, et al. 2008	44	46.76	9.24	43	44.64	11.05	followup	12.00	0.21	[-0.21; 0.63]
Zhang, LMM, et al., 2021	80	67.90	15.85	80	64.81	13.17	followup	24.00	0.21	[-0.10; 0.52]
Zhang, LMM, et al., 2021	80	72.01	14.81	80	68.72	12.35	followup	36.00	0.24	[-0.07; 0.55]
Zhang, LMM, et al., 2021	80	74.27	15.02	80	70.16	13.58	followup	48.00	0.29	[-0.03; 0.60]
Parker, PA, et al., 2009 I.	39	47.28	9.18	36	44.63	9.12	followup	6.00	0.29	[-0.17; 0.74]
Parker, PA, et al., 2009 II.	38	51.36	9.18	32	48.51	9.50	followup	24.00	0.30	[-0.17; 0.78]
Fillion, L, et al. 2008	44	45.10	10.42	43	41.76	9.76	followup	4.00	0.33	[-0.10; 0.75]
Yun et al., 2017	134	82.90	13.10	72	78.20	12.40	followup	48.00	0.36	[0.08; 0.65]
Ruiz-Vozmediano, J, et al.,2020	31	85.00	14.10	32	78.30	18.90	followup	24.00	0.40	[-0.10; 0.89]
Liu, T, et al., 2019	49	74.61	18.94	53	67.34	11.65	followup	9.00	0.46	[0.07; 0.86]
Parker, PA, et al., 2009 II.	39	49.58	9.74	36	44.63	9.12	followup	6.00	0.52	[0.06; 0.98]
Parker, PA, et al., 2009 I.	37	50.22	8.46	32	45.12	8.88	followup	48.00	0.58	[0.10; 1.07]
Liu, T, et al., 2019	49	85.72	12.30	53	77.09	16.10	followup	12.00	0.59	[0.20; 0.99]
Cengiz, HO, et al., 2023	32	16.75	6.33	33	12.33	7.00	followup	8.00	0.65	[0.15; 1.15]
Parker, PA, et al., 2009 II.	37	51.76	10.16	32	45.12	8.88	followup	48.00	0.68	[0.20; 1.17]
Wu, Q, et al., 2021	43	80.45	8.21	43	71.45	7.46	followup	12.00	1.14	[0.68; 1.59]
Willems, R, et al., 2016	188	83.70	2.60	221	80.10	2.20	followup	24.00	1.50	[1.28; 1.72]
Zhao, X, et al., 2021	52	65.12	6.01	51	57.51	3.24	followup	12.00	1.56	[1.12; 2.00]
Guan, S, et al., 2019	50	69.11	8.27	50	55.93	5.80	followup	8.00	1.83	[1.36; 2.30]
Beatty, L. et al. 2015	30	90.41	3.39	30	83.67	3.36	followup	26.07	1.97	[1.35; 2.59]
Beatty, L. et al. 2015	30	85.63	3.50	30	75.66	3.47	followup	13.03	2.82	[2.10; 3.55]
Rodríguez Vega, B, et al., 2010	39	56.60	2.58	33	41.79	2.81	followup	12.00	> 5.45	[4.43; 6.48]
Rodríguez Vega, B, et al., 2010	39	66.52	2.58	33	39.77	2.81	followup	24.00	> 9.85	[8.13; 11.56]
Random effect	2349			2273					0.30	[0.00; 0.60]
Prediction interval										[-2.50; 3.10]
advanced										
Lu, Z, et al., 201	203	81.30	3.50	103	83.60	3.90	baseline	0.00	-0.63	[-0.87; -0.39]
Serfaty M, et al., 2018	20	12.60	4.80	22	14.70	6.40	baseline	0.00	-0.36	[-0.97; 0.25]
Heiney, SP, et al., 2003	33	6.60	1.90	33	7.10	1.70	baseline	0.00	-0.27	[-0.76; 0.21]
Walczak, A, et al.,2017	61	16.39	5.50	49	16.96	6.24	baseline	0.00	-0.10	[-0.47; 0.28]
Penedo, FJ, et al., 2020 I.	95	-23.26	3.80	97	-23.09	3.84	baseline	0.00	-0.04	[-0.33; 0.24]
Boele FW, et al ., 2017 II.	35	46.88	11.10	28	46.82	9.50	baseline	0.00	0.01	[-0.49; 0.50]
Penedo, FJ, et al., 2020 II.	95	-20.27	4.68	97	-20.48	4.63	baseline	0.00	0.04	[-0.24; 0.33]
Penedo, FJ, et al., 2007	41	-23.98	28.88	30	-25.67	14.84	baseline	0.00	0.07	[-0.40; 0.54]
Schofield, P, et al., 2013	55	67.08	25.66	53	64.68	25.92	baseline	0.00	0.09	[-0.29; 0.47]
Boele FW, et al ., 2017 I.	45	47.63	10.90	44	45.55	9.10	baseline	0.00	0.21	[-0.21; 0.62]
Huri M, et al., 2015	19	36.68	8.73	15	-36.26	7.93	baseline	0.00	> 8.49	[6.25; 10.73]
Serfaty M, et al., 2018	20	12.20	8.00	22	17.50	4.50	followup	12.00	-0.81	[-1.44; -0.18]
Heiney, SP, et al., 2003	33	6.40	1.40	33	7.20	2.10	followup	16.00	-0.44	[-0.93; 0.05]
Heiney, SP, et al., 2003	33	6.40	1.80	33	6.90	2.10	followup	6.00	-0.25	[-0.74; 0.23]
Schofield, P, et al., 2013	55	57.21	24.88	53	60.20	25.40	followup	8.00	-0.12	[-0.50; 0.26]
Penedo, FJ, et al., 2007	41	-25.51	13.16	30	-23.83	21.36	followup	12.00	-0.10	[-0.57; 0.37]
Penedo, FJ, et al., 2020 I.	95	-23.29	4.00	97	-23.11	4.04	followup	48.00	-0.04	[-0.33; 0.24]
Penedo, FJ, et al., 2020 I.	95	-23.16	3.90	97	-22.99	3.94	followup	24.00	-0.04	[-0.33; 0.24]
Serfaty M, et al., 2018	20	15.70	7.30	22	15.50	4.90	followup	18.00	0.03	[-0.57; 0.64]
Penedo, FJ, et al., 2020 II.	95	-20.08	4.87	97	-20.29	4.83	followup	48.00	0.04	[-0.24; 0.33]
Penedo, FJ, et al., 2020 II.	95	-20.49	4.78	97	-20.70	4.73	followup	24.00	0.04	[-0.24; 0.33]
Boele FW, et al ., 2017 II.	35	48.16	9.90	28	47.43	11.50	followup	12.00	0.07	[-0.43; 0.56]
Boele FW, et al ., 2017 I.	45	48.57	9.80	44	47.25	10.70	followup	12.00	0.13	[-0.29; 0.54]
Serfaty M, et al., 2018	20	16.30	8.80	22	14.60	5.60	followup	24.00	0.23	[-0.38; 0.84]
Boele FW, et al ., 2017 II.	35	51.07	11.70	28	48.47	10.50	followup	6.00	0.23	[-0.27; 0.73]
Walczak, A, et al.,2017	61	18.44	6.04	49	16.76	5.50	followup	4.00	0.29	[-0.09; 0.67]
Boele FW, et al ., 2017 I.	45	51.17	11.40	44	46.95	10.10	followup	6.00	0.39	[-0.03; 0.81]
Lu, Z, et al., 201	203	82.60	4.50	103	77.60	6.30	followup	9.00	0.96	[0.71; 1.21]
Huri M, et al., 2015	19	0.57	5.38	15	-41.73	6.14	followup	12.00	> 7.21	[5.27; 9.15]
Random effect	1747			1485					0.27	[-0.94; 1.48]
Prediction interval										[-2.87; 3.41]
survivor										
Chen, et al., 2017	58	61.20	7.50	65	63.10	7.30	baseline	0.00	-0.26	[-0.61; 0.10]
Qiu, H, et al., 2018 II.	98	7.72	5.28	196	8.58	5.92	baseline	0.00	-0.15	[-0.39; 0.09]
Peng, L, et al., 2022	28	78.33	12.78	29	79.08	10.04	baseline	0.00	-0.06	[-0.58; 0.45]
Jelvehzadeh, F, et al., 2022	24	3.15	1.52	24	3.21	2.08	baseline	0.00	-0.03	[-0.60; 0.53]
Qiu, H, et al., 2018 I.	98	8.71	5.89	196	8.58	5.92	baseline	0.00	0.02	[-0.22; 0.26]
McCusker, J, et al., 2021	121	41.60	9.80	124	40.70	9.20	baseline	0.00	0.09	[-0.16; 0.35]
Von Ah, D, et al., 2012 II.	27	44.69	4.40	29	43.18	4.72	baseline	0.00	0.33	[-0.20; 0.85]
Von Ah, D, et al., 2012 I.	26	45.71	5.98	29	43.18	4.72	baseline	0.00	0.47	[-0.07; 1.00]
Peng, L, et al., 2022	28	78.33	12.52	29	78.16	10.60	followup	0.10	0.01	[-0.50; 0.53]
Von Ah, D, et al., 2012 I.	26	45.03	6.17	29	44.49	4.81	followup	8.00	0.10	[-0.43; 0.63]
Von Ah, D, et al., 2012 II.	27	45.00	4.48	29	44.49	4.81	followup	8.00	0.11	[-0.42; 0.63]
Peng, L, et al., 2022	28	83.10	12.63	28	81.38	14.52	followup	4.00	0.12	[-0.40; 0.65]
Chen, et al., 2017	58	48.40	9.10	65	46.80	8.50	followup	24.00	0.18	[-0.17; 0.54]
Chen, et al., 2017	58	48.60	7.40	65	47.10	7.70	followup	8.00	0.20	[-0.16; 0.55]
Qiu, H, et al., 2018 II.	98	9.83	9.73	196	8.01	8.85	followup	4.00	0.20	[-0.04; 0.44]
Qiu, H, et al., 2018 II.	98	14.04	11.82	196	11.52	12.78	followup	24.00	0.20	[-0.04; 0.45]
Qiu, H, et al., 2018 II.	98	12.99	11.82	196	10.18	11.80	followup	12.00	0.24	[-0.01; 0.48]
McCusker, J, et al., 2021	121	43.90	9.80	124	41.40	10.10	followup	24.00	0.25	[-0.00; 0.50]
Qiu, H, et al., 2018 I.	98	12.92	9.73	196	8.01	8.85	followup	4.00	0.54	[0.29; 0.78]
Qiu, H, et al., 2018 I.	98	16.92	11.12	196	10.18	11.80	followup	12.00	0.58	[0.33; 0.83]
Qiu, H, et al., 2018 I.	98	18.96	10.43	196	11.52	12.78	followup	24.00	0.62	[0.37; 0.86]
Jelvehzadeh, F, et al., 2022	24	5.64	1.73	24	3.70	1.68	followup	12.00	1.12	[0.51; 1.73]
Jelvehzadeh, F, et al., 2022	24	5.64	1.73	24	3.43	1.75	followup	8.00	1.25	[0.63; 1.87]
Random effect	1462			2285					-0.05	[-0.43; 0.33]
Prediction interval										[-3.16; 3.06]
**Random effect**	**5558**			**6043**					**0.21**	**[-0.16; 0.58]**
**Prediction interval**										**[-2.47; 2.89]**

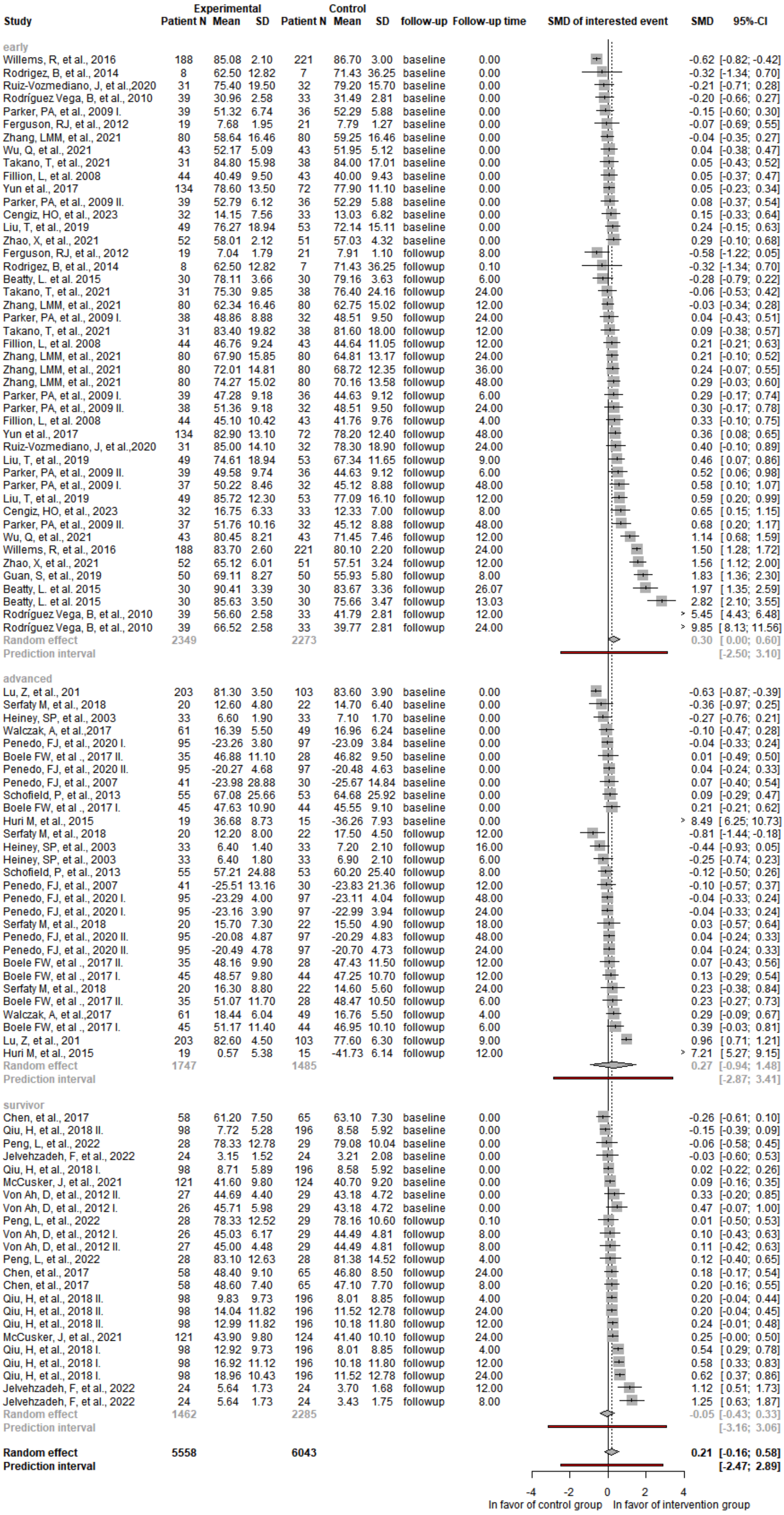

Figure S21.1. Forest plot represents the difference between the intervention vs. control group in the Physical QoL domain with the cancer stage subgroups as predicted at week 0 (post-intervention). SMD - Standardized mean difference, CI - confidence interval.

Figure S21.2.T12

Study	Experimental Patient N	Experimental Mean	Experimental SD	Control Patient N	Control Mean	Control SD	follow-up	Follow-up time	SMD	95%-CI
**early**										
Willems, R, et al., 2016	188	85.08	2.10	221	86.70	3.00	baseline	0.00	-0.62	[-0.82; -0.42]
Rodrigez, B, et al., 2014	8	62.50	12.82	7	71.43	36.25	baseline	0.00	-0.32	[-1.34; 0.70]
Ruiz-Vozmediano, J, et al.,2020	31	75.40	19.50	32	79.20	15.70	baseline	0.00	-0.21	[-0.71; 0.28]
Rodríguez Vega, B, et al., 2010	39	30.96	2.58	33	31.49	2.81	baseline	0.00	-0.20	[-0.66; 0.27]
Parker, PA, et al., 2009 I.	39	51.32	6.74	36	52.29	5.88	baseline	0.00	-0.15	[-0.60; 0.30]
Ferguson, RJ, et al., 2012	19	7.68	1.95	21	7.79	1.27	baseline	0.00	-0.07	[-0.69; 0.55]
Zhang, LMM, et al., 2021	80	58.64	16.46	80	59.25	16.46	baseline	0.00	-0.04	[-0.35; 0.27]
Wu, Q, et al., 2021	43	52.17	5.09	43	51.95	5.12	baseline	0.00	0.04	[-0.38; 0.47]
Takano, T, et al., 2021	31	84.80	15.98	38	84.00	17.01	baseline	0.00	0.05	[-0.43; 0.52]
Fillion, L, et al. 2008	44	40.49	9.50	43	40.00	9.43	baseline	0.00	0.05	[-0.37; 0.47]
Yun et al., 2017	134	78.60	13.50	72	77.90	11.10	baseline	0.00	0.05	[-0.23; 0.34]
Parker, PA, et al., 2009 II.	39	52.79	6.12	36	52.29	5.88	baseline	0.00	0.08	[-0.37; 0.54]
Cengiz, HO, et al., 2023	32	14.15	7.56	33	13.03	6.82	baseline	0.00	0.15	[-0.33; 0.64]
Liu, T, et al., 2019	49	76.27	18.94	53	72.14	15.11	baseline	0.00	0.24	[-0.15; 0.63]
Zhao, X, et al., 2021	52	58.01	2.12	51	57.03	4.32	baseline	0.00	0.29	[-0.10; 0.68]
Ferguson, RJ, et al., 2012	19	7.04	1.79	21	7.91	1.10	followup	8.00	-0.58	[-1.22; 0.05]
Rodrigez, B, et al., 2014	8	62.50	12.82	7	71.43	36.25	followup	0.10	-0.32	[-1.34; 0.70]
Beatty, L. et al. 2015	30	78.11	3.66	30	79.16	3.63	followup	6.00	-0.28	[-0.79; 0.22]
Takano, T, et al., 2021	31	75.30	9.85	38	76.40	24.16	followup	24.00	-0.06	[-0.53; 0.42]
Zhang, LMM, et al., 2021	80	62.34	16.46	80	62.75	15.02	followup	12.00	-0.03	[-0.34; 0.28]
Parker, PA, et al., 2009 I.	38	48.86	8.88	32	48.51	9.50	followup	24.00	0.04	[-0.43; 0.51]
Takano, T, et al., 2021	31	83.40	19.82	38	81.60	18.00	followup	12.00	0.09	[-0.38; 0.57]
Fillion, L, et al. 2008	44	46.76	9.24	43	44.64	11.05	followup	12.00	0.21	[-0.21; 0.63]
Zhang, LMM, et al., 2021	80	67.90	15.85	80	64.81	13.17	followup	24.00	0.21	[-0.10; 0.52]
Zhang, LMM, et al., 2021	80	72.01	14.81	80	68.72	12.35	followup	36.00	0.24	[-0.07; 0.55]
Zhang, LMM, et al., 2021	80	74.27	15.02	80	70.16	13.58	followup	48.00	0.29	[-0.03; 0.60]
Parker, PA, et al., 2009 I.	39	47.28	9.18	36	44.63	9.12	followup	6.00	0.29	[-0.17; 0.74]
Parker, PA, et al., 2009 II.	38	51.36	9.18	32	48.51	9.50	followup	24.00	0.30	[-0.17; 0.78]
Fillion, L, et al. 2008	44	45.10	10.42	43	41.76	9.76	followup	4.00	0.33	[-0.10; 0.75]
Yun et al., 2017	134	82.90	13.10	72	78.20	12.40	followup	48.00	0.36	[0.08; 0.65]
Ruiz-Vozmediano, J, et al.,2020	31	85.00	14.10	32	78.30	18.90	followup	24.00	0.40	[-0.10; 0.89]
Liu, T, et al., 2019	49	74.61	18.94	53	67.34	11.65	followup	9.00	0.46	[0.07; 0.86]
Parker, PA, et al., 2009 II.	39	49.58	9.74	36	44.63	9.12	followup	6.00	0.52	[0.06; 0.98]
Parker, PA, et al., 2009 I.	37	50.22	8.46	32	45.12	8.88	followup	48.00	0.58	[0.10; 1.07]
Liu, T, et al., 2019	49	85.72	12.30	53	77.09	16.10	followup	12.00	0.59	[0.20; 0.99]
Cengiz, HO, et al., 2023	32	16.75	6.33	33	12.33	7.00	followup	8.00	0.65	[0.15; 1.15]
Parker, PA, et al., 2009 II.	37	51.76	10.16	32	45.12	8.88	followup	48.00	0.68	[0.20; 1.17]
Wu, Q, et al., 2021	43	80.45	8.21	43	71.45	7.46	followup	12.00	1.14	[0.68; 1.59]
Willems, R, et al., 2016	188	83.70	2.60	221	80.10	2.20	followup	24.00	1.50	[1.28; 1.72]
Zhao, X, et al., 2021	52	65.12	6.01	51	57.51	3.24	followup	12.00	1.56	[1.12; 2.00]
Guan, S, et al., 2019	50	69.11	8.27	50	55.93	5.80	followup	8.00	1.83	[1.36; 2.30]
Beatty, L. et al. 2015	30	90.41	3.39	30	83.67	3.36	followup	26.07	1.97	[1.35; 2.59]
Beatty, L. et al. 2015	30	85.63	3.50	30	75.66	3.47	followup	13.03	2.82	[2.10; 3.55]
Rodríguez Vega, B, et al., 2010	39	56.60	2.58	33	41.79	2.81	followup	12.00	5.45	[4.43; 6.48]
Rodríguez Vega, B, et al., 2010	39	66.52	2.58	33	39.77	2.81	followup	24.00	9.85	[8.13; 11.56]
Random effect	2349			2273					0.81	[0.17; 1.45]
Prediction interval										[-2.06; 3.68]
**advanced**										
Lu, Z, et al., 201	203	81.30	3.50	103	83.60	3.90	baseline	0.00	-0.63	[-0.87; -0.39]
Serfaty M, et al., 2018	20	12.60	4.80	22	14.70	6.40	baseline	0.00	-0.36	[-0.97; 0.25]
Heiney, SP, et al., 2003	33	6.60	1.90	33	7.10	1.70	baseline	0.00	-0.27	[-0.76; 0.21]
Walczak, A, et al.,2017	61	16.39	5.50	49	16.96	6.24	baseline	0.00	-0.10	[-0.47; 0.28]
Penedo, FJ, et al., 2020 I.	95	-23.26	3.80	97	-23.09	3.84	baseline	0.00	-0.04	[-0.33; 0.24]
Boele FW, et al ., 2017 II.	35	46.88	11.10	28	46.82	9.50	baseline	0.00	0.01	[-0.49; 0.50]
Penedo, FJ, et al., 2020 II.	95	-20.27	4.68	97	-20.48	4.63	baseline	0.00	0.04	[-0.24; 0.33]
Penedo, FJ, et al., 2007	41	-23.98	28.88	30	-25.67	14.84	baseline	0.00	0.07	[-0.40; 0.54]
Schofield, P, et al., 2013	55	67.08	25.66	53	64.68	25.92	baseline	0.00	0.09	[-0.29; 0.47]
Boele FW, et al ., 2017 I.	45	47.63	10.90	44	45.55	9.10	baseline	0.00	0.21	[-0.21; 0.62]
Huri M, et al., 2015	19	36.68	8.73	15	-36.26	7.93	baseline	0.00	8.49	[6.25; 10.73]
Serfaty M, et al., 2018	20	12.20	8.00	22	17.50	4.50	followup	12.00	-0.81	[-1.44; -0.18]
Heiney, SP, et al., 2003	33	6.40	1.40	33	7.20	2.10	followup	16.00	-0.44	[-0.93; 0.05]
Heiney, SP, et al., 2003	33	6.40	1.80	33	6.90	2.10	followup	6.00	-0.25	[-0.74; 0.23]
Schofield, P, et al., 2013	55	57.21	24.88	53	60.20	25.40	followup	8.00	-0.12	[-0.50; 0.26]
Penedo, FJ, et al., 2007	41	-25.51	13.16	30	-23.83	21.36	followup	12.00	-0.10	[-0.57; 0.37]
Penedo, FJ, et al., 2020 I.	95	-23.29	4.00	97	-23.11	4.04	followup	48.00	-0.04	[-0.33; 0.24]
Penedo, FJ, et al., 2020 I.	95	-23.16	3.90	97	-22.99	3.94	followup	24.00	-0.04	[-0.33; 0.24]
Serfaty M, et al., 2018	20	15.70	7.30	22	15.50	4.90	followup	18.00	0.03	[-0.57; 0.64]
Penedo, FJ, et al., 2020 II.	95	-20.08	4.87	97	-20.29	4.83	followup	48.00	0.04	[-0.24; 0.33]
Penedo, FJ, et al., 2020 II.	95	-20.49	4.78	97	-20.70	4.73	followup	24.00	0.04	[-0.24; 0.33]
Boele FW, et al ., 2017 II.	35	48.16	9.90	28	47.43	11.50	followup	12.00	0.07	[-0.43; 0.56]
Boele FW, et al ., 2017 I.	45	48.57	9.80	44	47.25	10.70	followup	12.00	0.13	[-0.29; 0.54]
Serfaty M, et al., 2018	20	16.30	8.80	22	14.60	5.60	followup	24.00	0.23	[-0.38; 0.84]
Boele FW, et al ., 2017 II.	35	51.07	11.70	28	48.47	10.50	followup	6.00	0.23	[-0.27; 0.73]
Walczak, A, et al.,2017	61	18.44	6.04	49	16.76	5.50	followup	4.00	0.29	[-0.09; 0.67]
Boele FW, et al ., 2017 I.	45	51.17	11.40	44	46.95	10.10	followup	6.00	0.39	[-0.03; 0.81]
Lu, Z, et al., 201	203	82.60	4.50	103	77.60	6.30	followup	9.00	0.96	[0.71; 1.21]
Huri M, et al., 2015	19	0.57	5.38	15	-41.73	6.14	followup	12.00	7.21	[5.27; 9.15]
Random effect	1747			1485					0.78	[-0.44; 2.00]
Prediction interval										[-2.40; 3.95]
**survivor**										
Chen, et al., 2017	58	61.20	7.50	65	63.10	7.30	baseline	0.00	-0.26	[-0.61; 0.10]
Qiu, H, et al., 2018 II.	98	7.72	5.28	196	8.58	5.92	baseline	0.00	-0.15	[-0.39; 0.09]
Peng, L, et al., 2022	28	78.33	12.78	29	79.08	10.04	baseline	0.00	-0.06	[-0.58; 0.45]
Jelvehzadeh, F, et al., 2022	24	3.15	1.52	24	3.21	2.08	baseline	0.00	-0.03	[-0.60; 0.53]
Qiu, H, et al., 2018 I.	98	8.71	5.89	196	8.58	5.92	baseline	0.00	0.02	[-0.22; 0.26]
McCusker, J, et al., 2021	121	41.60	9.80	124	40.70	9.20	baseline	0.00	0.09	[-0.16; 0.35]
Von Ah, D, et al., 2012 II.	27	44.69	4.40	29	43.18	4.72	baseline	0.00	0.33	[-0.20; 0.85]
Von Ah, D, et al., 2012 I.	26	45.71	5.98	29	43.18	4.72	baseline	0.00	0.47	[-0.07; 1.00]
Peng, L, et al., 2022	28	78.33	12.52	29	78.16	10.60	followup	0.10	0.01	[-0.50; 0.53]
Von Ah, D, et al., 2012 I.	26	45.03	6.17	29	44.49	4.81	followup	8.00	0.10	[-0.43; 0.63]
Von Ah, D, et al., 2012 II.	27	45.00	4.48	29	44.49	4.81	followup	8.00	0.11	[-0.42; 0.63]
Peng, L, et al., 2022	28	83.10	12.63	28	81.38	14.52	followup	4.00	0.12	[-0.40; 0.65]
Chen, et al., 2017	58	48.40	9.10	65	46.80	8.50	followup	24.00	0.18	[-0.17; 0.54]
Chen, et al., 2017	58	48.60	7.40	65	47.10	7.70	followup	8.00	0.20	[-0.16; 0.55]
Qiu, H, et al., 2018 II.	98	9.83	9.73	196	8.01	8.85	followup	4.00	0.20	[-0.04; 0.44]
Qiu, H, et al., 2018 II.	98	14.04	11.82	196	11.52	12.78	followup	24.00	0.20	[-0.04; 0.45]
Qiu, H, et al., 2018 II.	98	12.99	11.82	196	10.18	11.80	followup	12.00	0.24	[-0.01; 0.48]
McCusker, J, et al., 2021	121	43.90	9.80	124	41.40	10.10	followup	24.00	0.25	[-0.00; 0.50]
Qiu, H, et al., 2018 I.	98	12.92	9.73	196	8.01	8.85	followup	4.00	0.54	[0.29; 0.78]
Qiu, H, et al., 2018 I.	98	16.92	11.12	196	10.18	11.80	followup	12.00	0.58	[0.33; 0.83]
Qiu, H, et al., 2018 I.	98	18.96	10.43	196	11.52	12.78	followup	24.00	0.62	[0.37; 0.86]
Jelvehzadeh, F, et al., 2022	24	5.64	1.73	24	3.70	1.68	followup	12.00	1.12	[0.51; 1.73]
Jelvehzadeh, F, et al., 2022	24	5.64	1.73	24	3.43	1.75	followup	8.00	1.25	[0.63; 1.87]
Random effect	1462			2285					0.46	[0.15; 0.76]
Prediction interval										[-2.68; 3.59]
**Random effect**	**5558**			**6043**					**0.71**	**[0.20; 1.23]**
**Prediction interval**										**[-1.95; 3.38]**

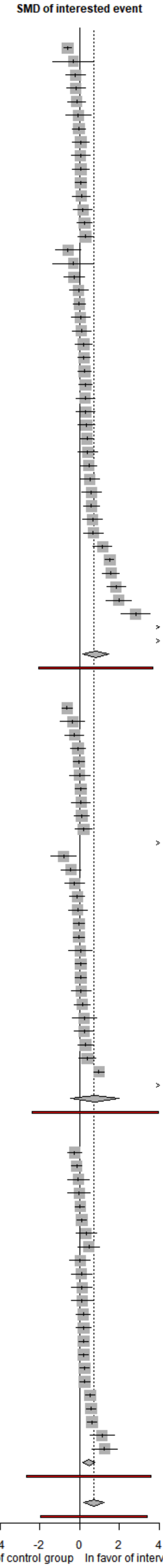

Figure S21.2. Forest plot represents the difference between the intervention vs. control group in the Physical QoL domain with the cancer stage subgroups as predicted at week 12 (post-intervention). SMD - Standardized mean difference, CI - confidence interval.

Figure S21.3.T24

Study	Experimental Patient N	Experimental Mean	Experimental SD	Control Patient N	Control Mean	Control SD	follow-up	Follow-up time	SMD	95%-CI
early										
Willems, R, et al., 2016	188	85.08	2.10	221	86.70	3.00	baseline	0.00	-0.62	[-0.82; -0.42]
Rodrigez, B, et al., 2014	8	62.50	12.82	7	71.43	36.25	baseline	0.00	-0.32	[-1.34; 0.70]
Ruiz-Vozmediano, J, et al.,2020	31	75.40	19.50	32	79.20	15.70	baseline	0.00	-0.21	[-0.71; 0.28]
Rodríguez Vega, B, et al., 2010	39	30.96	2.58	33	31.49	2.81	baseline	0.00	-0.20	[-0.66; 0.27]
Parker, PA, et al., 2009 I.	39	51.32	6.74	36	52.29	5.88	baseline	0.00	-0.15	[-0.60; 0.30]
Ferguson, RJ, et al., 2012	19	7.68	1.95	21	7.79	1.27	baseline	0.00	-0.07	[-0.69; 0.55]
Zhang, LMM, et al., 2021	80	58.64	16.46	80	59.25	16.46	baseline	0.00	-0.04	[-0.35; 0.27]
Wu, Q, et al., 2021	43	52.17	5.09	43	51.95	5.12	baseline	0.00	0.04	[-0.38; 0.47]
Takano, T, et al., 2021	31	84.80	15.98	38	84.00	17.01	baseline	0.00	0.05	[-0.43; 0.52]
Fillion, L, et al. 2008	44	40.49	9.50	43	40.00	9.43	baseline	0.00	0.05	[-0.37; 0.47]
Yun et al., 2017	134	78.60	13.50	72	77.90	11.10	baseline	0.00	0.05	[-0.23; 0.34]
Parker, PA, et al., 2009 II.	39	52.79	6.12	36	52.29	5.88	baseline	0.00	0.08	[-0.37; 0.54]
Cengiz, HO, et al., 2023	32	14.15	7.56	33	13.03	6.82	baseline	0.00	0.15	[-0.33; 0.64]
Liu, T, et al., 2019	49	76.27	18.94	53	72.14	15.11	baseline	0.00	0.24	[-0.15; 0.63]
Zhao, X, et al., 2021	52	58.01	2.12	51	57.03	4.32	baseline	0.00	0.29	[-0.10; 0.68]
Ferguson, RJ, et al., 2012	19	7.04	1.79	21	7.91	1.10	followup	8.00	-0.58	[-1.22; 0.05]
Rodrigez, B, et al., 2014	8	62.50	12.82	7	71.43	36.25	followup	0.10	-0.32	[-1.34; 0.70]
Beatty, L. et al. 2015	30	78.11	3.66	30	79.16	3.63	followup	6.00	-0.28	[-0.79; 0.22]
Takano, T, et al., 2021	31	75.30	9.85	38	76.40	24.16	followup	24.00	-0.06	[-0.53; 0.42]
Zhang, LMM, et al., 2021	80	62.34	16.46	80	62.75	15.02	followup	12.00	-0.03	[-0.34; 0.28]
Parker, PA, et al., 2009 I.	38	48.86	8.88	32	48.51	9.50	followup	24.00	0.04	[-0.43; 0.51]
Takano, T, et al., 2021	31	83.40	19.82	38	81.60	18.00	followup	12.00	0.09	[-0.38; 0.57]
Fillion, L, et al. 2008	44	46.76	9.24	43	44.64	11.05	followup	12.00	0.21	[-0.21; 0.63]
Zhang, LMM, et al., 2021	80	67.90	15.85	80	64.81	13.17	followup	24.00	0.21	[-0.10; 0.52]
Zhang, LMM, et al., 2021	80	72.01	14.81	80	68.72	12.35	followup	36.00	0.24	[-0.07; 0.55]
Zhang, LMM, et al., 2021	80	74.27	15.02	80	70.16	13.58	followup	48.00	0.29	[-0.03; 0.60]
Parker, PA, et al., 2009 I.	39	47.28	9.18	36	44.63	9.12	followup	6.00	0.29	[-0.17; 0.74]
Parker, PA, et al., 2009 II.	38	51.36	9.18	32	48.51	9.50	followup	24.00	0.30	[-0.17; 0.78]
Fillion, L, et al. 2008	44	45.10	10.42	43	41.76	9.76	followup	4.00	0.33	[-0.10; 0.75]
Yun et al., 2017	134	82.90	13.10	72	78.20	12.40	followup	48.00	0.36	[0.08; 0.65]
Ruiz-Vozmediano, J, et al.,2020	31	85.00	14.10	32	78.30	18.90	followup	24.00	0.40	[-0.10; 0.89]
Liu, T, et al., 2019	49	74.61	18.94	53	67.34	11.65	followup	9.00	0.46	[0.07; 0.86]
Parker, PA, et al., 2009 II.	39	49.58	9.74	36	44.63	9.12	followup	6.00	0.52	[0.06; 0.98]
Parker, PA, et al., 2009 I.	37	50.22	8.46	32	45.12	8.88	followup	48.00	0.58	[0.10; 1.07]
Liu, T, et al., 2019	49	85.72	12.30	53	77.09	16.10	followup	12.00	0.59	[0.20; 0.99]
Cengiz, HO, et al., 2023	32	16.75	6.33	33	12.33	7.00	followup	8.00	0.65	[0.15; 1.15]
Parker, PA, et al., 2009 II.	37	51.76	10.16	32	45.12	8.88	followup	48.00	0.68	[0.20; 1.17]
Wu, Q, et al., 2021	43	80.45	8.21	43	71.45	7.46	followup	12.00	1.14	[0.68; 1.59]
Willems, R, et al., 2016	188	83.70	2.60	221	80.10	2.20	followup	24.00	1.50	[1.28; 1.72]
Zhao, X, et al., 2021	52	65.12	6.01	51	57.51	3.24	followup	12.00	1.56	[1.12; 2.00]
Guan, S, et al., 2019	50	69.11	8.27	50	55.93	5.80	followup	8.00	1.83	[1.36; 2.30]
Beatty, L. et al. 2015	30	90.41	3.39	30	83.67	3.36	followup	26.07	1.97	[1.35; 2.59]
Beatty, L. et al. 2015	30	85.63	3.50	30	75.66	3.47	followup	13.03	2.82	[2.10; 3.55]
Rodríguez Vega, B, et al., 2010	39	56.60	2.58	33	41.79	2.81	followup	12.00	5.45	[4.43; 6.48]
Rodríguez Vega, B, et al., 2010	39	66.52	2.58	33	39.77	2.81	followup	24.00	9.85	[8.13; 11.56]
Random effect	2349			2273					1.05	[0.20; 1.90]
Prediction interval										[-1.86; 3.96]
advanced										
Lu, Z, et al., 201	203	81.30	3.50	103	83.60	3.90	baseline	0.00	-0.63	[-0.87; -0.39]
Serfaty M, et al., 2018	20	12.60	4.80	22	14.70	6.40	baseline	0.00	-0.36	[-0.97; 0.25]
Heiney, SP, et al., 2003	33	6.60	1.90	33	7.10	1.70	baseline	0.00	-0.27	[-0.76; 0.21]
Walczak, A, et al.,2017	61	16.39	5.50	49	16.96	6.24	baseline	0.00	-0.10	[-0.47; 0.28]
Penedo, FJ, et al., 2020 I.	95	-23.26	3.80	97	-23.09	3.84	baseline	0.00	-0.04	[-0.33; 0.24]
Boele FW, et al ., 2017 II.	35	46.88	11.10	28	46.82	9.50	baseline	0.00	0.01	[-0.49; 0.50]
Penedo, FJ, et al., 2020 II.	95	-20.27	4.68	97	-20.48	4.63	baseline	0.00	0.04	[-0.24; 0.33]
Penedo, FJ, et al., 2007	41	-23.98	28.88	30	-25.67	14.84	baseline	0.00	0.07	[-0.40; 0.54]
Schofield, P, et al., 2013	55	67.08	25.66	53	64.68	25.92	baseline	0.00	0.09	[-0.29; 0.47]
Boele FW, et al ., 2017 I.	45	47.63	10.90	44	45.55	9.10	baseline	0.00	0.21	[-0.21; 0.62]
Huri M, et al., 2015	19	36.68	8.73	15	-36.26	7.93	baseline	0.00	8.49	[6.25; 10.73]
Serfaty M, et al., 2018	20	12.20	8.00	22	17.50	4.50	followup	12.00	-0.81	[-1.44; -0.18]
Heiney, SP, et al., 2003	33	6.40	1.40	33	7.20	2.10	followup	16.00	-0.44	[-0.93; 0.05]
Heiney, SP, et al., 2003	33	6.40	1.80	33	6.90	2.10	followup	6.00	-0.25	[-0.74; 0.23]
Schofield, P, et al., 2013	55	57.21	24.88	53	60.20	25.40	followup	8.00	-0.12	[-0.50; 0.26]
Penedo, FJ, et al., 2007	41	-25.51	13.16	30	-23.83	21.36	followup	12.00	-0.10	[-0.57; 0.37]
Penedo, FJ, et al., 2020 I.	95	-23.29	4.00	97	-23.11	4.04	followup	48.00	-0.04	[-0.33; 0.24]
Penedo, FJ, et al., 2020 I.	95	-23.16	3.90	97	-22.99	3.94	followup	24.00	-0.04	[-0.33; 0.24]
Serfaty M, et al., 2018	20	15.70	7.30	22	15.50	4.90	followup	18.00	0.03	[-0.57; 0.64]
Penedo, FJ, et al., 2020 II.	95	-20.08	4.87	97	-20.29	4.83	followup	48.00	0.04	[-0.24; 0.33]
Penedo, FJ, et al., 2020 II.	95	-20.49	4.78	97	-20.70	4.73	followup	24.00	0.04	[-0.24; 0.33]
Boele FW, et al ., 2017 II.	35	48.16	9.90	28	47.43	11.50	followup	12.00	0.07	[-0.43; 0.56]
Boele FW, et al ., 2017 I.	45	48.57	9.80	44	47.25	10.70	followup	12.00	0.13	[-0.29; 0.54]
Serfaty M, et al., 2018	20	16.30	8.80	22	14.60	5.60	followup	24.00	0.23	[-0.38; 0.84]
Boele FW, et al ., 2017 II.	35	51.07	11.70	28	48.47	10.50	followup	6.00	0.23	[-0.27; 0.73]
Walczak, A, et al.,2017	61	18.44	6.04	49	16.76	5.50	followup	4.00	0.29	[-0.09; 0.67]
Boele FW, et al ., 2017 I.	45	51.17	11.40	44	46.95	10.10	followup	6.00	0.39	[-0.03; 0.81]
Lu, Z, et al., 201	203	82.60	4.50	103	77.60	6.30	followup	9.00	0.96	[0.71; 1.21]
Huri M, et al., 2015	19	0.57	5.38	15	-41.73	6.14	followup	12.00	7.21	[5.27; 9.15]
Random effect	1747			1485					1.02	[-0.23; 2.27]
Prediction interval										[-2.13; 4.17]
survivor										
Chen, et al., 2017	58	61.20	7.50	65	63.10	7.30	baseline	0.00	-0.26	[-0.61; 0.10]
Qiu, H, et al., 2018 II.	98	7.72	5.28	196	8.58	5.92	baseline	0.00	-0.15	[-0.39; 0.09]
Peng, L, et al., 2022	28	78.33	12.78	29	79.08	10.04	baseline	0.00	-0.06	[-0.58; 0.45]
Jelvehzadeh, F, et al., 2022	24	3.15	1.52	24	3.21	2.08	baseline	0.00	-0.03	[-0.60; 0.53]
Qiu, H, et al., 2018 I.	98	8.71	5.89	196	8.58	5.92	baseline	0.00	0.02	[-0.22; 0.26]
McCusker, J, et al., 2021	121	41.60	9.80	124	40.70	9.20	baseline	0.00	0.09	[-0.16; 0.35]
Von Ah, D, et al., 2012 II.	27	44.69	4.40	29	43.18	4.72	baseline	0.00	0.33	[-0.20; 0.85]
Von Ah, D, et al., 2012 I.	26	45.71	5.98	29	43.18	4.72	baseline	0.00	0.47	[-0.07; 1.00]
Peng, L, et al., 2022	28	78.33	12.52	29	78.16	10.60	followup	0.10	0.01	[-0.50; 0.53]
Von Ah, D, et al., 2012 I.	26	45.03	6.17	29	44.49	4.81	followup	8.00	0.10	[-0.43; 0.63]
Von Ah, D, et al., 2012 II.	27	45.00	4.48	29	44.49	4.81	followup	8.00	0.11	[-0.42; 0.63]
Peng, L, et al., 2022	28	83.10	12.63	28	81.38	14.52	followup	4.00	0.12	[-0.40; 0.65]
Chen, et al., 2017	58	48.40	9.10	65	46.80	8.50	followup	24.00	0.18	[-0.17; 0.54]
Chen, et al., 2017	58	48.60	7.40	65	47.10	7.70	followup	8.00	0.20	[-0.16; 0.55]
Qiu, H, et al., 2018 II.	98	9.83	9.73	196	8.01	8.85	followup	4.00	0.20	[-0.04; 0.44]
Qiu, H, et al., 2018 II.	98	14.04	11.82	196	11.52	12.78	followup	24.00	0.20	[-0.04; 0.45]
Qiu, H, et al., 2018 II.	98	12.99	11.82	196	10.18	11.80	followup	12.00	0.24	[-0.01; 0.48]
McCusker, J, et al., 2021	121	43.90	9.80	124	41.40	10.10	followup	24.00	0.25	[-0.00; 0.50]
Qiu, H, et al., 2018 I.	98	12.92	9.73	196	8.01	8.85	followup	4.00	0.54	[0.29; 0.78]
Qiu, H, et al., 2018 I.	98	16.92	11.12	196	10.18	11.80	followup	12.00	0.58	[0.33; 0.83]
Qiu, H, et al., 2018 I.	98	18.96	10.43	196	11.52	12.78	followup	24.00	0.62	[0.37; 0.86]
Jelvehzadeh, F, et al., 2022	24	5.64	1.73	24	3.70	1.68	followup	12.00	1.12	[0.51; 1.73]
Jelvehzadeh, F, et al., 2022	24	5.64	1.73	24	3.43	1.75	followup	8.00	1.25	[0.63; 1.87]
Random effect	1462			2285					0.70	[0.18; 1.23]
Prediction interval										[-2.39; 3.80]
**Random effect**	**5558**			**6043**					**0.96**	**[0.27; 1.65]**
**Prediction interval**										**[-1.76; 3.68]**

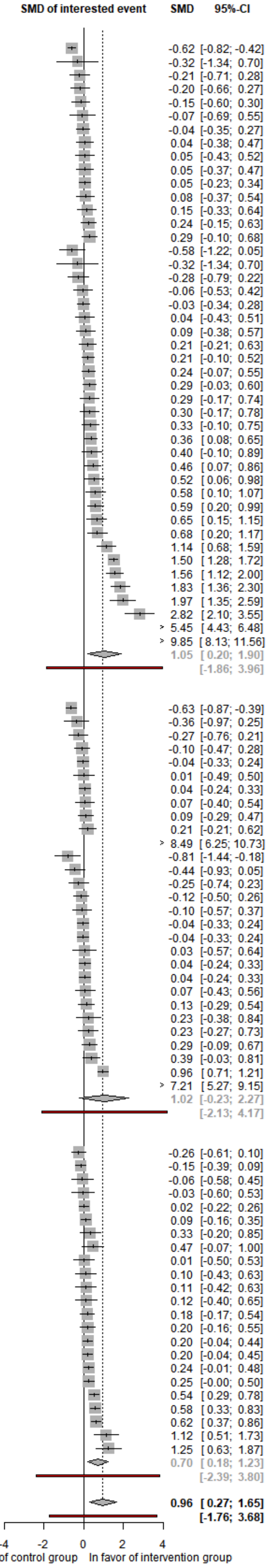

Figure S21.3. Forest plot represents the difference between the intervention vs. control group in the Physical QoL domain with the cancer stage subgroups as predicted at week 24 (post-intervention). SMD - Standardized mean difference, CI - confidence interval.

Figure S21.4.T48

Study	Experimental Patient N	Experimental Mean	Experimental SD	Control Patient N	Control Mean	Control SD	follow-up	Follow-up time	SMD	95%-CI
**early**										
Willems, R, et al., 2016	188	85.08	2.10	221	86.70	3.00	baseline	0.00	-0.62	[-0.82; -0.42]
Rodrigez, B, et al., 2014	8	62.50	12.82	7	71.43	36.25	baseline	0.00	-0.32	[-1.34; 0.70]
Ruiz-Vozmediano, J, et al.,2020	31	75.40	19.50	32	79.20	15.70	baseline	0.00	-0.21	[-0.71; 0.28]
Rodríguez Vega, B, et al., 2010	39	30.96	2.58	33	31.49	2.81	baseline	0.00	-0.20	[-0.66; 0.27]
Parker, PA, et al., 2009 I.	39	51.32	6.74	36	52.29	5.88	baseline	0.00	-0.15	[-0.60; 0.30]
Ferguson, RJ, et al., 2012	19	7.68	1.95	21	7.79	1.27	baseline	0.00	-0.07	[-0.69; 0.55]
Zhang, LMM, et al., 2021	80	58.64	16.46	80	59.25	16.46	baseline	0.00	-0.04	[-0.35; 0.27]
Wu, Q, et al., 2021	43	52.17	5.09	43	51.95	5.12	baseline	0.00	0.04	[-0.38; 0.47]
Takano, T, et al., 2021	31	84.80	15.98	38	84.00	17.01	baseline	0.00	0.05	[-0.43; 0.52]
Fillion, L, et al. 2008	44	40.49	9.50	43	40.00	9.43	baseline	0.00	0.05	[-0.37; 0.47]
Yun et al., 2017	134	78.60	13.50	72	77.90	11.10	baseline	0.00	0.05	[-0.23; 0.34]
Parker, PA, et al., 2009 II.	39	52.79	6.12	36	52.29	5.88	baseline	0.00	0.08	[-0.37; 0.54]
Cengiz, HO, et al., 2023	32	14.15	7.56	33	13.03	6.82	baseline	0.00	0.15	[-0.33; 0.64]
Liu, T, et al., 2019	49	76.27	18.94	53	72.14	15.11	baseline	0.00	0.24	[-0.15; 0.63]
Zhao, X, et al., 2021	52	58.01	2.12	51	57.03	4.32	baseline	0.00	0.29	[-0.10; 0.68]
Ferguson, RJ, et al., 2012	19	7.04	1.79	21	7.91	1.10	followup	8.00	-0.58	[-1.22; 0.05]
Rodrigez, B, et al., 2014	8	62.50	12.82	7	71.43	36.25	followup	0.10	-0.32	[-1.34; 0.70]
Beatty, L. et al. 2015	30	78.11	3.66	30	79.16	3.63	followup	6.00	-0.28	[-0.79; 0.22]
Takano, T, et al., 2021	31	75.30	9.85	38	76.40	24.16	followup	24.00	-0.06	[-0.53; 0.42]
Zhang, LMM, et al., 2021	80	62.34	16.46	80	62.75	15.02	followup	12.00	-0.03	[-0.34; 0.28]
Parker, PA, et al., 2009 I.	38	48.86	8.88	32	48.51	9.50	followup	24.00	0.04	[-0.43; 0.51]
Takano, T, et al., 2021	31	83.40	19.82	38	81.60	18.00	followup	12.00	0.09	[-0.38; 0.57]
Fillion, L, et al. 2008	44	46.76	9.24	43	44.64	11.05	followup	12.00	0.21	[-0.21; 0.63]
Zhang, LMM, et al., 2021	80	67.90	15.85	80	64.81	13.17	followup	24.00	0.21	[-0.10; 0.52]
Zhang, LMM, et al., 2021	80	72.01	14.81	80	68.72	12.35	followup	36.00	0.24	[-0.07; 0.55]
Zhang, LMM, et al., 2021	80	74.27	15.02	80	70.16	13.58	followup	48.00	0.29	[-0.03; 0.60]
Parker, PA, et al., 2009 I.	39	47.28	9.18	36	44.63	9.12	followup	6.00	0.29	[-0.17; 0.74]
Parker, PA, et al., 2009 II.	38	51.36	9.18	32	48.51	9.50	followup	24.00	0.30	[-0.17; 0.78]
Fillion, L, et al. 2008	44	45.10	10.42	43	41.76	9.76	followup	4.00	0.33	[-0.10; 0.75]
Yun et al., 2017	134	82.90	13.10	72	78.20	12.40	followup	48.00	0.36	[0.08; 0.65]
Ruiz-Vozmediano, J, et al.,2020	31	85.00	14.10	32	78.30	18.90	followup	24.00	0.40	[-0.10; 0.89]
Liu, T, et al., 2019	49	74.61	18.94	53	67.34	11.65	followup	9.00	0.46	[0.07; 0.86]
Parker, PA, et al., 2009 II.	39	49.58	9.74	36	44.63	9.12	followup	6.00	0.52	[0.06; 0.98]
Parker, PA, et al., 2009 I.	37	50.22	8.46	32	45.12	8.88	followup	48.00	0.58	[0.10; 1.07]
Liu, T, et al., 2019	49	85.72	12.30	53	77.09	16.10	followup	12.00	0.59	[0.20; 0.99]
Cengiz, HO, et al., 2023	32	16.75	6.33	33	12.33	7.00	followup	8.00	0.65	[0.15; 1.15]
Parker, PA, et al., 2009 II.	37	51.76	10.16	32	45.12	8.88	followup	48.00	0.68	[0.20; 1.17]
Wu, Q, et al., 2021	43	80.45	8.21	43	71.45	7.46	followup	12.00	1.14	[0.68; 1.59]
Willems, R, et al., 2016	188	83.70	2.60	221	80.10	2.20	followup	24.00	1.50	[1.28; 1.72]
Zhao, X, et al., 2021	52	65.12	6.01	51	57.51	3.24	followup	12.00	1.56	[1.12; 2.00]
Guan, S, et al., 2019	50	69.11	8.27	50	55.93	5.80	followup	8.00	1.83	[1.36; 2.30]
Beatty, L. et al. 2015	30	90.41	3.39	30	83.67	3.36	followup	26.07	1.97	[1.35; 2.59]
Beatty, L. et al. 2015	30	85.63	3.50	30	75.66	3.47	followup	13.03	2.82	[2.10; 3.55]
Rodríguez Vega, B, et al., 2010	39	56.60	2.58	33	41.79	2.81	followup	12.00	5.45	[4.43; 6.48]
Rodríguez Vega, B, et al., 2010	39	66.52	2.58	33	39.77	2.81	followup	24.00	9.85	[8.13; 11.56]
Random effect	2349			2273					0.75	[0.06; 1.44]
Prediction interval										[-2.44; 3.95]
**advanced**										
Lu, Z, et al., 201	203	81.30	3.50	103	83.60	3.90	baseline	0.00	-0.63	[-0.87; -0.39]
Serfaty M, et al., 2018	20	12.60	4.80	22	14.70	6.40	baseline	0.00	-0.36	[-0.97; 0.25]
Heiney, SP, et al., 2003	33	6.60	1.90	33	7.10	1.70	baseline	0.00	-0.27	[-0.76; 0.21]
Walczak, A, et al.,2017	61	16.39	5.50	49	16.96	6.24	baseline	0.00	-0.10	[-0.47; 0.28]
Penedo, FJ, et al., 2020 I.	95	-23.26	3.80	97	-23.09	3.84	baseline	0.00	-0.04	[-0.33; 0.24]
Boele FW, et al ., 2017 II.	35	46.88	11.10	28	46.82	9.50	baseline	0.00	0.01	[-0.49; 0.50]
Penedo, FJ, et al., 2020 II.	95	-20.27	4.68	97	-20.48	4.63	baseline	0.00	0.04	[-0.24; 0.33]
Penedo, FJ, et al., 2007	41	-23.98	28.88	30	-25.67	14.84	baseline	0.00	0.07	[-0.40; 0.54]
Schofield, P, et al., 2013	55	67.08	25.66	53	64.68	25.92	baseline	0.00	0.09	[-0.29; 0.47]
Boele FW, et al ., 2017 I.	45	47.63	10.90	44	45.55	9.10	baseline	0.00	0.21	[-0.21; 0.62]
Huri M, et al., 2015	19	36.68	8.73	15	-36.26	7.93	baseline	0.00	8.49	[6.25; 10.73]
Serfaty M, et al., 2018	20	12.20	8.00	22	17.50	4.50	followup	12.00	-0.81	[-1.44; -0.18]
Heiney, SP, et al., 2003	33	6.40	1.40	33	7.20	2.10	followup	16.00	-0.44	[-0.93; 0.05]
Heiney, SP, et al., 2003	33	6.40	1.80	33	6.90	2.10	followup	6.00	-0.25	[-0.74; 0.23]
Schofield, P, et al., 2013	55	57.21	24.88	53	60.20	25.40	followup	8.00	-0.12	[-0.50; 0.26]
Penedo, FJ, et al., 2007	41	-25.51	13.16	30	-23.83	21.36	followup	12.00	-0.10	[-0.57; 0.37]
Penedo, FJ, et al., 2020 I.	95	-23.29	4.00	97	-23.11	4.04	followup	48.00	-0.04	[-0.33; 0.24]
Penedo, FJ, et al., 2020 I.	95	-23.16	3.90	97	-22.99	3.94	followup	24.00	-0.04	[-0.33; 0.24]
Serfaty M, et al., 2018	20	15.70	7.30	22	15.50	4.90	followup	18.00	0.03	[-0.57; 0.64]
Penedo, FJ, et al., 2020 II.	95	-20.08	4.87	97	-20.29	4.83	followup	48.00	0.04	[-0.24; 0.33]
Penedo, FJ, et al., 2020 II.	95	-20.49	4.78	97	-20.70	4.73	followup	24.00	0.04	[-0.24; 0.33]
Boele FW, et al ., 2017 II.	35	48.16	9.90	28	47.43	11.50	followup	12.00	0.07	[-0.43; 0.56]
Boele FW, et al ., 2017 I.	45	48.57	9.80	44	47.25	10.70	followup	12.00	0.13	[-0.29; 0.54]
Serfaty M, et al., 2018	20	16.30	8.80	22	14.60	5.60	followup	24.00	0.23	[-0.38; 0.84]
Boele FW, et al ., 2017 II.	35	51.07	11.70	28	48.47	10.50	followup	6.00	0.23	[-0.27; 0.73]
Walczak, A, et al.,2017	61	18.44	6.04	49	16.76	5.50	followup	4.00	0.29	[-0.09; 0.67]
Boele FW, et al ., 2017 I.	45	51.17	11.40	44	46.95	10.10	followup	6.00	0.39	[-0.03; 0.81]
Lu, Z, et al., 201	203	82.60	4.50	103	77.60	6.30	followup	9.00	0.96	[0.71; 1.21]
Huri M, et al., 2015	19	0.57	5.38	15	-41.73	6.14	followup	12.00	7.21	[5.27; 9.15]
Random effect	1747			1485					0.72	[-0.38; 1.82]
Prediction interval										[-2.52; 3.96]
**survivor**										
Chen, et al., 2017	58	61.20	7.50	65	63.10	7.30	baseline	0.00	-0.26	[-0.61; 0.10]
Qiu, H, et al., 2018 II.	98	7.72	5.28	196	8.58	5.92	baseline	0.00	-0.15	[-0.39; 0.09]
Peng, L, et al., 2022	28	78.33	12.78	29	79.08	10.04	baseline	0.00	-0.06	[-0.58; 0.45]
Jelvehzadeh, F, et al., 2022	24	3.15	1.52	24	3.21	2.08	baseline	0.00	-0.03	[-0.60; 0.53]
Qiu, H, et al., 2018 I.	98	8.71	5.89	196	8.58	5.92	baseline	0.00	0.02	[-0.22; 0.26]
McCusker, J, et al., 2021	121	41.60	9.80	124	40.70	9.20	baseline	0.00	0.09	[-0.16; 0.35]
Von Ah, D, et al., 2012 II.	27	44.69	4.40	29	43.18	4.72	baseline	0.00	0.33	[-0.20; 0.85]
Von Ah, D, et al., 2012 I.	26	45.71	5.98	29	43.18	4.72	baseline	0.00	0.47	[-0.07; 1.00]
Peng, L, et al., 2022	28	78.33	12.52	29	78.16	10.60	followup	0.10	0.01	[-0.50; 0.53]
Von Ah, D, et al., 2012 I.	26	45.03	6.17	29	44.49	4.81	followup	8.00	0.10	[-0.43; 0.63]
Von Ah, D, et al., 2012 II.	27	45.00	4.48	29	44.49	4.81	followup	8.00	0.11	[-0.42; 0.63]
Peng, L, et al., 2022	28	83.10	12.63	28	81.38	14.52	followup	4.00	0.12	[-0.40; 0.65]
Chen, et al., 2017	58	48.40	9.10	65	46.80	8.50	followup	24.00	0.18	[-0.17; 0.54]
Chen, et al., 2017	58	48.60	7.40	65	47.10	7.70	followup	8.00	0.20	[-0.16; 0.55]
Qiu, H, et al., 2018 II.	98	9.83	9.73	196	8.01	8.85	followup	4.00	0.20	[-0.04; 0.44]
Qiu, H, et al., 2018 II.	98	14.04	11.82	196	11.52	12.78	followup	24.00	0.20	[-0.04; 0.45]
Qiu, H, et al., 2018 II.	98	12.99	11.82	196	10.18	11.80	followup	12.00	0.24	[-0.01; 0.48]
McCusker, J, et al., 2021	121	43.90	9.80	124	41.40	10.10	followup	24.00	0.25	[-0.00; 0.50]
Qiu, H, et al., 2018 I.	98	12.92	9.73	196	8.01	8.85	followup	4.00	0.54	[0.29; 0.78]
Qiu, H, et al., 2018 I.	98	16.92	11.12	196	10.18	11.80	followup	12.00	0.58	[0.33; 0.83]
Qiu, H, et al., 2018 I.	98	18.96	10.43	196	11.52	12.78	followup	24.00	0.62	[0.37; 0.86]
Jelvehzadeh, F, et al., 2022	24	5.64	1.73	24	3.70	1.68	followup	12.00	1.12	[0.51; 1.73]
Jelvehzadeh, F, et al., 2022	24	5.64	1.73	24	3.43	1.75	followup	8.00	1.25	[0.63; 1.87]
Random effect	1462			2285					0.40	[-0.02; 0.83]
Prediction interval										[-2.51; 3.31]
**Random effect**	**5558**			**6043**					**0.68**	**[0.14; 1.21]**
**Prediction interval**										**[-2.49; 3.85]**

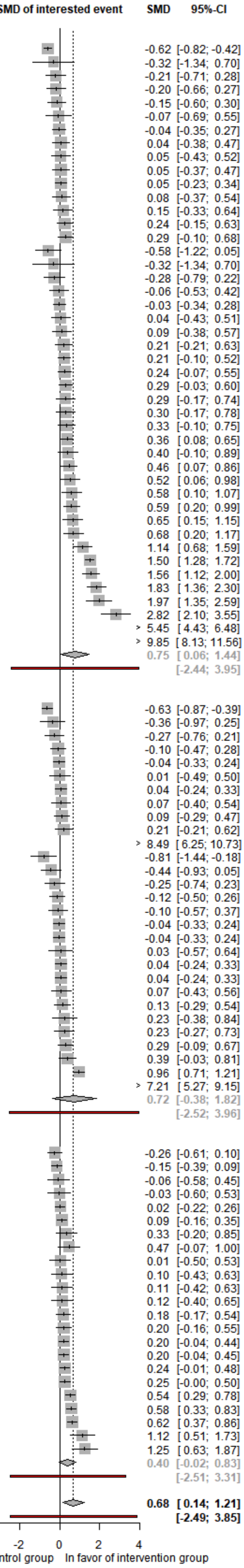

Figure S21.4. Forest plot represents the difference between the intervention vs. control group in the Physical QoL domain with the cancer stage subgroups as predicted at week 48 (post-intervention). SMD - Standardized mean difference, CI - confidence interval.

S22.Subgroup analysis of Physical QoL: Cancer type

Figure S22.1.T0

Study	Experimental Patient N	Experimental Mean	Experimental SD	Control Patient N	Control Mean	Control SD	follow-up	Follow-up time	SMD	95%-CI
**breast**										
Rahmani, S, et al. 2015	12	61.66	8.59	12	65.00	12.75	baseline	0.00	-0.30	[-1.10; 0.51]
Heiney, SP, et al., 2003	33	6.60	1.90	33	7.10	1.70	baseline	0.00	-0.27	[-0.76; 0.21]
Nápoles AM, et al. 2015	76	15.29	5.78	75	16.76	5.02	baseline	0.00	-0.27	[-0.59; 0.05]
Dirksen, S. et al, 2007 I.	34	22.00	5.60	38	23.10	4.10	baseline	0.00	-0.22	[-0.69; 0.24]
Ruiz-Vozmediano, J, et al.,2020	31	75.40	19.50	32	79.20	15.70	baseline	0.00	-0.21	[-0.71; 0.28]
Qiu, H, et al., 2018 II.	98	7.72	5.28	196	8.58	5.92	baseline	0.00	-0.15	[-0.39; 0.09]
Ding, K, et al. 2020	34	72.76	6.03	40	73.17	5.52	baseline	0.00	-0.07	[-0.53; 0.39]
Ferguson, RJ, et al., 2012	19	7.68	1.95	21	7.79	1.27	baseline	0.00	-0.07	[-0.69; 0.55]
Peng, L, et al., 2022	28	78.33	12.78	29	79.08	10.04	baseline	0.00	-0.06	[-0.58; 0.45]
Jelvehzadeh, F, et al., 2022	24	3.15	1.52	24	3.21	2.08	baseline	0.00	-0.03	[-0.60; 0.53]
Kim, YH, et al., 2017	30	76.00	14.30	30	76.40	15.20	baseline	0.00	-0.03	[-0.53; 0.48]
Hoffman, CJ, et al., 2012 I.	102	21.88	4.29	111	21.89	4.35	baseline	0.00	-0.00	[-0.27; 0.27]
Dirksen, S. et al, 2007 II.	34	19.10	4.00	38	19.10	4.70	baseline	0.00	0.00	[-0.46; 0.46]
Qiu, H, et al., 2018 I.	98	8.71	5.89	196	8.58	5.92	baseline	0.00	0.02	[-0.22; 0.26]
Hoffman, CJ, et al., 2012 II.	102	17.83	5.03	110	17.65	5.83	baseline	0.00	0.03	[-0.24; 0.30]
Fillion, L, et al. 2008	44	40.49	9.50	43	40.00	9.43	baseline	0.00	0.05	[-0.37; 0.47]
Klinkhammer-Schalke, M et al., 2012	100	78.68	26.40	100	76.65	27.16	baseline	0.00	0.08	[-0.20; 0.35]
Klafke, N, et al., 2019	120	85.10	19.30	113	82.60	20.20	baseline	0.00	0.13	[-0.13; 0.38]
Cengiz, HO, et al., 2023	32	14.15	7.56	33	13.03	6.82	baseline	0.00	0.15	[-0.33; 0.64]
Arving, C, et al., 2007	47	81.00	17.00	38	77.00	18.00	baseline	0.00	0.23	[-0.20; 0.66]
Hernandez, EG, et al. 2018 I.	28	18.93	5.70	28	17.57	5.96	baseline	0.00	0.23	[-0.30; 0.76]
Elyasi, F, et al., 2021 IV.	20	11.60	2.90	20	10.50	3.90	baseline	0.00	0.31	[-0.31; 0.94]
Hernandez, EG, et al. 2018 II.	28	15.75	6.10	28	13.82	5.68	baseline	0.00	0.32	[-0.20; 0.85]
Von Ah, D, et al., 2012 II.	27	44.69	4.40	29	43.18	4.72	baseline	0.00	0.33	[-0.20; 0.85]
Elyasi, F, et al., 2021 III.	20	21.00	4.60	20	18.90	6.40	baseline	0.00	0.37	[-0.26; 0.99]
Elyasi, F, et al., 2021 II.	15	11.90	3.20	15	10.50	3.90	baseline	0.00	0.38	[-0.34; 1.10]
Elyasi, F, et al., 2021 I.	15	21.00	1.40	15	18.90	6.40	baseline	0.00	0.44	[-0.28; 1.17]
Von Ah, D, et al., 2012 I.	26	45.71	5.98	29	43.18	4.72	baseline	0.00	0.47	[-0.07; 1.00]
Ferguson, RJ, et al., 2012	19	7.04	1.79	21	7.91	1.10	followup	8.00	-0.58	[-1.22; 0.05]
Heiney, SP, et al., 2003	33	6.40	1.40	33	7.20	2.10	followup	16.00	-0.44	[-0.93; 0.05]
Heiney, SP, et al., 2003	33	6.40	1.80	33	6.90	2.10	followup	6.00	-0.25	[-0.74; 0.23]
Elyasi, F, et al., 2021 III.	20	17.60	2.70	20	18.70	6.20	followup	24.00	-0.23	[-0.85; 0.40]
Klinkhammer-Schalke, M et al., 2012	100	77.91	23.85	100	79.69	20.55	followup	12.00	-0.08	[-0.36; 0.20]
Elyasi, F, et al., 2021 IV.	20	10.50	2.10	20	10.50	3.70	followup	24.00	0.00	[-0.62; 0.62]
Rahmani, S, et al. 2015	12	12.00	69.44	12	12.00	58.33	followup	16.00	0.00	[-0.80; 0.80]
Peng, L, et al., 2022	28	78.33	12.52	29	78.16	10.60	followup	0.10	0.01	[-0.50; 0.53]
Nápoles AM, et al. 2015	76	18.13	4.81	75	18.01	4.44	followup	12.00	0.03	[-0.29; 0.34]
Arving, C, et al., 2007	47	79.00	20.00	38	78.00	17.00	followup	12.00	0.05	[-0.37; 0.48]
Klafke, N, et al., 2019	120	68.70	23.50	113	67.30	22.60	followup	24.00	0.06	[-0.20; 0.32]
Klinkhammer-Schalke, M et al., 2012	100	80.20	20.30	100	78.42	22.07	followup	24.00	0.08	[-0.19; 0.36]
Von Ah, D, et al., 2012 I.	26	45.03	6.17	29	44.49	4.81	followup	8.00	0.10	[-0.43; 0.63]
Von Ah, D, et al., 2012 II.	27	45.00	4.48	29	44.49	4.81	followup	8.00	0.11	[-0.42; 0.63]
Arving, C, et al., 2007	47	80.00	19.00	38	78.00	17.00	followup	4.00	0.11	[-0.32; 0.54]
Peng, L, et al., 2022	28	83.10	12.63	28	81.38	14.52	followup	4.00	0.12	[-0.40; 0.65]
Klinkhammer-Schalke, M et al., 2012	100	83.25	22.34	100	80.20	21.57	followup	48.00	0.14	[-0.14; 0.42]
Dirksen, S. et al, 2007 I.	34	24.80	3.30	38	24.30	3.80	followup	10.00	0.14	[-0.32; 0.60]
Arving, C, et al., 2007	47	82.00	19.00	38	79.00	18.00	followup	24.00	0.16	[-0.27; 0.59]
Klafke, N, et al., 2019	120	80.90	21.60	113	77.00	21.40	followup	48.00	0.18	[-0.08; 0.44]
Qiu, H, et al., 2018 II.	98	9.83	9.73	196	8.01	8.85	followup	4.00	0.20	[-0.04; 0.44]
Qiu, H, et al., 2018 II.	98	14.04	11.82	196	11.52	12.78	followup	24.00	0.20	[-0.04; 0.45]
Fillion, L, et al. 2008	44	46.76	9.24	43	44.64	11.05	followup	12.00	0.21	[-0.21; 0.63]
Klafke, N, et al., 2019	120	75.30	20.20	113	70.70	21.70	followup	12.00	0.22	[-0.04; 0.48]
Nápoles AM, et al. 2015	76	19.44	4.26	75	18.44	4.58	followup	24.00	0.23	[-0.10; 0.55]
Hoffman, CJ, et al., 2012 I.	102	22.86	4.22	111	21.84	4.54	followup	8.00	0.23	[-0.04; 0.50]
Qiu, H, et al., 2018 II.	98	12.99	11.82	196	10.18	11.80	followup	12.00	0.24	[-0.01; 0.48]
Elyasi, F, et al., 2021 I.	15	20.00	3.90	15	18.70	6.20	followup	24.00	0.24	[-0.47; 0.96]
Klinkhammer-Schalke, M et al., 2012	100	84.26	19.79	100	78.68	23.35	followup	36.00	0.26	[-0.02; 0.54]
Dirksen, S. et al, 2007 II.	34	22.70	4.20	38	21.50	4.70	followup	10.00	0.27	[-0.20; 0.73]
Elyasi, F, et al., 2021 II.	15	11.40	2.60	15	10.50	3.70	followup	24.00	0.27	[-0.45; 0.99]
Hoffman, CJ, et al., 2012 I.	102	22.97	4.34	111	21.67	4.87	followup	12.00	0.28	[0.01; 0.55]
Fillion, L, et al. 2008	44	45.10	10.42	43	41.76	9.76	followup	4.00	0.33	[-0.10; 0.75]
Hoffman, CJ, et al., 2012 II.	102	19.45	5.32	110	17.53	5.37	followup	12.00	0.36	[0.09; 0.63]
Hoffman, CJ, et al., 2012 II.	102	19.46	5.27	110	17.41	6.06	followup	8.00	0.36	[0.09; 0.63]
Kim, YH, et al., 2017	30	75.10	14.20	30	68.90	18.10	followup	6.00	0.38	[-0.13; 0.89]
Ruiz-Vozmediano, J, et al.,2020	31	85.00	14.10	32	78.30	18.90	followup	24.00	0.40	[-0.10; 0.89]
Kim, YH, et al., 2017	30	75.60	17.40	30	68.40	18.10	followup	9.00	0.40	[-0.11; 0.91]
Hernandez, EG, et al. 2018 II.	28	17.64	4.61	28	15.56	4.81	followup	24.00	0.44	[-0.10; 0.97]
Qiu, H, et al., 2018 I.	98	12.92	9.73	196	8.01	8.85	followup	4.00	0.54	[0.29; 0.78]
Hernandez, EG, et al. 2018 II.	28	17.54	3.20	28	15.40	4.39	followup	8.00	0.55	[0.02; 1.08]
Qiu, H, et al., 2018 I.	98	16.92	11.12	196	10.18	11.80	followup	12.00	0.58	[0.33; 0.83]
Qiu, H, et al., 2018 I.	98	18.96	10.43	196	11.52	12.78	followup	24.00	0.62	[0.37; 0.86]
Cengiz, HO, et al., 2023	32	16.75	6.33	33	12.33	7.00	followup	8.00	0.65	[0.15; 1.15]
Hernandez, EG, et al. 2018 I.	28	21.50	3.39	28	17.68	6.16	followup	8.00	0.76	[0.21; 1.30]
Hernandez, EG, et al. 2018 I.	28	21.27	4.09	28	17.52	5.34	followup	24.00	0.78	[0.23; 1.32]
Rahmani, S, et al. 2015	12	72.77	3.43	12	66.11	10.81	followup	8.00	0.80	[-0.04; 1.64]
Jelvehzadeh, F, et al., 2022	24	5.64	1.73	24	3.70	1.68	followup	12.00	1.12	[0.51; 1.73]
Jelvehzadeh, F, et al., 2022	24	5.64	1.73	24	3.43	1.75	followup	8.00	1.25	[0.63; 1.87]
Ding, K, et al. 2020	34	104.47	7.43	40	82.38	5.42	followup	4.00	3.40	[2.68; 4.13]
Random effect	4087			4900					0.55	[0.20; 0.89]
Prediction interval										[-0.63; 1.72]
**gastroenterological**										
Cheung YL, et al. 2002	29	15.75	1.75	30	17.50	3.83	baseline	0.00	-0.58	[-1.10; -0.06]
Zhang, LMM, et al., 2021	80	58.64	16.46	80	59.25	16.46	baseline	0.00	-0.04	[-0.35; 0.27]
Qin, X, et al., 2017	50	78.56	8.38	50	78.53	11.59	baseline	0.00	0.00	[-0.39; 0.39]
Baoyindeligeer, L.Z. et al. 2020	65	62.75	13.46	65	62.35	12.28	baseline	0.00	0.03	[-0.31; 0.37]
Zhang, LMM, et al., 2021	80	62.34	16.46	80	62.75	15.02	followup	12.00	-0.03	[-0.34; 0.28]
Zhang, LMM, et al., 2021	80	67.90	15.85	80	64.81	13.17	followup	24.00	0.21	[-0.10; 0.52]
Zhang, LMM, et al., 2021	80	72.01	14.81	80	68.72	12.35	followup	36.00	0.24	[-0.07; 0.55]
Zhang, LMM, et al., 2021	80	74.27	15.02	80	70.16	13.58	followup	48.00	0.29	[-0.03; 0.60]
Qin, X, et al., 2017	50	89.42	12.35	50	80.37	14.32	followup	2.00	0.67	[0.27; 1.07]
Baoyindeligeer, L.Z. et al. 2020	65	84.04	12.72	65	72.65	13.25	followup	2.00	0.87	[0.51; 1.23]
Cheung YL, et al. 2002	29	26.58	1.45	30	22.60	2.14	followup	10.00	2.14	[1.49; 2.79]
Cheung YL, et al. 2002	29	24.96	1.32	30	20.86	1.75	followup	5.00	2.60	[1.90; 3.31]
Random effect	717			720					0.81	[-0.03; 1.64]
Prediction interval										[-0.98; 2.60]
**gynaecological**										
Zhou, L, et al., 2020	37	13.65	1.84	36	14.85	1.47	baseline	0.00	-0.71	[-1.19; -0.24]
Powell, CB, et al., 2008 I.	21	21.60	5.80	43	22.10	7.40	baseline	0.00	-0.07	[-0.59; 0.45]
Chan, et al. 2005	80	65.81	61.89	75	62.85	68.50	baseline	0.00	0.05	[-0.27; 0.36]
Sandsund, C, et al., 2017	72	77.20	20.20	70	75.50	22.30	baseline	0.00	0.08	[-0.25; 0.41]
Powell, CB, et al., 2008 II.	21	18.90	6.20	43	18.00	6.00	baseline	0.00	0.15	[-0.38; 0.67]
Chan, et al. 2005	80	56.52	61.89	75	72.73	75.34	followup	12.00	-0.23	[-0.55; 0.08]
Chan, et al. 2005	80	78.26	99.01	75	92.89	37.67	followup	72.00	-0.19	[-0.51; 0.12]
Chan, et al. 2005	80	67.00	67.17	75	78.06	58.20	followup	24.00	-0.17	[-0.49; 0.14]
Chan, et al. 2005	80	81.23	81.30	75	86.76	65.04	followup	60.00	-0.07	[-0.39; 0.24]
Chan, et al. 2005	80	82.61	67.17	75	86.96	54.73	followup	48.00	-0.07	[-0.39; 0.24]
Chan, et al. 2005	80	83.00	63.59	75	83.60	59.93	followup	36.00	-0.01	[-0.32; 0.31]
Sandsund, C, et al., 2017	72	80.10	21.40	70	79.00	20.00	followup	12.00	0.05	[-0.28; 0.38]
Sandsund, C, et al., 2017	72	81.70	18.10	70	79.20	19.90	followup	24.00	0.13	[-0.20; 0.46]
Powell, CB, et al., 2008 I.	21	25.40	3.30	43	22.60	6.10	followup	12.00	0.52	[-0.01; 1.05]
Powell, CB, et al., 2008 II.	21	22.60	5.80	43	17.40	7.40	followup	12.00	0.74	[0.20; 1.28]
Zhou, L, et al., 2020	37	45.12	5.62	36	38.94	5.19	followup	12.00	1.13	[0.63; 1.63]
Random effect	934			979					0.50	[0.03; 0.98]
Prediction interval										[-0.95; 1.95]
**prostate**										
Berglund, G. et al. 2007	39	73.30	23.00	150	84.60	18.30	baseline	0.00	-0.58	[-0.94; -0.22]
Parker, PA, et al., 2009 I.	39	51.32	6.74	36	52.29	5.88	baseline	0.00	-0.15	[-0.60; 0.30]
Penedo, FJ, et al., 2020 I.	95	-23.26	3.80	97	-23.09	3.84	baseline	0.00	-0.04	[-0.33; 0.24]
Penedo, FJ, et al., 2020 II.	95	-20.27	4.68	97	-20.48	4.63	baseline	0.00	0.04	[-0.24; 0.33]
Penedo, FJ, et al., 2007	41	-23.98	28.88	30	-25.67	14.84	baseline	0.00	0.07	[-0.40; 0.54]
Parker, PA, et al., 2009 II.	39	52.79	6.12	36	52.29	5.88	baseline	0.00	0.08	[-0.37; 0.54]
Karlsen RV et al., 2021	16	58.60	3.10	19	55.80	5.40	baseline	0.00	0.61	[-0.07; 1.29]
Huri M, et al., 2015	19	36.68	8.73	15	-36.26	7.93	baseline	0.00	8.49	[6.25; 10.73]
Berglund, G. et al. 2007	39	78.20	22.50	150	87.80	16.90	followup	48.00	-0.53	[-0.88; -0.17]
Penedo, FJ, et al., 2007	41	-25.51	13.16	30	-23.83	21.36	followup	12.00	-0.10	[-0.57; 0.37]
Karlsen RV et al., 2021	16	53.20	9.40	19	53.80	6.60	followup	32.00	-0.07	[-0.74; 0.59]
Northhouse, LL, et al., 2007	107	43.30	6.60	121	43.60	6.50	followup	32.00	-0.05	[-0.31; 0.21]
Penedo, FJ, et al., 2020 I.	95	-23.29	4.00	97	-23.11	4.04	followup	48.00	-0.04	[-0.33; 0.24]
Penedo, FJ, et al., 2020 I.	95	-23.16	3.90	97	-22.99	3.94	followup	24.00	-0.04	[-0.33; 0.24]
Northhouse, LL, et al., 2007	112	48.60	6.70	123	48.70	6.50	followup	16.00	-0.02	[-0.27; 0.24]
Northhouse, LL, et al., 2007	104	42.70	6.50	114	42.50	6.40	followup	48.00	0.03	[-0.23; 0.30]
Parker, PA, et al., 2009 I.	38	48.86	8.88	32	48.51	9.50	followup	24.00	0.04	[-0.43; 0.51]
Penedo, FJ, et al., 2020 II.	95	-20.08	4.87	97	-20.29	4.83	followup	48.00	0.04	[-0.24; 0.33]
Penedo, FJ, et al., 2020 II.	95	-20.49	4.78	97	-20.70	4.73	followup	24.00	0.04	[-0.24; 0.33]
Parker, PA, et al., 2009 I.	39	47.28	9.18	36	44.63	9.12	followup	6.00	0.29	[-0.17; 0.74]
Parker, PA, et al., 2009 II.	38	51.36	9.18	32	48.51	9.50	followup	24.00	0.30	[-0.17; 0.78]
Parker, PA, et al., 2009 II.	39	49.58	9.74	36	44.63	9.12	followup	6.00	0.52	[0.06; 0.98]
Parker, PA, et al., 2009 I.	37	50.22	8.46	32	45.12	8.88	followup	48.00	0.58	[0.10; 1.07]
Parker, PA, et al., 2009 II.	37	51.76	10.16	32	45.12	8.88	followup	48.00	0.68	[0.20; 1.17]
Huri M, et al., 2015	19	0.57	5.38	15	-41.73	6.14	followup	12.00	7.21	[5.27; 9.15]
Random effect	1429			1640					0.73	[-0.14; 1.60]
Prediction interval										[-0.73; 2.19]
**Random effect**	**7167**			**8239**					**0.58**	**[0.22; 0.95]**
**Prediction interval**										**[-0.41; 1.57]**

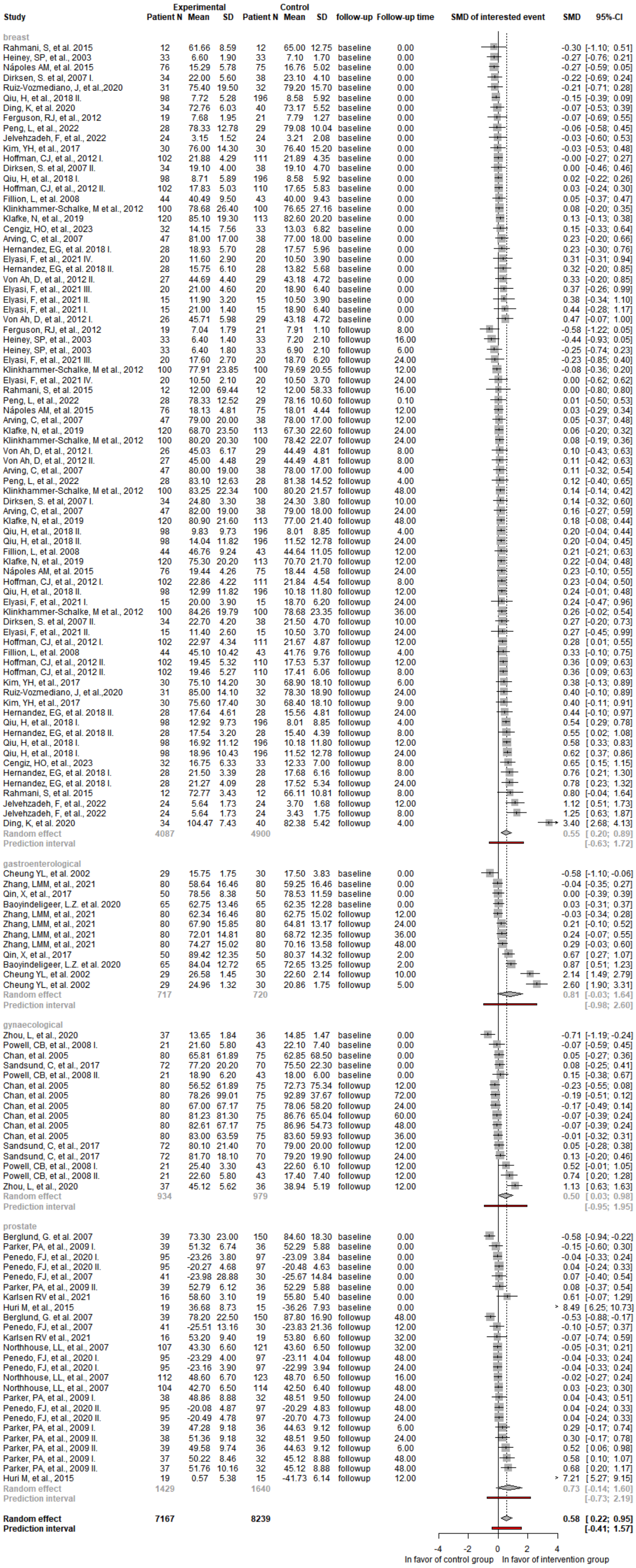

Figure S22.1. Subgroup analysis of the Physical QoL. Forest plot represents the difference between the intervention vs. control group in the Physical QoL domain with the cancer type subgroups as predicted at week 0 (post-intervention). SMD - Standardized mean difference, CI - confidence interval.

Figure S22.2.T12

Study	Experimental Patient N	Experimental Mean	Experimental SD	Control Patient N	Control Mean	Control SD	follow-up	Follow-up time	SMD	95%-CI
**breast**										
Rahmani, S, et al. 2015	12	61.66	8.59	12	65.00	12.75	baseline	0.00	-0.30	[-1.10; 0.51]
Heiney, SP, et al., 2003	33	6.60	1.90	33	7.10	1.70	baseline	0.00	-0.27	[-0.76; 0.21]
Nápoles AM, et al. 2015	76	15.29	5.78	75	16.76	5.02	baseline	0.00	-0.27	[-0.59; 0.05]
Dirksen, S. et al, 2007 I.	34	22.00	5.60	38	23.10	4.10	baseline	0.00	-0.22	[-0.69; 0.24]
Ruiz-Vozmediano, J, et al.,2020	31	75.40	19.50	32	79.20	15.70	baseline	0.00	-0.21	[-0.71; 0.28]
Qiu, H, et al., 2018 II.	98	7.72	5.28	196	8.58	5.92	baseline	0.00	-0.15	[-0.39; 0.09]
Ding, K, et al. 2020	34	72.76	6.03	40	73.17	5.52	baseline	0.00	-0.07	[-0.53; 0.39]
Ferguson, RJ, et al., 2012	19	7.68	1.95	21	7.79	1.27	baseline	0.00	-0.07	[-0.69; 0.55]
Peng, L, et al., 2022	28	78.33	12.78	29	79.08	10.04	baseline	0.00	-0.06	[-0.58; 0.45]
Jelvehzadeh, F, et al., 2022	24	3.15	1.52	24	3.21	2.08	baseline	0.00	-0.03	[-0.60; 0.53]
Kim, YH, et al., 2017	30	76.00	14.30	30	76.40	15.20	baseline	0.00	-0.03	[-0.53; 0.48]
Hoffman, CJ, et al., 2012 I.	102	21.88	4.29	111	21.89	4.35	baseline	0.00	-0.00	[-0.27; 0.27]
Dirksen, S. et al, 2007 II.	34	19.10	4.00	38	19.10	4.70	baseline	0.00	0.00	[-0.46; 0.46]
Qiu, H, et al., 2018 I.	98	8.71	5.89	196	8.58	5.92	baseline	0.00	0.02	[-0.22; 0.26]
Hoffman, CJ, et al., 2012 II.	102	17.83	5.03	110	17.65	5.83	baseline	0.00	0.03	[-0.24; 0.30]
Fillion, L, et al. 2008	44	40.49	9.50	43	40.00	9.43	baseline	0.00	0.05	[-0.37; 0.47]
Klinkhammer-Schalke, M et al., 2012	100	78.68	26.40	100	76.65	27.16	baseline	0.00	0.08	[-0.20; 0.35]
Klafke, N, et al., 2019	120	85.10	19.30	113	82.60	20.20	baseline	0.00	0.13	[-0.13; 0.38]
Cengiz, HO, et al., 2023	32	14.15	7.56	33	13.03	6.82	baseline	0.00	0.15	[-0.33; 0.64]
Arving, C, et al., 2007	47	81.00	17.00	38	77.00	18.00	baseline	0.00	0.23	[-0.20; 0.66]
Hernandez, EG, et al. 2018 I.	28	18.93	5.70	28	17.57	5.96	baseline	0.00	0.23	[-0.30; 0.76]
Elyasi, F, et al., 2021 IV.	20	11.60	2.90	20	10.50	3.90	baseline	0.00	0.31	[-0.31; 0.94]
Hernandez, EG, et al. 2018 II.	28	15.75	6.10	28	13.82	5.68	baseline	0.00	0.32	[-0.20; 0.85]
Von Ah, D, et al., 2012 II.	27	44.69	4.40	29	43.18	4.72	baseline	0.00	0.33	[-0.20; 0.85]
Elyasi, F, et al., 2021 III.	20	21.00	4.60	20	18.90	6.40	baseline	0.00	0.37	[-0.26; 0.99]
Elyasi, F, et al., 2021 II.	15	11.90	3.20	15	10.50	3.90	baseline	0.00	0.38	[-0.34; 1.10]
Elyasi, F, et al., 2021 I.	15	21.00	1.40	15	18.90	6.40	baseline	0.00	0.44	[-0.28; 1.17]
Von Ah, D, et al., 2012 I.	26	45.71	5.98	29	43.18	4.72	baseline	0.00	0.47	[-0.07; 1.00]
Ferguson, RJ, et al., 2012	19	7.04	1.79	21	7.91	1.10	followup	8.00	-0.58	[-1.22; 0.05]
Heiney, SP, et al., 2003	33	6.40	1.40	33	7.20	2.10	followup	16.00	-0.44	[-0.93; 0.05]
Heiney, SP, et al., 2003	33	6.40	1.80	33	6.90	2.10	followup	6.00	-0.25	[-0.74; 0.23]
Elyasi, F, et al., 2021 III.	20	17.60	2.70	20	18.70	6.20	followup	24.00	-0.23	[-0.85; 0.40]
Klinkhammer-Schalke, M et al., 2012	100	77.91	23.85	100	79.69	20.55	followup	12.00	-0.08	[-0.36; 0.20]
Elyasi, F, et al., 2021 IV.	20	10.50	2.10	20	10.50	3.70	followup	24.00	0.00	[-0.62; 0.62]
Rahmani, S, et al. 2015	12	12.00	69.44	12	12.00	58.33	followup	16.00	0.00	[-0.80; 0.80]
Peng, L, et al., 2022	28	78.33	12.52	29	78.16	10.60	followup	0.10	0.01	[-0.50; 0.53]
Nápoles AM, et al. 2015	76	18.13	4.81	75	18.01	4.44	followup	12.00	0.03	[-0.29; 0.34]
Arving, C, et al., 2007	47	79.00	20.00	38	78.00	17.00	followup	12.00	0.05	[-0.37; 0.48]
Klafke, N, et al., 2019	120	68.70	23.50	113	67.30	22.60	followup	24.00	0.06	[-0.20; 0.32]
Klinkhammer-Schalke, M et al., 2012	100	80.20	20.30	100	78.42	22.07	followup	24.00	0.08	[-0.19; 0.36]
Von Ah, D, et al., 2012 I.	26	45.03	6.17	29	44.49	4.81	followup	8.00	0.10	[-0.43; 0.63]
Von Ah, D, et al., 2012 II.	27	45.00	4.48	29	44.49	4.81	followup	8.00	0.11	[-0.42; 0.63]
Arving, C, et al., 2007	47	80.00	19.00	38	78.00	17.00	followup	4.00	0.11	[-0.32; 0.54]
Peng, L, et al., 2022	28	83.10	12.63	28	81.38	14.52	followup	4.00	0.12	[-0.40; 0.65]
Klinkhammer-Schalke, M et al., 2012	100	83.25	22.34	100	80.20	21.57	followup	48.00	0.14	[-0.14; 0.42]
Dirksen, S. et al, 2007 I.	34	24.80	3.30	38	24.30	3.80	followup	10.00	0.14	[-0.32; 0.60]
Arving, C, et al., 2007	47	82.00	19.00	38	79.00	18.00	followup	24.00	0.16	[-0.27; 0.59]
Klafke, N, et al., 2019	120	80.90	21.60	113	77.00	21.40	followup	48.00	0.18	[-0.08; 0.44]
Qiu, H, et al., 2018 II.	98	9.83	9.73	196	8.01	8.85	followup	4.00	0.20	[-0.04; 0.44]
Qiu, H, et al., 2018 II.	98	14.04	11.82	196	11.52	12.78	followup	24.00	0.20	[-0.04; 0.45]
Fillion, L, et al. 2008	44	46.76	9.24	43	44.64	11.05	followup	12.00	0.21	[-0.21; 0.63]
Klafke, N, et al., 2019	120	75.30	20.20	113	70.70	21.70	followup	12.00	0.22	[-0.04; 0.48]
Nápoles AM, et al. 2015	76	19.44	4.26	75	18.44	4.58	followup	24.00	0.23	[-0.10; 0.55]
Hoffman, CJ, et al., 2012 I.	102	22.86	4.22	111	21.84	4.54	followup	8.00	0.23	[-0.04; 0.50]
Qiu, H, et al., 2018 II.	98	12.99	11.82	196	10.18	11.80	followup	12.00	0.24	[-0.01; 0.48]
Elyasi, F, et al., 2021 I.	15	20.00	3.90	15	18.70	6.20	followup	24.00	0.24	[-0.47; 0.96]
Klinkhammer-Schalke, M et al., 2012	100	84.26	19.79	100	78.68	23.35	followup	36.00	0.26	[-0.02; 0.54]
Dirksen, S. et al, 2007 II.	34	22.70	4.20	38	21.50	4.70	followup	10.00	0.27	[-0.20; 0.73]
Elyasi, F, et al., 2021 II.	15	11.40	2.60	15	10.50	3.70	followup	24.00	0.27	[-0.45; 0.99]
Hoffman, CJ, et al., 2012 I.	102	22.97	4.34	111	21.67	4.87	followup	12.00	0.28	[0.01; 0.55]
Fillion, L, et al. 2008	44	45.10	10.42	43	41.76	9.76	followup	4.00	0.33	[-0.10; 0.75]
Hoffman, CJ, et al., 2012 II.	102	19.45	5.32	110	17.53	5.37	followup	12.00	0.36	[0.09; 0.63]
Hoffman, CJ, et al., 2012 II.	102	19.46	5.27	110	17.41	6.06	followup	8.00	0.36	[0.09; 0.63]
Kim, YH, et al., 2017	30	75.10	14.20	30	68.90	18.10	followup	6.00	0.38	[-0.13; 0.89]
Ruiz-Vozmediano, J, et al.,2020	31	85.00	14.10	32	78.30	18.90	followup	24.00	0.40	[-0.10; 0.89]
Kim, YH, et al., 2017	30	75.60	17.40	30	68.40	18.10	followup	9.00	0.40	[-0.11; 0.91]
Hernandez, EG, et al. 2018 II.	28	17.64	4.61	28	15.56	4.81	followup	24.00	0.44	[-0.10; 0.97]
Qiu, H, et al., 2018 I.	98	12.92	9.73	196	8.01	8.85	followup	4.00	0.54	[0.29; 0.78]
Hernandez, EG, et al. 2018 II.	28	17.54	3.20	28	15.40	4.39	followup	8.00	0.55	[0.02; 1.08]
Qiu, H, et al., 2018 I.	98	16.92	11.12	196	10.18	11.80	followup	12.00	0.58	[0.33; 0.83]
Qiu, H, et al., 2018 I.	98	18.96	10.43	196	11.52	12.78	followup	24.00	0.62	[0.37; 0.86]
Cengiz, HO, et al., 2023	32	16.75	6.33	33	12.33	7.00	followup	8.00	0.65	[0.15; 1.15]
Hernandez, EG, et al. 2018 I.	28	21.50	3.39	28	17.68	6.16	followup	8.00	0.76	[0.21; 1.30]
Hernandez, EG, et al. 2018 I.	28	21.27	4.09	28	17.52	5.34	followup	24.00	0.78	[0.23; 1.32]
Rahmani, S, et al. 2015	12	72.77	3.43	12	66.11	10.81	followup	8.00	0.80	[-0.04; 1.64]
Jelvehzadeh, F, et al., 2022	24	5.64	1.73	24	3.70	1.68	followup	12.00	1.12	[0.51; 1.73]
Jelvehzadeh, F, et al., 2022	24	5.64	1.73	24	3.43	1.75	followup	8.00	1.25	[0.63; 1.87]
Ding, K, et al. 2020	34	104.47	7.43	40	82.38	5.42	followup	4.00	3.40	[2.68; 4.13]
Random effect	4087			4900					0.36	[0.19; 0.53]
Prediction interval										[-0.77; 1.49]
**gastroenterological**										
Cheung YL, et al. 2002	29	15.75	1.75	30	17.50	3.83	baseline	0.00	-0.58	[-1.10; -0.06]
Zhang, LMM, et al., 2021	80	58.64	16.46	80	59.25	16.46	baseline	0.00	-0.04	[-0.35; 0.27]
Qin, X, et al., 2017	50	78.56	8.38	50	78.53	11.59	baseline	0.00	0.00	[-0.39; 0.39]
Baoyindeligeer, L.Z. et al. 2020	65	62.75	13.46	65	62.35	12.28	baseline	0.00	0.03	[-0.31; 0.37]
Zhang, LMM, et al., 2021	80	62.34	16.46	80	62.75	15.02	followup	12.00	-0.03	[-0.34; 0.28]
Zhang, LMM, et al., 2021	80	67.90	15.85	80	64.81	13.17	followup	24.00	0.21	[-0.10; 0.52]
Zhang, LMM, et al., 2021	80	72.01	14.81	80	68.72	12.35	followup	36.00	0.24	[-0.07; 0.55]
Zhang, LMM, et al., 2021	80	74.27	15.02	80	70.16	13.58	followup	48.00	0.29	[-0.03; 0.60]
Qin, X, et al., 2017	50	89.42	12.35	50	80.37	14.32	followup	2.00	0.67	[0.27; 1.07]
Baoyindeligeer, L.Z. et al. 2020	65	84.04	12.72	65	72.65	13.25	followup	2.00	0.87	[0.51; 1.23]
Cheung YL, et al. 2002	29	26.58	1.45	30	22.60	2.14	followup	10.00	2.14	[1.49; 2.79]
Cheung YL, et al. 2002	29	24.96	1.32	30	20.86	1.75	followup	5.00	2.60	[1.90; 3.31]
Random effect	717			720					0.62	[-0.12; 1.36]
Prediction interval										[-1.24; 2.49]
**gynaecological**										
Zhou, L, et al., 2020	37	13.65	1.84	36	14.85	1.47	baseline	0.00	-0.71	[-1.19; -0.24]
Powell, CB, et al., 2008 I.	21	21.60	5.80	43	22.10	7.40	baseline	0.00	-0.07	[-0.59; 0.45]
Chan, et al. 2005	80	65.81	61.89	75	62.85	68.50	baseline	0.00	0.05	[-0.27; 0.36]
Sandsund, C, et al., 2017	72	77.20	20.20	70	75.50	22.30	baseline	0.00	0.08	[-0.25; 0.41]
Powell, CB, et al., 2008 II.	21	18.90	6.20	43	18.00	6.00	baseline	0.00	0.15	[-0.38; 0.67]
Chan, et al. 2005	80	56.52	61.89	75	72.73	75.34	followup	12.00	-0.23	[-0.55; 0.08]
Chan, et al. 2005	80	78.26	99.01	75	92.89	37.67	followup	72.00	-0.19	[-0.51; 0.12]
Chan, et al. 2005	80	67.00	67.17	75	78.06	58.20	followup	24.00	-0.17	[-0.49; 0.14]
Chan, et al. 2005	80	81.23	81.30	75	86.76	65.04	followup	60.00	-0.07	[-0.39; 0.24]
Chan, et al. 2005	80	82.61	67.17	75	86.96	54.73	followup	48.00	-0.07	[-0.39; 0.24]
Chan, et al. 2005	80	83.00	63.59	75	83.60	59.93	followup	36.00	-0.01	[-0.32; 0.31]
Sandsund, C, et al., 2017	72	80.10	21.40	70	79.00	20.00	followup	12.00	0.05	[-0.28; 0.38]
Sandsund, C, et al., 2017	72	81.70	18.10	70	79.20	19.90	followup	24.00	0.13	[-0.20; 0.46]
Powell, CB, et al., 2008 I.	21	25.40	3.30	43	22.60	6.10	followup	12.00	0.52	[-0.01; 1.05]
Powell, CB, et al., 2008 II.	21	22.60	5.80	43	17.40	7.40	followup	12.00	0.74	[0.20; 1.28]
Zhou, L, et al., 2020	37	45.12	5.62	36	38.94	5.19	followup	12.00	1.13	[0.63; 1.63]
Random effect	934			979					0.31	[0.02; 0.61]
Prediction interval										[-1.21; 1.83]
**prostate**										
Berglund, G. et al. 2007	39	73.30	23.00	150	84.60	18.30	baseline	0.00	-0.58	[-0.94; -0.22]
Parker, PA, et al., 2009 I.	39	51.32	6.74	36	52.29	5.88	baseline	0.00	-0.15	[-0.60; 0.30]
Penedo, FJ, et al., 2020 I.	95	-23.26	3.80	97	-23.09	3.84	baseline	0.00	-0.04	[-0.33; 0.24]
Penedo, FJ, et al., 2020 II.	95	-20.27	4.68	97	-20.48	4.63	baseline	0.00	0.04	[-0.24; 0.33]
Penedo, FJ, et al., 2007	41	-23.98	28.88	30	-25.67	14.84	baseline	0.00	0.07	[-0.40; 0.54]
Parker, PA, et al., 2009 II.	39	52.79	6.12	36	52.29	5.88	baseline	0.00	0.08	[-0.37; 0.54]
Karlsen RV et al., 2021	16	58.60	3.10	19	55.80	5.40	baseline	0.00	0.61	[-0.07; 1.29]
Huri M, et al., 2015	19	36.68	8.73	15	-36.26	7.93	baseline	0.00	8.49	[6.25; 10.73]
Berglund, G. et al. 2007	39	78.20	22.50	150	87.80	16.90	followup	48.00	-0.53	[-0.88; -0.17]
Penedo, FJ, et al., 2007	41	-25.51	13.16	30	-23.83	21.36	followup	12.00	-0.10	[-0.57; 0.37]
Karlsen RV et al., 2021	16	53.20	9.40	19	53.80	6.60	followup	32.00	-0.07	[-0.74; 0.59]
Northhouse, LL, et al., 2007	107	43.30	6.60	121	43.60	6.50	followup	32.00	-0.05	[-0.31; 0.21]
Penedo, FJ, et al., 2020 I.	95	-23.29	4.00	97	-23.11	4.04	followup	48.00	-0.04	[-0.33; 0.24]
Penedo, FJ, et al., 2020 I.	95	-23.16	3.90	97	-22.99	3.94	followup	24.00	-0.04	[-0.33; 0.24]
Northhouse, LL, et al., 2007	112	48.60	6.70	123	48.70	6.50	followup	16.00	-0.02	[-0.27; 0.24]
Northhouse, LL, et al., 2007	104	42.70	6.50	114	42.50	6.40	followup	48.00	0.03	[-0.23; 0.30]
Parker, PA, et al., 2009 I.	38	48.86	8.88	32	48.51	9.50	followup	24.00	0.04	[-0.43; 0.51]
Penedo, FJ, et al., 2020 II.	95	-20.08	4.87	97	-20.29	4.83	followup	48.00	0.04	[-0.24; 0.33]
Penedo, FJ, et al., 2020 II.	95	-20.49	4.78	97	-20.70	4.73	followup	24.00	0.04	[-0.24; 0.33]
Parker, PA, et al., 2009 I.	39	47.28	9.18	36	44.63	9.12	followup	6.00	0.29	[-0.17; 0.74]
Parker, PA, et al., 2009 II.	38	51.36	9.18	32	48.51	9.50	followup	24.00	0.30	[-0.17; 0.78]
Parker, PA, et al., 2009 II.	39	49.58	9.74	36	44.63	9.12	followup	6.00	0.52	[0.06; 0.98]
Parker, PA, et al., 2009 I.	37	50.22	8.46	32	45.12	8.88	followup	48.00	0.58	[0.10; 1.07]
Parker, PA, et al., 2009 II.	37	51.76	10.16	32	45.12	8.88	followup	48.00	0.68	[0.20; 1.17]
Huri M, et al., 2015	19	0.57	5.38	15	-41.73	6.14	followup	12.00	7.21	[5.27; 9.15]
Random effect	1429			1640					0.55	[-0.28; 1.37]
Prediction interval										[-0.95; 2.04]
**Random effect**	**7167**			**8239**					**0.39**	**[0.21; 0.57]**
**Prediction interval**										**[-0.53; 1.31]**

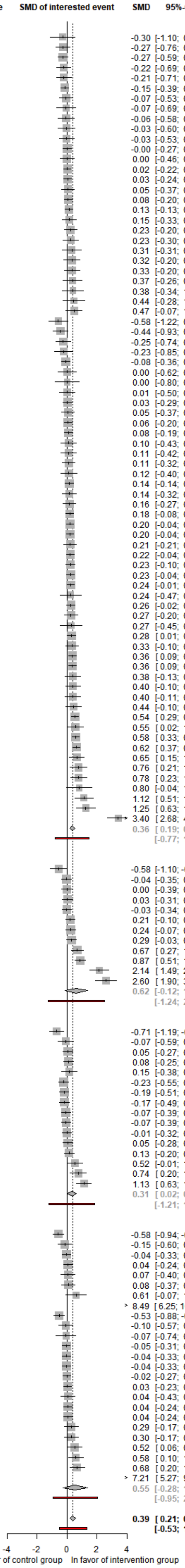

Figure S22.2. Subgroup analysis of the Physical QoL. Forest plot represents the difference between the intervention vs. control group in the Physical QoL domain with the cancer type subgroups as predicted at week 12 (post-intervention). SMD - Standardized mean difference, CI - confidence interval.

Figure S22.3.T24

Study	Experimental Patient N	Experimental Mean	Experimental SD	Control Patient N	Control Mean	Control SD	follow-up	Follow-up time	SMD of interested event	SMD	95%-CI
**breast**											
Rahmani, S, et al. 2015	12	61.66	8.59	12	65.00	12.75	baseline	0.00		-0.30	[-1.10; 0.51]
Heiney, SP, et al., 2003	33	6.60	1.90	33	7.10	1.70	baseline	0.00		-0.27	[-0.76; 0.21]
Nápoles AM, et al. 2015	76	15.29	5.78	75	16.76	5.02	baseline	0.00		-0.27	[-0.59; 0.05]
Dirksen, S. et al, 2007 I.	34	22.00	5.60	38	23.10	4.10	baseline	0.00		-0.22	[-0.69; 0.24]
Ruiz-Vozmediano, J, et al.,2020	31	75.40	19.50	32	79.20	15.70	baseline	0.00		-0.21	[-0.71; 0.28]
Qiu, H, et al., 2018 II.	98	7.72	5.28	196	8.58	5.92	baseline	0.00		-0.15	[-0.39; 0.09]
Ding, K, et al. 2020	34	72.76	6.03	40	73.17	5.52	baseline	0.00		-0.07	[-0.53; 0.39]
Ferguson, RJ, et al., 2012	19	7.68	1.95	21	7.79	1.27	baseline	0.00		-0.07	[-0.69; 0.55]
Peng, L, et al., 2022	28	78.33	12.78	29	79.08	10.04	baseline	0.00		-0.06	[-0.58; 0.45]
Jelvehzadeh, F, et al., 2022	24	3.15	1.52	24	3.21	2.08	baseline	0.00		-0.03	[-0.60; 0.53]
Kim, YH, et al., 2017	30	76.00	14.30	30	76.40	15.20	baseline	0.00		-0.03	[-0.53; 0.48]
Hoffman, CJ, et al., 2012 I.	102	21.88	4.29	111	21.89	4.35	baseline	0.00		-0.00	[-0.27; 0.27]
Dirksen, S. et al, 2007 II.	34	19.10	4.00	38	19.10	4.70	baseline	0.00		0.00	[-0.46; 0.46]
Qiu, H, et al., 2018 I.	98	8.71	5.89	196	8.58	5.92	baseline	0.00		0.02	[-0.22; 0.26]
Hoffman, CJ, et al., 2012 II.	102	17.83	5.03	110	17.65	5.83	baseline	0.00		0.03	[-0.24; 0.30]
Fillion, L, et al. 2008	44	40.49	9.50	43	40.00	9.43	baseline	0.00		0.05	[-0.37; 0.47]
Klinkhammer-Schalke, M et al., 2012	100	78.68	26.40	100	76.65	27.16	baseline	0.00		0.08	[-0.20; 0.35]
Klafke, N, et al., 2019	120	85.10	19.30	113	82.60	20.20	baseline	0.00		0.13	[-0.13; 0.38]
Cengiz, HO, et al., 2023	32	14.15	7.56	33	13.03	6.82	baseline	0.00		0.15	[-0.33; 0.64]
Arving, C, et al., 2007	47	81.00	17.00	38	77.00	18.00	baseline	0.00		0.23	[-0.20; 0.66]
Hernandez, EG, et al. 2018 I.	28	18.93	5.70	28	17.57	5.96	baseline	0.00		0.23	[-0.30; 0.76]
Elyasi, F, et al., 2021 IV.	20	11.60	2.90	20	10.50	3.90	baseline	0.00		0.31	[-0.31; 0.94]
Hernandez, EG, et al. 2018 II.	28	15.75	6.10	28	13.82	5.68	baseline	0.00		0.32	[-0.20; 0.85]
Von Ah, D, et al., 2012 II.	27	44.69	4.40	29	43.18	4.72	baseline	0.00		0.33	[-0.20; 0.85]
Elyasi, F, et al., 2021 III.	20	21.00	4.60	20	18.90	6.40	baseline	0.00		0.37	[-0.26; 0.99]
Elyasi, F, et al., 2021 II.	15	11.90	3.20	15	10.50	3.90	baseline	0.00		0.38	[-0.34; 1.10]
Elyasi, F, et al., 2021 I.	15	21.00	1.40	15	18.90	6.40	baseline	0.00		0.44	[-0.28; 1.17]
Von Ah, D, et al., 2012 I.	26	45.71	5.98	29	43.18	4.72	baseline	0.00		0.47	[-0.07; 1.00]
Ferguson, RJ, et al., 2012	19	7.04	1.79	21	7.91	1.10	followup	8.00		-0.58	[-1.22; 0.05]
Heiney, SP, et al., 2003	33	6.40	1.40	33	7.20	2.10	followup	16.00		-0.44	[-0.93; 0.05]
Heiney, SP, et al., 2003	33	6.40	1.80	33	6.90	2.10	followup	6.00		-0.25	[-0.74; 0.23]
Elyasi, F, et al., 2021 III.	20	17.60	2.70	20	18.70	6.20	followup	24.00		-0.23	[-0.85; 0.40]
Klinkhammer-Schalke, M et al., 2012	100	77.91	23.85	100	79.69	20.55	followup	12.00		-0.08	[-0.36; 0.20]
Elyasi, F, et al., 2021 IV.	20	10.50	2.10	20	10.50	3.70	followup	24.00		0.00	[-0.62; 0.62]
Rahmani, S, et al. 2015	12	12.00	69.44	12	12.00	58.33	followup	16.00		0.00	[-0.80; 0.80]
Peng, L, et al., 2022	28	78.33	12.52	29	78.16	10.60	followup	0.10		0.01	[-0.50; 0.53]
Nápoles AM, et al. 2015	76	18.13	4.81	75	18.01	4.44	followup	12.00		0.03	[-0.29; 0.34]
Arving, C, et al., 2007	47	79.00	20.00	38	78.00	17.00	followup	12.00		0.05	[-0.37; 0.48]
Klafke, N, et al., 2019	120	68.70	23.50	113	67.30	22.60	followup	24.00		0.06	[-0.20; 0.32]
Klinkhammer-Schalke, M et al., 2012	100	80.20	20.30	100	78.42	22.07	followup	24.00		0.08	[-0.19; 0.36]
Von Ah, D, et al., 2012 I.	26	45.03	6.17	29	44.49	4.81	followup	8.00		0.10	[-0.43; 0.63]
Von Ah, D, et al., 2012 II.	27	45.00	4.48	29	44.49	4.81	followup	8.00		0.11	[-0.42; 0.63]
Arving, C, et al., 2007	47	80.00	19.00	38	78.00	17.00	followup	4.00		0.11	[-0.32; 0.54]
Peng, L, et al., 2022	28	83.10	12.63	28	81.38	14.52	followup	4.00		0.12	[-0.40; 0.65]
Klinkhammer-Schalke, M et al., 2012	100	83.25	22.34	100	80.20	21.57	followup	48.00		0.14	[-0.14; 0.42]
Dirksen, S. et al, 2007 I.	34	24.80	3.30	38	24.30	3.80	followup	10.00		0.14	[-0.32; 0.60]
Arving, C, et al., 2007	47	82.00	19.00	38	79.00	18.00	followup	24.00		0.16	[-0.27; 0.59]
Klafke, N, et al., 2019	120	80.90	21.60	113	77.00	21.40	followup	48.00		0.18	[-0.08; 0.44]
Qiu, H, et al., 2018 II.	98	9.83	9.73	196	8.01	8.85	followup	4.00		0.20	[-0.04; 0.44]
Qiu, H, et al., 2018 II.	98	14.04	11.82	196	11.52	12.78	followup	24.00		0.20	[-0.04; 0.45]
Fillion, L, et al. 2008	44	46.76	9.24	43	44.64	11.05	followup	12.00		0.21	[-0.21; 0.63]
Klafke, N, et al., 2019	120	75.30	20.20	113	70.70	21.70	followup	12.00		0.22	[-0.04; 0.48]
Nápoles AM, et al. 2015	76	19.44	4.26	75	18.44	4.58	followup	24.00		0.23	[-0.10; 0.55]
Hoffman, CJ, et al., 2012 I.	102	22.86	4.22	111	21.84	4.54	followup	8.00		0.23	[-0.04; 0.50]
Qiu, H, et al., 2018 II.	98	12.99	11.82	196	10.18	11.80	followup	12.00		0.24	[-0.01; 0.48]
Elyasi, F, et al., 2021 I.	15	20.00	3.90	15	18.70	6.20	followup	24.00		0.24	[-0.47; 0.96]
Klinkhammer-Schalke, M et al., 2012	100	84.26	19.79	100	78.68	23.35	followup	36.00		0.26	[-0.02; 0.54]
Dirksen, S. et al, 2007 II.	34	22.70	4.20	38	21.50	4.70	followup	10.00		0.27	[-0.20; 0.73]
Elyasi, F, et al., 2021 II.	15	11.40	2.60	15	10.50	3.70	followup	24.00		0.27	[-0.45; 0.99]
Hoffman, CJ, et al., 2012 I.	102	22.97	4.34	111	21.67	4.87	followup	12.00		0.28	[0.01; 0.55]
Fillion, L, et al. 2008	44	45.10	10.42	43	41.76	9.76	followup	4.00		0.33	[-0.10; 0.75]
Hoffman, CJ, et al., 2012 II.	102	19.45	5.32	110	17.53	5.37	followup	12.00		0.36	[0.09; 0.63]
Hoffman, CJ, et al., 2012 II.	102	19.46	5.27	110	17.41	6.06	followup	8.00		0.36	[0.09; 0.63]
Kim, YH, et al., 2017	30	75.10	14.20	30	68.90	18.10	followup	6.00		0.38	[-0.13; 0.89]
Ruiz-Vozmediano, J, et al.,2020	31	85.00	14.10	32	78.30	18.90	followup	24.00		0.40	[-0.10; 0.89]
Kim, YH, et al., 2017	30	75.60	17.40	30	68.40	18.10	followup	9.00		0.40	[-0.11; 0.91]
Hernandez, EG, et al. 2018 II.	28	17.64	4.61	28	15.56	4.81	followup	24.00		0.44	[-0.10; 0.97]
Qiu, H, et al., 2018 I.	98	12.92	9.73	196	8.01	8.85	followup	4.00		0.54	[0.29; 0.78]
Hernandez, EG, et al. 2018 II.	28	17.54	3.20	28	15.40	4.39	followup	8.00		0.55	[0.02; 1.08]
Qiu, H, et al., 2018 I.	98	16.92	11.12	196	10.18	11.80	followup	12.00		0.58	[0.33; 0.83]
Qiu, H, et al., 2018 I.	98	18.96	10.43	196	11.52	12.78	followup	24.00		0.62	[0.37; 0.86]
Cengiz, HO, et al., 2023	32	16.75	6.33	33	12.33	7.00	followup	8.00		0.65	[0.15; 1.15]
Hernandez, EG, et al. 2018 I.	28	21.50	3.39	28	17.68	6.16	followup	8.00		0.76	[0.21; 1.30]
Hernandez, EG, et al. 2018 I.	28	21.27	4.09	28	17.52	5.34	followup	24.00		0.78	[0.23; 1.32]
Rahmani, S, et al. 2015	12	72.77	3.43	12	66.11	10.81	followup	8.00		0.80	[-0.04; 1.64]
Jelvehzadeh, F, et al., 2022	24	5.64	1.73	24	3.70	1.68	followup	12.00		1.12	[0.51; 1.73]
Jelvehzadeh, F, et al., 2022	24	5.64	1.73	24	3.43	1.75	followup	8.00		1.25	[0.63; 1.87]
Ding, K, et al. 2020	34	104.47	7.43	40	82.38	5.42	followup	4.00		3.40	[2.68; 4.13]
Random effect	4087			4900						0.23	[0.11; 0.36]
Prediction interval											[-0.89; 1.35]
**gastroenterological**											
Cheung YL, et al. 2002	29	15.75	1.75	30	17.50	3.83	baseline	0.00		-0.58	[-1.10; -0.06]
Zhang, LMM, et al., 2021	80	58.64	16.46	80	59.25	16.46	baseline	0.00		-0.04	[-0.35; 0.27]
Qin, X, et al., 2017	50	78.56	8.38	50	78.53	11.59	baseline	0.00		0.00	[-0.39; 0.39]
Baoyindeligeer, L.Z. et al. 2020	65	62.75	13.46	65	62.35	12.28	baseline	0.00		0.03	[-0.31; 0.37]
Zhang, LMM, et al., 2021	80	62.34	16.46	80	62.75	15.02	followup	12.00		-0.03	[-0.34; 0.28]
Zhang, LMM, et al., 2021	80	67.90	15.85	80	64.81	13.17	followup	24.00		0.21	[-0.10; 0.52]
Zhang, LMM, et al., 2021	80	72.01	14.81	80	68.72	12.35	followup	36.00		0.24	[-0.07; 0.55]
Zhang, LMM, et al., 2021	80	74.27	15.02	80	70.16	13.58	followup	48.00		0.29	[-0.03; 0.60]
Qin, X, et al., 2017	50	89.42	12.35	50	80.37	14.32	followup	2.00		0.67	[0.27; 1.07]
Baoyindeligeer, L.Z. et al. 2020	65	84.04	12.72	65	72.65	13.25	followup	2.00		0.87	[0.51; 1.23]
Cheung YL, et al. 2002	29	26.58	1.45	30	22.60	2.14	followup	10.00		2.14	[1.49; 2.79]
Cheung YL, et al. 2002	29	24.96	1.32	30	20.86	1.75	followup	5.00		2.60	[1.90; 3.31]
Random effect	717			720						0.50	[-0.16; 1.15]
Prediction interval											[-1.27; 2.26]
**gynaecological**											
Zhou, L, et al., 2020	37	13.65	1.84	36	14.85	1.47	baseline	0.00		-0.71	[-1.19; -0.24]
Powell, CB, et al., 2008 I.	21	21.60	5.80	43	22.10	7.40	baseline	0.00		-0.07	[-0.59; 0.45]
Chan, et al. 2005	80	65.81	61.89	75	62.85	68.50	baseline	0.00		0.05	[-0.27; 0.36]
Sandsund, C, et al., 2017	72	77.20	20.20	70	75.50	22.30	baseline	0.00		0.08	[-0.25; 0.41]
Powell, CB, et al., 2008 II.	21	18.90	6.20	43	18.00	6.00	baseline	0.00		0.15	[-0.38; 0.67]
Chan, et al. 2005	80	56.52	61.89	75	72.73	75.34	followup	12.00		-0.23	[-0.55; 0.08]
Chan, et al. 2005	80	78.26	99.01	75	92.89	37.67	followup	72.00		-0.19	[-0.51; 0.12]
Chan, et al. 2005	80	67.00	67.17	75	78.06	58.20	followup	24.00		-0.17	[-0.49; 0.14]
Chan, et al. 2005	80	81.23	81.30	75	86.76	65.04	followup	60.00		-0.07	[-0.39; 0.24]
Chan, et al. 2005	80	82.61	67.17	75	86.96	54.73	followup	48.00		-0.07	[-0.39; 0.24]
Chan, et al. 2005	80	83.00	63.59	75	83.60	59.93	followup	36.00		-0.01	[-0.32; 0.31]
Sandsund, C, et al., 2017	72	80.10	21.40	70	79.00	20.00	followup	12.00		0.05	[-0.28; 0.38]
Sandsund, C, et al., 2017	72	81.70	18.10	70	79.20	19.90	followup	24.00		0.13	[-0.20; 0.46]
Powell, CB, et al., 2008 I.	21	25.40	3.30	43	22.60	6.10	followup	12.00		0.52	[-0.01; 1.05]
Powell, CB, et al., 2008 II.	21	22.60	5.80	43	17.40	7.40	followup	12.00		0.74	[0.20; 1.28]
Zhou, L, et al., 2020	37	45.12	5.62	36	38.94	5.19	followup	12.00		1.13	[0.63; 1.63]
Random effect	934			979						0.19	[-0.06; 0.43]
Prediction interval											[-1.31; 1.68]
**prostate**											
Berglund, G. et al. 2007	39	73.30	23.00	150	84.60	18.30	baseline	0.00		-0.58	[-0.94; -0.22]
Parker, PA, et al., 2009 I.	39	51.32	6.74	36	52.29	5.88	baseline	0.00		-0.15	[-0.60; 0.30]
Penedo, FJ, et al., 2020 I.	95	-23.26	3.80	97	-23.09	3.84	baseline	0.00		-0.04	[-0.33; 0.24]
Penedo, FJ, et al., 2020 II.	95	-20.27	4.68	97	-20.48	4.63	baseline	0.00		0.04	[-0.24; 0.33]
Penedo, FJ, et al., 2007	41	-23.98	28.88	30	-25.67	14.84	baseline	0.00		0.07	[-0.40; 0.54]
Parker, PA, et al., 2009 II.	39	52.79	6.12	36	52.29	5.88	baseline	0.00		0.08	[-0.37; 0.54]
Karlsen RV et al., 2021	16	58.60	3.10	19	55.80	5.40	baseline	0.00		0.61	[-0.07; 1.29]
Huri M, et al., 2015	19	36.68	8.73	15	-36.26	7.93	baseline	0.00		8.49	[6.25; 10.73]
Berglund, G. et al. 2007	39	78.20	22.50	150	87.80	16.90	followup	48.00		-0.53	[-0.88; -0.17]
Penedo, FJ, et al., 2007	41	-25.51	13.16	30	-23.83	21.36	followup	12.00		-0.10	[-0.57; 0.37]
Karlsen RV et al., 2021	16	53.20	9.40	19	53.80	6.60	followup	32.00		-0.07	[-0.74; 0.59]
Northhouse, LL, et al., 2007	107	43.30	6.60	121	43.60	6.50	followup	32.00		-0.05	[-0.31; 0.21]
Penedo, FJ, et al., 2020 I.	95	-23.29	4.00	97	-23.11	4.04	followup	48.00		-0.04	[-0.33; 0.24]
Penedo, FJ, et al., 2020 I.	95	-23.16	3.90	97	-22.99	3.94	followup	24.00		-0.04	[-0.33; 0.24]
Northhouse, LL, et al., 2007	112	48.60	6.70	123	48.70	6.50	followup	16.00		-0.02	[-0.27; 0.24]
Northhouse, LL, et al., 2007	104	42.70	6.50	114	42.50	6.40	followup	48.00		0.03	[-0.23; 0.30]
Parker, PA, et al., 2009 I.	38	48.86	8.88	32	48.51	9.50	followup	24.00		0.04	[-0.43; 0.51]
Penedo, FJ, et al., 2020 II.	95	-20.08	4.87	97	-20.29	4.83	followup	48.00		0.04	[-0.24; 0.33]
Penedo, FJ, et al., 2020 II.	95	-20.49	4.78	97	-20.70	4.73	followup	24.00		0.04	[-0.24; 0.33]
Parker, PA, et al., 2009 I.	39	47.28	9.18	36	44.63	9.12	followup	6.00		0.29	[-0.17; 0.74]
Parker, PA, et al., 2009 II.	38	51.36	9.18	32	48.51	9.50	followup	24.00		0.30	[-0.17; 0.78]
Parker, PA, et al., 2009 II.	39	49.58	9.74	36	44.63	9.12	followup	6.00		0.52	[0.06; 0.98]
Parker, PA, et al., 2009 I.	37	50.22	8.46	32	45.12	8.88	followup	48.00		0.58	[0.10; 1.07]
Parker, PA, et al., 2009 II.	37	51.76	10.16	32	45.12	8.88	followup	48.00		0.68	[0.20; 1.17]
Huri M, et al., 2015	19	0.57	5.38	15	-41.73	6.14	followup	12.00		7.21	[5.27; 9.15]
Random effect	1429			1640						0.42	[-0.37; 1.21]
Prediction interval											[-1.06; 1.90]
**Random effect**	**7167**			**8239**						**0.26**	**[0.13; 0.40]**
**Prediction interval**											**[-0.66; 1.19]**

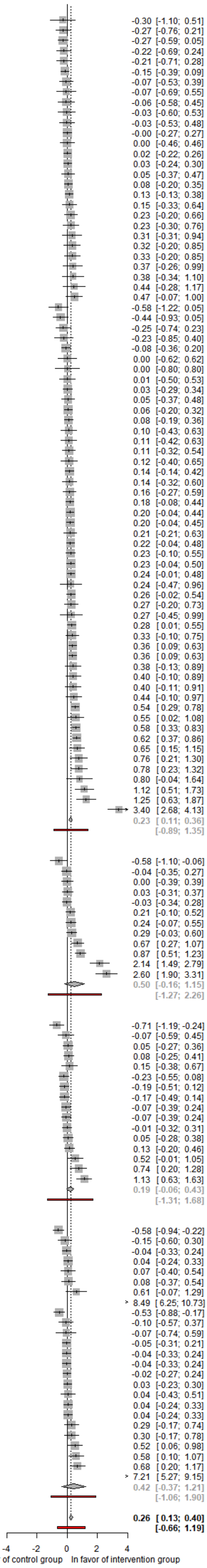

Figure S22.3.Subgroup analysis of the Physical QoL. Forest plot represents the difference between the intervention vs. control group in the Physical QoL domain with the cancer type subgroups as predicted at week 24 (post-intervention). SMD - Standardized mean difference, CI - confidence interval.

Figure S22.4.T48

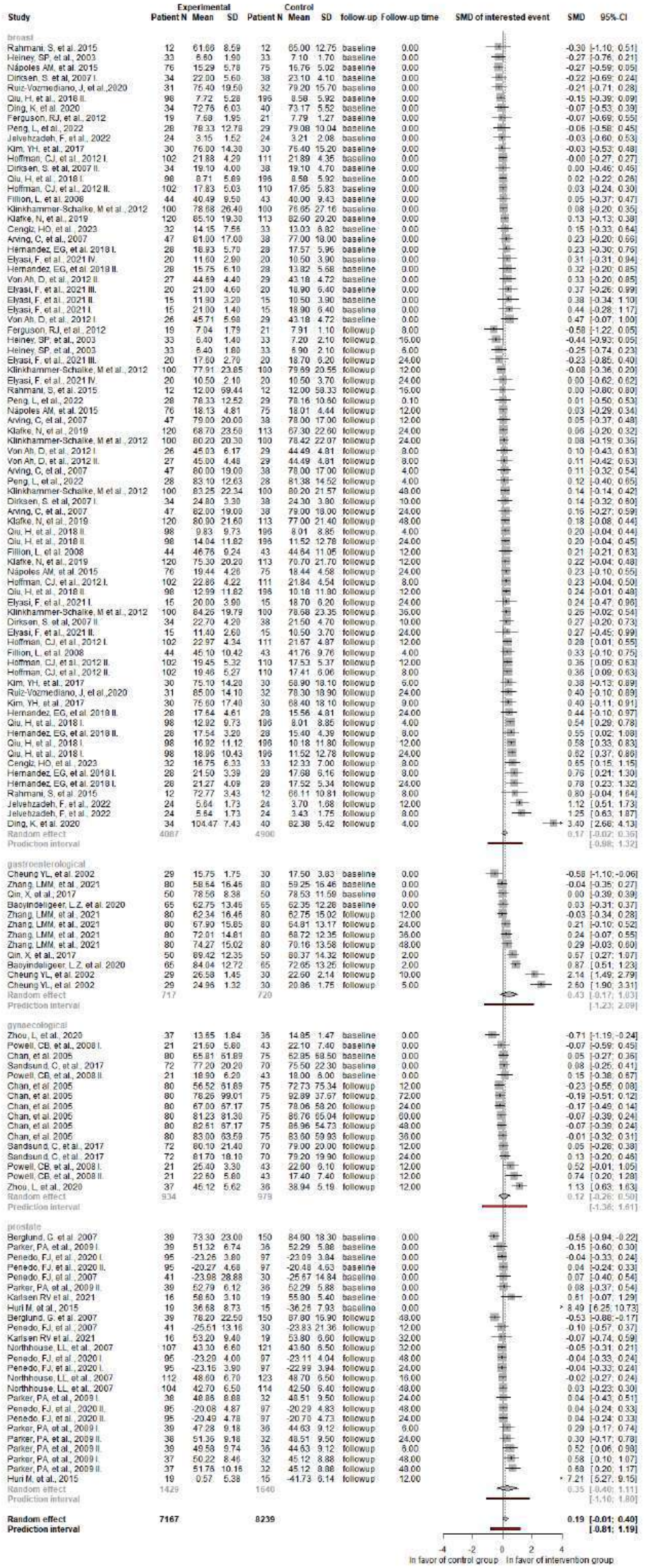

Figure S22.4. Subgroup analysis of the Physical QoL. Forest plot represents the difference between the intervention vs. control group in the Physical QoL domain with the cancer type subgroups as predicted at week 48 (post-intervention). SMD - Standardized mean difference, CI - confidence interval.

S23. Table S.3. Risk of bias assessment

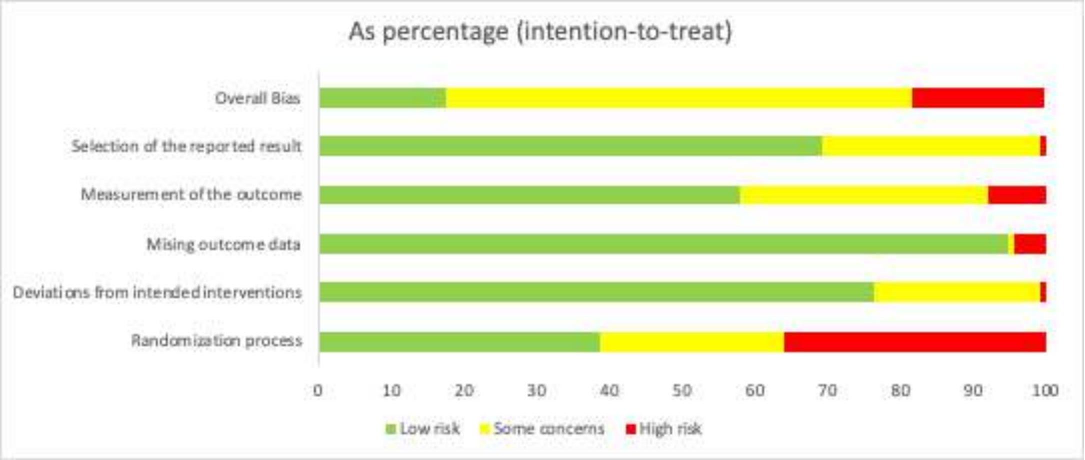

Study ID	Experimental	Comparator	Outcome	Weight	Randomization process	Deviations from intended inter	Missing outcome data	Measurement of the outcome	Selection of the reported result	Overall
Antoni et al. 2006	psychological inte	usual care	quality of life	1	+	+	+	+	?	!
Aranda et al. 2006	psychological inte	usual care	quality of life	1	?	+	+	+	?	+
Armes et al. 2007	psychological inte	usual care	quality of life	1	+	+	+	+	?	!
Arving et al. 2007	psychological inte	usual care	quality of life	1	?	+	+	+	+	!
Ashing et al. 2019	psychological inte	usual care	quality of life	1	+	+	+	+	?	!
Baoyindeligeer et al.	psychological inte	usual care	quality of life	1	?	?	+	+	?	!
Beatty et al. 2015	psychological inte	usual care	quality of life	1	+	?	+	+	?	!
Beatty et al. 2015	psychological inte	usual care	quality of life	1	?	+	+	+	+	!
Berglund et al. 2007	psychological inte	usual care	quality of life	1	−	+	+	+	?	−
Boele et al. 2017	psychological inte	usual care	quality of life	1	−	+	+	+	+	−
Boesen et al.2007	psychological inte	usual care	recurrence free sur	1	+	+	+	+	?	!
Braeken et al. 2013	psychological inte	usual care	quality of life	1	?	+	+	?	+	!
Breitbart et al. 2018	psychological inte	usual care	quality of life	1	+	+	+	−	+	−
Chan et al. 2005	psychological inte	usual care	quality of life	1	+	+	+	+	?	!
Chen et al. 2017	psychological inte	usual care	quality of life	1	+	+	+	+	?	!
Cheung et al. 2002	psychological inte	usual care	quality of life	1	?	+	+	−	?	−
Chu et al. 2020	psychological inte	usual care	quality of life	1	+	?	?	?	+	!
Compen et al. 2018	psychological inte	usual care	quality of life	1	+	+	+	+	+	+
Dieng et al. 2020	psychological inte	usual care	quality of life	1	+	+	+	+	?	!
Ding et al. 2020	psychological inte	usual care	quality of life	1	+	+	+	?	?	!
Dirksen et al. 2007	psychological inte	usual care	quality of life	1	−	+	+	+	?	−
Dolbeault et al. 200E	psychological inte	usual care	quality of life	1	?	+	+	+	?	!
Dos santos et al.202(	psychological inte	usual care	quality of life	1	+	+	+	+	+	+
Edmonds et al.1999	psychological inte	usual care	quality of life	1	−	−	+	?	+	!
Elyasi et al. 2021	psychological inte	usual care	quality of life	1	−	?	+	?	?	!
Esplen et al. 2018	psychological inte	usual care	quality of life	1	?	+	+	+	+	!
Fang et al. 2020	psychological inte	usual care	quality of life	1	?	?	+	?	+	!
Fann et al. 2009	psychological inte	usual care	quality of life	1	?	+	+	+	?	!
Ferguson et al. 2012	psychological inte	usual care	quality of life	1	−	+	+	?	+	!
Fillion et al. 2008	psychological inte	usual care	quality of life	1	+	+	+	+	?	!
Gao et al. 2020	psychological inte	usual care	quality of life	1	+	+	+	+	?	!
Garssen et al. 2012	psychological inte	usual care	quality of life	1	?	?	+	+	?	!
Gaston-Johansson et	psychological inte	usual care	quality of life	1	−	+	+	+	?	−
Giesler et al. 2005	psychological inte	usual care	quality of life	1	+	+	+	+	+	+
Girgis et al. 2009	psychological inte	usual care	quality of life	1	−	+	+	−	+	−
Guan et al. 2019	psychological inte	usual care	quality of life	1	−	+	+	?	+	!
Guan et al. 2019	psychological inte	usual care	recurrence free sur	1	−	+	+	+	+	!
Guo et al. 2013	psychological inte	usual care	quality of life	1	−	+	+	?	+	!
Guo et al. 2013	psychological inte	usual care	overall survival	1	−	+	+	+	+	!
Guo et al. 2013	psychological inte	usual care	recurrence free sur	1	−	+	+	+	+	!
Hall et al. 2011	psychological inte	usual care	quality of life	1	+	+	+	+	+	+
Ham et al. 2019	psychological inte	usual care	quality of life	1	+	+	+	+	?	!
Hauffman et al. 2020	psychological inte	usual care	quality of life	1	−	+	−	−	?	−
Hawkes et al. 2014	psychological inte	usual care	quality of lfe	1	+	+	+	+	+	+

+ Low risk
? Some concerns
− High risk

Heiney et al. 2003	psychological inte	usual care	quality of life	1	?	+	+	–	+	–
Henderson et al. 201	psychological outc	usual care	quality of life	1	–	+	+	?	?	–
Hoffman et al. 2012	psychological inte	usual care	quality of life	1	–	+	+	–	+	–
Hun et al. 2015	psychological inte	ususal care	quality of life	1	+	+	+	+	+	+
Jelvehzadeh et al. 20	psychological inte	usual care	quality of life	1	–	+	+	?	+	!
Jelvehzadeh et al. 20	psychological inte	usual care	quality of life	1	–	+	+	?	+	–
Johansson et al. 200	psychological inte	usual care	quality of life	1	–	+	+	?	+	–
Karlsen et al. 2021	psychological inte	usual care	quality of life	1	+	+	+	+	+	+
Kim et al. 2017	psychological inte	usual care	quality of life	1	–	+	+	?	+	!
Klafke et al. 2019	psychological inte	usual care	quality of life	1	?	+	+	?	+	!
Klinkhammer-Schalk	psychological inte	usual care	quality of life	1	+	+	+	+	+	+
Lee et al. 2018	psychologiacal int	usual care	quality of life	1	?	+	+	?	+	!
Li et al. 2019	psychological inte	usual care	quality of life	1	–	+	+	?	?	+
Liu et al. 2019	psychological inte	usual care	quality of life	1	+	+	+	+	+	+
Lutgendorf et al. 201	psychological care	usual care	quality of life	1	–	+	+	+	+	!
Marchioro et al. 199	psychological inte	usual care	quality of life§	1	?	+	+	+	+	!
McCaughan et al. 20	psychological inte	usual care	quality of life	1	+	+	+	?	+	!
McLachlan et al. 200	psychological inte	usual care	quality of life	1	?	+	+	?	+	!
Miaskowski et al. 20	psychological care	usual care	quality of life	1	+	+	+	–	–	–
Mihuta et al. 2018	psychological inte	usual care	quality of life	1	?	?	+	?	+	!
Nápoles et al. 2015	psychological inte	usual care	quality of life	1	+	+	+	+	+	+
Nikbakhsh et al. 201	psychological inte	usual care	quality of life	1	?	?	+	?	?	!
Northouse et al. 200	psychological inte	usual care	quality of life	1	–	+	+	+	+	!
Parker et al. 2009	psychological inte	usual care	quality of life	1	+	?	+	+	?	!
Penedo et al. 2007	psychological inte	usual care	qualty of life	1	+	+	+	+	?	!
Penedo et al. 2020	psychological inte	usual care	quality of life	1	+	+	+	+	+	+
Peoples et al. 2017	pcychological inte	usual care	quality of life	1	+	+	+	+	+	+
Petersen et al. 2002	psychological inte	usual care	quality of life	1	+	?	+	+	+	!
Powell et al. 2008	psychological inte	usual care	quality of life	1	–	?	+	?	+	!
Rahmani et al. 2015	psychological inte	usual care	quality of life	1	–	+	+	?	+	!
Reese et al. 2021	psychological inte	usual care	quality of life	1	–	?	+	?	+	!
Reich et al. 2016	psychological inte	usual care	quality of life	1	–	+	+	?	+	!
Rodin et al. 2019	psychological inte	usual care	quality of life	1	+	?	+	?	+	!
Rodriguez et al. 201	psychological inte	usual care	quality of life	1	?	?	+	+	?	!
Rosen et al. 2018	psychological inte	usual care	quality of life	1	–	?	+	+	+	!
Ruiz-Vozmediano et	psychological inte	usual care	quality of life	1	–	+	+	+	+	!
Sandsund et al. 2016	psichological inter	usual care	quality of life	1	+	?	+	+	+	!
Savard et al. 2006	psychological inte	usual care	quality of life	1	–	+	+	?	+	–
Schellekens et al. 20	psychological inte	usual care	quality of life	1	–	+	+	?	+	!
Schofield et al. 2016	psychological inte	usual care	quality of life	1	–	+	+	?	+	!
Serfaty et al. 2015	psychological inte	usual care	quality of life	1	–	+	+	+	+	!
Takano et al. 2021	psychological inte	usual cate	quality of life	1	?	+	+	+	+	!
Takano et al. 2021	psychological inte	usual care	overall survival	1	?	+	+	+	+	!
Thomas et al. 2012	psychological inte	usual care	quality of life	1	+	+	+	+	+	+
Trask et al. 2003	psychological inte	usual care	quality of life	1	+	+	+	?	+	!
Turner et al. 2016	psychological inte	usual care	quality of life	1	–	?	+	?	+	!
van de Wal et al. 201	psychological inte	usual care	quality of life	1	+	+	+	+	+	+
van den Berg et al. 20	psychological inte	usual care	quality of life	1	–	+	+	+	+	–
Van Der Hout et al. 2	psychological inte	usual care	quality of life	1	+	+	+	+	?	!
Van der Meulen et al	psychological inte	usual care	quality of life	1	?	+	+	?	+	!

Vanbutsele et al. 201	psychological inte	usual care	overall survival	1	?	+	−	+	+	−
Vanbutsele et al. 201	psychological inte	usual care	quality of life	1	?	+	−	+	+	−
Von Ah et al. 2012	psychological inte	usual care	quality of life	1	−	?	+	+	+	!
Walczak et al. 2017	psychological inte	usual care	quality of life	1	+	+	+	+	+	+
Walker et al. 1999	psychological inte	usual care	quality of life	1	?	+	+	+	+	!
Walker et al. 2014	psychological inte	usual care	quality of life	1	+	+	+	+	+	+
Wang et al. 2019	psychological inte	usual care	quality of life	1	?	+	+	?	+	!
Wang et al. 2019	psychological inte	usual care	overall survival	1	?	+	−	?	+	−
Willems et al. 2016	psychological inte	usual care	quality of life	1	−	?	+	?	+	!
Wu et al. 2016	psychological inte	usual care	quality of life	1	?	+	−	−	?	−
Wu et al. 2021	psychological inte	usual care	quality of life	1	−	?	+	?	+	!
Yoo et al. 2005	psychological inte	usual care	quality of life	1	?	+	+	+	+	!
Yun et al. 2017	psychological inte	usual care	quality of life	1	−	?	+	?	?	!
Zhang et al. 2021	psychological inte	usual care	quality of life	1	+	?	+	+	+	!
Zhang et al. 2021	psychological inte	usual care	overall survival	1	+	?	+	+	+	!
Zhang et al. 2021	psychological inte	usual care	quality of life	1	+	+	+	+	+	+
Zhang et al. 2021	psychological inte	usual care	recurrence free sur	1	+	+	+	+	+	+
Zhao et al. 2021	psychological inte	usual care	quality of life	1	−	?	+	?	?	!
Zhao et al., 2015	psychological inte	usual care	quality of life	1	−	?	+	?	?	!
Zhou et al. 2020	psychological inte	usual care	quality of life	1	+	?	+	−	+	−
Anderson et al. 2006	psychological inte	usual care	quality of life	1	?	+	+	+	?	!
Björneklett et al. 20	psychological inte	usual care	quality of life	1	+	+	+	+	?	!
Børøsund et al. 2020	psychological inte	usual care	quality of life	1	+	+	+	+	+	+
Carbajal-López et al.	psychological inte	usual care	quality of life	1	+	?	+	?	?	!
Rahmani et al. 2014	psychological inte	usual care	quality of life	1	−	+	+	?	+	!
Carlson et al. 2013	psychological inte	usual care	quality of life	1	+	+	+	+	+	+
El et al. 2008	psychological inte	usual care	quality of life	1	−	?	+	?	+	!
Lapid et al. 2007	psychological inte	usual care	quality of life	1	?	+	+	+	+	!
Lu et al. 2017	psychological inte	usual care	quality of life	1	−	+	+	?	+	+
Murphy et al. 2019	psychological inte	usual care	quality of life	1	−	+	+	?	+	!
Pettiford et al. 2017	psychological inte	usual care	quality of life	1	−	?	+	+	?	!
Poort et al. 2020	psychological inte	usual care	quality of life	1	+	+	+	+	+	+
Ramirez et al. 2019	psychological inte	usual care	quality of life	1	+	+	+	+	?	!
Rottmann et al. 201	psychological inte	usual care	quality of life	1	−	+	+	+	+	−
Sharpe et al. 2014	psychological inte	usual care	quality of life	1	−	+	+	?	+	!
Sohl et al. 2017	psychological inte	usual care	quality of life	1	−	+	+	?	+	!
Van Amstel et al. 201	psychological inte	usual care	quality of life	1	−	?	+	?	+	!
Yanez et al. 2015	psychological inte	usual care	quality of life	1	+	?	+	?	+	!

S24. Publication bias

Figure S24.1.Overall Survival

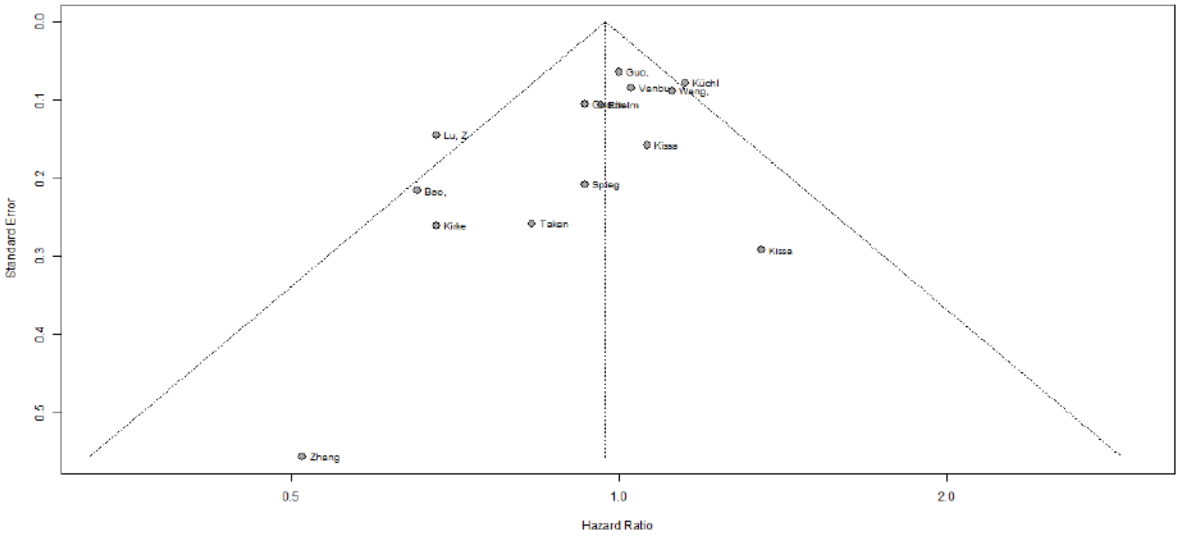

Figure S24.1. Funnel plot for OS outcome showing possible publication bias (Egger's test p-value is 0.0524).

Figure S4.2. Recurrence-free Survival

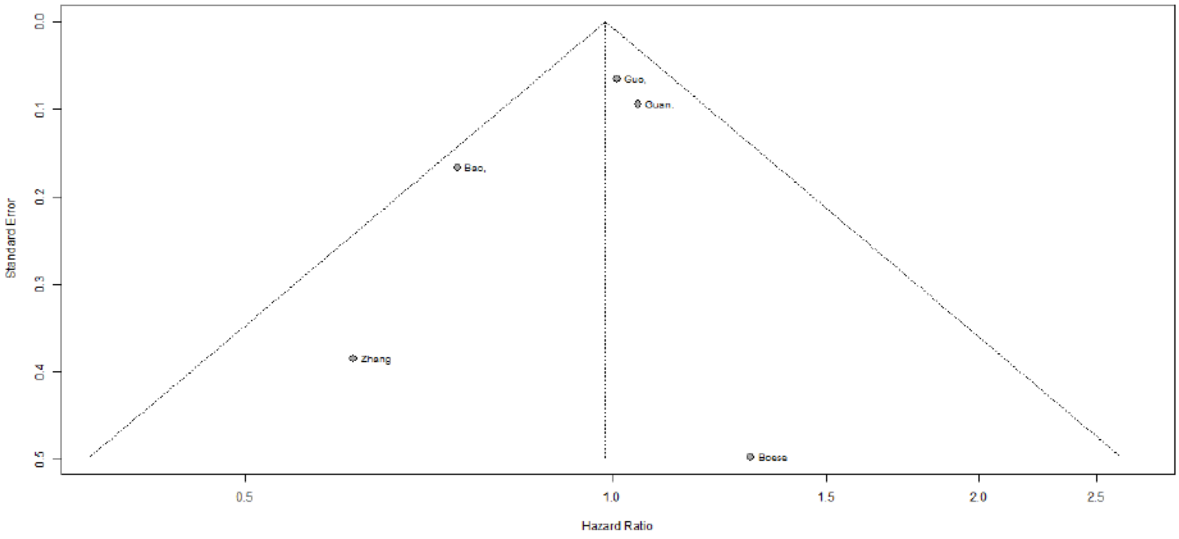

Figure S24.2.Funnel plot for RFS outcome showing no publication bias (Egger's test p-value is 0.4226).

S24.3.Global Qol

Figure S24.3.1.Provider

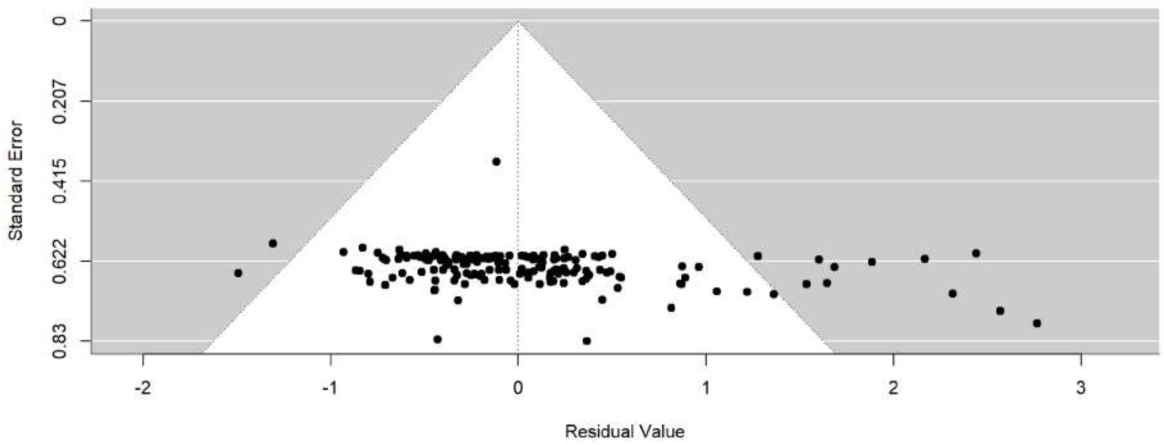

Figure S24.3.1.Funnel plot for Global QoL provider subgroup showing possible publication bias.

Figure S24.3.2.Environment

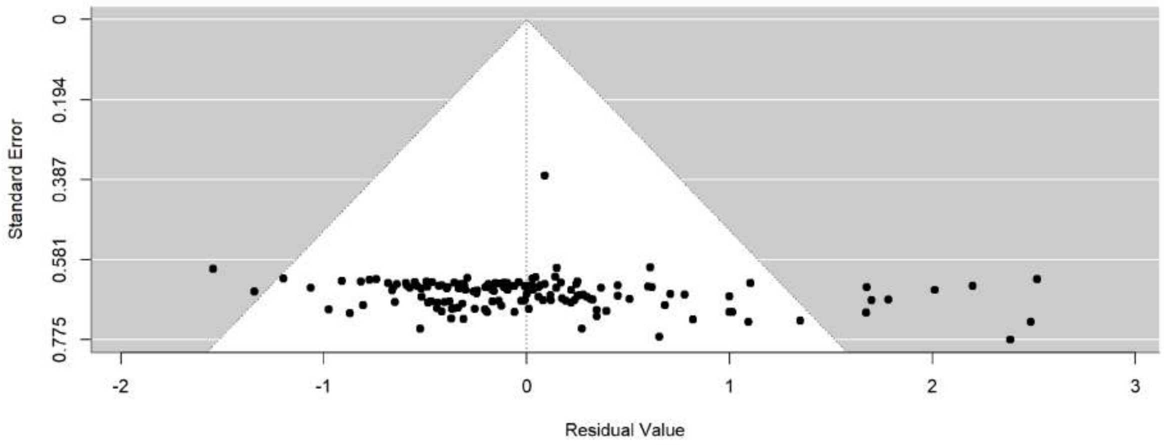

Figure S24.2 .Funnel plot for Global QoL environment subgroup showing possible publication bias.

Figure S24.3.3.Type

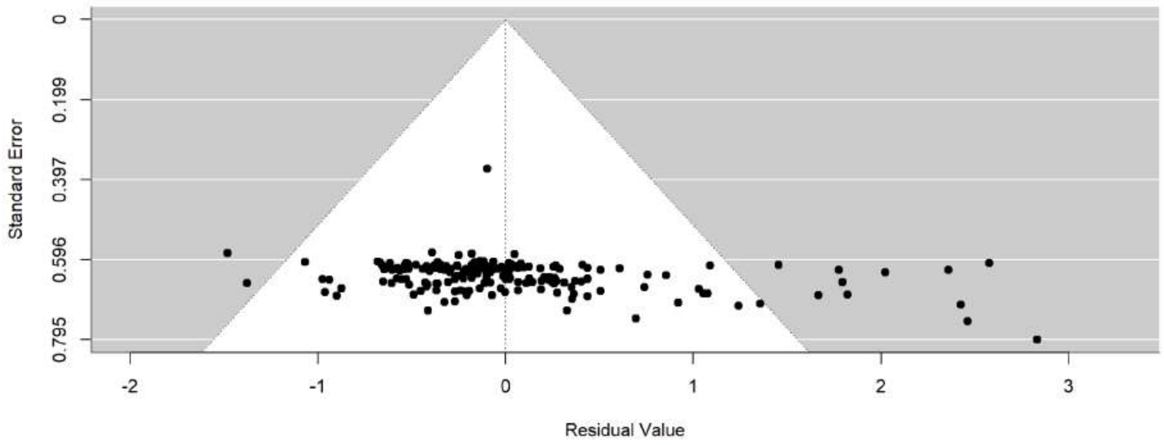

Figure S24.3.3.Funnel plot for Global QoL type subgroup showing possible publication bias.

Figure S24.3.4.Duration of Intervention

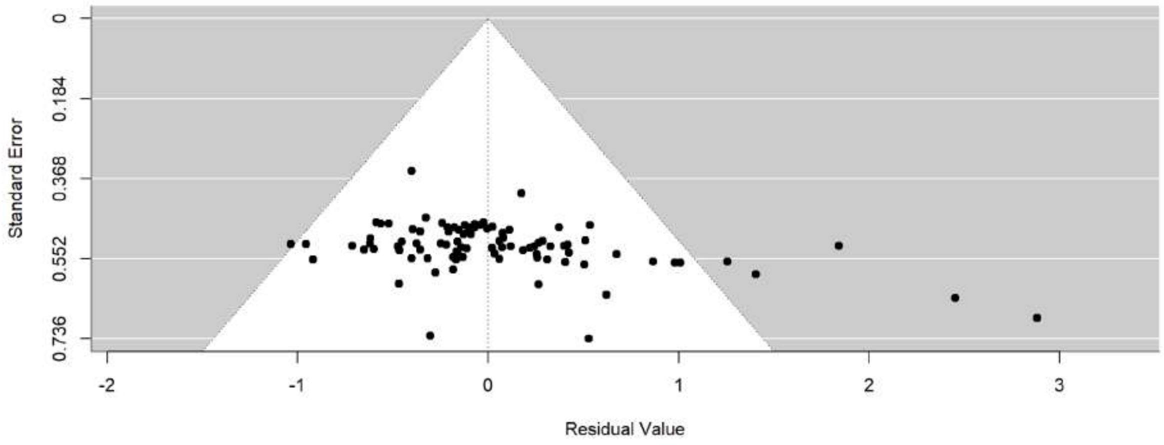

Figure S23.3.4. Funnel plot for Global QoL intervention duration subgroup showing possible publication bias.

Figure S24.3.5.Cancer Stage

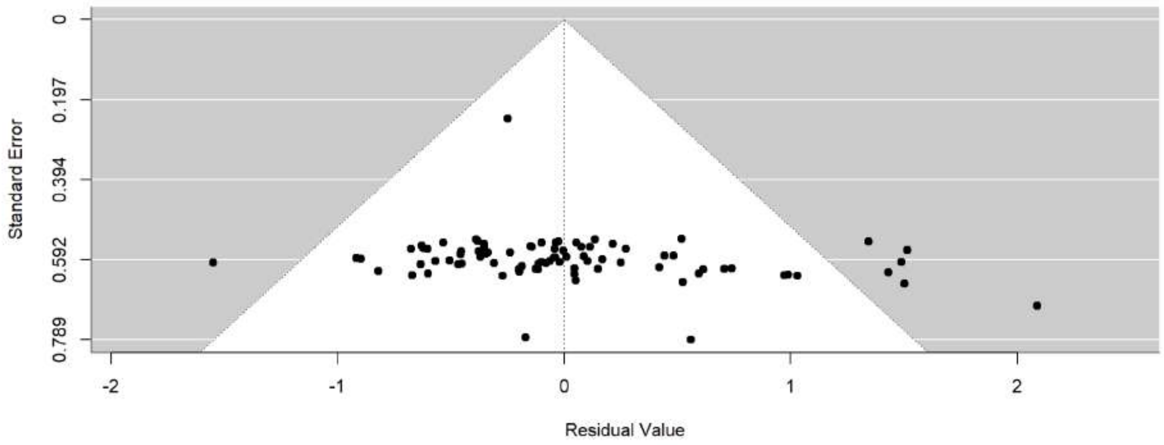

Figure S24.3.5. Funnel plot for Global QoL cancer stage subgroup showing possible publication bias.

Figure S24.3.6.Cancer Type

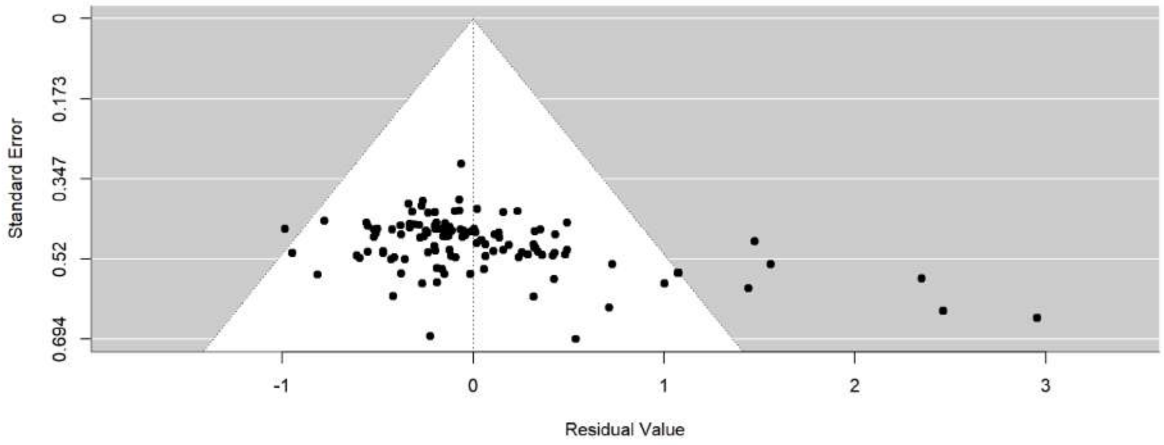

Figure S24.3.6. Funnel plot for Global QoL cancer type subgroup showing possible publication bias.

S24.4.Emotional Qol

Figure S4.4.1.Provider

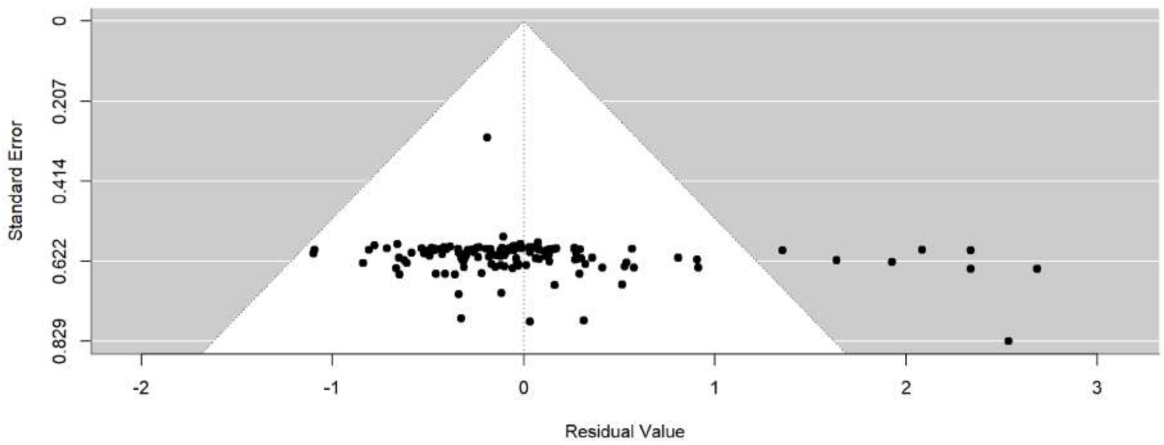

Figure S24.4.1. Funnel plot for Emotional QoL provider subgroup showing possible publication bias.

Figure S24.4.2.Environment

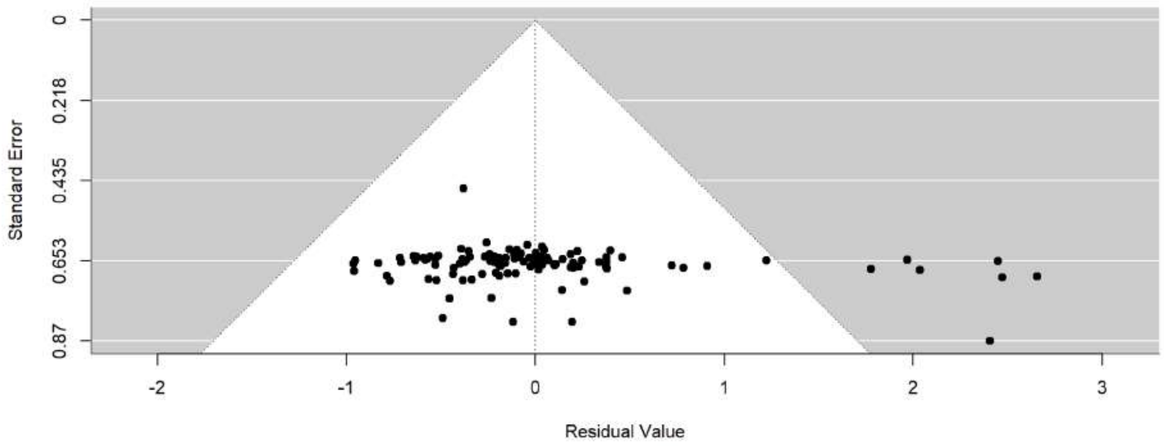

Figure S24.4.2. Funnel plot for Emotional QoL environment subgroup showing possible publication bias.

Figure S24.4.3.Type

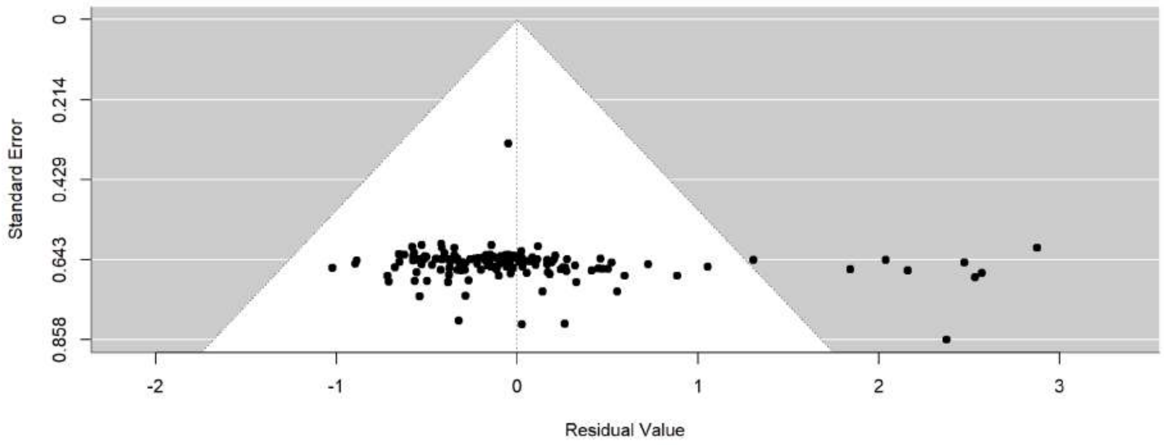

Figure S24.4.3. Funnel plot for Emotional QoL type subgroup showing possible publication bias.

Figure S24.4.4.Duration of Intervention

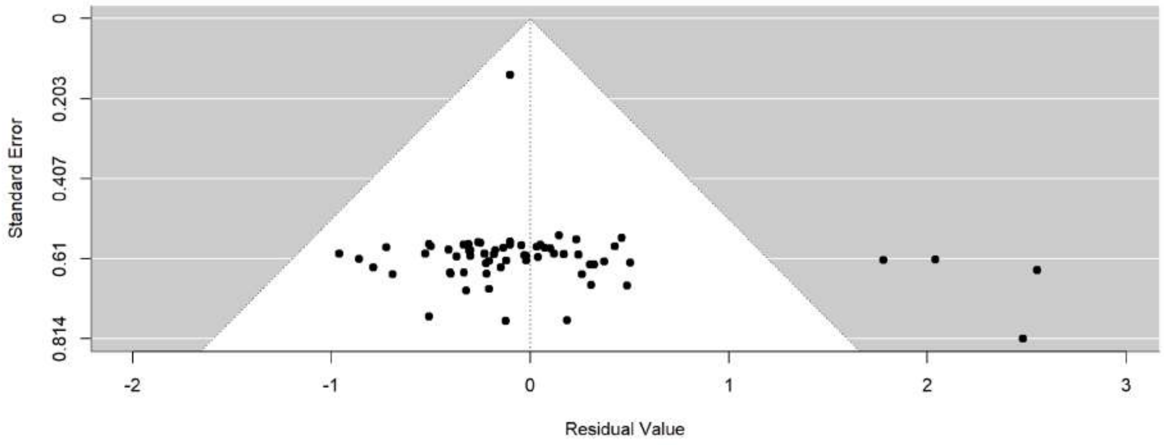

Figure S24.4.4. Funnel plot for Emotional QoL duration subgroup showing possible publication bias.

Figure S24.4.5.Cancer Stage

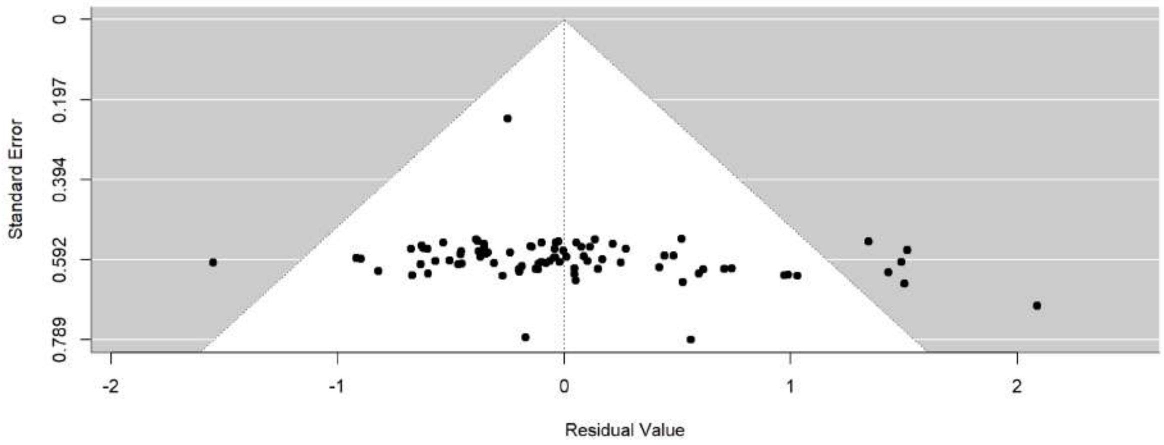

Figure S24.4.5. Funnel plot for Emotional QoL cancer stage subgroup showing possible publication bias.

Figure S24.4.6.Cancer Type

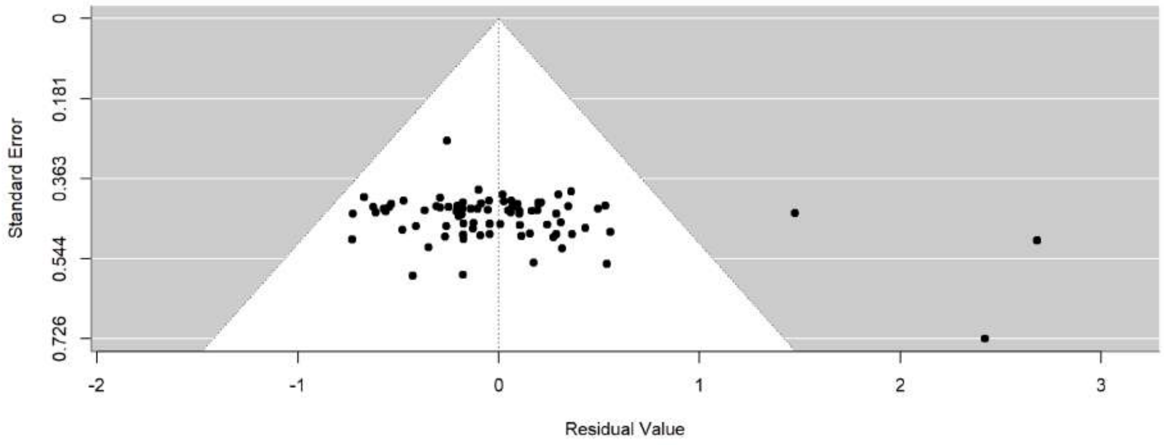

Figure S24.4.6. Funnel plot for Emotional QoL cancer type subgroup showing possible publication bias.

S24.5.Social Qol

Figure S24.5.1.Provider

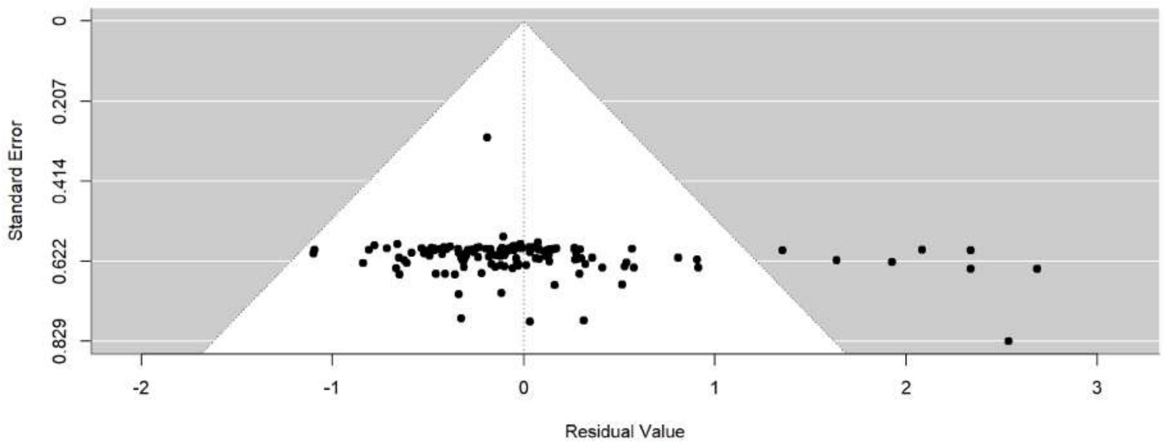

Figure S24.5.1. Funnel plot for Social QoL provider subgroup showing possible publication bias

Figure S24.5.2.Environment

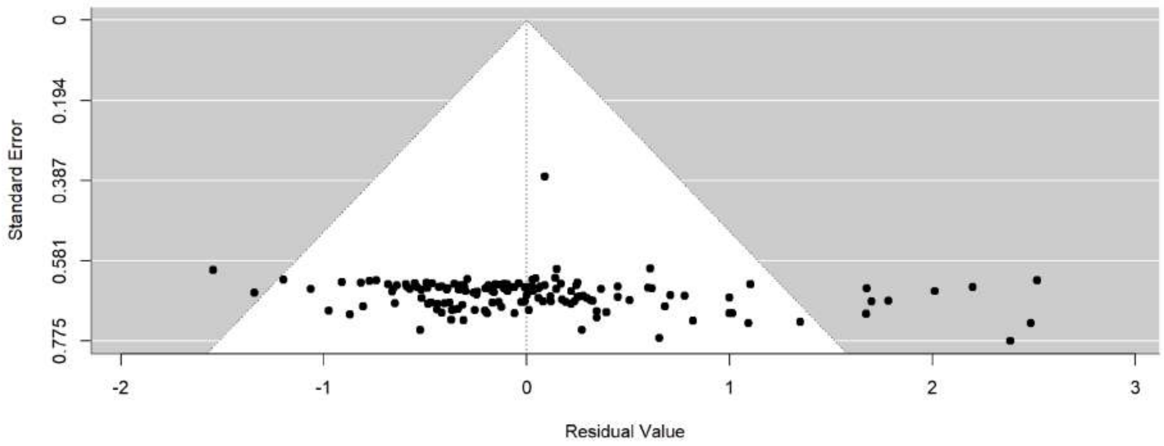

Figure S4.5.2. Funnel plot for Social QoLenvironment subgroup showing possible publication bias

Figure S24.5.3.Type

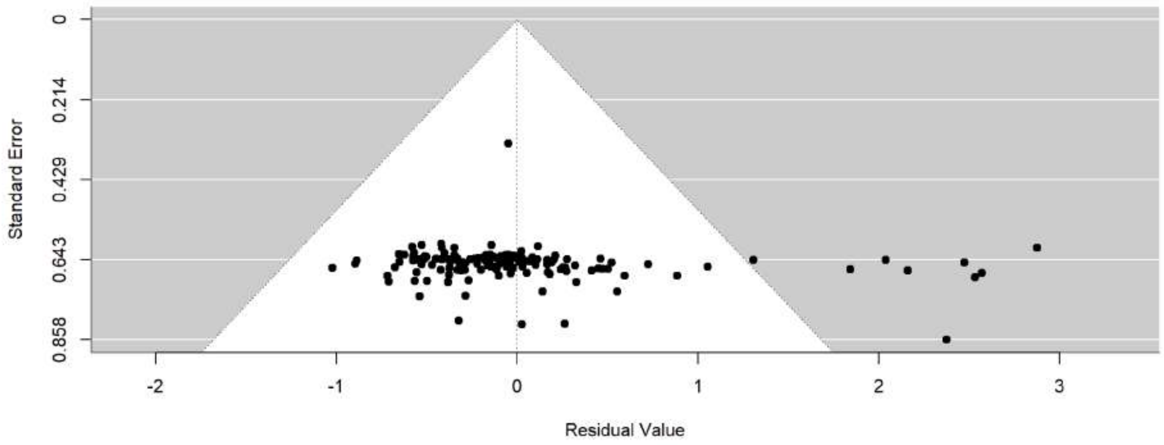

Figure S24.5.3.Funnel plot for Social QoL type subgroup showing possible publication bias

Figure S24.5.4. Duration of Intervention

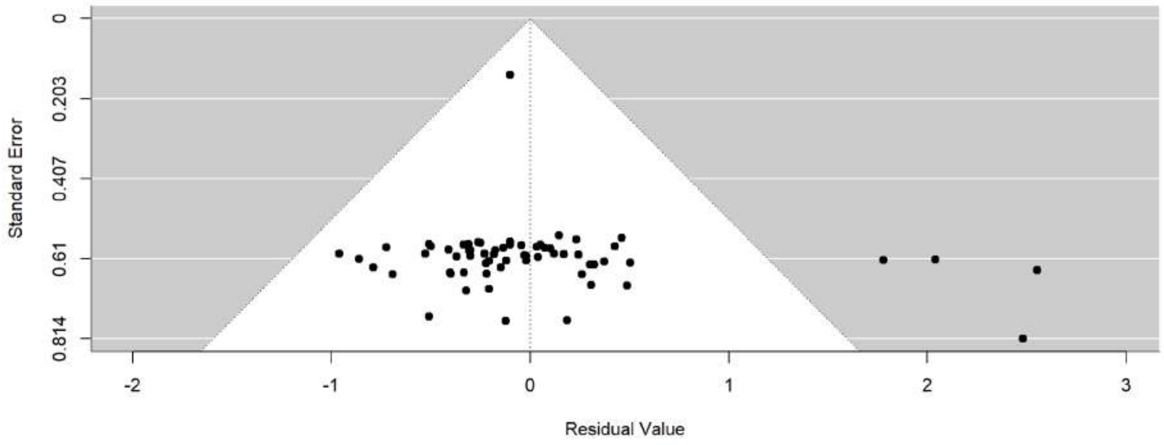

Figure S24.5.4. Funnel plot for Social QoL duration subgroup showing possible publication bias

Figure S24.5.5.Cancer Stage

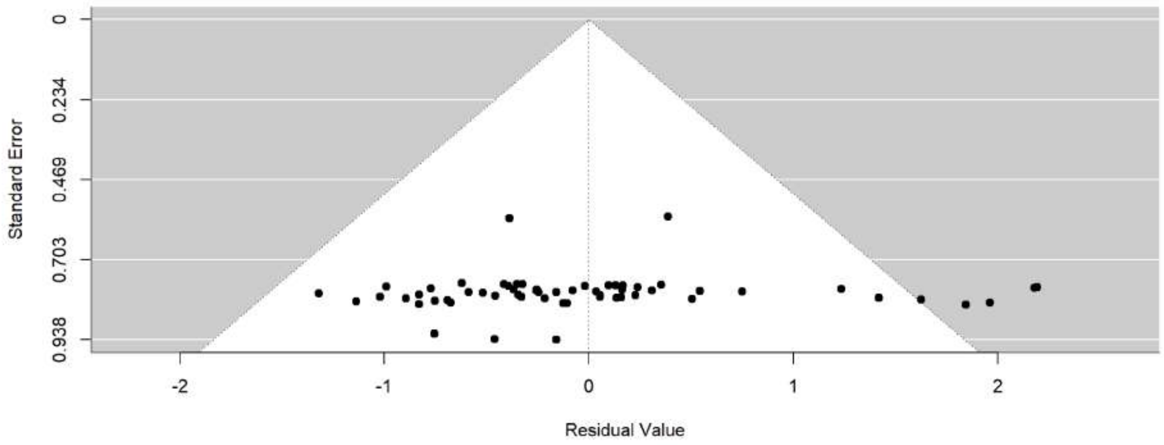

Figure S24.5.5. Funnel plot for Social QoL cancer stage subgroup showing possible publication bias

Figure S24.5.6.Cancer Type

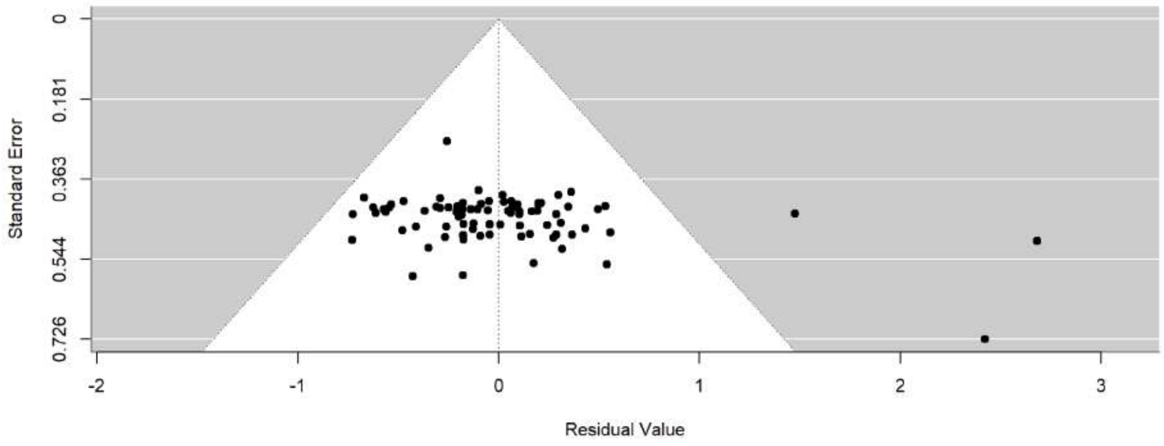

Figure S24.5.6. Funnel plot for Social QoL cancer type subgroup showing possible publication bias

S24.6. Physical Qol

Figure S24.6.1. Provider

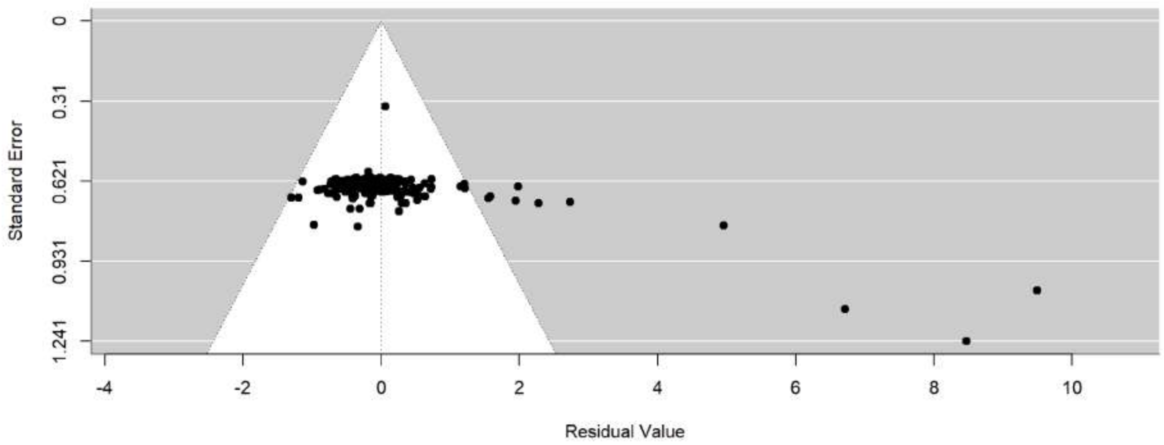

Figure S24.6.1. Funnel plot for Physical QoL provider subgroup showing possible publication bias

Figure S24.6.2. Environment

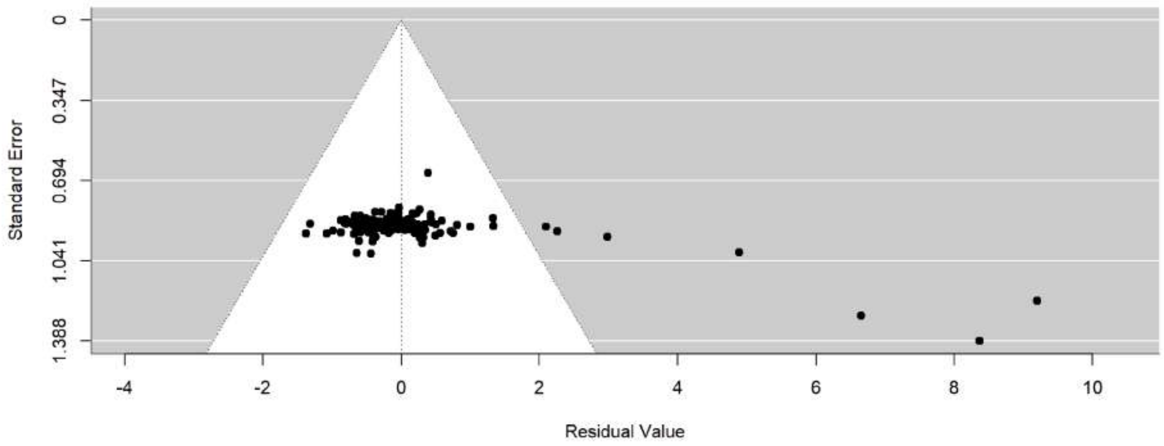

Figure S24.6.2. Funnel plot for Physical QoLenvironment subgroup showing possible publication bias

Figure S24.6.3.Type

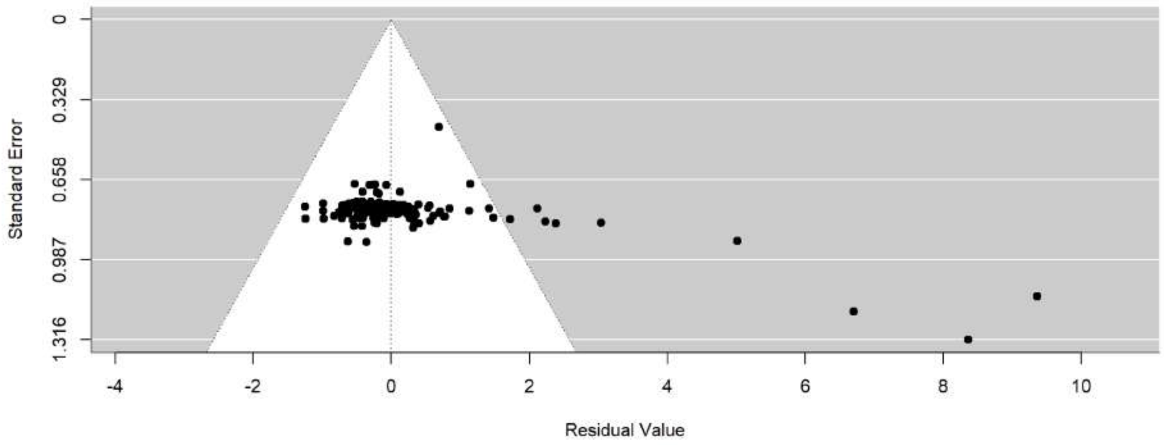

Figure S24.6.3. Funnel plot for Physical QoL type subgroup showing possible publication bias

Figure S24.6.4. Duration of Intervention

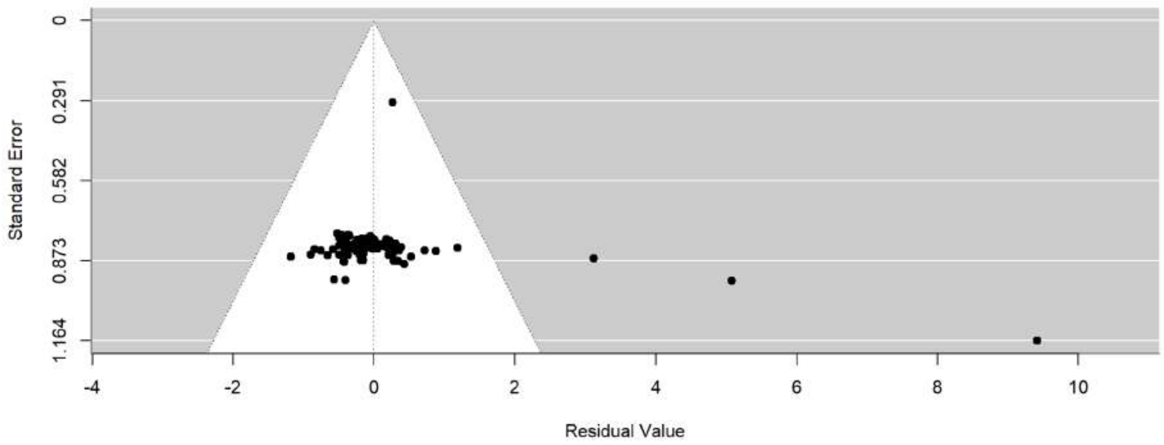

Figure S24.6.4. Funnel plot for Physical QoL duration subgroup showing possible publication bias

Figure S24.6.5. Cancer Stage

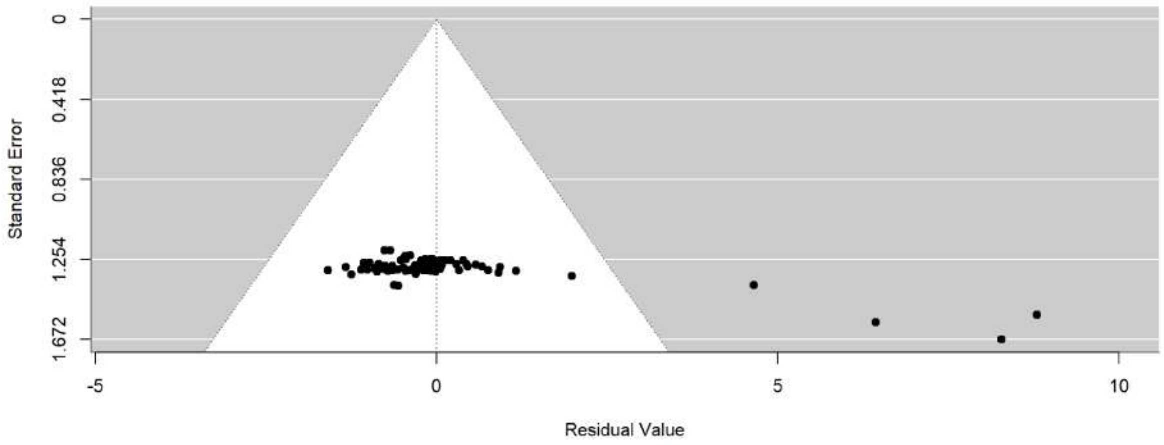

Figure S24.6.5. Funnel plot for Physical QoL cancer stage subgroup showing possible publication bias

Figure S24.6.6. Cancer Type

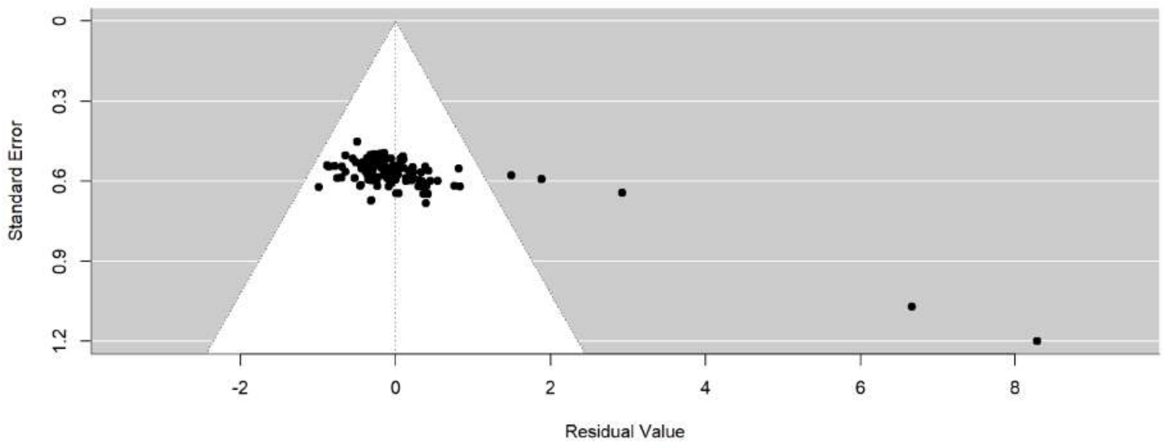

Figure S24.6.6 Funnel plot for Type QoL cancer stage subgroup showing possible publication bias

REFERENCES

1 Huri, M., Huri, E., Kayihan, H. & Altuntas, O. Effects of occupational therapy on quality of life of patients with metastatic prostate cancer. A randomized controlled study. *Saudi Med J* **36**, 954-961 (2015). https://doi.org:10.15537/smj.2015.8.11461

2 Giesler, R. B. *et al.* Improving the quality of life of patients with prostate carcinoma: a randomized trial testing the efficacy of a nurse-driven intervention. *Cancer* **104**, 752-762 (2005). https://doi.org:10.1002/cncr.21231

3 Wang, J., Yan, C. & Fu, A. A randomized clinical trial of comprehensive education and care program compared to basic care for reducing anxiety and depression and improving quality of life and survival in patients with hepatocellular carcinoma who underwent surgery. *Medicine (Baltimore)* **98**, e17552 (2019). https://doi.org:10.1097/MD.0000000000017552

4 Trask, P. C., Paterson, A. G., Griffith, K. A., Riba, M. B. & Schwartz, J. L. Cognitive-behavioral intervention for distress in patients with melanoma: comparison with standard medical care and impact on quality of life. *Cancer* **98**, 854-864 (2003). https://doi.org:10.1002/cncr.11579

5 Takano, T. *et al.* Effectiveness of self-help workbook intervention on quality of life in cancer patients receiving chemotherapy: results of a randomized controlled trial. *BMC Cancer* **21**, 588 (2021). https://doi.org:10.1186/s12885-021-08333-2

6 McCaughan, E., Curran, C., Northouse, L. & Parahoo, K. Evaluating a psychosocial intervention for men with prostate cancer and their partners: Outcomes and lessons learned from a randomized controlled trial. *Appl Nurs Res* **40**, 143-151 (2018). https://doi.org:10.1016/j.apnr.2018.01.008

7 Walker, J. *et al.* Integrated collaborative care for major depression comorbid with a poor prognosis cancer (SMaRT Oncology-3): a multicentre randomised controlled trial in patients with lung cancer. *Lancet Oncol* **15**, 1168-1176 (2014). https://doi.org:10.1016/S1470-2045(14)70343-2

8 Zhang, L. *et al.* Reminiscence therapy exhibits alleviation of anxiety and improvement of life quality in postoperative gastric cancer patients: A randomized, controlled study. *Medicine* **100**, e26821 (2021). https://doi.org:10.1097/md.0000000000026821

9 Walker, L. G. *et al.* Psychological, clinical and pathological effects of relaxation training and guided imagery during primary chemotherapy. *Br J Cancer* **80**, 262-268 (1999). https://doi.org:10.1038/sj.bjc.6690349

10 Arving, C. *et al.* Individual psychosocial support for breast cancer patients: a randomized study of nurse versus psychologist interventions and standard care. *Cancer Nurs* **30**, E10-19 (2007). https://doi.org:10.1097/01.NCC.0000270709.64790.05

11 Liu, T. *et al.* Mindfulness-based stress reduction in patients with differentiated thyroid cancer receiving radioactive iodine therapy: a randomized controlled trial. *Cancer Manag Res* **11**, 467-474 (2019). https://doi.org:10.2147/CMAR.S183299

12 Lutgendorf, S. K. *et al.* Preservation of immune function in cervical cancer patients during chemoradiation using a novel integrative approach. *Brain Behav Immun* **24**, 1231-1240 (2010). https://doi.org:10.1016/j.bbi.2010.06.014
13 Miaskowski, C. *et al.* The use of a responder analysis to identify differences in patient outcomes following a self-care intervention to improve cancer pain management. *Pain* **129**, 55-63 (2007). https://doi.org:10.1016/j.pain.2006.09.031
14 Lee, Y. H., Lai, G. M., Lee, D. C., Tsai Lai, L. J. & Chang, Y. P. Promoting Physical and Psychological Rehabilitation Activities and Evaluating Potential Links Among Cancer-Related Fatigue, Fear of Recurrence, Quality of Life, and Physiological Indicators in Cancer Survivors. *Integr Cancer Ther* **17**, 1183-1194 (2018). https://doi.org:10.1177/1534735418805149
15 Hawkes, A. L., Pakenham, K. I., Chambers, S. K., Patrao, T. A. & Courneya, K. S. Effects of a multiple health behavior change intervention for colorectal cancer survivors on psychosocial outcomes and quality of life: a randomized controlled trial. *Ann Behav Med* **48**, 359-370 (2014). https://doi.org:10.1007/s12160-014-9610-2
16 Yoo, H. J., Ahn, S. H., Kim, S. B., Kim, W. K. & Han, O. S. Efficacy of progressive muscle relaxation training and guided imagery in reducing chemotherapy side effects in patients with breast cancer and in improving their quality of life. *Support Care Cancer* **13**, 826-833 (2005). https://doi.org:10.1007/s00520-005-0806-7
17 Klinkhammer-Schalke, M. *et al.* Direct improvement of quality of life using a tailored quality of life diagnosis and therapy pathway: randomised trial in 200 women with breast cancer. *Br J Cancer* **106**, 826-838 (2012). https://doi.org:10.1038/bjc.2012.4
18 Marchioro, G. *et al.* The impact of a psychological intervention on quality of life in non-metastatic breast cancer. *Eur J Cancer* **32A**, 1612-1615 (1996). https://doi.org:10.1016/0959-8049(96)00134-7
19 Walczak, A. *et al.* Encouraging early discussion of life expectancy and end-of-life care: A randomised controlled trial of a nurse-led communication support program for patients and caregivers. *Int J Nurs Stud* **67**, 31-40 (2017). https://doi.org:10.1016/j.ijnurstu.2016.10.008
20 Petersen, R. W. & Quinlivan, J. A. Preventing anxiety and depression in gynaecological cancer: a randomised controlled trial. *BJOG* **109**, 386-394 (2002). https://doi.org:10.1111/j.1471-0528.2002.01271.x
21 Vanbutsele, G. *et al.* Effect of early and systematic integration of palliative care in patients with advanced cancer: a randomised controlled trial. *Lancet Oncol* **19**, 394-404 (2018). https://doi.org:10.1016/S1470-2045(18)30060-3
22 Schofield, P. *et al.* A tailored, supportive care intervention using systematic assessment designed for people with inoperable lung cancer: a randomised controlled trial. *Psychooncology* **22**, 2445-2453 (2013). https://doi.org:10.1002/pon.3306
23 Qin, X. *et al.* The effect of psychological status on postoperative nutrition, immune function and life quality in elderly patients with gastric cancer. *Biomedical Research (India)* **28**, 3912-3916 (2017).
24 Zhou, L., Tian, Y., Yu, J., Chen, T. & An, F. Effect of at-home cognitive behavior therapy combined with nursing on revised piper fatigue scale, pittsburgh sleep quality index, self-rating anxiety scale and self-rating

depression scale of ovarian cancer patients after chemotherapy. *International journal of clinical and experimental medicine* **13**, 4227-4234 (2020).
25 Northouse, L. L. *et al.* Randomized clinical trial of a family intervention for prostate cancer patients and their spouses. *Cancer* **110**, 2809-2818 (2007). https://doi.org:10.1002/cncr.23114
26 Penedo, F. J. *et al.* Technology-Based Psychosocial Intervention to Improve Quality of Life and Reduce Symptom Burden in Men with Advanced Prostate Cancer: Results from a Randomized Controlled Trial. *Int J Behav Med* **27**, 490-505 (2020). https://doi.org:10.1007/s12529-019-09839-7
27 Penedo, F. J. *et al.* Cognitive behavioral stress management intervention improves quality of life in Spanish monolingual hispanic men treated for localized prostate cancer: results of a randomized controlled trial. *Int J Behav Med* **14**, 164-172 (2007). https://doi.org:10.1007/BF03000188
28 Rodriguez Vega, B. *et al.* Combined therapy versus usual care for the treatment of depression in oncologic patients: a randomized controlled trial. *Psychooncology* **20**, 943-952 (2011). https://doi.org:10.1002/pon.1800
29 Peoples, A. R. *et al.* Effects of cognitive behavioral therapy for insomnia and armodafinil on quality of life in cancer survivors: a randomized placebo-controlled trial. *J Cancer Surviv* **11**, 401-409 (2017). https://doi.org:10.1007/s11764-017-0597-0
30 Parker, P. A. *et al.* The effects of a presurgical stress management intervention for men with prostate cancer undergoing radical prostatectomy. *J Clin Oncol* **27**, 3169-3176 (2009). https://doi.org:10.1200/JCO.2007.16.0036
31 van de Wal, M., Thewes, B., Gielissen, M., Speckens, A. & Prins, J. Efficacy of Blended Cognitive Behavior Therapy for High Fear of Recurrence in Breast, Prostate, and Colorectal Cancer Survivors: The SWORD Study, a Randomized Controlled Trial. *J Clin Oncol* **35**, 2173-2183 (2017). https://doi.org:10.1200/JCO.2016.70.5301
32 Serfaty, M. *et al.* Acceptance and commitment therapy for adults with advanced cancer (CanACT): A feasibility randomised controlled trial. *Psychooncology* **28**, 488-496 (2019). https://doi.org:10.1002/pon.4960
33 Sandsund, C. *et al.* Holistic needs assessment and care plans for women with gynaecological cancer: do they improve cancer-specific health-related quality of life? A randomised controlled trial using mixed methods. *BMJ Support Palliat Care* **10**, e16 (2020). https://doi.org:10.1136/bmjspcare-2016-001207
34 Wu, H. X. *et al.* Psychological and behavioral intervention improves the quality of life and mental health of patients suffering from differentiated thyroid cancer treated with postoperative radioactive iodine-131. *Neuropsychiatr Dis Treat* **12**, 1055-1060 (2016). https://doi.org:10.2147/NDT.S105460
35 Rodin, G. *et al.* Emotion And Symptom-focused Engagement (EASE): a randomized phase II trial of an integrated psychological and palliative care intervention for patients with acute leukemia. *Support Care Cancer* **28**, 163-176 (2020). https://doi.org:10.1007/s00520-019-04723-2
36 Zhou, J. *et al.* Effect of PDCA-based nursing management model on the quality of life and complications of patients with acute leukemia undergoing chemotherapy. *Am J Transl Res* **13**, 3246-3253 (2021).
37 Thomas, M. L. *et al.* A randomized, clinical trial of education or motivational-interviewing-based coaching compared to usual care to improve cancer pain management. *Oncol Nurs Forum* **39**, 39-49 (2012). https://doi.org:10.1188/12.ONF.39-49

38 Fernandez-Rodriguez, C., Villoria-Fernandez, E., Fernandez-Garcia, P., Gonzalez-Fernandez, S. & Perez-Alvarez, M. Effects of Behavioral Activation on the Quality of Life and Emotional State of Lung Cancer and Breast Cancer Patients During Chemotherapy Treatment. *Behav Modif* **43**, 151-180 (2019). https://doi.org:10.1177/0145445517746915
39 Fann, J. R., Fan, M. Y. & Unutzer, J. Improving primary care for older adults with cancer and depression. *J Gen Intern Med* **24 Suppl 2**, S417-424 (2009). https://doi.org:10.1007/s11606-009-0999-4
40 Ohlsson-Nevo, E., Karlsson, J. & Nilsson, U. Effects of a psycho-educational programme on health-related quality of life in patients treated for colorectal and anal cancer: A feasibility trial. *Eur J Oncol Nurs* **21**, 181-188 (2016). https://doi.org:10.1016/j.ejon.2015.10.002
41 Antoni, M. H. *et al.* How stress management improves quality of life after treatment for breast cancer. *J Consult Clin Psychol* **74**, 1143-1152 (2006). https://doi.org:10.1037/0022-006X.74.6.1152
42 Ashing, K. T. & George, M. Exploring the efficacy of a paraprofessional delivered telephonic psychoeducational intervention on emotional well-being in African American breast cancer survivors. *Support Care Cancer* **28**, 1163-1171 (2020). https://doi.org:10.1007/s00520-019-04899-7
43 Esplen, M. J., Warner, E., Boquiren, V., Wong, J. & Toner, B. Restoring body image after cancer (ReBIC): A group therapy intervention. *Psychooncology* **29**, 671-680 (2020). https://doi.org:10.1002/pon.5304
44 Burns, D. S. The effect of the bonny method of guided imagery and music on the mood and life quality of cancer patients. *J Music Ther* **38**, 51-65 (2001). https://doi.org:10.1093/jmt/38.1.51
45 Dos Santos, M. *et al.* Cognitive rehabilitation program to improve cognition of cancer patients treated with chemotherapy: A 3-arm randomized trial. *Cancer* **126**, 5328-5336 (2020). https://doi.org:10.1002/cncr.33186
46 Gaston-Johansson, F. *et al.* Long-term effect of the self-management comprehensive coping strategy program on quality of life in patients with breast cancer treated with high-dose chemotherapy. *Psychooncology* **22**, 530-539 (2013). https://doi.org:10.1002/pon.3031
47 Garssen, B. *et al.* Stress management training for breast cancer surgery patients. *Psychooncology* **22**, 572-580 (2013). https://doi.org:10.1002/pon.3034
48 Hall, S. *et al.* A novel approach to enhancing hope in patients with advanced cancer: a randomised phase II trial of dignity therapy. *BMJ Support Palliat Care* **1**, 315-321 (2011). https://doi.org:10.1136/bmjspcare-2011-000054
49 Napoles, A. M. *et al.* Nuevo Amanecer: results of a randomized controlled trial of a community-based, peer-delivered stress management intervention to improve quality of life in Latinas with breast cancer. *Am J Public Health* **105 Suppl 3**, e55-63 (2015). https://doi.org:10.2105/AJPH.2015.302598
50 Gao, Q., Li, H., Zou, Y., Hou, B. & Liu, L. Effectiveness of a comprehensive post-operative health education program in improving quality of life after gastric cancer surgery. *Ann Palliat Med* **9**, 921-926 (2020). https://doi.org:10.21037/apm.2020.04.14
51 Dieng, M. *et al.* Benefits of a brief psychological intervention targeting fear of cancer recurrence in people at high risk of developing another melanoma: 12-month follow-up results of a randomized controlled trial. *Br J Dermatol* **182**, 860-868 (2020). https://doi.org:10.1111/bjd.17990

52 Karlsen, R. V. *et al.* Couple Counseling and Pelvic Floor Muscle Training for Men Operated for Prostate Cancer and for Their Female Partners: Results From the Randomized ProCan Trial. *Sex Med* **9**, 100350 (2021). https://doi.org:10.1016/j.esxm.2021.100350

53 Boele, F. W. *et al.* Internet-based guided self-help for glioma patients with depressive symptoms: a randomized controlled trial. *J Neurooncol* **137**, 191-203 (2018). https://doi.org:10.1007/s11060-017-2712-5

54 Braeken, A. P. *et al.* Psychosocial screening effects on health-related outcomes in patients receiving radiotherapy. A cluster randomised controlled trial. *Psychooncology* **22**, 2736-2746 (2013). https://doi.org:10.1002/pon.3340

55 Cheung, Y. L., Molassiotis, A. & Chang, A. M. The effect of progressive muscle relaxation training on anxiety and quality of life after stoma surgery in colorectal cancer patients. *Psychooncology* **12**, 254-266 (2003). https://doi.org:10.1002/pon.638

56 van der Hout, A. *et al.* Role of eHealth application Oncokompas in supporting self-management of symptoms and health-related quality of life in cancer survivors: a randomised, controlled trial. *Lancet Oncol* **21**, 80-94 (2020). https://doi.org:10.1016/S1470-2045(19)30675-8

57 Ham, K. *et al.* Preliminary Results From a Randomized Controlled Study for an App-Based Cognitive Behavioral Therapy Program for Depression and Anxiety in Cancer Patients. *Front Psychol* **10**, 1592 (2019). https://doi.org:10.3389/fpsyg.2019.01592

58 Ding, K. *et al.* Managing Cancer and Living Meaningfully (CALM) Intervention on Chemotherapy-Related Cognitive Impairment in Breast Cancer Survivors. *Integr Cancer Ther* **19**, 1534735420938450 (2020). https://doi.org:10.1177/1534735420938450

59 Fillion, L. *et al.* A brief intervention for fatigue management in breast cancer survivors. *Cancer Nurs* **31**, 145-159 (2008). https://doi.org:10.1097/01.NCC.0000305698.97625.95

60 Gonzalez-Hernandez, E. *et al.* Cognitively-Based Compassion Training (CBCT((R))) in Breast Cancer Survivors: A Randomized Clinical Trial Study. *Integr Cancer Ther* **17**, 684-696 (2018). https://doi.org:10.1177/1534735418772095

61 Breitbart, W. *et al.* Individual meaning-centered psychotherapy for the treatment of psychological and existential distress: A randomized controlled trial in patients with advanced cancer. *Cancer* **124**, 3231-3239 (2018). https://doi.org:10.1002/cncr.31539

62 van der Meulen, I. C. *et al.* Long-term effect of a nurse-led psychosocial intervention on health-related quality of life in patients with head and neck cancer: a randomised controlled trial. *Br J Cancer* **110**, 593-601 (2014). https://doi.org:10.1038/bjc.2013.733

63 Turner, J. *et al.* A tiered multidisciplinary approach to the psychosocial care of adult cancer patients integrated into routine care: the PROMPT study (a cluster-randomised controlled trial). *Support Care Cancer* **25**, 17-26 (2017). https://doi.org:10.1007/s00520-016-3382-0

64 Reese, J. B. *et al.* Efficacy of a multimedia intervention in facilitating breast cancer patients' clinical communication about sexual health: Results of a randomized controlled trial. *Psychooncology* **30**, 681-690 (2021). https://doi.org:10.1002/pon.5613

65 Powell, C. B. *et al.* A randomized study of the effectiveness of a brief psychosocial intervention for women attending a gynecologic cancer clinic. *Gynecol Oncol* **111**, 137-143 (2008). https://doi.org:10.1016/j.ygyno.2008.06.024
66 Nikbakhsh, N. *et al.* Citalopram and group psychotherapy in breast cancer patients: A randomized clinical trial. *Med J Islam Repub Iran* **32**, 68 (2018). https://doi.org:10.14196/mjiri.32.68
67 Fang, P., Wu. The effect of psychological support combined with humanistic care on the negative emotions and satisfaction with nursing among gastric cancer surgery patients. (2020).
68 Mihuta, M. E., Green, H. J. & Shum, D. H. K. Web-based cognitive rehabilitation for survivors of adult cancer: A randomised controlled trial. *Psychooncology* **27**, 1172-1179 (2018). https://doi.org:10.1002/pon.4615
69 McLachlan, S. A. *et al.* Randomized trial of coordinated psychosocial interventions based on patient self-assessments versus standard care to improve the psychosocial functioning of patients with cancer. *J Clin Oncol* **19**, 4117-4125 (2001). https://doi.org:10.1200/JCO.2001.19.21.4117
70 Edmonds, C. V., Lockwood, G. A. & Cunningham, A. J. Psychological response to long-term group therapy: a randomized trial with metastatic breast cancer patients. *Psychooncology* **8**, 74-91 (1999). https://doi.org:10.1002/(SICI)1099-1611(199901/02)8:1<74::AID-PON339>3.0.CO;2-K
71 Li, J. & Liu, X. Incremental patient care program decreases anxiety, reduces depression and improves the quality of life in patients with colorectal cancer receiving adjuvant chemotherapy. *Exp Ther Med* **18**, 2789-2798 (2019). https://doi.org:10.3892/etm.2019.7877
72 Elyasi, F. *et al.* Cognitive-Behavioral Therapy and Hypnosis Intervention on Anxiety, Depression, and Quality of Life in Patients with Breast Cancer Undergoing Chemotherapy: A Clinical Trial. *Middle East Journal of Cancer* **12**, 236-248 (2021). https://doi.org:10.30476/mejc.2020.83225.1149
73 Ferguson, R. J. *et al.* Development of CBT for chemotherapy-related cognitive change: results of a waitlist control trial. *Psychooncology* **21**, 176-186 (2012). https://doi.org:10.1002/pon.1878
74 Willems, R. A. *et al.* Short-term effectiveness of a web-based tailored intervention for cancer survivors on quality of life, anxiety, depression, and fatigue: randomized controlled trial. *Psychooncology* **26**, 222-230 (2017). https://doi.org:10.1002/pon.4113
75 Wu, Q., Liu, Q. & Pan, S. Effect of wechat platform based continuity of care on the quality of life and psychosocial competence in postoperative patients with breast cancer. *International journal of clinical and experimental medicine* **14**, 1162-1171 (2021).
76 Yun, Y. H. *et al.* A randomized controlled trial of physical activity, dietary habit, and distress management with the Leadership and Coaching for Health (LEACH) program for disease-free cancer survivors. *BMC Cancer* **17**, 298 (2017). https://doi.org:10.1186/s12885-017-3290-9
77 Zhao, Y. H. & Xu, Y. Effect of Comprehensive Nursing Based on Cognitive Behavior on Psychological Function of Glioma Patients. *Neuropsychiatr Dis Treat* **17**, 777-785 (2021). https://doi.org:10.2147/NDT.S286164

78 Zhao, X. *et al.* Influence of psychological intervention on pain and immune functions of patients receiving lung cancer surgery. *Pak J Med Sci* **32**, 155-159 (2016). https://doi.org:10.12669/pjms.321.8935
79 Rosen, K. D., Paniagua, S. M., Kazanis, W., Jones, S. & Potter, J. S. Quality of life among women diagnosed with breast Cancer: A randomized waitlist controlled trial of commercially available mobile app-delivered mindfulness training. *Psychooncology* **27**, 2023-2030 (2018). https://doi.org:10.1002/pon.4764
80 Ruiz-Vozmediano, J. *et al.* Influence of a Multidisciplinary Program of Diet, Exercise, and Mindfulness on the Quality of Life of Stage IIA-IIB Breast Cancer Survivors. *Integr Cancer Ther* **19**, 1534735420924757 (2020). https://doi.org:10.1177/1534735420924757
81 Von Ah, D. *et al.* Advanced cognitive training for breast cancer survivors: a randomized controlled trial. *Breast Cancer Res Treat* **135**, 799-809 (2012). https://doi.org:10.1007/s10549-012-2210-6
82 Lengacher, C. A. *et al.* Randomized controlled trial of mindfulness-based stress reduction (MBSR) for survivors of breast cancer. *Psychooncology* **18**, 1261-1272 (2009). https://doi.org:10.1002/pon.1529
83 Beatty, L., Koczwara, B. & Wade, T. Evaluating the efficacy of a self-guided Web-based CBT intervention for reducing cancer-distress: a randomised controlled trial. *Support Care Cancer* **24**, 1043-1051 (2016). https://doi.org:10.1007/s00520-015-2867-6
84 Chu, X. Effect of Mindfulness Based Cognitive Therapy on the Mental Health and Quality of Life in Patients with Breast Cancer. (2020). https://doi.org:10.36468/pharmaceutical-sciences.spl.143
85 Berglund, G. *et al.* "Between Men": a psychosocial rehabilitation programme for men with prostate cancer. *Acta Oncol* **46**, 83-89 (2007). https://doi.org:10.1080/02841860600857326
86 Dirksen, S. R. & Epstein, D. R. Efficacy of an insomnia intervention on fatigue, mood and quality of life in breast cancer survivors. *J Adv Nurs* **61**, 664-675 (2008). https://doi.org:10.1111/j.1365-2648.2007.04560.x
87 Dolbeault, S. *et al.* The effectiveness of a psycho-educational group after early-stage breast cancer treatment: results of a randomized French study. *Psychooncology* **18**, 647-656 (2009). https://doi.org:10.1002/pon.1440
88 Aranda, S. *et al.* Meeting the support and information needs of women with advanced breast cancer: a randomised controlled trial. *Br J Cancer* **95**, 667-673 (2006). https://doi.org:10.1038/sj.bjc.6603320
89 Baoyindeligeer, L. Z. Rehabilitation and quality of life improvement of patients with esophageal carcinoma surgery by psychological intervention. (2020).
90 Armes, J., Chalder, T., Addington-Hall, J., Richardson, A. & Hotopf, M. A randomized controlled trial to evaluate the effectiveness of a brief, behaviorally oriented intervention for cancer-related fatigue. *Cancer* **110**, 1385-1395 (2007). https://doi.org:10.1002/cncr.22923
91 Compen, F. *et al.* Face-to-Face and Internet-Based Mindfulness-Based Cognitive Therapy Compared With Treatment as Usual in Reducing Psychological Distress in Patients With Cancer: A Multicenter Randomized Controlled Trial. *J Clin Oncol* **36**, 2413-2421 (2018). https://doi.org:10.1200/JCO.2017.76.5669
92 Schofield, P. *et al.* Nurse-led group consultation intervention reduces depressive symptoms in men with localised prostate cancer: a cluster

randomised controlled trial. *BMC Cancer* **16**, 637 (2016). https://doi.org:10.1186/s12885-016-2687-1
93 Hauffman, A. *et al.* Cocreated internet-based stepped care for individuals with cancer and concurrent symptoms of anxiety and depression: Results from the U-CARE AdultCan randomized controlled trial. *Psychooncology* **29**, 2012-2018 (2020). https://doi.org:10.1002/pon.5489
94 Jelvehzadeh, F., Dogaheh, E. R., Bernstein, C., Shakiba, S. & Ranjbar, H. The effect of a group cognitive behavioral therapy on the quality of life and emotional disturbance of women with breast cancer. *Support Care Cancer* **30**, 305-312 (2022). https://doi.org:10.1007/s00520-021-06421-4
95 Qiu, H. Effects of cognitive behavioral therapy for depression on improving insomnia and quality of life in Chinese women with breast cancer: results of a randomized, controlled, multicenter trial. (2018). https://doi.org:https://doi.org/10.2147%2FNDT.S171297
96 Girgis, A., Breen, S., Stacey, F. & Lecathelinais, C. Impact of two supportive care interventions on anxiety, depression, quality of life, and unmet needs in patients with nonlocalized breast and colorectal cancers. *J Clin Oncol* **27**, 6180-6190 (2009). https://doi.org:10.1200/JCO.2009.22.8718
97 Guan, S. The improvement strategies of psychological intervention nursing on the anxiety and living quality of patients with gynecologic malignancies during postoperative chemotherapy. (2019).
98 Guo, Z. The benefits of psychosocial interventions for cancer patients undergoing radiotherapy. (2013).
99 Heiney, S. P. *et al.* Efficacy of therapeutic group by telephone for women with breast cancer. *Cancer Nurs* **26**, 439-447 (2003). https://doi.org:10.1097/00002820-200312000-00003
100 Henderson, V. P. *et al.* A randomized controlled trial of mindfulness-based stress reduction for women with early-stage breast cancer receiving radiotherapy. *Integr Cancer Ther* **12**, 404-413 (2013). https://doi.org:10.1177/1534735412473640
101 Henderson, V. P. *et al.* The effects of mindfulness-based stress reduction on psychosocial outcomes and quality of life in early-stage breast cancer patients: a randomized trial. *Breast Cancer Res Treat* **131**, 99-109 (2012). https://doi.org:10.1007/s10549-011-1738-1
102 Hoffman, C. J. *et al.* Effectiveness of mindfulness-based stress reduction in mood, breast- and endocrine-related quality of life, and well-being in stage 0 to III breast cancer: a randomized, controlled trial. *J Clin Oncol* **30**, 1335-1342 (2012). https://doi.org:10.1200/JCO.2010.34.0331
103 Johansson, B. *et al.* Health-related quality of life and distress in cancer patients: results from a large randomised study. *Br J Cancer* **99**, 1975-1983 (2008). https://doi.org:10.1038/sj.bjc.6604789
104 Kim, Y. H., Choi, K. S., Han, K. & Kim, H. W. A psychological intervention programme for patients with breast cancer under chemotherapy and at a high risk of depression: A randomised clinical trial. *J Clin Nurs* **27**, 572-581 (2018). https://doi.org:10.1111/jocn.13910
105 Klafke, N. *et al.* The effects of an integrated supportive care intervention on quality of life outcomes in outpatients with breast and gynecologic cancer undergoing chemotherapy: Results from a randomized controlled trial. *Cancer Med* **8**, 3666-3676 (2019). https://doi.org:10.1002/cam4.2196

106 Rahmani, S. & Talepasand, S. The effect of group mindfulness - based stress reduction program and conscious yoga on the fatigue severity and global and specific life quality in women with breast cancer. *Med J Islam Repub Iran* **29**, 175 (2015).

107 Reich, R. R. *et al.* Mindfulness-Based Stress Reduction in Post-treatment Breast Cancer Patients: Immediate and Sustained Effects Across Multiple Symptom Clusters. *J Pain Symptom Manage* **53**, 85-95 (2017). https://doi.org:10.1016/j.jpainsymman.2016.08.005

108 Savard, J. *et al.* Randomized clinical trial on cognitive therapy for depression in women with metastatic breast cancer: psychological and immunological effects. *Palliat Support Care* **4**, 219-237 (2006). https://doi.org:10.1017/s1478951506060305

109 Schellekens, M. P. J. *et al.* Mindfulness-Based Cancer Recovery (MBCR) versus Supportive Expressive Group Therapy (SET) for distressed breast cancer survivors: evaluating mindfulness and social support as mediators. *J Behav Med* **40**, 414-422 (2017). https://doi.org:10.1007/s10865-016-9799-6

110 van den Berg, S. BREATH: Web-Based Self-Management for Psychological Adjustment After Primary Breast Cancer—Results of a Multicenter Randomized Controlled Trial. *Journal of Clinical Oncology* (2015). https://doi.org:10.1200/JCO.2013.54.9386

111 Wang, T. J. *et al.* Efficacy of a self-management program on quality of life in colorectal cancer patients: A randomized controlled trial. *Eur J Oncol Nurs* **67**, 102431 (2023). https://doi.org:10.1016/j.ejon.2023.102431

112 Xia, S. Effect of cognitive behavioral stress management on anxiety, depression, and quality of life in colorectal cancer patients post tumor resection: a randomized, controlled study. *Ir J Med Sci* **192**, 1637-1644 (2023). https://doi.org:10.1007/s11845-023-03405-7

113 Peng, L. *et al.* Effects of an online mindfulness-based intervention on Fear of Cancer Recurrence and quality of life among Chinese breast cancer survivors. *Complement Ther Clin Pract* **49**, 101686 (2022). https://doi.org:10.1016/j.ctcp.2022.101686

114 Kissane, D. W. *et al.* Meaning and Purpose (MaP) therapy in advanced cancer patients: a randomised controlled trial. *Support Care Cancer* **31**, 734 (2023). https://doi.org:10.1007/s00520-023-08189-1

115 Zaman, A. *et al.* Effectiveness of a Tailored Work-Related Support Intervention for Patients Diagnosed with Gastrointestinal Cancer: A Multicenter Randomized Controlled Trial. *J Occup Rehabil* **31**, 323-338 (2021). https://doi.org:10.1007/s10926-020-09920-z

116 Kim, S. H., Choe, Y. H., Cho, Y. U., Park, S. & Lee, M. H. Effects of a partnership-based, needs-tailored self-management support intervention for post-treatment breast cancer survivors: A randomized controlled trial. *Psychooncology* **31**, 460-469 (2022). https://doi.org:10.1002/pon.5828

117 McCusker, J. *et al.* CanDirect: Effectiveness of a Telephone-Supported Depression Self-Care Intervention for Cancer Survivors. *J Clin Oncol* **39**, 1150-1161 (2021). https://doi.org:10.1200/jco.20.01802

118 Lee, J. T., Lee, Y. H. & Chang, Y. P. Mindfulness Stress Management for Female Cancer Survivors Facing the Uncertainty of Disease Progression: A Randomized Controlled Study. *Int J Environ Res Public Health* **19** (2022). https://doi.org:10.3390/ijerph19084497

119 Lu, Z. *et al.* Early Interdisciplinary Supportive Care in Patients With Previously Untreated Metastatic Esophagogastric Cancer: A Phase III Randomized Controlled Trial. *J Clin Oncol* **39**, 748-756 (2021). https://doi.org:10.1200/jco.20.01254
120 Kirkegaard, A. M. *et al.* Effects on long-term survival of psychosocial group intervention in early-stage breast cancer: follow-up of a randomized controlled trial. *Acta Oncol* **62**, 422-428 (2023). https://doi.org:10.1080/0284186x.2023.2203329
121 Oner Cengiz, H., Bayir, B., Sayar, S. & Demirtas, M. Effect of mindfulness-based therapy on spiritual well-being in breast cancer patients: a randomized controlled study. *Support Care Cancer* **31**, 438 (2023). https://doi.org:10.1007/s00520-023-07904-2
122 Seliniotaki, T. *et al.* Effects of an 8-Week Stress Management Program in Women with Breast Cancer: A Randomized Controlled Trial. *Adv Exp Med Biol* **1339**, 85-95 (2021). https://doi.org:10.1007/978-3-030-78787-5_12
123 Anderson, K. O. *et al.* Brief cognitive-behavioral audiotape interventions for cancer-related pain: Immediate but not long-term effectiveness. *Cancer* **107**, 207-214 (2006). https://doi.org:10.1002/cncr.21964
124 Bjorneklett, H. G. *et al.* A randomised controlled trial of support group intervention after breast cancer treatment: results on anxiety and depression. *Acta Oncol* **51**, 198-207 (2012). https://doi.org:10.3109/0284186X.2011.610352
125 Borosund, E. *et al.* Results from a randomized controlled trial testing StressProffen; an application-based stress-management intervention for cancer survivors. *Cancer Med* **9**, 3775-3785 (2020). https://doi.org:10.1002/cam4.3000
126 Carbajal-Lopez, E. B., Juarez-Garcia, D. M., Espinoza-Velazco, A. & Calderillo-Ruiz, G. Internet-Delivered Cognitive Behavioral Therapy and Psychoeducation Program for Patients with Gastrointestinal Stromal Tumors. *J Cancer Educ* **37**, 668-674 (2022). https://doi.org:10.1007/s13187-020-01866-3
127 Rahmani, S., Talepasand, S. & Ghanbary-Motlagh, A. Comparison of effectiveness of the metacognition treatment and the mindfulness-based stress reduction treatment on global and specific life quality of women with breast cancer. *Iran J Cancer Prev* **7**, 184-196 (2014).
128 Carlson LE, D. R., Stephen J, Faris P, Tamagawa R, Drysdale E, Speca M. Randomized controlled trial of Mindfulness-based cancer recovery versus supportive expressive group therapy for distressed survivors of breast cancer. (2013). https://doi.org:https://doi.org/10.1200/jco.2012.47.5210
129 Ell, K. *et al.* Randomized controlled trial of collaborative care management of depression among low-income patients with cancer. *J Clin Oncol* **26**, 4488-4496 (2008). https://doi.org:10.1200/JCO.2008.16.6371
130 Lapid, M. I. *et al.* Improving the quality of life of geriatric cancer patients with a structured multidisciplinary intervention: a randomized controlled trial. *Palliat Support Care* **5**, 107-114 (2007). https://doi.org:10.1017/s1478951507070174
131 Lu, J. *et al.* Effectiveness of an Intervention to Promote Self-Efficacy on Quality of Life of Patients with Nasopharyngeal Carcinoma of the Zhuang Tribe Minority in Guangxi, China: A Prospective Study. *Med Sci Monit* **23**, 4077-4086 (2017). https://doi.org:10.12659/msm.903205

132 Murphy, M. J. *et al.* Randomised controlled trial of internet-delivered cognitive behaviour therapy for clinical depression and/or anxiety in cancer survivors (iCanADAPT Early). *Psychooncology* **29**, 76-85 (2020). https://doi.org:10.1002/pon.5267

133 Pettiford, J. *et al.* A Bio-Psychosocial Intervention Program for Improving Quality of Life in Breast Cancer Survivors - Final Outcome of a Prospective Randomized Trial. *Breast J* **23**, 537-544 (2017). https://doi.org:10.1111/tbj.12786

134 Poort, H. *et al.* Cognitive behavioral therapy or graded exercise therapy compared with usual care for severe fatigue in patients with advanced cancer during treatment: a randomized controlled trial. *Ann Oncol* **31**, 115-122 (2020). https://doi.org:10.1016/j.annonc.2019.09.002

135 Ramirez, A. G. *et al.* Assessing the effect of patient navigator assistance for psychosocial support services on health-related quality of life in a randomized clinical trial in Latino breast, prostate, and colorectal cancer survivors. *Cancer* **126**, 1112-1123 (2020). https://doi.org:10.1002/cncr.32626

136 Rottmann, N. *et al.* No improvement in distress and quality of life following psychosocial cancer rehabilitation. A randomised trial. *Psychooncology* **21**, 505-514 (2012). https://doi.org:10.1002/pon.1924

137 Sharpe, M. *et al.* Integrated collaborative care for comorbid major depression in patients with cancer (SMaRT Oncology-2): a multicentre randomised controlled effectiveness trial. *Lancet* **384**, 1099-1108 (2014). https://doi.org:10.1016/S0140-6736(14)61231-9

138 Sohl, S. J., Dietrich, M. S., Wallston, K. A. & Ridner, S. H. A randomized controlled trial of expressive writing in breast cancer survivors with lymphedema. *Psychol Health* **32**, 826-842 (2017). https://doi.org:10.1080/08870446.2017.1307372

139 Ploos van Amstel, F. K. *et al.* Does a regular nurse-led distress screening and discussion improve quality of life of breast cancer patients treated with curative intent? A randomized controlled trial. *Psychooncology* **29**, 719-728 (2020). https://doi.org:10.1002/pon.5324

140 Yanez, B. *et al.* Feasibility, acceptability, and preliminary efficacy of a technology-assisted psychosocial intervention for racially diverse men with advanced prostate cancer. *Cancer* **121**, 4407-4415 (2015). https://doi.org:10.1002/cncr.29658

141 Wenzel, L. *et al.* Psychosocial telephone counseling for survivors of cervical cancer: results of a randomized biobehavioral trial. *J Clin Oncol* **33**, 1171-1179 (2015). https://doi.org:10.1200/JCO.2014.57.4079

142 Urech, C. *et al.* Web-Based Stress Management for Newly Diagnosed Patients With Cancer (STREAM): A Randomized, Wait-List Controlled Intervention Study. *J Clin Oncol* **36**, 780-788 (2018). https://doi.org:10.1200/JCO.2017.74.8491

143 Lerman, R., Jarski, R., Rea, H., Gellish, R. & Vicini, F. Improving symptoms and quality of life of female cancer survivors: a randomized controlled study. *Ann Surg Oncol* **19**, 373-378 (2012). https://doi.org:10.1245/s10434-011-2051-2
